# LINC01198 activates Hippo signaling to stimulate IL-1β autocrine for driving vemurafenib resistance by associating with TAOK1/2 in melanoma

**DOI:** 10.1038/s41420-025-02773-6

**Published:** 2025-10-27

**Authors:** Jieyu Liu, Xiaoting Liang, Ke Wang, Chunting Zhang, Can Li, Lei Zhao, Yanjie Kuang, Min Wang, Jun Liu, Liang Zhou, Li Ma

**Affiliations:** 1 Institute of Molecular Immunology, School of Laboratory Medicine and Biotechnology, Southern Medical University, Guangzhou, China; 2 Key Laboratory of Infectious Diseases Research in South China, Ministry of Education, Southern Medical University, Guangzhou, China; 3 Department of Toxicology, Guangdong Provincial Key Laboratory of Tropical Disease Research, School of Public Health, Southern Medical University, Guangzhou, China; 4 MOE Key Laboratory of Coal Environmental Pathogenicity and Prevention, Shanxi Key Laboratory of Environmental Health Impairment and Prevention, School of Public Health, Shanxi Medical University, Taiyuan, 030001 China; 5 Department of Occupational Health and Occupational Medicine, Guangdong Provincial Key Laboratory of Tropical Disease Research, School of Public Health, Southern Medical University, Guangzhou, China; 6 State Key Laboratory of Organ Failure Research, Division of Nephrology, Nanfang Hospital, Southern Medical University, Guangzhou, China; 7 Guangdong Basic Research Center of Excellence for Integrated Traditional and Western Medicine for Qingzhi Diseases, Southern Medical University, Guangzhou, China

**Keywords:** Targeted therapies, Long non-coding RNAs

## Abstract

Vemurafenib (VEM) is an important targeted drug for treating melanoma harboring BRAF-V600E mutation. Despite its remarkable curative efficacy in early clinical treatment, most patients developed drug resistance within one year. Nevertheless, the critical factors driving vemurafenib resistance and mechanisms leading to treatment failure in melanoma are debating and inconclusive. In this study, we established vemurafenib-resistance melanoma cell strain together with acute vemurafenib treatment and characterized LINC01198 as the only one LncRNA up-regulated in both of stable vemurafenib-resistant and acute vemurafenib-treated melanoma cells. Functionally, loss of LINC01198 significantly compromised melanoma resistance against vemurafenib. Mechanistically, LINC01198 directly associates with TAOK1/2 to inhibit TAOK1/2 phosphorylation and thereby elicits Hippo signaling through TAOK/LATS axis, which redistributes YAP/TAZ into nucleus and promotes the expression and secretion of IL-1β to support vemurafenib resistance in melanoma. Our study not only identifies LINC01198 as a potent indicator and critical factor for driving vemurafenib resistance, but also suggests a series of therapeutic targets for tackling vemurafenib resistance in melanoma.

## Introduction

Cutaneous melanoma (CM, hereafter referred to simply as melanoma) originates from mutated melanocytes. Although melanoma accounts for only 1% of cutaneous cancer, it leads to the highest mortality of 17.5% among cutaneous cancers owing to its high degree of malignancy and rapid development [1, 2]. The incidence and mortality of melanoma keep increasing globally [3]. Melanoma is mainly driven by genetic mutations, especially BRAFV600E mutation [4, 5]. Given the essential roles of mutant BRAF in melanoma initiation, drugs targeting BRAF mutation were developed and bring great benefits to patients with unresectable and metastatic melanoma, such as Vemurafenib [6, 7]. However, patients may develop various drug resistance within one year after treatment with vemurafenib, which seriously compromises the therapeutic efficacy and life quality of patients [8, 9].

Targeted drug resistance may be induced by further mutation of target gene, reactivation of downstream pathway, activation of bypass signaling, or enhanced cell plasticity [10]. Reports have shown that Hippo-YAP/TAZ signaling is important for induction of resistance against targeted therapy, chemotherapy, radiotherapy and immunotherapy [11–13]. Hippo pathway is an evolutionarily conserved signaling cascade and regulates numerous biological processes including cell growth and fate decision, organ size control and regeneration. The core axis in Hippo pathway contains a kinase cascade including MST1/2 and LATS1/2, as well as downstream transcriptional coactivators YAP and TAZ [14]. In parallel to MST1/2, additional kinases including MAP4K1/2/3/5, MAP4K4/6 and TAO kinases (TAOK1/2/3) can also directly phosphorylate LATS1/2 and thus involve in modulating YAP/TAZ. Activation of YAP/TAZ belongs to known bypass mechanisms involved in EGFR and RAS/MAPK blockades modulating cytoskeleton remodeling, apoptosis, DNA damage and cytokines production [15]. Yet, as an alternative bypass mechanism, how Hippo pathway is activated is still debated and inconclusive, especially in melanoma.

Long noncoding RNAs (LncRNAs) are a group of noncoding transcripts with lengths exceeding two hundred nucleotides that play diverse roles in pathophysiological processes. Notably, LncRNAs are deeply involved in drug resistance related cell cycle [16], pyroptosis [17], drug efflux system [18], epithelial-mesenchymal transition [19] and generation of cancer stem cells [20, 21]. For examples, LncRNA EIF3J-DT induces chemoresistance of gastric cancer via activation of autophagy [22]. LncRNA DIO3OS induces glycolysis-dominant metabolic reprogramming to promote aromatase inhibitor resistance in breast cancer [23]. Although LncRNAs are important in tumor drug resistance, the roles and mechanisms of LncRNAs in melanoma drug resistance are still underexplored.

Cytokines are able to modulate cell growth, metastasis and drug resistance in tumor microenvironment [24]. Reports have shown complicated cross communications in tumor microenvironment mediated by cytokines. Communicable cytokines were traditionally thought to be dominantly secreted from immune cells and act on tumor cells. Yet, cytokines can also be secreted from tumor cells themselves. For example, as one of the classic inflammatory factors, IL-1β not only plays an important role in immune response, but also influences the oncogenesis and development of tumors. Tumor cell-derived IL-1β promotes desmoplasia and immune suppression during pancreatic cancer progression [25]. Endogenous IL-1β from breast cancer cells drives metastasis and colonization in bone microenvironment [26]. However, the influences of IL-1β on tumor cell themselves are often overlooked and remains largely unknown especially for tumor-derived IL-1β.

In this study, we sought to explore key factors and mechanisms driving drug resistance against vemurafenib treatment in BRAF-mutated melanoma. LINC01198 was upregulated upon vemurafenib treatment and sustained higher expression in vemurafenib-resistant melanoma cells. LINC01198 interacts with TAOK1/2 and inhibits their phosphorylation, thereby attenuating the downstream kinase cascade. This activation of Hippo signaling promotes YAP/TAZ nuclear translocation, enhancing the production and secretion of the tumor-derived cytokine IL-1β, which contributes to vemurafenib resistance in melanoma.

## Results

### LINC01198 is induced by vemurafenib treatment and highly-expressed in vemurafenib-resistant melanoma cells

**Fig. 1:**
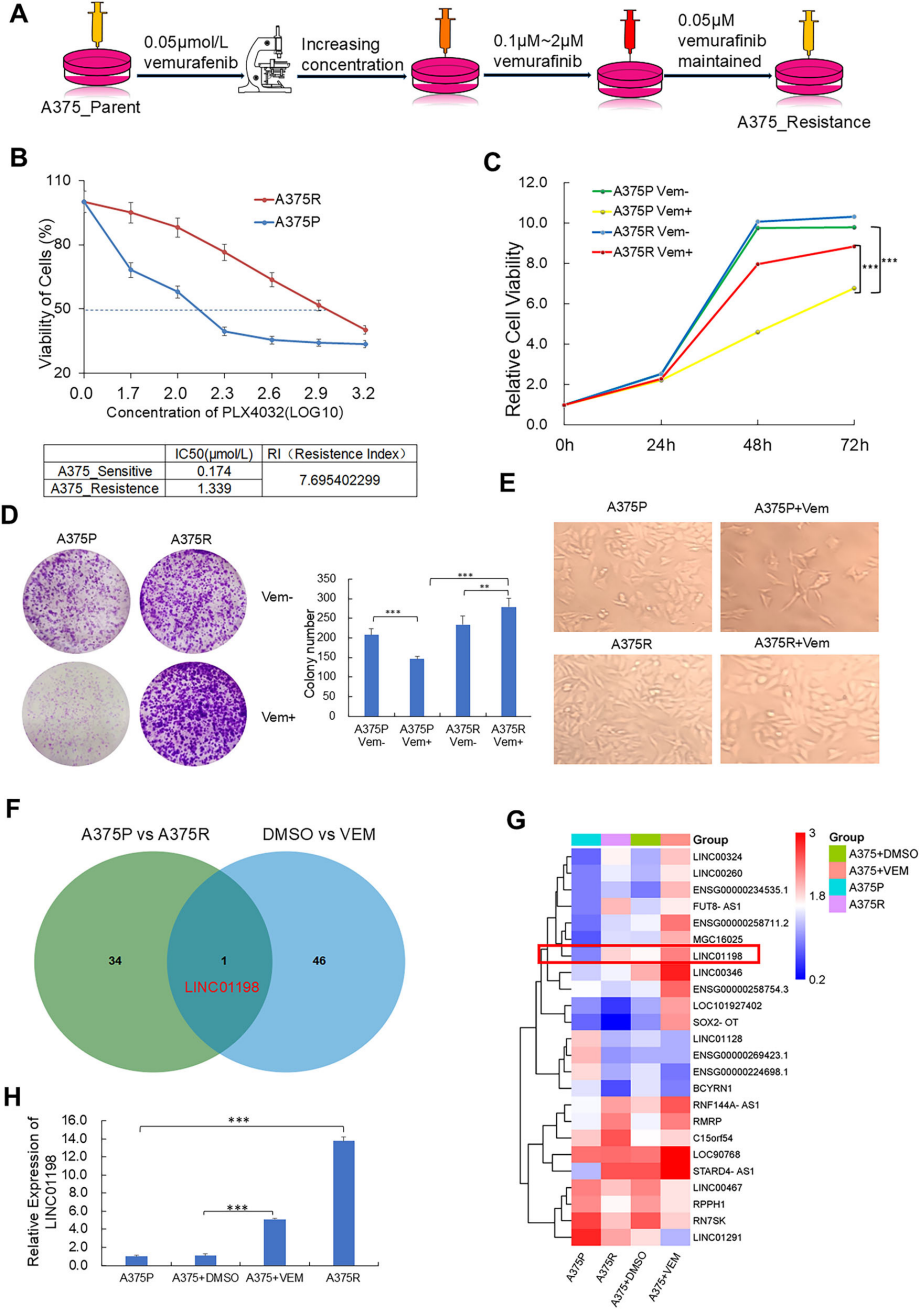
LINC01198 is induced by vemurafenib treatment and highly-expressed in vemurafenib-resistant melanoma cells. **A** The flow chart indicating the process for constructing vemurafenib-resistant A375 cell strain. **B** The IC50 of A375 and A375R cells after treated with DMSO or VEM for 72 h were measured by CCK-8. **C** The viabilities of vemurafenib-resistant (A375R) and parental A375 cells exposed to indicated VEM concentrations were detected by CCK-8. **D** Colony formation assay performed in cells from the two groups 14 d after being treated with DMSO or 2 μM VEM (left). The statistic result was shown on the right. **E** The morphology of A375R and A375P treated with DMSO or 2 μM VEM for 48 h. **F** Venn analysis of upregulated LncRNAs from two transcriptomic sequencing pairs: pair 1 (A375R relative to A375P) and pair 2 (A375 cells treatment with 2 μM VEM relative to A375 cells with mock treatment). **G** The heatmap of differentially expressed LncRNAs from the above two transcriptomic sequencing pairs. **H** RNA expression levels of LINC01198 were verified in A375P, A375R and A375 cells with mock and 2 μM VEM treatments for 48 h were detected by RT-qPCR assay. Data are presented as the means ± SD, and *p* values are determined using two-tailed Student’s *t-*test. ** *p* < 0.01; *** *p* < 0.001; ns, no significance.

To explore key factors driving vemurafenib-resistance in melanoma, vemurafenib-resistant A375 melanoma cells (A375R) were generated by dose-escalating treatments of vemurafenib in A375 melanoma cells (Fig. 1A). CCK-8 assays were performed to determine the IC50 of vemurafenib-resistant cells together with parental cells (vemurafenib-sensitive cells) and cell viability following vemurafenib treatments. In comparison with parental cells, IC50 of A375R cells is significantly increased (resistance index, RI > 5) and the viability of A375R cells were significantly higher than that of parental A375 cells after vemurafenib treatment (Fig. 1B–D). Vemurafenib treatment also caused significant morphological changes in parental A375 cells, resulting in an elongated shape. On the contrary, with the same dose treatment of vemurafenib, vemurafenib-resistant cells A375R did not undergo morphological change (Fig. 1E). Such results suggested that we have successfully established cell stain with resistance against vemurafenib. To identify key factors responsible for vemurafenib resistance, we performed two pairs of transcriptomic sequencing. One pair for A375 cells treated with control solvent or 2 μM vemurafenib to explore responsive factors after acute vemurafenib treatment and the other pair for A375R cells with parental A375 cells (A375P) to identified factors involved in persistent vemurafenib resistance in melanoma cells. There were 35 significantly-upregulated LncRNAs (*p* < 0.05, fold change > 1.5) identified from A375P and A375R pair, while 47 were screened out from acute vemurafenib-treatment pair. Interestingly, Venn analysis indicated LINC01198 was the only one LncRNA upregulated in both of the above two compared pairs (Fig. 1F). Clustering heat map analysis also showed that LINC01198 was one of the top 10 among the differentially-expressed genes (Fig. 1G). Therefore, LINC01198 was chosen for further investigation. First, qPCR was conducted and verified the significant upregulations of LINC01198 in both vemurafenib-resistant and acute vemurafenib-treated melanoma cells (Fig. 1H). According to TCGA database, the expression levels of LINC01198 in most cancers were extremely low but significantly-higher expressed in melanoma compared with normal tissues (Supplementary Fig. S1A, B), suggesting higher expression of LINC01198 may endow special function in melanoma. In addition, migration assay showed the migratory capacity of A375R cells is significantly higher than that of A375P cells. Additionally, the migratory ability of melanoma cells was notably reduced after LINC01198 knockdown (Supplementary Fig. S1C). The above results indicated that LINC01198 might be related to vemurafenib resistance in melanoma.

### LINC01198 promotes melanoma resistance against vemurafenib

**Fig. 2:**
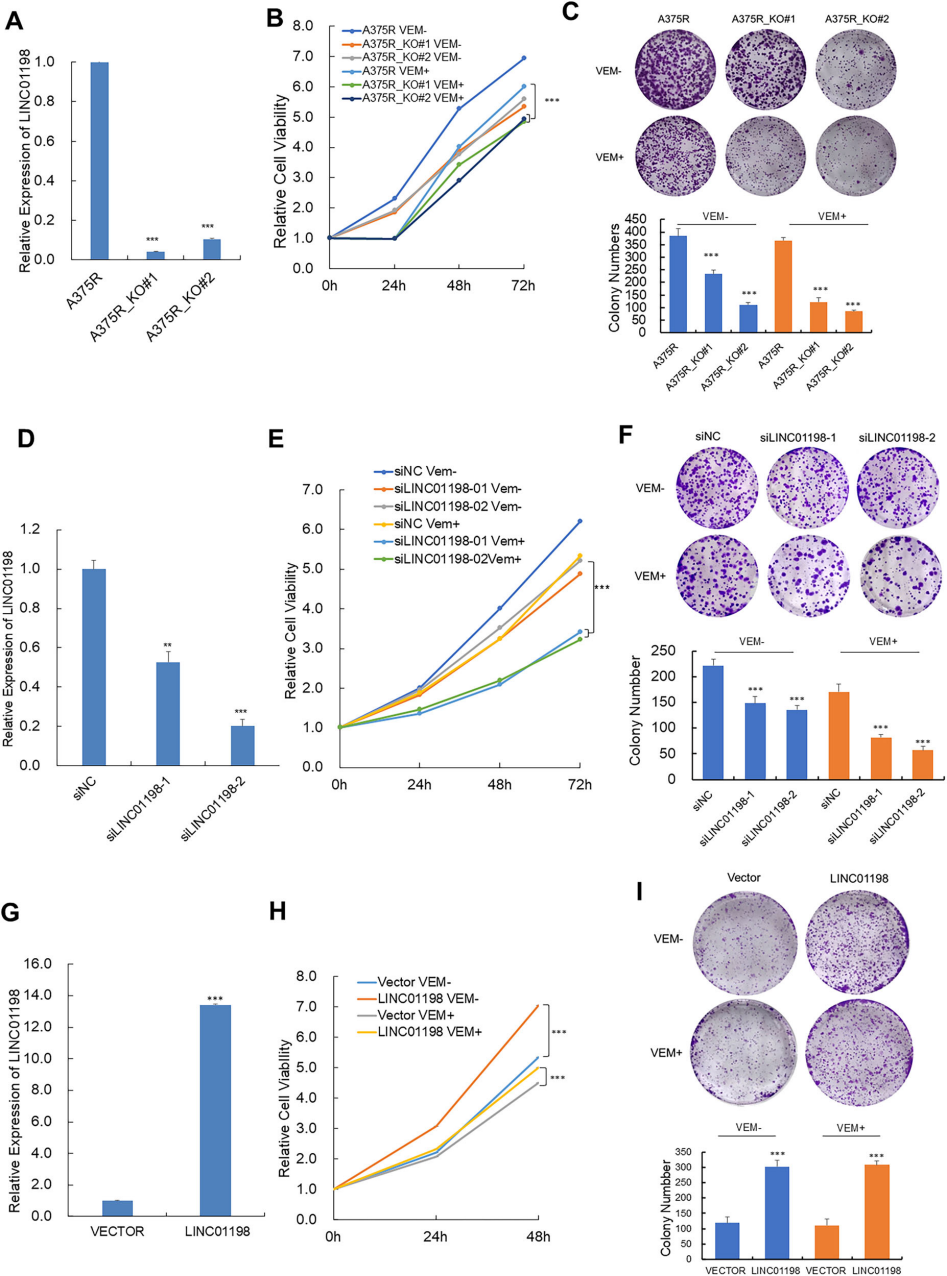
LINC01198 promotes target therapy resistance. **A** The RNA levels of LINC01198 in A375R and A375R cells with LINC01198 knockout. Measurements of **B** relative cell activity by CCK-8 assay and **C** colony formation assay in A375R and A375R with LINC01198 knockout. **D** LINC01198 expression was detected by qPCR after treating with siNC and siLINC01198 in A375R cells. Measurements of **E** relative cell activity by CCK-8 assay and **F** colony formation assay in A375R cells treated with siNC and siLINC01198 in A375R cells. **G** The RNA levels of LINC01198 after overexpression of LINC01198 in A375R. Measurements of **H** cell proliferation by CCK-8 assay and **I** colony formation assay after overexpression of LINC01198 in A375R cells. Data are presented as the means ± SD, and the *p* values are determined using two-tailed Student’s *t* test. * *p* < 0.05; ** *p* < 0.01; *** *p* < 0.001.

To explore the roles of LINC01198 in vemurafenib resistance in melanoma, LINC01198 was knocked out in A375R melanoma cell lines using CRISPER-Cas9 (Supplementary Fig. S2) and the knockout efficacy was verified by qPCR (Fig. 2A). CCK-8 and colony forming assays were conducted and showed both of knockout of LINC01198 significantly sensitized vemurafenib-resistant A375 cells to vemurafenib treatment as evidenced by decreased cell viability and less formed cell colonies (Fig. 2B, C). At the same time, we also performed RNA interference to silence LINC01198 expression (Fig. 2D). The same results were observed upon knockdown of LINC01198 (Fig. 2E, F). On the contrary, overexpression of LINC01198 in A375R cells led to enhanced resistance against vemurafenib (Fig. 2G–I). Collectively, our data demonstrated LINC01198 plays critical role in promoting vemurafenib resistance in melanoma.

### Transcriptomic sequencing reveals LINC01198 positively regulates IL1B expression

**Fig. 3:**
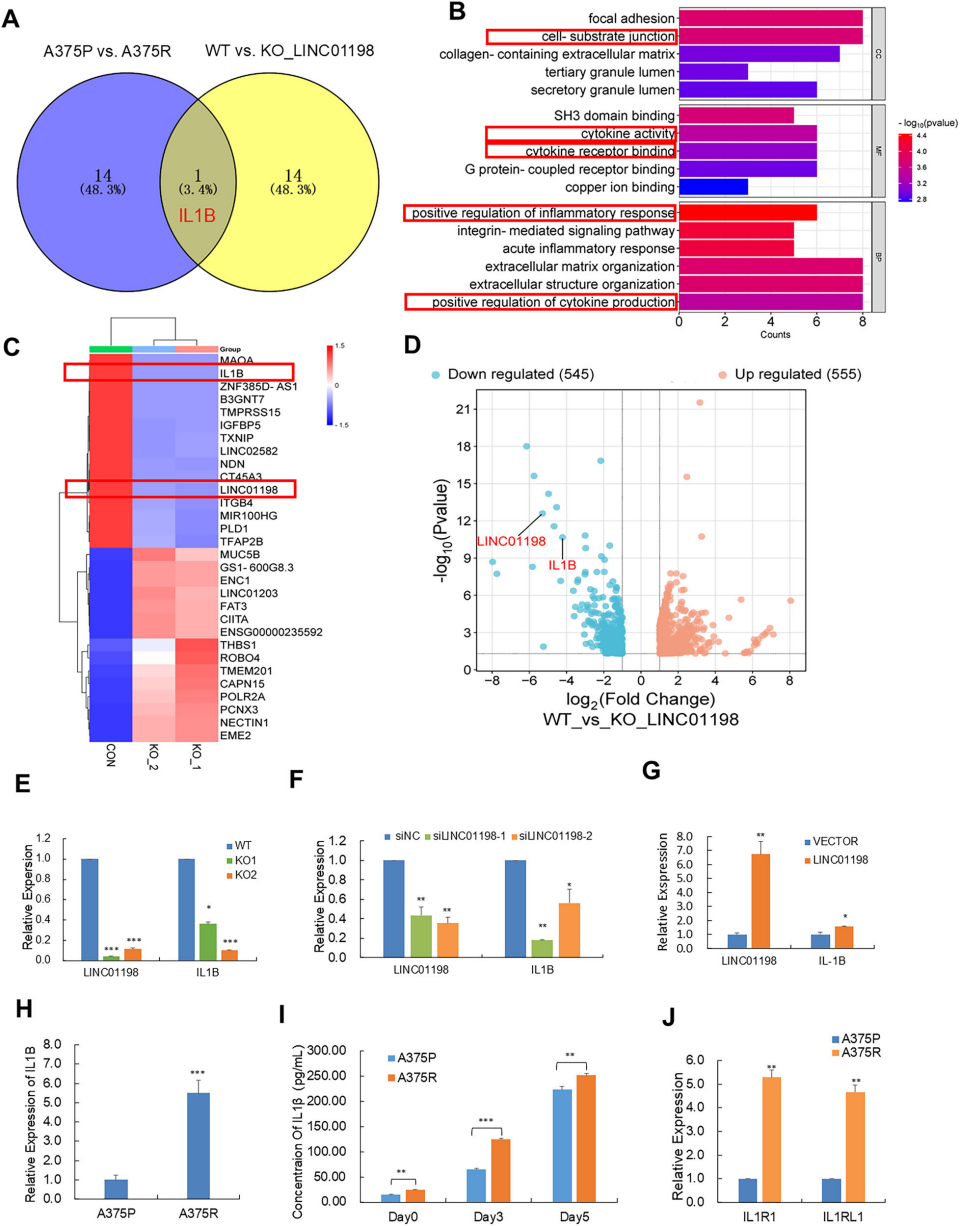
Transcriptomic sequencing reveals LINC01198 positively regulates cytokine IL-1β expression. **A** Venn analysis was performed to find common upregulated factors from three RNA-Seq comparable pairs: A375P VS. A375R, A375P with mock treatment VS. A375P treated with 2 μM VEM, wild type LINC01198 VS. LINC01198_KO strains. **B** Gene Ontology (GO) analysis of the differentially-expressed genes in A375R cells. Cytokine related signaling pathways are highlighted. **C** The heatmap of differentially expressed LncRNAs between A375R cells and A375R with LINC01198 knockout. **D** Differentially expressed genes between A375R cells and A375R KO_LINC01198 were determined by RNA-Seq and shown by volcano plot. LINC01198 and IL1B were shown by lead marks. The expression levels of LINC01198 and IL1B in melanoma cells were determined by qPCR after LINC01198 knockout **E**, knockdown **F** and LINC01198 overexpression **G**. **H** The expression levels of IL1B in A375P and A375R cells were detected by qPCR. **I** Levels of mature IL-1β in culture medium of A375P and A375R cells were measured by ELISA assay. **J** The expression levels of IL1R1 and IL1RL1 in A375P and A375R cells were detected by qPCR. Data are presented as means ± SD, and *p* values are determined using two-tailed Student’s *t-*test. * *p* < 0.05; ** *p* < 0.01; *** *p* < 0.001.

To probe the function executed by LINC01198 in melanoma resistance against vemurafenib, we analyzed differentially expressed mRNAs in the sequencing data above to explore the key downstream effectors of LINC01198 in melanoma. Based on the criteria (fold change > 1.5, *p* < 0.05), Venn analysis between the top 15 differentially-upregulated coding genes from vemurafenib-resistant group and the top 15 significantly downregulated genes after LINC01198 knockout, which identified only common gene IL1B (Fig. 3A). The significance of IL1B was also supported by the significant enrichment of cytokine-related pathways through GO analysis of the vemurafenib-resistant group (Fig. 3B). Importantly, IL1B was pronouncedly downregulated in response to LINC01198 knockout as evidenced by volcano plots and heatmaps (Fig. 3C, D). The correlated expression pattern between LINC01198 and IL1B was further confirmed by detecting IL1B expression in both RNA and protein levels after LINC01198 knockout and knockdown (Fig. 3E, F), while overexpression of LINC01198 in vemurafenib-resistant A375R cells led to significant upregulation of IL1B (Fig. 3G). In addition, IL1B was significantly upregulated (Fig. 3H) together with corresponding higher levels of IL1B protein product, cytokine IL-1β, in culture medium in vemurafenib-resistant cells compared with parental A375P cells (Fig. 3I). Interestingly, the expression of IL-1β receptor was also coordinately upregulated in A375R cells (Fig. 3J), which suggests greater responsiveness to autocrine IL-1β in A375R cells than in A375P cells.

### IL-1β promoted vemurafenib resistance through enhancing melanoma cell viability

**Fig. 4:**
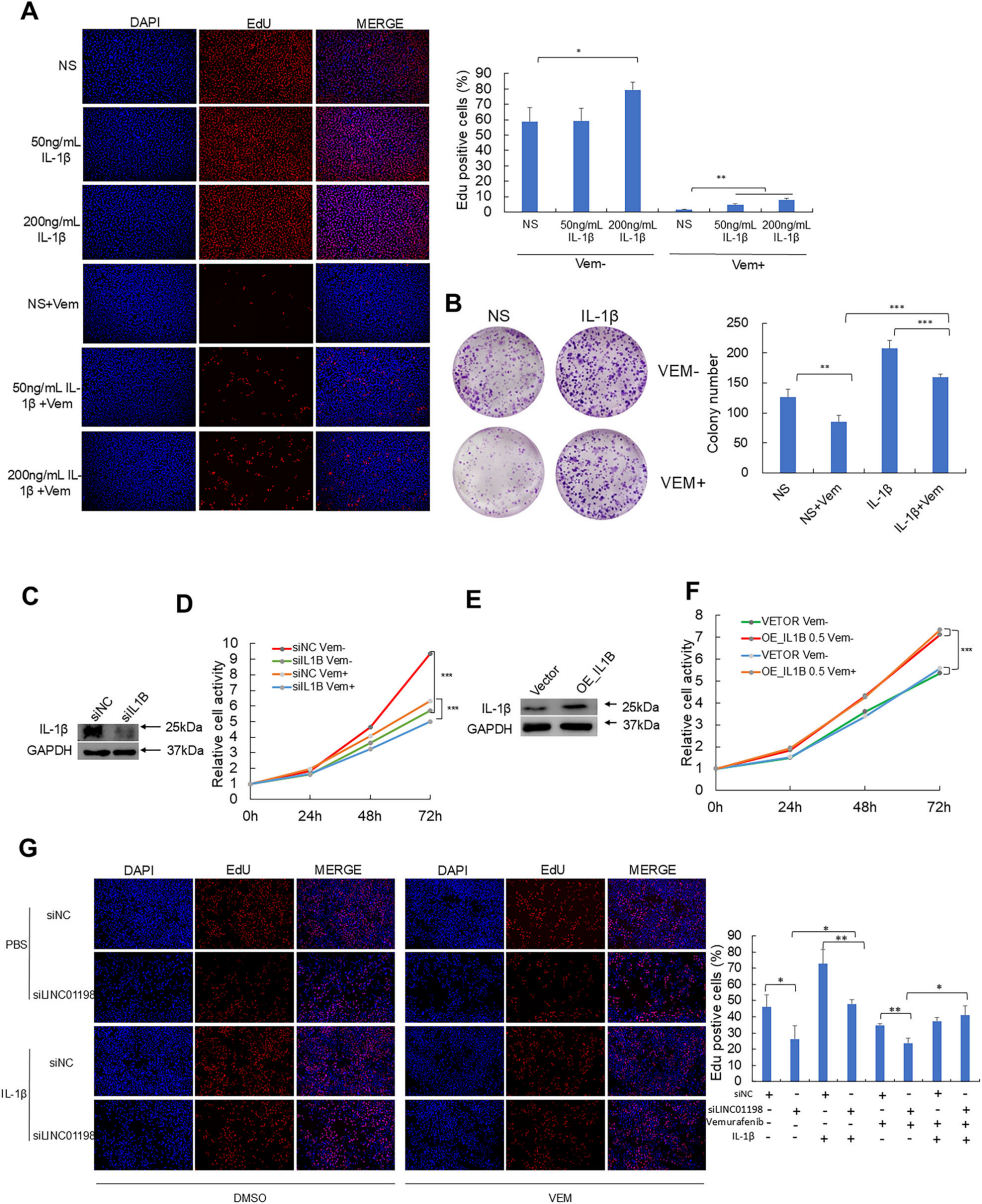
IL-1β promoted vemurafenib resistance through enhancing melanoma cell viability. **A** EdU assays were conducted after treatments with vemurafenib together with 50 ng/mL or 200 ng/mL of IL-1β respectively. **B** Colony formation assay in A375R cells treated with vemurafenib and/or IL-1β. The level of IL-1β was detected by Western blot after IL1B knockdown **C** and overexpression **E**. Measurements of relative cell viability by CCK-8 assay in A375R cells treated with 2 μM vemurafenib after IL1B knockdown **D** and overexpression **F**. **G** EdU assay detected cell viabilities after treatments with vemurafenib and IL-1β or LINC01198 knockdown. Data are presented as the means ± SD and *p* values are determined using two-tailed Student’s *t-*test. *, *p* < 0.05; **, *p* < 0.01; ***, *p* < 0.001.

To validate the role of IL-1β induced by vemurafenib treatment in melanoma, we evaluated the cell viability in response to various levels of IL-1β. IL-1β dose-dependently enhanced cell viability in A375R melanoma cells, which is also supported by EdU cell proliferation and colony formation assays (Fig. 4A, B). Previous reports proposed IL-1β mainly secreted by immune cells in tumor microenvironment could support tumor growth [27]. We would like to know whether IL-1β secreted by melanoma cells themselves also promote their own viability? To verify the role of tumor-derived IL-1β in vemurafenib-resistant melanoma, we knocked down IL1B in A375R cells which were verified by Western blot (Fig. 4C). CCK-8 assay illustrated loss of IL1B significantly inhibited the viability of vemurafenib-resistant A375R melanoma cells, while overexpression of IL1B strongly increased the viability of vemurafenib-resistant A375R cells (Fig. 4D). The results of IL1B overexpression are shown in Fig. 4E and Fig. 4F. Furthermore, the pro-survival effect of IL-1β was further validated in A375R cells, during which the addition of IL-1β restored the compromised cell viability after LINC01198 depletion in response to vemurafenib treatment using EdU assay (Fig. 4G). Collectively, we have demonstrated both of exogenous recombinant human IL-1β and melanoma cell-secreted IL-1β can promote the viability of vemurafenib-resistant A375R melanoma cells against vemurafenib.

### LINC01198 modulates Hippo signaling through TAOK1/2-elicited kinase cascade

**Fig. 5:**
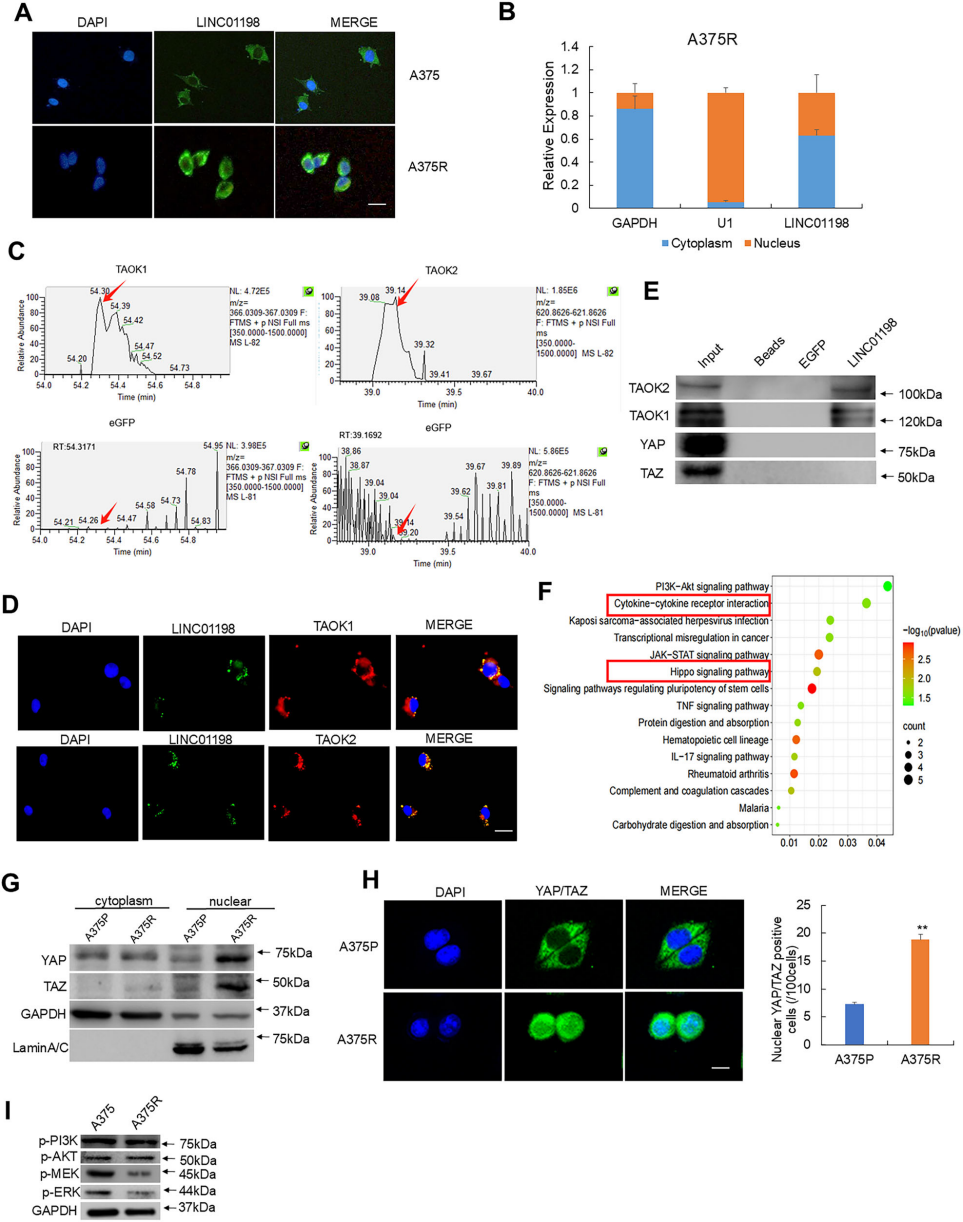
LINC01198 colocalizes with TAOK1/2 and potentially modulates Hippo pathway. **A** Visualization of LINC01198 in A375R and A375P cells by RNA fluorescence in situ hybridization (FISH). Scale bars, 100 μm. **B** After fractionation of cytoplasmic and nuclear portions of A375R cells, qRT-PCR was performed to detect the ratio of LINC01198 in cytoplasm and nucleus. **C** Arrows indicate the identified TAOK1 and TAOK2 peptide peaks in LINC01198-pulldown sample, which are lack in control EGFP RNA sample. **D** Biotin-labeled LINC01198 transcript was used to retrieve interacting protein partners by RNA pulldown with beads only and EGFP RNA as controls. The resulting protein mix from A375R cells was applied to detect TAOK1 and TAOK2 by immunofluorescence and **E** Western blot. **F** Kyoto Encyclopedia of Genes and Genomes (KEGG) pathway analysis of the differentially-expressed genes between A375P and A375R. “Cytokine-cytokine receptor interaction” and “Hippo signaling pathway” were highlighted. Color bars at the right represent gene clusters established through k-means clustering. **G** YAP and TAZ levels in cytoplasm and nucleus were detected by Western blot in A375R and A375P cells. **H** Detection of YAP-GFP and TAZ-GFP subcellular localization in A375R and A375P cells. Nuclear/cytoplasmic fluorescence ratios were calculated. Scale bar: 100 μm. **I** p-PI3K, p-AKT and GAPDH were detected by Western blot in A375R cells and A375P cells. Data are presented as the means ± SD, and *p* values are determined using two-tailed Student’s *t-*test. * *p* < 0.05; ** *p* < 0.01; *** *p* < 0.001.

LncRNAs exert their biological functions mainly through their interacting protein partners and rely on their subcellular localization in cells. To explore how LINC01198 influences IL1B expression, we first determined the subcellular localization of LINC01198 in vemurafenib-resistant melanoma cells by fluorescent in situ hybridization (FISH) and nuclear-cytoplasmic fractionation. The results indicated LINC01198 localized mainly in cytoplasm while smaller portion of LINC01198 was apparently in nucleus (Fig. 5A, B). Further, we conducted an RNA pulldown assay using in vitro-transcribed full-length of LINC01198 accompanied with control EGFP RNA. The specific binding proteins of LINC01198 was identified using high-performance liquid chromatography-mass spectrometry (HPLC-MS). Among the identified proteins, TAOK1 and TAOK2 were found to be associated with LINC01198 using EGFP RNA as negative control (Fig. 5C). Co-focal scanning detection of co-stained RNA fluorescence in situ hybridization for LINC01198 and Immunofluorescence (IF) staining for TAOKs in A375R cells indicated apparent colocalization of LINC01198 with TAOK1 and TAOK2 (Fig. 5D), which was also confirmed using RNA immunoprecipitation with specific antibodies (Fig. 5E). TAOK1/2 phosphorylation influences downstream phosphorylation of LATS and MST in Hippo pathway [28]. Among the pathways suggested by KEGG analysis of transcriptomic sequencing between A375P and A375R cells, the Hippo pathway was most pronounced and thus was selected for further analysis (Fig. 5F). Interestingly, Western Blot detection after nuclear-cytoplasm fraction and immunofluorescence staining showed that YAP/TAZ was significantly enriched in nuclear fraction from vemurafenib-resistant cells (Fig. 5G, H). Moreover, PI3K-AKT and MEK-ERK signaling pathway were suppressed in resistant A375R cells (Fig. 5I), which suggested PI3K-AKT and MEK-ERK signalings might not be responsible for vemurafenib resistance in melanoma.

**Fig. 6:**
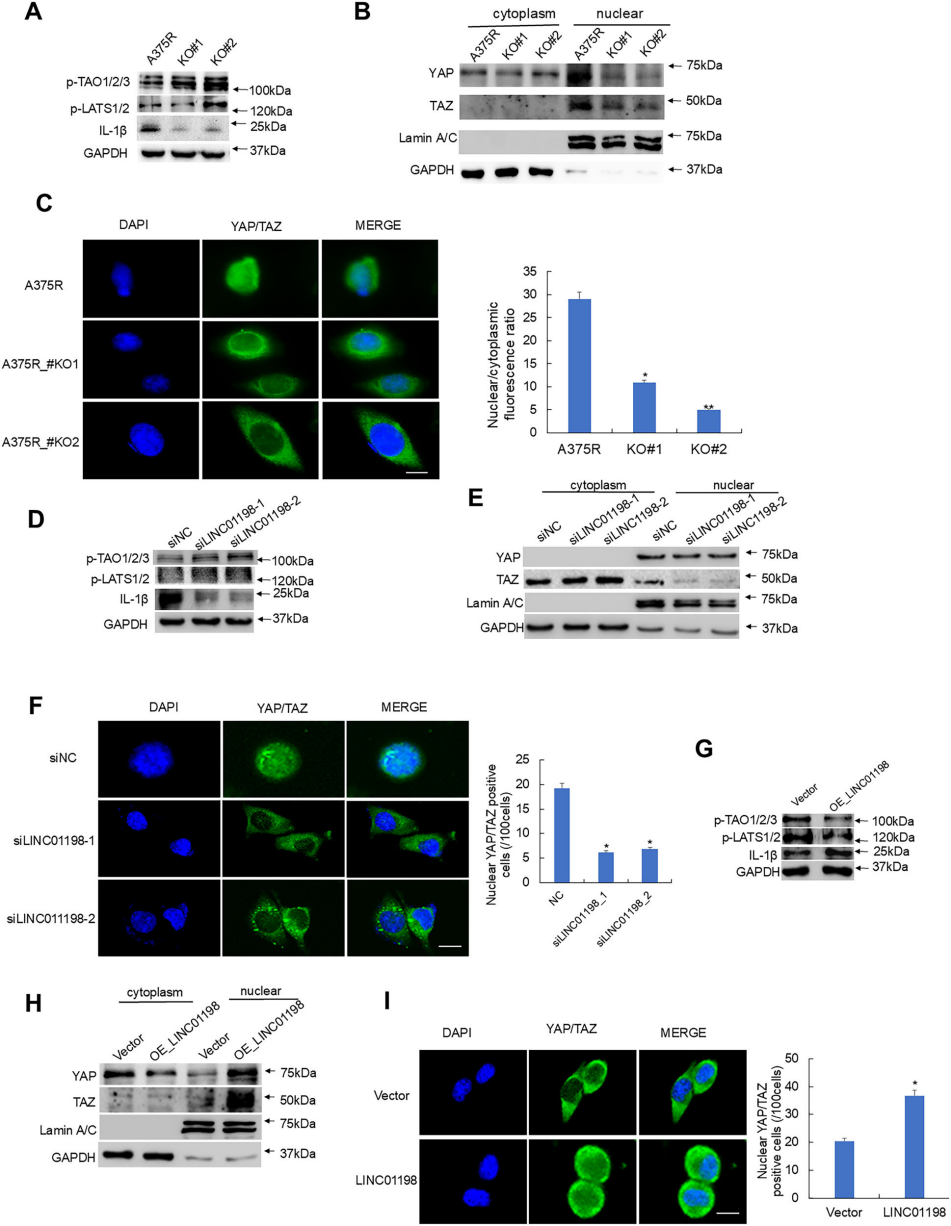
LINC01198 modulates Hippo signaling through TAOK1/2-elicited kinase cascade. p-TAOK1/2/3, p-LATS1/2, IL-1β and GAPDH were detected by Western blot in A375R cells after LINC01198 knockout **A**, knockdown **D** or overexpression **G**. YAP and TAZ levels in cytoplasm and nucleus were detected by Western blot in A375R cells after LINC01198 knockout **B**, knockdown **E** or LINC01198 overexpression **H**. Detection of YAP-GFP and TAZ-GFP cellular localization in A375R cells after LINC01198 knockout **C**, knockdown **F** and LINC01198 overexpression **I**. Scale bar: 100 μm. Nuclear/cytoplasmic fluorescence ratios in different groups were calculated. Data are presented as the means ± SD and *p* values are determined using two-tailed Student’s *t-*test. * *p* < 0.05; ** *p* < 0.01; *** *p* < 0.001.

Phosphorylated YAP/TAZ would be degraded through proteasome pathway in the cytoplasm. Only non-phosphorylated YAP/TAZ can enter the nucleus and recruit transcription factors like TEADs to promote downstream gene transcription [29]. Interestingly, LINC01198 did not directly bind to YAP and TAZ, which exclude the direct regulation of YAP/TAZ modification by LINC01198. Further, knockout of LINC01198 in A375R cells promoted the phosphorylation of TAOK1/2 and LATS1/2 (Fig. 6A). Loss of LINC01198 resulted in decreased nuclear distribution of non-phosphorylated YAP/TAZ (Fig. 6B, C). The results of LINC01198 knockdown are shown in Fig. 6D–F. In contrast, Overexpression of LINC01198 in A375R cells inhibited the phosphorylation of TAOK1/2 and LATS1/2 (Fig. 6G), while enhanced nuclear distribution of non-phosphorylated YAP/TAZ (Fig. 6H, I). Inhibited TAOK1/2 phosphorylation by LINC01198 blocks the core kinase cascade of Hippo pathway, thereby keeping YAP/TAZ in non-phosphorylated status to enter nucleus and promote the transcription of downstream genes.

### IL-1β expression regulated by LINC01198 is dependent on TAOK1/2-modulated Hippo signaling

**Fig. 7:**
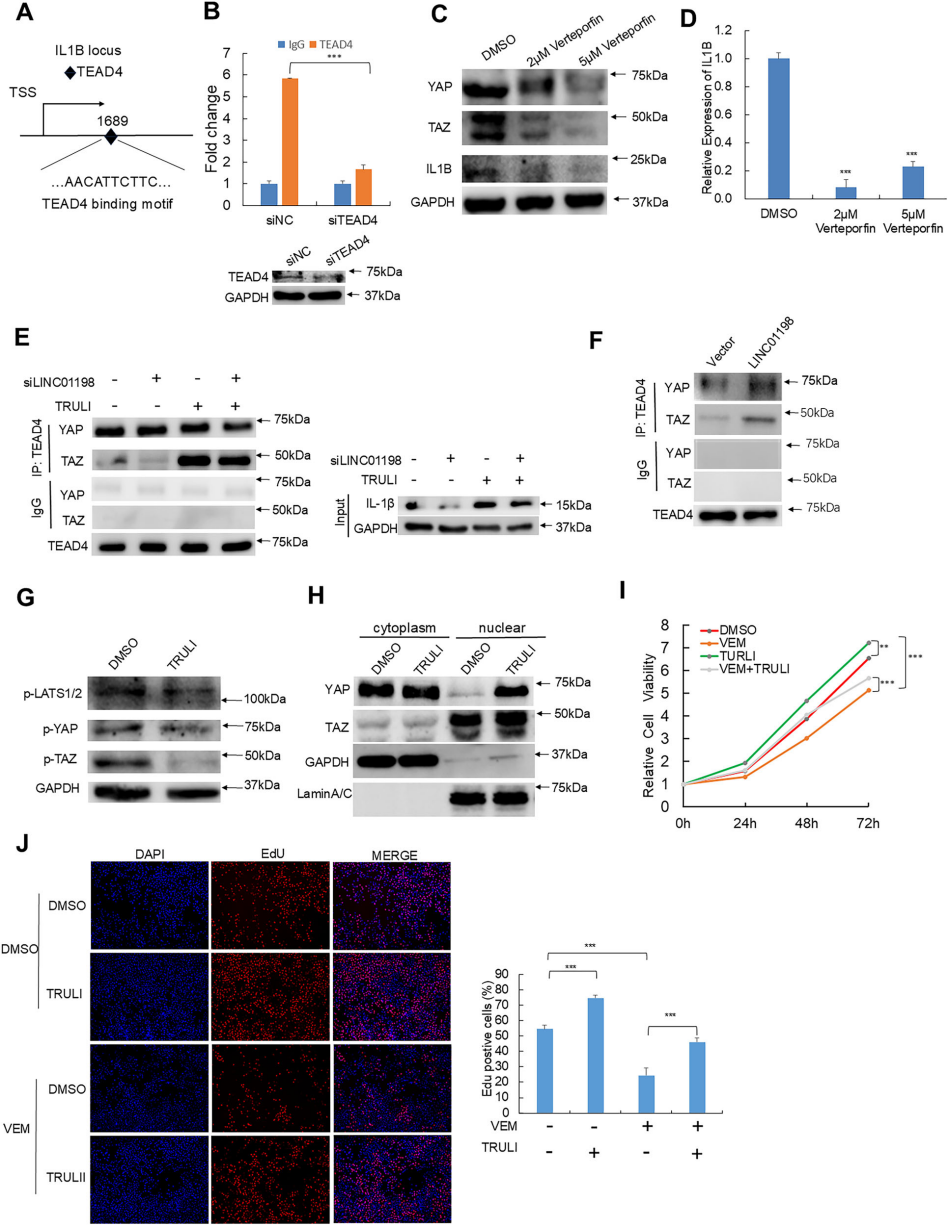
IL-1β expression regulated by LINC01198 is dependent on TAOK1/2-modulated Hippo signaling. **A** Predicted binding site of TEAD4 (diamond) at the promoter region of IL1B by NCBI (https://www.ncbi.nlm.nih.gov/) and JASPER (https://jaspar.elixir.no/). **B** The binding enrichment of TEAD4 at IL1B promoter was detected by ChIP-qPCR after knockdown of TEAD4. **C** YAP, TAZ and IL-1β were detected by Western blot in A375R cells after Verteporfin treatment. **D** RNA expression levels of IL1B in A375R cells were detected by RT-qPCR. Co-IP experiments detected the amount of YAP and TAZ proteins bound to TEAD4 in A375R cells with LINC01198 knockdown **E** or overexpression **F**. **G** p-LATS, p-YAP, p-TAZ protein levels in A375R cells treated with or without TRULI. **H** YAP and TAZ levels in cytoplasm and nucleus were detected by Western blot in A375R with or without TRULI. Cell viabilities were measured by CCK-8 assay **I** or EdU assay **J**. Data are presented as the means ± SD and *p* values are determined using two-tailed Student’s *t-*test. * *p* < 0.05; ** *p* < 0.01; *** *p* < 0.001.

In classic Hippo pathway, YAP/TAZ act as transcriptional coactivators and usually function in same complex together with transcription factor from TEAD family members to transcriptionally modulate downstream gene expression [30]. TEAD4 is the representative transcription factor from TEAD family and works in classic Hippo signaling [31]. To detect whether IL1B expression was regulated by TEAD4, the specific binding sites of TEAD4 on the promoter regions of IL1B (Fig. 7A) were predicted by NCBI (https://www.ncbi.nlm.nih.gov/) and JASPER (https://jaspar.elixir.no/). Further, we performed ChIP assay and found TEAD4 was indeed significantly enriched at the promoter region of IL1B gene locus, while silence of TEAD4 led to remarkably reduced enrichment of TEAD4 at the same locus (Fig. 7B). When the interaction of TEAD with YAP/TAZ was disrupted using Verteporfin, an inhibitor against the association between YAP and TEADs, the protein levels of YAP and TAZ together with IL1B in both RNA and protein expression decreased (Fig. 7C, D). Depletion of LINC01198 reduced the association between TEAD4 and YAP/TAZ and decreased IL-1β expression (Fig. 7E), while LINC01198 overexpression enhanced the association between TEAD4 and YAP/TAZ (Fig. 7F). In addition, TRULI, an inhibitor for core kinase LATS1/2 of Hippo signaling, was applied to verify the role of the Hippo pathway in vemurafenib resistance. TRULI treatment significantly reduced the phosphorylation of LATS1/2, YAP and TAZ, while YAP and TAZ were notably enriched in nucleus (Fig. 7G, H). Further, TRULI restored the reduced nuclear distribution of TAZ and the downregulated expression of IL-1β caused by LINC01198 deletion together with increased phosphorylation of TAOK1/2. Such results indicated that TRULI can effectively inhibit the core kinase cascade and thus activates Hippo pathway. CCK-8 and EdU assays were also conducted and demonstrated the promoting effect of TRULI on vemurafenib resistance (Fig. 7I, J). Collectively, LINC01198/TAOK/LATS activates Hippo signaling enhances nuclear translocation of YAP/TAZ to promote TEAD4-mediated IL-1β transcription.

### LINC01198 promotes melanoma resistance against vemurafenib in vivo

**Fig. 8:**
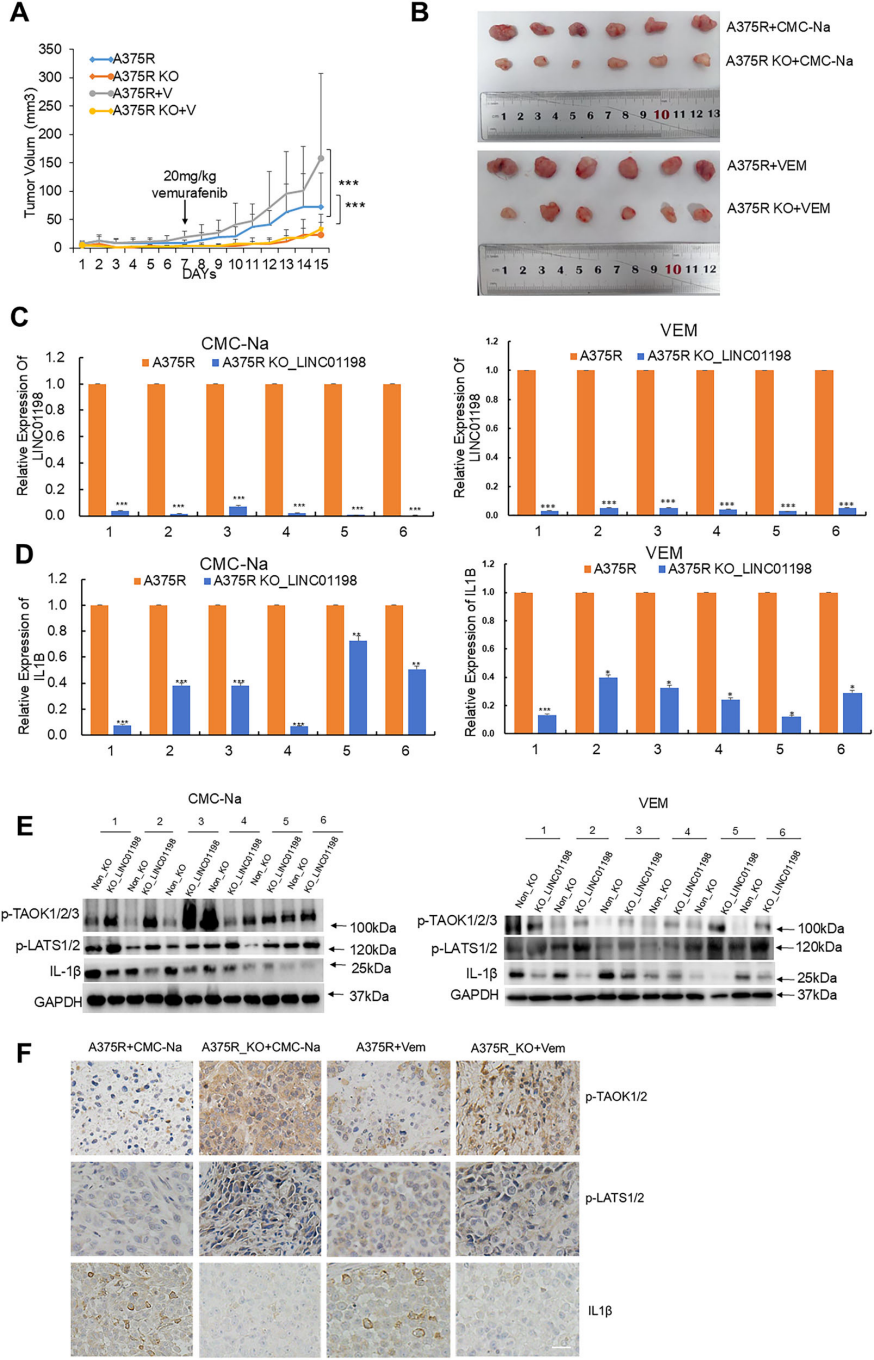
LINC01198 promotes melanoma resistance to vemurafenib in vivo. **A** Loss of LINC01198 sensitized melanoma to vemurafenib treatment in mouse xenograft model. **B** Tumor volumes (mm^3^) were plotted according to day. Mice were sacrificed at the end of the experiment and dissected tumors were shown. The expression levels of LINC01198 **C** and IL1B **D** in dissected xenografts were detected by qPCR. Statistical data of qPCR represented independent experiments ± s.d. **E** The protein levels of p-TAOK1/2/3, p-LATS1/2 and IL-1β were detected in xenografts after LINC01198 knockout with or without vemurafenib treatments by Western blot. **F** The amount of p-TAOK1/2//3, p-LATS1/2, IL-1β in tumor sections were evaluated using IHC staining. Scale bar, 50 µm. Data are presented as the means ± SD and *p* values are determined using two-tailed Student’s *t*-test. * *p* < 0.05; ** *p* < 0.01; *** *p* < 0.001.

To further validate the functional mechanism of LINC01198 for maintaining vemurafenib resistance in melanoma in vivo, A375R and LINC01198-knockout A375R cells were subcutaneously injected into nude mice. Vemurafenib was administered intragastrically into mice at 20 mg/kg twice a day in 7 days. The volumes of xenografts were calculated by measuring the size every day. Mice were sacrificed one week after vemurafenib administration and xenograft tumors were dissected for validation. Knockout of LINC01198 significantly delayed tumor growth and resulted in smaller tumor size compared with control A375R cell-derived xenografts (Fig. 8A, B). Subsequently, qPCR detection confirmed the loss of LINC01198 in KO_LINC01198 xenografts, while IL1B was also consistently and significantly down-regulated (Fig. 8C, D). Western blot and IHC detection indicated p-TAOK and p-LATS were significantly up-regulated, while IL-1β was markedly down-regulated in LINC01198 knockout group (Fig. 8E, F). In short, the above in vivo results suggest LINC01198 promotes IL1B gene expression for enhancing vemurafenib resistance in melanoma.

### LINC01198 and IL1B are higher-expressed in melanoma patients unresponsive to vemurafenib treatment

**Fig. 9:**
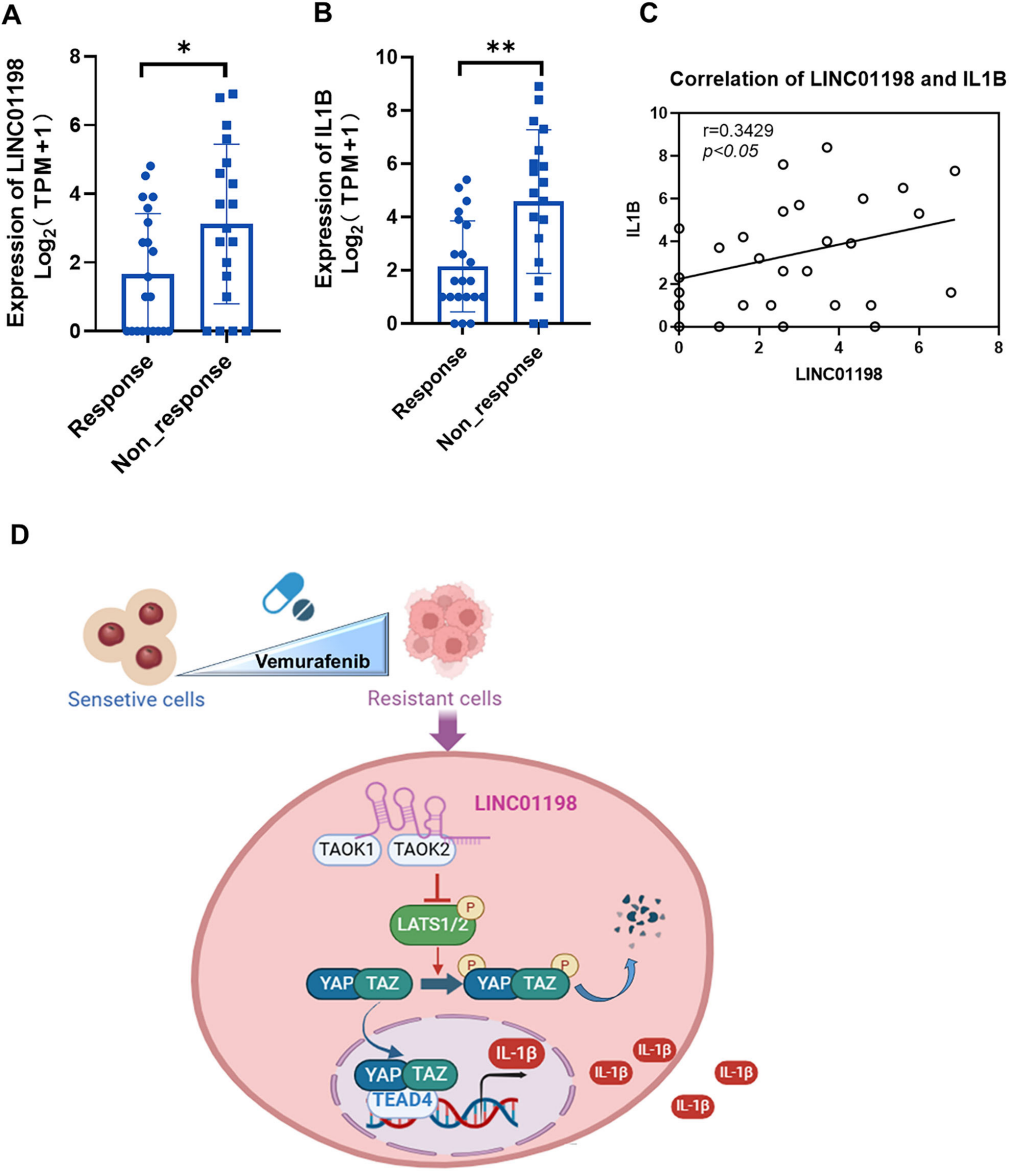
LINC01198 and IL1B are higher-expressed in melanoma patients unresponsive to vemurafenib treatment. **A** Expression levels of LINC01198 in vemurafenib responsive and non-responsive melanoma patients. **B** Expression levels of IL1B in vemurafenib responsive and non-responsive melanoma patients. **C** Correlation analysis between LINC01198 and IL1B gene expression. **D** A model depicts that LINC01198 enhances the resistance of melanoma to vemurafenib by associating with TAOK1/2 to activate Hippo signaling and transcriptionally elicit IL-1B expression. Data are presented as the means ± SD and *p* values are determined using two-tailed Student’s *t-*test. * *p* < 0.05; ** *p* < 0.01; *** *p* < 0.001.

To further validate our findings, we checked the expression of LINC01198 and IL1B using publicly available GEO datasets (GSE196434, GSE50535) and TCGA datasets (TCGA-EE-A3AD, TCGA-WE-A8ZR) from clinical treatments using vemurafenib. In melanoma patients unresponsive to vemurafenib treatments, both LINC01198 and IL1B were significantly upregulated compared to patients sensitive to vemurafenib treatments (Fig. 9A, B). In addition, LINC01198 expression was positively correlated with IL1B expression (Fig. 9C). Thus, the above results from clinical practice demonstrated higher expression of LINC01198 coordinately upregulates IL1B and contribute to melanoma resistance against vemurafenib, which is quite consistent with our in vitro and in vivo findings.

## Discussion

Targeted drug therapy using vemurafenib has revolutionized the treatment of patients with inoperable melanoma harboring BRAF gene mutation through significantly-improved overall survival and progression-free survival rates [7]. However, most of patients treated with vemurafenib developed various degrees of resistance within one year, which compromised treatment efficacy and patient quality of life [32]. To tackle vemurafenib resistance, it is essential to elucidate the underlying mechanism and identify key factors for establishing drug resistance. In this study, LINC01198 is identified highly upregulated and acts as driving factor, which associates with TAOK1/TAOK2 to activate Hippo pathway and transcriptionally induce IL-1B expression for supporting melanoma resistance against vemurafenib.

LncRNAs not only regulate tumor proliferation and migration [33] but also play important roles in tumor resistance against radiotherapy [34], chemotherapy [35], targeted therapy [36] and immunotherapy [37]. In melanoma, several LncRNAs have been identified their roles in drug resistance. LncRNA MSC-AS1 promotes glutaminolysis to enhance tumor proliferation by modulating miR-330-3p/YAP1 axis in melanoma [38]. LncRNA JPX interacts with and destabilizes YTHDF2 to facilitate melanoma progression [39]. LncRNA LENOX enhances the association of RAP2C GTPase with mitochondrial fission regulator DRP1, increasing DRP1 S637 phosphorylation, mitochondrial fusion and oxidative phosphorylation, thereby supporting melanoma resistance against MAPK inhibition [40]. In this study, LINC01198 associates with TAOK1/TAOK2 by regulating their phosphorylation to elicit Hippo signaling and bypassing vemurafenib-blocked BRAF pathway. Interestingly, IL-1β is transcriptionally induced to support vemurafenib resistance in melanoma. Moreover, the enhanced sensitivity of melanoma cells to vemurafenib following LINC01198 depletion suggests that LINC01198 could serve as a therapeutic target for combination therapy.

Since LINC01198 is a potential key target for tackling resistance against vemurafenib in melanoma, efficient targeting method is critical for clinical application. With the development of antisense oligonucleotides (ASOs) and gene-editing technologies like CRISPR/Cas9, specific knockdown or knockout of LINC01198 has become possible and worth to enter clinical trial. In our study, the regulatory function of LINC01198 in vemurafenib resistance was characterized through specific knockout (CRISPR/Cas9) in melanoma cells and RNA interference (siRNA) technologies in both cell and animal experiments, which endorses strong supports for the clinical applications of CRISPR/Cas9 and RNA interference technologies targeting LINC01198.

Although drug resistance and metastasis of tumor are two separated biological processes, they are interrelated and mutually reinforced to determine the fatal outcomes of tumors. Interestingly, LncRNA are deeply involved in regulating both of drug resistance and metastasis. For examples, in hormone-sensitive prostate cancer, HOTAIR regulates androgen receptor (AR) signaling by repressing E3-ubiquitin-mediated degradation of AR, which induces castration resistance to promote metastasis [41]. Metastasis-associated lung adenocarcinoma transcript 1 (MALAT1) significantly upregulates MRP1 and MDR1 by activating STAT3 in cisplatin (DDP) resistant non-small cell lung cancer [42]. In this study, the higher migratory capacity in vemurafenib-resistant melanoma cells compared with parental cells suggesting key signaling pathway in establishing drug resistance also contributes to enhanced metastasis. Such finding demonstrates LINC01198 plays roles in both metastasis and vemurafenib resistance in melanoma.

Hippo pathway primarily responds to diverse stimulatory factors including cell polarity, mechanical cue, cell density, soluble factors and cellular stress [15], while the effects mediated by Hippo signaling may be executed through multiple downstream effectors influencing cellular processes including apoptosis, cell cycle and inflammatory responses [12]. In this study, Hippo signaling is stimulated by the association of LIN01198 with TAOK1/2 to promote redistribution of YAP/TAZ into nucleus and bind with TEAD4 for eliciting IL-1B expression. IL-1β from IL-1 family is crucial in both inflammatory responses and tumorigenesis. Evidence suggests that tumor-derived pro-inflammatory cytokine IL-1β is essential for shaping tumor microenvironment in pancreatic cancer [25] by activating quiescent pancreatic stellate cells and driving them towards secretory phenotype to foster immunosuppressive milieu characterized by M2 macrophages and myeloid-derived suppressor cells. We sought to investigate whether tumor-derived IL-1β could also protect melanoma cells from vemurafenib destruction.

As the ultimate effector, IL-1β is critical in melanoma resistance against vemurafenib, which endorses the inhibition of IL-1β to be a key strategy for compromising drug resistance. For IL-1β inhibitors that have been approved for clinical use like Canakinumab and Rilonacept, they are originally used to treat auto-inflammatory diseases like Familial Cold Auto-inflammatory Syndrome (CAPS) and gout. Currently, the effectiveness of IL-1β inhibitors in cancer treatment is still being evaluated in clinical trial phase. Reports indicated IL-1β inhibitors can be used in combinatorial treatments to improve the therapeutic efficacy in patients harboring non-small cell lung cancer at the early stage [43]. In a post hoc analysis of the Phase III cardiovascular CANTOS trial, canakinumab, a monoclonal anti-IL-1β antibody, significantly reduced the incidence of lung cancer [44]. These reports have confirmed the potential of IL-1β inhibitors in cancer treatment. Future study may focus on the combinatorial application of IL-1β inhibitors in targeted therapy and provides new perspectives for improving cancer treatment efficacy and tackle down resistance from drugs like vemurafenib. Future study may focus on the combinatory treat of vemurafenib-resistant melanoma by targeting both of LINC01198 and IL-1β may also endorse promising for improving cancer treatment efficacy and tackle down vemurafenib resistance.

The regulatory nature of cytokines also opens up intriguing possibilities. Previous report showed LINC01198 interacts with and activates the NF-κB component p65 to trigger type I and type II interferon responses in melanoma [45]. In the future, it may be worth to silence LINC01198 in immune cells, such as tumor-associated macrophages, to assess the impact on immune defence. Should our hypothesis be confirmed—that is, if LINC01198 knockdown enhances the tumor-killing capability of the immune cells—it would provide robust evidence supporting the potential of LINC01198 as a therapeutic target for drug-resistant melanoma. Also, our findings may contribute to the development of new biomarkers for predicting drug resistance and prognosis in melanoma patients.

Collectively, LINC01198 is induced upon vemurafenib treatment and stably expressed in vemurafenib-resistant melanoma cells. Mechanistically, LINC01198 associates with TAOK1/2 to attenuate their phosphorylation and following enzymatic cascade, which potentiates nuclear transportation of YAP/TAZ to transcriptionally promote IL1B expression and enhance the viability of melanoma cells treated with vemurafenib (Fig. 9D). Our work highlights LINC01198 as critical modulator for establishing resistance against vemurafenib in melanoma harboring BRAF mutation, which also provide a series of therapeutic targets together with vemurafenib for combination therapy of melanoma, especially in the fields of personalized medicine and precision therapy.

## Materials and methods

### Cell culture and treatment

Melanoma cell A375 were obtained from Guangzhou Cellcook Biotech company. A375R (Vemurafenib-resistance) cell strains were established in our lab by stepwisely-escalated concentrations of vemurafenib (Selleck, S1267) over 6-months. All cell lines were identified by short tandem repeat (STR) analysis and tested for mycoplasma contamination. STR profiles authentication information of all the cell lines used in this study were listed in Supplementary Fig. S3. A375 and A375R cells were cultured in DMEM (Corning) medium with 10% fetal bovine serum (ExCell Bio, FSP500) and antibiotics. All cells were cultured in 37 °C humidified incubators together with 5% CO_2_.

### IC50 assay

For IC50 assay, cells were cultured at 3000 per well for A375 and A375R cells in 96-well plates with fresh medium. Six replicates were set up for each of the concentration groups. After 12 h of culture, cells were treated with indicated concentrations of vemurafenib for 72 h and analyzed by CCK-8 assay. The half inhibitory concentration (IC50) was calculated by GraphPad Prism 9.0 (GraphPad Software, Inc., CA, USA) using the logarithm of concentration vs. response.

### RNA extraction and qRT-PCR detection

Cells were harvested with TransZol Up reagent (TransGen) to isolate total RNA. All-in-One Script RT Reagent Kit (TransGen) was used to reversely transcribe RNA (1.0 μg) into cDNA. Real-time PCR analysis was conducted with PerfectStart Green qPCR SuperMix (TransGen). The expression of glyceraldehyde-3-phosphate dehydrogenase (GAPDH) was used to normalize expression data. The specific primers were listed in Supplementary Table S5. Relative gene expression levels of target gene were calculated by 2^−ΔΔCt^. Each sample was repeated three times. All the qPCR experiments were repeated 3 times.

### Cell transfection

A375, A375R cells were transfected with specific siRNAs or control siNC using EL transfection reagent (TransGene) according to manufacturer’s instructions to silence target gene expression. LINC01198 siRNAs were purchased from RiboBio along with siNC and their sequences were listed in Supplementary Table S5. PEI transfection reagent (Servicebio) is used for plasmid transfection.

### Construction of LINC01198 knockout cell strains

For LINC01198 knockout, single guide RNAs (sgRNA) were designed using online CRISPR design tool (CRISPOR, http://crispor.tefor.net/). A ranked list of sgRNAs was generated with specificity and efficiency scores. Four pairs sgRNAs flanking the genomic locus of LINC01198 were selected and assessed using off-target searching tool (Cas-OFFinder; http://www.rgenome.net/cas-offinder). The pair of oligos was annealed and ligated to Bbs (I-digested pSpCas9BB)-2A-Puro (PX459) V2.0 (Addgene plasmid #62988) respectively. Such two pX459 plasmids expressing each sgRNAs were cotransfected into cells with Lipofectamine 2000 (Thermo Fisher Scientific). After isolation of clonal cell strains by dilution, 100 cells were seeded into each well of 96-well plate. After clone screening, colonies with genomic knockout of LINC01198 were determined by Sanger sequencing and LINC01198 expression levels in each clone were validated by qPCR. The knockout design and sequences of sgRNAs and primers for CRISPR knockout and genomic validation are shown in Supplementary Fig. S2 and Supplementary Table S5.

### Cell viability and colony forming assays

The viability of cells was measured with CCK-8 (Sigma) following manufacturer’s protocol. Equal number of cells (5000 per well) transfected with each siRNA were plated in 96-well plates with 4-well technical replicates. After 0, 24, 48, and 72 h, the cells were incubated with 10 μL CCK-8 solution in cell counting kit at 37 °C for 1.5 h. Subsequently, optical density (OD) value was measured at 450 nm using microplate reader. The colony formation assay was performed by seeding transfected cells into six well plates (1000 cells per well) and culturing for two weeks. Colonies were fixed with methanol and then stained with 0.1% crystal violet in PBS for 10 min. Colony formation was quantified by counting stained colonies. EdU incorporation assay was conducted using a Cell-Light EdU Apollo®567 In Vitro Imaging Kit (RiboBio). The kit for labeling/detecting proliferating cells was used in accordance with the manufacturer’s instructions. Confocal laser scanning microscopy was used to determine the percentage of EdU-positive cells. The experiment was performed with three replicates.

### Immunofluorescence staining and Immunofluorescence-FISH

Cells were fixed and permeabilized with 0.5% NP-40 for 10 min, non-specific binding was blocked with 5% BSA in PBS for 1 h. Cells were then incubated with the primary antibody overnight at 4^o^C, followed by incubation in dark with the secondary antibody for 1 h. Nuclei were stained with DAPI. Antibodies against YAP/TAZ were used to observe subcellular distributions. ImageJ was used to analyze the fluorescence intensity of YAP/TAZ (Green) in nucleus and cytoplasm. Cells with a nuclear/cytoplasmic ratio greater than 1.5 were considered as positive cells using ImageJ for analysis. At least 100 cells were counted. Antibodies were used with 1:250 dilution. Co-localization of LINC01198 with TAOK1/TAOK2 in melanoma cells was detected using FISH for LINC01198 (Green) and immunofluorescence staining for TAOK1/TAOK2 (Red) and observed by confocal microscope. All Immunofluorescence and FISH staining were performed with at least three biological replicates.

### Chromatin immunoprecipitation (ChIP) assay

Chromatin immunoprecipitation (ChIP) procedure was performed following the guidance as before [46]. 5 μg antibodies against TEAD4 (Santa Cruz) or isotype IgG (Merck Millipore) as negative control were added and co-precipitates were captured by Protein G magnetic beads. Genomic DNA pellets were purified using phenol chloroform extraction and ethanol precipitation and then resuspended in 20 μL water for qPCR analysis. QPCR Primers are listed in Supplementary Table S5. The experiment was repeated with three replicates.

### In vitro transcription and RNA pull down assay

Biotin-labeled RNAs were transcribed in vitro using RNA max-T7 biotin-labeled transcription kit (Ribo Biotechnology Co., Ltd.). The above RNAs were denatured at 90 °C for 2 min and then renatured with RNA structure buffer at RT for 20 min. A375R cell pellets (5 × 10^6^) were resuspended in 1 mL RIP buffer (150 mM KCl, 25 mM Tris pH 7.4, 0.5 mM DTT, 0.5% NP40, 1 mM phenylmethyl sulfonyl fluoride and 1 × PIC) and sonicated with 10 cycles (30 s interval, 30 s sonication). After centrifugation at 13,000 rpm for 10 min, total cell lysate was mixed with 3 μg of renatured RNA respectively and incubated with rotation for 1 h at RT. Each pull-down reaction was mixed with 30 μL of washed streptavidin agarose beads (Life Technologies) at RT for 1 h. After washing thoroughly with three times, the RNA–protein binding mixture was boiled in SDS buffer and the eluted proteins were detected by western blot or mass spectrometry.

### Animal xenograft tumor model

This study was approved by the Institutional Animal Care and Use Committee (IACUC) of Nanfang Hospital affiliated with Southern Medical University (L2019178). To establish xenograft models, four-week-old male mice were subcutaneously injected with 0.2 mL cell suspension, containing 4×10^6^ either A375R or A375R with LINC01198-knockout cells. Xenograft tumors developed 7 days later and xenografted mice were randomly divided into four groups (8 mice per group): two groups with and without LINC01198 knockout, while two vemurafenib-treated groups with and without LINC01198 knockout. Vemurafenib treatment was conducted twice a day at the dose of 25 mg/kg [47]. Mice were weighed, and tumor volume were measured every two days. Tumor volume was calculated following the formula (0.52 × length × width^2^). After 4 weeks, all mice were euthanized and xenografts were dissected and measured.

### Immunoblotting and IHC assays

Xenograft tumors were formalin-fixed and paraffin-embedded and sectioned for IHC staining. The following antibodies were used: p-TAOK1/2/3 (Abcam, 1:100), p-LATS1/2 (Bioss, 1:100) and IL-1β (Santa Cruz, 1:100). Stained sections were imaged using BX53 microscope (Olympus) to get representative images for statistical analysis. All immunoblotting and IHC were performed in three or more biological replicates.

### Statistical analysis

SPSS 23.0 (IBM SPSS Inc. Chicago, IL, USA) statistical software was used. All statistical analyses were performed using either independent-sample *t*-tests (unpaired, two-tailed) or one-way *ANOVA* with homogeneity of variance tests. Sample numbers (n) are indicated in the respective figure legends.

### Statement

According to the standards of the National Institutes of Health (NIH), the tumor diameter was no more than 20 mm. This study was approved by the Institutional Animal Care and Use Committee (IACUC) of Nanfang Hospital affiliated with Southern Medical University (L2019178). We confirm that all methods were performed in accordance with the relevant guidelines and regulations.

## Supplementary information

Supplementary Materials

Original Data

## Data Availability

Mass spectrometry data has been deposited to ProteomeXchange (http://www.proteomexchange.org) with identifier PXD056757. RNA-seq data have been submitted in Gene Expression Omnibus (https://www.ncbi.nlm.nih.gov/geo/) with accession ID GSE289512. All data generated or analyzed during this study are included in this article and Supplementary files and available from the corresponding authors on request.
