# Supplementary material for: LINC01198 activates Hippo signaling to stimulate IL-1β autocrine for driving vemurafenib resistance by associating with TAOK1/2 in melanoma
Source: Cell Death Discov. 2025 Oct 27;11:486. doi: 10.1038/s41420-025-02773-6 (PMC12559216; doi:10.1038/s41420-025-02773-6)
Supplement: Supplementary file 1 — Supplementary Materials [file 41420_2025_2773_MOESM1_ESM.pdf]

Supplementary Figure S1

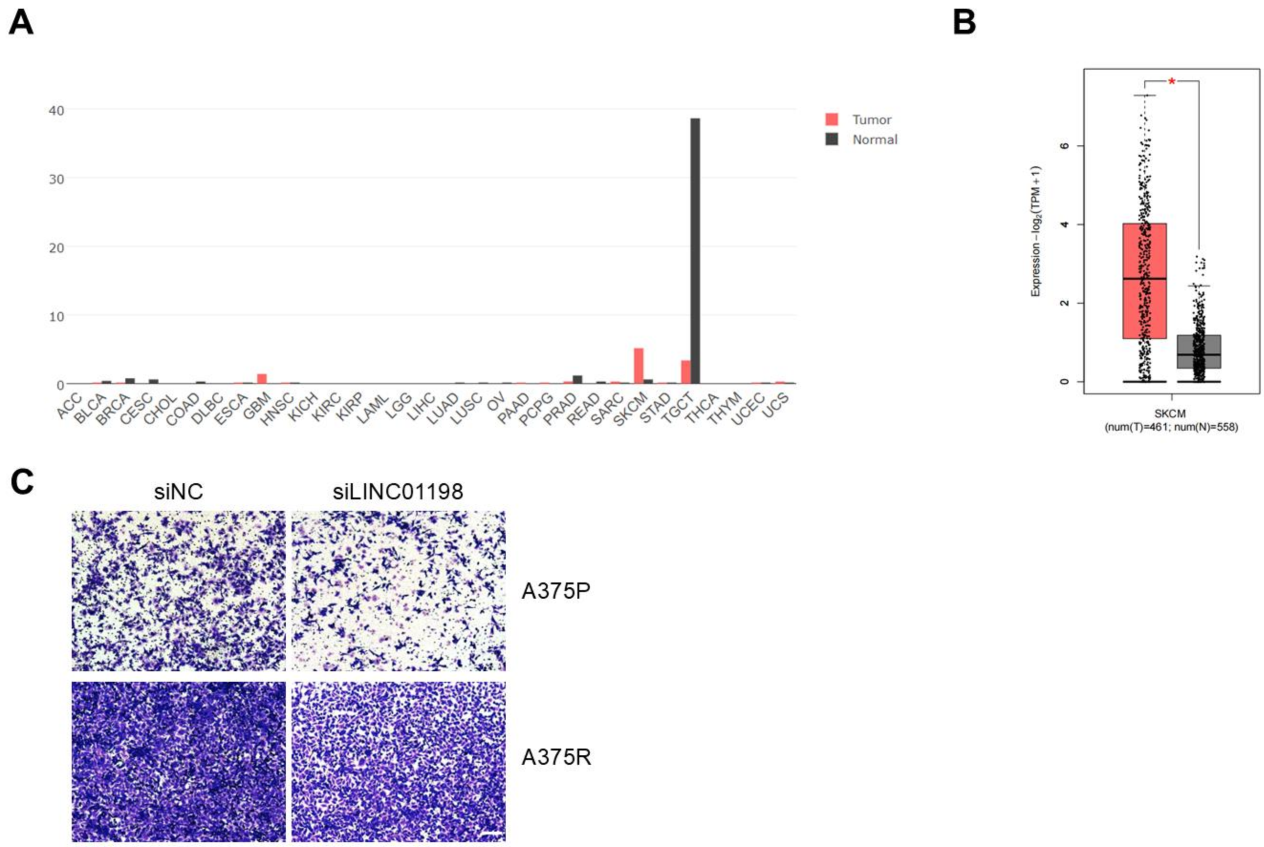

Supplementary Figure S1. Normalized LINC01198 expression levels in different types of cancer. (A) The diagram showing the expression levels of LINC01198 in different types of cancer were analyzed and generated in Gene Expression Profiling Interactive Analysis (http://gepia.cancer-pku.cn/) using TCGA as data source. (B) The diagram showing the expression levels of LINC01198 in melanoma and normal skin. (C) Transwell migration assay were performed in A375P and A375R cells treated with siRNAs targeting LINC01198. GEO Accession viewer (nih.gov)

Supplementary Figure S2

A

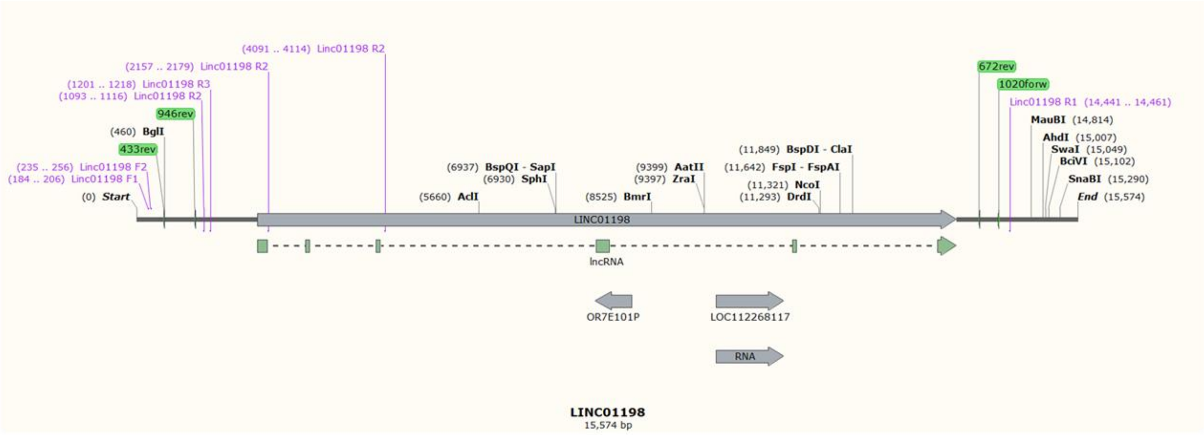

B

gRNA Oligo	Purification and synthesis by PAGE	
	gRNA946F	CACCGtcccggagcctaagtgccta
	gRNA946R	AAACtaggcacttaggctccgggaC
	gRNA433F	CACCgcaccacccgtgagagtgct
	gRNA433R	AAACagcactctcacgggtggtgc
	gRNA1020F	CACCggggagtgggttaatctaat
	gRNA1020R	AAACattagattaacccactcccc
	gRNA672F	CACCgcattatgtgattgctcagg
	gRNA672R	AAACcctgagcaatcacataatgc
PCR Identification primers		
	LINC01198 F1	GCACAAGTTTGATGGCACAAGTC
	LINC01198 F2	ACCTCAAACACTGCAAAGGTTC
	LINC01198 R1	TATGTGCATTTCGGCTGCTCA
	LINC01198 R2	CTTAGGACCCACTTGGAGGACTGT
	LINC01198 R3	GACACCAATCGCAGGGGT

C

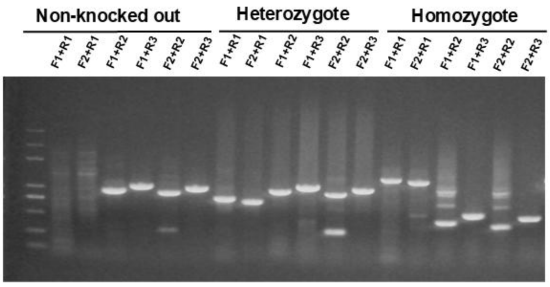

Supplementary Figure S2. Construction of LINC01198 knockout strain from A375R cells. (A) The gRNA sites used for targeting using CRISPR-Cas9 method. (B) Primers were designed to located upstream or downstream for identification of LINC01198 knockout. (C) PCR was conducted to detect the deletion of LINC01198 with the primers (B) using Genomic DNA to determine the knockout efficiency.

Supplementary Figure S3

A375 cell line authentication

Figure 1. STR profiles of A-375 cell line

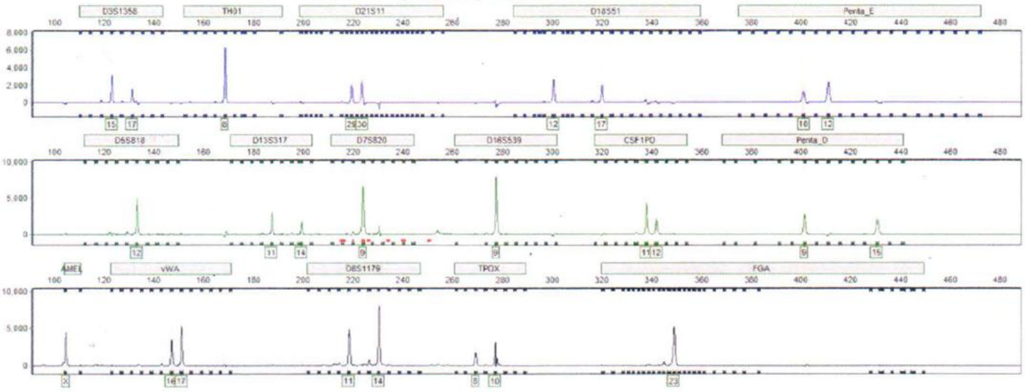

Table 1. STR profiles of A-375 cell line

	Allele1	Allele2
D3S1358	15	17
TH01	8	
D21S11	29	30
D18S51	12	17
Penta E	10	12
D5S818	12	
D13S317	11	14
D7S820	9	
D16S539	9	
CSF1PO	11	12
Penta D	9	15
AMEL	x	
vWA	16	17
D8S1179	11	14
TPOX	8	10
FGA	23	

Figure 2. Search result in ATCC database

SEARCH THE STR DATABASE

As part of our continuing efforts to characterize and authenticate the cell lines in the Cell Biology collection, ATCC has developed a comprehensive database of short tandem repeat (STR) DNA profiles for all of our human cell lines. View our brief tutorial before starting.

1. STR Profiling Analysis
2. Matching Algorithm
3. Interrogating the Database

Showing 1-- 5 Of 5

PageSize: 100 ▼

Add to Cart	%Match	ATCC® Number	Designation	D5S818	D13S317	D7S820	D16S539	vWA	TH01	AMEL	TPOX	CSF1PO
☐	100.0	CRL-1872	A375.S2MelanomaHuman	12	11,14	9	9	16,17	8	X	8,10	11,12
☐	100.0	CRL-1619	A375MelanomaHuman	12	11,14	9	9	16,17	8	X	8,10	11,12
☐	100.0	CRL-3222	A375-MA1MelanomaHuman	12	11,14	9	9	16,17	8	X	8,10	11,12
☐	100.0	CRL-3223	A375-MA2MelanomaHuman	12	11,14	9	9	16,17	8	X	8,10	11,12
☐	100.0	CRL-3224	A375PMelanomaHuman	12	11,14	9	9	16,17	8	X	8,10	11,12

Add to Cart   Export to Excel

Supplementary Table S1. The list of differentially expressed LncRNAs from the above two transcriptomic

gene	gene_type	Position	Strand	A375P	A375+DMSO	A375R	A375+VEM	log2(Fold_change)	p-value	Description	Website
LINC00290	lncRNA	chr4:181985242-182080302	-	1.39E-17	1.39E-17	1.1053179	36.065439	7.2274603	0.0026	long intergenic non-protein coding RNA 290	http://www.ncbi.nlm.nih.gov/gene/?term=728081
ENSG00000273124.1	lincRNA	chr10:92757237-92757470	-	0	1.0115835	0.0435257	88.178363	6.1402741	0.0039	None	http://asia.ensembl.org/Homo_sapiens/Gene/Idhistory?g=ENSG00000273124
LOC100507487	lncRNA	chr4:129349171-129440553	+	2.9469704	8.1003484	15.923974	108.0491	3.460408	0.004	uncharacterized LOC100507487	http://www.ncbi.nlm.nih.gov/gene/?term=100507487
LOC105378047	lncRNA	chr6:149348365-149353797	-	3.93943	3.0420801	7.4604067	79.637179	3.5948669	0.0057	uncharacterized LOC105378047	http://www.ncbi.nlm.nih.gov/gene/?term=105378047
ENSG00000235565.1	lincRNA	chr1:95086707-95089740	-	1.39E-17	1.39E-17	3.2139977	15.194694	6.2257233	0.0059	None	http://asia.ensembl.org/Homo_sapiens/Gene/Idhistory?g=ENSG00000235565
ENSG00000258390.1	lincRNA	chr14:96039899-96048293	+	0.9865334	0.0014139	2.165044	32.269985	4.8016056	0.007	None	http://asia.ensembl.org/Homo_sapiens/Gene/Idhistory?g=ENSG00000258390
LINC00324	lncRNA	chr17:8123948-8127361	-	5.9003044	15.191839	45.524248	88.118522	2.650644	0.0072	long intergenic non-protein coding RNA 324	http://www.ncbi.nlm.nih.gov/gene/?term=284029

gene	gene_type	Position	Strand	A375P	A375+DMSO	A375R	A375+VEM	log2(Fold_change)	p-value	Description	Website
ENSG00000234535.1	lincRNA	chr13:34185104-34185659	+	7.87677	8.1065923	19.09629	105.20265	2.9404567	0.0078	None	http://asia.ensembl.org/Homo_sapiens/Gene/Idhistory?g=ENSG00000234535
ABHD11-AS1	lncRNA	chr7:73149399-73150330	+	3.9349442	6.077639	16.972658	59.719659	2.9080784	0.0084	ABHD11 antisense RNA 1 (tail to tail)	http://www.ncbi.nlm.nih.gov/gene/?term=171022
STXBP5-AS1	lncRNA	chr6:147162524-147525750	-	3.9442481	1.0185486	16.95066	27.48418	3.097642	0.0086	STXBP5 antisense RNA 1	http://www.ncbi.nlm.nih.gov/gene/?term=729178
ENSG00000258998.1	lincRNA	chr14:45232360-45252032	-	1.39E-17	1.39E-17	1.1021799	19.950484	6.4155456	0.0088	None	http://asia.ensembl.org/Homo_sapiens/Gene/Idhistory?g=ENSG00000258998
LOC101927549	lncRNA	chr10:107433368-107580091	-	1.39E-17	1.39E-17	15.816441	1.855273	6.1625509	0.0089	uncharacterized LOC101927549	http://www.ncbi.nlm.nih.gov/gene/?term=101927549
LINC00518	lncRNA	chr6:10428018-10435055	-	4.9257971	3.043271	7.4601322	74.899611	3.3295761	0.0094	long intergenic non-protein coding RNA 518	http://www.ncbi.nlm.nih.gov/gene/?term=221718
ENSG00000255745.1	lincRNA	chr12:24857499-24927147	+	1.39E-17	1.39E-17	8.4509219	4.7173422	5.7447613	0.0098	None	http://asia.ensembl.org/Homo_sapiens/Gene/Idhistory?g=ENSG00000255745
HCG23	lncRNA	chr6:32358287-32361468	+	0	2.0223361	10.592691	13.264707	3.4180247	0.0126	HLA complex group 23 (non-protein coding)	http://www.ncbi.nlm.nih.gov/gene/?term=414764

gene	gene_type	Position	Strand	A375P	A375+DMSO	A375R	A375+VEM	log2(Fold_change)	p-value	Description	Website
ENSG00000184274.3	lincRNA	chr21:46720160-46725172	-	2.9576054	1.0175099	8.5053486	29.402795	3.174379	0.0127	None	http://asia.ensembl.org/Homo_sapiens/Gene/Idhistory?g=ENSG00000184274
LOC104968399	lncRNA	chr18:3255434-3262357	-	14.772427	12.161215	2.1096441	0.9342255	-3.054475	0.0139	uncharacterized LOC104968399	http://www.ncbi.nlm.nih.gov/gene/?term=104968399
ENSG00000256955.1	lincRNA	chr12:132341965-132348596	+	2.9610412	0.0033471	5.3379547	32.257896	3.5533416	0.0147	None	http://asia.ensembl.org/Homo_sapiens/Gene/Idhistory?g=ENSG00000256955
ENSG00000272512.1	lincRNA	chr1:931346-933431	-	40.398117	0.0091876	1.0501289	0	-4.999162	0.0157	None	http://asia.ensembl.org/Homo_sapiens/Gene/Idhistory?g=ENSG00000272512
ENSG00000257877.1	lincRNA	chr12:112250597-112251224	+	1.39E-17	1.39E-17	1.099875	15.207829	6.0526869	0.0157	None	http://asia.ensembl.org/Homo_sapiens/Gene/Idhistory?g=ENSG00000257877
LINC00431	lncRNA	chr13:111618374-111642949	+	38.42398	4.0644088	2.1191094	1.8883159	-3.328777	0.0167	long intergenic non-protein coding RNA 431	http://www.ncbi.nlm.nih.gov/gene/?term=104355135
ENSG00000261467.2	lincRNA	chr7:73400322-73403097	+	10.843124	2.0349031	1.39E-17	1.39E-17	-5.71666	0.0169	None	http://asia.ensembl.org/Homo_sapiens/Gene/Idhistory?g=ENSG00000261467

gene	gene_type	Position	Strand	A375P	A375+DMSO	A375R	A375+VEM	log2(Fold_change)	p-value	Description	Website
ENSG00000249776.1	lincRNA	chr5:91745963-91996570	-	1.39E-17	1.39E-17	0.0355039	20.905157	6.4069863	0.0177	None	http://asia.ensembl.org/Homo_sapiens/Gene/Idhistory?g=ENSG00000249776
ENSG00000254416.1	lincRNA	chr11:110225855-110277124	+	3.9299139	13.164128	6.4027206	150.69374	3.1819596	0.0178	None	http://asia.ensembl.org/Homo_sapiens/Gene/Idhistory?g=ENSG00000254416
RNF144A-AS1	lncRNA	chr2:7052407-7058813	-	36.428278	79.023716	136.52187	361.89425	2.1080582	0.0181	RNF144A antisense RNA 1	http://www.ncbi.nlm.nih.gov/gene/?term=386597
FUT8-AS1	lncRNA	chr14:65877310-65879335	-	7.8684879	23.295824	95.19882	53.96958	2.2502223	0.0194	FUT8 antisense RNA 1	http://www.ncbi.nlm.nih.gov/gene/?term=645431
STARD13-AS	lncRNA	chr13:33851691-33855471	+	0	5.0571304	12.729083	29.39095	3.0040773	0.0198	STARD13 antisense RNA	http://www.ncbi.nlm.nih.gov/gene/?term=100874241
ENSG00000259828.1	lincRNA	chr6:141768148-141772143	+	0.9733455	18.223381	71.933218	51.135551	2.6659796	0.0201	None	http://asia.ensembl.org/Homo_sapiens/Gene/Idhistory?g=ENSG00000259828
LINC00482	lncRNA	chr17:79276624-79283048	-	2.9512229	4.0522767	10.621483	35.086257	2.6657819	0.0203	long intergenic non-protein coding RNA 482	http://www.ncbi.nlm.nih.gov/gene/?term=284185
LINC02029	lncRNA	chr3:156799630-156806336	+	1.39E-17	1.39E-17	10.538965	1.8605818	5.6585264	0.0216	long intergenic non-protein coding RNA 2029	http://www.ncbi.nlm.nih.gov/gene/?term=105374177

gene	gene_type	Position	Strand	A375P	A375+DMSO	A375R	A375+VEM	log2(Fold_change)	p-value	Description	Website
LOC100507144	lncRNA	chr11:35154197-35159579	-	1.9617622	7.0862849	21.179714	31.270154	2.5053585	0.0216	uncharacterized LOC100507144	http://www.ncbi.nlm.nih.gov/gene/?term=100507144
SOX2-OT	lncRNA	chr3:180774468-181460016	+	5.9025291	11.141615	2.1664348	174.38147	3.3538477	0.0221	SOX2 overlapping transcript	http://www.ncbi.nlm.nih.gov/gene/?term=347689
LOC729870	lncRNA	chr4:153855668-153857989	-	0.9784825	4.0481143	4.2833593	42.688312	3.1646254	0.0222	uncharacterized LOC729870	http://www.ncbi.nlm.nih.gov/gene/?term=729870
HCG26	lncRNA	chr6:31439006-31440185	+	0	4.0452208	5.3395782	36.997893	3.3164367	0.0224	HLA complex group 26 (non-protein coding)	http://www.ncbi.nlm.nih.gov/gene/?term=352961
ENSG00000251396.2	lincRNA	chr8:61297147-61429354	-	3.9309104	11.139047	8.5192167	101.42713	2.8476134	0.0225	None	http://asia.ensembl.org/Homo_sapiens/Gene/Idhistory?g=ENSG00000251396
NFIA-AS2	lncRNA	chr1:61405916-61436448	-	1.39E-17	1.39E-17	1.096794	11.411505	5.6776576	0.0233	NFIA antisense RNA 2	http://www.ncbi.nlm.nih.gov/gene/?term=100996570
FLJ16779	lncRNA	chr20:61885330-61892967	+	0.9784825	4.0481143	1.1064677	61.649089	3.578981	0.0243	uncharacterized LOC100192386	http://www.ncbi.nlm.nih.gov/gene/?term=100192386
ENSG00000224239.1	lincRNA	chr3:898807-899774	+	0	1.0115835	5.3115223	9.4849344	3.5898798	0.0244	None	http://asia.ensembl.org/Homo_sapiens/Gene/Idhistory?g=ENSG00000224239

gene	gene_type	Position	Strand	A375P	A375+DMSO	A375R	A375+VEM	log2(Fold_change)	p-value	Description	Website
ENSG00000258711.2	lincRNA	chr14:51422977-51428720	-	6.8810582	35.450162	24.390783	239.72914	2.6347478	0.0264	None	http://asia.ensembl.org/Homo_sapiens/Gene/Idhistory?g=ENSG00000258711
ENSG00000224698.1	lincRNA	chr1:108963311-108975806	+	66.973637	29.39106	13.737685	7.556596	-2.167371	0.0267	None	http://asia.ensembl.org/Homo_sapiens/Gene/Idhistory?g=ENSG00000224698
PACRG-AS1	lncRNA	chr6:163731017-163745505	-	0	5.0571304	9.5620638	30.347945	2.9266034	0.027	PACRG antisense RNA 1	http://www.ncbi.nlm.nih.gov/gene/?term=285796
LOC101929237	lncRNA	chr8:22735485-22745535	+	21.679672	3.0500413	2.0968244	0	-3.444125	0.0272	uncharacterized LOC101929237	http://www.ncbi.nlm.nih.gov/gene/?term=101929237
LINC01268	lncRNA	chr6:114189178-114194512	-	0	2.0223361	5.323911	15.183876	3.203192	0.0273	long intergenic non-protein coding RNA 1268	http://www.ncbi.nlm.nih.gov/gene/?term=285758
ADD3-AS1	lncRNA	chr10:111705317-111768139	-	0	2.0223361	1.1046476	30.378884	3.8117409	0.0293	ADD3 antisense RNA 1	http://www.ncbi.nlm.nih.gov/gene/?term=100505933
FOXC2-AS1	lncRNA	chr16:86598751-86601367	-	17.737386	4.0621383	2.0968244	0	-3.266421	0.0295	FOXC2 antisense RNA 1	http://www.ncbi.nlm.nih.gov/gene/?term=103752587
ENSG00000234902.2	lincRNA	chr2:208051441-208100790	-	5.9119997	3.0442618	1.39E-17	1.39E-17	-5.204745	0.03	None	http://asia.ensembl.org/Homo_sapiens/Gene/Idhistory?g=ENSG00000234902

gene	gene_type	Position	Strand	A375P	A375+DMSO	A375R	A375+VEM	log2(Fold_change)	p-value	Description	Website
RBFADN	lncRNA	chr18:77827256-77839206	+	5.9119997	3.0442618	1.39E-17	1.39E-17	-5.204745	0.03	RBFA downstream neighbor (non-protein coding)	http://www.ncbi.nlm.nih.gov/gene/?term=100506070
HCP5B	lncRNA	chr6:29839672-29841560	-	0.9865334	0.0014139	2.15798	15.200932	3.8248938	0.0312	HLA complex P5B (non-protein coding)	http://www.ncbi.nlm.nih.gov/gene/?term=352990
ENSG00000236983.1	lincRNA	chr10:27232252-27233347	-	0.9865334	0.0014139	2.15798	15.200932	3.8248931	0.0312	None	http://asia.ensembl.org/Homo_sapiens/Gene/Idhistory?g=ENSG00000236983
LOC105375734	lncRNA	chr8:123426566-123580883	-	1.39E-17	1.39E-17	15.801378	0	6.0027166	0.0313	None	http://www.ncbi.nlm.nih.gov/gene/?term=LOC105375734
ENSG00000231081.1	lincRNA	chr20:25989427-25991167	-	1.9647311	4.0504131	5.3395782	36.997893	2.7675714	0.0324	None	http://asia.ensembl.org/Homo_sapiens/Gene/Idhistory?g=ENSG00000231081
TTC39C-AS1	lncRNA	chr18:21574177-21595424	-	7.8757778	9.1189077	1.0618631	0.9417386	-2.937537	0.0328	TTC39C antisense RNA 1	http://www.ncbi.nlm.nih.gov/gene/?term=102724246
LINC01198	lincRNA	chr13:47029270-47060641	-	68.925581	160.07951	204.23709	627.13688	1.8592364	0.033	None	http://asia.ensembl.org/Homo_sapiens/Gene/Idhistory?g=ENSG00000231817

gene	gene_type	Position	Strand	A375P	A375+DMSO	A375R	A375+VEM	log2(Fold_change)	p-value	Description	Website
LOC101927855	lncRNA	chr17:59439029-59445065	-	1.39E-17	1.39E-17	1.0945149	9.5121739	5.4454532	0.0331	uncharacterized LOC101927855	http://www.ncbi.nlm.nih.gov/gene/?term=101927855
LINC01251	lncRNA	chr9:33732973-33738414	-	1.39E-17	1.39E-17	1.0945149	9.5121739	5.4454516	0.0331	long intergenic non-protein coding RNA 1251	http://www.ncbi.nlm.nih.gov/gene/?term=101929688
ENSG00000223704.1	lincRNA	chr22:27254597-27261749	+	3.9376603	4.0538126	4.2843094	50.26991	2.7344818	0.0341	None	http://asia.ensembl.org/Homo_sapiens/Gene/Idhistory?g=ENSG00000223704
CASC15	lncRNA	chr6:21666675-22194629	+	2.9447217	12.150078	11.690529	74.891839	2.5020186	0.0351	cancer susceptibility 15 (non-protein coding)	http://www.ncbi.nlm.nih.gov/gene/?term=401237
LOC101927830	lncRNA	chrX:154576450-154603823	-	1.39E-17	1.39E-17	2.143672	6.6519582	5.1833864	0.0352	uncharacterized LOC101927830	http://www.ncbi.nlm.nih.gov/gene/?term=101927830
DLEU7-AS1	lncRNA	chr13:51381991-51424041	+	0	4.0452208	0.0426482	68.283747	3.999944	0.0356	DLEU7 antisense RNA 1	http://www.ncbi.nlm.nih.gov/gene/?term=100874074
BCYRN1	lncRNA	chr2:47562454-47562653	+	28.557669	28.371023	4.2554039	8.5399636	-2.128027	0.0358	brain cytoplasmic RNA 1	http://www.ncbi.nlm.nih.gov/gene/?term=618
ENSG00000269843.1	lincRNA	chr19:41337126-41343115	-	1.9577189	19.237785	6.4026338	160.16713	2.9598223	0.0359	None	http://asia.ensembl.org/Homo_sapiens/Gene/Idhistory?g=ENSG00000269843

gene	gene_type	Position	Strand	A375P	A375+DMSO	A375R	A375+VEM	log2(Fold_change)	p-value	Description	Website
BOK-AS1	lncRNA	chr2:242483799-242498558	-	7.8715532	15.193884	0.0142562	2.863611	-2.871979	0.0362	BOK antisense RNA 1	http://www.ncbi.nlm.nih.gov/gene/?term=100379249
LINC00202-1	lncRNA	chr10:27220135-27230930	-	0.9752757	9.1088353	5.3438603	71.113827	2.8933031	0.037	None	http://www.ncbi.nlm.nih.gov/gene/?term=LINC00202-1
ENSG00000259380.1	lincRNA	chr15:38431796-38519088	-	0	1.0115835	1.1009457	17.105174	3.8826759	0.0379	None	http://asia.ensembl.org/Homo_sapiens/Gene/Idhistory?g=ENSG00000259380
ENSG00000225643.1	lincRNA	chr1:25907969-25916847	+	1.9740602	0.0024941	2.161703	20.892888	3.3833344	0.0387	None	http://asia.ensembl.org/Homo_sapiens/Gene/Idhistory?g=ENSG00000225643
ENSG00000237595.2	lincRNA	chr20:48780001-48782639	-	0	4.0452208	4.2799636	29.417507	2.9894356	0.0396	None	http://asia.ensembl.org/Homo_sapiens/Gene/Idhistory?g=ENSG00000237595
ENSG00000251432.2	lincRNA	chr4:129213906-129396151	+	2.9512229	4.0522767	6.3954375	34.150475	2.4939681	0.0399	None	http://asia.ensembl.org/Homo_sapiens/Gene/Idhistory?g=ENSG00000251432
LOC101928725	lncRNA	chr15:59060271-59063173	-	2.9610412	0.0033471	13.741659	8.5067629	2.7994991	0.0399	uncharacterized LOC101928725	http://www.ncbi.nlm.nih.gov/gene/?term=101928725

gene	gene_type	Position	Strand	A375P	A375+DMSO	A375R	A375+VEM	log2(Fold_change)	p-value	Description	Website
LINC01429	lncRNA	chr20:50448336-50479452	-	2.9498577	5.0640843	1.39E-17	1.39E-17	-5.047098	0.0399	long intergenic non-protein coding RNA 1429	http://www.ncbi.nlm.nih.gov/gene/?term=101927678
FLJ37035	lncRNA	chr10:127393859-127408062	-	2.9498577	5.0640843	5.3411682	43.63287	2.5764235	0.0404	uncharacterized LOC399821	http://www.ncbi.nlm.nih.gov/gene/?term=399821
ENSG00000272459.1	lincRNA	chr5:176981825-176982365	+	0.9834014	1.0142839	9.5149397	6.6163298	2.863997	0.041	None	http://asia.ensembl.org/Homo_sapiens/Gene/Idhistory?g=ENSG00000272459
LINC00346	lncRNA	chr13:111516334-111522655	-	21.651738	111.43945	32.854469	735.16524	2.52659	0.0413	long intergenic non-protein coding RNA 346	http://www.ncbi.nlm.nih.gov/gene/?term=283487
LOC101929452	lncRNA	chr2:7202858-7218011	-	0.9775497	5.0599257	4.2826794	38.897132	2.7909113	0.0413	uncharacterized LOC101929452	http://www.ncbi.nlm.nih.gov/gene/?term=101929452
MIR4500HG	lncRNA	chr13:88096242-88323218	-	21.671631	10.138916	4.2193018	1.8765303	-2.343737	0.0415	MIR4500 host gene	http://www.ncbi.nlm.nih.gov/gene/?term=642345
MGC16025	lncRNA	chr2:240115027-240117153	-	4.9120707	25.318757	26.500108	118.4596	2.2531749	0.0416	uncharacterized LOC85009	http://www.ncbi.nlm.nih.gov/gene/?term=85009
LOC90768	lncRNA	chr4:183059811-183065668	-	332.85803	321.18851	326.97396	1834.9315	1.7245409	0.0416	uncharacterized LOC90768	http://www.ncbi.nlm.nih.gov/gene/?term=90768

gene	gene_type	Position	Strand	A375P	A375+DMSO	A375R	A375+VEM	log2(Fold_change)	p-value	Description	Website
ENSG00000249364.1	lincRNA	chr5:66675206-67101066	+	0	1.0115835	1.10044	16.156556	3.8067265	0.0423	None	http://asia.ensembl.org/Homo_sapiens/Gene/Idhistory?g=ENSG00000249364
LINC00323	lncRNA	chr21:42513427-42519991	-	24.637924	1.023602	2.0968244	0	-3.492124	0.0425	long intergenic non-protein coding RNA 323	http://www.ncbi.nlm.nih.gov/gene/?term=284835
ENSG00000272733.1	lincRNA	chr22:23923067-23926046	-	1.39E-17	1.39E-17	0.03124	13.318175	5.7680677	0.0433	None	http://asia.ensembl.org/Homo_sapiens/Gene/Idhistory?g=ENSG00000272733
ENSG00000227911.3	lincRNA	chr13:33907816-33909414	-	1.39E-17	1.39E-17	0.03124	13.318175	5.7680671	0.0433	None	http://asia.ensembl.org/Homo_sapiens/Gene/Idhistory?g=ENSG00000227911
STARD4-AS1	lncRNA	chr5:110847924-111075423	+	16.726635	372.84093	348.13156	1623.6842	2.3389028	0.0433	STARD4 antisense RNA 1	http://www.ncbi.nlm.nih.gov/gene/?term=100505678
ENSG00000264956.1	lincRNA	chr17:21793031-21814739	+	9.8421902	16.208263	0.0172203	3.8168628	-2.664377	0.0438	None	http://asia.ensembl.org/Homo_sapiens/Gene/Idhistory?g=ENSG00000264956
LOC105376554	lncRNA	chr11:12052422-12083332	+	1.956635	33.41964	20.158582	200.89069	2.6353149	0.0444	None	http://www.ncbi.nlm.nih.gov/gene/?term=LOC105376554

gene	gene_type	Position	Strand	A375P	A375+DMSO	A375R	A375+VEM	log2(Fold_change)	p-value	Description	Website
BVES-AS1	lncRNA	chr6:105585562-105617820	+	0.9768041	6.0719379	5.340565	40.789431	2.6705243	0.0444	BVES antisense RNA 1	http://www.ncbi.nlm.nih.gov/gene/?term=154442
ENSG00000233942.1	lincRNA	chr7:95101147-95103310	+	1.39E-17	1.39E-17	2.1405164	5.7004547	5.0226205	0.0453	None	http://asia.ensembl.org/Homo_sapiens/Gene/Idhistory?g=ENSG000002[illegible]
ENSG00000234698.1	lincRNA	chr20:48782899-48788279	+	1.39E-17	1.39E-17	2.1405164	5.7004547	5.0226196	0.0453	None	http://asia.ensembl.org/Homo_sapiens/Gene/Idhistory?g=ENSG000002[illegible]
ENSG00000204792.2	lincRNA	chr2:75145275-75159921	+	760.27709	88.162252	154.39352	16.999829	-2.306138	0.0472	None	http://asia.ensembl.org/Homo_sapiens/Gene/Idhistory?g=ENSG000002[illegible]
ENSG00000230836.1	lincRNA	chr2:75167385-75169797	+	2048.3833	185.43041	431.58787	54.890151	-2.198615	0.049	None	http://asia.ensembl.org/Homo_sapiens/Gene/Idhistory?g=ENSG000002[illegible]
ENSG00000223401.1	lincRNA	chr3:187461474-187463208	+	3.9329795	8.101931	13.7928	38.871184	2.1090166	0.0495	None	http://asia.ensembl.org/Homo_sapiens/Gene/Idhistory?g=ENSG000002[illegible]
SNHG15	lncRNA	chr7:45022627-45026259	-	1520.5306	330.31791	422.0708	66.258608	-1.921962	0.0497	small nucleolar RNA host gene 15	http://www.ncbi.nlm.nih.gov/gene/?term=285958

gene	gene_type	Position	Strand	A375P	A375+DMSO	A375R	A375+VEM	log2(Fold_change)	p-value	Description	Website
ENSG00000262223.2	lincRNA	chr17:79353398-79359046	-	1.39E-17	1.39E-17	0.0304689	12.369316	5.6639021	0.0497	None	http://asia.ensembl.org/Homo_sapiens/Gene/Idhistory?g=ENSG00000262223
ENSG00000228692.2	lincRNA	chr6:164007615-164009197	-	1.39E-17	1.39E-17	0.0304689	12.369316	5.6639008	0.0497	None	http://asia.ensembl.org/Homo_sapiens/Gene/Idhistory?g=ENSG00000228692

Supplementary Table S2. The list of differentially expressed genes in response to LINC01198 knockout in A375.

id	CON_Mean	KD_Mean	AllMean	log2FoldChange	pvalue	qvalue	State	gene_id	XF_1_count	XF_1_FPKM	XF_1_TPM	XF_1_CPM	XF_2_count	XF_2_FPKM	XF_2_TPM	XF_2_CPM	XF_3_count	XF_3_FPKM	XF_3_TPM	XF_3_CPM	Symbol	Description	KEGG_A_class	KEGG_B_class	Pathway	K_ID	GO Component	GO Function	GO Process
IGFBP5	6.54396962603947	2.65589020839232	4.59992991721589	-3.89638	6.90326340854898E-33	1.17783480276663E-28	Down	ENSG00000115461	3115	14.522409831	54.832439932	93.901901964	209	1.076880556	4.324886983	6.963109678	160	0.83926	3.376259109	5.42665516	IGFBP5	insulin like growth factor binding protein 5 [Source:HGNC Symbol;Acc:HGNC:5474]	-	-	-	-	GO:0005576//extracellular region;GO:0005783//endoplasmic reticulum;GO:0043227//membrane-bounded organelle;GO:0032991//macromolecular complex;GO:0012505//endomembrane system;GO:0005623//cell;GO:0044424//intracellular part;GO:0044421//extracellular region part;GO:0043229//intracellular organelle;GO:0043226//organelle;GO:0005615//extracellular space;GO:0005622//intracellular;GO:0044444//cytoplasmic part;GO:0044464//cell part;GO:0005737//cytoplasm;GO:0005575//cellular_component;GO:0043231//intracellular membrane-bounded organelle	GO:0003674//molecular_function	GO:0019538//protein metabolic process;GO:0051179//localization;GO:0010467//gene expression;GO:0044267//cellular protein metabolic process;GO:0006412//translation;GO:0034645//cellular macromolecule biosynthetic process;GO:0006807//nitrogen compound metabolic process;GO:0043043//peptide biosynthetic process;GO:0042592//homeostatic process;GO:0030154//cell differentiation;GO:1901576//organic substance biosynthetic process;GO:0065008//regulation of biological quality;GO:0007165//signal transduction;GO:0044271//cellular nitrogen compound biosynthetic process;GO:0040007//growth;GO:0048856//anatomical structure development;GO:1901564//organonitrogen compound metabolic process;GO:1901566//organonitrogen compound biosynthetic process;GO:0007154//cell communication;GO:0044767//single-organism developmental process;GO:0051674//localization of cell;GO:0008150//biological_process;GO:0043604//amide biosynthetic process;GO:0007568//aging;GO:0065007//biological regulation;GO:0006928//movement of cell or [illegible]

id	CON_Mean	KD_Mean	AllMean	log2FoldChange	pvalue	qvalue	State	gene_id	XF_1_count	XF_1_FPKM	XF_1_TPM	XF_1_CPM	XF_2_count	XF_2_FPKM	XF_2_TPM	XF_2_CPM	XF_3_count	XF_3_FPKM	XF_3_TPM	XF_3_CPM	Symbol	Description	KEGG_A_class	KEGG_B_class	Pathway	K_ID	GO Component	GO Function	GO Process
MUC5B	4.317378957	7.469352234	5.893365595	3.166500272	2.907307668773 4E-22	2.480224172230 61E-18	Up	ENSG00000117983	663	1.074641537	4.057537159	19.986183307	4707	8.43208362	33.864302285	156.819891172	5790	10.559043102	42.477975184	196.377083615	MUC5B	mucin 5B, oligomeric mucus/gel-forming [Source:HGNC Symbol;Acc:HGNC:7516]	Organismal Systems	Digestive system	ko04970//Salivary secretion	K13908	GO:0005737//cytoplasm;GO:0044424//intracellular part;GO:0043229//intracellular organelle;GO:0005886//plasma membrane;GO:0005615//extracellular space;GO:0005622//intracellular;GO:0043227//membrane-bounded organelle;GO:0005576//extracellular region;GO:0005794//Golgi apparatus;GO:0043231//intracellular membrane-bounded organelle;GO:0005575//cellular_component;GO:0044444//cytoplasmic part;GO:0044421//extracellular region part;GO:0012505//endomembrane system;GO:0005623//cell;GO:0071944//cell periphery;GO:0043226//organelle;GO:0044464//cell part;GO:0016020//membrane	GO:0003674//molecular_function	GO:0009058//biosynthetic process;GO:0008152//metabolic process;GO:0044267//cellular protein metabolic process;GO:0044260//cellular macromolecule metabolic process;GO:0023052//signaling;GO:0044699//single-organism process;GO:0043170//macromolecule metabolic process;GO:0050789//regulation of biological process;GO:0050794//regulation of cellular process;GO:0006950//response to stress;GO:0051716//cellular response to stimulus;GO:0044237//cellular metabolic process;GO:0043412//macromolecule modification;GO:0044763//single-organism cellular process;GO:0019538//protein metabolic process;GO:0071704//organic substance metabolic process;GO:0044238//primary metabolic process;GO:0044700//single organism signaling;GO:0007154//cell communication;GO:0002376//immune system process;GO:0065007//biological regulation;GO:0036211//protein modification process;GO:0006464//cellular protein modification process;GO:0007165//signal transduction;GO:0050896//response to [illegible]
TMPRSS15	3.48532796	-2.0305	0.727411568	-6.14768	9.773621996366 93E-19	5.558584616733 75E-15	Down	ENSG00000154646	371	2.563920686	9.680626607	11.183822032	6	0.045827117	0.184047434	0.199897885	3	0.023326406	0.093839801	0.101749784	TMPRSS15	transmembrane serine protease 15 [Source:HGNC Symbol;Acc:HGNC:9490]	-	-	-	-	GO:0005575//cellular_component	GO:0003824//catalytic activity;GO:0008233//peptidase activity;GO:0003674//molecular_function;GO:0016787//hydrolase activity	GO:0051179//localization;GO:0006810//transport;GO:0051234//establishment of localization;GO:0008150//biological_process;GO:0016192//vesicle-mediated transport

id	CON_Mean	KD_Mean	AllMean	log2FoldChange	pvalue	qvalue	State	gene_id	XF_1_count	XF_1_FPKM	XF_1_TPM	XF_1_CPM	XF_2_count	XF_2_FPKM	XF_2_TPM	XF_2_CPM	XF_3_count	XF_3_FPKM	XF_3_TPM	XF_3_CPM	Symbol	Description	KEGG_A_class	KEGG_B_class	Pathway	K_ID	GO Component	GO Function	GO Process
TXNIP	7.203721836	5.046113447	6.124917641	-2.15955	1.44250471802444E-17	6.15300387473324E-14	Down	ENSG00000265972	4923	41.177634211	155.47489575	148.404193698	1017	9.401412761	37.757249333	33.882691592	927	8.723830004	35.095096292	31.440683335	TXNIP	thioredoxin interacting protein [Source:HGNC Symbol;Acc:HGNC:16952]	-	-	-	-	GO:0005575//cellular_component;GO:0044444//cytoplasmic part;GO:0043229//intracellular organelle;GO:0043231//intracellular membrane-bounded organelle;GO:0044464//cell part;GO:0005634//nucleus;GO:0005737//cytoplasm;GO:0005829//cytosol;GO:0005622//intracellular;GO:0043226//organelle;GO:0043227//membrane-bounded organelle;GO:0005623//cell;GO:0005739//mitochondrion;GO:0044424//intracellular part	GO:0005515//protein binding;GO:0019899//enzyme binding;GO:0005488//binding;GO:0030234//enzyme regulator activity;GO:0003674//molecular_function;GO:0098772//molecular function regulator	GO:0051301//cell division;GO:0051716//cellular response to stimulus;GO:0008283//cell proliferation;GO:0030154//cell differentiation;GO:0006807//nitrogen compound metabolic process;GO:0044699//single-organism process;GO:0045184//establishment of protein localization;GO:0050896//response to stimulus;GO:0008104//protein localization;GO:0051179//localization;GO:0006950//response to stress;GO:0033036//macromolecule localization;GO:0065007//biological regulation;GO:0051234//establishment of localization;GO:0015031//protein transport;GO:0051169//nuclear transport;GO:0008150//biological_process;GO:0044767//single-organism developmental process;GO:0051641//cellular localization;GO:0007165//signal transduction;GO:0048856//anatomical structure development;GO:0044237//cellular metabolic process;GO:0023052//signaling;GO:0034641//cellular nitrogen compound metabolic process;GO:0006913//nucleocytoplasmic [illegible]
CT45A3	3.093559874	-2.02924	0.532157998	-5.75052	2.39844848862956E-16	8.08071989711147E-13	Down	ENSG00000269096	282	7.290660846	27.527437085	8.500910547	3	0.085719505	0.344260251	0.099948943	6	0.174527932	0.702108428	0.203499569	CT45A3	cancer/testis antigen family 45 member A3 [Source:HGNC Symbol;Acc:HGNC:33268]	-	-	-	-	GO:0043229//intracellular organelle;GO:0005622//intracellular;GO:0005575//cellular_component;GO:0005634//nucleus;GO:0032991//macromolecular complex;GO:0043226//organelle;GO:0043231//intracellular membrane-bounded organelle;GO:0043227//membrane-bounded organelle;GO:0044464//cell part;GO:0044424//intracellular part;GO:0005623//cell	GO:0003674//molecular_function	GO:0008150//biological_process;GO:0008152//metabolic process;GO:0009987//cellular process;GO:0006807//nitrogen compound metabolic process;GO:0034641//cellular nitrogen compound metabolic process;GO:0044237//cellular metabolic process

id	CON_Mean	KD_Mean	AllMean	log2FoldChange	pvalue	qvalue	State	gene_id	XF_1_count	XF_1_FPKM	XF_1_TPM	XF_1_CPM	XF_2_count	XF_2_FPKM	XF_2_TPM	XF_2_CPM	XF_3_count	XF_3_FPKM	XF_3_TPM	XF_3_CPM	Symbol	Description	KEGG_A_class	KEGG_B_class	Pathway	K_ID	GO Component	GO Function	GO Process
ENC1	4.343132237505559	6.803056206729775	5.573094222117667	2.465380405692255	2.841655103895722E-16	8.080719897111437E-13	Up	ENSG000001716117	675	2.456885315	9.276491853	20.347924182	3293	13.246875492	53.201108549	109.710622824	3284	13.448695625	54.102758506	111.382097166	ENC1	ectodermal-neural cortex 1 [Source:HGNC Symbol;Acc:HGNC:3345]	-	-	-	-	GO:0005623//cell;GO:0043226//organelle;GO:0005654//nucleoplasm;GO:0043229//intracellular organelle;GO:0005575//cellular_component;GO:0070013//intracellular organelle lumen;GO:0044446//intracellular organelle part;GO:0044424//intracellular part;GO:0005634//nucleus;GO:0032991//macromolecular complex;GO:0044464//cell part;GO:0000228//nuclear chromosome;GO:0031981//nuclear lumen;GO:0005694//chromosome;GO:0031974//membrane-enclosed lumen;GO:0005737//cytoplasm;GO:0005856//cytoskeleton;GO:0044422//organelle part;GO:0043231//intracellular membrane-bounded organelle;GO:0043232//intracellular non-membrane-bounded organelle;GO:0043233//organelle lumen;GO:0044428//nuclear part;GO:0043228//non-membrane-bounded organelle;GO:0043227//membrane-bounded organelle;GO:0005730//nucleolus;GO:0005622//intracellular	GO:0008092//cytoskeletal protein binding;GO:0003674//molecular_function;GO:0005488//binding;GO:0005515//protein binding	GO:0048869//cellular developmental process;GO:0044238//primary metabolic process;GO:0044699//single-organism process;GO:0044237//cellular metabolic process;GO:0009059//macromolecule biosynthetic process;GO:0009058//biosynthetic process;GO:0044767//single-organism developmental process;GO:0043043//peptide biosynthetic process;GO:1901566//organonitrogen compound biosynthetic process;GO:0044271//cellular nitrogen compound biosynthetic process;GO:0010467//gene expression;GO:0044260//cellular macromolecule metabolic process;GO:0048856//anatomical structure development;GO:1901576//organic substance biosynthetic process;GO:0019538//protein metabolic process;GO:0043603//cellular amide metabolic process;GO:0044249//cellular biosynthetic process;GO:0009987//cellular process;GO:0043170//macromolecule metabolic process;GO:0034645//cellular macromolecule biosynthetic process;GO:0044267//cellular protein metabolic process;GO:0009056//catabolic [illegible]

id	CON_Mean	KD_Mean	AllMean	log2FoldChange	pvalue	qvalue	State	gene_id	XF_1_count	XF_1_FPKM	XF_1_TPM	XF_1_CPM	XF_2_count	XF_2_FPKM	XF_2_TPM	XF_2_CPM	XF_3_count	XF_3_FPKM	XF_3_TPM	XF_3_CPM	Symbol	Description	KEGG_A_class	KEGG_B_class	Pathway	K_ID	GO Component	GO Function	GO Process
NDN	3.368969035	-1.28212	1.043426525	-4.97032	6.65702E-15	1.6226E-11	Down	ENSG00000182636	342	5.409031962	20.422947953	10.309614919	7	0.12235792	0.491404717	0.2332142	12	0.213535749	0.859032977	0.406999137	NDN	necdin, MAGE family member [Source:HGNC Symbol;Acc:HGNC:7675]	-	-	-	-	GO:0043229//intracellular organelle;GO:0005737//cytoplasm;GO:0005829//cytosol;GO:0043233//organelle lumen;GO:0005622//intracellular;GO:0044428//nuclear part;GO:0005856//cytoskeleton;GO:0043228//non-membrane-bounded organelle;GO:0005634//nucleus;GO:0044430//cytoskeletal part;GO:0043226//organelle;GO:0005654//nucleoplasm;GO:0043232//intracellular non-membrane-bounded organelle;GO:0031974//membrane-enclosed lumen;GO:0005575//cellular_component;GO:0031981//nuclear lumen;GO:0005623//cell;GO:0044464//cell part;GO:0044422//organelle part;GO:0044424//intracellular part;GO:0005815//microtubule organizing center;GO:0043231//intracellular membrane-bounded organelle;GO:0070013//intracellular organelle lumen;GO:0015630//microtubule cytoskeleton;GO:0044446//intracellular organelle part;GO:0044444//cytoplasmic part;GO:0043227//membrane-bounded organelle	GO:0003674//molecular_function;GO:0008092//cytoskeletal protein binding;GO:0003676//nucleic acid binding;GO:0003700//transcription factor activity, sequence-specific DNA binding;GO:0005515//protein binding;GO:0001071//nucleic acid binding transcription factor activity;GO:0003677//DNA binding;GO:0005488//binding;GO:1901363//heterocyclic compound binding;GO:0097159//organic cyclic compound binding	GO:0032501//multicellular organismal process;GO:0071840//cellular component organization or biogenesis;GO:0009653//anatomical structure morphogenesis;GO:0048870//cell motility;GO:0044700//single organism signaling;GO:0008150//biological_process;GO:0044237//cellular metabolic process;GO:0009987//cellular process;GO:0032502//developmental process;GO:0044767//single-organism developmental process;GO:0050789//regulation of biological process;GO:0048869//cellular developmental process;GO:0003008//system process;GO:0040011//locomotion;GO:0009058//biosynthetic process;GO:0050794//regulation of cellular process;GO:0016043//cellular component organization;GO:0008283//cell proliferation;GO:0044699//single-organism process;GO:0042592//homeostatic process;GO:0051716//cellular response to stimulus;GO:0048856//anatomical structure development;GO:0007154//cell communication;GO:0000902//cell morphogenesis;GO:004000 [illegible]

id	CON_Mean	KD_Mean	AllMean	log2FoldChange	pvalue	qvalue	State	gene_id	XF_1_count	XF_1_FPKM	XF_1_TPM	XF_1_CPM	XF_2_count	XF_2_FPKM	XF_2_TPM	XF_2_CPM	XF_3_count	XF_3_FPKM	XF_3_TPM	XF_3_CPM	Symbol	Description	KEGG_A_class	KEGG_B_class	Pathway	K_ID	GO Component	GO Function	GO Process
MAOA	3.326565714	-0.94	1.193283641	-4.53705	7.96651E-14	1.69906E-10	Down	ENSG00000189221	332	1.810449383	6.835735819	10.00816419	13	0.0783487855	0.31465852	0.4331120855	12	0.0736250255	0.2961861177	0.4069991377	MAOA	monoamine oxidase A [Source:HGNC Symbol;Acc:HGNC:6833]	Metabolism; Organismal Systems; Human Diseases	Amino acid metabolism; Xenobiotics biodegradation and metabolism; Nervous system; Substance dependence	ko05034//Alcoholism;ko04728//Dopaminergic synapse;ko04726//Serotonergic synapse;ko00982//Drug metabolism - cytochrome P450;ko05031//Amphetamine addiction;ko05030//Cocaine [illegible]	K00274	GO:0005739//mitochondrion;GO:0043227//membrane-bounded organelle;GO:0005737//cytoplasm;GO:0043231//intracellular membrane-bounded organelle;GO:0005623//cell;GO:0044424//intracellular part;GO:0043226//organelle;GO:0043229//intracellular organelle;GO:0005829//cytosol;GO:0044444//cytoplasmic part;GO:0005575//cellular_component;GO:0005622//intracellular;GO:0044464//cell part	GO:0003824//catalytic activity;GO:0003674//molecular_function;GO:0016491//oxidoreductase activity	GO:0023052//signaling;GO:0051716//cellular response to stimulus;GO:0006807//nitrogen compound metabolic process;GO:0034641//cellular nitrogen compound metabolic process;GO:0044699//single-organism process;GO:0065007//biological regulation;GO:0044237//cellular metabolic process;GO:0050794//regulation of cellular process;GO:0050789//regulation of biological process;GO:0009987//cellular process;GO:0008150//biological_process;GO:0007165//signal transduction;GO:0044763//single-organism cellular process;GO:0007154//cell communication;GO:0044700//single organism signaling;GO:0008152//metabolic process;GO:0009056//catabolic process;GO:0050896//response to stimulus
LINC01198	3.047308458	-1.89469	0.576306897	-5.29674	2.5337E-13	4.80334E-10	Down	ENSG00000231817	273	1.446327749	5.460917323	8.229604891	2	0.01171048	0.04703075	0.0666326288	10	0.0596073722	0.2397945011	0.3391659148	-	-	-	-	-	-	-	-	-
LINC02582	2.994116716	-1.39955	0.797281072	-4.67124	2.72266E-12	4.6454E-09	Down	ENSG00000261780	263	3.18016613	12.007392047	7.928154163	13	0.1737312820	0.6977265530	0.4331120855	5	0.0680236560	0.2736523690	0.1695829740	-	-	-	-	-	-	-	-	-

id	CON_Mean	KD_Mean	AllMean	log2FoldChange	pvalue	qvalue	State	gene_id	XF_1_count	XF_1_FPKM	XF_1_TPM	XF_1_CPM	XF_2_count	XF_2_FPKM	XF_2_TPM	XF_2_CPM	XF_3_count	XF_3_FPKM	XF_3_TPM	XF_3_CPM	Symbol	Description	KEGG_A_class	KEGG_B_class	Pathway	K_ID	GO Component	GO Function	GO Process
ITGB4	5.259662642	2.248830497	3.75424657	-3.0004	1.52346E-11	2.36302E-08	Down	ENSG00000132470	1277	5.693722533	21.4978576 15	38.4952580 44	110	0.542049189	2.176937329	3.664794567	171	0.857822467	3.45093406	5.799737703	ITGB4	integrin subunit beta 4 [Source:HGNC Symbol;Acc:HGNC:6158]	Cellular Processes;Human Diseases;Environmental Information Processing	Signal transduction;Signaling molecules and interaction;Cellular community;Cardiovascular diseases;Cell motility	ko04151//PI3K-Akt signaling pathway;ko04510//Focal adhesion;ko04810//Regulation of actin cytoskeleton;ko04512//ECM-receptor interaction;ko05414//Dilated cardiomyopathy (DCM);ko05410/[illegible]	K06525	GO:0016020//membrane;GO:0032991//macromolecular complex;GO:0012505//endomembrane system;GO:0044422//organelle part;GO:0005623//cell;GO:0044421//extracellular region part;GO:0031974//membrane-enclosed lumen;GO:0005730//nucleolus;GO:0071944//cell periphery;GO:0043229//intracellular organelle;GO:0043227//membrane-bounded organelle;GO:0005635//nuclear envelope;GO:0005615//extracellular space;GO:0005576//extracellular region;GO:0005886//plasma membrane;GO:0005622//intracellular;GO:0043228//non-membrane-bounded organelle;GO:0005575//cellular_component;GO:0044428//nuclear part;GO:0043233//organelle lumen;GO:0031975//envelope;GO:0031981//nuclear lumen;GO:0044446//intracellular organelle part;GO:0031967//organelle envelope;GO:0070013//intracellular organelle lumen;GO:0043232//intracellular non-membrane-bounded organelle;GO:0005634//nucleus;GO:0044424//intracellular part;GO:0043231//intracellular membrane-bounded organelle;GO:0043226//organelle;GO:0044464//cell part	GO:0003674//molecular_function	GO:0048856//anatomical structure development;GO:0048869//cellular developmental process;GO:0051179//localization;GO:0009653//anatomical structure morphogenesis;GO:0030154//cell differentiation;GO:0048646//anatomical structure formation involved in morphogenesis;GO:0006928//movement of cell or subcellular component;GO:0009790//embryo development;GO:0030198//extracellular matrix organization;GO:0051716//cellular response to stimulus;GO:0006950//response to stress;GO:0065007//biological regulation;GO:0009056//catabolic process;GO:0032501//multicellular organismal process;GO:0008152//metabolic process;GO:0044700//single organism signaling;GO:0071840//cellular component organization or biogenesis;GO:0044085//cellular component biogenesis;GO:0050896//response to stimulus;GO:0044767//single-organism developmental process;GO:0009987//cellular process;GO:0006914//autophagy;GO:0022607//cellular component assembly;GO:0044707//sin[illegible]
GS1-600G8.3	1.55592107	4.772986637	3.164453853	3.258732613	1.82039E-11	2.58829E-08	Up	ENSG00000231216	95	1.273357902	4.807832963	2.863781922	801	11.8658815 98	47.6548643 73	26.6863677 14	805	12.1399994 56	48.8380046 01	27.3028587 76	-	-	-	-	-	-	-	-	-

id	CON_Mean	KD_Mean	AllMean	log2FoldChange	pvalue	qvalue	State	gene_id	XF_1_count	XF_1_FPKM	XF_1_TPM	XF_1_CPM	XF_2_count	XF_2_FPKM	XF_2_TPM	XF_2_CPM	XF_3_count	XF_3_FPKM	XF_3_TPM	XF_3_CPM	Symbol	Description	KEGG_A_class	KEGG_B_class	Pathway	K_ID	GO Component	GO Function	GO Process
IL1B	2.765621162	-1.14135	0.8121357771	-4.21861	2.12182E-11	2.78481E-08	Down	ENSG00000125538	224	2.244099808	8.473075016	6.752496321	11	0.121794436	0.489141684	0.366479457	10	0.112717164	0.453449888	0.339165948	IL1B	interleukin 1 beta [Source:HGNC Symbol;Acc:HGNC:5992]	Organismal Systems;Cellular Processes;Human Diseases;Environmental Information Processing	Endocrine and metabolic diseases;Cell growth and death;Sensory system;Immune system;Infectious diseases;Signal transduction;Development;Immune diseases;Signaling molecules and inter	ko05168//Herpes simplex infection;ko04060//Cytokine-cytokine receptor interaction;ko05332//Graft-versus-host disease;ko04010//MAPK signaling pathway;ko05164//Influenza A;ko05152//Tub	K04519	GO:0031982//vesicle;GO:0005623//cell;GO:0043226//organelle;GO:0044464//cell part;GO:0005576//extracellular region;GO:0097708//intracellular vesicle;GO:0031410//cytoplasmic vesicle;GO:0005829//cytosol;GO:0005615//extracellular space;GO:0043227//membrane-bounded organelle;GO:0005622//intracellular;GO:0044444//cytoplasmic part;GO:0005764//lysosome;GO:0043231//intracellular membrane-bounded organelle;GO:0005773//vacuole;GO:0044421//extracellular region part;GO:0005737//cytoplasm;GO:0000323//lytic vacuole;GO:0005575//cellular_component;GO:0044424//intracellular part;GO:0043229//intracellular organelle	GO:0003674//molecular_function	GO:0006913//nucleocytoplasmic transport;GO:0050794//regulation of cellular process;GO:0006928//movement of cell or subcellular component;GO:0007154//cell communication;GO:0006950//response to stress;GO:0032502//developmental process;GO:0071840//cellular component organization or biogenesis;GO:0007155//cell adhesion;GO:0048856//anatomical structure development;GO:0055085//transmembrane transport;GO:0044085//cellular component biogenesis;GO:0051716//cellular response to stimulus;GO:0044260//cellular macromolecule metabolic process;GO:0006807//nitrogen compound metabolic process;GO:0065008//regulation of biological quality;GO:0002376//immune system process;GO:0044700//single organism signaling;GO:0044710//single-organism metabolic process;GO:0007267//cell-cell signaling;GO:0009058//biosynthetic process;GO:0046907//intracellular transport;GO:0008283//cell proliferation;GO:0044238//primary metabolic process;GO:0071704//orga
MIR100HG	8.090246658	6.40342504	7.246835849	-1.68003	9.8098E-11	1.19553E-07	Down	ENSG00000255248	9104	9.579417897	36.169124997	274.440743333	2256	2.62353328	10.536437737	75.161604947	2753	3.259184801	13.111374747	93.372385353	-	-	-	-	-	-	-	-	-

id	CON_Mean	KD_Mean	AllMean	log2FoldChange	pvalue	qvalue	State	gene_id	XF_1_count	XF_1_FPKM	XF_1_TPM	XF_1_CPM	XF_2_count	XF_2_FPKM	XF_2_TPM	XF_2_CPM	XF_3_count	XF_3_FPKM	XF_3_TPM	XF_3_CPM	Symbol	Description	KEGG_A_class	KEGG_B_class	Pathway	K_ID	GO Component	GO Function	GO Process
TFAP2B	6.201452187	3.078510165	4.639981176	-2.97121	1.56259E-10	1.77739E-07	Down	ENSG00000008196	2456	12.028643209	45.41669488	74.0362989 48	151	0.817345809	3.282562979	5.030763452	400	2.204165378	8.867137042	13.5666379 01	TFAP2B	transcription factor AP-2 beta [Source:HGNC Symbol;Acc:HGNC:11743]	-	-	-	-	GO:0031981//nuclear lumen;GO:0070013//intracellular organelle lumen;GO:0044428//nuclear part;GO:0044422//organelle part;GO:0043233//organelle lumen;GO:0005654//nucleoplasm;GO:0043231//intracellular membrane-bounded organelle;GO:0005575//cellular_component;GO:0005634//nucleus;GO:0000228//nuclear chromosome;GO:0043229//intracellular organelle;GO:0043226//organelle;GO:0005694//chromosome;GO:0005622//intracellular;GO:0044424//intracellular part;GO:0044446//intracellular organelle part;GO:0043228//non-membrane-bounded organelle;GO:0044464//cell part;GO:0043227//membrane-bounded organelle;GO:0005623//cell;GO:0043232//intracellular non-membrane-bounded organelle;GO:0031974//membrane-enclosed lumen	GO:0030234//enzyme regulator activity;GO:0003677//DNA binding;GO:0003676//nucleic acid binding;GO:0005488//binding;GO:0003674//molecular_function;GO:1901363//heterocyclic compound binding;GO:0001071//nucleic acid binding transcription factor activity;GO:0003700//transcription factor activity, sequence-specific DNA binding;GO:0098772//molecular function regulator;GO:0097159//organic cyclic compound binding	GO:0008219//cell death;GO:0008152//metabolic process;GO:0033036//macromolecule localization;GO:0009987//cellular process;GO:0044700//single organism signaling;GO:0006810//transport;GO:0007154//cell communication;GO:0007165//signal transduction;GO:0071702//organic substance transport;GO:0044767//single-organism developmental process;GO:0023052//signaling;GO:0051716//cellular response to stimulus;GO:0071704//organic substance metabolic process;GO:0051234//establishment of localization;GO:0044710//single-organism metabolic process;GO:0044763//single-organism cellular process;GO:0044237//cellular metabolic process;GO:0034641//cellular nitrogen compound metabolic process;GO:0050896//response to stimulus;GO:0030154//cell differentiation;GO:0050789//regulation of biological process;GO:0050794//regulation of cellular process;GO:0065007//biological regulation;GO:0009058//biosynthetic process;GO:0008283//cell proliferation;GO:0015031//protein [illegible]

id	CON_Mean	KD_Mean	AllMean	log2FoldChange	pvalue	qvalue	State	gene_id	XF_1_count	XF_1_FPKM	XF_1_TPM	XF_1_CPM	XF_2_count	XF_2_FPKM	XF_2_TPM	XF_2_CPM	XF_3_count	XF_3_FPKM	XF_3_TPM	XF_3_CPM	Symbol	Description	KEGG_A_class	KEGG_B_class	Pathway	K_ID	GO Component	GO Function	GO Process
PLD1	5.888188505	3.744128261	4.816158383	-2.12715	7.13396E-10	7.60748E-07	Down	ENSG00000075651	1976	4.631933435	17.4888475 68	59.5666639 75	327	0.847156669	3.402287122	10.89443476	470	1.239564505	4.986644128	15.940799534	PLD1	phospholipase D1 [Source:HGNC Symbol;Acc:HGNC:9067]	Organismal Systems;Metabolism;Cellular Processes;Human Diseases;Environmental Information Processing	Lipid metabolism; Signal transduction; Nervous system;Immune system; Transport and catabolism;Endocrine system; Cancers	ko05200//Pathways in cancer;ko04144//Endocytosis; ko04014//Ras signaling pathway; ko04024//cAMP signaling pathway; ko04071//Sphingolipid signaling pathway; ko04724//Glutamatergi	K01115	GO:0005575//cellular_component;GO:0043231//intracellular membrane-bounded organelle;GO:0044464//cell part;GO:0097708//intracellular vesicle;GO:0005623//cell;GO:0031982//vesicle;GO:0005773//vacuole;GO:0012505//endomembrane system;GO:0043226//organelle;GO:0000323//lytic vacuole;GO:0005764//lysosome;GO:0016020//membrane;GO:0044424//intracellular part;GO:0005794//Golgi apparatus;GO:0071944//cell periphery;GO:0005886//plasma membrane;GO:0005768//endosome;GO:0043229//intracellular organelle;GO:0044444//cytoplasmic part;GO:0005622//intracellular;GO:0031410//cytoplasmic vesicle;GO:0005783//endoplasmic reticulum;GO:0005737//cytoplasm;GO:0043227//membrane-bounded organelle	GO:0003674//molecular_function;GO:0043167//ion binding;GO:0008289//lipid binding;GO:0005488//binding	GO:0071840//cellular component organization or biogenesis;GO:0044260//cellular macromolecule metabolic process;GO:0044085//cellular component biogenesis;GO:0010467//gene expression;GO:0009056//catabolic process;GO:0002376//immune system process;GO:0050789//regulation of biological process;GO:0044271//cellular nitrogen compound biosynthetic process;GO:0051674//localization of cell;GO:0016192//vesicle-mediated transport;GO:0009059//macromolecule biosynthetic process;GO:0065007//biological regulation;GO:0050794//regulation of cellular process;GO:0051234//establishment of localization;GO:0006629//lipid metabolic process;GO:0009987//cellular process;GO:0044267//cellular protein metabolic process;GO:0050896//response to stimulus;GO:0044710//single-organism metabolic process;GO:0023052//signaling;GO:0006412//translation;GO:0044249//cellular biosynthetic process;GO:0006518//peptide metabolic process;GO:0043043//peptide biosynthetic [illegible]

id	CON_Mean	KD_Mean	AllMean	log2FoldChange	pvalue	qvalue	State	gene_id	XF_1_count	XF_1_FPKM	XF_1_TPM	XF_1_CPM	XF_2_count	XF_2_FPKM	XF_2_TPM	XF_2_CPM	XF_3_count	XF_3_FPKM	XF_3_TPM	XF_3_CPM	Symbol	Description	KEGG_A_class	KEGG_B_class	Pathway	K_ID	GO Component	GO Function	GO Process
B3GNT7	5.806466044	3.85087617	4.828671107	-1.96238	1.34568E-09	1.35059E-06	Down	ENSG00000156966	1867	15.466021169	58.395244771	56.280851033	431	3.945955329	15.847450058	14.359331442	414	3.858606823	15.522789638	14.041470227	B3GNT7	UDP-GlcNAc:betaGal beta-1,3-N-acetylglucosaminyltransferase 7 [Source:HGNC Symbol;Acc:HGNC:18811]	Metabolism	Glycan biosynthesis and metabolism	ko00533//Glycosaminoglycan biosynthesis - keratan sulfate	K09664	GO:0044464//cell part;GO:0043227//membrane-bounded organelle;GO:0005623//cell;GO:0044424//intracellular part;GO:0044444//cytoplasmic part;GO:0043229//intracellular organelle;GO:0005783//endoplasmic reticulum;GO:0012505//endomembrane system;GO:0005575//cellular_component;GO:0005794//Golgi apparatus;GO:0043226//organelle;GO:0005737//cytoplasm;GO:0005622//intracellular;GO:0043231//intracellular membrane-bounded organelle	GO:0016757//transferase activity, transferring glycosyl groups;GO:0003824//catalytic activity;GO:0016740//transferase activity;GO:0003674//molecular_function	GO:0005975//carbohydrate metabolic process;GO:0044238//primary metabolic process;GO:0006790//sulfur compound metabolic process;GO:0044237//cellular metabolic process;GO:0008150//biological_process;GO:0009987//cellular process;GO:0044710//single-organism metabolic process;GO:0009058//biosynthetic process;GO:0071704//organic substance metabolic process;GO:0044281//small molecule metabolic process;GO:0008152//metabolic process;GO:0006464//cellular protein modification process;GO:0044267//cellular protein metabolic process;GO:0044260//cellular macromolecule metabolic process;GO:0036211//protein modification process;GO:0044699//single-organism process;GO:0043412//macromolecule modification;GO:0019538//protein metabolic process;GO:0043170//macromolecule metabolic process
ZNF385D-AS1	0.156850883	-3.35941	-1.60128	-7.97502	2.02077E-09	1.91547E-06	Down	ENSG00000225542	34	1.104453101	4.170097043	1.024932477	0	0	0	0	0	0	0	0	-	-	-	-	-	-	-	-	-
GAGE12J	0.372277791	-3.1429	-1.38531	-5.83578	5.0249E-09	4.51236E-06	Down	ENSG00000224659	40	2.228840877	8.415461685	1.205802914	0	0	0	0	1	0.062692412	0.252205307	0.033916595	-	-	-	-	-	-	-	-	-

id	CON_Mean	KD_Mean	AllMean	log2FoldChange	pvalue	qvalue	State	gene_id	XF_1_count	XF_1_FPKM	XF_1_TPM	XF_1_CPM	XF_2_count	XF_2_FPKM	XF_2_TPM	XF_2_CPM	XF_3_count	XF_3_FPKM	XF_3_TPM	XF_3_CPM	Symbol	Description	KEGG_A_class	KEGG_B_class	Pathway	K_ID	GO Component	GO Function	GO Process
CEBPD	5.630284655	3.656686409	4.643485532	-1.97245	1.28772E-08	1.02327E-05	Down	ENSG00000221869	1652	39.776086556	150.18305517	49.799660368	331	8.808067106	35.374298981	11.027700017	411	11.133962015	44.790816517	13.939720443	CEBPD	CCAAT enhancer binding protein delta [Source:HGNC Symbol;Acc:HGNC:1835]	-	-	-	-	GO:0043228//non-membrane-bounded organelle;GO:0044464//cell part;GO:0043233//organelle lumen;GO:0031974//membrane-enclosed lumen;GO:0044422//organelle part;GO:0044446//intracellular organelle part;GO:0044424//intracellular part;GO:0005634//nucleus;GO:0043231//intracellular membrane-bounded organelle;GO:0005575//cellular_component;GO:0005623//cell;GO:0070013//intracellular organelle lumen;GO:0005622//intracellular;GO:0005694//chromosome;GO:0044428//nuclear part;GO:0043227//membrane-bounded organelle;GO:0000228//nuclear chromosome;GO:0043229//intracellular organelle;GO:0043226//organelle;GO:0005654//nucleoplasm;GO:0043232//intracellular non-membrane-bounded organelle;GO:0031981//nuclear lumen	GO:1901363//heterocyclic compound binding;GO:0003700//transcription factor activity, sequence-specific DNA binding;GO:0097159//organic cyclic compound binding;GO:0001071//nucleic acid binding transcription factor activity;GO:0003676//nucleic acid binding;GO:0005488//binding;GO:0003677//DNA binding;GO:0003674//molecular_function	GO:0002376//immune system process;GO:0050789//regulation of biological process;GO:0009058//biosynthetic process;GO:0008150//biological_process;GO:0032502//developmental process;GO:0050896//response to stimulus;GO:0008152//metabolic process;GO:0044767//single-organism developmental process;GO:0034641//cellular nitrogen compound metabolic process;GO:0044237//cellular metabolic process;GO:0048869//cellular developmental process;GO:0007154//cell communication;GO:0044763//single-organism cellular process;GO:0030154//cell differentiation;GO:0044700//single organism signaling;GO:0007165//signal transduction;GO:0065007//biological regulation;GO:0006807//nitrogen compound metabolic process;GO:0009987//cellular process;GO:0044699//single-organism process;GO:0051716//cellular response to stimulus;GO:0023052//signaling;GO:0050794//regulation of cellular process;GO:0048856//anatomical structure development

id	CON_Mean	KD_Mean	AllMean	log2FoldChange	pvalue	qvalue	State	gene_id	XF_1_count	XF_1_FPKM	XF_1_TPM	XF_1_CPM	XF_2_count	XF_2_FPKM	XF_2_TPM	XF_2_CPM	XF_3_count	XF_3_FPKM	XF_3_TPM	XF_3_CPM	Symbol	Description	KEGG_A_class	KEGG_B_class	Pathway	K_ID	GO Component	GO Function	GO Process
NCAM2	5.121398962	2.755954984	3.938676973	-2.37314	1.3057E-08	1.02327E-05	Down	ENSG00000154654	1160	4.220164678	15.934127248	34.96828452	220	0.884575083	3.552564152	7.329589135	175	0.716317171	2.881672395	5.935404082	NCAM2	neural cell adhesion molecule 2 [Source:HGNC Symbol;Acc:HGNC:7657]	Human Diseases;Environmental Information Processing	Signaling molecules and interaction;Neurodegenerative diseases	ko04514//Cell adhesion molecules (CAMs);ko05020//Prion diseases	K06491	GO:0043229//intracellular organelle;GO:0005886//plasma membrane;GO:0044446//intracellular organelle part;GO:0043227//membrane-bounded organelle;GO:0005575//cellular_component;GO:0043226//organelle;GO:0071944//cell periphery;GO:0044422//organelle part;GO:0044424//intracellular part;GO:0070013//intracellular organelle lumen;GO:0016020//membrane;GO:0044464//cell part;GO:0043231//intracellular membrane-bounded organelle;GO:0044428//nuclear part;GO:0031974//membrane-enclosed lumen;GO:0031981//nuclear lumen;GO:0005622//intracellular;GO:0005654//nucleoplasm;GO:0005634//nucleus;GO:0043233//organelle lumen;GO:0005623//cell	GO:0003674//molecular_function	GO:0003008//system process;GO:0044767//single-organism developmental process;GO:0030154//cell differentiation;GO:0048869//cellular developmental process;GO:0044699//single-organism process;GO:0032501//multicellular organismal process;GO:0022610//biological adhesion;GO:0008150//biological_process;GO:0009987//cellular process;GO:0032502//developmental process;GO:0007155//cell adhesion;GO:0044763//single-organism cellular process;GO:0050877//neurological system process;GO:0048856//anatomical structure development

id	CON_Mean	KD_Mean	AllMean	log2FoldChange	pvalue	qvalue	State	gene_id	XF_1_count	XF_1_FPKM	XF_1_TPM	XF_1_CPM	XF_2_count	XF_2_FPKM	XF_2_TPM	XF_2_CPM	XF_3_count	XF_3_FPKM	XF_3_TPM	XF_3_CPM	Symbol	Description	KEGG_A_class	KEGG_B_class	Pathway	K_ID	GO Component	GO Function	GO Process
IL1A	3.823923226	0.890529946	2.357226586	-2.99263	1.31942E-08	1.02327E-05	Down	ENSG00000115008	470	7.024384851	26.5220555 62	14.1681842 45	57	0.941512103	3.781230346	1.899029912	47	0.790322238	3.179387381	1.594079953	IL1A	interleukin 1 alpha [Source:HGNC Symbol;Acc:HGNC:5991]	Organismal Systems;Environmental Information Processing;Human Diseases;Cellular Processes	Immune system;Infectious diseases;Signal transduction;Development;Signaling molecules and interaction;Immune diseases;Neurodegenerative diseases;Endocrine and metabolic diseases;Cell gro[illegible]	ko04060//Cytokine-cytokine receptor interaction;ko05332//Graft-versus-host disease;ko04010//MAPK signaling pathway;ko05164//Influenza A;ko05152//Tuberculosis;ko05323//Rheumatoid arthr[illegible]	K04383	GO:0044424//intracellular part;GO:0005575//cellular_component;GO:0005615//extracellular space;GO:0005623//cell;GO:0044464//cell part;GO:0005829//cytosol;GO:0044444//cytoplasmic part;GO:0005622//intracellular;GO:0044421//extracellular region part;GO:0005737//cytoplasm;GO:0005576//extracellular region	GO:0005488//binding;GO:0043167//ion binding;GO:0003674//molecular_function	GO:0000278//mitotic cell cycle;GO:0008104//protein localization;GO:0015031//protein transport;GO:0006807//nitrogen compound metabolic process;GO:0008152//metabolic process;GO:0044700//single organism signaling;GO:0051234//establishment of localization;GO:0044767//single-organism developmental process;GO:0051301//cell division;GO:0009987//cellular process;GO:0065008//regulation of biological quality;GO:0045184//establishment of protein localization;GO:0009653//anatomical structure morphogenesis;GO:0044237//cellular metabolic process;GO:0044763//single-organism cellular process;GO:0048646//anatomical structure formation involved in morphogenesis;GO:0034641//cellular nitrogen compound metabolic process;GO:0007165//signal transduction;GO:0009058//biosynthetic process;GO:0050789//regulation of biological process;GO:0048869//cellular developmental process;GO:0044699//single-organism process;GO:0051179//localization;GO:0050896//response to stimulus;GO:0008219//cell [illegible]

id	CON_Mean	KD_Mean	AllMean	log2FoldChange	pvalue	qvalue	State	gene_id	XF_1_count	XF_1_FPKM	XF_1_TPM	XF_1_CPM	XF_2_count	XF_2_FPKM	XF_2_TPM	XF_2_CPM	XF_3_count	XF_3_FPKM	XF_3_TPM	XF_3_CPM	Symbol	Description	KEGG_A_class	KEGG_B_class	Pathway	K_ID	GO Component	GO Function	GO Process
CIITA	5.861706252	7.451883299	6.656794776	1.592468065	1.72464E-08	1.22656E-05	Up	ENSG00000179583	1940	2.816618088	10.634739268	58.481441352	5021	8.056697676	32.356705388	167.281213847	5298	8.654342773	34.815556106	179.690118997	CIITA	class II major histocompatibility complex transactivator [Source:HGNC Symbol;Acc:HGNC:7067]	Human Diseases;Organismal Systems	Immune diseases;Immune system;Infectious diseases	ko04612//Antigen processing and presentation;ko05164//Influenza A;ko05152//Tuberculosis;ko05145//Toxoplasmosis;ko05340//Primary immunodeficiency	K08060	GO:0031974//membrane-enclosed lumen;GO:0044446//intracellular organelle part;GO:0043227//membrane-bounded organelle;GO:0044422//organelle part;GO:0044464//cell part;GO:0005634//nucleus;GO:0043226//organelle;GO:0044428//nuclear part;GO:0043229//intracellular organelle;GO:0044424//intracellular part;GO:0005654//nucleoplasm;GO:0031981//nuclear lumen;GO:0044444//cytoplasmic part;GO:0005829//cytosol;GO:0005623//cell;GO:0005575//cellular_component;GO:0070013//intracellular organelle lumen;GO:0005622//intracellular;GO:0005737//cytoplasm;GO:0043233//organelle lumen;GO:0043231//intracellular membrane-bounded organelle	GO:0003676//nucleic acid binding;GO:0003677//DNA binding;GO:0016301//kinase activity;GO:0016740//transferase activity;GO:1901363//heterocyclic compound binding;GO:0043167//ion binding;GO:0003824//catalytic activity;GO:0005488//binding;GO:0008134//transcription factor binding;GO:0016772//transferase activity, transferring phosphorus-containing groups;GO:0097159//organic cyclic compound binding;GO:0005515//protein binding;GO:0016746//transferase activity, transferring acyl groups;GO:0003674//molecular_function	GO:0008152//metabolic process;GO:0009987//cellular process;GO:0009058//biosynthetic process;GO:0007154//cell communication;GO:0008150//biological_process;GO:0034641//cellular nitrogen compound metabolic process;GO:0050794//regulation of cellular process;GO:0051716//cellular response to stimulus;GO:0050896//response to stimulus;GO:0023052//signaling;GO:0050789//regulation of biological process;GO:0007165//signal transduction;GO:0065007//biological regulation;GO:0006950//response to stress;GO:0044763//single-organism cellular process;GO:0044699//single-organism process;GO:0006807//nitrogen compound metabolic process;GO:0002376//immune system process;GO:0032502//developmental process;GO:0044237//cellular metabolic process;GO:0044700//single organism signaling;GO:0044767//single-organism developmental process;GO:0007568//aging
FAT3	3.826968455	5.720324224	4.773646339	1.900888629	1.72533E-08	1.22656E-05	Up	ENSG00000165323	471	0.722929191	2.729572566	14.198329318	1516	2.571666619	10.328134 74	50.507532402	1587	2.740612824	11.025211511	53.825635872	FAT3	FAT atypical cadherin 3 [Source:HGNC Symbol;Acc:HGNC:23112]	-	-	-	-	GO:0005575//cellular_component;GO:0044464//cell part;GO:0016020//membrane;GO:0005886//plasma membrane;GO:0005623//cell;GO:0071944//cell periphery	GO:0003674//molecular_function;GO:0043167//ion binding;GO:0005488//binding	GO:0022610//biological adhesion;GO:0032502//developmental process;GO:0008150//biological_process;GO:0048856//anatomical structure development;GO:0007155//cell adhesion
SNRPGP19	-0.05105	-3.35941	-1.70523	-7.74653	1.8246E-08	1.24526E-05	Down	ENSG00000270526	29	4.62543446	17.464309311	0.874207113	0	0	0	0	0	0	0	0	-	-	-	-	-	-	-	-	-

id	CON_Mean	KD_Mean	AllMean	log2FoldChange	pvalue	qvalue	State	gene_id	XF_1_count	XF_1_FPKM	XF_1_TPM	XF_1_CPM	XF_2_count	XF_2_FPKM	XF_2_TPM	XF_2_CPM	XF_3_count	XF_3_FPKM	XF_3_TPM	XF_3_CPM	Symbol	Description	KEGG_A_class	KEGG_B_class	Pathway	K_ID	GO Component	GO Function	GO Process
QPRT	3.585825452	0.6536755577	2.119750515	-3.00913	2.04842E-08	1.34423E-05	Down	ENSG00000103485	398	3.260255163	12.3097851 84	11.997738999	44	0.398347236	1.599812313	1.465917827	43	0.396308037	1.594307626	1.458413574	QPRT	quinolinate phosphoribosyltransferase [Source:HGNC Symbol;Acc:HGNC:9755]	Metabolism	Metabolism of cofactors and vitamins	ko00760//Nicotinate and nicotinamide metabolism	K00767	GO:0005576//extracellular region;GO:0044464//cell part;GO:0005575//cellular_component;GO:0005737//cytoplasm;GO:0005622//intracellular;GO:0032991//macromolecular complex;GO:0005829//cytosol;GO:0044424//intracellular part;GO:0044444//cytoplasmic part;GO:0005615//extracellular space;GO:0043226//organelle;GO:0044421//extracellular region part;GO:0005623//cell	GO:0003824//catalytic activity;GO:0016757//transferase activity, transferring glycosyl groups;GO:0003674//molecular_function;GO:0016740//transferase activity	GO:0044710//single-organism metabolic process;GO:0008150//biological_process;GO:0044699//single-organism process;GO:0009058//biosynthetic process;GO:0034641//cellular nitrogen compound metabolic process;GO:0009987//cellular process;GO:0009056//catabolic process;GO:0008152//metabolic process;GO:0051186//cofactor metabolic process;GO:0044237//cellular metabolic process;GO:0006807//nitrogen compound metabolic process;GO:0044281//small molecule metabolic process

id	CON_Mean	KD_Mean	AllMean	log2FoldChange	pvalue	qvalue	State	gene_id	XF_1_count	XF_1_FPKM	XF_1_TPM	XF_1_CPM	XF_2_count	XF_2_FPKM	XF_2_TPM	XF_2_CPM	XF_3_count	XF_3_FPKM	XF_3_TPM	XF_3_CPM	Symbol	Description	KEGG_A_class	KEGG_B_class	Pathway	K_ID	GO Component	GO Function	GO Process
THBS1	8.622003464	10.802658389	9.712330927	2.259792961	2.89292E-08	1.82811E-05	Up	ENSG00000137801	13163	43.328193282	163.594787864	396.799594079	73887	268.796954679	1079.522184106	2461.642510951	37509	138.914342931	558.838519227	1272.17755256	THBS1	thrombospondin 1 [Source:HGNC Symbol;Acc:HGNC:11785]	Human Diseases;Environmental Information Processing;Cellular Processes	Cell growth and death;Signaling molecules and interaction;Cancers;Cellular community;Infectious diseases;Signal transduction;Transport and catabolism	ko04151//PI3K-Akt signaling pathway;ko04145//Phagosome;ko04510//Focal adhesion;ko04015//Rap1 signaling pathway;ko05205//Proteoglycans in cancer;ko05206//MicroRNAs in canc[illegible]	K16857	GO:0071944//cell periphery;GO:0031982//vesicle;GO:0005623//cell;GO:0005737//cytoplasm;GO:0043226//organelle;GO:0005886//plasma membrane;GO:0043227//membrane-bounded organelle;GO:0005615//extracellular space;GO:0005576//extracellular region;GO:0005575//cellular_component;GO:0097708//intracellular vesicle;GO:0005622//intracellular;GO:0032991//macromolecular complex;GO:0043229//intracellular organelle;GO:0005783//endoplasmic reticulum;GO:0044444//cytoplasmic part;GO:0044464//cell part;GO:0016020//membrane;GO:0044421//extracellular region part;GO:0043231//intracellular membrane-bounded organelle;GO:0012505//endomembrane system;GO:0044424//intracellular part;GO:0031012//extracellular matrix;GO:0031410//cytoplasmic vesicle	GO:0005488//binding;GO:0043167//ion binding;GO:0003674//molecular_function;GO:0008289//lipid binding;GO:0005198//structural molecule activity	GO:0044271//cellular nitrogen compound biosynthetic process;GO:0034641//cellular nitrogen compound metabolic process;GO:0044763//single-organism cellular process;GO:0022610//biological adhesion;GO:0044237//cellular metabolic process;GO:0008219//cell death;GO:0043043//peptide biosynthetic process;GO:0008152//metabolic process;GO:0016192//vesicle-mediated transport;GO:0030154//cell differentiation;GO:0048646//anatomical structure formation involved in morphogenesis;GO:1901576//organic substance biosynthetic process;GO:0071840//cellular component organization or biogenesis;GO:0065007//biological regulation;GO:0007165//signal transduction;GO:0036211//protein modification process;GO:0007049//cell cycle;GO:0050789//regulation of biological process;GO:0006950//response to stress;GO:0009987//cellular process;GO:0009059//macromolecule biosynthetic process;GO:0044700//single organism signaling;GO:0002376//immune system [illegible]
MSTRG.16781	2.079615334	-1.04397	0.517821152	-3.38977	5.39428E-08	3.28704E-05	Down	MSTRG.16781	138	2.690827979	10.15979202	4.160020055	10	0.215500092	0.865475319	0.333163142	13	0.285197757	1.147322074	0.440915732	-	-	-	-	-	-	-	-	-
ENSG00000285576	1.351177167	-2.46604	-0.55743	-4.32046	6.84493E-08	4.02718E-05	Down	ENSG00000285576	82	1.955613904	7.383835271	2.471895975	0	0	0	0	7	0.187829243	0.755618274	0.237416163	-	-	-	-	-	-	-	-	-
GABPB1-AS1	6.549971715	5.036078197	5.793024956	-1.50711	7.45811E-08	4.24168E-05	Down	ENSG00000244879	3128	5.488898534	20.724501144	94.293787911	864	1.675609495	6.729457273	28.785295512	1076	2.124352754	8.546058831	36.494255953	-	-	-	-	-	-	-	-	-

id	CON_Mean	KD_Mean	AllMean	log2FoldChange	pvalue	qvalue	State	gene_id	XF_1_count	XF_1_FPKM	XF_1_TPM	XF_1_CPM	XF_2_count	XF_2_FPKM	XF_2_TPM	XF_2_CPM	XF_3_count	XF_3_FPKM	XF_3_TPM	XF_3_CPM	Symbol	Description	KEGG_A_class	KEGG_B_class	Pathway	K_ID	GO Component	GO Function	GO Process
FBXO2	3.16749569	-0.22125	1.473123	-3.40965	8.15492E-08	4.48836E-05	Down	ENSG00000116661	297	2.830568018	10.68741019	8.95308664	14	0.147463926	0.59223357	0.466428399	35	0.375302187	1.509803191	1.187080816	FBXO2	F-box protein 2 [Source:HGNC Symbol;Acc:HGNC:13581]	Genetic Information Processing	Folding, sorting and degradation	ko04141//Protein processing in endoplasmic reticulum;ko04120//Ubiquitin mediated proteolysis	K10099	GO:0005737//cytoplasm;GO:0043226//organelle;GO:0005575//cellular_component;GO:0032991//macromolecular complex;GO:0043229//intracellular organelle;GO:0005783//endoplasmic reticulum;GO:0044464//cell part;GO:0012505//endomembrane system;GO:0043231//intracellular membrane-bounded organelle;GO:0005829//cytosol;GO:0044444//cytoplasmic part;GO:0043227//membrane-bounded organelle;GO:0044424//intracellular part;GO:0005623//cell;GO:0005622//intracellular	GO:0003674//molecular_function	GO:0043170//macromolecule metabolic process;GO:0019538//protein metabolic process;GO:0044260//cellular macromolecule metabolic process;GO:0008152//metabolic process;GO:0006950//response to stress;GO:0009056//catabolic process;GO:0008283//cell proliferation;GO:0044237//cellular metabolic process;GO:0006464//cellular protein modification process;GO:0008150//biological_process;GO:0036211//protein modification process;GO:0044238//primary metabolic process;GO:0044267//cellular protein metabolic process;GO:0044699//single-organism process;GO:0071704//organic substance metabolic process;GO:0050896//response to stimulus;GO:0009987//cellular process;GO:0043412//macromolecule modification

id	CON_Mean	KD_Mean	AllMean	log2FoldChange	pvalue	qvalue	State	gene_id	XF_1_count	XF_1_FPKM	XF_1_TPM	XF_1_CPM	XF_2_count	XF_2_FPKM	XF_2_TPM	XF_2_CPM	XF_3_count	XF_3_FPKM	XF_3_TPM	XF_3_CPM	Symbol	Description	KEGG_A_class	KEGG_B_class	Pathway	K_ID	GO Component	GO Function	GO Process
EME2	3.571371501	5.466011386	4.518691444	1.904338003	8.98965E-08	4.79317E-05	Up	ENSG000001977774	394	1.625671873	6.138069121	11.877158708	1365	6.224578285	24.998684801	45.476768951	1238	5.747159089	23.120246673	41.988744303	EME2	essential meiotic structure-specific endonuclease subunit 2 [Source:HGNC Symbol;Acc:HGNC:27289]	Genetic Information Processing	Replication and repair	ko03460//Fanconi anemia pathway	K10883	GO:0005694//chromosome;GO:0032991//macromolecular complex;GO:0043233//organelle lumen;GO:0031981//nuclear lumen;GO:0044424//intracellular part;GO:0044422//organelle part;GO:0043226//organelle;GO:0044428//nuclear part;GO:0044446//intracellular organelle part;GO:0043231//intracellular membrane-bounded organelle;GO:0005575//cellular_component;GO:0070013//intracellular organelle lumen;GO:0043227//membrane-bounded organelle;GO:0005634//nucleus;GO:0043232//intracellular non-membrane-bounded organelle;GO:0044464//cell part;GO:0043228//non-membrane-bounded organelle;GO:0000228//nuclear chromosome;GO:0005622//intracellular;GO:0031974//membrane-enclosed lumen;GO:0043229//intracellular organelle;GO:0005623//cell	GO:0005488//binding;GO:0003676//nucleic acid binding;GO:0004518//nuclease activity;GO:0003674//molecular_function;GO:0016788//hydrolase activity, acting on ester bonds;GO:0003824//catalytic activity;GO:0016787//hydrolase activity;GO:1901363//heterocyclic compound binding;GO:0097159//organic cyclic compound binding;GO:0003677//DNA binding	GO:0009987//cellular process;GO:0050896//response to stimulus;GO:0000003//reproduction;GO:0000278//mitotic cell cycle;GO:0008150//biological_process;GO:0044699//single-organism process;GO:0007059//chromosome segregation;GO:0090304//nucleic acid metabolic process;GO:0034641//cellular nitrogen compound metabolic process;GO:0006807//nitrogen compound metabolic process;GO:0044237//cellular metabolic process;GO:0006139//nucleobase-containing compound metabolic process;GO:0044238//primary metabolic process;GO:0071704//organic substance metabolic process;GO:0006725//cellular aromatic compound metabolic process;GO:0008152//metabolic process;GO:0006950//response to stress;GO:0006259//DNA metabolic process;GO:0046483//heterocycle metabolic process;GO:0043170//macromolecule metabolic process;GO:0044763//single-organism cellular process;GO:0009058//biosynthetic process;GO:0007049//cell cycle;GO:1901360//organic cyclic compound metabolic process;GO:0044260//cellu [illegible]
LINC01203	4.664100236	6.231775104	5.44793767	1.572266541	1.16553E-07	6.02613E-05	Up	ENSG000002269855	844	3.617065894	13.656999813	25.442441495	2140	10.136041014	40.707608265	71.296912494	2287	11.027474012	44.36242592	77.567252198	-	-	-	-	-	-	-	-	-
CCDC102B	5.817990725	4.152963092	4.985476908	-1.66655	1.4837E-07	7.44554E-05	Down	ENSG000001506366	1882	9.773131288	36.900531 01	56.733027126	563	3.231194646	12.976881774	18.757084922	483	2.822000907	11.352627633	16.381715265	CCDC102B	coiled-coil domain containing 102B [Source:HGNC Symbol;Acc:HGNC:26295]	-	-	-	-	-	GO:0003674//molecular_function	-

id	CON_Mean	KD_Mean	AllMean	log2FoldChange	pvalue	qvalue	State	gene_id	XF_1_count	XF_1_FPKM	XF_1_TPM	XF_1_CPM	XF_2_count	XF_2_FPKM	XF_2_TPM	XF_2_CPM	XF_3_count	XF_3_FPKM	XF_3_TPM	XF_3_CPM	Symbol	Description	KEGG_A_class	KEGG_B_class	Pathway	K_ID	GO Component	GO Function	GO Process
TMEM201	4.861153871	6.451503288	5.65632858	1.606251693	1.75913E-07	8.57548E-05	Up	ENSG00000188807	968	4.179978589	15.78239614 3	29.18043053	2960	14.12638449 8	56.73332670 3	98.61629017 8	2243	10.89742472 8	43.83925065	76.07492202 9	TMEM201	transmembrane protein 201 [Source:HGNC Symbol;Acc:HGNC:33719]	-	-	-	-	GO:0044424//intracellular part;GO:0005856//cytoskeleton;GO:0005634//nucleus;GO:0044428//nuclear part;GO:0043231//intracellular membrane-bounded organelle;GO:0044464//cell part;GO:0031967//organelle envelope;GO:0044422//organelle part;GO:0012505//endomembrane system;GO:0005622//intracellular;GO:0031975//envelope;GO:0043227//membrane-bounded organelle;GO:0043226//organelle;GO:0043228//non-membrane-bounded organelle;GO:0043232//intracellular non-membrane-bounded organelle;GO:0005623//cell;GO:0005737//cytoplasm;GO:0043229//intracellular organelle;GO:0044446//intracellular organelle part;GO:0005575//cellular_component;GO:0005635//nuclear envelope	GO:0005488//binding;GO:0008092//cytoskeletal protein binding;GO:0003674//molecular_function;GO:0005515//protein binding	GO:0030705//cytoskeleton-dependent intracellular transport;GO:0051641//cellular localization;GO:0009987//cellular process;GO:0051649//establishment of localization in cell;GO:0006928//movement of cell or subcellular component;GO:0008150//biological_process;GO:0044763//single-organism cellular process;GO:0046907//intracellular transport;GO:0006810//transport;GO:0051179//localization;GO:0040011//locomotion;GO:0051674//localization of cell;GO:0051234//establishment of localization;GO:0044699//single-organism process;GO:0048870//cell motility

id	CON_Mean	KD_Mean	AllMean	log2FoldChange	pvalue	qvalue	State	gene_id	XF_1_count	XF_1_FPKM	XF_1_TPM	XF_1_CPM	XF_2_count	XF_2_FPKM	XF_2_TPM	XF_2_CPM	XF_3_count	XF_3_FPKM	XF_3_TPM	XF_3_CPM	Symbol	Description	KEGG_A_class	KEGG_B_class	Pathway	K_ID	GO Component	GO Function	GO Process
SEMA3A	7.042783555	5.711165305	6.37697443	-1.33103	2.14218E-07	0.000101527	Down	ENSG00000075213	4403	14.335107011	54.125238839	132.72875581	1636	5.886757761	23.641955332	54.505490112	1452	5.318813649	21.397055777	49.246895558	SEMA3A	semaphorin 3A [Source:HGNC Symbol;Acc:HGNC:10723]	Organismal Systems	Development	ko04360//Axon guidance	K06840	GO:0071944//cell periphery;GO:0044421//extracellular region part;GO:0005886//plasma membrane;GO:0005615//extracellular space;GO:0044464//cell part;GO:0016020//membrane;GO:0005623//cell;GO:0005575//cellular_component;GO:0005576//extracellular region	GO:0003674//molecular_function	GO:0051674//localization of cell;GO:0009653//anatomical structure morphogenesis;GO:0071704//organic substance metabolic process;GO:0051716//cellular response to stimulus;GO:0048870//cell motility;GO:0040007//growth;GO:0023052//signaling;GO:0000902//cell morphogenesis;GO:0030154//cell differentiation;GO:0044707//single-multicellular organism process;GO:0044237//cellular metabolic process;GO:0000003//reproduction;GO:0008150//biological_process;GO:0003013//circulatory system process;GO:0044267//cellular protein metabolic process;GO:0065007//biological regulation;GO:0006928//movement of cell or subcellular component;GO:0007165//signal transduction;GO:0006950//response to stress;GO:0048856//anatomical structure development;GO:0032501//multicellular organismal process;GO:0040011//locomotion;GO:0044763//single-organism cellular process;GO:0003008//system process;GO:0032502//developmental process;GO:0051179//localization;GO:0048869//cellul [illegible]
PCNX3	6.5508929	7.99540947	7.273151185	1.447802	2.23687E-07	0.00010315	Up	ENSG00000197136	3130	11.840140301	44.704962152	94.354078057	8005	33.466820877	134.406937799	266.697095567	7062	30.056342344	120.913661574	239.51899214	PCNX3	pecanex 3 [Source:HGNC Symbol;Acc:HGNC:18760]	-	-	-	-	GO:0005575//cellular_component	-	-

id	CON_Mean	KD_Mean	AllMean	log2FoldChange	pvalue	qvalue	State	gene_id	XF_1_count	XF_1_FPKM	XF_1_TPM	XF_1_CPM	XF_2_count	XF_2_FPKM	XF_2_TPM	XF_2_CPM	XF_3_count	XF_3_FPKM	XF_3_TPM	XF_3_CPM	Symbol	Description	KEGG_A_class	KEGG_B_class	Pathway	K_ID	GO Component	GO Function	GO Process
VGF	7.444560693	6.131207002	6.787883848	-1.31191	2.44864E-07	0.000109944	Down	ENSG00000128564	5818	65.441803698	247.089416436	175.384033909	2202	27.374076111	109.937712897	73.362523977	1933	24.462976737	98.41211071	65.560777656	VGF	VGF nerve growth factor inducible [Source:HGNC Symbol;Acc:HGNC:12684]	-	-	-	-	GO:0005783//endoplasmic reticulum;GO:0005623//cell;GO:0097708//intracellular vesicle;GO:0005794//Golgi apparatus;GO:0043227//membrane-bounded organelle;GO:0043226//organelle;GO:0031982//vesicle;GO:0044421//extracellular region part;GO:0044464//cell part;GO:0012505//endomembrane system;GO:0031410//cytoplasmic vesicle;GO:0005576//extracellular region;GO:0005615//extracellular space;GO:0044424//intracellular part;GO:0043231//intracellular membrane-bounded organelle;GO:0005737//cytoplasm;GO:0043229//intracellular organelle;GO:0005575//cellular_component;GO:0044444//cytoplasmic part;GO:0005622//intracellular	GO:0003674//molecular_function	GO:0000003//reproduction;GO:0043412//macromolecule modification;GO:0048856//anatomical structure development;GO:0042592//homeostatic process;GO:0051179//localization;GO:0033036//macromolecule localization;GO:0043170//macromolecule metabolic process;GO:0071702//organic substance transport;GO:0044699//single-organism process;GO:0065007//biological regulation;GO:0065008//regulation of biological quality;GO:0044267//cellular protein metabolic process;GO:0007267//cell-cell signaling;GO:0044237//cellular metabolic process;GO:0023052//signaling;GO:0008104//protein localization;GO:0019538//protein metabolic process;GO:0008152//metabolic process;GO:0051234//establishment of localization;GO:0050896//response to stimulus;GO:0044700//single organism signaling;GO:0006950//response to stress;GO:0006810//transport;GO:0050789//regulation of biological process;GO:0036211//protein modification process;GO:0015031//protein transport;GO:0009987//cell [illegible]

id	CON_Mean	KD_Mean	AllMean	log2FoldChange	pvalue	qvalue	State	gene_id	XF_1_count	XF_1_FPKM	XF_1_TPM	XF_1_CPM	XF_2_count	XF_2_FPKM	XF_2_TPM	XF_2_CPM	XF_3_count	XF_3_FPKM	XF_3_TPM	XF_3_CPM	Symbol	Description	KEGG_A_class	KEGG_B_class	Pathway	K_ID	GO Component	GO Function	GO Process
NECTIN1	4.806663587	6.292891878	5.549777733	1.490487237	3.19815E-07	0.000136948	Up	ENSG00000110400	932	3.594116401	13.570349134	28.095207907	2415	10.292810402	41.337213731	80.458898912	2206	9.571447873	38.504978272	74.820008023	NECTIN1	nectin cell adhesion molecule 1 [Source:HGNC Symbol;Acc:HGNC:9706]	Cellular Processes;Human Diseases;Environmental Information Processing	Signaling molecules and interaction;Cellular community;Infectious diseases	ko05168//Herpes simplex infection;ko04514//Cell adhesion molecules (CAMs);ko04520//Adherens junction	K06081	GO:0044464//cell part;GO:0005886//plasma membrane;GO:0005622//intracellular;GO:0071944//cell periphery;GO:0016020//membrane;GO:0005623//cell;GO:0043226//organelle;GO:0005576//extracellular region;GO:0005575//cellular_component	GO:0003674//molecular_function	GO:0051179//localization;GO:0044699//single-organism process;GO:0006810//transport;GO:0044419//interspecies interaction between organisms;GO:0044085//cellular component biogenesis;GO:0009653//anatomical structure morphogenesis;GO:0007155//cell adhesion;GO:0044403//symbiosis, encompassing mutualism through parasitism;GO:0048869//cellular developmental process;GO:0051704//multi-organism process;GO:0030154//cell differentiation;GO:0032989//cellular component morphogenesis;GO:0008150//biological_process;GO:0000902//cell morphogenesis;GO:0022610//biological adhesion;GO:0016043//cellular component organization;GO:0048646//anatomical structure formation involved in morphogenesis;GO:0032502//developmental process;GO:0044767//single-organism developmental process;GO:0040011//locomotion;GO:0034330//cell junction organization;GO:0044763//single-organism cellular process;GO:0048856//anatomical structure development;GO:0071840//cellular component organization or biogenesis;GO:0022607//cellular component [illegible]

id	CON_Mean	KD_Mean	AllMean	log2FoldChange	pvalue	qvalue	State	gene_id	XF_1_count	XF_1_FPKM	XF_1_TPM	XF_1_CPM	XF_2_count	XF_2_FPKM	XF_2_TPM	XF_2_CPM	XF_3_count	XF_3_FPKM	XF_3_TPM	XF_3_CPM	Symbol	Description	KEGG_A_class	KEGG_B_class	Pathway	K_ID	GO Component	GO Function	GO Process
PLAC8	3.578616578	0.920532552	2.249574565	-2.71788	3.21061E-07	0.000136948	Down	ENSG00000145287	396	3.152217812	11.9018672 41	11.9374488 53	56	0.492662688	1.978594963	1.865713598	50	0.447802941	1.801466481	1.695829738	PLAC8	placenta associated 8 [Source:HGNC Symbol;Acc:HGNC:19254]	-	-	-	-	GO:0043226//organelle;GO:0005622//intracellular;GO:0005764//lysosome;GO:0043227//membrane-bounded organelle;GO:0044424//intracellular part;GO:0005737//cytoplasm;GO:0005773//vacuole;GO:0000323//lytic vacuole;GO:0005575//cellular_component;GO:0044444//cytoplasmic part;GO:0043229//intracellular organelle;GO:0097708//intracellular vesicle;GO:0005623//cell;GO:0031410//cytoplasmic vesicle;GO:0031982//vesicle;GO:0044464//cell part;GO:0005576//extracellular region;GO:0043231//intracellular membrane-bounded organelle	GO:0003674//molecular_function	GO:0042592//homeostatic process;GO:0009058//biosynthetic process;GO:0048869//cellular developmental process;GO:0040007//growth;GO:0051179//localization;GO:0008283//cell proliferation;GO:0008219//cell death;GO:0008152//metabolic process;GO:0044237//cellular metabolic process;GO:0006810//transport;GO:0051234//establishment of localization;GO:0032502//developmental process;GO:0006807//nitrogen compound metabolic process;GO:0016192//vesicle-mediated transport;GO:0065007//biological regulation;GO:0034641//cellular nitrogen compound metabolic process;GO:0009987//cellular process;GO:0008150//biological_process;GO:0065008//regulation of biological quality;GO:0050896//response to stimulus;GO:0006950//response to stress;GO:0030154//cell differentiation;GO:0044699//single-organism process;GO:0044767//single-organism developmental process;GO:0044763//single-organism cellular process;GO:0002376//immune system process
ENSG00000235592	2.423324366	4.71847057	3.570897468	2.316329918	3.48377E-07	0.000144976	Up	ENSG00000235592	176	7.179340763	27.1071244 87	5.305532824	749	33.7671439 42	135.613072784	24.9539193 73	798	36.6244149 02	147.336361094	27.0654426 12	-	-	-	-	-	-	-	-	-

id	CON_Mean	KD_Mean	AllMean	log2FoldChange	pvalue	qvalue	State	gene_id	XF_1_count	XF_1_FPKM	XF_1_TPM	XF_1_CPM	XF_2_count	XF_2_FPKM	XF_2_TPM	XF_2_CPM	XF_3_count	XF_3_FPKM	XF_3_TPM	XF_3_CPM	Symbol	Description	KEGG_A_class	KEGG_B_class	Pathway	K_ID	GO Component	GO Function	GO Process
ROBO4	3.148146995	5.282427938	4.215287466	2.192808816	3.8394E-07	0.000155971	Up	ENSG00000154133	293	1.33421546	5.037613588	8.832506348	1486	7.478556341	30.034817487	49.508042975	881	4.513673712	18.158058272	29.880519977	ROBO4	roundabout guidance receptor 4 [Source:HGNC Symbol;Acc:HGNC:17985]	-	-	-	-	GO:0005576//extracellular region;GO:0071944//cell periphery;GO:0005623//cell;GO:0043226//organelle;GO:0044464//cell part;GO:0005615//extracellular space;GO:0005575//cellular_component;GO:0005886//plasma membrane;GO:0044421//extracellular region part;GO:0016020//membrane	GO:0003674//molecular_function	GO:0016043//cellular component organization;GO:0006928//movement of cell or subcellular component;GO:0032502//developmental process;GO:0051674//localization of cell;GO:0030154//cell differentiation;GO:0048869//cellular developmental process;GO:0032989//cellular component morphogenesis;GO:0009987//cellular process;GO:0048646//anatomical structure formation involved in morphogenesis;GO:0048870//cell motility;GO:0040011//locomotion;GO:0009653//anatomical structure morphogenesis;GO:0008150//biological_process;GO:0000902//cell morphogenesis;GO:0022610//biological adhesion;GO:0007155//cell adhesion;GO:0044767//single-organism developmental process;GO:0071840//cellular component organization or biogenesis;GO:0051179//localization;GO:0044763//single-organism cellular process;GO:0044699//single-organism process;GO:0048856//anatomical structure development
CAPN15	5.679128708	7.161229121	6.420178914	1.491842	4.12996E-07	0.000161213	Up	ENSG00000103326	1709	6.446187377	24.338948307	51.517929521	4711	19.638783337	78.871809788	156.953156429	3773	16.011925926	64.414377851	127.967312	CAPN15	calpain 15 [Source:HGNC Symbol;Acc:HGNC:11182]	-	-	-	-	GO:0005737//cytoplasm;GO:0005623//cell;GO:0044424//intracellular part;GO:0005622//intracellular;GO:0044464//cell part;GO:0005575//cellular_component	GO:0003824//catalytic activity;GO:0016787//hydrolase activity;GO:0003674//molecular_function;GO:0043167//ion binding;GO:0005488//binding;GO:0008233//peptidase activity	GO:0008150//biological_process

id	CON_Mean	KD_Mean	AllMean	log2FoldChange	pvalue	qvalue	State	gene_id	XF_1_count	XF_1_FPKM	XF_1_TPM	XF_1_CPM	XF_2_count	XF_2_FPKM	XF_2_TPM	XF_2_CPM	XF_3_count	XF_3_FPKM	XF_3_TPM	XF_3_CPM	Symbol	Description	KEGG_A_class	KEGG_B_class	Pathway	K_ID	GO Component	GO Function	GO Process
S100A4	8.087551144	6.724986119	7.406268632	-1.34948	4.22146E-07	0.000161213	Down	ENSG00000196154	9087	289.564774941	1093.313252663	273.928277094	3598	126.71469204	508.902049402	119.87209867	2697	96.694562417	388.992561448	91.473056047	S100A4	S100 calcium binding protein A4 [Source:HGNC Symbol;Acc:HGNC:10494]	-	-	-	-	GO:0005634//nucleus;GO:0031012//extracellular matrix;GO:0005623//cell;GO:0043226//organelle;GO:0044421//extracellular region part;GO:0005575//cellular_component;GO:0005615//extracellular space;GO:0005622//intracellular;GO:0043227//membrane-bounded organelle;GO:0005737//cytoplasm;GO:0005576//extracellular region;GO:0044464//cell part;GO:0043229//intracellular organelle;GO:0044424//intracellular part;GO:0043231//intracellular membrane-bounded organelle	GO:0003674//molecular_function;GO:0043167//ion binding;GO:0003723//RNA binding;GO:1901363//heterocyclic compound binding;GO:0005515//protein binding;GO:0005488//binding;GO:0097159//organic cyclic compound binding;GO:0003676//nucleic acid binding;GO:0008092//cytoskeletal protein binding	GO:0044763//single-organism cellular process;GO:0007165//signal transduction;GO:0050794//regulation of cellular process;GO:0009987//cellular process;GO:0008150//biological_process;GO:0050789//regulation of biological process;GO:0023052//signaling;GO:0044700//single organism signaling;GO:0048869//cellular developmental process;GO:0044767//single-organism developmental process;GO:0048856//anatomical structure development;GO:0007154//cell communication;GO:0032502//developmental process;GO:0065007//biological regulation;GO:0044699//single-organism process;GO:0050896//response to stimulus;GO:0030154//cell differentiation;GO:0051716//cellular response to stimulus
ENSG00000230074	1.030332113	-2.02924	-0.49946	-3.63567	4.25189E-07	0.000161213	Down	ENSG00000230074	65	1.389666479	5.246980677	1.959429736	3	0.070885775	0.284686137	0.099948943	6	0.144325935	0.580608814	0.203499569	-	-	-	-	-	-	-	-	-

id	CON_Mean	KD_Mean	AllMean	log2FoldChange	pvalue	qvalue	State	gene_id	XF_1_count	XF_1_FPKM	XF_1_TPM	XF_1_CPM	XF_2_count	XF_2_FPKM	XF_2_TPM	XF_2_CPM	XF_3_count	XF_3_FPKM	XF_3_TPM	XF_3_CPM	Symbol	Description	KEGG_A_class	KEGG_B_class	Pathway	K_ID	GO Component	GO Function	GO Process
PCSK1	4.433812603	1.927662756	3.180737679	-2.5079	4.36135E-07	0.000161768	Down	ENSG00000175426	719	4.045978605	15.276450769	21.674307388	134	0.833374297	3.346935394	4.464386109	89	0.56348272	2.266834672	3.018576933	PCSK1	proprotein convertase subtilisin/kexin type 1 [Source:HGNC Symbol;Acc:HGNC:8743]	-	-	-	-	GO:0044421//extracellular region part;GO:0044444//cytoplasmic part;GO:0044464//cell part;GO:0005576//extracellular region;GO:0044424//intracellular part;GO:0005737//cytoplasm;GO:0031410//cytoplasmic vesicle;GO:0005575//cellular_component;GO:0097708//intracellular vesicle;GO:0005623//cell;GO:0005615//extracellular space;GO:0043227//membrane-bounded organelle;GO:0043226//organelle;GO:0005622//intracellular;GO:0031982//vesicle	GO:0016787//hydrolase activity;GO:0003824//catalytic activity;GO:0003674//molecular_function;GO:0008233//peptidase activity	GO:0044699//single-organism process;GO:0043170//macromolecule metabolic process;GO:0019538//protein metabolic process;GO:0009058//biosynthetic process;GO:0044238//primary metabolic process;GO:0071704//organic substance metabolic process;GO:0044237//cellular metabolic process;GO:0023052//signaling;GO:0051604//protein maturation;GO:0008150//biological_process;GO:0044700//single organism signaling;GO:0044763//single-organism cellular process;GO:0010467//gene expression;GO:0006807//nitrogen compound metabolic process;GO:0007154//cell communication;GO:0034641//cellular nitrogen compound metabolic process;GO:0007267//cell-cell signaling;GO:0009987//cellular process;GO:0008152//metabolic process

id	CON_Mean	KD_Mean	AllMean	log2FoldChange	pvalue	qvalue	State	gene_id	XF_1_count	XF_1_FPKM	XF_1_TPM	XF_1_CPM	XF_2_count	XF_2_FPKM	XF_2_TPM	XF_2_CPM	XF_3_count	XF_3_FPKM	XF_3_TPM	XF_3_CPM	Symbol	Description	KEGG_A_class	KEGG_B_class	Pathway	K_ID	GO Component	GO Function	GO Process
POLR2A	7.289008027	8.711372594	8.00019031	1.42720066	6.28055E-07	0.0002279 97	Up	ENSG00000181222	5223	18.84472957	71.1522752 93	157.447715557	13414	53.4895319 38	214.820649339	446.905039342	11374	46.1720345 56	185.745480822	385.767348711	POLR2A	RNA polymerase II subunit A [Source:HGNC Symbol;Acc:HGNC:9187]	Genetic Information Processing; Metabolism; Human Diseases	Infectious diseases;Transcription;Neurodegenerative diseases;Nucleotide metabolism	ko05168//Herpes simplex infection; ko05169//Epstein-Barr virus infection; ko05016//Huntington's disease; ko00230//Purine metabolism; ko00240//Pyrimidine metabolism; ko03020//RNA polymer	K03006	GO:0005634//nucleus;GO:0031974//membrane-enclosed lumen;GO:0044424//intracellular part;GO:0043226//organelle;GO:0044446//intracellular organelle part;GO:0043231//intracellular membrane-bounded organelle;GO:0031981//nuclear lumen;GO:0070013//intracellular organelle lumen;GO:0043229//intracellular organelle;GO:0044428//nuclear part;GO:0005575//cellular_component;GO:0005623//cell;GO:0044464//cell part;GO:0043227//membrane-bounded organelle;GO:0005737//cytoplasm;GO:0032991//macromolecular complex;GO:0005622//intracellular;GO:0005654//nucleoplasm;GO:0044422//organelle part;GO:0043233//organelle lumen	GO:0016772//transferase activity, transferring phosphorus-containing groups;GO:0003723//RNA binding;GO:0016779//nucleotidyltransferase activity;GO:1901363//heterocyclic compound binding;GO:0003824//catalytic activity;GO:0097159//organic cyclic compound binding;GO:0003674//molecular_function;GO:0019899//enzyme binding;GO:0016740//transferase activity;GO:0005488//binding;GO:0003676//nucleic acid binding;GO:0003677//DNA binding;GO:0005515//protein binding;GO:0043167//ion binding	GO:0046483//heterocycle metabolic process;GO:0050789//regulation of biological process;GO:0006950//response to stress;GO:0010467//gene expression;GO:0044419//interspecies interaction between organisms;GO:0006396//RNA processing;GO:0007154//cell communication;GO:0044699//single-organism process;GO:0043170//macromolecule metabolic process;GO:0051716//cellular response to stimulus;GO:0050896//response to stimulus;GO:0009987//cellular process;GO:1901360//organic cyclic compound metabolic process;GO:0016071//mRNA metabolic process;GO:0007165//signal transduction;GO:0065007//biological regulation;GO:0023052//signaling;GO:0009058//biosynthetic process;GO:0044237//cellular metabolic process;GO:0090304//nucleic acid metabolic process;GO:0044700//single organism signaling;GO:0044238//primary metabolic process;GO:0006807//nitrogen compound metabolic process;GO:0008150//biological_process;GO:0044260 [illegible]

id	CON_Mean	KD_Mean	AllMean	log2FoldChange	pvalue	qvalue	State	gene_id	XF_1_count	XF_1_FPKM	XF_1_TPM	XF_1_CPM	XF_2_count	XF_2_FPKM	XF_2_TPM	XF_2_CPM	XF_3_count	XF_3_FPKM	XF_3_TPM	XF_3_CPM	Symbol	Description	KEGG_A_class	KEGG_B_class	Pathway	K_ID	GO Component	GO Function	GO Process
COL27A1	5.971733249	7.354488646	6.663110948	1.384999869	7.16281E-07	0.000254608	Up	ENSG00000196739	2094	4.505301732	17.010722704	63.123782572	4676	11.118912671	44.65494375	155.787085431	4970	12.030938257	48.399262298	168.565475918	COL27A1	collagen type XXVII alpha 1 chain [Source:HGNC Symbol;Acc:HGNC:22986]	Organismal Systems;Cellular Processes;Environmental Information Processing;Human Diseases	Signaling molecules and interaction;Cellular community;Digestive system;Infectious diseases;Signal transduction;Immune system	ko04151//PI3K-Akt signaling pathway;ko04510//Focal adhesion;ko04611//Platelet activation;ko05146//Amoebiasis;ko04512//ECM-receptor interaction;ko04974//Protein digestion and absorption	K06236	GO:0043231//intracellular membrane-bounded organelle;GO:0005737//cytoplasm;GO:0043226//organelle;GO:0005576//extracellular region;GO:0005623//cell;GO:0044421//extracellular region part;GO:0044464//cell part;GO:0005615//extracellular space;GO:0043227//membrane-bounded organelle;GO:0043229//intracellular organelle;GO:0044424//intracellular part;GO:0005783//endoplasmic reticulum;GO:0005622//intracellular;GO:0032991//macromolecular complex;GO:0044444//cytoplasmic part;GO:0012505//endomembrane system;GO:0031012//extracellular matrix;GO:0005575//cellular_component	GO:0043167//ion binding;GO:0003674//molecular_function;GO:0005488//binding;GO:0005198//structural molecule activity	GO:0071840//cellular component organization or biogenesis;GO:0048869//cellular developmental process;GO:0040007//growth;GO:0044763//single-organism cellular process;GO:0030198//extracellular matrix organization;GO:0016043//cellular component organization;GO:0009987//cellular process;GO:0044767//single-organism developmental process;GO:0043062//extracellular structure organization;GO:0008150//biological_process;GO:0032502//developmental process;GO:0030154//cell differentiation;GO:0048856//anatomical structure development;GO:0044699//single-organism process

id	CON_Mean	KD_Mean	AllMean	log2FoldChange	pvalue	qvalue	State	gene_id	XF_1_count	XF_1_FPKM	XF_1_TPM	XF_1_CPM	XF_2_count	XF_2_FPKM	XF_2_TPM	XF_2_CPM	XF_3_count	XF_3_FPKM	XF_3_TPM	XF_3_CPM	Symbol	Description	KEGG_A_class	KEGG_B_class	Pathway	K_ID	GO Component	GO Function	GO Process
WDR90	5.25740712	6.648197916	5.952802518	1.395889888	1.1465E-06	0.000399215	Up	ENSG00000161996	1275	3.64865843	13.776284137	38.434967899	3164	10.006912691	40.189012777	105.412818285	2757	8.876784862	35.710418398	93.508051732	WDR90	WD repeat domain 90 [Source:HGNC Symbol;Acc:HGNC:26960]	-	-	-	-	GO:0043228//non-membrane-bounded organelle;GO:0005575//cellular_component;GO:0043229//intracellular organelle;GO:0043226//organelle;GO:0015630//microtubule cytoskeleton;GO:0005622//intracellular;GO:0044464//cell part;GO:0005737//cytoplasm;GO:0005623//cell;GO:0005856//cytoskeleton;GO:0044424//intracellular part;GO:0043232//intracellular non-membrane-bounded organelle;GO:0044446//intracellular organelle part;GO:0005815//microtubule organizing center;GO:0044430//cytoskeletal part;GO:0044422//organelle part	GO:0005488//binding;GO:0005515//protein binding;GO:0003674//molecular_function;GO:0008092//cytoskeletal protein binding	GO:0016043//cellular component organization;GO:0008150//biological_process;GO:0009987//cellular process;GO:0071840//cellular component organization or biogenesis;GO:0022607//cellular component assembly;GO:0044085//cellular component biogenesis
ATAD3B	5.201015724	6.733464142	5.967239933	1.558135923	1.19515E-06	0.000407833	Up	ENSG00000160072	1226	4.808464654	18.155378643	36.957859328	3772	16.350395179	65.665231722	125.669137349	2603	11.486455392	46.208862143	88.284896144	ATAD3B	ATPase family AAA domain containing 3B [Source:HGNC Symbol;Acc:HGNC:24007]	-	-	-	-	GO:0031410//cytoplasmic vesicle;GO:0043226//organelle;GO:0044444//cytoplasmic part;GO:0043231//intracellular membrane-bounded organelle;GO:0043227//membrane-bounded organelle;GO:0005886//plasma membrane;GO:0097708//intracellular vesicle;GO:0044464//cell part;GO:0005623//cell;GO:0071944//cell periphery;GO:0044424//intracellular part;GO:0005622//intracellular;GO:0031982//vesicle;GO:0016020//membrane;GO:0043229//intracellular organelle;GO:0005575//cellular_component;GO:0005737//cytoplasm;GO:0005739//mitochondrion	GO:0043167//ion binding;GO:0005488//binding;GO:0003674//molecular_function	GO:0007005//mitochondrion organization;GO:0016192//vesicle-mediated transport;GO:0006996//organelle organization;GO:0009987//cellular process;GO:0006810//transport;GO:0002376//immune system process;GO:0008150//biological_process;GO:0016043//cellular component organization;GO:0071840//cellular component organization or biogenesis;GO:0051234//establishment of localization;GO:0051179//localization

id	CON_Mean	KD_Mean	AllMean	log2FoldChange	pvalue	qvalue	State	gene_id	XF_1_count	XF_1_FPKM	XF_1_TPM	XF_1_CPM	XF_2_count	XF_2_FPKM	XF_2_TPM	XF_2_CPM	XF_3_count	XF_3_FPKM	XF_3_TPM	XF_3_CPM	Symbol	Description	KEGG_A_class	KEGG_B_class	Pathway	K_ID	GO Component	GO Function	GO Process
HES7	3.438312113	0.909148679	2.173730396	-2.58952	1.22762E-06	0.0004107	Down	ENSG00000179111	359	5.756426148	21.734608418	10.822081157	51	0.903793631	3.629748246	1.699132027	54	0.974200062	3.919109493	1.831496117	HES7	hes family bHLH transcription factor 7 [Source:HGNC Symbol;Acc:HGNC:15977]	-	-	-	-	GO:0044424//intracellular part;GO:0005623//cell;GO:0043229//intracellular organelle;GO:0070013//intracellular organelle lumen;GO:0043228//non-membrane-bounded organelle;GO:0043227//membrane-bounded organelle;GO:0031974//membrane-enclosed lumen;GO:0043233//organelle lumen;GO:0043226//organelle;GO:0044446//intracellular organelle part;GO:0000228//nuclear chromosome;GO:0043231//intracellular membrane-bounded organelle;GO:0005694//chromosome;GO:0005575//cellular_component;GO:0031981//nuclear lumen;GO:0044428//nuclear part;GO:0005634//nucleus;GO:0043232//intracellular non-membrane-bounded organelle;GO:0044422//organelle part;GO:0005622//intracellular;GO:0044464//cell part	GO:0001071//nucleic acid binding transcription factor activity;GO:0008134//transcription factor binding;GO:1901363//heterocyclic compound binding;GO:0005488//binding;GO:0003700//transcription factor activity, sequence-specific DNA binding;GO:0003677//DNA binding;GO:0005515//protein binding;GO:0097159//organic cyclic compound binding;GO:0003674//molecular_function;GO:0003676//nucleic acid binding	GO:0009987//cellular process;GO:0044767//single-organism developmental process;GO:0044237//cellular metabolic process;GO:0032501//multicellular organismal process;GO:0050896//response to stimulus;GO:0034641//cellular nitrogen compound metabolic process;GO:0050789//regulation of biological process;GO:0023052//signaling;GO:0008152//metabolic process;GO:0044763//single-organism cellular process;GO:0044707//single-multicellular organism process;GO:0009058//biosynthetic process;GO:0044700//single organism signaling;GO:0065007//biological regulation;GO:0007165//signal transduction;GO:0044699//single-organism process;GO:0048869//cellular developmental process;GO:0006807//nitrogen compound metabolic process;GO:0007154//cell communication;GO:0008150//biological_process;GO:0050794//regulation of cellular process;GO:0007275//multicellular organism development;GO:0009790//embryo development;GO:0009653//anatomical structure morphogenesis;GO:0048856//anatomical structure [illegible]
MSTRG.20666	5.079879914	3.013800356	4.046840135	-2.05133	1.40527E-06	0.00046109	Down	MSTRG.20666	1127	4.810747255	18.163997089	33.973497115	194	0.915231516	3.675684221	6.463364964	285	1.368766568	5.506411116	9.666229504	-	-	-	-	-	-	-	-	-
ENSG00000166104	4.733901083	6.152798514	5.443349798	1.422137632	1.48594E-06	0.000478362	Up	ENSG00000166104	886	6.855373346	25.883916759	26.708534555	2125	18.171757644	72.980051147	70.797167778	2064	17.968134386	72.284008993	70.00385	-	-	-	-	-	-	-	-	-

id	CON_Mean	KD_Mean	AllMean	log2FoldChange	pvalue	qvalue	State	gene_id	XF_1_count	XF_1_FPKM	XF_1_TPM	XF_1_CPM	XF_2_count	XF_2_FPKM	XF_2_TPM	XF_2_CPM	XF_3_count	XF_3_FPKM	XF_3_TPM	XF_3_CPM	Symbol	Description	KEGG_A_class	KEGG_B_class	Pathway	K_ID	GO Component	GO Function	GO Process
GPR153	6.086085259	4.679946841	5.38301605	-1.40746	1.66202E-06	0.000525136	Down	ENSG00000158292	2267	16.278913811	61.464493435	68.338880177	718	5.698216682	22.884750779	23.921113631	789	6.374510067	25.644016962	26.760193259	GPR153	G protein-coupled receptor 153 [Source:HGNC Symbol;Acc:HGNC:23618]	-	-	-	-	GO:0005886//plasma membrane;GO:0005623//cell;GO:0071944//cell periphery;GO:0005575//cellular_component;GO:0016020//membrane;GO:0044464//cell part	GO:0003674//molecular_function	GO:0023052//signaling;GO:0050794//regulation of cellular process;GO:0044699//single-organism process;GO:0007165//signal transduction;GO:0044700//single organism signaling;GO:0051716//cellular response to stimulus;GO:0008150//biological_process;GO:0044763//single-organism cellular process;GO:0050789//regulation of biological process;GO:0009987//cellular process;GO:0007154//cell communication;GO:0050896//response to stimulus;GO:0065007//biological regulation
ATP8A1	4.409641436	2.169867226	3.289754331	-2.26057	1.72201E-06	0.0005342	Down	ENSG00000124406	707	1.903077642	7.185473466	21.312566513	122	0.362942257	1.457621492	4.064590338	138	0.417938216	1.681323675	4.680490076	ATP8A1	ATPase phospholipid transporting 8A1 [Source:HGNC Symbol;Acc:HGNC:13531]	-	-	-	-	GO:0043231//intracellular membrane-bounded organelle;GO:0005623//cell;GO:0031410//cytoplasmic vesicle;GO:0071944//cell periphery;GO:0016020//membrane;GO:0043227//membrane-bounded organelle;GO:0005783//endoplasmic reticulum;GO:0005773//vacuole;GO:0097708//intracellular vesicle;GO:0005886//plasma membrane;GO:0031982//vesicle;GO:0005764//lysosome;GO:0005794//Golgi apparatus;GO:0043226//organelle;GO:0005575//cellular_component;GO:0000323//lytic vacuole;GO:0012505//endomembrane system;GO:0044424//intracellular part;GO:0005576//extracellular region;GO:0005622//intracellular;GO:0005737//cytoplasm;GO:0044464//cell part;GO:0043229//intracellular organelle;GO:0005615//extracellular space;GO:0044421//extracellular region part;GO:0044444//cytoplasmic part	GO:0043167//ion binding;GO:0003674//molecular_function;GO:0016787//hydrolase activity;GO:0017111//nucleoside-triphosphatase activity;GO:0005215//transporter activity;GO:0005488//binding;GO:0016817//hydrolase activity, acting on acid anhydrides;GO:0003824//catalytic activity;GO:0016462//pyrophosphatase activity;GO:0016887//ATPase activity;GO:0022857//transmembrane transporter activity;GO:0016818//hydrolase activity, acting on acid anhydrides, in phosphorus-containing anhydrides	GO:0044699//single-organism process;GO:0040011//locomotion;GO:0051179//localization;GO:0055085//transmembrane transport;GO:0032501//multicellular organismal process;GO:0071840//cellular component organization or biogenesis;GO:0002376//immune system process;GO:0008150//biological_process;GO:0061024//membrane organization;GO:0016043//cellular component organization;GO:0006810//transport;GO:0051234//establishment of localization;GO:0003008//system process;GO:0051674//localization of cell;GO:0044763//single-organism cellular process;GO:0006928//movement of cell or subcellular component;GO:0048870//cell motility;GO:0009987//cellular process;GO:0050877//neurological system process;GO:0016192//vesicle-mediated transport

id	CON_Mean	KD_Mean	AllMean	log2FoldChange	pvalue	qvalue	State	gene_id	XF_1_count	XF_1_FPKM	XF_1_TPM	XF_1_CPM	XF_2_count	XF_2_FPKM	XF_2_TPM	XF_2_CPM	XF_3_count	XF_3_FPKM	XF_3_TPM	XF_3_CPM	Symbol	Description	KEGG_A_class	KEGG_B_class	Pathway	K_ID	GO Component	GO Function	GO Process
FAM20A	3.645688708	1.187863469	2.416776089	-2.50183	1.84475E-06	0.000560751	Down	ENSG00000108950	415	2.02397755	7.641956723	12.510205238	59	0.318016913	1.277195693	1.965662541	70	0.384106396	1.545221645	2.374161633	FAM20A	FAM20A golgi associated secretory pathway pseudokinase [Source:HGNC Symbol;Acc:HGNC:23015]	-	-	-	-	GO:0005576//extracellular region;GO:0043231//intracellular membrane-bounded organelle;GO:0043227//membrane-bounded organelle;GO:0044424//intracellular part;GO:0005794//Golgi apparatus;GO:0012505//endomembrane system;GO:0043229//intracellular organelle;GO:0044464//cell part;GO:0044444//cytoplasmic part;GO:0043226//organelle;GO:0005623//cell;GO:0005783//endoplasmic reticulum;GO:0005615//extracellular space;GO:0044421//extracellular region part;GO:0005622//intracellular;GO:0005575//cellular_component;GO:0005737//cytoplasm	GO:0098772//molecular function regulator;GO:0003674//molecular_function;GO:0030234//enzyme regulator activity	GO:0071704//organic substance metabolic process;GO:0043170//macromolecule metabolic process;GO:0009987//cellular process;GO:0008152//metabolic process;GO:0042592//homeostatic process;GO:0008150//biological_process;GO:0065008//regulation of biological quality;GO:0043412//macromolecule modification;GO:0048646//anatomical structure formation involved in morphogenesis;GO:0006464//cellular protein modification process;GO:0044238//primary metabolic process;GO:0065007//biological regulation;GO:0044260//cellular macromolecule metabolic process;GO:0044267//cellular protein metabolic process;GO:0009653//anatomical structure morphogenesis;GO:0044237//cellular metabolic process;GO:0019538//protein metabolic process;GO:0032502//developmental process;GO:0036211//protein modification process;GO:0048856//anatomical structure development
CLIP4	7.342389504	6.144373763	6.743381634	-1.19862	1.87333E-06	0.000560751	Down	ENSG00000115295	5420	20.998110129	79.282820533	163.38629491	2040	8.734774588	35.079947063	67.965281069	2125	9.262660821	37.26275882	72.072763848	CLIP4	CAP-Gly domain containing linker protein family member 4 [Source:HGNC Symbol;Acc:HGNC:26108]	-	-	-	-	GO:0005575//cellular_component;GO:0005622//intracellular;GO:0044464//cell part;GO:0043226//organelle;GO:0005623//cell	GO:0003674//molecular_function	-
PCDH10	5.77527574	4.237437992	5.006356866	-1.53323	2.08686E-06	0.000613896	Down	ENSG00000138650	1827	5.409059921	20.423053521	55.075048118	623	2.038505576	8.186893317	20.756063777	491	1.635538011	6.579605969	16.653048023	PCDH10	protocadherin 10 [Source:HGNC Symbol;Acc:HGNC:13404]	-	-	-	-	GO:0071944//cell periphery;GO:0044464//cell part;GO:0005623//cell;GO:0005575//cellular_component;GO:0016020//membrane;GO:0005886//plasma membrane	GO:0005488//binding;GO:0003674//molecular_function;GO:0043167//ion binding	GO:0022610//biological adhesion;GO:0008150//biological_process;GO:0007155//cell adhesion

id	CON_Mean	KD_Mean	AllMean	log2FoldChange	pvalue	qvalue	State	gene_id	XF_1_count	XF_1_FPKM	XF_1_TPM	XF_1_CPM	XF_2_count	XF_2_FPKM	XF_2_TPM	XF_2_CPM	XF_3_count	XF_3_FPKM	XF_3_TPM	XF_3_CPM	Symbol	Description	KEGG_A_class	KEGG_B_class	Pathway	K_ID	GO Component	GO Function	GO Process
KRTAP2-3	-2.66856	1.330946426	-0.66881	5.380313263	2.19369E-06	0.000624601	Up	ENSG00000212724	2	0.068903024	0.260157987	0.060290146	112	4.264488224	17.12671799 8	3.731427196	45	1.744282016	7.017072235	1.526246764	KRTAP2-3	keratin associated protein 2-3 [Source:HGNC Symbol;Acc:HGNC:18906]	-	-	-	-	GO:0043232//intracellular non-membrane-bounded organelle;GO:0005622//intracellular;GO:0044424//intracellular part;GO:0044444//cytoplasmic part;GO:0043226//organelle;GO:0043229//intracellular organelle;GO:0044464//cell part;GO:0005737//cytoplasm;GO:0005829//cytosol;GO:0005623//cell;GO:0043228//non-membrane-bounded organelle;GO:0005856//cytoskeleton;GO:0005575//cellular_component	GO:0003674//molecular_function	GO:0030154//cell differentiation;GO:0009987//cellular process;GO:0044767//single-organism developmental process;GO:0044699//single-organism process;GO:0048856//anatomical structure development;GO:0044763//single-organism cellular process;GO:0048869//cellular developmental process;GO:0008150//biological_process;GO:0032502//developmental process
PPARG	5.889645584	4.433926209	5.161785897	-1.45721	2.19646E-06	0.000624601	Down	ENSG00000132170	1978	4.976792765	18.79093714 7	59.62695412	676	1.879795379	7.549493317	22.52182843 3	595	1.684364734	6.776030998	20.18037387 8	PPARG	peroxisome proliferator activated receptor gamma [Source:HGNC Symbol;Acc:HGNC:9236]	Environmental Information Processing;Human Diseases;Organismal Systems	Neurodegenerative diseases;Cancers;Endocrine system; Development;Signal transduction	ko05200//Pathways in cancer;ko05016//Huntington's disease; ko04380//Osteoclast differentiation;ko05202//Transcriptional misregulation in cancers; ko04152//MAPK signaling pathway; ko03320//P[illegible]	K08530	GO:0000228//nuclear chromosome;GO:0044444//cytoplasmic part;GO:0005737//cytoplasm;GO:0044424//intracellular part;GO:0044446//intracellular organelle part;GO:0043227//membrane-bounded organelle;GO:0031981//nuclear lumen;GO:0043229//intracellular organelle;GO:0031974//membrane-enclosed lumen;GO:0005575//cellular_component;GO:0005694//chromosome;GO:0005623//cell;GO:0005829//cytosol;GO:0005654//nucleoplasm;GO:0043232//intracellular non-membrane-bounded organelle;GO:0043231//intracellular membrane-bounded organelle;GO:0043226//organelle;GO:0005634//nucleus;GO:0044428//nuclear part;GO:0032991//macromolecular complex;GO:0070013//intracellular organelle lumen;GO:0005622//intracellular;GO:0043228//non-membrane-bounded organelle;GO:0044422//organelle part;GO:0044464//cell part;GO:0043233//organelle lumen	GO:0003676//nucleic acid binding;GO:0003674//molecular_function;GO:0008134//transcription factor binding;GO:1901363//heterocyclic compound binding;GO:0019899//enzyme binding;GO:0097159//organic cyclic compound binding;GO:0001071//nucleic acid binding transcription factor activity;GO:0003677//DNA binding;GO:0043167//ion binding;GO:0008092//cytoskeletal protein binding;GO:0005488//binding;GO:0005515//protein binding;GO:0003700//transcription factor activity, sequence-specific DNA binding;GO:0008289//lipid binding	GO:0032502//developmental process;GO:0006629//lipid metabolic process;GO:0034641//cellular nitrogen compound metabolic process;GO:0044700//single organism signaling;GO:0061024//membrane organization;GO:0023052//signaling;GO:0040011//locomotion;GO:0006259//DNA metabolic process;GO:0007154//cell communication;GO:0044699//single-organism process;GO:0051716//cellular response to stimulus;GO:0006950//response to stress;GO:1901360//organic cyclic compound metabolic process;GO:0002376//immune system process;GO:0009058//biosynthetic process;GO:0016043//cellular component organization;GO:0044763//single-organism cellular process;GO:0044238//primary metabolic process;GO:0048646//anatomical structure formation involved in morphogenesis;GO:0042592//homeostatic process;GO:0009653//anatomical structure morphogenesis;GO:0003013//circulatory system process;GO:0021700//developmental maturation;GO:0045184//establishment of protein [illegible]

id	CON_Mean	KD_Mean	AllMean	log2FoldChange	pvalue	qvalue	State	gene_id	XF_1_count	XF_1_FPKM	XF_1_TPM	XF_1_CPM	XF_2_count	XF_2_FPKM	XF_2_TPM	XF_2_CPM	XF_3_count	XF_3_FPKM	XF_3_TPM	XF_3_CPM	Symbol	Description	KEGG_A_class	KEGG_B_class	Pathway	K_ID	GO Component	GO Function	GO Process
TFRC	7.172340004	8.477949463	7.825144734	1.307067795	2.4267E-06	0.000678759	Up	ENSG00000072274	4817	18.630846289	70.344713579	145.208815975	10942	46.772788109	187.845366165	364.547110518	10088	43.899231186	176.602219997	342.15060786	TFRC	transferrin receptor [Source:HGNC Symbol;Acc:HGNC:11763]	Cellular Processes;Environmental Information Processing;Organismal Systems	Signal transduction;Immune system;Transport and catabolism	ko04144//Endocytosis;ko04145//Phagosome;ko04640//Hematopoietic cell lineage;ko04066//HIF-1 signaling pathway	K06503	GO:0005623//cell;GO:0044424//intracellular part;GO:0005576//extracellular region;GO:0005773//vacuole;GO:0032991//macromolecular complex;GO:0005768//endosome;GO:0005737//cytoplasm;GO:0043226//organelle;GO:0016020//membrane;GO:0031410//cytoplasmic vesicle;GO:0012505//endomembrane system;GO:0071944//cell periphery;GO:0005615//extracellular space;GO:0044464//cell part;GO:0043229//intracellular organelle;GO:0043231//intracellular membrane-bounded organelle;GO:0044421//extracellular region part;GO:0005575//cellular_component;GO:0031982//vesicle;GO:0097708//intracellular vesicle;GO:0005886//plasma membrane;GO:0044444//cytoplasmic part;GO:0005622//intracellular;GO:0043227//membrane-bounded organelle	GO:0003674//molecular_function;GO:0019899//enzyme binding;GO:0097159//organic cyclic compound binding;GO:0005515//protein binding;GO:1901363//heterocyclic compound binding;GO:0005488//binding;GO:0003723//RNA binding;GO:0003676//nucleic acid binding	GO:0044700//single organism signaling;GO:0022610//biological adhesion;GO:0008219//cell death;GO:0006259//DNA metabolic process;GO:0036211//protein modification process;GO:0009987//cellular process;GO:0002376//immune system process;GO:0007154//cell communication;GO:0019538//protein metabolic process;GO:0022607//cellular component assembly;GO:0051179//localization;GO:0090304//nucleic acid metabolic process;GO:0044260//cellular macromolecule metabolic process;GO:0043412//macromolecule modification;GO:0044238//primary metabolic process;GO:0051704//multi-organism process;GO:0065007//biological regulation;GO:0044403//symbiosis, encompassing mutualism through parasitism;GO:0051716//cellular response to stimulus;GO:0045184//establishment of protein localization;GO:0044237//cellular metabolic process;GO:0043933//macromolecular complex subunit organization;GO:0050789//regulation of biological process;GO:0065008//regulation of biological [illegible]
DDX12P	4.17407272	5.730339951	4.952206336	1.567043756	2.51063E-06	0.000684758	Up	ENSG00000214826	600	6.137442727	23.173217365	18.087043717	1718	19.422269386	78.002262685	57.237427881	1420	16.342573651	65.744540604	48.161564548	-	-	-	-	-	-	-	-	-

id	CON_Mean	KD_Mean	AllMean	log2FoldChange	pvalue	qvalue	State	gene_id	XF_1_count	XF_1_FPKM	XF_1_TPM	XF_1_CPM	XF_2_count	XF_2_FPKM	XF_2_TPM	XF_2_CPM	XF_3_count	XF_3_FPKM	XF_3_TPM	XF_3_CPM	Symbol	Description	KEGG_A_class	KEGG_B_class	Pathway	K_ID	GO Component	GO Function	GO Process
HCFC1	7.449014791	8.764141795	8.106578293	1.316558045	2.52841E-06	0.000684758	Up	ENSG00000172534	5836	19.523542917	73.715279125	175.926645221	13361	49.399542191	198.394739048	445.139274687	12286	46.243400635	186.032579429	416.699283125	HCFC1	host cell factor C1 [Source:HGNC Symbol;Acc:HGNC:4839]	Human Diseases	Infectious diseases	ko05168//Herpes simplex infection	K14966	GO:0005634//nucleus;GO:0031981//nuclear lumen;GO:0044428//nuclear part;GO:0044422//organelle part;GO:0005575//cellular_component;GO:0032991//macromolecular complex;GO:0005737//cytoplasm;GO:0043227//membrane-bounded organelle;GO:0005622//intracellular;GO:0043231//intracellular membrane-bounded organelle;GO:0043226//organelle;GO:0044464//cell part;GO:0043233//organelle lumen;GO:0070013//intracellular organelle lumen;GO:0043229//intracellular organelle;GO:0044446//intracellular organelle part;GO:0005654//nucleoplasm;GO:0031974//membrane-enclosed lumen;GO:0044424//intracellular part;GO:0005623//cell	GO:0005488//binding;GO:0008134//transcription factor binding;GO:0016740//transferase activity;GO:0060090//binding, bridging;GO:0097159//organic cyclic compound binding;GO:0030674//protein binding, bridging;GO:0003677//DNA binding;GO:0003676//nucleic acid binding;GO:0003824//catalytic activity;GO:0003674//molecular_function;GO:0016746//transferase activity, transferring acyl groups;GO:1901363//heterocyclic compound binding;GO:0005515//protein binding	GO:0044238//primary metabolic process;GO:0007275//multicellular organism development;GO:0044763//single-organism cellular process;GO:0043412//macromolecule modification;GO:0043170//macromolecule metabolic process;GO:0009987//cellular process;GO:0048856//anatomical structure development;GO:0006807//nitrogen compound metabolic process;GO:0032501//multicellular organismal process;GO:0044707//single-multicellular organism process;GO:0044267//cellular protein metabolic process;GO:0044403//symbiosis, encompassing mutualism through parasitism;GO:0008152//metabolic process;GO:0016043//cellular component organization;GO:0051704//multi-organism process;GO:0032502//developmental process;GO:0034641//cellular nitrogen compound metabolic process;GO:0007005//mitochondrion organization;GO:0065003//macromolecular complex assembly;GO:0051276//chromosome organization;GO:0036211//protein modification process;GO:0044767//single-organism developmental process;GO:0009058//bios[illegible]

id	CON_Mean	KD_Mean	AllMean	log2FoldChange	pvalue	qvalue	State	gene_id	XF_1_count	XF_1_FPKM	XF_1_TPM	XF_1_CPM	XF_2_count	XF_2_FPKM	XF_2_TPM	XF_2_CPM	XF_3_count	XF_3_FPKM	XF_3_TPM	XF_3_CPM	Symbol	Description	KEGG_A_class	KEGG_B_class	Pathway	K_ID	GO Component	GO Function	GO Process
GNG13	-3.35941	0.202928778	-1.57824	8.044727491	2.69112E-06	0.000717435	Up	ENSG00000127588	0	0	0	0	37	1.251475764	5.026083169	1.232703627	26	0.895260369	3.601542997	0.881831464	GNG13	G protein subunit gamma 13 [Source:HGNC Symbol;Acc:HGNC:14131]	Organismal Systems;Environmental Information Processing;Human Diseases	Immune system;Substance dependence;Nervous system;Signal transduction;Environmental adaptation;Cancers;Sensory system	ko05200//Pathways in cancer;ko04151//PI3K-Akt signaling pathway;ko04014//Ras signaling pathway;ko04062//Chemokine signaling pathway;ko05034//Alcoholism;ko04728//Dopami	K04547	GO:0071944//cell periphery;GO:0005622//intracellular;GO:0032991//macromolecular complex;GO:0044464//cell part;GO:0016020//membrane;GO:0005575//cellular_component;GO:0005886//plasma membrane;GO:0005623//cell	GO:0016787//hydrolase activity;GO:0003824//catalytic activity;GO:0016462//pyrophosphatase activity;GO:0003674//molecular_function;GO:0016817//hydrolase activity, acting on acid anhydrides;GO:0016818//hydrolase activity, acting on acid anhydrides, in phosphorus-containing anhydrides;GO:0003924//GTPase activity;GO:0017111//nucleoside-triphosphatase activity	GO:0050877//neurological system process;GO:0023052//signaling;GO:0051716//cellular response to stimulus;GO:0050794//regulation of cellular process;GO:0007165//signal transduction;GO:0003008//system process;GO:0044763//single-organism cellular process;GO:0050896//response to stimulus;GO:0065007//biological regulation;GO:0008150//biological_process;GO:0044699//single-organism process;GO:0050789//regulation of biological process;GO:0007154//cell communication;GO:0032501//multicellular organismal process;GO:0009987//cellular process;GO:0044700//single organism signaling

id	CON_Mean	KD_Mean	AllMean	log2FoldChange	pvalue	qvalue	State	gene_id	XF_1_count	XF_1_FPKM	XF_1_TPM	XF_1_CPM	XF_2_count	XF_2_FPKM	XF_2_TPM	XF_2_CPM	XF_3_count	XF_3_FPKM	XF_3_TPM	XF_3_CPM	Symbol	Description	KEGG_A_class	KEGG_B_class	Pathway	K_ID	GO Component	GO Function	GO Process
SEMA3C	7.74605945	6.536416653	7.141238052	-1.20515	2.90207E-06	0.000761771	Down	ENSG00000075223	7171	35.437756966	133.802771222	216.170317491	2521	13.768922659	55.297715258	83.990428223	2963	16.474568894	66.275544275	100.494870251	SEMA3C	semaphorin 3C [Source:HGNC Symbol;Acc:HGNC:10725]	Organismal Systems	Development	ko04360//Axon guidance	K06840	GO:0016020//membrane;GO:0005575//cellular_component;GO:0005576//extracellular region;GO:0044421//extracellular region part;GO:0005886//plasma membrane;GO:0005615//extracellular space;GO:0043226//organelle;GO:0071944//cell periphery;GO:0044464//cell part;GO:0005623//cell	GO:0003674//molecular_function	GO:0051716//cellular response to stimulus;GO:0040011//locomotion;GO:0071840//cellular component organization or biogenesis;GO:0032989//cellular component morphogenesis;GO:0065007//biological regulation;GO:0044699//single-organism process;GO:0044707//single-multicellular organism process;GO:0007165//signal transduction;GO:0032502//developmental process;GO:0048869//cellular developmental process;GO:0016043//cellular component organization;GO:0007275//multicellular organism development;GO:0000902//cell morphogenesis;GO:0007154//cell communication;GO:0050794//regulation of cellular process;GO:0044767//single-organism developmental process;GO:0040007//growth;GO:0044763//single-organism cellular process;GO:0032501//multicellular organismal process;GO:0008150//biological_process;GO:0009790//embryo development;GO:0030154//cell differentiation;GO:0006928//movement of cell or subcellular component;GO:0009987//cellular process;GO:0050789//regu[illegible]

id	CON_Mean	KD_Mean	AllMean	log2FoldChange	pvalue	qvalue	State	gene_id	XF_1_count	XF_1_FPKM	XF_1_TPM	XF_1_CPM	XF_2_count	XF_2_FPKM	XF_2_TPM	XF_2_CPM	XF_3_count	XF_3_FPKM	XF_3_TPM	XF_3_CPM	Symbol	Description	KEGG_A_class	KEGG_B_class	Pathway	K_ID	GO Component	GO Function	GO Process
COL7A1	2.398975341	4.567912953	3.483444147	2.193260239	3.31277E-06	0.000856402	Up	ENSG00000114270	173	0.468435966	1.768679389	5.215097605	648	1.939187248	7.788018492	21.588971634	748	2.27877597	9.167287998	25.369612875	COL7A1	collagen type VII alpha 1 chain [Source:HGNC Symbol;Acc:HGNC:2214]	Organismal Systems	Digestive system	ko04974//Protein digestion and absorption	K16628	GO:0005623//cell;GO:0044424//intracellular part;GO:0005576//extracellular region;GO:0043231//intracellular membrane-bounded organelle;GO:0012505//endomembrane system;GO:0032991//macromolecular complex;GO:0097708//intracellular vesicle;GO:0043229//intracellular organelle;GO:0005615//extracellular space;GO:0031012//extracellular matrix;GO:0005622//intracellular;GO:0044444//cytoplasmic part;GO:0043227//membrane-bounded organelle;GO:0005575//cellular_component;GO:0031410//cytoplasmic vesicle;GO:0044421//extracellular region part;GO:0031982//vesicle;GO:0044464//cell part;GO:0043226//organelle;GO:0005794//Golgi apparatus;GO:0005737//cytoplasm;GO:0005783//endoplasmic reticulum	GO:0005198//structural molecule activity;GO:0003674//molecular_function;GO:0030234//enzyme regulator activity;GO:0098772//molecular function regulator	GO:0032501//multicellular organismal process;GO:0048856//anatomical structure development;GO:0061024//membrane organization;GO:0044699//single-organism process;GO:0030198//extracellular matrix organization;GO:0071840//cellular component organization or biogenesis;GO:0007275//multicellular organism development;GO:0048646//anatomical structure formation involved in morphogenesis;GO:0008150//biological_process;GO:0032502//developmental process;GO:0016192//vesicle-mediated transport;GO:0043062//extracellular structure organization;GO:0044707//single-multicellular organism process;GO:0048869//cellular developmental process;GO:0051234//establishment of localization;GO:0016043//cellular component organization;GO:0044763//single-organism cellular process;GO:0000902//cell morphogenesis;GO:0022607//cellular component assembly;GO:0043933//macromolecular complex subunit organization;GO:0044085//cellular component biogenesis;GO:0032989//cellular component morphogenesis;GO:0044767//single-organism [illegible]

id	CON_Mean	KD_Mean	AllMean	log2FoldChange	pvalue	qvalue	State	gene_id	XF_1_count	XF_1_FPKM	XF_1_TPM	XF_1_CPM	XF_2_count	XF_2_FPKM	XF_2_TPM	XF_2_CPM	XF_3_count	XF_3_FPKM	XF_3_TPM	XF_3_CPM	Symbol	Description	KEGG_A_class	KEGG_B_class	Pathway	K_ID	GO Component	GO Function	GO Process
NR4A1	5.628540433	6.935262386	6.281901409	1.311258686	3.73725E-06	0.000951717	Up	ENSG00000123358	1650	6.818282416	25.7438720781	49.739370222	3400	15.527822954	62.361678798	113.275468448	3821	17.764949767	71.466617574	129.595308548	NR4A1	nuclear receptor subfamily 4 group A member 1 [Source:HGNC Symbol;Acc:HGNC:7980]	Organismal Systems; Environmental Information Processing	Signal transduction; Endocrine system	ko04151//PI3K-Akt signaling pathway; ko04010//MAPK signaling pathway; ko04925//Aldosterone synthesis and secretion	K04465	GO:0044464//cell part;GO:0070013//intracellular organelle lumen;GO:0031975//envelope;GO:0044444//cytoplasmic part;GO:0031981//nuclear lumen;GO:0043233//organelle lumen;GO:0000228//nuclear chromosome;GO:0043227//membrane-bounded organelle;GO:0043226//organelle;GO:0044428//nuclear part;GO:0032991//macromolecular complex;GO:0005623//cell;GO:0044446//intracellular organelle part;GO:0012505//endomembrane system;GO:0044422//organelle part;GO:0005575//cellular_component;GO:0005739//mitochondrion;GO:0005829//cytosol;GO:0031967//organelle envelope;GO:0043228//non-membrane-bounded organelle;GO:0043231//intracellular membrane-bounded organelle;GO:0005622//intracellular;GO:0031974//membrane-enclosed lumen;GO:0043232//intracellular non-membrane-bounded organelle;GO:0005737//cytoplasm;GO:0043229//intracellular organelle;GO:0005634//nucleus;GO:0005694//chromosome;GO:0005635//nuclear envelope;GO:0005654//nucleoplasm;GO:0044424//intracellular part	GO:0001071//nucleic acid binding transcription factor activity;GO:0097159//organic cyclic compound binding;GO:0008134//transcription factor binding;GO:0003674//molecular_function;GO:1901363//heterocyclic compound binding;GO:0003676//nucleic acid binding;GO:0003677//DNA binding;GO:0043167//ion binding;GO:0003700//transcription factor activity, sequence-specific DNA binding;GO:0005515//protein binding;GO:0005488//binding	GO:0044763//single-organism cellular process;GO:0051179//localization;GO:0048646//anatomical structure formation involved in morphogenesis;GO:0008219//cell death;GO:0008152//metabolic process;GO:0008283//cell proliferation;GO:0048869//cellular developmental process;GO:0008150//biological_process;GO:0040011//locomotion;GO:0006928//movement of cell or subcellular component;GO:0051674//localization of cell;GO:0009653//anatomical structure morphogenesis;GO:0050789//regulation of biological process;GO:0044699//single-organism process;GO:0007154//cell communication;GO:0034641//cellular nitrogen compound metabolic process;GO:0023052//signaling;GO:0051716//cellular response to stimulus;GO:0007049//cell cycle;GO:0065007//biological regulation;GO:0050896//response to stimulus;GO:0050794//regulation of cellular process;GO:0044700//single organism signaling;GO:0009987//cellular process;GO:0006807//nitrogen compound metabolic process;GO:0048856//anatomical structure [illegible]

id	CON_Mean	KD_Mean	AllMean	log2FoldChange	pvalue	qvalue	State	gene_id	XF_1_count	XF_1_FPKM	XF_1_TPM	XF_1_CPM	XF_2_count	XF_2_FPKM	XF_2_TPM	XF_2_CPM	XF_3_count	XF_3_FPKM	XF_3_TPM	XF_3_CPM	Symbol	Description	KEGG_A_class	KEGG_B_class	Pathway	K_ID	GO Component	GO Function	GO Process
ZDHHC11	2.407137429	4.536377793	3.471757611	2.152529888	5.1309E-06	0.001286576	Up	ENSG00000188818	174	0.754385543	2.848342695	5.245242678	734	3.517068123	14.124985405	24.454174659	632	3.082883343	12.402131601	21.435287883	ZDHHC11	zinc finger DHHC-type containing 11 [Source:HGNC Symbol;Acc:HGNC:19158]	-	-	-	-	GO:0043229//intracellular organelle;GO:0005623//cell;GO:0005794//Golgi apparatus;GO:0044464//cell part;GO:0005737//cytoplasm;GO:0043231//intracellular membrane-bounded organelle;GO:0044424//intracellular part;GO:0043227//membrane-bounded organelle;GO:0012505//endomembrane system;GO:0005622//intracellular;GO:0044444//cytoplasmic part;GO:0005575//cellular_component;GO:0005783//endoplasmic reticulum;GO:0043226//organelle	GO:0016740//transferase activity;GO:0003674//molecular_function;GO:0003824//catalytic activity;GO:0016746//transferase activity, transferring acyl groups	GO:0051649//establishment of localization in cell;GO:0070727//cellular macromolecule localization;GO:0044260//cellular macromolecule metabolic process;GO:0008150//biological_process;GO:0051234//establishment of localization;GO:0043412//macromolecule modification;GO:0071704//organic substance metabolic process;GO:0036211//protein modification process;GO:0071702//organic substance transport;GO:0008152//metabolic process;GO:0006810//transport;GO:0009987//cellular process;GO:0008104//protein localization;GO:0046907//intracellular transport;GO:0045184//establishment of protein localization;GO:0051641//cellular localization;GO:0015031//protein transport;GO:0044237//cellular metabolic process;GO:0044238//primary metabolic process;GO:0009058//biosynthetic process;GO:0044267//cellular protein metabolic process;GO:0006605//protein targeting;GO:0043170//macromolecule metabolic process;GO:0033036//macromolecule localization;GO:0019538//p [illegible]

id	CON_Mean	KD_Mean	AllMean	log2FoldChange	pvalue	qvalue	State	gene_id	XF_1_count	XF_1_FPKM	XF_1_TPM	XF_1_CPM	XF_2_count	XF_2_FPKM	XF_2_TPM	XF_2_CPM	XF_3_count	XF_3_FPKM	XF_3_TPM	XF_3_CPM	Symbol	Description	KEGG_A_class	KEGG_B_class	Pathway	K_ID	GO Component	GO Function	GO Process
SYVN1	5.248349602	6.530829506	5.889589554	1.285624561	5.20301E-06	0.001286576	Up	ENSG00000162298	1267	6.950647373	26.243644066	38.193807316	2634	15.970003955	64.137532995	87.755171733	2814	17.368752981	69.8727575	95.441297633	SYVN1	synoviolin 1 [Source:HGNC Symbol;Acc:HGNC:20738]	Genetic Information Processing	Folding, sorting and degradation	ko04141//Protein processing in endoplasmic reticulum;ko04120//Ubiquitin mediated proteolysis	K10601	GO:0031974//membrane-enclosed lumen;GO:0012505//endomembrane system;GO:0005634//nucleus;GO:0044444//cytoplasmic part;GO:0044424//intracellular part;GO:0043227//membrane-bounded organelle;GO:0044446//intracellular organelle part;GO:0031981//nuclear lumen;GO:0044422//organelle part;GO:0005783//endoplasmic reticulum;GO:0043226//organelle;GO:0005622//intracellular;GO:0043233//organelle lumen;GO:0044464//cell part;GO:0043229//intracellular organelle;GO:0032991//macromolecular complex;GO:0070013//intracellular organelle lumen;GO:0044428//nuclear part;GO:0005654//nucleoplasm;GO:0005737//cytoplasm;GO:0005623//cell;GO:0005575//cellular_component;GO:0043231//intracellular membrane-bounded organelle	GO:0005515//protein binding;GO:0005488//binding;GO:0051082//unfolded protein binding;GO:0003674//molecular_function;GO:0043167//ion binding;GO:0019899//enzyme binding	GO:0023052//signaling;GO:0071702//organic substance transport;GO:0008219//cell death;GO:0051179//localization;GO:0043170//macromolecule metabolic process;GO:0051234//establishment of localization;GO:0009056//catabolic process;GO:0009987//cellular process;GO:0033036//macromolecule localization;GO:0051716//cellular response to stimulus;GO:0044267//cellular protein metabolic process;GO:0043412//macromolecule modification;GO:0050789//regulation of biological process;GO:0065007//biological regulation;GO:0008104//protein localization;GO:0045184//establishment of protein localization;GO:0044699//single-organism process;GO:0006464//cellular protein modification process;GO:0007165//signal transduction;GO:0008150//biological_process;GO:0015031//protein transport;GO:0006810//transport;GO:0044260//cellular macromolecule metabolic process;GO:0036211//protein modification process;GO:0006950//response to stress;GO:0050896//response to stimulus;GO:0044237//cell [illegible]

id	CON_Mean	KD_Mean	AllMean	log2FoldChange	pvalue	qvalue	State	gene_id	XF_1_count	XF_1_FPKM	XF_1_TPM	XF_1_CPM	XF_2_count	XF_2_FPKM	XF_2_TPM	XF_2_CPM	XF_3_count	XF_3_FPKM	XF_3_TPM	XF_3_CPM	Symbol	Description	KEGG_A_class	KEGG_B_class	Pathway	K_ID	GO Component	GO Function	GO Process
HCFC1R1	6.246499353	4.964276443	5.605387898	-1.28329	5.41479E-06	0.001307707	Down	ENSG00000103145	2534	53.49272733	201.97314306	76.3876146 32	881	20.5543927 55	82.5490117 11	29.3516728 54	955	22.6823165 18	91.2486926	32.3903479 88	HCFC1R1	host cell factor C1 regulator 1 [Source:HGNC Symbol;Acc:HGNC:21198]	-	-	-	-	GO:0043233//organelle lumen;GO:0005654//nucleoplasm;GO:0005575//cellular_component;GO:0043229//intracellular organelle;GO:0005634//nucleus;GO:0044446//intracellular organelle part;GO:0043227//membrane-bounded organelle;GO:0043231//intracellular membrane-bounded organelle;GO:0005622//intracellular;GO:0043226//organelle;GO:0044424//intracellular part;GO:0044464//cell part;GO:0005737//cytoplasm;GO:0005623//cell;GO:0044428//nuclear part;GO:0031981//nuclear lumen;GO:0044422//organelle part;GO:0031974//membrane-enclosed lumen;GO:0070013//intracellular organelle lumen	-	-
S100P	0.850481443	-1.83891	-0.49421	-3.16399	5.44175E-06	0.001307707	Down	ENSG00000163993	57	3.648129837	13.7742883 22	1.718269153	4	0.282941098	1.136326831	0.133265257	7	0.504068287	2.027816345	0.237416163	S100P	S100 calcium binding protein P [Source:HGNC Symbol;Acc:HGNC:10504]	-	-	-	-	GO:0043227//membrane-bounded organelle;GO:0031974//membrane-enclosed lumen;GO:0044428//nuclear part;GO:0005886//plasma membrane;GO:0031982//vesicle;GO:0043233//organelle lumen;GO:0005634//nucleus;GO:0044464//cell part;GO:0070013//intracellular organelle lumen;GO:0043226//organelle;GO:0005576//extracellular region;GO:0005654//nucleoplasm;GO:0044422//organelle part;GO:0043231//intracellular membrane-bounded organelle;GO:0044421//extracellular region part;GO:0005737//cytoplasm;GO:0043229//intracellular organelle;GO:0097708//intracellular vesicle;GO:0031410//cytoplasmic vesicle;GO:0044424//intracellular part;GO:0005623//cell;GO:0071944//cell periphery;GO:0031981//nuclear lumen;GO:0005622//intracellular;GO:0005575//cellular_component;GO:0016020//membrane;GO:0044444//cytoplasmic part;GO:0044446//intracellular organelle part;GO:0005615//extracellular space	GO:0005488//binding;GO:0003674//molecular_function;GO:0043167//ion binding	GO:0040011//locomotion;GO:0048870//cell motility;GO:0044699//single-organism process;GO:0044763//single-organism cellular process;GO:0009987//cellular process;GO:0051179//localization;GO:0006928//movement of cell or subcellular component;GO:0002376//immune system process;GO:0016192//vesicle-mediated transport;GO:0051674//localization of cell;GO:0006810//transport;GO:0008150//biological_process;GO:0051234//establishment of localization

id	CON_Mean	KD_Mean	AllMean	log2FoldChange	pvalue	qvalue	State	gene_id	XF_1_count	XF_1_FPKM	XF_1_TPM	XF_1_CPM	XF_2_count	XF_2_FPKM	XF_2_TPM	XF_2_CPM	XF_3_count	XF_3_FPKM	XF_3_TPM	XF_3_CPM	Symbol	Description	KEGG_A_class	KEGG_B_class	Pathway	K_ID	GO Component	GO Function	GO Process
SEL1L3	6.567374451	5.259624318	5.913499384	-1.2932	5.57787E-06	0.001318054	Down	ENSG00000091490	3166	12.754149496	48.155997819	95.439300688	972	4.327603561	17.380197087	32.38345745	1305	5.914894581	23.795029796	44.261156152	SEL1L3	SEL1L family member 3 [Source:HGNC Symbol;Acc:HGNC:29108]	-	-	-	-	GO:0044428//nuclear part;GO:0031974//membrane-enclosed lumen;GO:0043227//membrane-bounded organelle;GO:0005622//intracellular;GO:0043226//organelle;GO:0044446//intracellular organelle part;GO:0070013//intracellular organelle lumen;GO:0043233//organelle lumen;GO:0005634//nucleus;GO:0044422//organelle part;GO:0044424//intracellular part;GO:0031981//nuclear lumen;GO:0005623//cell;GO:0043231//intracellular membrane-bounded organelle;GO:0005654//nucleoplasm;GO:0005575//cellular_component;GO:0044464//cell part;GO:0043229//intracellular organelle	GO:0003674//molecular_function	-
FDCSP	1.384630096	-1.19526	0.09468586	-2.87442	5.75351E-06	0.001318054	Down	ENSG00000181617	84	4.936035322	18.637048773	2.53218612	10	0.649440824	2.608235563	0.333163142	10	0.661142198	2.659708991	0.339165948	FDCSP	follicular dendritic cell secreted protein [Source:HGNC Symbol;Acc:HGNC:19215]	-	-	-	-	GO:0005575//cellular_component;GO:0005576//extracellular region	-	-
SNAPC4	5.288667984	6.564726329	5.926697156	1.279397103	5.7574E-06	0.001318054	Up	ENSG00000165684	1303	5.234412305	19.763634387	39.27903	2924	12.981996651	52.137321998	97.416902865	2657	12.009114107	48.311465922	90.116392257	SNAPC4	small nuclear RNA activating complex polypeptide 4 [Source:HGNC Symbol;Acc:HGNC:11137]	-	-	-	-	GO:0005634//nucleus;GO:0044464//cell part;GO:0044422//organelle part;GO:0043226//organelle;GO:0044446//intracellular organelle part;GO:0043229//intracellular organelle;GO:0005622//intracellular;GO:0043231//intracellular membrane-bounded organelle;GO:0031974//membrane-enclosed lumen;GO:0070013//intracellular organelle lumen;GO:0044424//intracellular part;GO:0032991//macromolecular complex;GO:0043227//membrane-bounded organelle;GO:0031981//nuclear lumen;GO:0043233//organelle lumen;GO:0005654//nucleoplasm;GO:0005575//cellular_component;GO:0044428//nuclear part;GO:0005623//cell	GO:0003674//molecular_function;GO:0003677//DNA binding;GO:0003676//nucleic acid binding;GO:0001071//nucleic acid binding transcription factor activity;GO:0003700//transcription factor activity, sequence-specific DNA binding;GO:0005488//binding;GO:1901363//heterocyclic compound binding;GO:0097159//organic cyclic compound binding	GO:0044237//cellular metabolic process;GO:0009058//biosynthetic process;GO:0008150//biological_process;GO:0006807//nitrogen compound metabolic process;GO:0008152//metabolic process;GO:0034641//cellular nitrogen compound metabolic process;GO:0009987//cellular process

id	CON_Mean	KD_Mean	AllMean	log2FoldChange	pvalue	qvalue	State	gene_id	XF_1_count	XF_1_FPKM	XF_1_TPM	XF_1_CPM	XF_2_count	XF_2_FPKM	XF_2_TPM	XF_2_CPM	XF_3_count	XF_3_FPKM	XF_3_TPM	XF_3_CPM	Symbol	Description	KEGG_A_class	KEGG_B_class	Pathway	K_ID	GO Component	GO Function	GO Process
SRRT	6.835766186	8.173061435	7.50441381	1.351411039	5.79381E-06	0.001318054	Up	ENSG00000087087	3814	23.666798661	89.3590320376	114.973307895	9800	67.208703097	269.91855635	326.499879645	7380	51.524180583	207.276629424	250.304469271	SRRT	serrate, RNA effector molecule [Source:HGNC Symbol;Acc:HGNC:24101]	-	-	-	-	GO:0005654//nucleoplasm;GO:0005575//cellular_component;GO:0043226//organelle;GO:0032991//macromolecular complex;GO:0043229//intracellular organelle;GO:0031974//membrane-enclosed lumen;GO:0031981//nuclear lumen;GO:0044422//organelle part;GO:0005737//cytoplasm;GO:0044464//cell part;GO:0005623//cell;GO:0043227//membrane-bounded organelle;GO:0043231//intracellular membrane-bounded organelle;GO:0043233//organelle lumen;GO:0044428//nuclear part;GO:0005634//nucleus;GO:0070013//intracellular organelle lumen;GO:0044446//intracellular organelle part;GO:0005622//intracellular;GO:0044424//intracellular part	GO:0003723//RNA binding;GO:0097159//organic cyclic compound binding;GO:0003674//molecular_function;GO:0005488//binding;GO:1901363//heterocyclic compound binding;GO:0003676//nucleic acid binding;GO:0003677//DNA binding	GO:0030154//cell differentiation;GO:0009058//biosynthetic process;GO:0044767//single-organism developmental process;GO:0006397//mRNA processing;GO:0044763//single-organism cellular process;GO:0008150//biological_process;GO:0032502//developmental process;GO:0016070//RNA metabolic process;GO:0006807//nitrogen compound metabolic process;GO:0044260//cellular macromolecule metabolic process;GO:0048869//cellular developmental process;GO:0010467//gene expression;GO:0006396//RNA processing;GO:0009987//cellular process;GO:0044238//primary metabolic process;GO:0043170//macromolecule metabolic process;GO:0034641//cellular nitrogen compound metabolic process;GO:0016071//mRNA metabolic process;GO:0008152//metabolic process;GO:0006725//cellular aromatic compound metabolic process;GO:0044237//cellular metabolic process;GO:0090304//nucleic acid metabolic process;GO:0071704//organic substance metabolic process;GO:0006139//nucleobase-containing
STON1	4.441780495	1.85820634	3.149993417	-2.49895	6.30395E-06	0.001415237	Down	ENSG00000243244	723	2.827200373	10.674694934	21.794887679	69	0.298200244	1.197609472	2.298825683	156	0.686339186	2.761073954	5.290988781	STON1	stonin 1 [Source:HGNC Symbol;Acc:HGNC:17003]	-	-	-	-	GO:0044424//intracellular part;GO:0005737//cytoplasm;GO:0005623//cell;GO:0005622//intracellular;GO:0044464//cell part;GO:0005575//cellular_component	-	GO:0051179//localization;GO:0016192//vesicle-mediated transport;GO:0006810//transport;GO:0051234//establishment of localization;GO:0008150//biological_process

id	CON_Mean	KD_Mean	AllMean	log2FoldChange	pvalue	qvalue	State	gene_id	XF_1_count	XF_1_FPKM	XF_1_TPM	XF_1_CPM	XF_2_count	XF_2_FPKM	XF_2_TPM	XF_2_CPM	XF_3_count	XF_3_FPKM	XF_3_TPM	XF_3_CPM	Symbol	Description	KEGG_A_class	KEGG_B_class	Pathway	K_ID	GO Component	GO Function	GO Process
NIPSNAP1	6.969579867	5.572132741	6.270856304	-1.36129	6.4936E-06	0.001438882	Down	ENSG00000184117	4185	42.292031487	159.682539136	126.157129926	1117	12.475468661	50.10304225	37.214323017	1753	19.931542273	80.182602709	59.455790601	NIPSNAP1	nipsnap homolog 1 [Source:HGNC Symbol;Acc:HGNC:7827]	-	-	-	-	GO:0043229//intracellular organelle;GO:0016020//membrane;GO:0043231//intracellular membrane-bounded organelle;GO:0044424//intracellular part;GO:0005622//intracellular;GO:0005739//mitochondrion;GO:0044464//cell part;GO:0005623//cell;GO:0043227//membrane-bounded organelle;GO:0005886//plasma membrane;GO:0071944//cell periphery;GO:0005737//cytoplasm;GO:0043226//organelle;GO:0044444//cytoplasmic part;GO:0005575//cellular_component	GO:0003674//molecular_function	GO:0050877//neurological system process;GO:0003008//system process;GO:0032501//multicellular organismal process;GO:0008150//biological_process
OIP5-AS1	7.331977237	6.183680615	6.757828926	-1.14699	6.66413E-06	0.001457735	Down	ENSG00000247556	5381	12.799703075	48.327995022	162.210637069	2032	5.341967218	21.454008382	67.698750555	2253	6.029676318	24.256785252	76.414087977	-	-	-	-	-	-	-	-	-
GSEC	4.80357515	2.921597565	3.862586357	-1.89458	7.38153E-06	0.001594223	Down	ENSG00000280832	930	7.530195477	28.431850911	28.034917761	216	1.932936846	7.762916095	7.196323878	225	2.049753913	8.245955151	7.631233819	-	-	-	-	-	-	-	-	-
VAT1	7.947843964	6.505058894	7.226451429	-1.39525	7.47919E-06	0.001595125	Down	ENSG00000108828	8248	71.735880255	270.854038065	248.636560963	2071	19.907122565	79.949493685	68.998086811	3453	33.789383058	135.931311306	117.114001679	VAT1	vesicle amine transport 1 [Source:HGNC Symbol;Acc:HGNC:16919]	-	-	-	-	GO:0043231//intracellular membrane-bounded organelle;GO:0044421//extracellular region part;GO:0043226//organelle;GO:0044464//cell part;GO:0005739//mitochondrion;GO:0097708//intracellular vesicle;GO:0005615//extracellular space;GO:0043229//intracellular organelle;GO:0005773//vacuole;GO:0005737//cytoplasm;GO:0043227//membrane-bounded organelle;GO:0000323//lytic vacuole;GO:0005764//lysosome;GO:0005622//intracellular;GO:0044444//cytoplasmic part;GO:0044424//intracellular part;GO:0031410//cytoplasmic vesicle;GO:0005576//extracellular region;GO:0005623//cell;GO:0031982//vesicle;GO:0005575//cellular_component	GO:0005488//binding;GO:0043167//ion binding;GO:0016491//oxidoreductase activity;GO:0003674//molecular_function;GO:0003824//catalytic activity	GO:0002376//immune system process;GO:0016192//vesicle-mediated transport;GO:0006810//transport;GO:0016043//cellular component organization;GO:0051179//localization;GO:0008150//biological_process;GO:0006996//organelle organization;GO:0048856//anatomical structure development;GO:0051234//establishment of localization;GO:0032502//developmental process;GO:0071840//cellular component organization or biogenesis;GO:0009987//cellular process;GO:0007005//mitochondrion organization
ENSG00000273119	0.156850883	-2.51577	-1.17946	-3.5236	8.56644E-06	0.001804452	Down	ENSG00000273119	34	1.470491359	5.552152161	1.024932477	4	0.19119836	0.767876524	0.133265257	1	0.048660825	0.195757634	0.033916595	-	-	-	-	-	-	-	-	-

id	CON_Mean	KD_Mean	AllMean	log2FoldChange	pvalue	qvalue	State	gene_id	XF_1_count	XF_1_FPKM	XF_1_TPM	XF_1_CPM	XF_2_count	XF_2_FPKM	XF_2_TPM	XF_2_CPM	XF_3_count	XF_3_FPKM	XF_3_TPM	XF_3_CPM	Symbol	Description	KEGG_A_class	KEGG_B_class	Pathway	K_ID	GO Component	GO Function	GO Process
FSTL3	5.950249286	4.586600836	5.268425061	-1.36206	8.95948E-06	0.001864228	Down	ENSG00000070404	2063	20.504215402	77.418016189	62.189285314	652	7.161964026	28.76334316	21.722236891	763	8.532265676	34.324452145	25.878361796	FSTL3	follistatin like 3 [Source:HGNC Symbol;Acc:HGNC:3973]	-	-	-	-	GO:0044421//extracellular region part;GO:0005737//cytoplasm;GO:0031410//cytoplasmic vesicle;GO:0044424//intracellular part;GO:0044464//cell part;GO:0031974//membrane-enclosed lumen;GO:0097708//intracellular vesicle;GO:0005634//nucleus;GO:0043226//organelle;GO:0005654//nucleoplasm;GO:0005783//endoplasmic reticulum;GO:0031981//nuclear lumen;GO:0070013//intracellular organelle lumen;GO:0043231//intracellular membrane-bounded organelle;GO:0043227//membrane-bounded organelle;GO:0005622//intracellular;GO:0005623//cell;GO:0043233//organelle lumen;GO:0005615//extracellular space;GO:0005794//Golgi apparatus;GO:0005575//cellular_component;GO:0044422//organelle part;GO:0044428//nuclear part;GO:0043229//intracellular organelle;GO:0044444//cytoplasmic part;GO:0031982//vesicle;GO:0012505//endomembrane system;GO:0044446//intracellular organelle part;GO:0005576//extracellular region	GO:0003674//molecular_function	GO:0044767//single-organism developmental process;GO:0044700//single organism signaling;GO:0044238//primary metabolic process;GO:0009058//biosynthetic process;GO:0065007//biological regulation;GO:0007154//cell communication;GO:0044260//cellular macromolecule metabolic process;GO:0043170//macromolecule metabolic process;GO:0050789//regulation of biological process;GO:0022610//biological adhesion;GO:0007165//signal transduction;GO:0044763//single-organism cellular process;GO:0002376//immune system process;GO:0048869//cellular developmental process;GO:0023052//signaling;GO:0048856//anatomical structure development;GO:0044237//cellular metabolic process;GO:0032502//developmental process;GO:0007155//cell adhesion;GO:0000003//reproduction;GO:0034641//cellular nitrogen compound metabolic process;GO:0050896//response to stimulus;GO:0044699//single-organism process;GO:0071704//organic substance metabolic process;GO:0044267//cellu[illegible]
ENSG00000260329	1.132323017	-1.37532	-0.1215	-2.84311	9.20933E-06	0.001893127	Down	ENSG00000260329	70	1.436456842	5.423647618	2.1101551	8	0.181436701	0.728672481	0.266530514	9	0.207793977	0.835934405	0.305249353	-	-	-	-	-	-	-	-	-

id	CON_Mean	KD_Mean	AllMean	log2FoldChange	pvalue	qvalue	State	gene_id	XF_1_count	XF_1_FPKM	XF_1_TPM	XF_1_CPM	XF_2_count	XF_2_FPKM	XF_2_TPM	XF_2_CPM	XF_3_count	XF_3_FPKM	XF_3_TPM	XF_3_CPM	Symbol	Description	KEGG_A_class	KEGG_B_class	Pathway	K_ID	GO Component	GO Function	GO Process
SPOCK1	6.181367306	4.925187376	5.553277341	-1.25618	1.07046E-05	0.002174307	Down	ENSG00000152377	2422	11.77602685	44.462888214	73.011366471	845	4.540691216	18.235983756	28.1522855541	943	5.158604653	20.7525509996	31.9833488851	SPOCK1	SPARC (osteonectin), cwcv and kazal like domains proteoglycan 1 [Source:HGNC Symbol;Acc:HGNC:11251]	-	-	-	-	GO:0005622//intracellular;GO:0005615//extracellular space;GO:0005576//extracellular region;GO:0044464//cell part;GO:0044421//extracellular region part;GO:0044424//intracellular part;GO:0005575//cellular_component;GO:0043226//organelle;GO:0005623//cell;GO:0005737//cytoplasm	GO:0005488//binding;GO:0043167//ion binding;GO:0098772//molecular function regulator;GO:0003674//molecular_function;GO:0030234//enzyme regulator activity	GO:0040011//locomotion;GO:0032502//developmental process;GO:0007155//cell adhesion;GO:0044699//single-organism process;GO:0048869//cellular developmental process;GO:0022610//biological adhesion;GO:0009987//cellular process;GO:0044763//single-organism cellular process;GO:0044767//single-organism developmental process;GO:0051674//localization of cell;GO:0030154//cell differentiation;GO:0051179//localization;GO:0040007//growth;GO:0048856//anatomical structure development;GO:0008150//biological_process;GO:0048870//cell motility;GO:0006928//movement of cell or subcellular component

id	CON_Mean	KD_Mean	AllMean	log2FoldChange	pvalue	qvalue	State	gene_id	XF_1_count	XF_1_FPKM	XF_1_TPM	XF_1_CPM	XF_2_count	XF_2_FPKM	XF_2_TPM	XF_2_CPM	XF_3_count	XF_3_FPKM	XF_3_TPM	XF_3_CPM	Symbol	Description	KEGG_A_class	KEGG_B_class	Pathway	K_ID	GO Component	GO Function	GO Process
HR	6.309906241	7.512663754	6.911284997	1.203899373	1.12009E-05	0.002248359	Up	ENSG00000168453	2648	10.0231231 71	37.844428414	79.824152938	5323	22.268048813	89.431268742	177.34274075	5437	23.154762138	93.149294114	184.404525668	HR	HR lysine demethylase and nuclear receptor corepressor [Source:HGNC Symbol;Acc:HGNC:5172]	-	-	-	-	GO:0044424//intracellular part;GO:0005654//nucleoplasm;GO:0044422//organelle part;GO:0070013//intracellular organelle lumen;GO:0043229//intracellular organelle;GO:0043228//non-membrane-bounded organelle;GO:0005694//chromosome;GO:0031981//nuclear lumen;GO:0043226//organelle;GO:0005623//cell;GO:0044464//cell part;GO:0043232//intracellular non-membrane-bounded organelle;GO:0043231//intracellular membrane-bounded organelle;GO:0031974//membrane-enclosed lumen;GO:0043227//membrane-bounded organelle;GO:0005575//cellular_component;GO:0043233//organelle lumen;GO:0005622//intracellular;GO:0044446//intracellular organelle part;GO:0044428//nuclear part;GO:0005634//nucleus	GO:0003677//DNA binding;GO:0003676//nucleic acid binding;GO:0016491//oxidoreductase activity;GO:1901363//heterocyclic compound binding;GO:0003824//catalytic activity;GO:0097159//organic cyclic compound binding;GO:0043167//ion binding;GO:0003674//molecular_function;GO:0005488//binding	GO:0009987//cellular process;GO:0051276//chromosome organization;GO:0044237//cellular metabolic process;GO:0006996//organelle organization;GO:0044267//cellular protein metabolic process;GO:0006464//cellular protein modification process;GO:0009058//biosynthetic process;GO:0034641//cellular nitrogen compound metabolic process;GO:0008152//metabolic process;GO:0016043//cellular component organization;GO:0044238//primary metabolic process;GO:0043412//macromolecule modification;GO:0036211//protein modification process;GO:0043170//macromolecule metabolic process;GO:0071840//cellular component organization or biogenesis;GO:0019538//protein metabolic process;GO:0006807//nitrogen compound metabolic process;GO:0044260//cellular macromolecule metabolic process;GO:0071704//organic substance metabolic process;GO:0008150//biological_process

id	CON_Mean	KD_Mean	AllMean	log2FoldChange	pvalue	qvalue	State	gene_id	XF_1_count	XF_1_FPKM	XF_1_TPM	XF_1_CPM	XF_2_count	XF_2_FPKM	XF_2_TPM	XF_2_CPM	XF_3_count	XF_3_FPKM	XF_3_TPM	XF_3_CPM	Symbol	Description	KEGG_A_class	KEGG_B_class	Pathway	K_ID	GO Component	GO Function	GO Process
PLXNA2	2.13962956	4.30272348	3.22117652	2.18741229	1.13649E-05	0.002251207	Up	ENSG00000076356	144	0.313534886	1.183817492	4.340890492	593	1.426982618	5.730940645	19.75657435	565	1.384100833	5.568099332	19.162876035	PLXNA2	plexin A2 [Source:HGNC Symbol;Acc:HGNC:9100]	Organismal Systems	Development	ko04360//Axon guidance	K06820	GO:0044464//cell part;GO:0032991//macromolecular complex;GO:0016020//membrane;GO:0005623//cell;GO:0005886//plasma membrane;GO:0071944//cell periphery;GO:0005575//cellular_component	GO:0003674//molecular_function	GO:0071840//cellular component organization or biogenesis;GO:0032989//cellular component morphogenesis;GO:0050794//regulation of cellular process;GO:0007155//cell adhesion;GO:0000902//cell morphogenesis;GO:0048646//anatomical structure formation involved in morphogenesis;GO:0016043//cellular component organization;GO:0007165//signal transduction;GO:0051179//localization;GO:0009653//anatomical structure morphogenesis;GO:0044767//single-organism developmental process;GO:0050896//response to stimulus;GO:0040011//locomotion;GO:0023052//signaling;GO:0007275//multicellular organism development;GO:0048870//cell motility;GO:0044707//single-multicellular organism process;GO:0044700//single organism signaling;GO:0009790//embryo development;GO:0051674//localization of cell;GO:0032501//multicellular organismal process;GO:0044763//single-organism cellular process;GO:0051716//cellular response to stimulus;GO:0048869//cellular developmental process;GO:0006928//movement of cell or subcellular component;GO:0008150//bi[illegible]

id	CON_Mean	KD_Mean	AllMean	log2FoldChange	pvalue	qvalue	State	gene_id	XF_1_count	XF_1_FPKM	XF_1_TPM	XF_1_CPM	XF_2_count	XF_2_FPKM	XF_2_TPM	XF_2_CPM	XF_3_count	XF_3_FPKM	XF_3_TPM	XF_3_CPM	Symbol	Description	KEGG_A_class	KEGG_B_class	Pathway	K_ID	GO Component	GO Function	GO Process
MT-ND6	8.435454401	9.668547349	9.052000875	1.236066008	1.14952E-05	0.002251207	Up	ENSG00000198695	11566	664.11030994	2507.489397614	348.657912718	25647	1627.549545821	6536.442507484	854.463511556	22429	1448.981530851	5829.107894872	760.715303697	MT-ND6	mitochondrially encoded NADH:ubiquinone oxidoreductase core subunit 6 [Source:HGNC Symbol;Acc:HGNC:7462]	Human Diseases;Metabolism	Energy metabolism; Neurodegenerative diseases	ko05012//Parkinson's disease; ko00190//Oxidative phosphorylation	K03884	GO:0005575//cellular_component;GO:0005737//cytoplasm;GO:0043227//membrane-bounded organelle;GO:0043231//intracellular membrane-bounded organelle;GO:0044444//cytoplasmic part;GO:0044464//cell part;GO:0044424//intracellular part;GO:0005622//intracellular;GO:0005739//mitochondrion;GO:0005623//cell;GO:0043226//organelle;GO:0043229//intracellular organelle	GO:0016491//oxidoreductase activity;GO:0003824//catalytic activity;GO:0003674//molecular_function	GO:0009987//cellular process;GO:0043933//macromolecular complex subunit organization;GO:0008152//metabolic process;GO:0044085//cellular component biogenesis;GO:0071840//cellular component organization or biogenesis;GO:0016043//cellular component organization;GO:0006996//organelle organization;GO:0022607//cellular component assembly;GO:0065003//macromolecular complex assembly;GO:0007005//mitochondrion organization;GO:0008150//biological_process;GO:0006950//response to stress;GO:0044237//cellular metabolic process;GO:0006091//generation of precursor metabolites and energy;GO:0050896//response to stimulus
MROH6	2.910460762	4.876779071	3.893619917	2.022997371	1.1611E-05	0.002251207	Up	ENSG00000204839	248	1.778301158	6.714353374	7.47597807	1110	8.796648149	35.32843902	36.981108817	671	5.413424139	21.77766432	22.758035079	-	-	-	-	-	-	-	-	-
NDOR1	4.831135917	6.144822049	5.487978983	1.322253116	1.23022E-05	0.002358421	Up	ENSG00000188566	948	5.508390338	20.798096586	28.577529073	2289	14.699507193	59.035059118	76.261043317	1895	12.388578846	49.838014649	64.271947055	NDOR1	NADPH dependent diflavin oxidoreductase 1 [Source:HGNC Symbol;Acc:HGNC:29838]	-	-	-	-	GO:0005737//cytoplasm;GO:0005623//cell;GO:0044464//cell part;GO:0043226//organelle;GO:0044422//organelle part;GO:0044446//intracellular organelle part;GO:0031981//nuclear lumen;GO:0005575//cellular_component;GO:0044444//cytoplasmic part;GO:0043228//non-membrane-bounded organelle;GO:0005634//nucleus;GO:0070013//intracellular organelle lumen;GO:0043229//intracellular organelle;GO:0005622//intracellular;GO:0005829//cytosol;GO:0043231//intracellular membrane-bounded organelle;GO:0005654//nucleoplasm;GO:0031974//membrane-enclosed lumen;GO:0044428//nuclear part;GO:0005856//cytoskeleton;GO:0043233//organelle lumen;GO:0044424//intracellular part;GO:0043232//intracellular non-membrane-bounded organelle;GO:0043227//membrane-bounded organelle	GO:0016491//oxidoreductase activity;GO:0003824//catalytic activity;GO:0043167//ion binding;GO:0003674//molecular_function;GO:0005488//binding	GO:0022607//cellular component assembly;GO:0044237//cellular metabolic process;GO:0006790//sulfur compound metabolic process;GO:0044763//single-organism cellular process;GO:0016043//cellular component organization;GO:0051186//cofactor metabolic process;GO:0008219//cell death;GO:0008152//metabolic process;GO:0009987//cellular process;GO:0044085//cellular component biogenesis;GO:0009058//biosynthetic process;GO:0071840//cellular component organization or biogenesis;GO:0008150//biological_process;GO:0044699//single-organism process

id	CON_Mean	KD_Mean	AllMean	log2FoldChange	pvalue	qvalue	State	gene_id	XF_1_count	XF_1_FPKM	XF_1_TPM	XF_1_CPM	XF_2_count	XF_2_FPKM	XF_2_TPM	XF_2_CPM	XF_3_count	XF_3_FPKM	XF_3_TPM	XF_3_CPM	Symbol	Description	KEGG_A_class	KEGG_B_class	Pathway	K_ID	GO Component	GO Function	GO Process
SLC39A10	6.476244062	5.323663903	5.899953982	-1.15354	1.39945E-05	0.002653047	Down	ENSG00000196950	2972	12.952314088	48.904210285	89.591156545	1226	5.905132466	23.715750445	40.845801227	1131	5.545708929	22.309832813	38.359668665	SLC39A10	solute carrier family 39 member 10 [Source:HGNC Symbol;Acc:HGNC:20861]	-	-	-	-	GO:0005623//cell;GO:0005886//plasma membrane;GO:0005575//cellular_component;GO:0044464//cell part;GO:0071944//cell periphery;GO:0016020//membrane	GO:0005215//transporter activity;GO:0003674//molecular_function;GO:0022857//transmembrane transporter activity	GO:0008283//cell proliferation;GO:0044238//primary metabolic process;GO:0044699//single-organism process;GO:0050794//regulation of cellular process;GO:0044237//cellular metabolic process;GO:0051179//localization;GO:0044267//cellular protein metabolic process;GO:0007154//cell communication;GO:0050789//regulation of biological process;GO:0036211//protein modification process;GO:0008219//cell death;GO:0044763//single-organism cellular process;GO:0042592//homeostatic process;GO:0006464//cellular protein modification process;GO:0023052//signaling;GO:0006810//transport;GO:0055085//transmembrane transport;GO:0051234//establishment of localization;GO:0071704//organic substance metabolic process;GO:0065007//biological regulation;GO:0050896//response to stimulus;GO:0002376//immune system process;GO:0043412//macromolecule modification;GO:0007165//signal transduction;GO:0043170//macromolecule metabolic process;GO:0044260//cellular macromolecule metabolic process;GO:0019538//prot[illegible]

id	CON_Mean	KD_Mean	AllMean	log2FoldChange	pvalue	qvalue	State	gene_id	XF_1_count	XF_1_FPKM	XF_1_TPM	XF_1_CPM	XF_2_count	XF_2_FPKM	XF_2_TPM	XF_2_CPM	XF_3_count	XF_3_FPKM	XF_3_TPM	XF_3_CPM	Symbol	Description	KEGG_A_class	KEGG_B_class	Pathway	K_ID	GO Component	GO Function	GO Process
C2CD2L	4.319542709	5.71257449	5.0160586	1.397695684	1.42681E-05	0.002675198	Up	ENSG00000172375	664	2.171910632	8.200509463	20.01632838	1591	5.751546872	23.0989314 91	53.0062559 71	1496	5.50555835	22.1483110 43	50.7392257 49	C2CD2L	C2CD2 like [Source:HGNC Symbol;Acc:HGNC:29000]	-	-	-	-	GO:0005622//intracellular;GO:0016020//membrane;GO:0044464//cell part;GO:0005623//cell;GO:0005783//endoplasmic reticulum;GO:0044444//cytoplasmic part;GO:0005737//cytoplasm;GO:0012505//endomembrane system;GO:0044424//intracellular part;GO:0043226//organelle;GO:0071944//cell periphery;GO:0043231//intracellular membrane-bounded organelle;GO:0005886//plasma membrane;GO:0043229//intracellular organelle;GO:0005575//cellular_component;GO:0043227//membrane-bounded organelle	GO:0043167//ion binding;GO:0003674//molecular_function;GO:0008289//lipid binding;GO:0005488//binding	GO:0007267//cell-cell signaling;GO:0015031//protein transport;GO:0045184//establishment of protein localization;GO:0008104//protein localization;GO:0009987//cellular process;GO:0033036//macromolecule localization;GO:0044763//single-organism cellular process;GO:0008150//biological_process;GO:0051234//establishment of localization;GO:0051179//localization;GO:0007154//cell communication;GO:0044699//single-organism process;GO:0042592//homeostatic process;GO:0044700//single organism signaling;GO:0065007//biological regulation;GO:0006810//transport;GO:0065008//regulation of biological quality;GO:0071702//organic substance transport;GO:0023052//signaling
ENSG00000286443	0.156850883	-2.32758	-1.08537	-3.27069	1.4835E-05	0.002696166	Down	ENSG00000286443	34	0.875262577	3.304739587	1.024932477	3	0.085353495	0.34279031	0.099948943	3	0.086891361	0.349555264	0.101749784	-	-	-	-	-	-	-	-	-
ZNF467	2.539262051	-0.40149	1.068885543	-2.9272	1.48621E-05	0.002696166	Down	ENSG00000181444	191	2.025222975	7.646659087	5.757708917	11	0.128905894	0.517702191	0.366479457	33	0.393685412	1.58375707	1.119247627	ZNF467	zinc finger protein 467 [Source:HGNC Symbol;Acc:HGNC:23154]	-	-	-	-	GO:0005623//cell;GO:0043227//membrane-bounded organelle;GO:0044464//cell part;GO:0005575//cellular_component;GO:0043231//intracellular membrane-bounded organelle;GO:0043226//organelle;GO:0005634//nucleus;GO:0044424//intracellular part;GO:0043229//intracellular organelle;GO:0005622//intracellular	GO:0005488//binding;GO:1901363//heterocyclic compound binding;GO:0003674//molecular_function;GO:0003677//DNA binding;GO:0097159//organic cyclic compound binding;GO:0043167//ion binding;GO:0003676//nucleic acid binding	GO:0008152//metabolic process;GO:0044237//cellular metabolic process;GO:0034641//cellular nitrogen compound metabolic process;GO:0006807//nitrogen compound metabolic process;GO:0009987//cellular process;GO:0008150//biological_process;GO:0009058//biosynthetic process

id	CON_Mean	KD_Mean	AllMean	log2FoldChange	pvalue	qvalue	State	gene_id	XF_1_count	XF_1_FPKM	XF_1_TPM	XF_1_CPM	XF_2_count	XF_2_FPKM	XF_2_TPM	XF_2_CPM	XF_3_count	XF_3_FPKM	XF_3_TPM	XF_3_CPM	Symbol	Description	KEGG_A_class	KEGG_B_class	Pathway	K_ID	GO Component	GO Function	GO Process
SRRM2	8.922065182	10.11867133	9.520368256	1.196762821	1.49161E-05	0.002696166	Up	ENSG00000167978	16207	37.6714624	142.236600076	488.56119587	32928	84.5893743 24	339.721505518	1097.039595607	32607	85.273992219	343.048748853	1105.918405084	SRRM2	serine/arginine repetitive matrix 2 [Source:HGNC Symbol;Acc:HGNC:16639]	-	-	-	-	GO:0044428//nuclear part;GO:0043226//organelle;GO:0005622//intracellular;GO:0044446//intracellular organelle part;GO:0043227//membrane-bounded organelle;GO:0032991//macromolecular complex;GO:0031974//membrane-enclosed lumen;GO:0044422//organelle part;GO:0031981//nuclear lumen;GO:0005634//nucleus;GO:0005623//cell;GO:0005575//cellular_component;GO:0043233//organelle lumen;GO:0044424//intracellular part;GO:0070013//intracellular organelle lumen;GO:0005654//nucleoplasm;GO:0043231//intracellular membrane-bounded organelle;GO:0043229//intracellular organelle;GO:0044464//cell part	GO:0003674//molecular_function;GO:0003723//RNA binding;GO:1901363//heterocyclic compound binding;GO:0005488//binding;GO:0044822//poly(A) RNA binding;GO:0097159//organic cyclic compound binding;GO:0003676//nucleic acid binding;GO:0003729//mRNA binding	GO:0090304//nucleic acid metabolic process;GO:0006139//nucleobase-containing compound metabolic process;GO:1901360//organic cyclic compound metabolic process;GO:0044237//cellular metabolic process;GO:0071704//organic substance metabolic process;GO:0006397//mRNA processing;GO:0010467//gene expression;GO:0046483//heterocycle metabolic process;GO:0006807//nitrogen compound metabolic process;GO:0034641//cellular nitrogen compound metabolic process;GO:0008150//biological_process;GO:0006725//cellular aromatic compound metabolic process;GO:0008152//metabolic process;GO:0043170//macromolecule metabolic process;GO:0009987//cellular process;GO:0006396//RNA processing;GO:0044260//cellular macromolecule metabolic process;GO:0044238//primary metabolic process;GO:0016071//mRNA metabolic process;GO:0016070//RNA metabolic process

id	CON_Mean	KD_Mean	AllMean	log2FoldChange	pvalue	qvalue	State	gene_id	XF_1_count	XF_1_FPKM	XF_1_TPM	XF_1_CPM	XF_2_count	XF_2_FPKM	XF_2_TPM	XF_2_CPM	XF_3_count	XF_3_FPKM	XF_3_TPM	XF_3_CPM	Symbol	Description	KEGG_A_class	KEGG_B_class	Pathway	K_ID	GO Component	GO Function	GO Process
KMT2D	7.2022568	8.39994796	7.80110238	1.199824336	1.50121E-05	0.002696166	Up	ENSG00000167548	4918	5.857274242	22.115381798	148.253468334	10491	13.809073241	55.458965013	349.521452791	9443	12.653565811	50.904030726	320.274404245	KMT2D	lysine methyltransferase 2D [Source:HGNC Symbol;Acc:HGNC:7133]	Metabolism	Amino acid metabolism	ko00310//Lysine degradation	K09187	GO:0044446//intracellular organelle part;GO:0032991//macromolecular complex;GO:0031974//membrane-enclosed lumen;GO:0043231//intracellular membrane-bounded organelle;GO:0005634//nucleus;GO:0044428//nuclear part;GO:0043233//organelle lumen;GO:0005623//cell;GO:0044464//cell part;GO:0070013//intracellular organelle lumen;GO:0043227//membrane-bounded organelle;GO:0005622//intracellular;GO:0044424//intracellular part;GO:0043229//intracellular organelle;GO:0044422//organelle part;GO:0031981//nuclear lumen;GO:0043226//organelle;GO:0005654//nucleoplasm;GO:0005575//cellular_component	GO:0003824//catalytic activity;GO:0003677//DNA binding;GO:0005515//protein binding;GO:0003676//nucleic acid binding;GO:0005488//binding;GO:0003674//molecular_function;GO:1901363//heterocyclic compound binding;GO:0042393//histone binding;GO:0016741//transferase activity, transferring one-carbon groups;GO:0016740//transferase activity;GO:0043167//ion binding;GO:0008168//methyltransferase activity;GO:0097159//organic cyclic compound binding	GO:0008152//metabolic process;GO:0044267//cellular protein metabolic process;GO:0023052//signaling;GO:0051276//chromosome organization;GO:0007165//signal transduction;GO:0040007//growth;GO:0016043//cellular component organization;GO:0006807//nitrogen compound metabolic process;GO:0048856//anatomical structure development;GO:0044763//single-organism cellular process;GO:0065007//biological regulation;GO:0019538//protein metabolic process;GO:0006996//organelle organization;GO:0048869//cellular developmental process;GO:0044767//single-organism developmental process;GO:0071704//organic substance metabolic process;GO:0008150//biological_process;GO:0030154//cell differentiation;GO:0022607//cellular component assembly;GO:0043170//macromolecule metabolic process;GO:0044700//single organism signaling;GO:0009058//biosynthetic process;GO:0009987//cellular process;GO:0036211//protein modification process;GO:0032502//developmental process;GO:0065003//macr[illegible]

id	CON_Mean	KD_Mean	AllMean	log2FoldChange	pvalue	qvalue	State	gene_id	XF_1_count	XF_1_FPKM	XF_1_TPM	XF_1_CPM	XF_2_count	XF_2_FPKM	XF_2_TPM	XF_2_CPM	XF_3_count	XF_3_FPKM	XF_3_TPM	XF_3_CPM	Symbol	Description	KEGG_A_class	KEGG_B_class	Pathway	K_ID	GO Component	GO Function	GO Process
SPNS2	1.735186618	4.078773425	2.906980022	2.393733167	1.5962E-05	0.002836918	Up	ENSG00000183018	108	0.800311669	3.02174653	3.255667869	581	4.758303485	19.109941842	19.356778579	422	3.518388148	14.154123909	14.312802985	SPNS2	sphingolipid transporter 2 [Source:HGNC Symbol;Acc:HGNC:26992]	-	-	-	-	GO:0044464//cell part;GO:0043229//intracellular organelle;GO:0005768//endosome;GO:0016020//membrane;GO:0043227//membrane-bounded organelle;GO:0005623//cell;GO:0005773//vacuole;GO:0044424//intracellular part;GO:0043226//organelle;GO:0071944//cell periphery;GO:0031982//vesicle;GO:0043231//intracellular membrane-bounded organelle;GO:0031410//cytoplasmic vesicle;GO:0044444//cytoplasmic part;GO:0005886//plasma membrane;GO:0097708//intracellular vesicle;GO:0012505//endomembrane system;GO:0005622//intracellular;GO:0005575//cellular_component;GO:0005737//cytoplasm	GO:0003674//molecular_function	GO:0002376//immune system process;GO:0008150//biological_process;GO:0006928//movement of cell or subcellular component;GO:0048870//cell motility;GO:0050896//response to stimulus;GO:0040011//locomotion;GO:0006810//transport;GO:0065007//biological regulation;GO:0051179//localization;GO:0008152//metabolic process;GO:0044710//single-organism metabolic process;GO:0048856//anatomical structure development;GO:0007154//cell communication;GO:0044699//single-organism process;GO:0006629//lipid metabolic process;GO:0065008//regulation of biological quality;GO:0007165//signal transduction;GO:0044700//single organism signaling;GO:0023052//signaling;GO:0051674//localization of cell;GO:0032502//developmental process;GO:0051716//cellular response to stimulus;GO:0044238//primary metabolic process;GO:0009987//cellular process;GO:0071704//organic substance metabolic process;GO:0050789//regulation of biological process;GO:0055085//trans

id	CON_Mean	KD_Mean	AllMean	log2FoldChange	pvalue	qvalue	State	gene_id	XF_1_count	XF_1_FPKM	XF_1_TPM	XF_1_CPM	XF_2_count	XF_2_FPKM	XF_2_TPM	XF_2_CPM	XF_3_count	XF_3_FPKM	XF_3_TPM	XF_3_CPM	Symbol	Description	KEGG_A_class	KEGG_B_class	Pathway	K_ID	GO Component	GO Function	GO Process
AMH	2.197246658	4.349535226	3.273390942	2.189831648	1.62111E-05	0.00285149	Up	ENSG00000104899	150	1.74787821	6.599485077	4.521760929	693	8.924702657	35.842721942	23.0882057 75	516	6.764964396	27.214775709	17.500962892	AMH	anti-Mullerian hormone [Source:HGNC Symbol;Acc:HGNC:464]	Environmental Information Processing	Signaling molecules and interaction;Signal transduction	ko04060//Cytokine-cytokine receptor interaction;ko04024//cAMP signaling pathway;ko04390//Hippo signaling pathway;ko04350//TGF-beta signaling pathway	K04665	GO:0005576//extracellular region;GO:0005575//cellular_component;GO:0005615//extracellular space;GO:0044421//extracellular region part	GO:0003674//molecular_function	GO:0050896//response to stimulus;GO:0006807//nitrogen compound metabolic process;GO:0009058//biosynthetic process;GO:0044767//single-organism developmental process;GO:0050794//regulation of cellular process;GO:0044237//cellular metabolic process;GO:0065007//biological regulation;GO:0007165//signal transduction;GO:0008150//biological_process;GO:0030154//cell differentiation;GO:0044700//single organism signaling;GO:0000003//reproduction;GO:0032502//developmental process;GO:0048856//anatomical structure development;GO:0023052//signaling;GO:0009987//cellular process;GO:0008152//metabolic process;GO:0048869//cellular developmental process;GO:0007154//cell communication;GO:0051716//cellular response to stimulus;GO:0007568//aging;GO:0044763//single-organism cellular process;GO:0007267//cell-cell signaling;GO:0050789//regulation of biological process;GO:0044699//single-organism process;GO:0040007//growth;GO:0034641//cellular nitrogen compound metabolic process

id	CON_Mean	KD_Mean	AllMean	log2FoldChange	pvalue	qvalue	State	gene_id	XF_1_count	XF_1_FPKM	XF_1_TPM	XF_1_CPM	XF_2_count	XF_2_FPKM	XF_2_TPM	XF_2_CPM	XF_3_count	XF_3_FPKM	XF_3_TPM	XF_3_CPM	Symbol	Description	KEGG_A_class	KEGG_B_class	Pathway	K_ID	GO Component	GO Function	GO Process
SUCLG2	7.725808652	6.64721944	7.186514046	-1.07928	1.66883E-05	0.002905459	Down	ENSG00000172340	7071	69.02714061	260.62661675	213.155810205	2954	31.870593359	127.996288473	98.416392293	2949	32.389908654	130.301365635	100.020037924	SUCLG2	succinate-CoA ligase GDP-forming subunit beta [Source:HGNC Symbol;Acc:HGNC:11450]	Metabolism	Overview;Carbohydrate metabolism	ko01200//Carbon metabolism;ko00020//Citrate cycle (TCA cycle);ko00640//Propanoate metabolism	K01900	GO:0005575//cellular_component;GO:0005737//cytoplasm;GO:0043231//intracellular membrane-bounded organelle;GO:0016020//membrane;GO:0005623//cell;GO:0032991//macromolecular complex;GO:0005622//intracellular;GO:0044444//cytoplasmic part;GO:0005739//mitochondrion;GO:0044424//intracellular part;GO:0043226//organelle;GO:0071944//cell periphery;GO:0044464//cell part;GO:0043227//membrane-bounded organelle;GO:0043229//intracellular organelle;GO:0005886//plasma membrane	GO:0003824//catalytic activity;GO:0016874//ligase activity;GO:0043167//ion binding;GO:0003674//molecular_function;GO:0005488//binding	GO:0044699//single-organism process;GO:0044281//small molecule metabolic process;GO:0051186//cofactor metabolic process;GO:0044710//single-organism metabolic process;GO:0008152//metabolic process;GO:0008150//biological_process;GO:0006790//sulfur compound metabolic process;GO:0034641//cellular nitrogen compound metabolic process;GO:0044237//cellular metabolic process;GO:0006807//nitrogen compound metabolic process;GO:0006091//generation of precursor metabolites and energy;GO:0009987//cellular process

id	CON_Mean	KD_Mean	AllMean	log2FoldChange	pvalue	qvalue	State	gene_id	XF_1_count	XF_1_FPKM	XF_1_TPM	XF_1_CPM	XF_2_count	XF_2_FPKM	XF_2_TPM	XF_2_CPM	XF_3_count	XF_3_FPKM	XF_3_TPM	XF_3_CPM	Symbol	Description	KEGG_A_class	KEGG_B_class	Pathway	K_ID	GO Component	GO Function	GO Process
RBM14	5.56885592	6.888356443	6.228606181	1.342277347	1.69829E-05	0.002926899	Up	ENSG00000239306	1583	7.364143571	27.804886704	47.71965034	4163	21.403675343	85.95983679	138.695816221	2924	15.304339978	61.567830293	99.172123055	RBM14	RNA binding motif protein 14 [Source:HGNC Symbol;Acc:HGNC:14219]	-	-	-	-	GO:0044422//organelle part;GO:0043226//organelle;GO:0005634//nucleus;GO:0043229//intracellular organelle;GO:0031974//membrane-enclosed lumen;GO:0005654//nucleoplasm;GO:0005622//intracellular;GO:0070013//intracellular organelle lumen;GO:0031981//nuclear lumen;GO:0044464//cell part;GO:0043227//membrane-bounded organelle;GO:0005730//nucleolus;GO:0044424//intracellular part;GO:0043228//non-membrane-bounded organelle;GO:0032991//macromolecular complex;GO:0043231//intracellular membrane-bounded organelle;GO:0043232//intracellular non-membrane-bounded organelle;GO:0005575//cellular_component;GO:0044446//intracellular organelle part;GO:0044428//nuclear part;GO:0005623//cell;GO:0043233//organelle lumen;GO:0005737//cytoplasm	GO:0005488//binding;GO:0097159//organic cyclic compound binding;GO:0003723//RNA binding;GO:0003674//molecular_function;GO:0003676//nucleic acid binding;GO:0003729//mRNA binding;GO:0044822//poly(A) RNA binding;GO:1901363//heterocyclic compound binding	GO:0009987//cellular process;GO:0044238//primary metabolic process;GO:0008152//metabolic process;GO:0016043//cellular component organization;GO:0002376//immune system process;GO:0022607//cellular component assembly;GO:0050896//response to stimulus;GO:0006807//nitrogen compound metabolic process;GO:0044085//cellular component biogenesis;GO:0006464//cellular protein modification process;GO:0050794//regulation of cellular process;GO:0044237//cellular metabolic process;GO:0019538//protein metabolic process;GO:0009058//biosynthetic process;GO:0043412//macromolecule modification;GO:0043170//macromolecule metabolic process;GO:0023052//signaling;GO:0006996//organelle organization;GO:0050789//regulation of biological process;GO:0044699//single-organism process;GO:0007154//cell communication;GO:0007165//signal transduction;GO:0034641//cellular nitrogen compound metabolic process;GO:0044267//cellular protein metabolic process;GO:0036211//protein modification [illegible]

id	CON_Mean	KD_Mean	AllMean	log2FoldChange	pvalue	qvalue	State	gene_id	XF_1_count	XF_1_FPKM	XF_1_TPM	XF_1_CPM	XF_2_count	XF_2_FPKM	XF_2_TPM	XF_2_CPM	XF_3_count	XF_3_FPKM	XF_3_TPM	XF_3_CPM	Symbol	Description	KEGG_A_class	KEGG_B_class	Pathway	K_ID	GO Component	GO Function	GO Process
EHD1	5.994257089	7.19979762	6.597027354	1.209937835	1.7319E-05	0.00295497	Up	ENSG00000110047	2127	9.051181533	34.174656513	64.118569977	4653	21.883231254	87.8857933	155.020810203	4030	19.294731345	77.620775987	136.683876851	EHD1	EH domain containing 1 [Source:HGNC Symbol;Acc:HGNC:3242]	Cellular Processes	Transport and catabolism	ko04144//Endocytosis	K12483	GO:0016020//membrane;GO:0043229//intracellular organelle;GO:0044464//cell part;GO:0043231//intracellular membrane-bounded organelle;GO:0031982//vesicle;GO:0005575//cellular_component;GO:0005623//cell;GO:0042995//cell projection;GO:0005811//lipid particle;GO:0043226//organelle;GO:0005576//extracellular region;GO:0005929//cilium;GO:0005886//plasma membrane;GO:0031410//cytoplasmic vesicle;GO:0044421//extracellular region part;GO:0005773//vacuole;GO:0097708//intracellular vesicle;GO:0043228//non-membrane-bounded organelle;GO:0071944//cell periphery;GO:0043232//intracellular non-membrane-bounded organelle;GO:0044444//cytoplasmic part;GO:0005622//intracellular;GO:0012505//endomembrane system;GO:0043227//membrane-bounded organelle;GO:0005615//extracellular space;GO:0044424//intracellular part;GO:0005737//cytoplasm;GO:0005768//endosome	GO:0019899//enzyme binding;GO:0043167//ion binding;GO:0005488//binding;GO:0005515//protein binding;GO:0003674//molecular_function	GO:0071840//cellular component organization or biogenesis;GO:0051234//establishment of localization;GO:0048646//anatomical structure formation involved in morphogenesis;GO:0009987//cellular process;GO:0022607//cellular component assembly;GO:0044699//single-organism process;GO:0045184//establishment of protein localization;GO:0030154//cell differentiation;GO:0006810//transport;GO:0016043//cellular component organization;GO:0009653//anatomical structure morphogenesis;GO:0032502//developmental process;GO:0044767//single-organism developmental process;GO:0015031//protein transport;GO:0008104//protein localization;GO:0065003//macromolecular complex assembly;GO:0051179//localization;GO:0016192//vesicle-mediated transport;GO:0043933//macromolecular complex subunit organization;GO:0048869//cellular developmental process;GO:0006950//response to stress;GO:0044763//single-organism cellular process;GO:0050896//response to stimulus;GO:0065008//regulation of biological [illegible]

id	CON_Mean	KD_Mean	AllMean	log2FoldChange	pvalue	qvalue	State	gene_id	XF_1_count	XF_1_FPKM	XF_1_TPM	XF_1_CPM	XF_2_count	XF_2_FPKM	XF_2_TPM	XF_2_CPM	XF_3_count	XF_3_FPKM	XF_3_TPM	XF_3_CPM	Symbol	Description	KEGG_A_class	KEGG_B_class	Pathway	K_ID	GO Component	GO Function	GO Process
SPTBN5	1.735186618	4.041375954	2.888281286	2.347310426	1.90121E-05	0.003211729	Up	ENSG00000137877	108	0.264430464	0.998413327	3.255667869	540	1.461241853	5.868530016	17.990809695	431	1.187301197	4.776394063	14.618052338	SPTBN5	spectrin beta, non-erythrocytic 5 [Source:HGNC Symbol;Acc:HGNC:15680]	-	-	-	-	GO:0044424//intracellular part;GO:0005622//intracellular;GO:0043232//intracellular non-membrane-bounded organelle;GO:0005829//cytosol;GO:0042995//cell projection;GO:0005623//cell;GO:0043229//intracellular organelle;GO:0032991//macromolecular complex;GO:0043226//organelle;GO:0044464//cell part;GO:0005575//cellular_component;GO:0005929//cilium;GO:0005856//cytoskeleton;GO:0005737//cytoplasm;GO:0043228//non-membrane-bounded organelle;GO:0044444//cytoplasmic part	GO:0005515//protein binding;GO:0008092//cytoskeletal protein binding;GO:0005488//binding;GO:0003674//molecular_function	GO:0019538//protein metabolic process;GO:0044260//cellular macromolecule metabolic process;GO:0051234//establishment of localization;GO:0022607//cellular component assembly;GO:0044763//single-organism cellular process;GO:0008150//biological_process;GO:0043170//macromolecule metabolic process;GO:0006810//transport;GO:0048856//anatomical structure development;GO:0016192//vesicle-mediated transport;GO:0032502//developmental process;GO:0007165//signal transduction;GO:0043933//macromolecular complex subunit organization;GO:0008152//metabolic process;GO:0023052//signaling;GO:0050896//response to stimulus;GO:0006996//organelle organization;GO:0000902//cell morphogenesis;GO:0071840//cellular component organization or biogenesis;GO:0044699//single-organism process;GO:0030154//cell differentiation;GO:0044238//primary metabolic process;GO:0016043//cellular component organization;GO:0007154//cell communication;GO:000701[illegible]

id	CON_Mean	KD_Mean	AllMean	log2FoldChange	pvalue	qvalue	State	gene_id	XF_1_count	XF_1_FPKM	XF_1_TPM	XF_1_CPM	XF_2_count	XF_2_FPKM	XF_2_TPM	XF_2_CPM	XF_3_count	XF_3_FPKM	XF_3_TPM	XF_3_CPM	Symbol	Description	KEGG_A_class	KEGG_B_class	Pathway	K_ID	GO Component	GO Function	GO Process
CCDC86	5.083704137	6.29590614	5.689805138	1.214973429	1.95939E-05	0.003277559	Up	ENSG00000110104	1130	10.192678735	38.484621425	34.063932334	2264	22.569759264	90.642975642	75.428135461	2363	23.9811231	96.473661681	80.144913399	CCDC86	coiled-coil domain containing 86 [Source:HGNC Symbol;Acc:HGNC:28359]	-	-	-	-	GO:0005622//intracellular;GO:0043233//organelle lumen;GO:0005654//nucleoplasm;GO:0031981//nuclear lumen;GO:0043226//organelle;GO:0044424//intracellular part;GO:0043229//intracellular organelle;GO:0031974//membrane-enclosed lumen;GO:0005730//nucleolus;GO:0043228//non-membrane-bounded organelle;GO:0044428//nuclear part;GO:0044422//organelle part;GO:0005634//nucleus;GO:0044464//cell part;GO:0043231//intracellular membrane-bounded organelle;GO:0005623//cell;GO:0043227//membrane-bounded organelle;GO:0070013//intracellular organelle lumen;GO:0043232//intracellular non-membrane-bounded organelle;GO:0044446//intracellular organelle part;GO:0005575//cellular_component	GO:0003676//nucleic acid binding;GO:0003674//molecular_function;GO:1901363//heterocyclic compound binding;GO:0097159//organic cyclic compound binding;GO:0005488//binding;GO:0003723//RNA binding	GO:0051704//multi-organism process;GO:0008150//biological_process;GO:0044403//symbiosis, encompassing mutualism through parasitism;GO:0044419//interspecies interaction between organisms
TNRC18	7.161223241	8.328760673	7.744991957	1.168674526	2.07777E-05	0.00342483	Up	ENSG00000182095	4780	11.204778249	42.306017902	144.093448279	9806	25.404337289	102.026995463	326.69977753	9153	24.139859391	97.112241916	310.438591767	TNRC18	trinucleotide repeat containing 18 [Source:HGNC Symbol;Acc:HGNC:11962]	-	-	-	-	GO:0044446//intracellular organelle part;GO:0044464//cell part;GO:0005622//intracellular;GO:0043227//membrane-bounded organelle;GO:0005623//cell;GO:0044424//intracellular part;GO:0043226//organelle;GO:0005575//cellular_component;GO:0044444//cytoplasmic part;GO:0012505//endomembrane system;GO:0044428//nuclear part;GO:0005635//nuclear envelope;GO:0005739//mitochondrion;GO:0005737//cytoplasm;GO:0044422//organelle part;GO:0031974//membrane-enclosed lumen;GO:0005654//nucleoplasm;GO:0043231//intracellular membrane-bounded organelle;GO:0005829//cytosol;GO:0043233//organelle lumen;GO:0031975//envelope;GO:0031967//organelle envelope;GO:0070013//intracellular organelle lumen;GO:0005634//nucleus;GO:0031981//nuclear lumen;GO:0043229//intracellular organelle	GO:0003674//molecular_function	-

id	CON_Mean	KD_Mean	AllMean	log2FoldChange	pvalue	qvalue	State	gene_id	XF_1_count	XF_1_FPKM	XF_1_TPM	XF_1_CPM	XF_2_count	XF_2_FPKM	XF_2_TPM	XF_2_CPM	XF_3_count	XF_3_FPKM	XF_3_TPM	XF_3_CPM	Symbol	Description	KEGG_A_class	KEGG_B_class	Pathway	K_ID	GO Component	GO Function	GO Process
ASS1	2.524331523	-0.00194	1.261193801	-2.59295	2.08758E-05	0.00342483	Down	ENSG00000130707	189	1.887812714	7.127837491	5.697418771	20	0.220784057	0.886696384	0.666326285	35	0.393333604	1.58234178	1.187080816	ASS1	argininosuccinate synthase 1 [Source:HGNC Symbol;Acc:HGNC:758]	Metabolism	Overview;Amino acid metabolism	ko01230//Biosynthesis of amino acids;ko00250//Alanine, aspartate and glutamate metabolism;ko00220//Arginine biosynthesis	K01940	GO:0044421//extracellular region part;GO:0005773//vacuole;GO:0000323//lytic vacuole;GO:0005615//extracellular space;GO:0005623//cell;GO:0012505//endomembrane system;GO:0005829//cytosol;GO:0043226//organelle;GO:0044464//cell part;GO:0005739//mitochondrion;GO:0005737//cytoplasm;GO:0005764//lysosome;GO:0043227//membrane-bounded organelle;GO:0005783//endoplasmic reticulum;GO:0005576//extracellular region;GO:0005575//cellular_component;GO:0005622//intracellular;GO:0044444//cytoplasmic part;GO:0005634//nucleus;GO:0043229//intracellular organelle;GO:0044424//intracellular part;GO:0043231//intracellular membrane-bounded organelle	GO:0003676//nucleic acid binding;GO:0097159//organic cyclic compound binding;GO:0005488//binding;GO:1901363//heterocyclic compound binding;GO:0016874//ligase activity;GO:0003723//RNA binding;GO:0003674//molecular_function;GO:0043167//ion binding;GO:0003824//catalytic activity	GO:0048856//anatomical structure development;GO:0044281//small molecule metabolic process;GO:0006082//organic acid metabolic process;GO:1901564//organonitrogen compound metabolic process;GO:0044238//primary metabolic process;GO:0043436//oxoacid metabolic process;GO:0007568//aging;GO:0007155//cell adhesion;GO:0009987//cellular process;GO:0032502//developmental process;GO:0071704//organic substance metabolic process;GO:0071941//nitrogen cycle metabolic process;GO:0006520//cellular amino acid metabolic process;GO:0008152//metabolic process;GO:0050896//response to stimulus;GO:0044237//cellular metabolic process;GO:0034641//cellular nitrogen compound metabolic process;GO:0008150//biological_process;GO:0044763//single-organism cellular process;GO:0006950//response to stress;GO:0044710//single-organism metabolic process;GO:0019752//carboxylic acid metabolic process;GO:0009058//biosynthetic process;GO:0022610//biological adhesion;GO:0002376//im

id	CON_Mean	KD_Mean	AllMean	log2FoldChange	pvalue	qvalue	State	gene_id	XF_1_count	XF_1_FPKM	XF_1_TPM	XF_1_CPM	XF_2_count	XF_2_FPKM	XF_2_TPM	XF_2_CPM	XF_3_count	XF_3_FPKM	XF_3_TPM	XF_3_CPM	Symbol	Description	KEGG_A_class	KEGG_B_class	Pathway	K_ID	GO Component	GO Function	GO Process
RPL22L1	7.399246638	6.281260658	6.840253648	-1.11197	2.13349E-05	0.003466827	Down	ENSG00000163584	5638	82.865880446	312.877715542	169.957920794	2086	33.884852035	136.085803208	69.497831524	2513	41.556510294	167.177687968	85.232402612	RPL22L1	ribosomal protein L22 like 1 [Source:HGNC Symbol;Acc:HGNC:27610]	Genetic Information Processing	Translation	ko03010//Ribosome	K02891	GO:0044464//cell part;GO:0032991//macromolecular complex;GO:0044444//cytoplasmic part;GO:0005623//cell;GO:0030529//intracellular ribonucleoprotein complex;GO:0005622//intracellular;GO:0005575//cellular_component;GO:0043229//intracellular organelle;GO:0043232//intracellular non-membrane-bounded organelle;GO:0043226//organelle;GO:0043228//non-membrane-bounded organelle;GO:0005840//ribosome;GO:0044424//intracellular part;GO:1990904//ribonucleoprotein complex;GO:0005737//cytoplasm	GO:1901363//heterocyclic compound binding;GO:0003676//nucleic acid binding;GO:0005198//structural molecule activity;GO:0003723//RNA binding;GO:0003674//molecular_function;GO:0003735//structural constituent of ribosome;GO:0097159//organic cyclic compound binding;GO:0005488//binding	GO:0009058//biosynthetic process;GO:0019538//protein metabolic process;GO:0034645//cellular macromolecule biosynthetic process;GO:1901576//organic substance biosynthetic process;GO:0043170//macromolecule metabolic process;GO:0044237//cellular metabolic process;GO:0044271//cellular nitrogen compound biosynthetic process;GO:0044260//cellular macromolecule metabolic process;GO:0044238//primary metabolic process;GO:1901566//organonitrogen compound biosynthetic process;GO:1901564//organonitrogen compound metabolic process;GO:0009059//macromolecule biosynthetic process;GO:0043043//peptide biosynthetic process;GO:0044267//cellular protein metabolic process;GO:0043603//cellular amide metabolic process;GO:0006807//nitrogen compound metabolic process;GO:0008152//metabolic process;GO:0044249//cellular biosynthetic process;GO:0008150//biological_process;GO:0009987//cellular process;GO:0006412//translation;GO:0034641//cellular nitrogen compound metabolic process;GO:0043604//amid [illegible]

id	CON_Mean	KD_Mean	AllMean	log2FoldChange	pvalue	qvalue	State	gene_id	XF_1_count	XF_1_FPKM	XF_1_TPM	XF_1_CPM	XF_2_count	XF_2_FPKM	XF_2_TPM	XF_2_CPM	XF_3_count	XF_3_FPKM	XF_3_TPM	XF_3_CPM	Symbol	Description	KEGG_A_class	KEGG_B_class	Pathway	K_ID	GO Component	GO Function	GO Process
CCNB1IP1	6.144616526	4.948598065	5.546607295	-1.19831	2.25539E-05	0.003630331	Down	ENSG00000100814	2361	19.340357888	73.023625177	71.172517026	926	8.38339864	33.668777315	30.850906995	889	8.193438243	32.961383242	30.151852735	CCNB1IP1	cyclin B1 interacting protein 1 [Source:HGNC Symbol;Acc:HGNC:19437]	-	-	-	-	GO:0044428//nuclear part;GO:0005575//cellular_component;GO:0005694//chromosome;GO:0005634//nucleus;GO:0044422//organelle part;GO:0043229//intracellular organelle;GO:0043228//non-membrane-bounded organelle;GO:0043232//intracellular non-membrane-bounded organelle;GO:0044424//intracellular part;GO:0005622//intracellular;GO:0000228//nuclear chromosome;GO:0043226//organelle;GO:0031974//membrane-enclosed lumen;GO:0070013//intracellular organelle lumen;GO:0043231//intracellular membrane-bounded organelle;GO:0044464//cell part;GO:0044446//intracellular organelle part;GO:0031981//nuclear lumen;GO:0043233//organelle lumen;GO:0043227//membrane-bounded organelle;GO:0005623//cell	GO:0003674//molecular_function;GO:0043167//ion binding;GO:0005488//binding	GO:0044707//single-multicellular organism process;GO:0071840//cellular component organization or biogenesis;GO:0019538//protein metabolic process;GO:0008152//metabolic process;GO:0009790//embryo development;GO:0006725//cellular aromatic compound metabolic process;GO:0034641//cellular nitrogen compound metabolic process;GO:0007049//cell cycle;GO:0009653//anatomical structure morphogenesis;GO:0006259//DNA metabolic process;GO:0022607//cellular component assembly;GO:0006996//organelle organization;GO:0051276//chromosome organization;GO:0048869//cellular developmental process;GO:0044260//cellular macromolecule metabolic process;GO:0044699//single-organism process;GO:0048856//anatomical structure development;GO:0007275//multicellular organism development;GO:0071704//organic substance metabolic process;GO:0032502//developmental process;GO:0044237//cellular metabolic process;GO:0006464//cellular protein modification [illegible]

id	CON_Mean	KD_Mean	AllMean	log2FoldChange	pvalue	qvalue	State	gene_id	XF_1_count	XF_1_FPKM	XF_1_TPM	XF_1_CPM	XF_2_count	XF_2_FPKM	XF_2_TPM	XF_2_CPM	XF_3_count	XF_3_FPKM	XF_3_TPM	XF_3_CPM	Symbol	Description	KEGG_A_class	KEGG_B_class	Pathway	K_ID	GO Component	GO Function	GO Process
DOK3	0.195064024	2.891046964	1.543055494	2.811040196	2.35937E-05	0.0037622	Up	ENSG00000146094	35	0.203643611	0.768899667	1.05507755	245	1.575467476	6.3272744	8.162496991	190	1.243804864	5.00370267	6.444153003	DOK3	docking protein 3 [Source:HGNC Symbol;Acc:HGNC:24583]	-	-	-	-	GO:0005575//cellular_component;GO:0044444//cytoplasmic part;GO:0016020//membrane;GO:0005886//plasma membrane;GO:0005623//cell;GO:0043226//organelle;GO:0044464//cell part;GO:0005737//cytoplasm;GO:0043227//membrane-bounded organelle;GO:0071944//cell periphery;GO:0097708//intracellular vesicle;GO:0031410//cytoplasmic vesicle;GO:0031982//vesicle;GO:0005622//intracellular;GO:0044424//intracellular part	GO:0003674//molecular_function	GO:0050794//regulation of cellular process;GO:0065007//biological regulation;GO:0007154//cell communication;GO:0050896//response to stimulus;GO:0006810//transport;GO:0009987//cellular process;GO:0044763//single-organism cellular process;GO:0050789//regulation of biological process;GO:0044700//single organism signaling;GO:0051179//localization;GO:0051716//cellular response to stimulus;GO:0007165//signal transduction;GO:0008150//biological_process;GO:0044699//single-organism process;GO:0016192//vesicle-mediated transport;GO:0002376//immune system process;GO:0023052//signaling;GO:0051234//establishment of localization
TFB1M	6.065605325	4.85552597	5.460565647	-1.21254	2.42612E-05	0.003763444	Down	ENSG00000029639	2235	16.38877106 4	61.87928280 7	67.37423784 6	841	6.815621572	27.37238855 5	28.01902028 4	860	7.095176718	28.54318688	29.16827148 7	TFB1M	transcription factor B1, mitochondrial [Source:HGNC Symbol;Acc:HGNC:17037]	-	-	-	-	GO:0043229//intracellular organelle;GO:0044464//cell part;GO:0005575//cellular_component;GO:0044424//intracellular part;GO:0005623//cell;GO:0005737//cytoplasm;GO:0043226//organelle;GO:0043231//intracellular membrane-bounded organelle;GO:0005739//mitochondrion;GO:0005622//intracellular;GO:0044444//cytoplasmic part;GO:0043227//membrane-bounded organelle	GO:0016741//transferase activity, transferring one-carbon groups;GO:0003677//DNA binding;GO:0003674//molecular_function;GO:0016740//transferase activity;GO:0097159//organic cyclic compound binding;GO:1901363//heterocyclic compound binding;GO:0003676//nucleic acid binding;GO:0003723//RNA binding;GO:0005488//binding;GO:0008168//methyltransferase activity;GO:0003824//catalytic activity	GO:0006807//nitrogen compound metabolic process;GO:0022613//ribonucleoprotein complex biogenesis;GO:0006996//organelle organization;GO:0071840//cellular component organization or biogenesis;GO:0009987//cellular process;GO:0007005//mitochondrion organization;GO:0044085//cellular component biogenesis;GO:0042254//ribosome biogenesis;GO:0044237//cellular metabolic process;GO:0008152//metabolic process;GO:0016043//cellular component organization;GO:0008150//biological_process;GO:0034641//cellular nitrogen compound metabolic process
ENSG00000255050	-1.03964	2.076020694	0.518188186	3.464798966	2.44379E-05	0.003763444	Up	ENSG00000255050	13	0.334088617	1.261422464	0.391885947	169	4.800048686	19.27759578 6	5.630457108	87	2.515553063	10.11981858 3	2.950743743	-	-	-	-	-	-	-	-	-
BANCR	2.625738934	0.269302668	1.447520801	-2.43465	2.45401E-05	0.003763444	Down	ENSG00000278910	203	7.216332301	27.24679388 7	6.119449791	28	1.10006698	4.418006559	0.932856799	38	1.519847406	6.11419423	1.288830601	-	-	-	-	-	-	-	-	-

id	CON_Mean	KD_Mean	AllMean	log2FoldChange	pvalue	qvalue	State	gene_id	XF_1_count	XF_1_FPKM	XF_1_TPM	XF_1_CPM	XF_2_count	XF_2_FPKM	XF_2_TPM	XF_2_CPM	XF_3_count	XF_3_FPKM	XF_3_TPM	XF_3_CPM	Symbol	Description	KEGG_A_class	KEGG_B_class	Pathway	K_ID	GO Component	GO Function	GO Process
RAVER1	5.537596809	6.76869005	6.15314343	1.242171142	2.45894E-05	0.003763444	Up	ENSG00000161847	1549	9.378332569	35.409884673	46.694717863	3619	24.216055688	97.254707955	120.571741269	2849	19.40718587	78.073169303	96.628378449	RAVER1	ribonucleoprotein, PTB binding 1 [Source:HGNC Symbol;Acc:HGNC:30296]	-	-	-	-	GO:0005737//cytoplasm;GO:0043227//membrane-bounded organelle;GO:0044424//intracellular part;GO:0043231//intracellular membrane-bounded organelle;GO:0044464//cell part;GO:0043226//organelle;GO:0005634//nucleus;GO:0005623//cell;GO:0043229//intracellular organelle;GO:0005622//intracellular;GO:0005575//cellular_component	GO:0003723//RNA binding;GO:1901363//heterocyclic compound binding;GO:0003674//molecular_function;GO:0097159//organic cyclic compound binding;GO:0003676//nucleic acid binding;GO:0005488//binding	GO:0044260//cellular macromolecule metabolic process;GO:0006807//nitrogen compound metabolic process;GO:0044237//cellular metabolic process;GO:1901360//organic cyclic compound metabolic process;GO:0006397//mRNA processing;GO:0034641//cellular nitrogen compound metabolic process;GO:0008152//metabolic process;GO:0006396//RNA processing;GO:0006139//nucleobase-containing compound metabolic process;GO:0008150//biological_process;GO:0043170//macromolecule metabolic process;GO:0009987//cellular process;GO:0090304//nucleic acid metabolic process;GO:0006725//cellular aromatic compound metabolic process;GO:0016071//mRNA metabolic process;GO:0016070//RNA metabolic process;GO:0046483//heterocycle metabolic process;GO:0010467//gene expression;GO:0044238//primary metabolic process;GO:0071704//organic substance metabolic process

id	CON_Mean	KD_Mean	AllMean	log2FoldChange	pvalue	qvalue	State	gene_id	XF_1_count	XF_1_FPKM	XF_1_TPM	XF_1_CPM	XF_2_count	XF_2_FPKM	XF_2_TPM	XF_2_CPM	XF_3_count	XF_3_FPKM	XF_3_TPM	XF_3_CPM	Symbol	Description	KEGG_A_class	KEGG_B_class	Pathway	K_ID	GO Component	GO Function	GO Process
KLHL21	6.523936765	7.718543232	7.121239999	1.203462549	2.47044E-05	0.003763444	Up	ENSG00000162413	3072	13.4191658 94	50.6669083 42	92.6056638 31	6933	33.4708023 03	134.422927 703	230.982006 692	5554	27.2964450 45	109.810870 549	188.372767 254	KLHL21	kelch like family member 21 [Source:HGNC Symbol;Acc:HGNC:29041]	-	-	-	-	GO:0097708//intracellular vesicle;GO:0044464//cell part;GO:0031410//cytoplasmic vesicle;GO:0032991//macromolecular complex;GO:0043226//organelle;GO:0005737//cytoplasm;GO:0031982//vesicle;GO:0043227//membrane-bounded organelle;GO:0043228//non-membrane-bounded organelle;GO:0005829//cytosol;GO:0044424//intracellular part;GO:0005575//cellular_component;GO:0012505//endomembrane system;GO:0005622//intracellular;GO:0005794//Golgi apparatus;GO:0005856//cytoskeleton;GO:0005623//cell;GO:0044444//cytoplasmic part;GO:0043229//intracellular organelle;GO:0043232//intracellular non-membrane-bounded organelle;GO:0043231//intracellular membrane-bounded organelle	GO:0003674//molecular_function	GO:0019538//protein metabolic process;GO:0008152//metabolic process;GO:0044238//primary metabolic process;GO:0044237//cellular metabolic process;GO:0065003//macromolecular complex assembly;GO:0009987//cellular process;GO:0051301//cell division;GO:0043170//macromolecule metabolic process;GO:0051179//localization;GO:0016192//vesicle-mediated transport;GO:0061024//membrane organization;GO:0043933//macromolecular complex subunit organization;GO:0044085//cellular component biogenesis;GO:0006810//transport;GO:0071840//cellular component organization or biogenesis;GO:0071704//organic substance metabolic process;GO:0043412//macromolecule modification;GO:0022607//cellular component assembly;GO:0044763//single-organism cellular process;GO:0044260//cellular macromolecule metabolic process;GO:0051234//establishment of localization;GO:0007049//cell cycle;GO:0008150//biological_process;GO:0044267//cellular protein metabolic process;GO:0036211//prot [illegible]

id	CON_Mean	KD_Mean	AllMean	log2FoldChange	pvalue	qvalue	State	gene_id	XF_1_count	XF_1_FPKM	XF_1_TPM	XF_1_CPM	XF_2_count	XF_2_FPKM	XF_2_TPM	XF_2_CPM	XF_3_count	XF_3_FPKM	XF_3_TPM	XF_3_CPM	Symbol	Description	KEGG_A_class	KEGG_B_class	Pathway	K_ID	GO Component	GO Function	GO Process
ATXN3L	-1.44713	1.461722479	0.007298552	3.285990598	2.55299E-05	0.003854787	Up	ENSG00000123594	9	0.192825626	0.728054068	0.271305656	84	1.989033687	7.988208022	2.798570397	73	1.759709607	7.079135892	2.475911417	ATXN3L	ataxin 3 like [Source:HGNC Symbol;Acc:HGNC:24173]	Genetic Information Processing	Folding, sorting and degradation	ko04141//Protein processing in endoplasmic reticulum	K11863	GO:0043227//membrane-bounded organelle;GO:0005623//cell;GO:0005634//nucleus;GO:0005829//cytosol;GO:0044444//cytoplasmic part;GO:0005737//cytoplasm;GO:0044424//intracellular part;GO:0044464//cell part;GO:0043231//intracellular membrane-bounded organelle;GO:0043229//intracellular organelle;GO:0005575//cellular_component;GO:0043226//organelle;GO:0005622//intracellular	GO:0003674//molecular_function;GO:0008233//peptidase activity;GO:0003824//catalytic activity;GO:0016787//hydrolase activity	GO:0044267//cellular protein metabolic process;GO:0043412//macromolecule modification;GO:0044260//cellular macromolecule metabolic process;GO:0036211//protein modification process;GO:0008152//metabolic process;GO:0044237//cellular metabolic process;GO:0009987//cellular process;GO:0019538//protein metabolic process;GO:0008150//biological_process;GO:0071704//organic substance metabolic process;GO:0043170//macromolecule metabolic process;GO:0044238//primary metabolic process;GO:0006464//cellular protein modification process

id	CON_Mean	KD_Mean	AllMean	log2FoldChange	pvalue	qvalue	State	gene_id	XF_1_count	XF_1_FPKM	XF_1_TPM	XF_1_CPM	XF_2_count	XF_2_FPKM	XF_2_TPM	XF_2_CPM	XF_3_count	XF_3_FPKM	XF_3_TPM	XF_3_CPM	Symbol	Description	KEGG_A_class	KEGG_B_class	Pathway	K_ID	GO Component	GO Function	GO Process
PHLDA2	5.015938482	6.375807304	5.695872893	1.392636132	2.61619E-05	0.003883841	Up	ENSG00000181649	1078	35.3221461462	133.366315868	32.496388545	2001	72.462983493	291.020403487	66.665944813	2987	110.118335353	442.995058467	101.308868525	PHLDA2	pleckstrin homology like domain family A member 2 [Source:HGNC Symbol;Acc:HGNC:12385]	-	-	-	-	GO:0044464//cell part;GO:0005622//intracellular;GO:0005623//cell;GO:0044424//intracellular part;GO:0005737//cytoplasm;GO:0005575//cellular_component	GO:0008289//lipid binding;GO:0043167//ion binding;GO:0005488//binding;GO:0003674//molecular_function	GO:0044767//single-organism developmental process;GO:0040011//locomotion;GO:0008152//metabolic process;GO:0006928//movement of cell or subcellular component;GO:0008150//biological_process;GO:0048870//cell motility;GO:0050789//regulation of biological process;GO:0050896//response to stimulus;GO:0044707//single-multicellular organism process;GO:0006091//generation of precursor metabolites and energy;GO:0044237//cellular metabolic process;GO:0032501//multicellular organismal process;GO:0008283//cell proliferation;GO:0007275//multicellular organism development;GO:0009790//embryo development;GO:0040007//growth;GO:0044699//single-organism process;GO:0044281//small molecule metabolic process;GO:0009987//cellular process;GO:0051674//localization of cell;GO:0044700//single organism signaling;GO:0048856//anatomical structure development;GO:0032502//developmental process;GO:0044763//single-organism cellular process;GO:0051179//localization;GO:0007154//cell communication;GO:002305[illegible]

id	CON_Mean	KD_Mean	AllMean	log2FoldChange	pvalue	qvalue	State	gene_id	XF_1_count	XF_1_FPKM	XF_1_TPM	XF_1_CPM	XF_2_count	XF_2_FPKM	XF_2_TPM	XF_2_CPM	XF_3_count	XF_3_FPKM	XF_3_TPM	XF_3_CPM	Symbol	Description	KEGG_A_class	KEGG_B_class	Pathway	K_ID	GO Component	GO Function	GO Process
ANKRD11	7.323646878	8.511493196	7.917570037	1.193916514	2.61776E-05	0.003883841	Up	ENSG00000167522	5350	8.259135546	31.184118821	161.27613981	9898	16.887636526	67.822860153	329.764878441	11683	20.292291519	81.633860874	396.24757649	ANKRD11	ankyrin repeat domain 11 [Source:HGNC Symbol;Acc:HGNC:21316]	-	-	-	-	GO:0044446//intracellular organelle part;GO:0031974//membrane-enclosed lumen;GO:0016020//membrane;GO:0005886//plasma membrane;GO:0005829//cytosol;GO:0005737//cytoplasm;GO:0043229//intracellular organelle;GO:0044424//intracellular part;GO:0043226//organelle;GO:0031981//nuclear lumen;GO:0005622//intracellular;GO:0044444//cytoplasmic part;GO:0043233//organelle lumen;GO:0043231//intracellular membrane-bounded organelle;GO:0043227//membrane-bounded organelle;GO:0071944//cell periphery;GO:0005654//nucleoplasm;GO:0044422//organelle part;GO:0070013//intracellular organelle lumen;GO:0005634//nucleus;GO:0005575//cellular_component;GO:0044428//nuclear part;GO:0005623//cell;GO:0044464//cell part	GO:0003674//molecular_function	GO:0048856//anatomical structure development;GO:0044699//single-organism process;GO:0044767//single-organism developmental process;GO:0044707//single-multicellular organism process;GO:0008150//biological_process;GO:0040007//growth;GO:0042592//homeostatic process;GO:0032502//developmental process;GO:0065007//biological regulation;GO:0007275//multicellular organism development;GO:0065008//regulation of biological quality;GO:0032501//multicellular organismal process;GO:0009790//embryo development

id	CON_Mean	KD_Mean	AllMean	log2FoldChange	pvalue	qvalue	State	gene_id	XF_1_count	XF_1_FPKM	XF_1_TPM	XF_1_CPM	XF_2_count	XF_2_FPKM	XF_2_TPM	XF_2_CPM	XF_3_count	XF_3_FPKM	XF_3_TPM	XF_3_CPM	Symbol	Description	KEGG_A_class	KEGG_B_class	Pathway	K_ID	GO Component	GO Function	GO Process
VAV3	3.16268279	0.944679457	2.053681123	-2.27041	2.87045E-05	0.004175206	Down	ENSG00000134215	296	1.22131694	4.611341269	8.922941567	50	0.228006531	0.91570274	1.665815712	58	0.269253011	1.083177954	1.967162496	VAV3	vav guanine nucleotide exchange factor 3 [Source:HGNC Symbol;Acc:HGNC:12659]	Environmental Information Processing;Cellular Processes;Organismal Systems	Signal transduction;Immune system;Cell motility;Cellular community	ko04650//Natural killer cell mediated cytotoxicity;ko04510//Focal adhesion;ko04810//Regulation of actin cytoskeleton;ko04062//Chemokine signaling pathway;ko04024//cAMP signaling [illegible]	K05730	GO:0005886//plasma membrane;GO:0005575//cellular_component;GO:0016020//membrane;GO:0044444//cytoplasmic part;GO:0005737//cytoplasm;GO:0005623//cell;GO:0005622//intracellular;GO:0044464//cell part;GO:0071944//cell periphery;GO:0044424//intracellular part;GO:0005829//cytosol	GO:0030674//protein binding, bridging;GO:0005515//protein binding;GO:0060090//binding, bridging;GO:0043167//ion binding;GO:0003674//molecular_function;GO:0098772//molecular function regulator;GO:0019899//enzyme binding;GO:0030234//enzyme regulator activity;GO:0005488//binding	GO:0044700//single organism signaling;GO:0008283//cell proliferation;GO:0008219//cell death;GO:0006928//movement of cell or subcellular component;GO:0007155//cell adhesion;GO:0048870//cell motility;GO:0050789//regulation of biological process;GO:0006950//response to stress;GO:0051716//cellular response to stimulus;GO:0007154//cell communication;GO:0032502//developmental process;GO:0009653//anatomical structure morphogenesis;GO:0050794//regulation of cellular process;GO:0007165//signal transduction;GO:0022610//biological adhesion;GO:0040011//locomotion;GO:0048856//anatomical structure development;GO:0051179//localization;GO:0022607//cellular component assembly;GO:0016192//vesicle-mediated transport;GO:0044699//single-organism process;GO:0065007//biological regulation;GO:0071704//organic substance metabolic process;GO:0044763//single-organism cellular process;GO:0048646//anatomical structure formation involved in morphogenesis;GO:0050896//response to [illegible]
SLC43A3	5.699209971	6.857299332	6.278254651	1.160272288	2.87626E-05	0.004175206	Up	ENSG00000134802	1733	11.565510575	43.668039348	52.241411269	3545	26.147074167	105.009919651	118.106334014	3289	24.695966388	99.349404761	111.55168014	SLC43A3	solute carrier family 43 member 3 [Source:HGNC Symbol;Acc:HGNC:17466]	-	-	-	-	GO:0005575//cellular_component	GO:0003674//molecular_function	GO:0051179//localization;GO:0006810//transport;GO:0008150//biological_process;GO:0055085//transmembrane transport;GO:0051234//establishment of localization

id	CON_Mean	KD_Mean	AllMean	log2FoldChange	pvalue	qvalue	State	gene_id	XF_1_count	XF_1_FPKM	XF_1_TPM	XF_1_CPM	XF_2_count	XF_2_FPKM	XF_2_TPM	XF_2_CPM	XF_3_count	XF_3_FPKM	XF_3_TPM	XF_3_CPM	Symbol	Description	KEGG_A_class	KEGG_B_class	Pathway	K_ID	GO Component	GO Function	GO Process
MT-ND5	10.464380838	11.6176955 96	11.0410382 17	1.15491712	2.88826E-05	0.004175206	Up	ENSG00000198786	47211	785.418893418	2965.515695895	1423.179034874	97531	1793.252453126	7201.926105021	3249.373445065	87960	1646.414831348	6623.362332305	2983.303674402	MT-ND5	mitochondrially encoded NADH:ubiquinone oxidoreductase core subunit 5 [Source:HGNC Symbol;Acc:HGNC:7461]	Human Diseases;Metabolism	Energy metabolism; Neurodegenerative diseases	ko05012//Parkinson's disease; ko00190//Oxidative phosphorylation	K03883	GO:0005737//cytoplasm;GO:0005739//mitochondrion;GO:0043229//intracellular organelle;GO:0044424//intracellular part;GO:0044464//cell part;GO:0005623//cell;GO:0043226//organelle;GO:0044444//cytoplasmic part;GO:0005622//intracellular;GO:0043231//intracellular membrane-bounded organelle;GO:0005575//cellular_component;GO:0032991//macromolecular complex;GO:0043227//membrane-bounded organelle	GO:0003824//catalytic activity;GO:0016491//oxidoreductase activity;GO:0003674//molecular_function	GO:0043933//macromolecular complex subunit organization;GO:0044085//cellular component biogenesis;GO:0022607//cellular component assembly;GO:0044237//cellular metabolic process;GO:0050896//response to stimulus;GO:0065003//macromolecular complex assembly;GO:0008150//biological_process;GO:0009987//cellular process;GO:0016043//cellular component organization;GO:0071840//cellular component organization or biogenesis;GO:0008152//metabolic process;GO:0006091//generation of precursor metabolites and energy;GO:0006996//organelle organization;GO:0006950//response to stress;GO:0007005//mitochondrion organization

id	CON_Mean	KD_Mean	AllMean	log2FoldChange	pvalue	qvalue	State	gene_id	XF_1_count	XF_1_FPKM	XF_1_TPM	XF_1_CPM	XF_2_count	XF_2_FPKM	XF_2_TPM	XF_2_CPM	XF_3_count	XF_3_FPKM	XF_3_TPM	XF_3_CPM	Symbol	Description	KEGG_A_class	KEGG_B_class	Pathway	K_ID	GO Component	GO Function	GO Process
GBP2	5.936216924	4.105918332	5.021067628	-1.72755	2.91202E-05	0.004175206	Down	ENSG00000162645	2043	8.526427226	32.193335278	61.586383856	342	1.577485736	6.335379988	11.394179473	744	3.493554824	14.05422193	25.233946496	GBP2	guanylate binding protein 2 [Source:HGNC Symbol;Acc:HGNC:4183]	-	-	-	-	GO:0005575//cellular_component;GO:0005634//nucleus;GO:0012505//endomembrane system;GO:0031982//vesicle;GO:0043232//intracellular non-membrane-bounded organelle;GO:0044446//intracellular organelle part;GO:0031974//membrane-enclosed lumen;GO:0005856//cytoskeleton;GO:0043227//membrane-bounded organelle;GO:0005654//nucleoplasm;GO:0044464//cell part;GO:0044444//cytoplasmic part;GO:0005829//cytosol;GO:0005794//Golgi apparatus;GO:0070013//intracellular organelle lumen;GO:0044422//organelle part;GO:0043226//organelle;GO:0044424//intracellular part;GO:0044428//nuclear part;GO:0005576//extracellular region;GO:0005623//cell;GO:0043228//non-membrane-bounded organelle;GO:0043229//intracellular organelle;GO:0005737//cytoplasm;GO:0031410//cytoplasmic vesicle;GO:0043231//intracellular membrane-bounded organelle;GO:0043233//organelle lumen;GO:0031981//nuclear lumen;GO:0097708//intracellular vesicle;GO:0005622//intracellular	GO:0016462//pyrophosphatase activity;GO:0005488//binding;GO:0016787//hydrolase activity;GO:0016818//hydrolase activity, acting on acid anhydrides, in phosphorus-containing anhydrides;GO:0003924//GTPase activity;GO:0016817//hydrolase activity, acting on acid anhydrides;GO:0003674//molecular_function;GO:0043167//ion binding;GO:0003824//catalytic activity;GO:0017111//nucleoside-triphosphatase activity	GO:0051716//cellular response to stimulus;GO:0065007//biological regulation;GO:0007165//signal transduction;GO:0050794//regulation of cellular process;GO:0009987//cellular process;GO:0050789//regulation of biological process;GO:0044763//single-organism cellular process;GO:0008150//biological_process;GO:0002376//immune system process;GO:0044699//single-organism process;GO:0050896//response to stimulus;GO:0007154//cell communication;GO:0006950//response to stress;GO:0044700//single organism signaling;GO:0023052//signaling

id	CON_Mean	KD_Mean	AllMean	log2FoldChange	pvalue	qvalue	State	gene_id	XF_1_count	XF_1_FPKM	XF_1_TPM	XF_1_CPM	XF_2_count	XF_2_FPKM	XF_2_TPM	XF_2_CPM	XF_3_count	XF_3_FPKM	XF_3_TPM	XF_3_CPM	Symbol	Description	KEGG_A_class	KEGG_B_class	Pathway	K_ID	GO Component	GO Function	GO Process
COLEC12	3.442289154	0.676811784	2.059550469	-2.66077	3.0908E-05	0.004394606	Down	ENSG00000158270	360	1.477900889	5.580128414	10.852226223	26	0.117965977	0.473766116	0.86622417	74	0.341798721	1.375022099	2.509828012	COLEC12	collectin subfamily member 12 [Source:HGNC Symbol;Acc:HGNC:16016]	Cellular Processes	Transport and catabolism	ko04145//Phagosome	K10062	GO:0031410//cytoplasmic vesicle;GO:0071944//cell periphery;GO:0044464//cell part;GO:0005886//plasma membrane;GO:0005615//extracellular space;GO:0005622//intracellular;GO:0043227//membrane-bounded organelle;GO:0005575//cellular_component;GO:0031012//extracellular matrix;GO:0032991//macromolecular complex;GO:0005576//extracellular region;GO:0043226//organelle;GO:0097708//intracellular vesicle;GO:0044421//extracellular region part;GO:0044444//cytoplasmic part;GO:0005623//cell;GO:0044424//intracellular part;GO:0016020//membrane;GO:0005737//cytoplasm;GO:0031982//vesicle	GO:0003674//molecular_function;GO:0043167//ion binding;GO:0005488//binding	GO:0044699//single-organism process;GO:0023052//signaling;GO:0002376//immune system process;GO:0050789//regulation of biological process;GO:0007165//signal transduction;GO:0051179//localization;GO:0006950//response to stress;GO:0009987//cellular process;GO:0006810//transport;GO:0044763//single-organism cellular process;GO:0044700//single organism signaling;GO:0051716//cellular response to stimulus;GO:0065007//biological regulation;GO:0016192//vesicle-mediated transport;GO:0051234//establishment of localization;GO:0050896//response to stimulus;GO:0008150//biological_process;GO:0050794//regulation of cellular process;GO:0007154//cell communication
KCNAB3	0.33853382	2.96663072	1.65258227	2.729877095	3.15752E-05	0.004452369	Up	ENSG00000170049	39	0.253757358	0.958114753	1.175657842	255	1.833727635	7.364479498	8.495660134	203	1.486092971	5.97840351	6.885068735	KCNAB3	potassium voltage-gated channel subfamily A regulatory beta subunit 3 [Source:HGNC Symbol;Acc:HGNC:6230]	-	-	-	-	GO:0044424//intracellular part;GO:0071944//cell periphery;GO:0005622//intracellular;GO:0005575//cellular_component;GO:0005886//plasma membrane;GO:0005737//cytoplasm;GO:0016020//membrane;GO:0044464//cell part;GO:0005623//cell	GO:0003674//molecular_function;GO:0022857//transmembrane transporter activity;GO:0005215//transporter activity	GO:0051179//localization;GO:0051234//establishment of localization;GO:0006810//transport;GO:0055085//transmembrane transport;GO:0008150//biological_process

id	CON_Mean	KD_Mean	AllMean	log2FoldChange	pvalue	qvalue	State	gene_id	XF_1_count	XF_1_FPKM	XF_1_TPM	XF_1_CPM	XF_2_count	XF_2_FPKM	XF_2_TPM	XF_2_CPM	XF_3_count	XF_3_FPKM	XF_3_TPM	XF_3_CPM	Symbol	Description	KEGG_A_class	KEGG_B_class	Pathway	K_ID	GO Component	GO Function	GO Process
TAC1	-0.19189	2.69571646	1.251914102	3.093955988	3.21294E-05	0.00449337	Up	ENSG00000006128	26	0.457810686	1.728561391	0.783771894	256	4.981878766	20.007848129	8.528976448	138	2.733931119	10.998331677	4.680490076	TAC1	tachykinin precursor 1 [Source:HGNC Symbol;Acc:HGNC:11517]	Environmental Information Processing	Signaling molecules and interaction	ko04080//Neuroactive ligand-receptor interaction	K05239	GO:0005623//cell;GO:0005576//extracellular region;GO:0016020//membrane;GO:0044421//extracellular region part;GO:0005615//extracellular space;GO:0005575//cellular_component;GO:0044464//cell part;GO:0005886//plasma membrane;GO:0071944//cell periphery	GO:0003674//molecular_function	GO:0016043//cellular component organization;GO:0032501//multicellular organismal process;GO:0002376//immune system process;GO:0006996//organelle organization;GO:0009987//cellular process;GO:0007165//signal transduction;GO:0006950//response to stress;GO:0048870//cell motility;GO:0050789//regulation of biological process;GO:0051674//localization of cell;GO:0044763//single-organism cellular process;GO:0022607//cellular component assembly;GO:0065007//biological regulation;GO:0007154//cell communication;GO:0044699//single-organism process;GO:0007010//cytoskeleton organization;GO:0000003//reproduction;GO:0065008//regulation of biological quality;GO:0051716//cellular response to stimulus;GO:0044700//single organism signaling;GO:0023052//signaling;GO:0050877//neurological system process;GO:0008283//cell proliferation;GO:0071840//cellular component organization or biogenesis;GO:0050896//response to stimulus;GO:0051179//loca[illegible]
PRR12	5.770538485	6.921160066	6.345849276	1.152462036	3.24643E-05	0.004503297	Up	ENSG00000126464	1821	7.266901996	27.437730505	54.894177681	3682	16.239167205	65.218526267	122.670669067	3460	15.535003686	62.495767344	117.351417843	PRR12	proline rich 12 [Source:HGNC Symbol;Acc:HGNC:29217]	-	-	-	-	GO:0044424//intracellular part;GO:0005622//intracellular;GO:0043226//organelle;GO:0043231//intracellular membrane-bounded organelle;GO:0043227//membrane-bounded organelle;GO:0044464//cell part;GO:0005575//cellular_component;GO:0005634//nucleus;GO:0043229//intracellular organelle;GO:0005623//cell	-	-

id	CON_Mean	KD_Mean	AllMean	log2FoldChange	pvalue	qvalue	State	gene_id	XF_1_count	XF_1_FPKM	XF_1_TPM	XF_1_CPM	XF_2_count	XF_2_FPKM	XF_2_TPM	XF_2_CPM	XF_3_count	XF_3_FPKM	XF_3_TPM	XF_3_CPM	Symbol	Description	KEGG_A_class	KEGG_B_class	Pathway	K_ID	GO Component	GO Function	GO Process
SERPINH1	6.948063571	8.086927725	7.517495648	1.140887554	3.34241E-05	0.004599053	Up	ENSG00000149257	4123	19.787953416	74.713617075	124.288135409	7703	40.859030196	164.094974851	256.635568664	8332	44.991731806	180.997240815	282.593067475	SERPINH1	serpin family H member 1 [Source:HGNC Symbol;Acc:HGNC:1546]	-	-	-	-	GO:0043231//intracellular membrane-bounded organelle;GO:0044421//extracellular region part;GO:0005575//cellular_component;GO:0005783//endoplasmic reticulum;GO:0043227//membrane-bounded organelle;GO:0032991//macromolecular complex;GO:0005623//cell;GO:0044444//cytoplasmic part;GO:0043229//intracellular organelle;GO:0005622//intracellular;GO:0043226//organelle;GO:0005737//cytoplasm;GO:0044424//intracellular part;GO:0031012//extracellular matrix;GO:0012505//endomembrane system;GO:0005576//extracellular region;GO:0005615//extracellular space;GO:0044464//cell part	GO:0051082//unfolded protein binding;GO:0003723//RNA binding;GO:0003676//nucleic acid binding;GO:0098772//molecular function regulator;GO:0097159//organic cyclic compound binding;GO:0005488//binding;GO:1901363//heterocyclic compound binding;GO:0030234//enzyme regulator activity;GO:0005515//protein binding;GO:0003674//molecular_function	GO:0009058//biosynthetic process;GO:0006950//response to stress;GO:0016043//cellular component organization;GO:0044238//primary metabolic process;GO:0044763//single-organism cellular process;GO:0030198//extracellular matrix organization;GO:0071704//organic substance metabolic process;GO:0010467//gene expression;GO:0043170//macromolecule metabolic process;GO:0008150//biological_process;GO:0048856//anatomical structure development;GO:0009987//cellular process;GO:0032502//developmental process;GO:0071840//cellular component organization or biogenesis;GO:0051604//protein maturation;GO:0048869//cellular developmental process;GO:0043062//extracellular structure organization;GO:0008152//metabolic process;GO:0044699//single-organism process;GO:0050896//response to stimulus;GO:0030154//cell differentiation;GO:0019538//protein metabolic process;GO:0044767//single-organism developmental process

id	CON_Mean	KD_Mean	AllMean	log2FoldChange	pvalue	qvalue	State	gene_id	XF_1_count	XF_1_FPKM	XF_1_TPM	XF_1_CPM	XF_2_count	XF_2_FPKM	XF_2_TPM	XF_2_CPM	XF_3_count	XF_3_FPKM	XF_3_TPM	XF_3_CPM	Symbol	Description	KEGG_A_class	KEGG_B_class	Pathway	K_ID	GO Component	GO Function	GO Process
PLEC	9.431880037	10.663295488	10.047587763	1.245733975	3.40661E-05	0.004649886	Up	ENSG00000178209	23078	40.942089895	154.585548202	695.687991502	55302	108.430956369	435.472280496	1842.458810624	41310	82.456128132	331.712763233	1401.094529213	PLEC	plectin [Source:HGNC Symbol;Acc:HGNC:9069]	-	-	-	-	GO:0071944//cell periphery;GO:0005615//extracellular space;GO:0005886//plasma membrane;GO:0005575//cellular_component;GO:0005737//cytoplasm;GO:0043228//non-membrane-bounded organelle;GO:0016020//membrane;GO:0044424//intracellular part;GO:0005829//cytosol;GO:0044444//cytoplasmic part;GO:0044464//cell part;GO:0005856//cytoskeleton;GO:0005622//intracellular;GO:0043232//intracellular non-membrane-bounded organelle;GO:0043229//intracellular organelle;GO:0043226//organelle;GO:0044421//extracellular region part;GO:0005576//extracellular region;GO:0005623//cell	GO:0003676//nucleic acid binding;GO:0005488//binding;GO:1901363//heterocyclic compound binding;GO:0003723//RNA binding;GO:0003674//molecular_function;GO:0005515//protein binding;GO:0097159//organic cyclic compound binding;GO:0008092//cytoskeletal protein binding;GO:0005198//structural molecule activity	GO:0050896//response to stimulus;GO:0007010//cytoskeleton organization;GO:0006996//organelle organization;GO:0034330//cell junction organization;GO:0009987//cellular process;GO:0044699//single-organism process;GO:0008150//biological_process;GO:0016043//cellular component organization;GO:0044763//single-organism cellular process;GO:0071840//cellular component organization or biogenesis;GO:0006950//response to stress;GO:0022607//cellular component assembly;GO:0044085//cellular component biogenesis
C15orf39	3.92116951	5.435261408	4.678215459	1.542209238	3.61466E-05	0.004894709	Up	ENSG00000167173	503	2.611154064	9.858966248	15.162971649	1526	8.755070698	35.16145878	50.840695545	1061	6.196918724	24.92958474	35.985507032	C15orf39	chromosome 15 open reading frame 39 [Source:HGNC Symbol;Acc:HGNC:24497]	-	-	-	-	GO:0044424//intracellular part;GO:0044464//cell part;GO:0005737//cytoplasm;GO:0005575//cellular_component;GO:0005623//cell;GO:0005829//cytosol;GO:0044444//cytoplasmic part;GO:0005622//intracellular	-	-

id	CON_Mean	KD_Mean	AllMean	log2FoldChange	pvalue	qvalue	State	gene_id	XF_1_count	XF_1_FPKM	XF_1_TPM	XF_1_CPM	XF_2_count	XF_2_FPKM	XF_2_TPM	XF_2_CPM	XF_3_count	XF_3_FPKM	XF_3_TPM	XF_3_CPM	Symbol	Description	KEGG_A_class	KEGG_B_class	Pathway	K_ID	GO Component	GO Function	GO Process
ALPK1	5.483622812	4.117701926	4.800662369	-1.36835	3.74951E-05	0.005021472	Down	ENSG00000073331	1492	3.648908706	13.7772291 08	44.97644871	483	1.305515154	5.243112116	16.0917797 82	536	1.474873827	5.933270013	18.1792947 87	ALPK1	alpha kinase 1 [Source:HGNC Symbol;Acc:HGNC:20917]	-	-	-	-	GO:0005737//cytoplasm;GO:0005623//cell;GO:0005829//cytosol;GO:0044464//cell part;GO:0044424//intracellular part;GO:0005622//intracellular;GO:0005575//cellular_component;GO:0044444//cytoplasmic part	GO:0005488//binding;GO:0016772//transferase activity, transferring phosphorus-containing groups;GO:0016301//kinase activity;GO:0003674//molecular_function;GO:0016740//transferase activity;GO:0043167//ion binding;GO:0003824//catalytic activity	GO:0050789//regulation of biological process;GO:0044238//primary metabolic process;GO:0044699//single-organism process;GO:0044267//cellular protein metabolic process;GO:0007165//signal transduction;GO:0043412//macromolecule modification;GO:0071704//organic substance metabolic process;GO:0006950//response to stress;GO:0044700//single organism signaling;GO:0002376//immune system process;GO:0065007//biological regulation;GO:0051716//cellular response to stimulus;GO:0023052//signaling;GO:0036211//protein modification process;GO:0006464//cellular protein modification process;GO:0044260//cellular macromolecule metabolic process;GO:0009987//cellular process;GO:0007154//cell communication;GO:0008150//biological_process;GO:0043170//macromolecule metabolic process;GO:0050896//response to stimulus;GO:0044237//cellular metabolic process;GO:0044763//single-organism cellular process;GO:0050794//regulation of cellular [illegible]
SLC6A10P	2.752868318	4.593279032	3.673073675	1.857133201	3.76789E-05	0.005021472	Up	ENSG00000214617	222	1.494129532	5.6414031	6.692206175	674	5.013439563	20.1346002 37	22.4551958 04	745	5.641407254	22.6948176 09	25.2678630 9	-	-	-	-	-	-	-	-	-

id	CON_Mean	KD_Mean	AllMean	log2FoldChange	pvalue	qvalue	State	gene_id	XF_1_count	XF_1_FPKM	XF_1_TPM	XF_1_CPM	XF_2_count	XF_2_FPKM	XF_2_TPM	XF_2_CPM	XF_3_count	XF_3_FPKM	XF_3_TPM	XF_3_CPM	Symbol	Description	KEGG_A_class	KEGG_B_class	Pathway	K_ID	GO Component	GO Function	GO Process
SRPK3	0.117597975	2.724948399	1.421273187	2.716741812	3.79656E-05	0.005021472	Up	ENSG00000184343	33	0.209693804	0.791743452	0.994787404	191	1.341360881	5.387073042	6.363416022	193	1.379827738	5.550909095	6.545902787	SRPK3	SRSF protein kinase 3 [Source:HGNC Symbol;Acc:HGNC:11402]	-	-	-	-	GO:0005623//cell;GO:0005622//intracellular;GO:0043231//intracellular membrane-bounded organelle;GO:0043229//intracellular organelle;GO:0005737//cytoplasm;GO:0044424//intracellular part;GO:0044464//cell part;GO:0005575//cellular_component;GO:0043226//organelle;GO:0005634//nucleus;GO:0043227//membrane-bounded organelle	GO:0003824//catalytic activity;GO:0016772//transferase activity, transferring phosphorus-containing groups;GO:0016301//kinase activity;GO:0003674//molecular_function;GO:0005488//binding;GO:0043167//ion binding;GO:0016740//transferase activity	GO:0090304//nucleic acid metabolic process;GO:0044699//single-organism process;GO:0044267//cellular protein metabolic process;GO:0051716//cellular response to stimulus;GO:0065003//macromolecular complex assembly;GO:0007154//cell communication;GO:0036211//protein modification process;GO:0006397//mRNA processing;GO:0044260//cellular macromolecule metabolic process;GO:0043412//macromolecule modification;GO:0071826//ribonucleoprotein complex subunit organization;GO:0010467//gene expression;GO:0071704//organic substance metabolic process;GO:0008150//biological_process;GO:0050794//regulation of cellular process;GO:0022613//ribonucleoprotein complex biogenesis;GO:0046483//heterocycle metabolic process;GO:0034622//cellular macromolecular complex assembly;GO:0044085//cellular component biogenesis;GO:0016071//mRNA metabolic process;GO:0065007//biological regulation;GO:0006725//cellular aromatic compound metabolic process;GO:0071840//cellular component organization
SMIM14	5.369211045	3.497599255	4.43340515	-1.7932	3.9186E-05	0.005143015	Down	ENSG00000163683	1378	6.364319044	24.029837044	41.539910403	233	1.189321468	4.776463744	7.76270122	467	2.42669676	9.762358552	15.839049749	SMIM14	small integral membrane protein 14 [Source:HGNC Symbol;Acc:HGNC:27321]	-	-	-	-	GO:0044444//cytoplasmic part;GO:0044464//cell part;GO:0043231//intracellular membrane-bounded organelle;GO:0005575//cellular_component;GO:0005737//cytoplasm;GO:0043229//intracellular organelle;GO:0005622//intracellular;GO:0043226//organelle;GO:0005783//endoplasmic reticulum;GO:0005623//cell;GO:0012505//endomembrane system;GO:0044424//intracellular part;GO:0043227//membrane-bounded organelle	GO:0003674//molecular_function	GO:0044767//single-organism developmental process;GO:0048856//anatomical structure development;GO:0032502//developmental process;GO:0009790//embryo development;GO:0008150//biological_process;GO:0007275//multicellular organism development;GO:0044699//single-organism process;GO:0032501//multicellular organismal process;GO:0044707//single-multicellular organism process

id	CON_Mean	KD_Mean	AllMean	log2FoldChange	pvalue	qvalue	State	gene_id	XF_1_count	XF_1_FPKM	XF_1_TPM	XF_1_CPM	XF_2_count	XF_2_FPKM	XF_2_TPM	XF_2_CPM	XF_3_count	XF_3_FPKM	XF_3_TPM	XF_3_CPM	Symbol	Description	KEGG_A_class	KEGG_B_class	Pathway	K_ID	GO Component	GO Function	GO Process
MBD6	5.166572478	6.347067392	5.756819935	1.183739025	4.03066E-05	0.005249709	Up	ENSG00000166987	1197	6.482869604	24.477449837	36.083652215	2507	15.006108484	60.266408246	83.523999823	2291	13.960280017	56.160811392	77.702918577	MBD6	methyl-CpG binding domain protein 6 [Source:HGNC Symbol;Acc:HGNC:20445]	-	-	-	-	GO:0044446//intracellular organelle part;GO:0005654//nucleoplasm;GO:0005730//nucleolus;GO:0005575//cellular_component;GO:0005634//nucleus;GO:0043231//intracellular membrane-bounded organelle;GO:0043226//organelle;GO:0043233//organelle lumen;GO:0070013//intracellular organelle lumen;GO:0005694//chromosome;GO:0031981//nuclear lumen;GO:0043232//intracellular non-membrane-bounded organelle;GO:0031974//membrane-enclosed lumen;GO:0005622//intracellular;GO:0043227//membrane-bounded organelle;GO:0005623//cell;GO:0044428//nuclear part;GO:0044422//organelle part;GO:0043228//non-membrane-bounded organelle;GO:0043229//intracellular organelle;GO:0044424//intracellular part;GO:0044464//cell part	GO:0097159//organic cyclic compound binding;GO:0005488//binding;GO:0003677//DNA binding;GO:0003676//nucleic acid binding;GO:1901363//heterocyclic compound binding;GO:0003674//molecular_function	GO:0036211//protein modification process;GO:0008150//biological_process;GO:0019538//protein metabolic process;GO:0043170//macromolecule metabolic process;GO:0008152//metabolic process;GO:0043412//macromolecule modification;GO:0044260//cellular macromolecule metabolic process;GO:0044267//cellular protein metabolic process;GO:0006464//cellular protein modification process;GO:0009987//cellular process;GO:0044237//cellular metabolic process;GO:0071704//organic substance metabolic process;GO:0044238//primary metabolic process

id	CON_Mean	KD_Mean	AllMean	log2FoldChange	pvalue	qvalue	State	gene_id	XF_1_count	XF_1_FPKM	XF_1_TPM	XF_1_CPM	XF_2_count	XF_2_FPKM	XF_2_TPM	XF_2_CPM	XF_3_count	XF_3_FPKM	XF_3_TPM	XF_3_CPM	Symbol	Description	KEGG_A_class	KEGG_B_class	Pathway	K_ID	GO Component	GO Function	GO Process
NRN1	5.160549978	3.549564941	4.35505746	-1.60199	4.08278E-05	0.005277304	Down	ENSG00000124785	1192	15.7186906661	59.349252055	35.932926851	295	4.299349389	17.266724797	9.828312704	397	5.890152282	23.695493999	13.464888117	NRN1	neuritin 1 [Source:HGNC Symbol;Acc:HGNC:17972]	-	-	-	-	GO:0032991//macromolecular complex;GO:0044421//extracellular region part;GO:0005576//extracellular region;GO:0005615//extracellular space;GO:0044464//cell part;GO:0016020//membrane;GO:0071944//cell periphery;GO:0005623//cell;GO:0005575//cellular_component;GO:0005886//plasma membrane	GO:0003674//molecular_function	GO:0007267//cell-cell signaling;GO:0006810//transport;GO:0009987//cellular process;GO:0051179//localization;GO:0009653//anatomical structure morphogenesis;GO:0048856//anatomical structure development;GO:0023052//signaling;GO:0016043//cellular component organization;GO:0044767//single-organism developmental process;GO:0016192//vesicle-mediated transport;GO:0048869//cellular developmental process;GO:0030154//cell differentiation;GO:0040007//growth;GO:0032502//developmental process;GO:0008150//biological_process;GO:0044763//single-organism cellular process;GO:0032989//cellular component morphogenesis;GO:0071840//cellular component organization or biogenesis;GO:0007154//cell communication;GO:0044700//single organism signaling;GO:0051234//establishment of localization;GO:0044699//single-organism process;GO:0000902//cell morphogenesis
MSTRG.18665	4.610323986	2.590194987	3.600259487	-1.98729	4.15712E-05	0.005332987	Down	MSTRG.18665	813	3.661727811	13.825630358	24.507944237	229	1.139912739	4.578032111	7.629435963	133	0.673973869	2.71132952	4.510907102	-	-	-	-	-	-	-	-	-
PI15	4.711007329	6.112527143	5.411767236	1.444474723	4.1972E-05	0.005343966	Up	ENSG00000137558	872	3.671812199	13.863706108	26.286503535	2592	12.062562723	48.444760367	86.355886535	1600	7.580186004	30.494330737	54.266551603	PI15	peptidase inhibitor 15 [Source:HGNC Symbol;Acc:HGNC:8946]	-	-	-	-	GO:0044421//extracellular region part;GO:0005576//extracellular region;GO:0043226//organelle;GO:0005575//cellular_component;GO:0005615//extracellular space	GO:0098772//molecular function regulator;GO:0003674//molecular_function;GO:0030234//enzyme regulator activity	GO:0048856//anatomical structure development;GO:0008150//biological_process;GO:0032502//developmental process
LENG8	7.040161816	8.186355388	7.613258602	1.151703771	4.22832E-05	0.005343966	Up	ENSG00000167615	4395	20.547083627	77.57987427	132.487595227	7942	41.035695994	164.804486787	264.598167769	9276	48.791925081	196.285038587	314.610332921	LENG8	leukocyte receptor cluster member 8 [Source:HGNC Symbol;Acc:HGNC:15500]	-	-	-	-	GO:0005575//cellular_component;GO:0005623//cell;GO:0044464//cell part;GO:0005634//nucleus;GO:0043231//intracellular membrane-bounded organelle;GO:0044424//intracellular part;GO:0005622//intracellular;GO:0043226//organelle;GO:0043227//membrane-bounded organelle;GO:0043229//intracellular organelle	GO:0003674//molecular_function	-

id	CON_Mean	KD_Mean	AllMean	log2FoldChange	pvalue	qvalue	State	gene_id	XF_1_count	XF_1_FPKM	XF_1_TPM	XF_1_CPM	XF_2_count	XF_2_FPKM	XF_2_TPM	XF_2_CPM	XF_3_count	XF_3_FPKM	XF_3_TPM	XF_3_CPM	Symbol	Description	KEGG_A_class	KEGG_B_class	Pathway	K_ID	GO Component	GO Function	GO Process
TMEM106B	6.551813499	5.495049683	6.023431591	-1.05817	4.43821E-05	0.005566796	Down	ENSG00000106460	3132	6.822833372	25.761055183	94.414368203	1314	3.16358122	12.705337798	43.777636924	1339	3.281855786	13.202577857	45.414320373	TMEM106B	transmembrane protein 106B [Source:HGNC Symbol;Acc:HGNC:22407]	-	-	-	-	GO:0044424//intracellular part;GO:0043226//organelle;GO:0043231//intracellular membrane-bounded organelle;GO:0005737//cytoplasm;GO:0012505//endomembrane system;GO:0005575//cellular_component;GO:0043229//intracellular organelle;GO:0031982//vesicle;GO:0000323//lytic vacuole;GO:0043227//membrane-bounded organelle;GO:0005768//endosome;GO:0097708//intracellular vesicle;GO:0005622//intracellular;GO:0031410//cytoplasmic vesicle;GO:0044444//cytoplasmic part;GO:0005764//lysosome;GO:0005623//cell;GO:0005773//vacuole;GO:0044464//cell part	GO:0003674//molecular_function	GO:0016043//cellular component organization;GO:0007034//vacuolar transport;GO:0009653//anatomical structure morphogenesis;GO:0048856//anatomical structure development;GO:0032502//developmental process;GO:0044699//single-organism process;GO:0048869//cellular developmental process;GO:0051234//establishment of localization;GO:0044763//single-organism cellular process;GO:0032989//cellular component morphogenesis;GO:0030154//cell differentiation;GO:0000902//cell morphogenesis;GO:0071840//cellular component organization or biogenesis;GO:0009987//cellular process;GO:0051179//localization;GO:0008150//biological_process;GO:0044767//single-organism developmental process;GO:0006810//transport

id	CON_Mean	KD_Mean	AllMean	log2FoldChange	pvalue	qvalue	State	gene_id	XF_1_count	XF_1_FPKM	XF_1_TPM	XF_1_CPM	XF_2_count	XF_2_FPKM	XF_2_TPM	XF_2_CPM	XF_3_count	XF_3_FPKM	XF_3_TPM	XF_3_CPM	Symbol	Description	KEGG_A_class	KEGG_B_class	Pathway	K_ID	GO Component	GO Function	GO Process
DGKZ	5.674909696	6.800427769	6.237668733	1.12731551	4.48867E-05	0.005566796	Up	ENSG00000149091	1704	4.761954589	17.979770027	51.367204156	3226	9.963699802	40.015464406	107.478429769	3340	10.501661859	42.247136177	113.281426472	DGKZ	diacylglycerol kinase zeta [Source:HGNC Symbol;Acc:HGNC:2857]	Metabolism; Human Diseases;Environmental Information Processing	Cancers;Lipid metabolism; Signal transduction	ko00564//Glycerophospholipid metabolism;ko05231//Choline metabolism in cancer;ko04070//Phosphatidylinositol signaling system;ko00561//Glycerolipid metabolism	K00901	GO:0005886//plasma membrane;GO:0043233//organelle lumen;GO:0005575//cellular_component;GO:0043229//intracellular organelle;GO:0005737//cytoplasm;GO:0044424//intracellular part;GO:0031974//membrane-enclosed lumen;GO:0044464//cell part;GO:0031981//nuclear lumen;GO:0070013//intracellular organelle lumen;GO:0044428//nuclear part;GO:0043231//intracellular membrane-bounded organelle;GO:0043227//membrane-bounded organelle;GO:0005623//cell;GO:0044422//organelle part;GO:0071944//cell periphery;GO:0044446//intracellular organelle part;GO:0043226//organelle;GO:0005634//nucleus;GO:0005622//intracellular;GO:0005654//nucleoplasm;GO:0016020//membrane	GO:0016772//transferase activity, transferring phosphorus-containing groups;GO:0043167//ion binding;GO:0016301//kinase activity;GO:0003824//catalytic activity;GO:0003674//molecular_function;GO:0005488//binding;GO:0016740//transferase activity	GO:0051716//cellular response to stimulus;GO:0044699//single-organism process;GO:0050794//regulation of cellular process;GO:0040011//locomotion;GO:0007267//cell-cell signaling;GO:0006928//movement of cell or subcellular component;GO:0048870//cell motility;GO:0044238//primary metabolic process;GO:0006629//lipid metabolic process;GO:0009987//cellular process;GO:0007154//cell communication;GO:0050789//regulation of biological process;GO:0044700//single organism signaling;GO:0051179//localization;GO:0051674//localization of cell;GO:0065007//biological regulation;GO:0044763//single-organism cellular process;GO:0050896//response to stimulus;GO:0008152//metabolic process;GO:0071704//organic substance metabolic process;GO:0023052//signaling;GO:0007165//signal transduction;GO:0044710//single-organism metabolic process;GO:0009058//biosynthetic process;GO:0006950//response to stress;GO:0008150//biological_process

id	CON_Mean	KD_Mean	AllMean	log2FoldChange	pvalue	qvalue	State	gene_id	XF_1_count	XF_1_FPKM	XF_1_TPM	XF_1_CPM	XF_2_count	XF_2_FPKM	XF_2_TPM	XF_2_CPM	XF_3_count	XF_3_FPKM	XF_3_TPM	XF_3_CPM	Symbol	Description	KEGG_A_class	KEGG_B_class	Pathway	K_ID	GO Component	GO Function	GO Process
MR1	4.110513635	2.15743077	3.133972	-1.97099	4.50251E-05	0.005566796	Down	ENSG00000153029	574	1.83277956	6.920048138	17.3032718 23	119	0.419938714	1.686526389	3.964641396	139	0.499354589	2.008853607	4.714406671	MR1	major histocompatibility complex, class I-related [Source:HGNC Symbol;Acc:HGNC:4975]	-	-	-	-	GO:0032991//macromolecular complex;GO:0005623//cell;GO:0005575//cellular_component;GO:0005576//extracellular region;GO:0071944//cell periphery;GO:0043227//membrane-bounded organelle;GO:0044421//extracellular region part;GO:0044424//intracellular part;GO:0005622//intracellular;GO:0043231//intracellular membrane-bounded organelle;GO:0005783//endoplasmic reticulum;GO:0043229//intracellular organelle;GO:0043226//organelle;GO:0044444//cytoplasmic part;GO:0005615//extracellular space;GO:0005886//plasma membrane;GO:0016020//membrane;GO:0005737//cytoplasm;GO:0044464//cell part;GO:0012505//endomembrane system	GO:0003674//molecular_function	GO:0008150//biological_process;GO:0050896//response to stimulus;GO:0002376//immune system process;GO:0006950//response to stress

id	CON_Mean	KD_Mean	AllMean	log2FoldChange	pvalue	qvalue	State	gene_id	XF_1_count	XF_1_FPKM	XF_1_TPM	XF_1_CPM	XF_2_count	XF_2_FPKM	XF_2_TPM	XF_2_CPM	XF_3_count	XF_3_FPKM	XF_3_TPM	XF_3_CPM	Symbol	Description	KEGG_A_class	KEGG_B_class	Pathway	K_ID	GO Component	GO Function	GO Process
UHRF1	6.63452473	7.835537522	7.235031126	1.218353739	4.6698E-05	0.005732091	Up	ENSG00000276043	3317	19.4346836767	73.3795954 71	99.991206682	7875	50.994358545	204.799720962	262.365974715	5751	37.91143565	152.513916947	195.05433642	UHRF1	ubiquitin like with PHD and ring finger domains 1 [Source:HGNC Symbol;Acc:HGNC:12556]	-	-	-	-	GO:0043227//membrane-bounded organelle;GO:0031981//nuclear lumen;GO:0000228//nuclear chromosome;GO:0043233//organelle lumen;GO:0043229//intracellular organelle;GO:0005575//cellular_component;GO:0005694//chromosome;GO:0005654//nucleoplasm;GO:0043228//non-membrane-bounded organelle;GO:0043231//intracellular membrane-bounded organelle;GO:0043232//intracellular non-membrane-bounded organelle;GO:0044424//intracellular part;GO:0044428//nuclear part;GO:0044422//organelle part;GO:0005623//cell;GO:0043226//organelle;GO:0031974//membrane-enclosed lumen;GO:0005634//nucleus;GO:0005622//intracellular;GO:0044464//cell part;GO:0070013//intracellular organelle lumen;GO:0044446//intracellular organelle part	GO:1901363//heterocyclic compound binding;GO:0042393//histone binding;GO:0003676//nucleic acid binding;GO:0005515//protein binding;GO:0003674//molecular_function;GO:0005488//binding;GO:0043167//ion binding;GO:0003677//DNA binding;GO:0097159//organic cyclic compound binding	GO:0071704//organic substance metabolic process;GO:0006996//organelle organization;GO:0043170//macromolecule metabolic process;GO:0044267//cellular protein metabolic process;GO:0044238//primary metabolic process;GO:0044260//cellular macromolecule metabolic process;GO:0019538//protein metabolic process;GO:0006725//cellular aromatic compound metabolic process;GO:0006807//nitrogen compound metabolic process;GO:0090304//nucleic acid metabolic process;GO:0008150//biological_process;GO:0050896//response to stimulus;GO:0006259//DNA metabolic process;GO:0016043//cellular component organization;GO:0008283//cell proliferation;GO:0034641//cellular nitrogen compound metabolic process;GO:0051276//chromosome organization;GO:0044699//single-organism process;GO:0046483//heterocycle metabolic process;GO:0006950//response to stress;GO:0008152//metabolic process;GO:1901360//organic cyclic compound metabolic process;GO:0009058//bios [illegible]
BMS1P12	-0.40399	-2.76323	-1.58361	-3.59178	4.71652E-05	0.005748089	Down	ENSG00000224599	22	0.9393648 77	3.5467714 23	0.6631916 03	2	0.0943804 94	0.3790438 65	0.0666326 28	1	0.0480405 02	0.1932621 41	0.0339165 95	-	-	-	-	-	-	-	-	-

id	CON_Mean	KD_Mean	AllMean	log2FoldChange	pvalue	qvalue	State	gene_id	XF_1_count	XF_1_FPKM	XF_1_TPM	XF_1_CPM	XF_2_count	XF_2_FPKM	XF_2_TPM	XF_2_CPM	XF_3_count	XF_3_FPKM	XF_3_TPM	XF_3_CPM	Symbol	Description	KEGG_A_class	KEGG_B_class	Pathway	K_ID	GO Component	GO Function	GO Process
ATXN2L	7.248983122	8.394159935	7.821572	1.151446355	4.93659E-05	0.005973619	Up	ENSG00000168488	5080	23.401126243	88.355917789	153.136970137	10900	55.49324959	222.867830004	363.147825319	9016	46.728609151	187.984524799	305.792018286	ATXN2L	ataxin 2 like [Source:HGNC Symbol;Acc:HGNC:31326]	-	-	-	-	GO:0044446//intracellular organelle part;GO:0043231//intracellular membrane-bounded organelle;GO:0005634//nucleus;GO:0005737//cytoplasm;GO:0070013//intracellular organelle lumen;GO:0044444//cytoplasmic part;GO:0031974//membrane-enclosed lumen;GO:0005622//intracellular;GO:0005654//nucleoplasm;GO:0044422//organelle part;GO:0043226//organelle;GO:0044428//nuclear part;GO:0043227//membrane-bounded organelle;GO:0005829//cytosol;GO:0005623//cell;GO:0043233//organelle lumen;GO:0031981//nuclear lumen;GO:0043229//intracellular organelle;GO:0044464//cell part;GO:0044424//intracellular part;GO:0005575//cellular_component	GO:0097159//organic cyclic compound binding;GO:1901363//heterocyclic compound binding;GO:0003674//molecular_function;GO:0003676//nucleic acid binding;GO:0005488//binding;GO:0003723//RNA binding	GO:0044085//cellular component biogenesis;GO:0009987//cellular process;GO:0016043//cellular component organization;GO:0022607//cellular component assembly;GO:0008150//biological_process;GO:0071840//cellular component organization or biogenesis
CLUH	6.803664686	7.974814066	7.389239376	1.184157288	5.12718E-05	0.006160564	Up	ENSG00000132361	3730	16.29581475	61.528306517	112.441121774	8494	41.012865686	164.712797416	282.988773235	6468	31.793120994	127.900548517	219.372534857	CLUH	clustered mitochondria homolog [Source:HGNC Symbol;Acc:HGNC:29094]	-	-	-	-	GO:0005623//cell;GO:0044424//intracellular part;GO:0005737//cytoplasm;GO:0044464//cell part;GO:0005575//cellular_component;GO:0005622//intracellular	GO:0005488//binding;GO:0003723//RNA binding;GO:0044822//poly(A) RNA binding;GO:0003729//mRNA binding;GO:0003676//nucleic acid binding;GO:0097159//organic cyclic compound binding;GO:0003674//molecular_function;GO:1901363//heterocyclic compound binding	GO:0008150//biological_process;GO:0009987//cellular process;GO:0006996//organelle organization;GO:0007005//mitochondrion organization;GO:0071840//cellular component organization or biogenesis;GO:0016043//cellular component organization

id	CON_Mean	KD_Mean	AllMean	log2FoldChange	pvalue	qvalue	State	gene_id	XF_1_count	XF_1_FPKM	XF_1_TPM	XF_1_CPM	XF_2_count	XF_2_FPKM	XF_2_TPM	XF_2_CPM	XF_3_count	XF_3_FPKM	XF_3_TPM	XF_3_CPM	Symbol	Description	KEGG_A_class	KEGG_B_class	Pathway	K_ID	GO Component	GO Function	GO Process
NOTCH3	3.246950037	4.856322509	4.051636273	1.619704603	5.21244E-05	0.006219202	Up	ENSG00000074181	314	1.00611744	3.798809891	9.465552879	883	3.126945736	12.5582051 12	29.4183054 82	820	2.956165784	11.8923595 25	27.8116076 97	NOTCH3	notch receptor 3 [Source:HGNC Symbol;Acc:HGNC:7883]	Human Diseases;Environmental Information Processing;Organismal Systems	Development;Cancers;Endocrine system;Signal transduction	ko05206//MicroRNAs in cancer;ko04919//Thyroid hormone signaling pathway;ko04330//Notch signaling pathway;ko04320//Dorso-ventral axis formation	K02599	GO:0043228//non-membrane-bounded organelle;GO:0043232//intracellular non-membrane-bounded organelle;GO:0005654//nucleoplasm;GO:0032991//macromolecular complex;GO:0071944//cell periphery;GO:0043227//membrane-bounded organelle;GO:0044464//cell part;GO:0005856//cytoskeleton;GO:0043233//organelle lumen;GO:0005623//cell;GO:0043226//organelle;GO:0043231//intracellular membrane-bounded organelle;GO:0005886//plasma membrane;GO:0044424//intracellular part;GO:0044444//cytoplasmic part;GO:0031974//membrane-enclosed lumen;GO:0016020//membrane;GO:0005634//nucleus;GO:0044422//organelle part;GO:0005575//cellular_component;GO:0070013//intracellular organelle lumen;GO:0031981//nuclear lumen;GO:0005794//Golgi apparatus;GO:0005622//intracellular;GO:0005576//extracellular region;GO:0005737//cytoplasm;GO:0005829//cytosol;GO:0044446//intracellular organelle part;GO:0043229//intracellular organelle;GO:0044428//nuclear part;GO:0012505//endomembrane system	GO:0005488//binding;GO:0019899//enzyme binding;GO:0005515//protein binding;GO:0003674//molecular_function;GO:0043167//ion binding	GO:0008152//metabolic process;GO:0009653//anatomical structure morphogenesis;GO:0008150//biological_process;GO:0048856//anatomical structure development;GO:0008283//cell proliferation;GO:0050896//response to stimulus;GO:0032502//developmental process;GO:0023052//signaling;GO:0009058//biosynthetic process;GO:0006807//nitrogen compound metabolic process;GO:0044700//single organism signaling;GO:0048646//anatomical structure formation involved in morphogenesis;GO:0030154//cell differentiation;GO:0034641//cellular nitrogen compound metabolic process;GO:0007165//signal transduction;GO:0051716//cellular response to stimulus;GO:0044237//cellular metabolic process;GO:0009987//cellular process;GO:0050789//regulation of biological process;GO:0044699//single-organism process;GO:0065007//biological regulation;GO:0050794//regulation of cellular process;GO:0044767//single-organism developmental process;GO:0044763//single-organism cellular [illegible]

id	CON_Mean	KD_Mean	AllMean	log2FoldChange	pvalue	qvalue	State	gene_id	XF_1_count	XF_1_FPKM	XF_1_TPM	XF_1_CPM	XF_2_count	XF_2_FPKM	XF_2_TPM	XF_2_CPM	XF_3_count	XF_3_FPKM	XF_3_TPM	XF_3_CPM	Symbol	Description	KEGG_A_class	KEGG_B_class	Pathway	K_ID	GO Component	GO Function	GO Process
BCOR	5.951645044	7.079692841	6.515668942	1.131958255	5.48719E-05	0.006501557	Up	ENSG00000183337	2065	6.246821421	23.586199834	62.249575459	3766	12.59099242	50.567000111	125.469239464	4215	14.346055884	57.712749149	142.958446688	BCOR	BCL6 corepressor [Source:HGNC Symbol;Acc:HGNC:20893]	-	-	-	-	GO:0044428//nuclear part;GO:0044446//intracellular organelle part;GO:0031974//membrane-enclosed lumen;GO:0070013//intracellular organelle lumen;GO:0043233//organelle lumen;GO:0043231//intracellular membrane-bounded organelle;GO:0005634//nucleus;GO:0044464//cell part;GO:0043226//organelle;GO:0043227//membrane-bounded organelle;GO:0031981//nuclear lumen;GO:0005622//intracellular;GO:0043229//intracellular organelle;GO:0044422//organelle part;GO:0005623//cell;GO:0032991//macromolecular complex;GO:0044424//intracellular part;GO:0005654//nucleoplasm;GO:0005575//cellular_component	GO:0019899//enzyme binding;GO:0005488//binding;GO:0003677//DNA binding;GO:0008134//transcription factor binding;GO:1901363//heterocyclic compound binding;GO:0003676//nucleic acid binding;GO:0003674//molecular_function;GO:0097159//organic cyclic compound binding;GO:0005515//protein binding	GO:0019538//protein metabolic process;GO:0007275//multicellular organism development;GO:0043170//macromolecule metabolic process;GO:0044238//primary metabolic process;GO:0044260//cellular macromolecule metabolic process;GO:0009058//biosynthetic process;GO:0036211//protein modification process;GO:0006464//cellular protein modification process;GO:0009790//embryo development;GO:0044699//single-organism process;GO:0032502//developmental process;GO:0009987//cellular process;GO:0006807//nitrogen compound metabolic process;GO:0048856//anatomical structure development;GO:0034641//cellular nitrogen compound metabolic process;GO:0044707//single-multicellular organism process;GO:0051276//chromosome organization;GO:0043412//macromolecule modification;GO:0071840//cellular component organization or biogenesis;GO:0044767//single-organism developmental process;GO:0032501//multicellular organismal process;GO:0071704//organic substance metabolic [illegible]

id	CON_Mean	KD_Mean	AllMean	log2FoldChange	pvalue	qvalue	State	gene_id	XF_1_count	XF_1_FPKM	XF_1_TPM	XF_1_CPM	XF_2_count	XF_2_FPKM	XF_2_TPM	XF_2_CPM	XF_3_count	XF_3_FPKM	XF_3_TPM	XF_3_CPM	Symbol	Description	KEGG_A_class	KEGG_B_class	Pathway	K_ID	GO Component	GO Function	GO Process
SLC7A5	8.718114459	9.972003929	9.345059194	1.279236349	5.89596E-05	0.006937715	Up	ENSG00000103257	14070	89.0304733 76	336.153444261	424.141175164	35886	250.963319302	1007.900074267	1195.589253157	24414	173.811869076	699.227779433	828.03974428	SLC7A5	solute carrier family 7 member 5 [Source:HGNC Symbol;Acc:HGNC:11063]	Human Diseases	Cancers	ko05230//Central carbon metabolism in cancer	K13780	GO:0044464//cell part;GO:0005829//cytosol;GO:0005773//vacuole;GO:0071944//cell periphery;GO:0044424//intracellular part;GO:0005764//lysosome;GO:0000323//lytic vacuole;GO:0043231//intracellular membrane-bounded organelle;GO:0005623//cell;GO:0043229//intracellular organelle;GO:0043227//membrane-bounded organelle;GO:0005575//cellular_component;GO:0044421//extracellular region part;GO:0005737//cytoplasm;GO:0005886//plasma membrane;GO:0005576//extracellular region;GO:0044444//cytoplasmic part;GO:0043226//organelle;GO:0005622//intracellular;GO:0005615//extracellular space;GO:0032991//macromolecular complex;GO:0016020//membrane	GO:0005215//transporter activity;GO:0003674//molecular_function;GO:0022857//transmembrane transporter activity	GO:0055085//transmembrane transport;GO:0008150//biological_process;GO:0040011//locomotion;GO:0002376//immune system process;GO:0051234//establishment of localization;GO:0006928//movement of cell or subcellular component;GO:0051179//localization;GO:0051674//localization of cell;GO:0009987//cellular process;GO:0044763//single-organism cellular process;GO:0044699//single-organism process;GO:0006810//transport;GO:0048870//cell motility
GNE	8.749849491	7.748121301	8.248985396	-1.00149	6.30374E-05	0.007366746	Down	ENSG00000159921	14383	74.0397170 37	279.552662704	433.57658297	6194	35.2392845 73	141.525373657	206.361250461	6477	37.5132828 23	150.912187912	219.67778421	GNE	glucosamine (UDP-N-acetyl)-2-epimerase/N-acetylmannosamine kinase [Source:HGNC Symbol;Acc:HGNC:23657]	Metabolism	Carbohydrate metabolism	ko00520//Amino sugar and nucleotide sugar metabolism	K12409	GO:0005575//cellular_component;GO:0005737//cytoplasm;GO:0005829//cytosol;GO:0044464//cell part;GO:0005623//cell;GO:0005622//intracellular;GO:0044424//intracellular part;GO:0044444//cytoplasmic part	GO:0016787//hydrolase activity;GO:0016772//transferase activity, transferring phosphorus-containing groups;GO:0016740//transferase activity;GO:0003824//catalytic activity;GO:0016301//kinase activity;GO:0043167//ion binding;GO:0016798//hydrolase activity, acting on glycosyl bonds;GO:0005488//binding;GO:0003674//molecular_function;GO:0016853//isomerase activity	GO:0071704//organic substance metabolic process;GO:0044237//cellular metabolic process;GO:0034641//cellular nitrogen compound metabolic process;GO:0009987//cellular process;GO:0044281//small molecule metabolic process;GO:0044238//primary metabolic process;GO:0008152//metabolic process;GO:0044710//single-organism metabolic process;GO:0008150//biological_process;GO:0005975//carbohydrate metabolic process;GO:0022610//biological adhesion;GO:0044699//single-organism process;GO:0009058//biosynthetic process;GO:0007155//cell adhesion;GO:0006807//nitrogen compound metabolic process
ENSG00000280441	-1.03964	2.802213731	0.881284704	4.72505155	6.39456E-05	0.007422039	Up	ENSG00000280441	13	0.0891663 13	0.3366663 37	0.3918859 47	540	4.0934720 58	16.4398956 9	17.9908096 95	75	0.57878148	2.3283800 55	2.5437446 06	-	-	-	-	-	-	-	-	-

id	CON_Mean	KD_Mean	AllMean	log2FoldChange	pvalue	qvalue	State	gene_id	XF_1_count	XF_1_FPKM	XF_1_TPM	XF_1_CPM	XF_2_count	XF_2_FPKM	XF_2_TPM	XF_2_CPM	XF_3_count	XF_3_FPKM	XF_3_TPM	XF_3_CPM	Symbol	Description	KEGG_A_class	KEGG_B_class	Pathway	K_ID	GO Component	GO Function	GO Process
NCOA7	6.634959178	5.598739388	6.116849283	-1.03763	6.82983E-05	0.007873688	Down	ENSG00000111912	3318	13.079815843	49.385620219	100.021351755	1427	6.217128342	24.968764899	47.542380434	1424	6.31584032	25.407994435	48.297230927	NCOA7	nuclear receptor coactivator 7 [Source:HGNC Symbol;Acc:HGNC:21081]	-	-	-	-	GO:0044424//intracellular part;GO:0043227//membrane-bounded organelle;GO:0005575//cellular_component;GO:0043226//organelle;GO:0044464//cell part;GO:0005623//cell;GO:0005622//intracellular;GO:0005634//nucleus;GO:0043231//intracellular membrane-bounded organelle;GO:0043229//intracellular organelle	GO:0008134//transcription factor binding;GO:0005515//protein binding;GO:0003674//molecular_function;GO:0005488//binding	GO:0071704//organic substance metabolic process;GO:0006950//response to stress;GO:0034641//cellular nitrogen compound metabolic process;GO:0008152//metabolic process;GO:0009058//biosynthetic process;GO:0009987//cellular process;GO:0044260//cellular macromolecule metabolic process;GO:0008150//biological_process;GO:0036211//protein modification process;GO:0006464//cellular protein modification process;GO:0044238//primary metabolic process;GO:0043170//macromolecule metabolic process;GO:0043412//macromolecule modification;GO:0044267//cellular protein metabolic process;GO:0008219//cell death;GO:0019538//protein metabolic process;GO:0006807//nitrogen compound metabolic process;GO:0044237//cellular metabolic process;GO:0044699//single-organism process;GO:0044763//single-organism cellular process;GO:0050896//response to stimulus

id	CON_Mean	KD_Mean	AllMean	log2FoldChange	pvalue	qvalue	State	gene_id	XF_1_count	XF_1_FPKM	XF_1_TPM	XF_1_CPM	XF_2_count	XF_2_FPKM	XF_2_TPM	XF_2_CPM	XF_3_count	XF_3_FPKM	XF_3_TPM	XF_3_CPM	Symbol	Description	KEGG_A_class	KEGG_B_class	Pathway	K_ID	GO Component	GO Function	GO Process
TMEM109	6.625808188	7.705637056	7.165722622	1.081497751	6.98378E-05	0.007997128	Up	ENSG00000110108	3297	46.5083318 79	175.602075952	99.3883052 25	5962	92.9489310 04	373.294530541	198.631865555	6344	100.686418862	405.051401	215.166877108	TMEM109	transmembrane protein 109 [Source:HGNC Symbol;Acc:HGNC:28771]	-	-	-	-	GO:0031967//organelle envelope;GO:0031975//envelope;GO:0043231//intracellular membrane-bounded organelle;GO:0044424//intracellular part;GO:0044464//cell part;GO:0043226//organelle;GO:0005623//cell;GO:0044428//nuclear part;GO:0043227//membrane-bounded organelle;GO:0005575//cellular_component;GO:0005783//endoplasmic reticulum;GO:0005635//nuclear envelope;GO:0012505//endomembrane system;GO:0005634//nucleus;GO:0005615//extracellular space;GO:0043229//intracellular organelle;GO:0005576//extracellular region;GO:0044446//intracellular organelle part;GO:0005737//cytoplasm;GO:0005622//intracellular;GO:0044421//extracellular region part;GO:0044444//cytoplasmic part;GO:0044422//organelle part	GO:0022857//transmembrane transporter activity;GO:0003674//molecular_function;GO:0005215//transporter activity	GO:0009987//cellular process;GO:0051234//establishment of localization;GO:0006810//transport;GO:0008150//biological_process;GO:0051716//cellular response to stimulus;GO:0006950//response to stress;GO:0023052//signaling;GO:0050896//response to stimulus;GO:0007154//cell communication;GO:0050794//regulation of cellular process;GO:0050789//regulation of biological process;GO:0007165//signal transduction;GO:0044763//single-organism cellular process;GO:0008219//cell death;GO:0044700//single organism signaling;GO:0055085//transmembrane transport;GO:0051179//localization;GO:0065007//biological regulation;GO:0044699//single-organism process

id	CON_Mean	KD_Mean	AllMean	log2FoldChange	pvalue	qvalue	State	gene_id	XF_1_count	XF_1_FPKM	XF_1_TPM	XF_1_CPM	XF_2_count	XF_2_FPKM	XF_2_TPM	XF_2_CPM	XF_3_count	XF_3_FPKM	XF_3_TPM	XF_3_CPM	Symbol	Description	KEGG_A_class	KEGG_B_class	Pathway	K_ID	GO Component	GO Function	GO Process
NACC2	5.83548501	6.929353737	6.382419374	1.095663652	7.09229E-05	0.008067239	Up	ENSG00000148411	1905	7.958198975	30.0478689938	57.426363802	3511	16.2103075557	65.1026223011	116.973579933	3670	17.249709396	69.3938569908	124.473902741	NACC2	NACC family member 2 [Source:HGNC Symbol;Acc:HGNC:23846]	-	-	-	-	GO:0005730//nucleolus;GO:0043228//non-membrane-bounded organelle;GO:0043227//membrane-bounded organelle;GO:0044444//cytoplasmic part;GO:0031974//membrane-enclosed lumen;GO:0031981//nuclear lumen;GO:0005694//chromosome;GO:0005737//cytoplasm;GO:0043231//intracellular membrane-bounded organelle;GO:0044446//intracellular organelle part;GO:0044424//intracellular part;GO:0005623//cell;GO:0044464//cell part;GO:0000228//nuclear chromosome;GO:0044428//nuclear part;GO:0043226//organelle;GO:0005634//nucleus;GO:0043233//organelle lumen;GO:0043232//intracellular non-membrane-bounded organelle;GO:0043229//intracellular organelle;GO:0070013//intracellular organelle lumen;GO:0005622//intracellular;GO:0044422//organelle part;GO:0005739//mitochondrion;GO:0005575//cellular_component	GO:1901363//heterocyclic compound binding;GO:0097159//organic cyclic compound binding;GO:0019899//enzyme binding;GO:0005488//binding;GO:0005515//protein binding;GO:0003700//transcription factor activity, sequence-specific DNA binding;GO:0003677//DNA binding;GO:0001071//nucleic acid binding transcription factor activity;GO:0003674//molecular_function;GO:0003676//nucleic acid binding	GO:0044700//single organism signaling;GO:0006950//response to stress;GO:0034641//cellular nitrogen compound metabolic process;GO:0009058//biosynthetic process;GO:0043933//macromolecular complex subunit organization;GO:0050789//regulation of biological process;GO:0008283//cell proliferation;GO:0044763//single-organism cellular process;GO:0044237//cellular metabolic process;GO:0050896//response to stimulus;GO:0000278//mitotic cell cycle;GO:0007154//cell communication;GO:0022607//cellular component assembly;GO:0051716//cellular response to stimulus;GO:0009987//cellular process;GO:0007165//signal transduction;GO:0044699//single-organism process;GO:0023052//signaling;GO:0065007//biological regulation;GO:0008152//metabolic process;GO:0008219//cell death;GO:0006807//nitrogen compound metabolic process;GO:0071840//cellular component organization or biogenesis;GO:0050794//regulation of cellular process;GO:0044085//cellu[illegible]

id	CON_Mean	KD_Mean	AllMean	log2FoldChange	pvalue	qvalue	State	gene_id	XF_1_count	XF_1_FPKM	XF_1_TPM	XF_1_CPM	XF_2_count	XF_2_FPKM	XF_2_TPM	XF_2_CPM	XF_3_count	XF_3_FPKM	XF_3_TPM	XF_3_CPM	Symbol	Description	KEGG_A_class	KEGG_B_class	Pathway	K_ID	GO Component	GO Function	GO Process
EIF4G1	8.521263966	9.645971901	9.083617933	1.130861434	7.49434E-05	0.008468111	Up	ENSG00000114867	12275	42.279566885	159.635476386	370.030769377	25996	98.959198495	397.432516393	866.090905229	21446	83.10960821	334.341648284	727.375291055	EIF4G1	eukaryotic translation initiation factor 4 gamma 1 [Source:HGNC Symbol;Acc:HGNC:3296]	Human Diseases;Genetic Information Processing	Translation;Cardiovascular diseases	ko03013//RNA transport;ko05416//Viral myocarditis	K03260	GO:0043231//intracellular membrane-bounded organelle;GO:0043227//membrane-bounded organelle;GO:0005622//intracellular;GO:0005575//cellular_component;GO:0044464//cell part;GO:0005634//nucleus;GO:0005829//cytosol;GO:0044444//cytoplasmic part;GO:0043226//organelle;GO:0005737//cytoplasm;GO:0043229//intracellular organelle;GO:0044424//intracellular part;GO:0032991//macromolecular complex;GO:0005623//cell	GO:0005488//binding;GO:0003723//RNA binding;GO:0003729//mRNA binding;GO:0008135//translation factor activity, RNA binding;GO:0003676//nucleic acid binding;GO:1901363//heterocyclic compound binding;GO:0097159//organic cyclic compound binding;GO:0043167//ion binding;GO:0003674//molecular_function;GO:0044822//poly(A) RNA binding	GO:0048869//cellular developmental process;GO:0044249//cellular biosynthetic process;GO:0071840//cellular component organization or biogenesis;GO:0044238//primary metabolic process;GO:0006807//nitrogen compound metabolic process;GO:0009987//cellular process;GO:0044763//single-organism cellular process;GO:0008152//metabolic process;GO:0044085//cellular component biogenesis;GO:0019538//protein metabolic process;GO:1901575//organic substance catabolic process;GO:0043933//macromolecular complex subunit organization;GO:0008219//cell death;GO:0044237//cellular metabolic process;GO:0016043//cellular component organization;GO:0034655//nucleobase-containing compound catabolic process;GO:1901361//organic cyclic compound catabolic process;GO:0044419//interspecies interaction between organisms;GO:0006464//cellular protein modification process;GO:0034641//cellular nitrogen compound metabolic process;GO:0006518//peptide metabolic [illegible]

id	CON_Mean	KD_Mean	AllMean	log2FoldChange	pvalue	qvalue	State	gene_id	XF_1_count	XF_1_FPKM	XF_1_TPM	XF_1_CPM	XF_2_count	XF_2_FPKM	XF_2_TPM	XF_2_CPM	XF_3_count	XF_3_FPKM	XF_3_TPM	XF_3_CPM	Symbol	Description	KEGG_A_class	KEGG_B_class	Pathway	K_ID	GO Component	GO Function	GO Process
SLC5A6	5.527336813	6.646739558	6.087038185	1.12457437	7.63632E-05	0.008571766	Up	ENSG00000138074	1538	8.296908028	31.326736842	46.363122061	3183	18.977420948	76.215695727	106.045828256	2735	16.600194461	66.780923378	92.761886647	SLC5A6	solute carrier family 5 member 6 [Source:HGNC Symbol;Acc:HGNC:11041]	Organismal Systems	Digestive system	ko04977//Vitamin digestion and absorption	K14386	GO:0005623//cell;GO:0043226//organelle;GO:0005575//cellular_component;GO:0005886//plasma membrane;GO:0016020//membrane;GO:0071944//cell periphery;GO:0044464//cell part	GO:0022857//transmembrane transporter activity;GO:0005215//transporter activity;GO:0003674//molecular_function	GO:0051179//localization;GO:0044281//small molecule metabolic process;GO:0051234//establishment of localization;GO:0044699//single-organism process;GO:0055085//transmembrane transport;GO:0008150//biological_process;GO:0044710//single-organism metabolic process;GO:0006790//sulfur compound metabolic process;GO:0034641//cellular nitrogen compound metabolic process;GO:0008152//metabolic process;GO:0044237//cellular metabolic process;GO:0009987//cellular process;GO:0051186//cofactor metabolic process;GO:0006807//nitrogen compound metabolic process;GO:0006810//transport
ENSG00000224888	-1.33397	1.389584597	0.027807796	3.07000901	7.89951E-05	0.008809243	Up	ENSG00000224888	10	0.062841511	0.23727146	0.301450729	85	0.590345364	2.370900807	2.831886711	65	0.459574455	1.848822104	2.204578659	-	-	-	-	-	-	-	-	-

id	CON_Mean	KD_Mean	AllMean	log2FoldChange	pvalue	qvalue	State	gene_id	XF_1_count	XF_1_FPKM	XF_1_TPM	XF_1_CPM	XF_2_count	XF_2_FPKM	XF_2_TPM	XF_2_CPM	XF_3_count	XF_3_FPKM	XF_3_TPM	XF_3_CPM	Symbol	Description	KEGG_A_class	KEGG_B_class	Pathway	K_ID	GO Component	GO Function	GO Process
ADM	3.458088583	4.963842595	4.210965589	1.513742728	8.11725E-05	0.008977984	Up	ENSG00000148926	364	4.719486676	17.819423408	10.972806522	923	13.226218517	53.118147555	30.750958052	911	13.28947003	53.462209849	30.898017819	ADM	adrenomedullin [Source:HGNC Symbol;Acc:HGNC:259]	Organismal Systems	Circulatory system	ko04270//Vascular smooth muscle contraction	K12333	GO:0005615//extracellular space;GO:0044464//cell part;GO:0005737//cytoplasm;GO:0044424//intracellular part;GO:0005576//extracellular region;GO:0005622//intracellular;GO:0005575//cellular_component;GO:0044421//extracellular region part;GO:0005623//cell	GO:0003674//molecular_function	GO:0044700//single organism signaling;GO:0009790//embryo development;GO:0050896//response to stimulus;GO:0051716//cellular response to stimulus;GO:0000003//reproduction;GO:0050789//regulation of biological process;GO:0042592//homeostatic process;GO:0008152//metabolic process;GO:0008150//biological_process;GO:0050794//regulation of cellular process;GO:0048869//cellular developmental process;GO:0040007//growth;GO:0009653//anatomical structure morphogenesis;GO:0044699//single-organism process;GO:0003008//system process;GO:0044238//primary metabolic process;GO:0009987//cellular process;GO:0048856//anatomical structure development;GO:0007165//signal transduction;GO:0006810//transport;GO:0007568//aging;GO:0065007//biological regulation;GO:0023052//signaling;GO:0044710//single-organism metabolic process;GO:0044763//single-organism cellular process;GO:0007267//cell-cell signaling;GO:0016192//vesicle-mediated transport;GO:0032502//dev[illegible]
C15orf54	1.641433981	3.945545451	2.793489716	2.403332204	8.17395E-05	0.008977984	Up	ENSG00000175746	101	0.989165809	3.734805409	3.044652359	613	6.635120414	26.647473331	20.422900635	332	3.658320162	14.717056417	11.260309458	-	-	-	-	-	-	-	-	-

id	CON_Mean	KD_Mean	AllMean	log2FoldChange	pvalue	qvalue	State	gene_id	XF_1_count	XF_1_FPKM	XF_1_TPM	XF_1_CPM	XF_2_count	XF_2_FPKM	XF_2_TPM	XF_2_CPM	XF_3_count	XF_3_FPKM	XF_3_TPM	XF_3_CPM	Symbol	Description	KEGG_A_class	KEGG_B_class	Pathway	K_ID	GO Component	GO Function	GO Process
PKMYT1	4.806663587	6.085764664	5.446214126	1.306254639	8.20868E-05	0.008977984	Up	ENSG00000127564	932	5.648413331	21.326783108	28.095207907	2416	16.182592526	64.991315283	80.492215227	1654	11.278256478	45.371298719	56.09804772	PKMYT1	protein kinase, membrane associated tyrosine/threonine 1 [Source:HGNC Symbol;Acc:HGNC:29650]	Cellular Processes;Organismal Systems	Cell growth and death;Endocrine system	ko04110//Cell cycle;ko04114//Oocyte meiosis;ko04914//Progesterone-mediated oocyte maturation	K06633	GO:0043232//intracellular non-membrane-bounded organelle;GO:0031974//membrane-enclosed lumen;GO:0043229//intracellular organelle;GO:0005737//cytoplasm;GO:0043233//organelle lumen;GO:0043231//intracellular membrane-bounded organelle;GO:0005623//cell;GO:0005575//cellular_component;GO:0005634//nucleus;GO:0044464//cell part;GO:0005622//intracellular;GO:0012505//endomembrane system;GO:0070013//intracellular organelle lumen;GO:0044444//cytoplasmic part;GO:0031981//nuclear lumen;GO:0044422//organelle part;GO:0005783//endoplasmic reticulum;GO:0044424//intracellular part;GO:0043227//membrane-bounded organelle;GO:0044446//intracellular organelle part;GO:0043228//non-membrane-bounded organelle;GO:0044428//nuclear part;GO:0043226//organelle;GO:0005794//Golgi apparatus;GO:0005829//cytosol;GO:0005730//nucleolus;GO:0005654//nucleoplasm	GO:0043167//ion binding;GO:0016772//transferase activity, transferring phosphorus-containing groups;GO:0005488//binding;GO:0016740//transferase activity;GO:0003824//catalytic activity;GO:0003674//molecular_function;GO:0016301//kinase activity	GO:0071704//organic substance metabolic process;GO:0007049//cell cycle;GO:0044237//cellular metabolic process;GO:0044267//cellular protein metabolic process;GO:0006464//cellular protein modification process;GO:0008150//biological_process;GO:0008152//metabolic process;GO:0043170//macromolecule metabolic process;GO:0036211//protein modification process;GO:0009987//cellular process;GO:0044238//primary metabolic process;GO:0044260//cellular macromolecule metabolic process;GO:0043412//macromolecule modification;GO:0019538//protein metabolic process;GO:0044699//single-organism process;GO:0044763//single-organism cellular process;GO:0000278//mitotic cell cycle

id	CON_Mean	KD_Mean	AllMean	log2FoldChange	pvalue	qvalue	State	gene_id	XF_1_count	XF_1_FPKM	XF_1_TPM	XF_1_CPM	XF_2_count	XF_2_FPKM	XF_2_TPM	XF_2_CPM	XF_3_count	XF_3_FPKM	XF_3_TPM	XF_3_CPM	Symbol	Description	KEGG_A_class	KEGG_B_class	Pathway	K_ID	GO Component	GO Function	GO Process
CHTF18	5.231211945	6.379279519	5.805245732	1.158176388	8.32343E-05	0.009045499	Up	ENSG00000127586	1252	6.379586076	24.087480959	37.741631223	2740	15.430476885	61.970724671	91.286701044	2192	12.566797785	50.554971629	74.345175697	CHTF18	chromosome transmission fidelity factor 18 [Source:HGNC Symbol;Acc:HGNC:18435]	-	-	-	-	GO:0032991//macromolecular complex;GO:0044422//organelle part;GO:0043226//organelle;GO:0044464//cell part;GO:0005829//cytosol;GO:0005654//nucleoplasm;GO:0005623//cell;GO:0005634//nucleus;GO:0043227//membrane-bounded organelle;GO:0005575//cellular_component;GO:0005622//intracellular;GO:0043231//intracellular membrane-bounded organelle;GO:0005737//cytoplasm;GO:0044424//intracellular part;GO:0031974//membrane-enclosed lumen;GO:0044446//intracellular organelle part;GO:0044428//nuclear part;GO:0043232//intracellular non-membrane-bounded organelle;GO:0044444//cytoplasmic part;GO:0070013//intracellular organelle lumen;GO:0043233//organelle lumen;GO:0043228//non-membrane-bounded organelle;GO:0031981//nuclear lumen;GO:0005694//chromosome;GO:0043229//intracellular organelle	GO:0005488//binding;GO:0016787//hydrolase activity;GO:0004386//helicase activity;GO:0017111//nucleoside-triphosphatase activity;GO:0097159//organic cyclic compound binding;GO:1901363//heterocyclic compound binding;GO:0016817//hydrolase activity, acting on acid anhydrides;GO:0016818//hydrolase activity, acting on acid anhydrides, in phosphorus-containing anhydrides;GO:0003824//catalytic activity;GO:0016462//pyrophosphatase activity;GO:0003676//nucleic acid binding;GO:0003677//DNA binding;GO:0016887//ATPase activity;GO:0003674//molecular_function;GO:0043167//ion binding	GO:0008150//biological_process;GO:0051276//chromosome organization;GO:0007049//cell cycle;GO:0006259//DNA metabolic process;GO:0090304//nucleic acid metabolic process;GO:1901360//organic cyclic compound metabolic process;GO:0034641//cellular nitrogen compound metabolic process;GO:0008152//metabolic process;GO:0006996//organelle organization;GO:0046483//heterocycle metabolic process;GO:0044237//cellular metabolic process;GO:0043170//macromolecule metabolic process;GO:0006807//nitrogen compound metabolic process;GO:0006725//cellular aromatic compound metabolic process;GO:0071704//organic substance metabolic process;GO:0044260//cellular macromolecule metabolic process;GO:0044699//single-organism process;GO:0044238//primary metabolic process;GO:0016043//cellular component organization;GO:0044763//single-organism cellular process;GO:0006139//nucleobase-containing compound metabolic process;GO:0071840//cellular component organization

id	CON_Mean	KD_Mean	AllMean	log2FoldChange	pvalue	qvalue	State	gene_id	XF_1_count	XF_1_FPKM	XF_1_TPM	XF_1_CPM	XF_2_count	XF_2_FPKM	XF_2_TPM	XF_2_CPM	XF_3_count	XF_3_FPKM	XF_3_TPM	XF_3_CPM	Symbol	Description	KEGG_A_class	KEGG_B_class	Pathway	K_ID	GO Component	GO Function	GO Process
YPEL5	5.876478587	4.454239565	5.165359076	-1.38067	8.62372E-05	0.009312527	Down	ENSG00000119801	1960	17.271073607	65.210603278	59.084342809	502	4.88885991	19.634272771	16.724789753	824	8.169328873	32.864393657	27.947274076	YPEL5	yippee like 5 [Source:HGNC Symbol;Acc:HGNC:18329]	-	-	-	-	GO:0043232//intracellular non-membrane-bounded organelle;GO:0044424//intracellular part;GO:0032991//macromolecular complex;GO:0031982//vesicle;GO:0005622//intracellular;GO:0044430//cytoskeletal part;GO:0044464//cell part;GO:0015630//microtubule cytoskeleton;GO:0043228//non-membrane-bounded organelle;GO:0005576//extracellular region;GO:0043231//intracellular membrane-bounded organelle;GO:0005737//cytoplasm;GO:0005623//cell;GO:0044422//organelle part;GO:0043229//intracellular organelle;GO:0043226//organelle;GO:0005856//cytoskeleton;GO:0097708//intracellular vesicle;GO:0005634//nucleus;GO:0005815//microtubule organizing center;GO:0044444//cytoplasmic part;GO:0031410//cytoplasmic vesicle;GO:0044446//intracellular organelle part;GO:0005575//cellular_component;GO:0043227//membrane-bounded organelle	GO:0043167//ion binding;GO:0003674//molecular_function;GO:0005488//binding	GO:0051179//localization;GO:0008283//cell proliferation;GO:0044699//single-organism process;GO:0051234//establishment of localization;GO:0006810//transport;GO:0008150//biological_process;GO:0002376//immune system process;GO:0016192//vesicle-mediated transport

id	CON_Mean	KD_Mean	AllMean	log2FoldChange	pvalue	qvalue	State	gene_id	XF_1_count	XF_1_FPKM	XF_1_TPM	XF_1_CPM	XF_2_count	XF_2_FPKM	XF_2_TPM	XF_2_CPM	XF_3_count	XF_3_FPKM	XF_3_TPM	XF_3_CPM	Symbol	Description	KEGG_A_class	KEGG_B_class	Pathway	K_ID	GO Component	GO Function	GO Process
CARD9	0.699159506	3.109087636	1.904123571	2.495966313	9.07589E-05	0.009739177	Up	ENSG00000187796	51	0.229496748	0.866513671	1.537398716	294	1.462157992	5.872209345	9.794996389	215	1.088530806	4.379050652	7.292067872	CARD9	caspase recruitment domain family member 9 [Source:HGNC Symbol;Acc:HGNC:16391]	Organismal Systems;Human Diseases	Immune system;Infectious diseases	ko05152//Tuberculosis;ko04621//NOD-like receptor signaling pathway	K12794	GO:0005623//cell;GO:0005829//cytosol;GO:0005886//plasma membrane;GO:0044464//cell part;GO:0071944//cell periphery;GO:0044424//intracellular part;GO:0044444//cytoplasmic part;GO:0005575//cellular_component;GO:0005737//cytoplasm;GO:0016020//membrane;GO:0032991//macromolecular complex;GO:0005622//intracellular	GO:0003674//molecular_function	GO:0036211//protein modification process;GO:0009058//biosynthetic process;GO:0043412//macromolecule modification;GO:0007165//signal transduction;GO:0043170//macromolecule metabolic process;GO:0009987//cellular process;GO:0006464//cellular protein modification process;GO:0050896//response to stimulus;GO:0051716//cellular response to stimulus;GO:0050789//regulation of biological process;GO:0044238//primary metabolic process;GO:0044700//single organism signaling;GO:0044763//single-organism cellular process;GO:0044267//cellular protein metabolic process;GO:0023052//signaling;GO:0002376//immune system process;GO:0071704//organic substance metabolic process;GO:0019538//protein metabolic process;GO:0006950//response to stress;GO:0008219//cell death;GO:0065007//biological regulation;GO:0044237//cellular metabolic process;GO:0008152//metabolic process;GO:0050794//regulation of cellular process;GO:0007154//cell communication;GO:000815[illegible]

id	CON_Mean	KD_Mean	AllMean	log2FoldChange	pvalue	qvalue	State	gene_id	XF_1_count	XF_1_FPKM	XF_1_TPM	XF_1_CPM	XF_2_count	XF_2_FPKM	XF_2_TPM	XF_2_CPM	XF_3_count	XF_3_FPKM	XF_3_TPM	XF_3_CPM	Symbol	Description	KEGG_A_class	KEGG_B_class	Pathway	K_ID	GO Component	GO Function	GO Process
WDR62	4.699422759	5.964053728	5.331738243	1.288511283	9.1815E-05	0.009747239	Up	ENSG00000075702	865	2.134360974	8.058732765	26.075488025	2190	5.972229533	23.985220656	72.962728206	1541	4.278093846	17.210344011	52.265472513	WDR62	WD repeat domain 62 [Source:HGNC Symbol;Acc:HGNC:24502]	-	-	-	-	GO:0044464//cell part;GO:0044424//intracellular part;GO:0005856//cytoskeleton;GO:0043232//intracellular non-membrane-bounded organelle;GO:0005815//microtubule organizing center;GO:0044444//cytoplasmic part;GO:0005737//cytoplasm;GO:0044422//organelle part;GO:0043227//membrane-bounded organelle;GO:0043226//organelle;GO:0043229//intracellular organelle;GO:0005623//cell;GO:0015630//microtubule cytoskeleton;GO:0005829//cytosol;GO:0005622//intracellular;GO:0005634//nucleus;GO:0043228//non-membrane-bounded organelle;GO:0005575//cellular_component;GO:0044446//intracellular organelle part;GO:0043231//intracellular membrane-bounded organelle;GO:0044430//cytoskeletal part	GO:0003674//molecular_function	GO:0044085//cellular component biogenesis;GO:0044767//single-organism developmental process;GO:0022607//cellular component assembly;GO:0030154//cell differentiation;GO:0044699//single-organism process;GO:0006996//organelle organization;GO:0008150//biological_process;GO:0009987//cellular process;GO:0000278//mitotic cell cycle;GO:0007049//cell cycle;GO:0007010//cytoskeleton organization;GO:0071840//cellular component organization or biogenesis;GO:0032502//developmental process;GO:0044763//single-organism cellular process;GO:0048856//anatomical structure development;GO:0048869//cellular developmental process;GO:0016043//cellular component organization

id	CON_Mean	KD_Mean	AllMean	log2FoldChange	pvalue	qvalue	State	gene_id	XF_1_count	XF_1_FPKM	XF_1_TPM	XF_1_CPM	XF_2_count	XF_2_FPKM	XF_2_TPM	XF_2_CPM	XF_3_count	XF_3_FPKM	XF_3_TPM	XF_3_CPM	Symbol	Description	KEGG_A_class	KEGG_B_class	Pathway	K_ID	GO Component	GO Function	GO Process
HGS	6.508845952	7.567742975	7.038294464	1.060176978	9.19766E-05	0.009747239	Up	ENSG00000185359	3040	8.446955618	31.893273357	91.6410215	5770	17.719156901	71.162349965	192.235133219	5414	16.92547184	68.089481767	183.624443988	HGS	hepatocyte growth factor-regulated tyrosine kinase substrate [Source:HGNC Symbol;Acc:HGNC:4897]	Cellular Processes	Transport and catabolism	ko04144//Endocytosis;ko04145//Phagosome	K12182	GO:0044464//cell part;GO:0044424//intracellular part;GO:0031410//cytoplasmic vesicle;GO:0005829//cytosol;GO:0012505//endomembrane system;GO:0005622//intracellular;GO:0044444//cytoplasmic part;GO:0005623//cell;GO:0005773//vacuole;GO:0005737//cytoplasm;GO:0005764//lysosome;GO:0043226//organelle;GO:0031982//vesicle;GO:0043229//intracellular organelle;GO:0005576//extracellular region;GO:0005615//extracellular space;GO:0043231//intracellular membrane-bounded organelle;GO:0000323//lytic vacuole;GO:0097708//intracellular vesicle;GO:0044421//extracellular region part;GO:0043227//membrane-bounded organelle;GO:0032991//macromolecular complex;GO:0005575//cellular_component;GO:0005768//endosome	GO:0016772//transferase activity, transferring phosphorus-containing groups;GO:0016740//transferase activity;GO:0019899//enzyme binding;GO:0043167//ion binding;GO:0003824//catalytic activity;GO:0005515//protein binding;GO:0005488//binding;GO:0003674//molecular_function;GO:0016301//kinase activity	GO:0006605//protein targeting;GO:0032502//developmental process;GO:0016192//vesicle-mediated transport;GO:0009987//cellular process;GO:0044238//primary metabolic process;GO:0033036//macromolecule localization;GO:0070727//cellular macromolecule localization;GO:0051234//establishment of localization;GO:0071702//organic substance transport;GO:0051649//establishment of localization in cell;GO:0019538//protein metabolic process;GO:0048856//anatomical structure development;GO:0016043//cellular component organization;GO:0036211//protein modification process;GO:0009653//anatomical structure morphogenesis;GO:0009056//catabolic process;GO:0045184//establishment of protein localization;GO:0006464//cellular protein modification process;GO:0015031//protein transport;GO:0006914//autophagy;GO:0006886//intracellular protein transport;GO:0044267//cellular protein metabolic process;GO:0050896//response to stimulus;GO:0006810//transport;GO:0008152//metabolic process;GO:0044699//singl[illegible]

id	CON_Mean	KD_Mean	AllMean	log2FoldChange	pvalue	qvalue	State	gene_id	XF_1_count	XF_1_FPKM	XF_1_TPM	XF_1_CPM	XF_2_count	XF_2_FPKM	XF_2_TPM	XF_2_CPM	XF_3_count	XF_3_FPKM	XF_3_TPM	XF_3_CPM	Symbol	Description	KEGG_A_class	KEGG_B_class	Pathway	K_ID	GO Component	GO Function	GO Process
PKD1	5.975854735	7.050410213	6.513132474	1.075878185	9.49409E-05	0.009999273	Up	ENSG00000008710	2100	3.415595824	12.896306815	63.30465301	3990	7.172336994	28.805002299	132.932093855	3820	6.990471132	28.121966771	129.561391953	PKD1	polycystin 1, transient receptor potential channel interacting [Source:HGNC Symbol;Acc:HGNC:9008]	-	-	-	-	GO:0031982//vesicle;GO:0005929//cilium;GO:0016020//membrane;GO:0005634//nucleus;GO:0005622//intracellular;GO:0044464//cell part;GO:0043229//intracellular organelle;GO:0044421//extracellular region part;GO:0005623//cell;GO:0043231//intracellular membrane-bounded organelle;GO:0097708//intracellular vesicle;GO:0005737//cytoplasm;GO:0043226//organelle;GO:0044444//cytoplasmic part;GO:0005783//endoplasmic reticulum;GO:0042995//cell projection;GO:0005615//extracellular space;GO:0005576//extracellular region;GO:0012505//endomembrane system;GO:0043227//membrane-bounded organelle;GO:0005575//cellular_component;GO:0032991//macromolecular complex;GO:0044424//intracellular part;GO:0031410//cytoplasmic vesicle;GO:0071944//cell periphery;GO:0005794//Golgi apparatus;GO:0005886//plasma membrane	GO:0005515//protein binding;GO:0003674//molecular_function;GO:0005215//transporter activity;GO:0019899//enzyme binding;GO:0005488//binding;GO:0022857//transmembrane transporter activity	GO:0006996//organelle organization;GO:0044707//single-multicellular organism process;GO:0033036//macromolecule localization;GO:0065007//biological regulation;GO:0044699//single-organism process;GO:0051716//cellular response to stimulus;GO:0048856//anatomical structure development;GO:0006810//transport;GO:0051169//nuclear transport;GO:0044237//cellular metabolic process;GO:0065003//macromolecular complex assembly;GO:0006950//response to stress;GO:0009987//cellular process;GO:0007049//cell cycle;GO:0044700//single organism signaling;GO:0050789//regulation of biological process;GO:0015031//protein transport;GO:0034641//cellular nitrogen compound metabolic process;GO:0006807//nitrogen compound metabolic process;GO:0019538//protein metabolic process;GO:0045184//establishment of protein localization;GO:0051649//establishment of localization in cell;GO:0044763//single-organism cellular process;GO:0008150//biological_process;GO:0071702//organic substance transport;GO:0022607//cell [illegible]
ENSG00000223886	0.117597975	-2.11567	-0.99903	-2.82614	0.000105233	0.011015207	Down	ENSG00000223886	33	2.148568908	8.112377833	0.994787404	3	0.215872447	0.866970741	0.099948943	5	0.366269922	1.473467292	0.169582974	-	-	-	-	-	-	-	-	-

id	CON_Mean	KD_Mean	AllMean	log2FoldChange	pvalue	qvalue	State	gene_id	XF_1_count	XF_1_FPKM	XF_1_TPM	XF_1_CPM	XF_2_count	XF_2_FPKM	XF_2_TPM	XF_2_CPM	XF_3_count	XF_3_FPKM	XF_3_TPM	XF_3_CPM	Symbol	Description	KEGG_A_class	KEGG_B_class	Pathway	K_ID	GO Component	GO Function	GO Process
FAM114A1	6.384144705	5.080077585	5.732111145	-1.25911	0.0001092	0.011360757	Down	ENSG00000197712	2788	17.254047041	65.1463158 67	84.0444631 38	772	5.280269883	21.2062241 69	25.7201946 01	1280	8.91259316	35.8544716 04	43.4132412 83	FAM114A1	family with sequence similarity 114 member A1 [Source:HGNC Symbol;Acc:HGNC:25087]	-	-	-	-	GO:0005794//Golgi apparatus;GO:0005829//cytosol;GO:0044428//nuclear part;GO:0044446//intracellular organelle part;GO:0005737//cytoplasm;GO:0043229//intracellular organelle;GO:0031981//nuclear lumen;GO:0012505//endomembrane system;GO:0044422//organelle part;GO:0043233//organelle lumen;GO:0070013//intracellular organelle lumen;GO:0043227//membrane-bounded organelle;GO:0044444//cytoplasmic part;GO:0043231//intracellular membrane-bounded organelle;GO:0043226//organelle;GO:0005623//cell;GO:0044424//intracellular part;GO:0005634//nucleus;GO:0031974//membrane-enclosed lumen;GO:0005622//intracellular;GO:0005654//nucleoplasm;GO:0005575//cellular_component;GO:0044464//cell part	GO:0003674//molecular_function	-

id	CON_Mean	KD_Mean	AllMean	log2FoldChange	pvalue	qvalue	State	gene_id	XF_1_count	XF_1_FPKM	XF_1_TPM	XF_1_CPM	XF_2_count	XF_2_FPKM	XF_2_TPM	XF_2_CPM	XF_3_count	XF_3_FPKM	XF_3_TPM	XF_3_CPM	Symbol	Description	KEGG_A_class	KEGG_B_class	Pathway	K_ID	GO Component	GO Function	GO Process
RGS9	3.2695098	1.167453546	2.218481673	-2.1217	0.000109962	0.01137076	Down	ENSG00000108370	319	1.363817649	5.149382932	9.616278243	52	0.245702502	0.986771974	1.732448341	77	0.37038403	1.490017939	2.611577796	RGS9	regulator of G protein signaling 9 [Source:HGNC Symbol;Acc:HGNC:10004]	Human Diseases;Organismal Systems	Substance dependence;Sensory system	ko05030//Cocaine addiction;ko04744//Phototransduction	K13765	GO:0005623//cell;GO:0043226//organelle;GO:0005622//intracellular;GO:0043227//membrane-bounded organelle;GO:0016020//membrane;GO:0005634//nucleus;GO:0005575//cellular_component;GO:0005737//cytoplasm;GO:0005886//plasma membrane;GO:0044424//intracellular part;GO:0043229//intracellular organelle;GO:0071944//cell periphery;GO:0043231//intracellular membrane-bounded organelle;GO:0044464//cell part	GO:0016787//hydrolase activity;GO:0016817//hydrolase activity, acting on acid anhydrides;GO:0003674//molecular_function;GO:0016818//hydrolase activity, acting on acid anhydrides, in phosphorus-containing anhydrides;GO:0003924//GTPase activity;GO:0098772//molecular function regulator;GO:0003824//catalytic activity;GO:0017111//nucleoside-triphosphatase activity;GO:0030234//enzyme regulator activity;GO:0016462//pyrophosphatase activity	GO:0032501//multicellular organismal process;GO:0008150//biological_process;GO:0051234//establishment of localization;GO:0051179//localization;GO:0007154//cell communication;GO:0003008//system process;GO:0044700//single organism signaling;GO:0044763//single-organism cellular process;GO:0006810//transport;GO:0065007//biological regulation;GO:0050794//regulation of cellular process;GO:0044699//single-organism process;GO:0051716//cellular response to stimulus;GO:0050896//response to stimulus;GO:0007165//signal transduction;GO:0050789//regulation of biological process;GO:0006457//protein folding;GO:0055085//transmembrane transport;GO:0032502//developmental process;GO:0050877//neurological system process;GO:0048856//anatomical structure development;GO:0009987//cellular process;GO:0023052//signaling
AHDC1	5.335394419	6.412820923	5.874107671	1.081101435	0.00011432	0.011750137	Up	ENSG00000126705	1346	4.890354113	18.464569672	40.575268072	2655	10.661059941	42.816149931	88.454814332	2370	9.68811975	38.974337572	80.382329563	AHDC1	AT-hook DNA binding motif containing 1 [Source:HGNC Symbol;Acc:HGNC:25230]	-	-	-	-	-	GO:0005488//binding;GO:0003677//DNA binding;GO:1901363//heterocyclic compound binding;GO:0003676//nucleic acid binding;GO:0097159//organic cyclic compound binding;GO:0003674//molecular_function	-

id	CON_Mean	KD_Mean	AllMean	log2FoldChange	pvalue	qvalue	State	gene_id	XF_1_count	XF_1_FPKM	XF_1_TPM	XF_1_CPM	XF_2_count	XF_2_FPKM	XF_2_TPM	XF_2_CPM	XF_3_count	XF_3_FPKM	XF_3_TPM	XF_3_CPM	Symbol	Description	KEGG_A_class	KEGG_B_class	Pathway	K_ID	GO Component	GO Function	GO Process
NCLN	6.610426355	7.672210655	7.141318505	1.06633899	0.000117314	0.01198567	Up	ENSG00000125912	3262	23.552868904	88.928854404	98.333227675	6498	51.853750897	208.251148116	216.489409993	5557	45.143596895	181.608178892	188.474517038	NCLN	nicalin [Source:HGNC Symbol;Acc:HGNC:26923]	-	-	-	-	GO:0005783//endoplasmic reticulum;GO:0043226//organelle;GO:0043231//intracellular membrane-bounded organelle;GO:0044424//intracellular part;GO:0043227//membrane-bounded organelle;GO:0005737//cytoplasm;GO:0005622//intracellular;GO:0005575//cellular_component;GO:0012505//endomembrane system;GO:0044464//cell part;GO:0044444//cytoplasmic part;GO:0032991//macromolecular complex;GO:0043229//intracellular organelle;GO:0005623//cell	GO:0003674//molecular_function	GO:0044085//cellular component biogenesis;GO:0050896//response to stimulus;GO:0016043//cellular component organization;GO:0008150//biological_process;GO:0065003//macromolecular complex assembly;GO:0050794//regulation of cellular process;GO:0007154//cell communication;GO:0050789//regulation of biological process;GO:0051716//cellular response to stimulus;GO:0023052//signaling;GO:0009987//cellular process;GO:0044763//single-organism cellular process;GO:0044700//single organism signaling;GO:0071840//cellular component organization or biogenesis;GO:0065007//biological regulation;GO:0043933//macromolecular complex subunit organization;GO:0022607//cellular component assembly;GO:0044699//single-organism process;GO:0007165//signal transduction
ENSG00000226180	-1.33397	1.320704967	-0.00663	3.001087764	0.000119799	0.012166694	Up	ENSG00000226180	10	0.115454128	0.43592156	0.301450729	82	1.046318563	4.202146196	2.731937768	61	0.792383102	3.187678034	2.06891228	-	-	-	-	-	-	-	-	-

id	CON_Mean	KD_Mean	AllMean	log2FoldChange	pvalue	qvalue	State	gene_id	XF_1_count	XF_1_FPKM	XF_1_TPM	XF_1_CPM	XF_2_count	XF_2_FPKM	XF_2_TPM	XF_2_CPM	XF_3_count	XF_3_FPKM	XF_3_TPM	XF_3_CPM	Symbol	Description	KEGG_A_class	KEGG_B_class	Pathway	K_ID	GO Component	GO Function	GO Process
TGFBI	6.172416654	4.967467701	5.569942177	-1.18397	0.000121327	0.012176969	Down	ENSG00000120708	2407	9.402512683	35.501181824	72.559190378	771	3.328609341	13.36811137	25.686878286	1096	4.816973934	19.378204753	37.172587848	TGFBI	transforming growth factor beta induced [Source:HGNC Symbol;Acc:HGNC:11771]	-	-	-	-	GO:0043227//membrane-bounded organelle;GO:0005615//extracellular space;GO:0043226//organelle;GO:0005575//cellular_component;GO:0005737//cytoplasm;GO:0043231//intracellular membrane-bounded organelle;GO:0044464//cell part;GO:0005622//intracellular;GO:0012505//endomembrane system;GO:0005886//plasma membrane;GO:0044444//cytoplasmic part;GO:0044421//extracellular region part;GO:0044424//intracellular part;GO:0005794//Golgi apparatus;GO:0016020//membrane;GO:0031012//extracellular matrix;GO:0005623//cell;GO:0043229//intracellular organelle;GO:0071944//cell periphery;GO:0005576//extracellular region	GO:0005198//structural molecule activity;GO:0003674//molecular_function	GO:0022610//biological adhesion;GO:0030198//extracellular matrix organization;GO:0030154//cell differentiation;GO:0048856//anatomical structure development;GO:0003008//system process;GO:0008283//cell proliferation;GO:0048646//anatomical structure formation involved in morphogenesis;GO:0044767//single-organism developmental process;GO:0032501//multicellular organismal process;GO:0009653//anatomical structure morphogenesis;GO:0016043//cellular component organization;GO:0043062//extracellular structure organization;GO:0071840//cellular component organization or biogenesis;GO:0044763//single-organism cellular process;GO:0007155//cell adhesion;GO:0008150//biological_process;GO:0032502//developmental process;GO:0050877//neurological system process;GO:0044699//single-organism process;GO:0009987//cellular process;GO:0048869//cellular developmental process

id	CON_Mean	KD_Mean	AllMean	log2FoldChange	pvalue	qvalue	State	gene_id	XF_1_count	XF_1_FPKM	XF_1_TPM	XF_1_CPM	XF_2_count	XF_2_FPKM	XF_2_TPM	XF_2_CPM	XF_3_count	XF_3_FPKM	XF_3_TPM	XF_3_CPM	Symbol	Description	KEGG_A_class	KEGG_B_class	Pathway	K_ID	GO Component	GO Function	GO Process
NCDN	5.711620284	6.806160411	6.258890347	1.103173677	0.000123216	0.012294241	Up	ENSG00000020129	1748	10.716613252	40.462847371	52.693587362	3663	24.819536119	99.678360833	122.037659096	2965	20.452044629	82.27653167	100.56270344	NCDN	neurochondrin [Source:HGNC Symbol;Acc:HGNC:17597]	-	-	-	-	GO:0005622//intracellular;GO:0044464//cell part;GO:0031410//cytoplasmic vesicle;GO:0005773//vacuole;GO:0031982//vesicle;GO:0005575//cellular_component;GO:0005829//cytosol;GO:0097708//intracellular vesicle;GO:0012505//endomembrane system;GO:0043227//membrane-bounded organelle;GO:0043229//intracellular organelle;GO:0044444//cytoplasmic part;GO:0044424//intracellular part;GO:0005623//cell;GO:0005737//cytoplasm;GO:0043226//organelle;GO:0005768//endosome;GO:0043231//intracellular membrane-bounded organelle	GO:0003674//molecular_function	GO:0030154//cell differentiation;GO:0007267//cell-cell signaling;GO:0032502//developmental process;GO:0023052//signaling;GO:0065007//biological regulation;GO:0042592//homeostatic process;GO:0007154//cell communication;GO:0009987//cellular process;GO:0065008//regulation of biological quality;GO:0044763//single-organism cellular process;GO:0048856//anatomical structure development;GO:0044767//single-organism developmental process;GO:0044699//single-organism process;GO:0008150//biological_process;GO:0044700//single organism signaling;GO:0048869//cellular developmental process

id	CON_Mean	KD_Mean	AllMean	log2FoldChange	pvalue	qvalue	State	gene_id	XF_1_count	XF_1_FPKM	XF_1_TPM	XF_1_CPM	XF_2_count	XF_2_FPKM	XF_2_TPM	XF_2_CPM	XF_3_count	XF_3_FPKM	XF_3_TPM	XF_3_CPM	Symbol	Description	KEGG_A_class	KEGG_B_class	Pathway	K_ID	GO Component	GO Function	GO Process
PRPF19	7.615882349	8.687560482	8.151721415	1.075482276	0.000124832	0.012383022	Up	ENSG00000110107	6552	67.663760668	255.478886472	197.51051739	11398	130.092274688	522.466843699	379.739349815	12951	150.480924507	605.369721004	439.253818635	PRPF19	pre-mRNA processing factor 19 [Source:HGNC Symbol;Acc:HGNC:17896]	Genetic Information Processing	Transcription; Folding, sorting and degradation	ko03040//Spliceosome;ko04120//Ubiquitin mediated proteolysis	K10599	GO:0005634//nucleus;GO:0032991//macromolecular complex;GO:0044446//intracellular organelle part;GO:0005654//nucleoplasm;GO:0005856//cytoskeleton;GO:0043228//non-membrane-bounded organelle;GO:0000228//nuclear chromosome;GO:0005737//cytoplasm;GO:0043232//intracellular non-membrane-bounded organelle;GO:0044424//intracellular part;GO:0031974//membrane-enclosed lumen;GO:0044464//cell part;GO:0031981//nuclear lumen;GO:0005694//chromosome;GO:0005623//cell;GO:0005575//cellular_component;GO:0043231//intracellular membrane-bounded organelle;GO:0043229//intracellular organelle;GO:0043226//organelle;GO:0005811//lipid particle;GO:0005622//intracellular;GO:0044422//organelle part;GO:0043227//membrane-bounded organelle;GO:0043233//organelle lumen;GO:0070013//intracellular organelle lumen;GO:0044428//nuclear part	GO:0003674//molecular_function	GO:0016071//mRNA metabolic process;GO:0007165//signal transduction;GO:0009987//cellular process;GO:0044260//cellular macromolecule metabolic process;GO:0044267//cellular protein metabolic process;GO:0043933//macromolecular complex subunit organization;GO:0010467//gene expression;GO:0019538//protein metabolic process;GO:0008150//biological_process;GO:0030154//cell differentiation;GO:0050896//response to stimulus;GO:0044767//single-organism developmental process;GO:0043170//macromolecule metabolic process;GO:0032502//developmental process;GO:0006396//RNA processing;GO:0044237//cellular metabolic process;GO:0046483//heterocycle metabolic process;GO:0044710//single-organism metabolic process;GO:0006807//nitrogen compound metabolic process;GO:0048869//cellular developmental process;GO:0006139//nucleobase-containing compound metabolic process;GO:0006950//response to stress;GO:0065003//macromolecular complex assembly;GO:0007154//cell [illegible]

id	CON_Mean	KD_Mean	AllMean	log2FoldChange	pvalue	qvalue	State	gene_id	XF_1_count	XF_1_FPKM	XF_1_TPM	XF_1_CPM	XF_2_count	XF_2_FPKM	XF_2_TPM	XF_2_CPM	XF_3_count	XF_3_FPKM	XF_3_TPM	XF_3_CPM	Symbol	Description	KEGG_A_class	KEGG_B_class	Pathway	K_ID	GO Component	GO Function	GO Process
GIGYF1	6.037619462	7.106044298	6.57183188	1.071989807	0.000126055	0.012432036	Up	ENSG00000146830	2192	9.362142209	35.348754533	66.077999713	3846	18.154511845	72.910789863	128.134544603	4281	20.571966865	82.758966842	145.196942134	GIGYF1	GRB10 interacting GYF protein 1 [Source:HGNC Symbol;Acc:HGNC:9126]	-	-	-	-	GO:0005575//cellular_component;GO:0032991//macromolecular complex	GO:0003674//molecular_function	GO:0065007//biological regulation;GO:0050896//response to stimulus;GO:0007165//signal transduction;GO:0023052//signaling;GO:0008150//biological_process;GO:0051716//cellular response to stimulus;GO:0044699//single-organism process;GO:0050794//regulation of cellular process;GO:0044763//single-organism cellular process;GO:0050789//regulation of biological process;GO:0007154//cell communication;GO:0044700//single organism signaling;GO:0009987//cellular process

id	CON_Mean	KD_Mean	AllMean	log2FoldChange	pvalue	qvalue	State	gene_id	XF_1_count	XF_1_FPKM	XF_1_TPM	XF_1_CPM	XF_2_count	XF_2_FPKM	XF_2_TPM	XF_2_CPM	XF_3_count	XF_3_FPKM	XF_3_TPM	XF_3_CPM	Symbol	Description	KEGG_A_class	KEGG_B_class	Pathway	K_ID	GO Component	GO Function	GO Process
TOMM40	6.469926579	7.573253915	7.021590247	1.11655122	0.000127729	0.012524767	Up	ENSG00000130204	2959	24.708939224	93.293843321	89.199270598	6432	59.360258519	238.398221445	214.290533253	4894	45.980004077	184.972961396	165.987814717	TOMM40	translocase of outer mitochondrial membrane 40 [Source:HGNC Symbol;Acc:HGNC:18001]	Human Diseases	Neurodegenerative diseases	ko05014//Amyotrophic lateral sclerosis (ALS)	K11518	GO:0043227//membrane-bounded organelle;GO:0043229//intracellular organelle;GO:0005622//intracellular;GO:0005737//cytoplasm;GO:0005623//cell;GO:0005739//mitochondrion;GO:0005575//cellular_component;GO:0044444//cytoplasmic part;GO:0044424//intracellular part;GO:0044464//cell part;GO:0043231//intracellular membrane-bounded organelle;GO:0043226//organelle;GO:0005829//cytosol;GO:0032991//macromolecular complex	GO:0005215//transporter activity;GO:0003674//molecular_function;GO:0022857//transmembrane transporter activity	GO:0008152//metabolic process;GO:0034613//cellular protein localization;GO:0071840//cellular component organization or biogenesis;GO:0008104//protein localization;GO:0051641//cellular localization;GO:0051234//establishment of localization;GO:0007005//mitochondrion organization;GO:0051649//establishment of localization in cell;GO:0070727//cellular macromolecule localization;GO:0006886//intracellular protein transport;GO:0045184//establishment of protein localization;GO:0008150//biological_process;GO:0006810//transport;GO:0051179//localization;GO:0009056//catabolic process;GO:0006996//organelle organization;GO:0033036//macromolecule localization;GO:0016043//cellular component organization;GO:0006914//autophagy;GO:0071702//organic substance transport;GO:0006605//protein targeting;GO:0009987//cellular process;GO:0055085//transmembrane transport;GO:0015031//protein transport;GO:0046907//intracellular transport
VCX2	0.80178327	-1.70853	-0.45337	-2.77393	0.000129411	0.012617209	Down	ENSG00000177504	55	2.03433007	7.68104486	1.657979007	11	0.449668045	1.805923102	0.366479457	3	0.124846361	0.502244434	0.101749784	VCX2	variable charge X-linked 2 [Source:HGNC Symbol;Acc:HGNC:18158]	-	-	-	-	GO:0005575//cellular_component	GO:0003674//molecular_function	GO:0048856//anatomical structure development;GO:0032502//developmental process;GO:0008150//biological_process

id	CON_Mean	KD_Mean	AllMean	log2FoldChange	pvalue	qvalue	State	gene_id	XF_1_count	XF_1_FPKM	XF_1_TPM	XF_1_CPM	XF_2_count	XF_2_FPKM	XF_2_TPM	XF_2_CPM	XF_3_count	XF_3_FPKM	XF_3_TPM	XF_3_CPM	Symbol	Description	KEGG_A_class	KEGG_B_class	Pathway	K_ID	GO Component	GO Function	GO Process
CEMIP2	7.854679262	8.910583554	8.382631408	1.057103264	0.000131037	0.012665351	Up	ENSG00000135048	7732	25.187130254	95.099354914	233.081703367	14768	53.167854856	213.52875395	492.015328836	13618	49.910977668	200.786875311	461.876187335	CEMIP2	cell migration inducing hyaluronidase 2 [Source:HGNC Symbol;Acc:HGNC:11869]	-	-	-	-	GO:0044464//cell part;GO:0043226//organelle;GO:0044421//extracellular region part;GO:0016020//membrane;GO:0005886//plasma membrane;GO:0005575//cellular_component;GO:0005622//intracellular;GO:0005623//cell;GO:0005576//extracellular region;GO:0005615//extracellular space;GO:0071944//cell periphery	GO:0016787//hydrolase activity;GO:0003824//catalytic activity;GO:0005488//binding;GO:0016798//hydrolase activity, acting on glycosyl bonds;GO:0043167//ion binding;GO:0003674//molecular_function	GO:0008152//metabolic process;GO:0008150//biological_process;GO:0044699//single-organism process;GO:0044281//small molecule metabolic process;GO:0048856//anatomical structure development;GO:0048646//anatomical structure formation involved in morphogenesis;GO:0044710//single-organism metabolic process;GO:0009056//catabolic process;GO:0032502//developmental process;GO:0009653//anatomical structure morphogenesis
HSPA8	10.208042606	11.328123147	10.768082876	1.134556991	0.000132129	0.012665351	Up	ENSG00000109971	39525	245.717468521	927.758442993	1191.484004859	87767	603.025974991	2421.827726333	2924.072952733	65426	457.62572247	1840.982548742	2219.027128256	HSPA8	heat shock protein family A (Hsp70) member 8 [Source:HGNC Symbol;Acc:HGNC:5241]	Environmental Information Processing;Human Diseases;Cellular Processes;Genetic Information Processing; Organismal Systems	Endocrine system; Transcription; Folding, sorting and degradation;Immune system; Transport and catabolism;Infectious diseases;Signal transduction	ko04612//Antigen processing and presentation; ko04144//Endocytosis;ko05169//Epstein-Barr virus infection; ko04010//MAPK signaling pathway; ko05164//Influenza A;ko05145//Toxoplasmosis;ko04[illegible]	K03283	GO:0032991//macromolecular complex;GO:0070013//intracellular organelle lumen;GO:0031974//membrane-enclosed lumen;GO:0043228//non-membrane-bounded organelle;GO:0044444//cytoplasmic part;GO:0005886//plasma membrane;GO:0097708//intracellular vesicle;GO:0044421//extracellular region part;GO:0043232//intracellular non-membrane-bounded organelle;GO:0012505//endomembrane system;GO:0005654//nucleoplasm;GO:0044428//nuclear part;GO:0005576//extracellular region;GO:0043231//intracellular membrane-bounded organelle;GO:0044422//organelle part;GO:0005730//nucleolus;GO:0031981//nuclear lumen;GO:0044446//intracellular organelle part;GO:0005615//extracellular space;GO:0043227//membrane-bounded organelle;GO:0016020//membrane;GO:0031410//cytoplasmic vesicle;GO:0000323//lytic vacuole;GO:0043226//organelle;GO:0005575//cellular_component;GO:0043233//organelle lumen;GO:0005737//cytoplasm;GO:0005829//cytosol;GO:0044424//intracellular part;GO:0005634//nucleus;GO:0005764//lysosome;GO:0005623//cell;GO:0044464//cell part;GO:0071944//cell periphery;GO:0005768//endosome;GO:0005773//vacuole;GO:0005622//intracellular;GO:0043229//intracellular [illegible]	GO:1901363//heterocyclic compound binding;GO:0003674//molecular_function;GO:0060090//binding, bridging;GO:0008289//lipid binding;GO:0005488//binding;GO:0003824//catalytic activity;GO:0016787//hydrolase activity;GO:0051082//unfolded protein binding;GO:0016887//ATPase activity;GO:0030674//protein binding, bridging;GO:0043167//ion binding;GO:0016817//hydrolase activity, acting on acid anhydrides;GO:0016462//pyrophosphatase activity;GO:0019899//enzyme binding;GO:0005515//protein binding;GO:0003723//RNA binding;GO:0016818//hydrolase activity, acting on acid anhydrides, in phosphorus-containing anhydrides;GO:0017111//nucleoside-triphosphatase activity;GO:0003676//nucleic acid binding;GO:0097159//organic cyclic compound binding	GO:0016071//mRNA metabolic process;GO:0030705//cytoskeleton-dependent intracellular transport;GO:0007034//vacuolar transport;GO:0006886//intracellular protein transport;GO:0051716//cellular response to stimulus;GO:0050896//response to stimulus;GO:1901361//organic cyclic compound catabolic process;GO:0043933//macromolecular complex subunit organization;GO:0044238//primary metabolic process;GO:0006397//mRNA processing;GO:0006605//protein targeting;GO:0015031//protein transport;GO:0046700//heterocycle catabolic process;GO:0044419//interspecies interaction between organisms;GO:1901360//organic cyclic compound metabolic process;GO:0007154//cell communication;GO:0006139//nucleobase-containing compound metabolic process;GO:0008152//metabolic process;GO:0007267//cell-cell signaling;GO:0002376//immune system process;GO:0051234//establishment of localization;GO:0070727//c[illegible]

id	CON_Mean	KD_Mean	AllMean	log2FoldChange	pvalue	qvalue	State	gene_id	XF_1_count	XF_1_FPKM	XF_1_TPM	XF_1_CPM	XF_2_count	XF_2_FPKM	XF_2_TPM	XF_2_CPM	XF_3_count	XF_3_FPKM	XF_3_TPM	XF_3_CPM	Symbol	Description	KEGG_A_class	KEGG_B_class	Pathway	K_ID	GO Component	GO Function	GO Process
HACL1	6.218365187	5.168132865	5.693249026	-1.05193	0.000132203	0.012665351	Down	ENSG00000131373	2485	24.480557536	92.431539688	74.910506061	1043	11.355854824	45.606533068	34.748915762	1071	11.870808163	47.755074933	36.32467298	HACL1	2-hydroxyacyl-CoA lyase 1 [Source:HGNC Symbol;Acc:HGNC:17856]	Cellular Processes	Transport and catabolism	ko04146//Peroxisome	K12261	GO:0031974//membrane-enclosed lumen;GO:0043233//organelle lumen;GO:0005654//nucleoplasm;GO:0044446//intracellular organelle part;GO:0031981//nuclear lumen;GO:0005634//nucleus;GO:0005622//intracellular;GO:0044444//cytoplasmic part;GO:0044428//nuclear part;GO:0005575//cellular_component;GO:0043231//intracellular membrane-bounded organelle;GO:0044424//intracellular part;GO:0044464//cell part;GO:0070013//intracellular organelle lumen;GO:0005737//cytoplasm;GO:0044422//organelle part;GO:0043226//organelle;GO:0043229//intracellular organelle;GO:0005777//peroxisome;GO:0005623//cell;GO:0042579//microbody;GO:0005829//cytosol;GO:0043227//membrane-bounded organelle	GO:0005488//binding;GO:0003674//molecular_function;GO:0043167//ion binding;GO:0016829//lyase activity;GO:0003824//catalytic activity	GO:0006886//intracellular protein transport;GO:0008152//metabolic process;GO:0009056//catabolic process;GO:0071704//organic substance metabolic process;GO:0008104//protein localization;GO:0006810//transport;GO:0044699//single-organism process;GO:0044238//primary metabolic process;GO:0006629//lipid metabolic process;GO:0045184//establishment of protein localization;GO:0006605//protein targeting;GO:0051649//establishment of localization in cell;GO:0071702//organic substance transport;GO:0034613//cellular protein localization;GO:0046907//intracellular transport;GO:0033036//macromolecule localization;GO:0051234//establishment of localization;GO:0051641//cellular localization;GO:0044710//single-organism metabolic process;GO:0015031//protein transport;GO:0070727//cellular macromolecule localization;GO:0008150//biological_process;GO:0044281//small molecule metabolic process;GO:0051179//localization
ENSG00000271327	0.920578748	-1.36003	-0.21973	-2.54125	0.000132874	0.012665351	Down	ENSG00000271327	60	1.2098357	4.56799141	1.808704372	12	0.26742192	1.073999874	0.399795771	6	0.136120113	0.54759761	0.203499569	-	-	-	-	-	-	-	-	-

id	CON_Mean	KD_Mean	AllMean	log2FoldChange	pvalue	qvalue	State	gene_id	XF_1_count	XF_1_FPKM	XF_1_TPM	XF_1_CPM	XF_2_count	XF_2_FPKM	XF_2_TPM	XF_2_CPM	XF_3_count	XF_3_FPKM	XF_3_TPM	XF_3_CPM	Symbol	Description	KEGG_A_class	KEGG_B_class	Pathway	K_ID	GO Component	GO Function	GO Process
CLDN4	4.603236791	2.754698654	3.678967723	-1.81828	0.000137682	0.012949796	Down	ENSG00000189143	809	5.189905075	19.5955878893	24.387363945	153	1.084783162	4.356624837	5.09739608	251	1.811675949	7.288191292	8.513065283	CLDN4	claudin 4 [Source:HGNC Symbol;Acc:HGNC:2046]	Cellular Processes;Environmental Information Processing;Human Diseases;Organismal Systems	Infectious diseases;Immune system;Signaling molecules and interaction;Cellular community	ko04514//Cell adhesion molecules (CAMs);ko04530//Tight junction;ko05160//Hepatitis C;ko04670//Leukocyte transendothelial migration	K06087	GO:0005575//cellular_component;GO:0044464//cell part;GO:0032991//macromolecular complex;GO:0071944//cell periphery;GO:0005886//plasma membrane;GO:0016020//membrane;GO:0005623//cell	GO:0022857//transmembrane transporter activity;GO:0003674//molecular_function;GO:0005215//transporter activity;GO:0005198//structural molecule activity	GO:0006950//response to stress;GO:0000902//cell morphogenesis;GO:0065007//biological regulation;GO:0051234//establishment of localization;GO:0048869//cellular developmental process;GO:0040011//locomotion;GO:0008150//biological_process;GO:0009653//anatomical structure morphogenesis;GO:0071840//cellular component organization or biogenesis;GO:0044763//single-organism cellular process;GO:0055085//transmembrane transport;GO:0048856//anatomical structure development;GO:0007155//cell adhesion;GO:0050896//response to stimulus;GO:0006928//movement of cell or subcellular component;GO:0044767//single-organism developmental process;GO:0006810//transport;GO:0065008//regulation of biological quality;GO:0016043//cellular component organization;GO:0051179//localization;GO:0044699//single-organism process;GO:0032989//cellular component morphogenesis;GO:0048870//cell motility;GO:0009987//cellular process;GO:0022610//biological adhesion;GO:0051674//localization of [illegible]

id	CON_Mean	KD_Mean	AllMean	log2FoldChange	pvalue	qvalue	State	gene_id	XF_1_count	XF_1_FPKM	XF_1_TPM	XF_1_CPM	XF_2_count	XF_2_FPKM	XF_2_TPM	XF_2_CPM	XF_3_count	XF_3_FPKM	XF_3_TPM	XF_3_CPM	Symbol	Description	KEGG_A_class	KEGG_B_class	Pathway	K_ID	GO Component	GO Function	GO Process
AKR1C3	4.500176993	2.3949876	3.447582297	-2.02324	0.000137741	0.012949796	Down	ENSG00000196139	753	5.803947805	21.914036522	22.699239865	104	0.885936252	3.558030778	3.464896682	222	1.925206861	7.744914808	7.529484035	AKR1C3	aldo-keto reductase family 1 member C3 [Source:HGNC Symbol;Acc:HGNC:386]	Metabolism; Organismal Systems	Endocrine system; Lipid metabolism	ko00140//Steroid hormone biosynthesis; ko00590//Arachidonic acid metabolism; ko04913//Ovarian Steroidogenesis	K04119	GO:0044421//extracellular region part;GO:0005737//cytoplasm;GO:0043226//organelle;GO:0043227//membrane-bounded organelle;GO:0005829//cytosol;GO:0005623//cell;GO:0005575//cellular_component;GO:0044424//intracellular part;GO:0005634//nucleus;GO:0005615//extracellular space;GO:0005622//intracellular;GO:0043231//intracellular membrane-bounded organelle;GO:0044444//cytoplasmic part;GO:0043229//intracellular organelle;GO:0005576//extracellular region;GO:0044464//cell part	GO:0003674//molecular_function;GO:0003824//catalytic activity;GO:0016491//oxidoreductase activity;GO:0043167//ion binding;GO:0005488//binding	GO:0065007//biological regulation;GO:0019748//secondary metabolic process;GO:0044767//single-organism developmental process;GO:0050896//response to stimulus;GO:0050789//regulation of biological process;GO:0044699//single-organism process;GO:0009058//biosynthetic process;GO:0044237//cellular metabolic process;GO:0032502//developmental process;GO:0044238//primary metabolic process;GO:0044763//single-organism cellular process;GO:0008152//metabolic process;GO:0048856//anatomical structure development;GO:0000003//reproduction;GO:0008283//cell proliferation;GO:0007154//cell communication;GO:0048869//cellular developmental process;GO:0071704//organic substance metabolic process;GO:0008150//biological_process;GO:0006950//response to stress;GO:0008219//cell death;GO:0044700//single organism signaling;GO:0023052//signaling;GO:0030154//cell differentiation;GO:0009987//cellular process;GO:0051186//cofactor metabolic process;GO:0050794//regulation of cellular [illegible]

id	CON_Mean	KD_Mean	AllMean	log2FoldChange	pvalue	qvalue	State	gene_id	XF_1_count	XF_1_FPKM	XF_1_TPM	XF_1_CPM	XF_2_count	XF_2_FPKM	XF_2_TPM	XF_2_CPM	XF_3_count	XF_3_FPKM	XF_3_TPM	XF_3_CPM	Symbol	Description	KEGG_A_class	KEGG_B_class	Pathway	K_ID	GO Component	GO Function	GO Process
ATF5	2.109934488	4.151136321	3.130535405	2.10091136	0.000138135	0.012949796	Up	ENSG00000169136	141	1.469728656	5.549272414	4.250455273	655	7.545707411	30.304504584	21.822185833	414	4.855280162	19.532306879	14.041470227	ATF5	activating transcription factor 5 [Source:HGNC Symbol;Acc:HGNC:790]	-	-	-	-	GO:0005815//microtubule organizing center;GO:0070013//intracellular organelle lumen;GO:0044422//organelle part;GO:0043226//organelle;GO:0005623//cell;GO:0044424//intracellular part;GO:0000228//nuclear chromosome;GO:0005654//nucleoplasm;GO:0044464//cell part;GO:0043227//membrane-bounded organelle;GO:0043231//intracellular membrane-bounded organelle;GO:0005694//chromosome;GO:0031974//membrane-enclosed lumen;GO:0005829//cytosol;GO:0044428//nuclear part;GO:0043228//non-membrane-bounded organelle;GO:0005737//cytoplasm;GO:0005856//cytoskeleton;GO:0032991//macromolecular complex;GO:0044446//intracellular organelle part;GO:0043232//intracellular non-membrane-bounded organelle;GO:0031981//nuclear lumen;GO:0044430//cytoskeletal part;GO:0005575//cellular_component;GO:0005634//nucleus;GO:0043233//organelle lumen;GO:0044444//cytoplasmic part;GO:0005622//intracellular;GO:0043229//intracellular organelle;GO:0015630//microtubule cytoskeleton	GO:0097159//organic cyclic compound binding;GO:0019899//enzyme binding;GO:0005488//binding;GO:0003700//transcription factor activity, sequence-specific DNA binding;GO:0001071//nucleic acid binding transcription factor activity;GO:0003676//nucleic acid binding;GO:0003677//DNA binding;GO:1901363//heterocyclic compound binding;GO:0008092//cytoskeletal protein binding;GO:0005515//protein binding;GO:0003674//molecular_function	GO:0044237//cellular metabolic process;GO:0016043//cellular component organization;GO:0006996//organelle organization;GO:0044763//single-organism cellular process;GO:0048856//anatomical structure development;GO:0008283//cell proliferation;GO:0007049//cell cycle;GO:0007010//cytoskeleton organization;GO:0008150//biological_process;GO:0032502//developmental process;GO:0009058//biosynthetic process;GO:0034641//cellular nitrogen compound metabolic process;GO:0044699//single-organism process;GO:0030154//cell differentiation;GO:0048869//cellular developmental process;GO:0008152//metabolic process;GO:0071840//cellular component organization or biogenesis;GO:0040007//growth;GO:0044767//single-organism developmental process;GO:0008219//cell death;GO:0006807//nitrogen compound metabolic process;GO:0009987//cellular process

id	CON_Mean	KD_Mean	AllMean	log2FoldChange	pvalue	qvalue	State	gene_id	XF_1_count	XF_1_FPKM	XF_1_TPM	XF_1_CPM	XF_2_count	XF_2_FPKM	XF_2_TPM	XF_2_CPM	XF_3_count	XF_3_FPKM	XF_3_TPM	XF_3_CPM	Symbol	Description	KEGG_A_class	KEGG_B_class	Pathway	K_ID	GO Component	GO Function	GO Process
H2AX	6.779503963	7.833695836	7.306599899	1.059720522	0.000140541	0.013103378	Up	ENSG00000188486	3668	68.508133368	258.666994776	110.572127257	7337	151.450927911	608.245866024	244.441797648	6157	129.38319324	520.495656515	208.824473889	H2AX	H2A.X variant histone [Source:HGNC Symbol;Acc:HGNC:4739]	Human Diseases	Substance dependence;Immune diseases	ko05322//Systemic lupus erythematosus;ko05034//Alcoholism	K11251	GO:0043229//intracellular organelle;GO:0000228//nuclear chromosome;GO:0005576//extracellular region;GO:0044446//intracellular organelle part;GO:0032991//macromolecular complex;GO:0005622//intracellular;GO:0044464//cell part;GO:0005634//nucleus;GO:0043231//intracellular membrane-bounded organelle;GO:0044424//intracellular part;GO:0031981//nuclear lumen;GO:0070013//intracellular organelle lumen;GO:0044430//cytoskeletal part;GO:0005623//cell;GO:0005654//nucleoplasm;GO:0005856//cytoskeleton;GO:0005575//cellular_component;GO:0043232//intracellular non-membrane-bounded organelle;GO:0043228//non-membrane-bounded organelle;GO:0044422//organelle part;GO:0005615//extracellular space;GO:0044421//extracellular region part;GO:0044428//nuclear part;GO:0043227//membrane-bounded organelle;GO:0031974//membrane-enclosed lumen;GO:0005694//chromosome;GO:0005815//microtubule organizing center;GO:0015630//microtubule cytoskeleton;GO:0043233//organelle lumen;GO:0043226//organelle	GO:0003677//DNA binding;GO:0005488//binding;GO:0042393//histone binding;GO:0005515//protein binding;GO:0003676//nucleic acid binding;GO:1901363//heterocyclic compound binding;GO:0097159//organic cyclic compound binding;GO:0003674//molecular_function;GO:0019899//enzyme binding	GO:0065003//macromolecular complex assembly;GO:0046483//heterocycle metabolic process;GO:0006139//nucleobase-containing compound metabolic process;GO:0000003//reproduction;GO:0006807//nitrogen compound metabolic process;GO:0043170//macromolecule metabolic process;GO:0009058//biosynthetic process;GO:0006996//organelle organization;GO:0071704//organic substance metabolic process;GO:0016043//cellular component organization;GO:0043933//macromolecular complex subunit organization;GO:0048856//anatomical structure development;GO:0034641//cellular nitrogen compound metabolic process;GO:0044763//single-organism cellular process;GO:0044403//symbiosis, encompassing mutualism through parasitism;GO:0044237//cellular metabolic process;GO:0007049//cell cycle;GO:0009987//cellular process;GO:0044260//cellular macromolecule metabolic process;GO:0006259//DNA metabolic process;GO:0032502//developmental process;GO:0006950//response to stress;GO:0050896//respon
TONSL-AS1	-1.70414	0.9817411 66	-0.3612	3.180412507	0.00014314	0.013273131	Up	ENSG00000232600	7	0.493026893	1.861527652	0.21101551	71	5.526771756	22.1962065 18	2.365458312	43	3.407508351	13.708065568	1.458413574	-	-	-	-	-	-	-	-	-
NEAT1	8.123445002	9.172014178	8.64772959	1.049205343	0.000144317	0.013309966	Up	ENSG00000245532	9316	12.335024324	46.573501794	280.83149878	16698	24.435183175	98.134751345	556.315815338	17306	25.781200368	103.715192641	586.960588781	-	-	-	-	-	-	-	-	-

id	CON_Mean	KD_Mean	AllMean	log2FoldChange	pvalue	qvalue	State	gene_id	XF_1_count	XF_1_FPKM	XF_1_TPM	XF_1_CPM	XF_2_count	XF_2_FPKM	XF_2_TPM	XF_2_CPM	XF_3_count	XF_3_FPKM	XF_3_TPM	XF_3_CPM	Symbol	Description	KEGG_A_class	KEGG_B_class	Pathway	K_ID	GO Component	GO Function	GO Process
SAT1	6.011758745	4.911327568	5.461543156	-1.10101	0.000145324	0.013327796	Down	ENSG00000130066	2153	26.149211068	98.731895181	64.902341871	845	11.342580798	45.553222919	28.152285541	925	12.640149132	50.850056767	31.372850146	SAT1	spermidine/spermine N1-acetyltransferase 1 [Source:HGNC Symbol;Acc:HGNC:10540]	Metabolism	Amino acid metabolism	ko00330//Arginine and proline metabolism	K00657	GO:0005622//intracellular;GO:0044464//cell part;GO:0005737//cytoplasm;GO:0005575//cellular_component;GO:0044424//intracellular part;GO:0005829//cytosol;GO:0005623//cell;GO:0044444//cytoplasmic part	GO:0003674//molecular_function;GO:0016746//transferase activity, transferring acyl groups;GO:0005488//binding;GO:0043167//ion binding;GO:0003824//catalytic activity;GO:0016740//transferase activity	GO:0009987//cellular process;GO:0006807//nitrogen compound metabolic process;GO:0032502//developmental process;GO:0009058//biosynthetic process;GO:0009653//anatomical structure morphogenesis;GO:0009056//catabolic process;GO:0034641//cellular nitrogen compound metabolic process;GO:0008150//biological_process;GO:0048856//anatomical structure development;GO:0008152//metabolic process;GO:0044699//single-organism process;GO:0048646//anatomical structure formation involved in morphogenesis;GO:0044237//cellular metabolic process;GO:0008283//cell proliferation

id	CON_Mean	KD_Mean	AllMean	log2FoldChange	pvalue	qvalue	State	gene_id	XF_1_count	XF_1_FPKM	XF_1_TPM	XF_1_CPM	XF_2_count	XF_2_FPKM	XF_2_TPM	XF_2_CPM	XF_3_count	XF_3_FPKM	XF_3_TPM	XF_3_CPM	Symbol	Description	KEGG_A_class	KEGG_B_class	Pathway	K_ID	GO Component	GO Function	GO Process
AVPR2	0.672320943	2.99749789	1.834909	2.400740194	0.000146073	0.013327796	Up	ENSG00000126895	50	0.669295579	2.52706748	1.507253643	255	3.772495619	15.1508141710	8.495660134	212	3.192858831	12.844551999	7.190318087	AVPR2	arginine vasopressin receptor 2 [Source:HGNC Symbol;Acc:HGNC:897]	Environmental Information Processing;Organismal Systems	Signaling molecules and interaction;Excretory system	ko04080//Neuroactive ligand-receptor interaction;ko04962//Vasopressin-regulated water reabsorption	K04228	GO:0044464//cell part;GO:0005768//endosome;GO:0097708//intracellular vesicle;GO:0005886//plasma membrane;GO:0031410//cytoplasmic vesicle;GO:0043226//organelle;GO:0005773//vacuole;GO:0005783//endoplasmic reticulum;GO:0044424//intracellular part;GO:0005575//cellular_component;GO:0043231//intracellular membrane-bounded organelle;GO:0005737//cytoplasm;GO:0043229//intracellular organelle;GO:0043227//membrane-bounded organelle;GO:0016020//membrane;GO:0005794//Golgi apparatus;GO:0044444//cytoplasmic part;GO:0071944//cell periphery;GO:0005623//cell;GO:0031982//vesicle;GO:0005622//intracellular;GO:0012505//endomembrane system	GO:0003674//molecular_function	GO:0007165//signal transduction;GO:0050789//regulation of biological process;GO:0032501//multicellular organismal process;GO:0009987//cellular process;GO:0003008//system process;GO:0044763//single-organism cellular process;GO:0061024//membrane organization;GO:0050896//response to stimulus;GO:0036211//protein modification process;GO:0044699//single-organism process;GO:0044700//single organism signaling;GO:0019538//protein metabolic process;GO:0008152//metabolic process;GO:0071840//cellular component organization or biogenesis;GO:0043170//macromolecule metabolic process;GO:0050794//regulation of cellular process;GO:0044260//cellular macromolecule metabolic process;GO:0048856//anatomical structure development;GO:0065008//regulation of biological quality;GO:0007154//cell communication;GO:0006810//transport;GO:0071704//organic substance metabolic process;GO:0008150//biological_process;GO:0043412//macromolecule modification;GO:0003013//circulatory system [illegible]

id	CON_Mean	KD_Mean	AllMean	log2FoldChange	pvalue	qvalue	State	gene_id	XF_1_count	XF_1_FPKM	XF_1_TPM	XF_1_CPM	XF_2_count	XF_2_FPKM	XF_2_TPM	XF_2_CPM	XF_3_count	XF_3_FPKM	XF_3_TPM	XF_3_CPM	Symbol	Description	KEGG_A_class	KEGG_B_class	Pathway	K_ID	GO Component	GO Function	GO Process
TICRR	5.159342456	6.262645874	5.710994165	1.111794591	0.000150048	0.013617656	Up	ENSG00000140534	1191	4.266521899	16.109158773	35.9027817 78	2498	9.889976589	39.7193827 61	83.2241529 95	2045	8.242357251	33.1581795 23	69.3594362 68	TICRR	TOPBP1 interacting checkpoint and replication regulator [Source:HGNC Symbol;Acc:HGNC:28704]	-	-	-	-	GO:0031974//membrane-enclosed lumen;GO:0031981//nuclear lumen;GO:0043233//organelle lumen;GO:0005575//cellular_component;GO:0044446//intracellular organelle part;GO:0005634//nucleus;GO:0070013//intracellular organelle lumen;GO:0005623//cell;GO:0043226//organelle;GO:0044422//organelle part;GO:0043231//intracellular membrane-bounded organelle;GO:0005622//intracellular;GO:0043227//membrane-bounded organelle;GO:0044464//cell part;GO:0044428//nuclear part;GO:0044424//intracellular part;GO:0005654//nucleoplasm;GO:0043229//intracellular organelle	GO:0003674//molecular_function	GO:0043170//macromolecule metabolic process;GO:0009058//biosynthetic process;GO:0006807//nitrogen compound metabolic process;GO:1901360//organic cyclic compound metabolic process;GO:0044763//single-organism cellular process;GO:0044699//single-organism process;GO:0071704//organic substance metabolic process;GO:0044238//primary metabolic process;GO:0008152//metabolic process;GO:0034641//cellular nitrogen compound metabolic process;GO:0090304//nucleic acid metabolic process;GO:0008150//biological_process;GO:0006950//response to stress;GO:0046483//heterocycle metabolic process;GO:0006139//nucleobase-containing compound metabolic process;GO:0006259//DNA metabolic process;GO:0044260//cellular macromolecule metabolic process;GO:0044237//cellular metabolic process;GO:0000278//mitotic cell cycle;GO:0009987//cellular process;GO:0007049//cell cycle;GO:0006725//cellular aromatic compound metabolic process;GO:0050896//response to stimulus

id	CON_Mean	KD_Mean	AllMean	log2FoldChange	pvalue	qvalue	State	gene_id	XF_1_count	XF_1_FPKM	XF_1_TPM	XF_1_CPM	XF_2_count	XF_2_FPKM	XF_2_TPM	XF_2_CPM	XF_3_count	XF_3_FPKM	XF_3_TPM	XF_3_CPM	Symbol	Description	KEGG_A_class	KEGG_B_class	Pathway	K_ID	GO Component	GO Function	GO Process
SLIT2	3.966059561	5.281328531	4.623694046	1.320675341	0.000163075	0.014644119	Up	ENSG00000145147	519	1.413688697	5.337681658	15.645292815	1129	3.398763783	13.649860382	37.614118788	1158	3.548876545	14.276775684	39.275416723	SLIT2	slit guidance ligand 2 [Source:HGNC Symbol;Acc:HGNC:11086]	Organismal Systems	Development	ko04360//Axon guidance	K06839	GO:0044421//extracellular region part;GO:0005576//extracellular region;GO:0016020//membrane;GO:0005623//cell;GO:0005622//intracellular;GO:0043226//organelle;GO:0005575//cellular_component;GO:0005737//cytoplasm;GO:0071944//cell periphery;GO:0044464//cell part;GO:0005615//extracellular space;GO:0005886//plasma membrane;GO:0044424//intracellular part	GO:0043167//ion binding;GO:0003674//molecular_function;GO:0005488//binding;GO:0098772//molecular function regulator;GO:0030234//enzyme regulator activity	GO:0006464//cellular protein modification process;GO:0044763//single-organism cellular process;GO:0036211//protein modification process;GO:0050794//regulation of cellular process;GO:0043412//macromolecule modification;GO:0044260//cellular macromolecule metabolic process;GO:0044767//single-organism developmental process;GO:0065007//biological regulation;GO:0000003//reproduction;GO:0008150//biological_process;GO:0051179//localization;GO:0071704//organic substance metabolic process;GO:0016043//cellular component organization;GO:0032501//multicellular organismal process;GO:0071840//cellular component organization or biogenesis;GO:0065003//macromolecular complex assembly;GO:0044238//primary metabolic process;GO:0044267//cellular protein metabolic process;GO:0007154//cell communication;GO:0044085//cellular component biogenesis;GO:0048856//anatomical structure development;GO:0007165//signal transduction;GO:0048870//cell motility;GO:0040011//locomotion;GO:0023052//signaling;GO:0044237//cellular [illegible]

id	CON_Mean	KD_Mean	AllMean	log2FoldChange	pvalue	qvalue	State	gene_id	XF_1_count	XF_1_FPKM	XF_1_TPM	XF_1_CPM	XF_2_count	XF_2_FPKM	XF_2_TPM	XF_2_CPM	XF_3_count	XF_3_FPKM	XF_3_TPM	XF_3_CPM	Symbol	Description	KEGG_A_class	KEGG_B_class	Pathway	K_ID	GO Component	GO Function	GO Process
BRD4	6.548588832	7.589203634	7.068896233	1.046062409	0.00016557	0.014745465	Up	ENSG00000141867	3125	6.785029724	25.618319489	94.203352693	6183	14.836846082	59.586629272	205.994771004	5205	12.71505875	51.151410674	176.535875685	BRD4	bromodomain containing 4 [Source:HGNC Symbol;Acc:HGNC:13575]	-	-	-	-	GO:0044446//intracellular organelle part;GO:0044464//cell part;GO:0000228//nuclear chromosome;GO:0031981//nuclear lumen;GO:0005575//cellular_component;GO:0044428//nuclear part;GO:0031974//membrane-enclosed lumen;GO:0043233//organelle lumen;GO:0043232//intracellular non-membrane-bounded organelle;GO:0005694//chromosome;GO:0005634//nucleus;GO:0044422//organelle part;GO:0044424//intracellular part;GO:0005654//nucleoplasm;GO:0070013//intracellular organelle lumen;GO:0043227//membrane-bounded organelle;GO:0043228//non-membrane-bounded organelle;GO:0043229//intracellular organelle;GO:0043226//organelle;GO:0005623//cell;GO:0005622//intracellular;GO:0043231//intracellular membrane-bounded organelle	GO:0005515//protein binding;GO:0016301//kinase activity;GO:0042393//histone binding;GO:0019899//enzyme binding;GO:0003824//catalytic activity;GO:0016740//transferase activity;GO:0003674//molecular_function;GO:0005488//binding;GO:0016772//transferase activity, transferring phosphorus-containing groups	GO:0044700//single organism signaling;GO:0043412//macromolecule modification;GO:0019538//protein metabolic process;GO:0044763//single-organism cellular process;GO:0000278//mitotic cell cycle;GO:0009058//biosynthetic process;GO:0023052//signaling;GO:0007154//cell communication;GO:0071704//organic substance metabolic process;GO:0006950//response to stress;GO:0006464//cellular protein modification process;GO:0006996//organelle organization;GO:0007049//cell cycle;GO:0051276//chromosome organization;GO:0044238//primary metabolic process;GO:0044267//cellular protein metabolic process;GO:0006807//nitrogen compound metabolic process;GO:0044419//interspecies interaction between organisms;GO:0034641//cellular nitrogen compound metabolic process;GO:0008152//metabolic process;GO:0044260//cellular macromolecule metabolic process;GO:0050896//response to stimulus;GO:0044699//single-organism [illegible]

id	CON_Mean	KD_Mean	AllMean	log2FoldChange	pvalue	qvalue	State	gene_id	XF_1_count	XF_1_FPKM	XF_1_TPM	XF_1_CPM	XF_2_count	XF_2_FPKM	XF_2_TPM	XF_2_CPM	XF_3_count	XF_3_FPKM	XF_3_TPM	XF_3_CPM	Symbol	Description	KEGG_A_class	KEGG_B_class	Pathway	K_ID	GO Component	GO Function	GO Process
ITPRID2	10.307086069	9.159402554	9.733244311	-1.11526	0.000166013	0.014745465	Down	ENSG00000138434	42334	131.766805837	497.513536001	1276.161514527	13687	47.083158816	189.091853739	456.000393133	20747	72.655404437	292.285430974	703.667591323	ITPRID2	ITPR interacting domain containing 2 [Source:HGNC Symbol;Acc:HGNC:11319]	-	-	-	-	GO:0044428//nuclear part;GO:0005575//cellular_component;GO:0005886//plasma membrane;GO:0071944//cell periphery;GO:0031981//nuclear lumen;GO:0005622//intracellular;GO:0043233//organelle lumen;GO:0044444//cytoplasmic part;GO:0005737//cytoplasm;GO:0005634//nucleus;GO:0044424//intracellular part;GO:0031974//membrane-enclosed lumen;GO:0005829//cytosol;GO:0043226//organelle;GO:0005623//cell;GO:0043227//membrane-bounded organelle;GO:0070013//intracellular organelle lumen;GO:0044464//cell part;GO:0044446//intracellular organelle part;GO:0005654//nucleoplasm;GO:0044422//organelle part;GO:0016020//membrane;GO:0043231//intracellular membrane-bounded organelle;GO:0043229//intracellular organelle	GO:0005515//protein binding;GO:0005488//binding;GO:0003674//molecular_function;GO:0008092//cytoskeletal protein binding	-

id	CON_Mean	KD_Mean	AllMean	log2FoldChange	pvalue	qvalue	State	gene_id	XF_1_count	XF_1_FPKM	XF_1_TPM	XF_1_CPM	XF_2_count	XF_2_FPKM	XF_2_TPM	XF_2_CPM	XF_3_count	XF_3_FPKM	XF_3_TPM	XF_3_CPM	Symbol	Description	KEGG_A_class	KEGG_B_class	Pathway	K_ID	GO Component	GO Function	GO Process
EIF4A1	2.815534273	4.507256836	3.661395555	1.705110075	0.000167579	0.014745465	Up	ENSG00000161960	232	1.345192711	5.079060526	6.993656904	677	4.338362136	17.423404869	22.555144747	658	4.292579217	17.26861721	22.317119347	EIF4A1	eukaryotic translation initiation factor 4A1 [Source:HGNC Symbol;Acc:HGNC:3282]	Genetic Information Processing	Translation	ko03013//RNA transport	K03257	GO:0044464//cell part;GO:0005576//extracellular region;GO:0005623//cell;GO:0005622//intracellular;GO:0005737//cytoplasm;GO:0044424//intracellular part;GO:0005575//cellular_component;GO:0032991//macromolecular complex;GO:0005829//cytosol;GO:0043226//organelle;GO:0044444//cytoplasmic part;GO:0005615//extracellular space;GO:0044421//extracellular region part	GO:0097159//organic cyclic compound binding;GO:0016462//pyrophosphatase activity;GO:0017111//nucleoside-triphosphatase activity;GO:0016887//ATPase activity;GO:0005488//binding;GO:0043167//ion binding;GO:0003676//nucleic acid binding;GO:0016818//hydrolase activity, acting on acid anhydrides, in phosphorus-containing anhydrides;GO:0003723//RNA binding;GO:0004386//helicase activity;GO:0016817//hydrolase activity, acting on acid anhydrides;GO:0003824//catalytic activity;GO:1901363//heterocyclic compound binding;GO:0044822//poly(A) RNA binding;GO:0003674//molecular_function;GO:0003729//mRNA binding;GO:0008135//translation factor activity, RNA binding;GO:0016787//hydrolase activity	GO:0043604//amide biosynthetic process;GO:1901576//organic substance biosynthetic process;GO:0044267//cellular protein metabolic process;GO:0006412//translation;GO:0006518//peptide metabolic process;GO:0043043//peptide biosynthetic process;GO:0006807//nitrogen compound metabolic process;GO:0019538//protein metabolic process;GO:0044260//cellular macromolecule metabolic process;GO:0044271//cellular nitrogen compound biosynthetic process;GO:0071704//organic substance metabolic process;GO:0008150//biological_process;GO:0010467//gene expression;GO:0044238//primary metabolic process;GO:0044237//cellular metabolic process;GO:1901564//organonitrogen compound metabolic process;GO:0044419//interspecies interaction between organisms;GO:0044249//cellular biosynthetic process;GO:0051704//multi-organism process;GO:0009059//macromolecule biosynthetic process;GO:0009987//cellular process;GO:0009058//biosynthetic process;GO:0043603//cellular amide metabolic [illegible]
CNTN3	4.990361839	3.467645152	4.229003495	-1.51502	0.00016766	0.014745465	Down	ENSG00000113805	1059	6.248508937	23.59257141	31.9236321 61	280	1.825908786	7.333078023	9.32856799	373	2.476196877	9.961492576	12.650889843	CNTN3	contactin 3 [Source:HGNC Symbol;Acc:HGNC:2173]	-	-	-	-	GO:0071944//cell periphery;GO:0005623//cell;GO:0005575//cellular_component;GO:0005576//extracellular region;GO:0016020//membrane;GO:0044464//cell part;GO:0005886//plasma membrane	GO:0003674//molecular_function	GO:0032502//developmental process;GO:0007155//cell adhesion;GO:0022610//biological adhesion;GO:0008150//biological_process;GO:0048856//anatomical structure development

id	CON_Mean	KD_Mean	AllMean	log2FoldChange	pvalue	qvalue	State	gene_id	XF_1_count	XF_1_FPKM	XF_1_TPM	XF_1_CPM	XF_2_count	XF_2_FPKM	XF_2_TPM	XF_2_CPM	XF_3_count	XF_3_FPKM	XF_3_TPM	XF_3_CPM	Symbol	Description	KEGG_A_class	KEGG_B_class	Pathway	K_ID	GO Component	GO Function	GO Process
SETD1A	5.610981236	6.6631440644	6.13706265	1.05481407	0.000169487	0.01482964	Up	ENSG00000099381	1630	7.529339376	28.428618518	49.136468765	3126	15.958749363	64.092333147	104.146798344	2849	14.806677666	59.565784548	96.628378449	SETD1A	SET domain containing 1A, histone lysine methyltransferase [Source:HGNC Symbol;Acc:HGNC:29010]	Metabolism	Amino acid metabolism	ko00310//Lysine degradation	K11422	GO:0043226//organelle;GO:0005623//cell;GO:0031974//membrane-enclosed lumen;GO:0005575//cellular_component;GO:0044424//intracellular part;GO:0005634//nucleus;GO:0005694//chromosome;GO:0032991//macromolecular complex;GO:0043233//organelle lumen;GO:0005654//nucleoplasm;GO:0043228//non-membrane-bounded organelle;GO:0005622//intracellular;GO:0043231//intracellular membrane-bounded organelle;GO:0044464//cell part;GO:0043232//intracellular non-membrane-bounded organelle;GO:0031981//nuclear lumen;GO:0043229//intracellular organelle;GO:0000228//nuclear chromosome;GO:0044446//intracellular organelle part;GO:0044422//organelle part;GO:0070013//intracellular organelle lumen;GO:0044428//nuclear part;GO:0043227//membrane-bounded organelle	GO:0003723//RNA binding;GO:0003824//catalytic activity;GO:1901363//heterocyclic compound binding;GO:0016741//transferase activity, transferring one-carbon groups;GO:0008134//transcription factor binding;GO:0097159//organic cyclic compound binding;GO:0003674//molecular_function;GO:0016740//transferase activity;GO:0005488//binding;GO:0003676//nucleic acid binding;GO:0008168//methyltransferase activity;GO:0005515//protein binding	GO:0044763//single-organism cellular process;GO:0044237//cellular metabolic process;GO:0044238//primary metabolic process;GO:0030154//cell differentiation;GO:0019538//protein metabolic process;GO:0032502//developmental process;GO:0048869//cellular developmental process;GO:0002376//immune system process;GO:0006464//cellular protein modification process;GO:0043412//macromolecule modification;GO:0044260//cellular macromolecule metabolic process;GO:0071704//organic substance metabolic process;GO:0071840//cellular component organization or biogenesis;GO:0009987//cellular process;GO:0044699//single-organism process;GO:0043170//macromolecule metabolic process;GO:0051276//chromosome organization;GO:0048856//anatomical structure development;GO:0044767//single-organism developmental process;GO:0044267//cellular protein metabolic process;GO:0008152//metabolic process;GO:0008150//biological_process;GO:0036211//protein modification process;GO:0006996//orga[illegible]

id	CON_Mean	KD_Mean	AllMean	log2FoldChange	pvalue	qvalue	State	gene_id	XF_1_count	XF_1_FPKM	XF_1_TPM	XF_1_CPM	XF_2_count	XF_2_FPKM	XF_2_TPM	XF_2_CPM	XF_3_count	XF_3_FPKM	XF_3_TPM	XF_3_CPM	Symbol	Description	KEGG_A_class	KEGG_B_class	Pathway	K_ID	GO Component	GO Function	GO Process
KMT2B	5.855009031	6.889144311	6.372076671	1.035788482	0.000171541	0.014932781	Up	ENSG00000272333	1931	5.564490555	21.00991486	58.2101135696	3599	11.4621369 83	46.0333755 11	119.905414984	3386	10.9780699 58	44.1636783 49	114.841589831	KMT2B	lysine methyltransferase 2B [Source:HGNC Symbol;Acc:HGNC:15840]	Metabolism	Amino acid metabolism	ko00310//Lysine degradation	K14959	GO:0044446//intracellular organelle part;GO:0031974//membrane-enclosed lumen;GO:0005575//cellular_component;GO:0043231//intracellular membrane-bounded organelle;GO:0005623//cell;GO:0043233//organelle lumen;GO:0070013//intracellular organelle lumen;GO:0044464//cell part;GO:0005654//nucleoplasm;GO:0044424//intracellular part;GO:0044428//nuclear part;GO:0005622//intracellular;GO:0044422//organelle part;GO:0043226//organelle;GO:0031981//nuclear lumen;GO:0032991//macromolecular complex;GO:0043227//membrane-bounded organelle;GO:0043229//intracellular organelle;GO:0005634//nucleus	GO:0016741//transferase activity, transferring one-carbon groups;GO:0016740//transferase activity;GO:0008168//methyltransferase activity;GO:0003674//molecular_function;GO:0005488//binding;GO:1901363//heterocyclic compound binding;GO:0003824//catalytic activity;GO:0003677//DNA binding;GO:0003676//nucleic acid binding;GO:0043167//ion binding;GO:0097159//organic cyclic compound binding;GO:0003700//transcription factor activity, sequence-specific DNA binding;GO:0001071//nucleic acid binding transcription factor activity	GO:0016043//cellular component organization;GO:0009058//biosynthetic process;GO:0048856//anatomical structure development;GO:0071840//cellular component organization or biogenesis;GO:0036211//protein modification process;GO:0044238//primary metabolic process;GO:0051276//chromosome organization;GO:0002376//immune system process;GO:0032502//developmental process;GO:0009987//cellular process;GO:0044699//single-organism process;GO:0044267//cellular protein metabolic process;GO:0008152//metabolic process;GO:0043412//macromolecule modification;GO:0050877//neurological system process;GO:0006996//organelle organization;GO:0044237//cellular metabolic process;GO:0000003//reproduction;GO:0034641//cellular nitrogen compound metabolic process;GO:0008150//biological_process;GO:0019538//protein metabolic process;GO:0030154//cell differentiation;GO:0006464//cellular protein modification process;GO:0048869//cellular developmental [illegible]

id	CON_Mean	KD_Mean	AllMean	log2FoldChange	pvalue	qvalue	State	gene_id	XF_1_count	XF_1_FPKM	XF_1_TPM	XF_1_CPM	XF_2_count	XF_2_FPKM	XF_2_TPM	XF_2_CPM	XF_3_count	XF_3_FPKM	XF_3_TPM	XF_3_CPM	Symbol	Description	KEGG_A_class	KEGG_B_class	Pathway	K_ID	GO Component	GO Function	GO Process
PLXNA3	4.117991906	5.455131299	4.786561602	1.360621565	0.000176564	0.015292053	Up	ENSG00000130827	577	1.485372079	5.608337475	17.3937070 41	1103	3.138163503	12.6032570 67	36.7478946 17	1509	4.370635481	17.5826297 61	51.1801414 81	PLXNA3	plexin A3 [Source:HGNC Symbol;Acc:HGNC:9101]	Organismal Systems	Development	ko04360//Axon guidance	K06820	GO:0016020//membrane;GO:0005575//cellular_component;GO:0071944//cell periphery;GO:0005886//plasma membrane;GO:0044464//cell part;GO:0032991//macromolecular complex;GO:0005623//cell	GO:0003674//molecular_function	GO:0000902//cell morphogenesis;GO:0044767//single-organism developmental process;GO:0050896//response to stimulus;GO:0040011//locomotion;GO:0071840//cellular component organization or biogenesis;GO:0040007//growth;GO:0007010//cytoskeleton organization;GO:0044700//single organism signaling;GO:0009987//cellular process;GO:0044763//single-organism cellular process;GO:0065007//biological regulation;GO:0044699//single-organism process;GO:0051674//localization of cell;GO:0050794//regulation of cellular process;GO:0051716//cellular response to stimulus;GO:0048856//anatomical structure development;GO:0051179//localization;GO:0023052//signaling;GO:0030154//cell differentiation;GO:0006996//organelle organization;GO:0007155//cell adhesion;GO:0022610//biological adhesion;GO:0007165//signal transduction;GO:0016043//cellular component organization;GO:0008150//biological_process;GO:0048869//cellular developmental [illegible]

id	CON_Mean	KD_Mean	AllMean	log2FoldChange	pvalue	qvalue	State	gene_id	XF_1_count	XF_1_FPKM	XF_1_TPM	XF_1_CPM	XF_2_count	XF_2_FPKM	XF_2_TPM	XF_2_CPM	XF_3_count	XF_3_FPKM	XF_3_TPM	XF_3_CPM	Symbol	Description	KEGG_A_class	KEGG_B_class	Pathway	K_ID	GO Component	GO Function	GO Process
GPRC5D	1.05131527 4	-1.45184	-0.20026	-2.675	0.000178525	0.0153838 15	Down	ENSG00000111291	66	1.77008435	6.683329069	1.989574809	4	0.118563396	0.476165425	0.133265257	14	0.422448689	1.699468859	0.474832327	GPRC5D	G protein-coupled receptor class C group 5 member D [Source:HGNC Symbol;Acc:HGNC:13310]	-	-	-	-	GO:0005576//extracellular region;GO:0005622//intracellular;GO:0032991//macromolecular complex;GO:0016020//membrane;GO:0071944//cell periphery;GO:0044464//cell part;GO:0005615//extracellular space;GO:0044421//extracellular region part;GO:0005886//plasma membrane;GO:0043226//organelle;GO:0005575//cellular_component;GO:0005623//cell	GO:0003674//molecular_function;GO:0030234//enzyme regulator activity;GO:0098772//molecular function regulator	GO:0007154//cell communication;GO:0036211//protein modification process;GO:0050789//regulation of biological process;GO:0065007//biological regulation;GO:0050896//response to stimulus;GO:0009987//cellular process;GO:0044237//cellular metabolic process;GO:0050794//regulation of cellular process;GO:0019538//protein metabolic process;GO:0023052//signaling;GO:0043170//macromolecule metabolic process;GO:0071704//organic substance metabolic process;GO:0043412//macromolecule modification;GO:0044260//cellular macromolecule metabolic process;GO:0044700//single organism signaling;GO:0051716//cellular response to stimulus;GO:0006464//cellular protein modification process;GO:0044238//primary metabolic process;GO:0044699//single-organism process;GO:0008150//biological_process;GO:0044763//single-organism cellular process;GO:0044267//cellular protein metabolic process;GO:0007165//signal transduction;GO:0008152//metabolic process

id	CON_Mean	KD_Mean	AllMean	log2FoldChange	pvalue	qvalue	State	gene_id	XF_1_count	XF_1_FPKM	XF_1_TPM	XF_1_CPM	XF_2_count	XF_2_FPKM	XF_2_TPM	XF_2_CPM	XF_3_count	XF_3_FPKM	XF_3_TPM	XF_3_CPM	Symbol	Description	KEGG_A_class	KEGG_B_class	Pathway	K_ID	GO Component	GO Function	GO Process
SERPINF1	4.583565553	2.446721	3.515143277	-2.02918	0.000181876	0.01559381	Down	ENSG00000132386	798	8.045407406	30.377142875	24.055768144	103	1.147685742	4.609249461	3.431580368	241	2.733745597	10.997585341	8.173899335	SERPINF1	serpin family F member 1 [Source:HGNC Symbol;Acc:HGNC:8824]	-	-	-	-	GO:0005622//intracellular;GO:0044421//extracellular region part;GO:0005575//cellular_component;GO:0044424//intracellular part;GO:0005576//extracellular region;GO:0044464//cell part;GO:0044444//cytoplasmic part;GO:0031982//vesicle;GO:0005615//extracellular space;GO:0031410//cytoplasmic vesicle;GO:0043226//organelle;GO:0031012//extracellular matrix;GO:0005737//cytoplasm;GO:0043227//membrane-bounded organelle;GO:0097708//intracellular vesicle;GO:0005623//cell	GO:0003674//molecular_function;GO:0030234//enzyme regulator activity;GO:0098772//molecular function regulator	GO:0009987//cellular process;GO:0006950//response to stress;GO:0007568//aging;GO:0065007//biological regulation;GO:0000003//reproduction;GO:0051674//localization of cell;GO:0050896//response to stimulus;GO:0044763//single-organism cellular process;GO:0003008//system process;GO:0008150//biological_process;GO:0042592//homeostatic process;GO:0030154//cell differentiation;GO:0008283//cell proliferation;GO:0048869//cellular developmental process;GO:0008219//cell death;GO:0044699//single-organism process;GO:0065008//regulation of biological quality;GO:0006928//movement of cell or subcellular component;GO:0048646//anatomical structure formation involved in morphogenesis;GO:0050877//neurological system process;GO:0051179//localization;GO:0009653//anatomical structure morphogenesis;GO:0032501//multicellular organismal process;GO:0048870//cell motility;GO:0044767//single-organism developmental process;GO:0040011//locomotion;GO:0032502//developmental process;GO:0048856//anatomical structure development

id	CON_Mean	KD_Mean	AllMean	log2FoldChange	pvalue	qvalue	State	gene_id	XF_1_count	XF_1_FPKM	XF_1_TPM	XF_1_CPM	XF_2_count	XF_2_FPKM	XF_2_TPM	XF_2_CPM	XF_3_count	XF_3_FPKM	XF_3_TPM	XF_3_CPM	Symbol	Description	KEGG_A_class	KEGG_B_class	Pathway	K_ID	GO Component	GO Function	GO Process
E2F1	5.515116534	6.611688812	6.063402673	1.110541605	0.000191113	0.016278943	Up	ENSG00000101412	1525	17.0896788 53	64.5257088 93	45.9712361 14	3301	40.8837001 25	164.194052 371	109.977153 338	2512	31.6722996 35	127.414496 264	85.1984860 17	E2F1	E2F transcription factor 1 [Source:HGNC Symbol;Acc:HGNC:3113]	Human Diseases;Cellular Processes	Cancers;Cell growth and death;Infectious diseases	ko05200//Pathways in cancer;ko05166//HTLV-I infection;ko05206//MicroRNAs in cancer;ko05161//Hepatitis B;ko04110//Cell cycle;ko05222//Small cell lung cancer;ko05215//Prostate cancer;ko05[illegible]	K17454	GO:0043233//organelle lumen;GO:0043227//membrane-bounded organelle;GO:0015630//microtubule cytoskeleton;GO:0043229//intracellular organelle;GO:0032991//macromolecular complex;GO:0000228//nuclear chromosome;GO:0005623//cell;GO:0070013//intracellular organelle lumen;GO:0005737//cytoplasm;GO:0044424//intracellular part;GO:0044422//organelle part;GO:0044464//cell part;GO:0005856//cytoskeleton;GO:0044428//nuclear part;GO:0005654//nucleoplasm;GO:0043232//intracellular non-membrane-bounded organelle;GO:0031981//nuclear lumen;GO:0044444//cytoplasmic part;GO:0005622//intracellular;GO:0005739//mitochondrion;GO:0043231//intracellular membrane-bounded organelle;GO:0043228//non-membrane-bounded organelle;GO:0005634//nucleus;GO:0005815//microtubule organizing center;GO:0043226//organelle;GO:0031974//membrane-enclosed lumen;GO:0005694//chromosome;GO:0044446//intracellular organelle part;GO:0005575//cellular_component;GO:0044430//cytoskeletal part	GO:0005515//protein binding;GO:1901363//heterocyclic compound binding;GO:0019899//enzyme binding;GO:0001071//nucleic acid binding transcription factor activity;GO:0097159//organic cyclic compound binding;GO:0003676//nucleic acid binding;GO:0005488//binding;GO:0003674//molecular_function;GO:0008134//transcription factor binding;GO:0003700//transcription factor activity, sequence-specific DNA binding;GO:0003677//DNA binding	GO:0007165//signal transduction;GO:0048856//anatomical structure development;GO:0023052//signaling;GO:0044270//cellular nitrogen compound catabolic process;GO:0016043//cellular component organization;GO:0030154//cell differentiation;GO:0019439//aromatic compound catabolic process;GO:0000003//reproduction;GO:0044700//single organism signaling;GO:0032502//developmental process;GO:0051704//multi-organism process;GO:0009058//biosynthetic process;GO:0046700//heterocycle catabolic process;GO:0044419//interspecies interaction between organisms;GO:0048869//cellular developmental process;GO:0044237//cellular metabolic process;GO:0008152//metabolic process;GO:0007154//cell communication;GO:0006807//nitrogen compound metabolic process;GO:0034655//nucleobase-containing compound catabolic process;GO:0006950//response to stress;GO:0008219//cell death;GO:0051234//establishment of localization;GO:0008150//biological_process;GO:0044[illegible]

id	CON_Mean	KD_Mean	AllMean	log2FoldChange	pvalue	qvalue	State	gene_id	XF_1_count	XF_1_FPKM	XF_1_TPM	XF_1_CPM	XF_2_count	XF_2_FPKM	XF_2_TPM	XF_2_CPM	XF_3_count	XF_3_FPKM	XF_3_TPM	XF_3_CPM	Symbol	Description	KEGG_A_class	KEGG_B_class	Pathway	K_ID	GO Component	GO Function	GO Process
SESN2	4.162065323	5.451827557	4.80694644	1.302666798	0.000191775	0.016278943	Up	ENSG00000130766	595	5.180912291	19.561633732	17.936318353	1442	13.876985889	55.731710702	48.042125148	1149	11.256547478	45.283965584	38.97016737	SESN2	sestrin 2 [Source:HGNC Symbol;Acc:HGNC:20746]	Cellular Processes	Cell growth and death	ko04115//p53 signaling pathway	K10141	GO:0005622//intracellular;GO:0043231//intracellular membrane-bounded organelle;GO:0043229//intracellular organelle;GO:0005737//cytoplasm;GO:0005575//cellular_component;GO:0032991//macromolecular complex;GO:0043227//membrane-bounded organelle;GO:0005634//nucleus;GO:0005829//cytosol;GO:0044424//intracellular part;GO:0044444//cytoplasmic part;GO:0005623//cell;GO:0005739//mitochondrion;GO:0043226//organelle;GO:0044464//cell part	GO:0003824//catalytic activity;GO:0043167//ion binding;GO:0030234//enzyme regulator activity;GO:0005488//binding;GO:0003674//molecular_function;GO:0016491//oxidoreductase activity;GO:0098772//molecular function regulator	GO:0006412//translation;GO:0042592//homeostatic process;GO:0009987//cellular process;GO:0051234//establishment of localization;GO:0006996//organelle organization;GO:0034645//cellular macromolecule biosynthetic process;GO:0007165//signal transduction;GO:0043043//peptide biosynthetic process;GO:0010467//gene expression;GO:0006914//autophagy;GO:0006518//peptide metabolic process;GO:0043170//macromolecule metabolic process;GO:0044249//cellular biosynthetic process;GO:0007154//cell communication;GO:0006139//nucleobase-containing compound metabolic process;GO:0044271//cellular nitrogen compound biosynthetic process;GO:0051179//localization;GO:0090304//nucleic acid metabolic process;GO:0044700//single organism signaling;GO:0071704//organic substance metabolic process;GO:0046483//heterocycle metabolic process;GO:0034641//cellular nitrogen compound metabolic process;GO:0044710//single-organism metabolic process;GO:0023052//signaling;GO:0008152//metabolic process;GO:0016043//cellu [illegible]
YJEFN3	2.63271671	4.375547302	3.504132006	1.758451932	0.000201175	0.016992347	Up	ENSG00000250067	204	2.07826795	7.846941648	6.149594864	601	6.766848552	27.176510004	20.023104864	617	7.072165922	28.450616747	20.926538962	YJEFN3	YjeF N-terminal domain containing 3 [Source:HGNC Symbol;Acc:HGNC:24785]	-	-	-	-	GO:0044444//cytoplasmic part;GO:0005739//mitochondrion;GO:0044424//intracellular part;GO:0044464//cell part;GO:0005623//cell;GO:0043226//organelle;GO:0005737//cytoplasm;GO:0043231//intracellular membrane-bounded organelle;GO:0005622//intracellular;GO:0005575//cellular_component;GO:0043227//membrane-bounded organelle;GO:0043229//intracellular organelle	GO:0016853//isomerase activity;GO:0003824//catalytic activity;GO:0003674//molecular_function	-

id	CON_Mean	KD_Mean	AllMean	log2FoldChange	pvalue	qvalue	State	gene_id	XF_1_count	XF_1_FPKM	XF_1_TPM	XF_1_CPM	XF_2_count	XF_2_FPKM	XF_2_TPM	XF_2_CPM	XF_3_count	XF_3_FPKM	XF_3_TPM	XF_3_CPM	Symbol	Description	KEGG_A_class	KEGG_B_class	Pathway	K_ID	GO Component	GO Function	GO Process
MGP	3.560435205	0.843652057	2.202043631	-2.51673	0.000204283	0.017110565	Down	ENSG00000111341	391	4.08268912	15.415059108	11.786723489	26	0.30004301	1.205010251	0.86622417	94	1.104315867	4.442552371	3.188159907	MGP	matrix Gla protein [Source:HGNC Symbol;Acc:HGNC:7060]	-	-	-	-	GO:0005615//extracellular space;GO:0031012//extracellular matrix;GO:0005575//cellular_component;GO:0005576//extracellular region;GO:0043226//organelle;GO:0044421//extracellular region part	GO:0003674//molecular_function;GO:0005488//binding;GO:0043167//ion binding;GO:0005198//structural molecule activity	GO:0032502//developmental process;GO:0008150//biological_process;GO:0044767//single-organism developmental process;GO:0044763//single-organism cellular process;GO:0048869//cellular developmental process;GO:0048856//anatomical structure development;GO:0030154//cell differentiation;GO:0009987//cellular process;GO:0044699//single-organism process
BCL9L	6.290182436	7.31282823	6.801505333	1.025712576	0.000205023	0.017110565	Up	ENSG00000186174	2612	7.29875142	27.55798476	78.738930315	4975	15.364169762	61.704427113	165.748663391	4409	13.861537473	55.763580005	149.538266262	BCL9L	BCL9 like [Source:HGNC Symbol;Acc:HGNC:23688]	-	-	-	-	GO:0005654//nucleoplasm;GO:0043228//non-membrane-bounded organelle;GO:0043229//intracellular organelle;GO:0044464//cell part;GO:0005730//nucleolus;GO:0005575//cellular_component;GO:0044424//intracellular part;GO:0005634//nucleus;GO:0043232//intracellular non-membrane-bounded organelle;GO:0043226//organelle;GO:0044446//intracellular organelle part;GO:0005623//cell;GO:0070013//intracellular organelle lumen;GO:0043233//organelle lumen;GO:0043227//membrane-bounded organelle;GO:0032991//macromolecular complex;GO:0031974//membrane-enclosed lumen;GO:0005622//intracellular;GO:0044428//nuclear part;GO:0044422//organelle part;GO:0031981//nuclear lumen;GO:0043231//intracellular membrane-bounded organelle	GO:0003674//molecular_function	GO:0048856//anatomical structure development;GO:0007165//signal transduction;GO:0022607//cellular component assembly;GO:0009058//biosynthetic process;GO:0044763//single-organism cellular process;GO:0044700//single organism signaling;GO:0032502//developmental process;GO:0009987//cellular process;GO:0007154//cell communication;GO:0044767//single-organism developmental process;GO:0000902//cell morphogenesis;GO:0050794//regulation of cellular process;GO:0044085//cellular component biogenesis;GO:0006807//nitrogen compound metabolic process;GO:0065007//biological regulation;GO:0008152//metabolic process;GO:0034641//cellular nitrogen compound metabolic process;GO:0050789//regulation of biological process;GO:0050896//response to stimulus;GO:0008150//biological_process;GO:0048869//cellular developmental process;GO:0044699//single-organism process;GO:0032989//cellular component morphogenesis;GO:0043933//macromolecular complex [illegible]

id	CON_Mean	KD_Mean	AllMean	log2FoldChange	pvalue	qvalue	State	gene_id	XF_1_count	XF_1_FPKM	XF_1_TPM	XF_1_CPM	XF_2_count	XF_2_FPKM	XF_2_TPM	XF_2_CPM	XF_3_count	XF_3_FPKM	XF_3_TPM	XF_3_CPM	Symbol	Description	KEGG_A_class	KEGG_B_class	Pathway	K_ID	GO Component	GO Function	GO Process
ZC3H6	3.462011553	1.460440709	2.461226131	-2.00562	0.000205584	0.017110565	Down	ENSG00000188177	365	0.932453525	3.520676148	11.002951595	63	0.177875237	0.714369196	2.098927798	97	0.278805906	1.121608297	3.289909691	ZC3H6	zinc finger CCCH-type containing 6 [Source:HGNC Symbol;Acc:HGNC:24762]	-	-	-	-	GO:0043229//intracellular organelle;GO:0044446//intracellular organelle part;GO:0044424//intracellular part;GO:0005694//chromosome;GO:0044428//nuclear part;GO:0043228//non-membrane-bounded organelle;GO:0031974//membrane-enclosed lumen;GO:0044464//cell part;GO:0043231//intracellular membrane-bounded organelle;GO:0043232//intracellular non-membrane-bounded organelle;GO:0043227//membrane-bounded organelle;GO:0043233//organelle lumen;GO:0031981//nuclear lumen;GO:0070013//intracellular organelle lumen;GO:0044422//organelle part;GO:0000228//nuclear chromosome;GO:0005575//cellular_component;GO:0043226//organelle;GO:0005634//nucleus;GO:0005623//cell;GO:0005622//intracellular	GO:0001071//nucleic acid binding transcription factor activity;GO:0003674//molecular_function;GO:0003700//transcription factor activity, sequence-specific DNA binding;GO:0005488//binding;GO:0043167//ion binding	GO:0044237//cellular metabolic process;GO:0034641//cellular nitrogen compound metabolic process;GO:0008150//biological_process;GO:0009058//biosynthetic process;GO:0009987//cellular process;GO:0006807//nitrogen compound metabolic process;GO:0008152//metabolic process
ADAMTSL4	4.80357515	3.292557666	4.048066408	-1.51982	0.000208547	0.017257552	Down	ENSG00000143382	930	4.358662587	16.457055495	28.034917761	292	1.512494366	6.074366516	9.728363761	280	1.476468677	5.939685937	9.496646531	ADAMTSL4	ADAMTS like 4 [Source:HGNC Symbol;Acc:HGNC:19706]	-	-	-	-	GO:0044424//intracellular part;GO:0005576//extracellular region;GO:0005575//cellular_component;GO:0005622//intracellular;GO:0044444//cytoplasmic part;GO:0005737//cytoplasm;GO:0005783//endoplasmic reticulum;GO:0043231//intracellular membrane-bounded organelle;GO:0005623//cell;GO:0012505//endomembrane system;GO:0043227//membrane-bounded organelle;GO:0043226//organelle;GO:0044421//extracellular region part;GO:0043229//intracellular organelle;GO:0031012//extracellular matrix;GO:0044464//cell part	GO:0008233//peptidase activity;GO:0016787//hydrolase activity;GO:0005515//protein binding;GO:0005488//binding;GO:0019899//enzyme binding;GO:0003824//catalytic activity;GO:0003674//molecular_function	GO:0030154//cell differentiation;GO:0044767//single-organism developmental process;GO:0044699//single-organism process;GO:0043062//extracellular structure organization;GO:0071840//cellular component organization or biogenesis;GO:0009987//cellular process;GO:0048856//anatomical structure development;GO:0008219//cell death;GO:0048869//cellular developmental process;GO:0032502//developmental process;GO:0008150//biological_process;GO:0030198//extracellular matrix organization;GO:0044763//single-organism cellular process;GO:0016043//cellular component organization

id	CON_Mean	KD_Mean	AllMean	log2FoldChange	pvalue	qvalue	State	gene_id	XF_1_count	XF_1_FPKM	XF_1_TPM	XF_1_CPM	XF_2_count	XF_2_FPKM	XF_2_TPM	XF_2_CPM	XF_3_count	XF_3_FPKM	XF_3_TPM	XF_3_CPM	Symbol	Description	KEGG_A_class	KEGG_B_class	Pathway	K_ID	GO Component	GO Function	GO Process
FST	7.958470308	9.319962607	8.639216457	1.438904124	0.000209873	0.017257552	Up	ENSG00000134363	8309	91.015774131	343.649368535	250.475410408	13615	164.826169515	661.962508934	453.601618507	26057	321.135432216	1291.895751185	883.764709458	FST	follistatin [Source:HGNC Symbol;Acc:HGNC:3971]	Environmental Information Processing	Signal transduction	ko04350//TGF-beta signaling pathway	K04661	GO:0043226//organelle;GO:0005737//cytoplasm;GO:0043227//membrane-bounded organelle;GO:0043231//intracellular membrane-bounded organelle;GO:0005623//cell;GO:0044464//cell part;GO:0043229//intracellular organelle;GO:0005622//intracellular;GO:0005576//extracellular region;GO:0044421//extracellular region part;GO:0005575//cellular_component;GO:0005634//nucleus;GO:0044424//intracellular part;GO:0005615//extracellular space	GO:0043167//ion binding;GO:0003674//molecular_function;GO:0005488//binding	GO:0007154//cell communication;GO:0048869//cellular developmental process;GO:0050794//regulation of cellular process;GO:0006807//nitrogen compound metabolic process;GO:0050789//regulation of biological process;GO:0008283//cell proliferation;GO:0048856//anatomical structure development;GO:0032502//developmental process;GO:0034641//cellular nitrogen compound metabolic process;GO:0044767//single-organism developmental process;GO:0008152//metabolic process;GO:0044699//single-organism process;GO:0008150//biological_process;GO:0044700//single organism signaling;GO:0002376//immune system process;GO:0000003//reproduction;GO:0051716//cellular response to stimulus;GO:0044763//single-organism cellular process;GO:0044237//cellular metabolic process;GO:0009058//biosynthetic process;GO:0007165//signal transduction;GO:0009987//cellular process;GO:0023052//signaling;GO:0030154//cell differentiation;GO:0065007//biological regulation;GO:0050896//response to stimulus
FAM167B	2.4393317	0.417570985	1.428451342	-2.10106	0.000210384	0.017257552	Down	ENSG00000183615	178	5.66612774	21.393667618	5.365822969	36	1.266512474	5.086472478	1.199387313	37	1.325146785	5.33093309	1.254914006	-	-	-	-	-	-	-	-	-

id	CON_Mean	KD_Mean	AllMean	log2FoldChange	pvalue	qvalue	State	gene_id	XF_1_count	XF_1_FPKM	XF_1_TPM	XF_1_CPM	XF_2_count	XF_2_FPKM	XF_2_TPM	XF_2_CPM	XF_3_count	XF_3_FPKM	XF_3_TPM	XF_3_CPM	Symbol	Description	KEGG_A_class	KEGG_B_class	Pathway	K_ID	GO Component	GO Function	GO Process
POM121	6.183150792	7.212272606	6.697711699	1.03319742	0.000211417	0.017259343	Up	ENSG00000196313	2425	7.422256238	28.02430341	73.10180169	4690	15.864911547	63.715468744	156.25351383	4068	14.008803681	56.35601724	137.972707452	POM121	POM121 transmembrane nucleoporin [Source:HGNC Symbol;Acc:HGNC:19702]	Genetic Information Processing	Translation	ko03013//RNA transport	K14316	GO:0044464//cell part;GO:0070013//intracellular organelle lumen;GO:0043229//intracellular organelle;GO:0043227//membrane-bounded organelle;GO:0005634//nucleus;GO:0031974//membrane-enclosed lumen;GO:0044446//intracellular organelle part;GO:0044422//organelle part;GO:0005622//intracellular;GO:0032991//macromolecular complex;GO:0043233//organelle lumen;GO:0043231//intracellular membrane-bounded organelle;GO:0005623//cell;GO:0005635//nuclear envelope;GO:0044428//nuclear part;GO:0005575//cellular_component;GO:0005654//nucleoplasm;GO:0031981//nuclear lumen;GO:0044444//cytoplasmic part;GO:0043226//organelle;GO:0031967//organelle envelope;GO:0005783//endoplasmic reticulum;GO:0044424//intracellular part;GO:0031975//envelope;GO:0012505//endomembrane system;GO:0005737//cytoplasm	GO:0005198//structural molecule activity;GO:0003674//molecular_function	GO:0008152//metabolic process;GO:0008104//protein localization;GO:0044237//cellular metabolic process;GO:0044699//single-organism process;GO:0006464//cellular protein modification process;GO:0009056//catabolic process;GO:0044267//cellular protein metabolic process;GO:0044419//interspecies interaction between organisms;GO:0033036//macromolecule localization;GO:0006913//nucleocytoplasmic transport;GO:0051179//localization;GO:0009987//cellular process;GO:0051234//establishment of localization;GO:0071702//organic substance transport;GO:0006091//generation of precursor metabolites and energy;GO:0051649//establishment of localization in cell;GO:0071704//organic substance metabolic process;GO:0051641//cellular localization;GO:0043170//macromolecule metabolic process;GO:0006810//transport;GO:0044403//symbiosis, encompassing mutualism through parasitism;GO:0050896//response to stimulus;GO:0008150//biological_process;GO:0051704//multi-organism process;GO:0006950//resp

id	CON_Mean	KD_Mean	AllMean	log2FoldChange	pvalue	qvalue	State	gene_id	XF_1_count	XF_1_FPKM	XF_1_TPM	XF_1_CPM	XF_2_count	XF_2_FPKM	XF_2_TPM	XF_2_CPM	XF_3_count	XF_3_FPKM	XF_3_TPM	XF_3_CPM	Symbol	Description	KEGG_A_class	KEGG_B_class	Pathway	K_ID	GO Component	GO Function	GO Process
NRGN	4.102996399	5.378738731	4.740867565	1.28396429	0.000213637	0.017357491	Up	ENSG00000154146	571	14.190302229	53.578497211	17.212836604	1320	36.255181211	145.605341597	43.977534809	1134	31.707682151	127.556836591	38.461418449	NRGN	neurogranin [Source:HGNC Symbol;Acc:HGNC:8000]	-	-	-	-	GO:0005739//mitochondrion;GO:0044424//intracellular part;GO:0005622//intracellular;GO:0071944//cell periphery;GO:0044464//cell part;GO:0005829//cytosol;GO:0016020//membrane;GO:0097708//intracellular vesicle;GO:0005886//plasma membrane;GO:0005623//cell;GO:0043231//intracellular membrane-bounded organelle;GO:0031982//vesicle;GO:0043227//membrane-bounded organelle;GO:0005634//nucleus;GO:0005737//cytoplasm;GO:0031410//cytoplasmic vesicle;GO:0043226//organelle;GO:0043229//intracellular organelle;GO:0044444//cytoplasmic part;GO:0005575//cellular_component	GO:0005488//binding;GO:0003674//molecular_function;GO:0003824//catalytic activity;GO:0016301//kinase activity;GO:0016772//transferase activity, transferring phosphorus-containing groups;GO:0043167//ion binding;GO:0016740//transferase activity;GO:0008289//lipid binding	GO:0007154//cell communication;GO:0048856//anatomical structure development;GO:0065007//biological regulation;GO:0051716//cellular response to stimulus;GO:0032502//developmental process;GO:0044763//single-organism cellular process;GO:0023052//signaling;GO:0003008//system process;GO:0044700//single organism signaling;GO:0007165//signal transduction;GO:0009987//cellular process;GO:0050896//response to stimulus;GO:0050877//neurological system process;GO:0007267//cell-cell signaling;GO:0008150//biological_process;GO:0032501//multicellular organismal process;GO:0050789//regulation of biological process;GO:0050794//regulation of cellular process;GO:0044699//single-organism process
PRR22	0.195064024	2.800202836	1.49763343	2.835229857	0.000217056	0.017551732	Up	ENSG00000212123	35	0.590418327	2.229249678	1.05507755	312	5.816838302	23.36115004	10.394690046	131	2.486331233	10.002262077	4.443073913	-	-	-	-	-	-	-	-	-

id	CON_Mean	KD_Mean	AllMean	log2FoldChange	pvalue	qvalue	State	gene_id	XF_1_count	XF_1_FPKM	XF_1_TPM	XF_1_CPM	XF_2_count	XF_2_FPKM	XF_2_TPM	XF_2_CPM	XF_3_count	XF_3_FPKM	XF_3_TPM	XF_3_CPM	Symbol	Description	KEGG_A_class	KEGG_B_class	Pathway	K_ID	GO Component	GO Function	GO Process
BTBD8	2.784541519	0.780423083	1.782482301	-2.05705	0.000218808	0.017609922	Down	ENSG00000189195	227	0.820298674	3.097211709	6.84293154	43	0.171733579	0.689703528	1.432601513	53	0.215485438	0.866876382	1.797579522	BTBD8	BTB domain containing 8 [Source:HGNC Symbol;Acc:HGNC:21019]	-	-	-	-	GO:0032991//macromolecular complex;GO:0005575//cellular_component;GO:0043233//organelle lumen;GO:0071944//cell periphery;GO:0005654//nucleoplasm;GO:0031410//cytoplasmic vesicle;GO:0016020//membrane;GO:0070013//intracellular organelle lumen;GO:0031982//vesicle;GO:0044446//intracellular organelle part;GO:0044422//organelle part;GO:0005737//cytoplasm;GO:0043226//organelle;GO:0043229//intracellular organelle;GO:0044424//intracellular part;GO:0097708//intracellular vesicle;GO:0044444//cytoplasmic part;GO:0031981//nuclear lumen;GO:0005634//nucleus;GO:0044464//cell part;GO:0044428//nuclear part;GO:0043231//intracellular membrane-bounded organelle;GO:0031974//membrane-enclosed lumen;GO:0043227//membrane-bounded organelle;GO:0005623//cell;GO:0005622//intracellular;GO:0005886//plasma membrane	GO:0003674//molecular_function	GO:0009987//cellular process;GO:0008150//biological_process;GO:0051234//establishment of localization;GO:0044763//single-organism cellular process;GO:0048869//cellular developmental process;GO:0032502//developmental process;GO:0048856//anatomical structure development;GO:0051179//localization;GO:0044699//single-organism process;GO:0006810//transport;GO:0016192//vesicle-mediated transport;GO:0044767//single-organism developmental process;GO:0030154//cell differentiation

id	CON_Mean	KD_Mean	AllMean	log2FoldChange	pvalue	qvalue	State	gene_id	XF_1_count	XF_1_FPKM	XF_1_TPM	XF_1_CPM	XF_2_count	XF_2_FPKM	XF_2_TPM	XF_2_CPM	XF_3_count	XF_3_FPKM	XF_3_TPM	XF_3_CPM	Symbol	Description	KEGG_A_class	KEGG_B_class	Pathway	K_ID	GO Component	GO Function	GO Process
PLP1	7.13811655	5.859821817	6.498969183	-1.21664	0.00022597	0.018080369	Down	ENSG00000123560	4704	23.292119373	87.944339218	141.802422741	1275	6.977381844	28.022038036	42.478300668	2290	12.757720431	51.323034352	77.669001982	PLP1	proteolipid protein 1 [Source:HGNC Symbol;Acc:HGNC:9086]	-	-	-	-	GO:0016020//membrane;GO:0044464//cell part;GO:0005886//plasma membrane;GO:0071944//cell periphery;GO:0005623//cell;GO:0005575//cellular_component	GO:0005198//structural molecule activity;GO:0003674//molecular_function	GO:0048869//cellular developmental process;GO:0009987//cellular process;GO:0071704//organic substance metabolic process;GO:0023052//signaling;GO:0044699//single-organism process;GO:0044710//single-organism metabolic process;GO:0032502//developmental process;GO:0044281//small molecule metabolic process;GO:0006950//response to stress;GO:0065007//biological regulation;GO:0007267//cell-cell signaling;GO:0048856//anatomical structure development;GO:0044238//primary metabolic process;GO:0006629//lipid metabolic process;GO:0008152//metabolic process;GO:0008150//biological_process;GO:0044700//single organism signaling;GO:0009058//biosynthetic process;GO:0050896//response to stimulus;GO:0044767//single-organism developmental process;GO:0050794//regulation of cellular process;GO:0030154//cell differentiation;GO:0051716//cellular response to stimulus;GO:0050789//regulation of biological process;GO:0007165//signal transduction;GO:0044763//[illegible]

id	CON_Mean	KD_Mean	AllMean	log2FoldChange	pvalue	qvalue	State	gene_id	XF_1_count	XF_1_FPKM	XF_1_TPM	XF_1_CPM	XF_2_count	XF_2_FPKM	XF_2_TPM	XF_2_CPM	XF_3_count	XF_3_FPKM	XF_3_TPM	XF_3_CPM	Symbol	Description	KEGG_A_class	KEGG_B_class	Pathway	K_ID	GO Component	GO Function	GO Process
WDR72	5.914909105	4.797720285	5.356314695	-1.11375	0.000226773	0.018080369	Down	ENSG00000166415	2013	7.078272678	26.725520487	60.682031671	749	2.910756955	11.689963932	24.953919373	891	3.524983777	14.180657468	30.219685924	WDR72	WD repeat domain 72 [Source:HGNC Symbol;Acc:HGNC:26790]	-	-	-	-	GO:0043226//organelle;GO:0097708//intracellular vesicle;GO:0005768//endosome;GO:0005773//vacuole;GO:0005622//intracellular;GO:0005623//cell;GO:0005737//cytoplasm;GO:0005575//cellular_component;GO:0031410//cytoplasmic vesicle;GO:0043231//intracellular membrane-bounded organelle;GO:0044444//cytoplasmic part;GO:0012505//endomembrane system;GO:0044464//cell part;GO:0043227//membrane-bounded organelle;GO:0044424//intracellular part;GO:0031982//vesicle;GO:0043229//intracellular organelle	GO:0003674//molecular_function	GO:0048646//anatomical structure formation involved in morphogenesis;GO:0009653//anatomical structure morphogenesis;GO:0044763//single-organism cellular process;GO:0032502//developmental process;GO:0030198//extracellular matrix organization;GO:0008150//biological_process;GO:0009987//cellular process;GO:0016043//cellular component organization;GO:0043062//extracellular structure organization;GO:0071840//cellular component organization or biogenesis;GO:0048856//anatomical structure development;GO:0044699//single-organism process
ENSG00000242021	-0.29405	-2.46264	-1.37834	-3.02287	0.000231662	0.018384258	Down	ENSG00000242021	24	0.815650224	3.079660499	0.723481749	3	0.11268201	0.452545043	0.099948943	2	0.076474847	0.307650668	0.06783319	-	-	-	-	-	-	-	-	-
FRMD8	6.142173614	7.176691087	6.659432351	1.040688069	0.000233036	0.018407697	Up	ENSG00000126391	2357	17.461768674	65.93061297	71.051936735	3935	32.21914391	129.396111075	131.099696572	4615	38.467703313	154.751726165	156.525084781	FRMD8	FERM domain containing 8 [Source:HGNC Symbol;Acc:HGNC:25462]	-	-	-	-	GO:0043228//non-membrane-bounded organelle;GO:0071944//cell periphery;GO:0005829//cytosol;GO:0005575//cellular_component;GO:0005886//plasma membrane;GO:0043226//organelle;GO:0016020//membrane;GO:0005737//cytoplasm;GO:0005622//intracellular;GO:0043229//intracellular organelle;GO:0005856//cytoskeleton;GO:0044424//intracellular part;GO:0044464//cell part;GO:0043232//intracellular non-membrane-bounded organelle;GO:0044444//cytoplasmic part;GO:0005623//cell	GO:0003674//molecular_function	GO:0071702//organic substance transport;GO:0008150//biological_process;GO:0051234//establishment of localization;GO:0051179//localization;GO:0045184//establishment of protein localization;GO:0006810//transport;GO:0015031//protein transport;GO:0008104//protein localization;GO:0033036//macromolecule localization

id	CON_Mean	KD_Mean	AllMean	log2FoldChange	pvalue	qvalue	State	gene_id	XF_1_count	XF_1_FPKM	XF_1_TPM	XF_1_CPM	XF_2_count	XF_2_FPKM	XF_2_TPM	XF_2_CPM	XF_3_count	XF_3_FPKM	XF_3_TPM	XF_3_CPM	Symbol	Description	KEGG_A_class	KEGG_B_class	Pathway	K_ID	GO Component	GO Function	GO Process
SLC1A5	7.030617876	8.209754093	7.620185984	1.216899247	0.000234543	0.018441347	Up	ENSG00000105281	4366	39.499816361	149.139938426	131.613388114	11016	110.147814457	442.367397278	367.012517772	6908	70.316877716	282.877771943	234.295836548	SLC1A5	solute carrier family 1 member 5 [Source:HGNC Symbol;Acc:HGNC:10943]	Human Diseases;Organismal Systems	Cancers;Digestive system	ko04974//Protein digestion and absorption;ko05230//Central carbon metabolism in cancer	K05616	GO:0031410//cytoplasmic vesicle;GO:0031982//vesicle;GO:0016020//membrane;GO:0005615//extracellular space;GO:0043227//membrane-bounded organelle;GO:0005886//plasma membrane;GO:0097708//intracellular vesicle;GO:0044464//cell part;GO:0005737//cytoplasm;GO:0005576//extracellular region;GO:0044444//cytoplasmic part;GO:0044424//intracellular part;GO:0005622//intracellular;GO:0071944//cell periphery;GO:0043226//organelle;GO:0005575//cellular_component;GO:0044421//extracellular region part;GO:0005623//cell	GO:0022857//transmembrane transporter activity;GO:0005215//transporter activity;GO:0003674//molecular_function;GO:0043167//ion binding;GO:0005488//binding	GO:0055085//transmembrane transport;GO:0016043//cellular component organization;GO:0071840//cellular component organization or biogenesis;GO:0043933//macromolecular complex subunit organization;GO:0044419//interspecies interaction between organisms;GO:0065003//macromolecular complex assembly;GO:0006810//transport;GO:0022607//cellular component assembly;GO:0008150//biological_process;GO:0051179//localization;GO:0051704//multi-organism process;GO:0044085//cellular component biogenesis;GO:0051234//establishment of localization;GO:0009987//cellular process;GO:0044403//symbiosis, encompassing mutualism through parasitism
NOP56	7.286521458	8.330787471	7.808654464	1.053019153	0.000235787	0.018454136	Up	ENSG00000101361	5214	31.752810081	119.889472322	157.176409901	10619	71.471907276	287.040117462	353.785941015	8476	58.076173155	233.634640771	287.477057119	NOP56	NOP56 ribonucleoprotein [Source:HGNC Symbol;Acc:HGNC:15911]	Genetic Information Processing	Translation	ko03008//Ribosome biogenesis in eukaryotes	K14564	GO:0043227//membrane-bounded organelle;GO:0043229//intracellular organelle;GO:0005622//intracellular;GO:0044422//organelle part;GO:0005730//nucleolus;GO:0043233//organelle lumen;GO:0043232//intracellular non-membrane-bounded organelle;GO:0044464//cell part;GO:0043228//non-membrane-bounded organelle;GO:0005737//cytoplasm;GO:0005634//nucleus;GO:0070013//intracellular organelle lumen;GO:0031974//membrane-enclosed lumen;GO:0005623//cell;GO:0044446//intracellular organelle part;GO:0032991//macromolecular complex;GO:0043226//organelle;GO:0043231//intracellular membrane-bounded organelle;GO:0005575//cellular_component;GO:0044424//intracellular part;GO:0031981//nuclear lumen;GO:0005654//nucleoplasm;GO:0044428//nuclear part	GO:0005515//protein binding;GO:0097159//organic cyclic compound binding;GO:1901363//heterocyclic compound binding;GO:0003676//nucleic acid binding;GO:0005488//binding;GO:0019899//enzyme binding;GO:0003723//RNA binding;GO:0003674//molecular_function	GO:0008150//biological_process;GO:0044237//cellular metabolic process;GO:0044085//cellular component biogenesis;GO:0022613//ribonucleoprotein complex biogenesis;GO:0006807//nitrogen compound metabolic process;GO:0009987//cellular process;GO:0008152//metabolic process;GO:0071840//cellular component organization or biogenesis;GO:0034641//cellular nitrogen compound metabolic process;GO:0042254//ribosome biogenesis

id	CON_Mean	KD_Mean	AllMean	log2FoldChange	pvalue	qvalue	State	gene_id	XF_1_count	XF_1_FPKM	XF_1_TPM	XF_1_CPM	XF_2_count	XF_2_FPKM	XF_2_TPM	XF_2_CPM	XF_3_count	XF_3_FPKM	XF_3_TPM	XF_3_CPM	Symbol	Description	KEGG_A_class	KEGG_B_class	Pathway	K_ID	GO Component	GO Function	GO Process
SF3B4	6.611751243	7.659994006	7.135872624	1.059842425	0.000237452	0.018499542	Up	ENSG00000143368	3265	57.760365548	218.086516721	98.423662893	6774	132.444080238	531.911988889	225.684712726	5241	104.317413789	419.658530728	177.756873096	SF3B4	splicing factor 3b subunit 4 [Source:HGNC Symbol;Acc:HGNC:10771]	Genetic Information Processing	Transcription	ko03040//Spliceosome	K12831	GO:0005575//cellular_component;GO:0044424//intracellular part;GO:0043229//intracellular organelle;GO:0032991//macromolecular complex;GO:0043232//intracellular non-membrane-bounded organelle;GO:0043231//intracellular membrane-bounded organelle;GO:0031981//nuclear lumen;GO:0044422//organelle part;GO:0044464//cell part;GO:0031974//membrane-enclosed lumen;GO:0005654//nucleoplasm;GO:0070013//intracellular organelle lumen;GO:0044446//intracellular organelle part;GO:0043228//non-membrane-bounded organelle;GO:0044428//nuclear part;GO:0005730//nucleolus;GO:0005634//nucleus;GO:0043227//membrane-bounded organelle;GO:0043226//organelle;GO:0043233//organelle lumen;GO:0005623//cell;GO:0005622//intracellular	GO:0005488//binding;GO:0003676//nucleic acid binding;GO:0003674//molecular_function;GO:0003723//RNA binding;GO:1901363//heterocyclic compound binding;GO:0097159//organic cyclic compound binding	GO:0071704//organic substance metabolic process;GO:0090304//nucleic acid metabolic process;GO:0044237//cellular metabolic process;GO:0006396//RNA processing;GO:0046483//heterocycle metabolic process;GO:1901360//organic cyclic compound metabolic process;GO:0010467//gene expression;GO:0006807//nitrogen compound metabolic process;GO:0043170//macromolecule metabolic process;GO:0009987//cellular process;GO:0008150//biological_process;GO:0006397//mRNA processing;GO:0006725//cellular aromatic compound metabolic process;GO:0008152//metabolic process;GO:0016071//mRNA metabolic process;GO:0016070//RNA metabolic process;GO:0006139//nucleobase-containing compound metabolic process;GO:0034641//cellular nitrogen compound metabolic process;GO:0044260//cellular macromolecule metabolic process;GO:0044238//primary metabolic process

id	CON_Mean	KD_Mean	AllMean	log2FoldChange	pvalue	qvalue	State	gene_id	XF_1_count	XF_1_FPKM	XF_1_TPM	XF_1_CPM	XF_2_count	XF_2_FPKM	XF_2_TPM	XF_2_CPM	XF_3_count	XF_3_FPKM	XF_3_TPM	XF_3_CPM	Symbol	Description	KEGG_A_class	KEGG_B_class	Pathway	K_ID	GO Component	GO Function	GO Process
PPRC1	6.181962047	7.264519322	6.723240684	1.09930449	0.000241059	0.018610798	Up	ENSG00000148840	2423	11.9799100 45	45.23269248	73.0415115 44	5280	28.8519172 11	115.872907 566	175.910139 237	3885	21.6116074 48	86.9413370 12	131.765970 612	PPRC1	PPARG related coactivator 1 [Source:HGNC Symbol;Acc:HGNC:30025]	-	-	-	-	GO:0005654//nucleoplasm;GO:0031974//membrane-enclosed lumen;GO:0044464//cell part;GO:0043229//intracellular organelle;GO:0043227//membrane-bounded organelle;GO:0044424//intracellular part;GO:0005623//cell;GO:0043231//intracellular membrane-bounded organelle;GO:0044428//nuclear part;GO:0070013//intracellular organelle lumen;GO:0005622//intracellular;GO:0005634//nucleus;GO:0044422//organelle part;GO:0043233//organelle lumen;GO:0043226//organelle;GO:0044446//intracellular organelle part;GO:0031981//nuclear lumen;GO:0005575//cellular_component	GO:0005488//binding;GO:1901363//heterocyclic compound binding;GO:0003676//nucleic acid binding;GO:0097159//organic cyclic compound binding;GO:0003674//molecular_function;GO:0003723//RNA binding;GO:0005515//protein binding;GO:0008134//transcription factor binding	GO:0044237//cellular metabolic process;GO:0007005//mitochondrion organization;GO:0009058//biosynthetic process;GO:0006807//nitrogen compound metabolic process;GO:0009987//cellular process;GO:0034641//cellular nitrogen compound metabolic process;GO:0008152//metabolic process;GO:0006996//organelle organization;GO:0071840//cellular component organization or biogenesis;GO:0008150//biological_process;GO:0016043//cellular component organization

id	CON_Mean	KD_Mean	AllMean	log2FoldChange	pvalue	qvalue	State	gene_id	XF_1_count	XF_1_FPKM	XF_1_TPM	XF_1_CPM	XF_2_count	XF_2_FPKM	XF_2_TPM	XF_2_CPM	XF_3_count	XF_3_FPKM	XF_3_TPM	XF_3_CPM	Symbol	Description	KEGG_A_class	KEGG_B_class	Pathway	K_ID	GO Component	GO Function	GO Process
CCN2	5.439537896	6.739652	6.089594818	1.367336416	0.000242152	0.018610798	Up	ENSG00000118523	1447	18.656937738	70.4432273 81	43.619920431	4285	61.060909563	245.228248713	142.760406559	2311	33.524914659	134.867381365	78.381250472	CCN2	cellular communication network factor 2 [Source:HGNC Symbol;Acc:HGNC:2500]	Environmental Information Processing	Signal transduction	ko04390//Hippo signaling pathway	K06827	GO:0005576//extracellular region;GO:0016020//membrane;GO:0005622//intracellular;GO:0005615//extracellular space;GO:0044444//cytoplasmic part;GO:0043227//membrane-bounded organelle;GO:0005575//cellular_component;GO:0044424//intracellular part;GO:0005737//cytoplasm;GO:0005794//Golgi apparatus;GO:0012505//endomembrane system;GO:0005623//cell;GO:0043229//intracellular organelle;GO:0043226//organelle;GO:0044421//extracellular region part;GO:0031012//extracellular matrix;GO:0043231//intracellular membrane-bounded organelle;GO:0071944//cell periphery;GO:0005886//plasma membrane;GO:0044464//cell part;GO:0005829//cytosol	GO:0043167//ion binding;GO:0005488//binding;GO:0003674//molecular_function	GO:0007165//signal transduction;GO:0065007//biological regulation;GO:0006807//nitrogen compound metabolic process;GO:0023052//signaling;GO:0050896//response to stimulus;GO:0006928//movement of cell or subcellular component;GO:0048856//anatomical structure development;GO:0032501//multicellular organismal process;GO:0048870//cell motility;GO:0040011//locomotion;GO:0009987//cellular process;GO:0030198//extracellular matrix organization;GO:0043412//macromolecule modification;GO:0046483//heterocycle metabolic process;GO:0006810//transport;GO:0007049//cell cycle;GO:0009058//biosynthetic process;GO:0065008//regulation of biological quality;GO:0008152//metabolic process;GO:0008219//cell death;GO:0044699//single-organism process;GO:0009653//anatomical structure morphogenesis;GO:0050789//regulation of biological process;GO:0044237//cellular metabolic process;GO:0050794//regulation of cellular process;GO:0032502//developmental process;GO:0022610//biological adhesion;GO:0044700//sin [illegible]
MEMO1P1	0.232291065	2.63523426	1.433762662	2.539759358	0.000244133	0.018678931	Up	ENSG00000226054	36	1.222097548	4.61428862	1.085222623	143	5.365127182	21.547021693	4.764232938	227	8.670120505	34.879028346	7.699067009	-	-	-	-	-	-	-	-	-

id	CON_Mean	KD_Mean	AllMean	log2FoldChange	pvalue	qvalue	State	gene_id	XF_1_count	XF_1_FPKM	XF_1_TPM	XF_1_CPM	XF_2_count	XF_2_FPKM	XF_2_TPM	XF_2_CPM	XF_3_count	XF_3_FPKM	XF_3_TPM	XF_3_CPM	Symbol	Description	KEGG_A_class	KEGG_B_class	Pathway	K_ID	GO Component	GO Function	GO Process
TONSL	5.564301253	6.603917925	6.084109589	1.046378581	0.000247842	0.018878048	Up	ENSG00000160949	1578	6.69324961	25.271784191	47.568924976	3137	14.705681413	59.059855557	104.513277801	2615	12.479512491	50.203831615	88.691895277	TONSL	tonsoku like, DNA repair protein [Source:HGNC Symbol;Acc:HGNC:7801]	-	-	-	-	GO:0044446//intracellular organelle part;GO:0043231//intracellular membrane-bounded organelle;GO:0044422//organelle part;GO:0031981//nuclear lumen;GO:0043229//intracellular organelle;GO:0031974//membrane-enclosed lumen;GO:0044428//nuclear part;GO:0005737//cytoplasm;GO:0043227//membrane-bounded organelle;GO:0043228//non-membrane-bounded organelle;GO:0043232//intracellular non-membrane-bounded organelle;GO:0005622//intracellular;GO:0005694//chromosome;GO:0044464//cell part;GO:0005623//cell;GO:0044424//intracellular part;GO:0005575//cellular_component;GO:0032991//macromolecular complex;GO:0000228//nuclear chromosome;GO:0005654//nucleoplasm;GO:0005634//nucleus;GO:0043233//organelle lumen;GO:0043226//organelle;GO:0070013//intracellular organelle lumen	GO:0003674//molecular_function;GO:0042393//histone binding;GO:0005515//protein binding;GO:0005488//binding	GO:0006139//nucleobase-containing compound metabolic process;GO:0006950//response to stress;GO:0034641//cellular nitrogen compound metabolic process;GO:0044238//primary metabolic process;GO:1901360//organic cyclic compound metabolic process;GO:0044237//cellular metabolic process;GO:0006807//nitrogen compound metabolic process;GO:0009987//cellular process;GO:0006259//DNA metabolic process;GO:0008152//metabolic process;GO:0009058//biosynthetic process;GO:0050896//response to stimulus;GO:0006725//cellular aromatic compound metabolic process;GO:0008150//biological_process;GO:0090304//nucleic acid metabolic process;GO:0044260//cellular macromolecule metabolic process;GO:0046483//heterocycle metabolic process;GO:0071704//organic substance metabolic process;GO:0043170//macromolecule metabolic process
PYY2	-2.66856	0.019064888	-1.32475	3.872992488	0.000253525	0.019225097	Up	ENSG00000237575	2	0.056031734	0.211559701	0.060290146	33	1.021782872	4.103607792	1.09943837	22	0.693461974	2.789728222	0.746165085	-	-	-	-	-	-	-	-	-

id	CON_Mean	KD_Mean	AllMean	log2FoldChange	pvalue	qvalue	State	gene_id	XF_1_count	XF_1_FPKM	XF_1_TPM	XF_1_CPM	XF_2_count	XF_2_FPKM	XF_2_TPM	XF_2_CPM	XF_3_count	XF_3_FPKM	XF_3_TPM	XF_3_CPM	Symbol	Description	KEGG_A_class	KEGG_B_class	Pathway	K_ID	GO Component	GO Function	GO Process
SPP1	4.049249575	2.087009573	3.068129574	-1.92323	0.000258153	0.019489431	Down	ENSG00000118785	550	2.286867596	8.634553877	16.5797900 74	166	0.762828712	3.063615504	5.530508165	90	0.42103359	1.693776057	3.052493528	SPP1	secreted phosphoprotein 1 [Source:HGNC Symbol;Acc:HGNC:11255]	Organismal Systems;Cellular Processes;Environmental Information Processing	Cellular commiunity;Signaling molecules and interaction;Immune system;Signal transduction	ko04151//PI3K-Akt signaling pathway;ko04510//Focal adhesion;ko04620//Toll-like receptor signaling pathway;ko04512//ECM-receptor interaction	K06250	GO:0005623//cell;GO:0043229//intracellular organelle;GO:0043231//intracellular membrane-bounded organelle;GO:0043226//organelle;GO:0005576//extracellular region;GO:0005783//endoplasmic reticulum;GO:0005622//intracellular;GO:0043227//membrane-bounded organelle;GO:0005615//extracellular space;GO:0012505//endomembrane system;GO:0005575//cellular_component;GO:0005794//Golgi apparatus;GO:0005737//cytoplasm;GO:0044421//extracellular region part;GO:0044444//cytoplasmic part;GO:0044424//intracellular part;GO:0044464//cell part	GO:0003674//molecular_function	GO:0006810//transport;GO:0006807//nitrogen compound metabolic process;GO:0022610//biological adhesion;GO:0048869//cellular developmental process;GO:0043412//macromolecule modification;GO:0042592//homeostatic process;GO:0044238//primary metabolic process;GO:0048856//anatomical structure development;GO:0032502//developmental process;GO:0051716//cellular response to stimulus;GO:0065007//biological regulation;GO:0044763//single-organism cellular process;GO:0040007//growth;GO:0050789//regulation of biological process;GO:0051179//localization;GO:0044767//single-organism developmental process;GO:0030198//extracellular matrix organization;GO:0009653//anatomical structure morphogenesis;GO:0044700//single organism signaling;GO:0043170//macromolecule metabolic process;GO:0008152//metabolic process;GO:0071840//cellular component organization or biogenesis;GO:0000902//cell morphogenesis;GO:0007154//cell communication;GO:0036211//protein modification [illegible]

id	CON_Mean	KD_Mean	AllMean	log2FoldChange	pvalue	qvalue	State	gene_id	XF_1_count	XF_1_FPKM	XF_1_TPM	XF_1_CPM	XF_2_count	XF_2_FPKM	XF_2_TPM	XF_2_CPM	XF_3_count	XF_3_FPKM	XF_3_TPM	XF_3_CPM	Symbol	Description	KEGG_A_class	KEGG_B_class	Pathway	K_ID	GO Component	GO Function	GO Process
SETD1B	5.108943133	6.150819255	5.629881194	1.044140432	0.000262284	0.019673936	Up	ENSG00000139718	1150	4.051283603	15.296480915	34.666833791	2142	8.33978557	33.493621772	71.363545122	2042	8.093687798	32.560097168	69.257686484	SETD1B	SET domain containing 1B, histone lysine methyltransferase [Source:HGNC Symbol;Acc:HGNC:29187]	Metabolism	Amino acid metabolism	ko00310//Lysine degradation	K11422	GO:0043231//intracellular membrane-bounded organelle;GO:0005634//nucleus;GO:0031981//nuclear lumen;GO:0005575//cellular_component;GO:0044424//intracellular part;GO:0005622//intracellular;GO:0044422//organelle part;GO:0044464//cell part;GO:0031974//membrane-enclosed lumen;GO:0043227//membrane-bounded organelle;GO:0043228//non-membrane-bounded organelle;GO:0043232//intracellular non-membrane-bounded organelle;GO:0005654//nucleoplasm;GO:0044446//intracellular organelle part;GO:0044428//nuclear part;GO:0005694//chromosome;GO:0032991//macromolecular complex;GO:0043226//organelle;GO:0070013//intracellular organelle lumen;GO:0043233//organelle lumen;GO:0005623//cell;GO:0043229//intracellular organelle	GO:0003674//molecular_function;GO:0003824//catalytic activity;GO:0016740//transferase activity;GO:0008168//methyltransferase activity;GO:1901363//heterocyclic compound binding;GO:0003723//RNA binding;GO:0003676//nucleic acid binding;GO:0016741//transferase activity, transferring one-carbon groups;GO:0005488//binding;GO:0097159//organic cyclic compound binding	GO:0051276//chromosome organization;GO:0071704//organic substance metabolic process;GO:0009987//cellular process;GO:0043412//macromolecule modification;GO:0044237//cellular metabolic process;GO:0036211//protein modification process;GO:0019538//protein metabolic process;GO:0071840//cellular component organization or biogenesis;GO:0008152//metabolic process;GO:0016043//cellular component organization;GO:0006464//cellular protein modification process;GO:0006996//organelle organization;GO:0044267//cellular protein metabolic process;GO:0044260//cellular macromolecule metabolic process;GO:0043170//macromolecule metabolic process;GO:0044238//primary metabolic process;GO:0008150//biological_process

id	CON_Mean	KD_Mean	AllMean	log2FoldChange	pvalue	qvalue	State	gene_id	XF_1_count	XF_1_FPKM	XF_1_TPM	XF_1_CPM	XF_2_count	XF_2_FPKM	XF_2_TPM	XF_2_CPM	XF_3_count	XF_3_FPKM	XF_3_TPM	XF_3_CPM	Symbol	Description	KEGG_A_class	KEGG_B_class	Pathway	K_ID	GO Component	GO Function	GO Process
TFF2	0.33853382	-1.83891	-0.75019	-2.6181	0.000262903	0.019673936	Down	ENSG00000160181	39	1.681913936	6.350422965	1.175657842	4	0.190651298	0.765679453	0.133265257	7	0.339651163	1.366382688	0.237416163	TFF2	trefoil factor 2 [Source:HGNC Symbol;Acc:HGNC:11756]	-	-	-	-	GO:0005575//cellular_component;GO:0005615//extracellular space;GO:0005576//extracellular region;GO:0044421//extracellular region part	GO:0003674//molecular_function	GO:0065007//biological regulation;GO:0044699//single-organism process;GO:0050896//response to stimulus;GO:0009987//cellular process;GO:0008150//biological_process;GO:0023052//signaling;GO:0065008//regulation of biological quality;GO:0042592//homeostatic process;GO:0050789//regulation of biological process;GO:0051716//cellular response to stimulus;GO:0007165//signal transduction;GO:0050794//regulation of cellular process;GO:0051234//establishment of localization;GO:0051179//localization;GO:0044763//single-organism cellular process;GO:0006810//transport;GO:0007154//cell communication;GO:0044700//single organism signaling
FAM111B	4.355838511	5.551402169	4.95362034	1.200157767	0.000264162	0.019681777	Up	ENSG00000189057	681	5.311460445	20.0545459587	20.5287946 19	1339	11.5421849 37	46.3548581 01	44.61054478	1421	12.4697234 52	50.1644512 88	48.1954811 43	FAM111B	family with sequence similarity 111 member B [Source:HGNC Symbol;Acc:HGNC:24200]	-	-	-	-	-	GO:0003674//molecular_function	-

id	CON_Mean	KD_Mean	AllMean	log2FoldChange	pvalue	qvalue	State	gene_id	XF_1_count	XF_1_FPKM	XF_1_TPM	XF_1_CPM	XF_2_count	XF_2_FPKM	XF_2_TPM	XF_2_CPM	XF_3_count	XF_3_FPKM	XF_3_TPM	XF_3_CPM	Symbol	Description	KEGG_A_class	KEGG_B_class	Pathway	K_ID	GO Component	GO Function	GO Process
PHRF1	5.732069812	6.823165757	6.277617784	1.111176114	0.000274852	0.020300973	Up	ENSG00000070047	1773	9.238930715	34.8835433389	53.447214184	2852	16.424914133	65.964509144	95.018128239	3899	22.859257206	91.960507305	132.240802939	PHRF1	PHD and ring finger domains 1 [Source:HGNC Symbol;Acc:HGNC:24351]	-	-	-	-	GO:0005575//cellular_component	GO:0019899//enzyme binding;GO:0005515//protein binding;GO:0043167//ion binding;GO:0005488//binding;GO:0003674//molecular_function	GO:0006139//nucleobase-containing compound metabolic process;GO:0008152//metabolic process;GO:0016070//RNA metabolic process;GO:0046483//heterocycle metabolic process;GO:0071704//organic substance metabolic process;GO:0006397//mRNA processing;GO:0010467//gene expression;GO:0034641//cellular nitrogen compound metabolic process;GO:1901360//organic cyclic compound metabolic process;GO:0090304//nucleic acid metabolic process;GO:0016071//mRNA metabolic process;GO:0006725//cellular aromatic compound metabolic process;GO:0044237//cellular metabolic process;GO:0009058//biosynthetic process;GO:0044238//primary metabolic process;GO:0044260//cellular macromolecule metabolic process;GO:0043170//macromolecule metabolic process;GO:0006396//RNA processing;GO:0006807//nitrogen compound metabolic process;GO:0008150//biological_process;GO:0009987//cellular process

id	CON_Mean	KD_Mean	AllMean	log2FoldChange	pvalue	qvalue	State	gene_id	XF_1_count	XF_1_FPKM	XF_1_TPM	XF_1_CPM	XF_2_count	XF_2_FPKM	XF_2_TPM	XF_2_CPM	XF_3_count	XF_3_FPKM	XF_3_TPM	XF_3_CPM	Symbol	Description	KEGG_A_class	KEGG_B_class	Pathway	K_ID	GO Component	GO Function	GO Process
HYOU1	8.126228858	9.133032989	8.629630923	1.008742208	0.000280787	0.020649964	Up	ENSG00000149428	9334	36.433265582	137.561525247	281.374110091	15846	68.358191842	274.535046899	527.930315597	17277	75.874272632	305.234616313	585.977007533	HYOU1	hypoxia up-regulated 1 [Source:HGNC Symbol;Acc:HGNC:16931]	Genetic Information Processing	Folding, sorting and degradation	ko04141//Protein processing in endoplasmic reticulum	K09486	GO:0043231//intracellular membrane-bounded organelle;GO:0032991//macromolecular complex;GO:0005737//cytoplasm;GO:0044464//cell part;GO:0005576//extracellular region;GO:0043227//membrane-bounded organelle;GO:0097708//intracellular vesicle;GO:0005575//cellular_component;GO:0031410//cytoplasmic vesicle;GO:0012505//endomembrane system;GO:0005615//extracellular space;GO:0005622//intracellular;GO:0044424//intracellular part;GO:0044421//extracellular region part;GO:0005783//endoplasmic reticulum;GO:0043229//intracellular organelle;GO:0005623//cell;GO:0044444//cytoplasmic part;GO:0043226//organelle;GO:0031982//vesicle	GO:0005488//binding;GO:0043167//ion binding;GO:0003674//molecular_function	GO:0051716//cellular response to stimulus;GO:0044699//single-organism process;GO:0050789//regulation of biological process;GO:0050794//regulation of cellular process;GO:0007154//cell communication;GO:0044763//single-organism cellular process;GO:0051179//localization;GO:0007165//signal transduction;GO:0044700//single organism signaling;GO:0008219//cell death;GO:0006810//transport;GO:0023052//signaling;GO:0008150//biological_process;GO:0065007//biological regulation;GO:0050896//response to stimulus;GO:0006950//response to stress;GO:0009987//cellular process;GO:0016192//vesicle-mediated transport;GO:0051234//establishment of localization
CTSW	-0.46228	1.994461465	0.766091704	2.652830378	0.000287489	0.021052096	Up	ENSG00000172543	21	0.349942803	1.321283309	0.63304653	93	1.712779008	6.878734685	3.098417225	141	2.643582012	10.634866249	4.78223986	CTSW	cathepsin W [Source:HGNC Symbol;Acc:HGNC:2546]	Cellular Processes	Transport and catabolism	ko04142//Lysosome	K08569	GO:0043229//intracellular organelle;GO:0000323//lytic vacuole;GO:0044421//extracellular region part;GO:0012505//endomembrane system;GO:0005576//extracellular region;GO:0005737//cytoplasm;GO:0043226//organelle;GO:0044444//cytoplasmic part;GO:0043231//intracellular membrane-bounded organelle;GO:0005575//cellular_component;GO:0005615//extracellular space;GO:0097708//intracellular vesicle;GO:0043227//membrane-bounded organelle;GO:0031982//vesicle;GO:0031410//cytoplasmic vesicle;GO:0044464//cell part;GO:0005622//intracellular;GO:0044424//intracellular part;GO:0005764//lysosome;GO:0005773//vacuole;GO:0005623//cell;GO:0005783//endoplasmic reticulum	GO:0008233//peptidase activity;GO:0016787//hydrolase activity;GO:0003824//catalytic activity;GO:0003674//molecular_function	GO:0008152//metabolic process;GO:0008150//biological_process;GO:0006810//transport;GO:0002376//immune system process;GO:0051234//establishment of localization;GO:0009056//catabolic process;GO:0051179//localization;GO:0016192//vesicle-mediated transport

id	CON_Mean	KD_Mean	AllMean	log2FoldChange	pvalue	qvalue	State	gene_id	XF_1_count	XF_1_FPKM	XF_1_TPM	XF_1_CPM	XF_2_count	XF_2_FPKM	XF_2_TPM	XF_2_CPM	XF_3_count	XF_3_FPKM	XF_3_TPM	XF_3_CPM	Symbol	Description	KEGG_A_class	KEGG_B_class	Pathway	K_ID	GO Component	GO Function	GO Process
CYB5R2	3.789994483	5.331781453	4.560887968	1.632728546	0.000300639	0.021761648	Up	ENSG00000166394	459	1.609841587	6.078298517	13.836588444	842	3.263797161	13.107817549	28.052336598	1665	6.570230397	26.431380292	56.471130262	CYB5R2	cytochrome b5 reductase 2 [Source:HGNC Symbol;Acc:HGNC:24376]	Metabolism	Carbohydrate metabolism	ko00520//Amino sugar and nucleotide sugar metabolism	K00326	GO:0005623//cell;GO:0005622//intracellular;GO:0043231//intracellular membrane-bounded organelle;GO:0043226//organelle;GO:0044464//cell part;GO:0005634//nucleus;GO:0005575//cellular_component;GO:0043227//membrane-bounded organelle;GO:0043229//intracellular organelle;GO:0044424//intracellular part	GO:0043167//ion binding;GO:0005488//binding;GO:0003674//molecular_function;GO:0016491//oxidoreductase activity;GO:0003824//catalytic activity	GO:0044710//single-organism metabolic process;GO:0009058//biosynthetic process;GO:0044699//single-organism process;GO:0008150//biological_process;GO:0071704//organic substance metabolic process;GO:0006629//lipid metabolic process;GO:0044238//primary metabolic process;GO:0006810//transport;GO:0051234//establishment of localization;GO:0051179//localization;GO:0008152//metabolic process
CACNG6	1.906183036	-0.28193	0.812125709	-2.24987	0.000301949	0.021761648	Down	ENSG00000130433	122	1.470491359	5.552152161	3.677698889	15	0.199817958	0.802493909	0.499744714	30	0.406836402	1.63666219	1.017497843	CACNG6	calcium voltage-gated channel auxiliary subunit gamma 6 [Source:HGNC Symbol;Acc:HGNC:13625]	Organismal Systems;Environmental Information Processing;Human Diseases	Signal transduction;Circulatory system;Endocrine system;Cardiovascular diseases	ko04010//MAPK signaling pathway;ko04921//Oxytocin signaling pathway;ko04261//Adrenergic signaling in cardiomyocytes;ko05414//Dilated cardiomyopathy (DCM);ko05410//Hypertrophic card	K04871	GO:0005575//cellular_component;GO:0071944//cell periphery;GO:0032991//macromolecular complex;GO:0005623//cell;GO:0005886//plasma membrane;GO:0044464//cell part;GO:0016020//membrane	GO:0003674//molecular_function;GO:0022857//transmembrane transporter activity;GO:0005215//transporter activity	GO:0006810//transport;GO:0032501//multicellular organismal process;GO:0003008//system process;GO:0051179//localization;GO:0003013//circulatory system process;GO:0008150//biological_process;GO:0051234//establishment of localization;GO:0055085//transmembrane transport

id	CON_Mean	KD_Mean	AllMean	log2FoldChange	pvalue	qvalue	State	gene_id	XF_1_count	XF_1_FPKM	XF_1_TPM	XF_1_CPM	XF_2_count	XF_2_FPKM	XF_2_TPM	XF_2_CPM	XF_3_count	XF_3_FPKM	XF_3_TPM	XF_3_CPM	Symbol	Description	KEGG_A_class	KEGG_B_class	Pathway	K_ID	GO Component	GO Function	GO Process
SLC1A4	5.375464356	6.505620944	5.94054265	1.159893522	0.000302281	0.021761648	Up	ENSG00000115902	1384	6.010773785	22.694951895	41.720780841	3277	15.729370666	63.171119616	109.177561796	2184	10.671926659	42.932094451	74.073842939	SLC1A4	solute carrier family 1 member 4 [Source:HGNC Symbol;Acc:HGNC:10942]	-	-	-	-	GO:0044444//cytoplasmic part;GO:0005575//cellular_component;GO:0044464//cell part;GO:0005623//cell;GO:0005815//microtubule organizing center;GO:0005615//extracellular space;GO:0071944//cell periphery;GO:0043227//membrane-bounded organelle;GO:0044422//organelle part;GO:0097708//intracellular vesicle;GO:0043228//non-membrane-bounded organelle;GO:0005856//cytoskeleton;GO:0015630//microtubule cytoskeleton;GO:0043232//intracellular non-membrane-bounded organelle;GO:0044421//extracellular region part;GO:0043229//intracellular organelle;GO:0005737//cytoplasm;GO:0031410//cytoplasmic vesicle;GO:0044446//intracellular organelle part;GO:0005576//extracellular region;GO:0005886//plasma membrane;GO:0044424//intracellular part;GO:0043226//organelle;GO:0016020//membrane;GO:0044430//cytoskeletal part;GO:0005622//intracellular;GO:0031982//vesicle	GO:0022857//transmembrane transporter activity;GO:0005215//transporter activity;GO:0003674//molecular_function	GO:0044700//single organism signaling;GO:0051234//establishment of localization;GO:0003008//system process;GO:0050877//neurological system process;GO:0006810//transport;GO:0023052//signaling;GO:0044763//single-organism cellular process;GO:0007154//cell communication;GO:0055085//transmembrane transport;GO:0007267//cell-cell signaling;GO:0009987//cellular process;GO:0032501//multicellular organismal process;GO:0008150//biological_process;GO:0044699//single-organism process;GO:0051179//localization

id	CON_Mean	KD_Mean	AllMean	log2FoldChange	pvalue	qvalue	State	gene_id	XF_1_count	XF_1_FPKM	XF_1_TPM	XF_1_CPM	XF_2_count	XF_2_FPKM	XF_2_TPM	XF_2_CPM	XF_3_count	XF_3_FPKM	XF_3_TPM	XF_3_CPM	Symbol	Description	KEGG_A_class	KEGG_B_class	Pathway	K_ID	GO Component	GO Function	GO Process
CCND1	8.447623607	9.496086792	8.9718552	1.059950617	0.000304333	0.021817358	Up	ENSG00000110092	11664	73.85257926	278.846084318	351.612129859	24249	169.688574719	681.490536293	807.887304235	18677	133.051930306	535.254619097	633.460240186	CCND1	cyclin D1 [Source:HGNC Symbol;Acc:HGNC:1582]	Human Diseases;Environmental Information Processing;Cellular Processes;Organismal Systems	Signal transduction;Infectious diseases;Cellular commiunity;Cardiovascular diseases;Cancers;Cell growth and death;Endocrine system	ko05200//Pathways in cancer;ko05166//HTLV-I infection;ko04151//PI3K-Akt signaling pathway;ko05203//Viral carcinogenesis;ko04510//Focal adhesion;ko05205//Proteoglycans in cancer;ko05[illegible]	K04503	GO:0043226//organelle;GO:0044464//cell part;GO:0005622//intracellular;GO:0005635//nuclear envelope;GO:0031975//envelope;GO:0031974//membrane-enclosed lumen;GO:0031981//nuclear lumen;GO:0005634//nucleus;GO:0044444//cytoplasmic part;GO:0044446//intracellular organelle part;GO:0043231//intracellular membrane-bounded organelle;GO:0005829//cytosol;GO:0005623//cell;GO:0005575//cellular_component;GO:0043229//intracellular organelle;GO:0043227//membrane-bounded organelle;GO:0005737//cytoplasm;GO:0012505//endomembrane system;GO:0031967//organelle envelope;GO:0005654//nucleoplasm;GO:0043233//organelle lumen;GO:0044422//organelle part;GO:0044428//nuclear part;GO:0032991//macromolecular complex;GO:0070013//intracellular organelle lumen;GO:0044424//intracellular part	GO:0016772//transferase activity, transferring phosphorus-containing groups;GO:0016740//transferase activity;GO:0030234//enzyme regulator activity;GO:0016301//kinase activity;GO:0019899//enzyme binding;GO:0003824//catalytic activity;GO:0098772//molecular function regulator;GO:0005488//binding;GO:0005515//protein binding;GO:0003674//molecular_function;GO:0008134//transcription factor binding	GO:0050896//response to stimulus;GO:0048856//anatomical structure development;GO:0044267//cellular protein metabolic process;GO:0007049//cell cycle;GO:0006807//nitrogen compound metabolic process;GO:0044260//cellular macromolecule metabolic process;GO:0000003//reproduction;GO:0044238//primary metabolic process;GO:0000278//mitotic cell cycle;GO:0051716//cellular response to stimulus;GO:0051301//cell division;GO:0051179//localization;GO:0008152//metabolic process;GO:0034641//cellular nitrogen compound metabolic process;GO:0044699//single-organism process;GO:0044767//single-organism developmental process;GO:0051234//establishment of localization;GO:0048869//cellular developmental process;GO:0006464//cellular protein modification process;GO:0043412//macromolecule modification;GO:0044237//cellular metabolic process;GO:0043170//macromolecule metabolic process;GO:0007267//cell-cell signaling;GO:0065007//biological regulation;GO:0009987//cellular process;GO:0008150//biolo[illegible]

id	CON_Mean	KD_Mean	AllMean	log2FoldChange	pvalue	qvalue	State	gene_id	XF_1_count	XF_1_FPKM	XF_1_TPM	XF_1_CPM	XF_2_count	XF_2_FPKM	XF_2_TPM	XF_2_CPM	XF_3_count	XF_3_FPKM	XF_3_TPM	XF_3_CPM	Symbol	Description	KEGG_A_class	KEGG_B_class	Pathway	K_ID	GO Component	GO Function	GO Process
NFRKB	5.776063773	6.77834274	6.277203256	1.006323519	0.000315809	0.022545341	Up	ENSG00000170322	1828	8.69853089	32.843149182	55.105193191	3459	18.191180898	73.058057365	115.241130989	3021	16.173959392	65.066222291	102.462032746	NFRKB	nuclear factor related to kappaB binding protein [Source:HGNC Symbol;Acc:HGNC:7802]	-	-	-	-	GO:0005694//chromosome;GO:0043226//organelle;GO:0043228//non-membrane-bounded organelle;GO:0043231//intracellular membrane-bounded organelle;GO:0031981//nuclear lumen;GO:0031974//membrane-enclosed lumen;GO:0044428//nuclear part;GO:0005623//cell;GO:0044424//intracellular part;GO:0043232//intracellular non-membrane-bounded organelle;GO:0000228//nuclear chromosome;GO:0044422//organelle part;GO:0005634//nucleus;GO:0005622//intracellular;GO:0005654//nucleoplasm;GO:0043227//membrane-bounded organelle;GO:0043229//intracellular organelle;GO:0032991//macromolecular complex;GO:0005575//cellular_component;GO:0044446//intracellular organelle part;GO:0070013//intracellular organelle lumen;GO:0043233//organelle lumen;GO:0044464//cell part	GO:0003674//molecular_function;GO:0019899//enzyme binding;GO:1901363//heterocyclic compound binding;GO:0005515//protein binding;GO:0003677//DNA binding;GO:0003676//nucleic acid binding;GO:0005488//binding;GO:0097159//organic cyclic compound binding	GO:0006464//cellular protein modification process;GO:0044238//primary metabolic process;GO:0006139//nucleobase-containing compound metabolic process;GO:0044267//cellular protein metabolic process;GO:1901360//organic cyclic compound metabolic process;GO:0008150//biological_process;GO:0009058//biosynthetic process;GO:0008152//metabolic process;GO:0090304//nucleic acid metabolic process;GO:0044237//cellular metabolic process;GO:0043412//macromolecule modification;GO:0009987//cellular process;GO:0044260//cellular macromolecule metabolic process;GO:0006725//cellular aromatic compound metabolic process;GO:0046483//heterocycle metabolic process;GO:0043170//macromolecule metabolic process;GO:0006259//DNA metabolic process;GO:0071704//organic substance metabolic process;GO:0034641//cellular nitrogen compound metabolic process;GO:0019538//protein metabolic process;GO:0006807//nitrogen compound metabolic process;GO:0006950//response to [illegible]

id	CON_Mean	KD_Mean	AllMean	log2FoldChange	pvalue	qvalue	State	gene_id	XF_1_count	XF_1_FPKM	XF_1_TPM	XF_1_CPM	XF_2_count	XF_2_FPKM	XF_2_TPM	XF_2_CPM	XF_3_count	XF_3_FPKM	XF_3_TPM	XF_3_CPM	Symbol	Description	KEGG_A_class	KEGG_B_class	Pathway	K_ID	GO Component	GO Function	GO Process
SKAP2	5.749828706	4.603247665	5.176538185	-1.13982	0.000317933	0.022602382	Down	ENSG00000005020	1795	10.093341874	38.109554026	54.110405787	638	3.964896193	15.923518937	21.255808491	798	5.048580976	20.309936737	27.065442612	SKAP2	src kinase associated phosphoprotein 2 [Source:HGNC Symbol;Acc:HGNC:15687]	-	-	-	-	GO:0044422//organelle part;GO:0005886//plasma membrane;GO:0044464//cell part;GO:0031981//nuclear lumen;GO:0031974//membrane-enclosed lumen;GO:0044428//nuclear part;GO:0043233//organelle lumen;GO:0005829//cytosol;GO:0005654//nucleoplasm;GO:0005623//cell;GO:0043231//intracellular membrane-bounded organelle;GO:0005622//intracellular;GO:0044424//intracellular part;GO:0070013//intracellular organelle lumen;GO:0044446//intracellular organelle part;GO:0016020//membrane;GO:0044444//cytoplasmic part;GO:0043229//intracellular organelle;GO:0005634//nucleus;GO:0071944//cell periphery;GO:0005575//cellular_component;GO:0005737//cytoplasm;GO:0043226//organelle;GO:0043227//membrane-bounded organelle	GO:0060090//binding, bridging;GO:0003674//molecular_function;GO:0005488//binding;GO:0030674//protein binding, bridging;GO:0005515//protein binding	GO:0044763//single-organism cellular process;GO:0071840//cellular component organization or biogenesis;GO:0044700//single organism signaling;GO:0050896//response to stimulus;GO:0009987//cellular process;GO:0002376//immune system process;GO:0007165//signal transduction;GO:0051716//cellular response to stimulus;GO:0016043//cellular component organization;GO:0044699//single-organism process;GO:0022607//cellular component assembly;GO:0050794//regulation of cellular process;GO:0008150//biological_process;GO:0043933//macromolecular complex subunit organization;GO:0023052//signaling;GO:0065007//biological regulation;GO:0065003//macromolecular complex assembly;GO:0008283//cell proliferation;GO:0050789//regulation of biological process;GO:0007154//cell communication;GO:0044085//cellular component biogenesis

id	CON_Mean	KD_Mean	AllMean	log2FoldChange	pvalue	qvalue	State	gene_id	XF_1_count	XF_1_FPKM	XF_1_TPM	XF_1_CPM	XF_2_count	XF_2_FPKM	XF_2_TPM	XF_2_CPM	XF_3_count	XF_3_FPKM	XF_3_TPM	XF_3_CPM	Symbol	Description	KEGG_A_class	KEGG_B_class	Pathway	K_ID	GO Component	GO Function	GO Process
MYBBP1A	7.40537138	8.404198848	7.904785114	1.003781559	0.000324596	0.022855719	Up	ENSG00000132382	5662	28.6474324451	108.164460127	170.681402543	10866	60.761173319	244.024470473	362.015070635	9171	52.206963825	210.023398175	311.049090472	MYBBP1A	MYB binding protein 1a [Source:HGNC Symbol;Acc:HGNC:7546]	-	-	-	-	GO:0044428//nuclear part;GO:0005634//nucleus;GO:0043232//intracellular non-membrane-bounded organelle;GO:0043228//non-membrane-bounded organelle;GO:0005622//intracellular;GO:0031974//membrane-enclosed lumen;GO:0070013//intracellular organelle lumen;GO:0043229//intracellular organelle;GO:0005730//nucleolus;GO:0032991//macromolecular complex;GO:0005575//cellular_component;GO:0031981//nuclear lumen;GO:0044446//intracellular organelle part;GO:0043233//organelle lumen;GO:0044422//organelle part;GO:0043231//intracellular membrane-bounded organelle;GO:0005737//cytoplasm;GO:0044464//cell part;GO:0005654//nucleoplasm;GO:0043227//membrane-bounded organelle;GO:0044424//intracellular part;GO:0043226//organelle;GO:0005623//cell	GO:0008134//transcription factor binding;GO:0097159//organic cyclic compound binding;GO:0003677//DNA binding;GO:0003723//RNA binding;GO:0003676//nucleic acid binding;GO:0003674//molecular_function;GO:0005488//binding;GO:1901363//heterocyclic compound binding;GO:0005515//protein binding	GO:0065007//biological regulation;GO:0050794//regulation of cellular process;GO:0007154//cell communication;GO:0044700//single organism signaling;GO:0008152//metabolic process;GO:0007165//signal transduction;GO:0044237//cellular metabolic process;GO:0009058//biosynthetic process;GO:0050789//regulation of biological process;GO:0008219//cell death;GO:0023052//signaling;GO:0034641//cellular nitrogen compound metabolic process;GO:0044085//cellular component biogenesis;GO:0042254//ribosome biogenesis;GO:0022613//ribonucleoprotein complex biogenesis;GO:0009987//cellular process;GO:0006807//nitrogen compound metabolic process;GO:0048869//cellular developmental process;GO:0007049//cell cycle;GO:0006950//response to stress;GO:0071840//cellular component organization or biogenesis;GO:0051716//cellular response to stimulus;GO:0008150//biological_process;GO:0050896//response to stimulus;GO:0030154//cell differentiation;GO:0044763//single-organism cellular process;GO:0044699//singl[illegible]

id	CON_Mean	KD_Mean	AllMean	log2FoldChange	pvalue	qvalue	State	gene_id	XF_1_count	XF_1_FPKM	XF_1_TPM	XF_1_CPM	XF_2_count	XF_2_FPKM	XF_2_TPM	XF_2_CPM	XF_3_count	XF_3_FPKM	XF_3_TPM	XF_3_CPM	Symbol	Description	KEGG_A_class	KEGG_B_class	Pathway	K_ID	GO Component	GO Function	GO Process
SAFB2	6.080354601	7.104573617	6.592464109	1.032935889	0.000325515	0.022855719	Up	ENSG00000130254	2258	11.310663762	42.705811127	68.067574522	4519	25.017684296	100.474148692	150.556424093	3636	20.491980478	82.437189602	123.320738519	SAFB2	scaffold attachment factor B2 [Source:HGNC Symbol;Acc:HGNC:21605]	-	-	-	-	GO:0031974//membrane-enclosed lumen;GO:0005654//nucleoplasm;GO:0070013//intracellular organelle lumen;GO:0043227//membrane-bounded organelle;GO:0005737//cytoplasm;GO:0044424//intracellular part;GO:0044428//nuclear part;GO:0044446//intracellular organelle part;GO:0044421//extracellular region part;GO:0005576//extracellular region;GO:0005575//cellular_component;GO:0043226//organelle;GO:0031981//nuclear lumen;GO:0043233//organelle lumen;GO:0043229//intracellular organelle;GO:0044464//cell part;GO:0044422//organelle part;GO:0005615//extracellular space;GO:0005623//cell;GO:0005634//nucleus;GO:0005622//intracellular;GO:0043231//intracellular membrane-bounded organelle	GO:0005488//binding;GO:0003723//RNA binding;GO:0097159//organic cyclic compound binding;GO:0003677//DNA binding;GO:1901363//heterocyclic compound binding;GO:0003676//nucleic acid binding;GO:0003674//molecular_function	GO:0090304//nucleic acid metabolic process;GO:0006396//RNA processing;GO:0046483//heterocycle metabolic process;GO:0043170//macromolecule metabolic process;GO:0008152//metabolic process;GO:0044237//cellular metabolic process;GO:0044260//cellular macromolecule metabolic process;GO:0009058//biosynthetic process;GO:0016071//mRNA metabolic process;GO:0050789//regulation of biological process;GO:0006807//nitrogen compound metabolic process;GO:0044763//single-organism cellular process;GO:0048869//cellular developmental process;GO:0044700//single organism signaling;GO:0008150//biological_process;GO:0007154//cell communication;GO:0007165//signal transduction;GO:0030154//cell differentiation;GO:1901360//organic cyclic compound metabolic process;GO:0044238//primary metabolic process;GO:0048856//anatomical structure development;GO:0009987//cellular process;GO:0006139//nucleobase-containing compound metabolic process;GO:0006725//cellu[illegible]

id	CON_Mean	KD_Mean	AllMean	log2FoldChange	pvalue	qvalue	State	gene_id	XF_1_count	XF_1_FPKM	XF_1_TPM	XF_1_CPM	XF_2_count	XF_2_FPKM	XF_2_TPM	XF_2_CPM	XF_3_count	XF_3_FPKM	XF_3_TPM	XF_3_CPM	Symbol	Description	KEGG_A_class	KEGG_B_class	Pathway	K_ID	GO Component	GO Function	GO Process
MEPCE	6.323985356	7.410486239	6.867235798	1.110387473	0.000337491	0.023392412	Up	ENSG00000146834	2674	25.095867009	94.754771168	80.607924832	6026	62.504392798	251.025457897	200.764109667	4168	44.011322206	177.053150972	141.364366927	MEPCE	methylphosphate capping enzyme [Source:HGNC Symbol;Acc:HGNC:20247]	-	-	-	-	-	GO:0005488//binding;GO:0008168//methyltransferase activity;GO:0003824//catalytic activity;GO:0097159//organic cyclic compound binding;GO:0003676//nucleic acid binding;GO:1901363//heterocyclic compound binding;GO:0016741//transferase activity, transferring one-carbon groups;GO:0003723//RNA binding;GO:0003674//molecular_function;GO:0016740//transferase activity	GO:0008152//metabolic process;GO:0006807//nitrogen compound metabolic process;GO:0044237//cellular metabolic process;GO:0008150//biological_process;GO:0009058//biosynthetic process;GO:0034641//cellular nitrogen compound metabolic process;GO:0009987//cellular process;GO:0000278//mitotic cell cycle;GO:0007049//cell cycle;GO:0044763//single-organism cellular process;GO:0044699//single-organism process
MAMDC4	1.786145284	3.807885312	2.797015298	2.081736861	0.000338643	0.023392412	Up	ENSG00000177943	112	0.61498145	2.321992962	3.376248161	506	3.070683973	12.332250836	16.85805501	332	2.051058189	8.251202122	11.260309458	MAMDC4	MAM domain containing 4 [Source:HGNC Symbol;Acc:HGNC:24083]	-	-	-	-	GO:0005575//cellular_component	GO:0003674//molecular_function	GO:0033036//macromolecule localization;GO:0015031//protein transport;GO:0051179//localization;GO:0008150//biological_process;GO:0071702//organic substance transport;GO:0008104//protein localization;GO:0051234//establishment of localization;GO:0006810//transport;GO:0045184//establishment of protein localization

id	CON_Mean	KD_Mean	AllMean	log2FoldChange	pvalue	qvalue	State	gene_id	XF_1_count	XF_1_FPKM	XF_1_TPM	XF_1_CPM	XF_2_count	XF_2_FPKM	XF_2_TPM	XF_2_CPM	XF_3_count	XF_3_FPKM	XF_3_TPM	XF_3_CPM	Symbol	Description	KEGG_A_class	KEGG_B_class	Pathway	K_ID	GO Component	GO Function	GO Process
DDX11	5.89545923	6.936742279	6.416100755	1.054754014	0.000344388	0.023693325	Up	ENSG00000013573	1986	7.155266488	27.016226953	59.868114703	4134	16.461054513	66.109653442	137.729643107	3149	12.7648329	51.351647105	106.803356875	DDX11	DEAD/H-box helicase 11 [Source:HGNC Symbol;Acc:HGNC:2736]	-	-	-	-	GO:0032991//macromolecular complex;GO:0005576//extracellular region;GO:0044446//intracellular organelle part;GO:0044430//cytoskeletal part;GO:0005623//cell;GO:0005575//cellular_component;GO:0043226//organelle;GO:0005730//nucleolus;GO:0070013//intracellular organelle lumen;GO:0005634//nucleus;GO:0044421//extracellular region part;GO:0043232//intracellular non-membrane-bounded organelle;GO:0005815//microtubule organizing center;GO:0031981//nuclear lumen;GO:0015630//microtubule cytoskeleton;GO:0044428//nuclear part;GO:0044424//intracellular part;GO:0005615//extracellular space;GO:0000228//nuclear chromosome;GO:0005856//cytoskeleton;GO:0044422//organelle part;GO:0043229//intracellular organelle;GO:0044464//cell part;GO:0043231//intracellular membrane-bounded organelle;GO:0005654//nucleoplasm;GO:0043233//organelle lumen;GO:0005694//chromosome;GO:0043227//membrane-bounded organelle;GO:0043228//non-membrane-bounded organelle;GO:0005622//intracellular;GO:0031974//membrane-enclosed lumen;GO:0005737//cytoplasm	GO:0016887//ATPase activity;GO:0016817//hydrolase activity, acting on acid anhydrides;GO:0003674//molecular_function;GO:0043167//ion binding;GO:0016818//hydrolase activity, acting on acid anhydrides, in phosphorus-containing anhydrides;GO:0097159//organic cyclic compound binding;GO:0016787//hydrolase activity;GO:0016462//pyrophosphatase activity;GO:0004386//helicase activity;GO:0005488//binding;GO:1901363//heterocyclic compound binding;GO:0003677//DNA binding;GO:0003824//catalytic activity;GO:0003676//nucleic acid binding;GO:0003723//RNA binding;GO:0017111//nucleoside-triphosphatase activity	GO:0009987//cellular process;GO:0044699//single-organism process;GO:0044403//symbiosis, encompassing mutualism through parasitism;GO:0051716//cellular response to stimulus;GO:0044419//interspecies interaction between organisms;GO:0034641//cellular nitrogen compound metabolic process;GO:0044238//primary metabolic process;GO:0016043//cellular component organization;GO:0044237//cellular metabolic process;GO:0050789//regulation of biological process;GO:0009058//biosynthetic process;GO:0051276//chromosome organization;GO:0007059//chromosome segregation;GO:0071704//organic substance metabolic process;GO:0006950//response to stress;GO:0071840//cellular component organization or biogenesis;GO:0050794//regulation of cellular process;GO:0044700//single organism signaling;GO:1901360//organic cyclic compound metabolic process;GO:0006996//organelle organization;GO:0007165//signal transduction;GO:0065007//[illegible]

id	CON_Mean	KD_Mean	AllMean	log2FoldChange	pvalue	qvalue	State	gene_id	XF_1_count	XF_1_FPKM	XF_1_TPM	XF_1_CPM	XF_2_count	XF_2_FPKM	XF_2_TPM	XF_2_CPM	XF_3_count	XF_3_FPKM	XF_3_TPM	XF_3_CPM	Symbol	Description	KEGG_A_class	KEGG_B_class	Pathway	K_ID	GO Component	GO Function	GO Process
CEP164	6.025078549	7.063566237	6.544322393	1.051725133	0.000359713	0.024648291	Up	ENSG00000110274	2173	5.079500878	19.178733423	65.505243328	4511	11.653992988	46.803893219	150.289893579	3441	9.049860619	36.406684874	116.707002542	CEP164	centrosomal protein 164 [Source:HGNC Symbol;Acc:HGNC:29182]	-	-	-	-	GO:0043227//membrane-bounded organelle;GO:0005815//microtubule organizing center;GO:0043229//intracellular organelle;GO:0005654//nucleoplasm;GO:0043228//non-membrane-bounded organelle;GO:0044422//organelle part;GO:0043226//organelle;GO:0005575//cellular_component;GO:0044430//cytoskeletal part;GO:0044421//extracellular region part;GO:0044424//intracellular part;GO:0070013//intracellular organelle lumen;GO:0005576//extracellular region;GO:0043233//organelle lumen;GO:0005615//extracellular space;GO:0044464//cell part;GO:0032991//macromolecular complex;GO:0042995//cell projection;GO:0005634//nucleus;GO:0043232//intracellular non-membrane-bounded organelle;GO:0031981//nuclear lumen;GO:0005623//cell;GO:0044428//nuclear part;GO:0043231//intracellular membrane-bounded organelle;GO:0005737//cytoplasm;GO:0044446//intracellular organelle part;GO:0031974//membrane-enclosed lumen;GO:0005856//cytoskeleton;GO:0005929//cilium;GO:0005829//cytosol;GO:0044444//cytoplasmic part;GO:0005622//intracellular;GO:0015630//microtubule cytoskeleton	GO:0003674//molecular_function	GO:0006139//nucleobase-containing compound metabolic process;GO:1901360//organic cyclic compound metabolic process;GO:0044237//cellular metabolic process;GO:0071704//organic substance metabolic process;GO:0006950//response to stress;GO:0044763//single-organism cellular process;GO:0051301//cell division;GO:0044085//cellular component biogenesis;GO:0006259//DNA metabolic process;GO:0034641//cellular nitrogen compound metabolic process;GO:0044699//single-organism process;GO:0046483//heterocycle metabolic process;GO:0071840//cellular component organization or biogenesis;GO:0090304//nucleic acid metabolic process;GO:0022607//cellular component assembly;GO:0044238//primary metabolic process;GO:0006725//cellular aromatic compound metabolic process;GO:0008150//biological_process;GO:0044260//cellular macromolecule metabolic process;GO:0016043//cellular component organization;GO:0008152//metabolic process;GO:0043170//macromolecule metabolic [illegible]

id	CON_Mean	KD_Mean	AllMean	log2FoldChange	pvalue	qvalue	State	gene_id	XF_1_count	XF_1_FPKM	XF_1_TPM	XF_1_CPM	XF_2_count	XF_2_FPKM	XF_2_TPM	XF_2_CPM	XF_3_count	XF_3_FPKM	XF_3_TPM	XF_3_CPM	Symbol	Description	KEGG_A_class	KEGG_B_class	Pathway	K_ID	GO Component	GO Function	GO Process
IGFBP7	5.780782949	4.705085231	5.24293409	-1.07822	0.000367242	0.025063502	Down	ENSG00000163453	1834	28.645628823	108.157650147	55.286063628	757	13.067590615	52.481078063	25.220449887	775	13.619357996	54.789316171	26.285360933	IGFBP7	insulin like growth factor binding protein 7 [Source:HGNC Symbol;Acc:HGNC:5476]	-	-	-	-	GO:0044444//cytoplasmic part;GO:0044424//intracellular part;GO:0043231//intracellular membrane-bounded organelle;GO:0005615//extracellular space;GO:0012505//endomembrane system;GO:0044464//cell part;GO:0005576//extracellular region;GO:0005737//cytoplasm;GO:0005623//cell;GO:0043226//organelle;GO:0031012//extracellular matrix;GO:0043229//intracellular organelle;GO:0005575//cellular_component;GO:0044421//extracellular region part;GO:0043227//membrane-bounded organelle;GO:0005783//endoplasmic reticulum;GO:0005622//intracellular	GO:0003674//molecular_function;GO:0005198//structural molecule activity	GO:0006464//cellular protein modification process;GO:0000003//reproduction;GO:0019538//protein metabolic process;GO:0043170//macromolecule metabolic process;GO:0008152//metabolic process;GO:0050896//response to stimulus;GO:0032502//developmental process;GO:0036211//protein modification process;GO:0040007//growth;GO:0008150//biological_process;GO:0009058//biosynthetic process;GO:0007155//cell adhesion;GO:0044260//cellular macromolecule metabolic process;GO:0008283//cell proliferation;GO:0044237//cellular metabolic process;GO:0044710//single-organism metabolic process;GO:0006629//lipid metabolic process;GO:0048856//anatomical structure development;GO:0009987//cellular process;GO:0044238//primary metabolic process;GO:0006950//response to stress;GO:0044267//cellular protein metabolic process;GO:0022610//biological adhesion;GO:0044699//single-organism process;GO:0043412//macromolecule modification;GO:0071704//organic substance [illegible]

id	CON_Mean	KD_Mean	AllMean	log2FoldChange	pvalue	qvalue	State	gene_id	XF_1_count	XF_1_FPKM	XF_1_TPM	XF_1_CPM	XF_2_count	XF_2_FPKM	XF_2_TPM	XF_2_CPM	XF_3_count	XF_3_FPKM	XF_3_TPM	XF_3_CPM	Symbol	Description	KEGG_A_class	KEGG_B_class	Pathway	K_ID	GO Component	GO Function	GO Process
RHBDL1	1.55592107	3.538725563	2.547323316	2.018817326	0.000374845	0.025406957	Up	ENSG00000103269	95	1.524098947	5.754558985	2.863781922	355	6.294460649	25.279341111	11.827291559	325	5.866361519	23.599786138	11.022893294	RHBDL1	rhomboid like 1 [Source:HGNC Symbol;Acc:HGNC:10007]	-	-	-	-	GO:0005575//cellular_component;GO:0071944//cell periphery;GO:0044464//cell part;GO:0016020//membrane;GO:0005886//plasma membrane;GO:0005623//cell	GO:0003674//molecular_function;GO:0008233//peptidase activity;GO:0003824//catalytic activity;GO:0016787//hydrolase activity	GO:0050896//response to stimulus;GO:0023052//signaling;GO:0044763//single-organism cellular process;GO:0051716//cellular response to stimulus;GO:0050789//regulation of biological process;GO:0065007//biological regulation;GO:0044699//single-organism process;GO:0044700//single organism signaling;GO:0007154//cell communication;GO:0009987//cellular process;GO:0008150//biological_process;GO:0007165//signal transduction;GO:0050794//regulation of cellular process
PLEKHA2	4.130370188	2.466152164	3.298261176	-1.68101	0.000375252	0.025406957	Down	ENSG00000169499	582	2.772508282	10.468193336	17.544432406	159	0.83711978	3.36197772	5.297293966	161	0.862922212	3.471449821	5.460571755	PLEKHA2	pleckstrin homology domain containing A2 [Source:HGNC Symbol;Acc:HGNC:14336]	-	-	-	-	-	GO:0005488//binding;GO:0043167//ion binding;GO:0008289//lipid binding;GO:0003674//molecular_function	-
SLCO4A1-AS1	3.676403216	1.861544738	2.768973977	-1.82342	0.00038229	0.025742926	Down	ENSG00000232803	424	8.332145302	31.459782647	12.781510893	89	1.932954347	7.762986381	2.965151968	122	2.697408448	10.851404623	4.13782456	-	-	-	-	-	-	-	-	-

id	CON_Mean	KD_Mean	AllMean	log2FoldChange	pvalue	qvalue	State	gene_id	XF_1_count	XF_1_FPKM	XF_1_TPM	XF_1_CPM	XF_2_count	XF_2_FPKM	XF_2_TPM	XF_2_CPM	XF_3_count	XF_3_FPKM	XF_3_TPM	XF_3_CPM	Symbol	Description	KEGG_A_class	KEGG_B_class	Pathway	K_ID	GO Component	GO Function	GO Process
CHERP	6.137888541	7.172171058	6.6550298	1.048216619	0.000384553	0.025742926	Up	ENSG00000085872	2350	11.777376762	44.467985094	70.840921225	4884	27.051850174	108.643613237	162.716878794	3695	20.834882396	83.816649747	125.321817609	CHERP	calcium homeostasis endoplasmic reticulum protein [Source:HGNC Symbol;Acc:HGNC:16930]	Genetic Information Processing	Transcription	ko03040//Spliceosome	K12841	GO:0012505//endomembrane system;GO:0044422//organelle part;GO:0005654//nucleoplasm;GO:0031981//nuclear lumen;GO:0043231//intracellular membrane-bounded organelle;GO:0005634//nucleus;GO:0043233//organelle lumen;GO:0005622//intracellular;GO:0070013//intracellular organelle lumen;GO:0005575//cellular_component;GO:0044446//intracellular organelle part;GO:0005737//cytoplasm;GO:0044424//intracellular part;GO:0044464//cell part;GO:0044428//nuclear part;GO:0043226//organelle;GO:0031974//membrane-enclosed lumen;GO:0005783//endoplasmic reticulum;GO:0043227//membrane-bounded organelle;GO:0043229//intracellular organelle;GO:0044444//cytoplasmic part;GO:0005623//cell	GO:0003676//nucleic acid binding;GO:0003723//RNA binding;GO:0097159//organic cyclic compound binding;GO:0003674//molecular_function;GO:1901363//heterocyclic compound binding;GO:0005488//binding	GO:0044260//cellular macromolecule metabolic process;GO:0008283//cell proliferation;GO:0006139//nucleobase-containing compound metabolic process;GO:0042592//homeostatic process;GO:0006397//mRNA processing;GO:0065007//biological regulation;GO:0090304//nucleic acid metabolic process;GO:0051716//cellular response to stimulus;GO:0051234//establishment of localization;GO:0008150//biological_process;GO:0050789//regulation of biological process;GO:0044238//primary metabolic process;GO:0044237//cellular metabolic process;GO:0044763//single-organism cellular process;GO:0034641//cellular nitrogen compound metabolic process;GO:0048856//anatomical structure development;GO:0006810//transport;GO:0007154//cell communication;GO:0016070//RNA metabolic process;GO:0065008//regulation of biological quality;GO:0010467//gene expression;GO:0023052//signaling;GO:0006807//nitrogen compound metabolic process;GO:0071704//organic substance metabolic process;GO:0044700//single organism signaling;GO:0050794//regulation of cellular [illegible]
PTOV1-AS2	-0.09649	2.230775508	1.067143774	2.454934531	0.000385612	0.025742926	Up	ENSG00000269352	28	0.825085083	3.115283813	0.84406204	148	4.819955532	19.35754417	4.930814509	124	4.11102394	16.53855409	4.205657749	-	-	-	-	-	-	-	-	-

id	CON_Mean	KD_Mean	AllMean	log2FoldChange	pvalue	qvalue	State	gene_id	XF_1_count	XF_1_FPKM	XF_1_TPM	XF_1_CPM	XF_2_count	XF_2_FPKM	XF_2_TPM	XF_2_CPM	XF_3_count	XF_3_FPKM	XF_3_TPM	XF_3_CPM	Symbol	Description	KEGG_A_class	KEGG_B_class	Pathway	K_ID	GO Component	GO Function	GO Process
TBX3	5.143551989	6.27321909	5.70838554	1.158618042	0.00038752	0.025742926	Up	ENSG00000135111	1178	5.958203999	22.496463511	35.510895831	2778	15.528980031	62.366325765	92.552720985	1866	10.618853323	42.718585727	63.288365808	TBX3	T-box transcription factor 3 [Source:HGNC Symbol;Acc:HGNC:11602]	Cellular Processes	Cellular community	ko04550//Signaling pathways regulating pluripotency of stem cells	K10177	GO:0044422//organelle part;GO:0005575//cellular_component;GO:0005694//chromosome;GO:0044464//cell part;GO:0043231//intracellular membrane-bounded organelle;GO:0070013//intracellular organelle lumen;GO:0044428//nuclear part;GO:0044424//intracellular part;GO:0043228//non-membrane-bounded organelle;GO:0043229//intracellular organelle;GO:0043226//organelle;GO:0043232//intracellular non-membrane-bounded organelle;GO:0044446//intracellular organelle part;GO:0043227//membrane-bounded organelle;GO:0000228//nuclear chromosome;GO:0043233//organelle lumen;GO:0031974//membrane-enclosed lumen;GO:0005622//intracellular;GO:0005634//nucleus;GO:0031981//nuclear lumen;GO:0005623//cell	GO:0003700//transcription factor activity, sequence-specific DNA binding;GO:0003674//molecular_function;GO:1901363//heterocyclic compound binding;GO:0001071//nucleic acid binding transcription factor activity;GO:0003677//DNA binding;GO:0097159//organic cyclic compound binding;GO:0008134//transcription factor binding;GO:0005488//binding;GO:0003676//nucleic acid binding;GO:0005515//protein binding	GO:0048856//anatomical structure development;GO:0048646//anatomical structure formation involved in morphogenesis;GO:0009653//anatomical structure morphogenesis;GO:0044763//single-organism cellular process;GO:0030154//cell differentiation;GO:0050896//response to stimulus;GO:0009058//biosynthetic process;GO:0007275//multicellular organism development;GO:0032502//developmental process;GO:0008219//cell death;GO:0007154//cell communication;GO:0007267//cell-cell signaling;GO:0007049//cell cycle;GO:0008150//biological_process;GO:0023052//signaling;GO:0008152//metabolic process;GO:0006810//transport;GO:0044767//single-organism developmental process;GO:0044699//single-organism process;GO:0044237//cellular metabolic process;GO:0044700//single organism signaling;GO:0000003//reproduction;GO:0032501//multicellular organismal process;GO:0034641//cellular nitrogen compound metabolic process;GO:0044707//single-multicellular organism process;GO:0008283//cell proliferation;GO:0007568//aging;GO:0006950//response to [illegible]
LINC02802	0.588672888	-1.4641	-0.43771	-2.35701	0.000389178	0.025742926	Down	ENSG00000232527	47	1.613688411	6.092823015	1.416818424	10	0.379456882	1.523946291	0.333163142	6	0.231776274	0.932412788	0.203499569	-	-	-	-	-	-	-	-	-

id	CON_Mean	KD_Mean	AllMean	log2FoldChange	pvalue	qvalue	State	gene_id	XF_1_count	XF_1_FPKM	XF_1_TPM	XF_1_CPM	XF_2_count	XF_2_FPKM	XF_2_TPM	XF_2_CPM	XF_3_count	XF_3_FPKM	XF_3_TPM	XF_3_CPM	Symbol	Description	KEGG_A_class	KEGG_B_class	Pathway	K_ID	GO Component	GO Function	GO Process
DCAF10	6.166418552	5.125487016	5.645952784	-1.03302	0.000392639	0.025766196	Down	ENSG00000122741	2397	8.715201984	32.9060944 33	72.2577396 49	917	3.684846239	14.7988033	30.5510601 67	1148	4.696206824	18.8923707 37	38.9362507 76	DCAF10	DDB1 and CUL4 associated factor 10 [Source:HGNC Symbol;Acc:HGNC:23686]	-	-	-	-	GO:0044464//cell part;GO:0043229//intracellular organelle;GO:0005623//cell;GO:0043231//intracellular membrane-bounded organelle;GO:0043233//organelle lumen;GO:0005634//nucleus;GO:0044422//organelle part;GO:0044428//nuclear part;GO:0031974//membrane-enclosed lumen;GO:0044424//intracellular part;GO:0043227//membrane-bounded organelle;GO:0031981//nuclear lumen;GO:0070013//intracellular organelle lumen;GO:0032991//macromolecular complex;GO:0005575//cellular_component;GO:0043226//organelle;GO:0044446//intracellular organelle part;GO:0005654//nucleoplasm;GO:0005622//intracellular	GO:0003674//molecular_function	GO:0009987//cellular process;GO:0044238//primary metabolic process;GO:0044237//cellular metabolic process;GO:0036211//protein modification process;GO:0008152//metabolic process;GO:0071704//organic substance metabolic process;GO:0043412//macromolecule modification;GO:0006464//cellular protein modification process;GO:0044267//cellular protein metabolic process;GO:0019538//protein metabolic process;GO:0043170//macromolecule metabolic process;GO:0044260//cellular macromolecule metabolic process;GO:0008150//biological_process
ZNF142	4.330313061	5.547479162	4.938896112	1.236927957	0.000401134	0.026091679	Up	ENSG00000115568	669	1.744554822	6.586936921	20.1670537 44	1605	4.625664738	18.5772480 45	53.472684337	1179	3.459140589	13.9157769 02	39.9876652 13	ZNF142	zinc finger protein 142 [Source:HGNC Symbol;Acc:HGNC:12927]	-	-	-	-	GO:0005575//cellular_component;GO:0043229//intracellular organelle;GO:0005623//cell;GO:0044424//intracellular part;GO:0005622//intracellular;GO:0043231//intracellular membrane-bounded organelle;GO:0043227//membrane-bounded organelle;GO:0005634//nucleus;GO:0043226//organelle;GO:0044464//cell part	GO:0003674//molecular_function;GO:0043167//ion binding;GO:0005488//binding;GO:1901363//heterocyclic compound binding;GO:0003677//DNA binding;GO:0097159//organic cyclic compound binding;GO:0003676//nucleic acid binding	GO:0008150//biological_process
RN7SL192P	-2.41729	0.112799307	-1.15224	3.396294199	0.000402187	0.026091679	Up	ENSG00000276757	3	0.302458925	1.141997852	0.090435219	29	3.231348205	12.9774984 83	0.966173113	29	3.289569391	13.2336089 16	0.983581248	-	-	-	-	-	-	-	-	-

id	CON_Mean	KD_Mean	AllMean	log2FoldChange	pvalue	qvalue	State	gene_id	XF_1_count	XF_1_FPKM	XF_1_TPM	XF_1_CPM	XF_2_count	XF_2_FPKM	XF_2_TPM	XF_2_CPM	XF_3_count	XF_3_FPKM	XF_3_TPM	XF_3_CPM	Symbol	Description	KEGG_A_class	KEGG_B_class	Pathway	K_ID	GO Component	GO Function	GO Process
SDHAF4	2.531816101	0.579053296	1.555434699	-2.00973	0.000413246	0.026542439	Down	ENSG00000154079	190	3.977474891	15.017800462	5.727563844	36	0.832907856	3.345062109	1.199387313	47	1.106999968	4.453350242	1.594079953	SDHAF4	succinate dehydrogenase complex assembly factor 4 [Source:HGNC Symbol;Acc:HGNC:20957]	-	-	-	-	GO:0005623//cell;GO:0032991//macromolecular complex;GO:0044464//cell part;GO:0043229//intracellular organelle;GO:0043227//membrane-bounded organelle;GO:0005739//mitochondrion;GO:0005622//intracellular;GO:0044424//intracellular part;GO:0044444//cytoplasmic part;GO:0005737//cytoplasm;GO:0043231//intracellular membrane-bounded organelle;GO:0043226//organelle;GO:0005575//cellular_component	GO:0003824//catalytic activity;GO:0003674//molecular_function;GO:0016491//oxidoreductase activity	GO:0071840//cellular component organization or biogenesis;GO:0009987//cellular process;GO:0007005//mitochondrion organization;GO:0050896//response to stimulus;GO:0002376//immune system process;GO:0006091//generation of precursor metabolites and energy;GO:0043933//macromolecular complex subunit organization;GO:0065003//macromolecular complex assembly;GO:0008150//biological_process;GO:0008152//metabolic process;GO:0006950//response to stress;GO:0016043//cellular component organization;GO:0006996//organelle organization;GO:0044237//cellular metabolic process;GO:0044085//cellular component biogenesis;GO:0022607//cellular component assembly
VSIG2	-0.19189	2.214911688	1.011511716	2.58636521	0.000422029	0.026789688	Up	ENSG00000019102	26	0.541653002	2.045125848	0.783771894	174	4.006246496	16.089586924	5.797038679	103	2.41424275	9.712257317	3.493409259	VSIG2	V-set and immunoglobulin domain containing 2 [Source:HGNC Symbol;Acc:HGNC:17149]	-	-	-	-	GO:0044464//cell part;GO:0016020//membrane;GO:0005886//plasma membrane;GO:0005575//cellular_component;GO:0071944//cell periphery;GO:0005623//cell	-	-
EDIL3	4.190716932	2.174721347	3.182719139	-1.93156	0.000429333	0.027033339	Down	ENSG00000164176	607	3.141297721	11.860636122	18.298059227	88	0.503319426	2.021393755	2.931835654	192	1.117937544	4.497351018	6.511986192	EDIL3	EGF like repeats and discoidin domains 3 [Source:HGNC Symbol;Acc:HGNC:3173]	-	-	-	-	GO:0005576//extracellular region;GO:0005575//cellular_component;GO:0005615//extracellular space;GO:0031012//extracellular matrix;GO:0044421//extracellular region part;GO:0043226//organelle	GO:0005488//binding;GO:0003674//molecular_function;GO:0043167//ion binding;GO:0005198//structural molecule activity	GO:0007155//cell adhesion;GO:0048856//anatomical structure development;GO:0032502//developmental process;GO:0008150//biological_process;GO:0022610//biological adhesion
ENSG00000254964	-3.35941	-0.77764	-2.06852	6.933515156	0.000430962	0.027033339	Up	ENSG00000254964	0	0	0	0	12	4.345606206	17.452497961	0.399795771	17	6.267196856	25.212306642	0.576582111	-	-	-	-	-	-	-	-	-
LINC00106	0.195064024	2.45410339	1.324583707	2.37023646	0.00043603	0.027223615	Up	ENSG00000236871	35	1.782225591	6.729170904	1.05507755	139	7.822580542	31.4164617	4.630967681	181	10.369769679	41.716547119	6.13890365	-	-	-	-	-	-	-	-	-
ENSG00000267096	-1.44713	0.93267937	-0.25722	2.733915683	0.000437186	0.027223615	Up	ENSG00000267096	9	0.338286354	1.277271912	0.271305656	57	2.367867721	9.509652879	1.899029912	50	2.11450092	8.506425888	1.695829738	-	-	-	-	-	-	-	-	-

id	CON_Mean	KD_Mean	AllMean	log2FoldChange	pvalue	qvalue	State	gene_id	XF_1_count	XF_1_FPKM	XF_1_TPM	XF_1_CPM	XF_2_count	XF_2_FPKM	XF_2_TPM	XF_2_CPM	XF_3_count	XF_3_FPKM	XF_3_TPM	XF_3_CPM	Symbol	Description	KEGG_A_class	KEGG_B_class	Pathway	K_ID	GO Component	GO Function	GO Process
MMP3	-1.85215	0.669200592	-0.59147	3.104651335	0.000441971	0.027269054	Up	ENSG00000149968	6	0.09755687	0.368346664	0.180870437	62	1.114137801	4.474516737	2.065611483	31	0.567105953	2.281410576	1.051414437	MMP3	matrix metallopeptidase 3 [Source:HGNC Symbol;Acc:HGNC:7173]	Environmental Information Processing;Human Diseases	Immune diseases;Cancers;Signal transduction	ko05323//Rheumatoid arthritis;ko05202//Transcriptional misregulation in cancers;ko04668//TNF signaling pathway	K01394	GO:0005576//extracellular region;GO:0031012//extracellular matrix;GO:0005575//cellular_component;GO:0044421//extracellular region part;GO:0005615//extracellular space	GO:0016787//hydrolase activity;GO:0043167//ion binding;GO:0003674//molecular_function;GO:0003824//catalytic activity;GO:0008233//peptidase activity;GO:0005488//binding	GO:0007154//cell communication;GO:0008152//metabolic process;GO:0044699//single-organism process;GO:0050896//response to stimulus;GO:0051186//cofactor metabolic process;GO:0007165//signal transduction;GO:0022607//cellular component assembly;GO:0044085//cellular component biogenesis;GO:0008219//cell death;GO:0030198//extracellular matrix organization;GO:0050794//regulation of cellular process;GO:0044700//single organism signaling;GO:0044237//cellular metabolic process;GO:0006950//response to stress;GO:0065007//biological regulation;GO:0023052//signaling;GO:0016043//cellular component organization;GO:0044763//single-organism cellular process;GO:0043062//extracellular structure organization;GO:0008150//biological_process;GO:0065003//macromolecular complex assembly;GO:0051716//cellular response to stimulus;GO:0071840//cellular component organization or biogenesis;GO:0043933//macromolecular complex subunit [illegible]
MIR3176	-0.72227	1.57130276	0.424516796	2.493085217	0.000444461	0.027269054	Up	ENSG00000266235	17	5.694069318	21.4991669778	0.512466239	92	34.0566767888	136.775872903	3.065100911	78	29.394382119	118.250661774	2.645494391	-	-	-	-	-	-	-	-	-

id	CON_Mean	KD_Mean	AllMean	log2FoldChange	pvalue	qvalue	State	gene_id	XF_1_count	XF_1_FPKM	XF_1_TPM	XF_1_CPM	XF_2_count	XF_2_FPKM	XF_2_TPM	XF_2_CPM	XF_3_count	XF_3_FPKM	XF_3_TPM	XF_3_CPM	Symbol	Description	KEGG_A_class	KEGG_B_class	Pathway	K_ID	GO Component	GO Function	GO Process
TRMT61A	4.046639572	5.360254033	4.703446802	1.351863124	0.000444894	0.027269054	Up	ENSG00000166166	549	5.144434256	19.4239031 68	16.5496450 01	1507	15.60698961	62.6796219 87	50.2076855 74	968	10.2055529 13	41.0559194 77	32.83126372	TRMT61A	tRNA methyltransferase 61A [Source:HGNC Symbol;Acc:HGNC:23790]	-	-	-	-	GO:0005622//intracellular;GO:0070013//intracellular organelle lumen;GO:0043231//intracellular membrane-bounded organelle;GO:0005634//nucleus;GO:0044464//cell part;GO:0043229//intracellular organelle;GO:0005654//nucleoplasm;GO:0044422//organelle part;GO:0031974//membrane-enclosed lumen;GO:0043226//organelle;GO:0044424//intracellular part;GO:0005623//cell;GO:0043233//organelle lumen;GO:0032991//macromolecular complex;GO:0031981//nuclear lumen;GO:0044446//intracellular organelle part;GO:0005575//cellular_component;GO:0044428//nuclear part;GO:0043227//membrane-bounded organelle	GO:0016741//transferase activity, transferring one-carbon groups;GO:0003674//molecular_function;GO:0003824//catalytic activity;GO:0008168//methyltransferase activity;GO:0016740//transferase activity	GO:0034641//cellular nitrogen compound metabolic process;GO:0046483//heterocycle metabolic process;GO:0006399//tRNA metabolic process;GO:0006807//nitrogen compound metabolic process;GO:0034660//ncRNA metabolic process;GO:0008150//biological_process;GO:0009987//cellular process;GO:0071704//organic substance metabolic process;GO:0044237//cellular metabolic process;GO:1901360//organic cyclic compound metabolic process;GO:0006139//nucleobase-containing compound metabolic process;GO:0006725//cellular aromatic compound metabolic process;GO:0043170//macromolecule metabolic process;GO:0044238//primary metabolic process;GO:0090304//nucleic acid metabolic process;GO:0016070//RNA metabolic process;GO:0008152//metabolic process;GO:0044260//cellular macromolecule metabolic process
PCDH18	4.535975091	3.00334242	3.769658755	-1.54343	0.000445907	0.027269054	Down	ENSG00000189184	772	3.480705392	13.1421418 03	23.2719962 49	230	1.146089183	4.602837482	7.662752277	237	1.202248423	4.836525259	8.038232956	PCDH18	protocadherin 18 [Source:HGNC Symbol;Acc:HGNC:14268]	-	-	-	-	GO:0005623//cell;GO:0016020//membrane;GO:0005575//cellular_component;GO:0044464//cell part;GO:0071944//cell periphery;GO:0005886//plasma membrane	GO:0043167//ion binding;GO:0005488//binding;GO:0003674//molecular_function	GO:0022610//biological adhesion;GO:0032502//developmental process;GO:0008150//biological_process;GO:0007155//cell adhesion;GO:0048856//anatomical structure development
AGMO	6.733995048	5.526539226	6.130267137	-1.14995	0.000454057	0.027668315	Down	ENSG00000187546	3554	29.85941721	112.740565766	107.13558895	1021	9.480478497	38.0747872 17	34.0159568 49	1800	17.01501409	68.44970129	61.0498705 54	AGMO	alkylglycerol monooxygenase [Source:HGNC Symbol;Acc:HGNC:33784]	-	-	-	-	GO:0005783//endoplasmic reticulum;GO:0043231//intracellular membrane-bounded organelle;GO:0005623//cell;GO:0044464//cell part;GO:0005737//cytoplasm;GO:0043227//membrane-bounded organelle;GO:0005622//intracellular;GO:0043229//intracellular organelle;GO:0043226//organelle;GO:0044424//intracellular part;GO:0012505//endomembrane system;GO:0005575//cellular_component;GO:0044444//cytoplasmic part	GO:0003674//molecular_function;GO:0016491//oxidoreductase activity;GO:0043167//ion binding;GO:0003824//catalytic activity;GO:0005488//binding	GO:0044699//single-organism process;GO:0006629//lipid metabolic process;GO:0009058//biosynthetic process;GO:0008152//metabolic process;GO:0044281//small molecule metabolic process;GO:0008150//biological_process;GO:0044238//primary metabolic process;GO:0071704//organic substance metabolic process;GO:0044710//single-organism metabolic process

id	CON_Mean	KD_Mean	AllMean	log2FoldChange	pvalue	qvalue	State	gene_id	XF_1_count	XF_1_FPKM	XF_1_TPM	XF_1_CPM	XF_2_count	XF_2_FPKM	XF_2_TPM	XF_2_CPM	XF_3_count	XF_3_FPKM	XF_3_TPM	XF_3_CPM	Symbol	Description	KEGG_A_class	KEGG_B_class	Pathway	K_ID	GO Component	GO Function	GO Process
CHST2	4.605011856	5.703716202	5.154364029	1.102509597	0.000465654	0.028173697	Up	ENSG00000175040	810	5.895101163	22.258205366	24.417509018	1595	12.829435352	51.524616745	53.139521228	1474	12.069787703	48.555549736	49.993060665	CHST2	carbohydrate sulfotransferase 2 [Source:HGNC Symbol;Acc:HGNC:1970]	Metabolism	Glycan biosynthesis and metabolism	ko00533//Glycosaminoglycan biosynthesis - keratan sulfate	K04745	GO:0005622//intracellular;GO:0044446//intracellular organelle part;GO:0043233//organelle lumen;GO:0031981//nuclear lumen;GO:0043231//intracellular membrane-bounded organelle;GO:0005575//cellular_component;GO:0005634//nucleus;GO:0005737//cytoplasm;GO:0005794//Golgi apparatus;GO:0043229//intracellular organelle;GO:0044428//nuclear part;GO:0070013//intracellular organelle lumen;GO:0044424//intracellular part;GO:0005829//cytosol;GO:0044464//cell part;GO:0043226//organelle;GO:0012505//endomembrane system;GO:0005623//cell;GO:0005654//nucleoplasm;GO:0043227//membrane-bounded organelle;GO:0044444//cytoplasmic part;GO:0044422//organelle part;GO:0031974//membrane-enclosed lumen	GO:0003674//molecular_function	GO:0044699//single-organism process;GO:0044281//small molecule metabolic process;GO:0071704//organic substance metabolic process;GO:0006950//response to stress;GO:0005975//carbohydrate metabolic process;GO:0009987//cellular process;GO:0006790//sulfur compound metabolic process;GO:0008150//biological_process;GO:0008152//metabolic process;GO:0032502//developmental process;GO:0044710//single-organism metabolic process;GO:0044237//cellular metabolic process;GO:0048856//anatomical structure development;GO:0050896//response to stimulus;GO:0044238//primary metabolic process;GO:0009058//biosynthetic process
RN7SL1	4.376770068	5.522801628	4.949785848	1.149732606	0.000470409	0.028269819	Up	ENSG00000276168	691	69.666372399	263.040172013	20.830245347	1378	153.544752628	616.654927929	45.909881036	1327	150.52615798	605.551690747	45.007321236	-	-	-	-	-	-	-	-	-

id	CON_Mean	KD_Mean	AllMean	log2FoldChange	pvalue	qvalue	State	gene_id	XF_1_count	XF_1_FPKM	XF_1_TPM	XF_1_CPM	XF_2_count	XF_2_FPKM	XF_2_TPM	XF_2_CPM	XF_3_count	XF_3_FPKM	XF_3_TPM	XF_3_CPM	Symbol	Description	KEGG_A_class	KEGG_B_class	Pathway	K_ID	GO Component	GO Function	GO Process
MARK2	5.376503944	6.393744095	5.885124019	1.030272587	0.000470556	0.028269819	Up	ENSG00000072518	1385	3.47634687	13.125685281	41.750925913	2194	6.086260905	24.443184903	73.095993463	2793	7.8875145	31.730682595	94.729049143	MARK2	microtubule affinity regulating kinase 2 [Source:HGNC Symbol;Acc:HGNC:3332]	-	-	-	-	GO:0044444//cytoplasmic part;GO:0005575//cellular_component;GO:0031974//membrane-enclosed lumen;GO:0043231//intracellular membrane-bounded organelle;GO:0005739//mitochondrion;GO:0005737//cytoplasm;GO:0043226//organelle;GO:0043229//intracellular organelle;GO:0043233//organelle lumen;GO:0043232//intracellular non-membrane-bounded organelle;GO:0005856//cytoskeleton;GO:0044464//cell part;GO:0016020//membrane;GO:0044422//organelle part;GO:0071944//cell periphery;GO:0005622//intracellular;GO:0070013//intracellular organelle lumen;GO:0031981//nuclear lumen;GO:0005654//nucleoplasm;GO:0005623//cell;GO:0005634//nucleus;GO:0044446//intracellular organelle part;GO:0044424//intracellular part;GO:0005886//plasma membrane;GO:0044428//nuclear part;GO:0043228//non-membrane-bounded organelle;GO:0043227//membrane-bounded organelle	GO:0030234//enzyme regulator activity;GO:0005515//protein binding;GO:0016772//transferase activity, transferring phosphorus-containing groups;GO:0016740//transferase activity;GO:0008092//cytoskeletal protein binding;GO:1901363//heterocyclic compound binding;GO:0003676//nucleic acid binding;GO:0043167//ion binding;GO:0003824//catalytic activity;GO:0097159//organic cyclic compound binding;GO:0098772//molecular function regulator;GO:0008289//lipid binding;GO:0003674//molecular_function;GO:0003723//RNA binding;GO:0016301//kinase activity;GO:0005488//binding	GO:0044763//single-organism cellular process;GO:0006464//cellular protein modification process;GO:0048870//cell motility;GO:0032502//developmental process;GO:0050789//regulation of biological process;GO:0051716//cellular response to stimulus;GO:0000902//cell morphogenesis;GO:0006914//autophagy;GO:0009987//cellular process;GO:0006928//movement of cell or subcellular component;GO:0065007//biological regulation;GO:0044700//single organism signaling;GO:0007005//mitochondrion organization;GO:0008150//biological_process;GO:0044699//single-organism process;GO:0071704//organic substance metabolic process;GO:0009056//catabolic process;GO:0044238//primary metabolic process;GO:0050794//regulation of cellular process;GO:0071840//cellular component organization or biogenesis;GO:0007154//cell communication;GO:0048869//cellular developmental process;GO:0016043//cellular component organization;GO:0048856//anatomical structure development;GO:0032989//cellular component morphogenesis;GO:003621 [illegible]

id	CON_Mean	KD_Mean	AllMean	log2FoldChange	pvalue	qvalue	State	gene_id	XF_1_count	XF_1_FPKM	XF_1_TPM	XF_1_CPM	XF_2_count	XF_2_FPKM	XF_2_TPM	XF_2_CPM	XF_3_count	XF_3_FPKM	XF_3_TPM	XF_3_CPM	Symbol	Description	KEGG_A_class	KEGG_B_class	Pathway	K_ID	GO Component	GO Function	GO Process
RBMS3	5.644163034	4.480622296	5.062392665	-1.15199	0.000474872	0.028429028	Down	ENSG00000144642	1668	2.727232279	10.297244182	50.281981533	570	1.030010258	4.136650002	18.990299122	753	1.385214289	5.572578654	25.539195848	RBMS3	RNA binding motif single stranded interacting protein 3 [Source:HGNC Symbol;Acc:HGNC:13427]	-	-	-	-	GO:0044424//intracellular part;GO:0044464//cell part;GO:0005622//intracellular;GO:0005575//cellular_component;GO:0005623//cell;GO:0032991//macromolecular complex;GO:0005737//cytoplasm	GO:0044822//poly(A) RNA binding;GO:0005488//binding;GO:0003723//RNA binding;GO:0003729//mRNA binding;GO:0003674//molecular_function;GO:0097159//organic cyclic compound binding;GO:0003676//nucleic acid binding;GO:1901363//heterocyclic compound binding	GO:0008152//metabolic process;GO:0034645//cellular macromolecule biosynthetic process;GO:0023052//signaling;GO:0043604//amide biosynthetic process;GO:0050896//response to stimulus;GO:0044699//single-organism process;GO:0044267//cellular protein metabolic process;GO:0009987//cellular process;GO:0065007//biological regulation;GO:0043043//peptide biosynthetic process;GO:0050794//regulation of cellular process;GO:1901564//organonitrogen compound metabolic process;GO:0007267//cell-cell signaling;GO:0009059//macromolecule biosynthetic process;GO:0044763//single-organism cellular process;GO:0010467//gene expression;GO:0050789//regulation of biological process;GO:0006518//peptide metabolic process;GO:0044249//cellular biosynthetic process;GO:0006412//translation;GO:0006807//nitrogen compound metabolic process;GO:0044260//cellular macromolecule metabolic process;GO:0051716//cellular response to stimulus;GO:0007165//signal transduction;GO:0043603//[illegible]

id	CON_Mean	KD_Mean	AllMean	log2FoldChange	pvalue	qvalue	State	gene_id	XF_1_count	XF_1_FPKM	XF_1_TPM	XF_1_CPM	XF_2_count	XF_2_FPKM	XF_2_TPM	XF_2_CPM	XF_3_count	XF_3_FPKM	XF_3_TPM	XF_3_CPM	Symbol	Description	KEGG_A_class	KEGG_B_class	Pathway	K_ID	GO Component	GO Function	GO Process
USB1	5.073483596	6.105273756	5.589378676	1.040339863	0.00048417	0.028821607	Up	ENSG00000103005	1122	4.108195281	15.511363033	33.822771751	2240	9.064562604	36.404416977	74.628543919	1833	7.551210759	30.377766255	62.169118181	USB1	U6 snRNA biogenesis phosphodiesterase 1 [Source:HGNC Symbol;Acc:HGNC:25792]	-	-	-	-	GO:0031981//nuclear lumen;GO:0044422//organelle part;GO:0044446//intracellular organelle part;GO:0005576//extracellular region;GO:0005622//intracellular;GO:0043233//organelle lumen;GO:0043227//membrane-bounded organelle;GO:0005634//nucleus;GO:0005623//cell;GO:0031974//membrane-enclosed lumen;GO:0044424//intracellular part;GO:0044428//nuclear part;GO:0005654//nucleoplasm;GO:0043229//intracellular organelle;GO:0070013//intracellular organelle lumen;GO:0044464//cell part;GO:0043231//intracellular membrane-bounded organelle;GO:0043226//organelle;GO:0005575//cellular_component	GO:0003674//molecular_function;GO:0016788//hydrolase activity, acting on ester bonds;GO:0016787//hydrolase activity;GO:0003824//catalytic activity;GO:0004518//nuclease activity	GO:0008152//metabolic process;GO:0009987//cellular process;GO:0006807//nitrogen compound metabolic process;GO:0044237//cellular metabolic process;GO:0034641//cellular nitrogen compound metabolic process;GO:0008150//biological_process
DBP	4.304327845	2.499206748	3.401767297	-1.76656	0.000486498	0.028821607	Down	ENSG00000105516	657	3.054018947	11.531096588	19.805312887	124	0.637042863	2.558443803	4.131222967	216	1.129681491	4.544595742	7.325984466	DBP	D-box binding PAR bZIP transcription factor [Source:HGNC Symbol;Acc:HGNC:2697]	-	-	-	-	GO:0005694//chromosome;GO:0005622//intracellular;GO:0005623//cell;GO:0043228//non-membrane-bounded organelle;GO:0043229//intracellular organelle;GO:0000228//nuclear chromosome;GO:0043227//membrane-bounded organelle;GO:0005575//cellular_component;GO:0044464//cell part;GO:0043232//intracellular non-membrane-bounded organelle;GO:0070013//intracellular organelle lumen;GO:0005634//nucleus;GO:0043231//intracellular membrane-bounded organelle;GO:0031974//membrane-enclosed lumen;GO:0043226//organelle;GO:0044424//intracellular part;GO:0044446//intracellular organelle part;GO:0044428//nuclear part;GO:0043233//organelle lumen;GO:0044422//organelle part;GO:0031981//nuclear lumen	GO:0005488//binding;GO:0001071//nucleic acid binding transcription factor activity;GO:0003676//nucleic acid binding;GO:0003700//transcription factor activity, sequence-specific DNA binding;GO:0097159//organic cyclic compound binding;GO:0003674//molecular_function;GO:1901363//heterocyclic compound binding;GO:0003677//DNA binding	GO:0048856//anatomical structure development;GO:0008152//metabolic process;GO:0006807//nitrogen compound metabolic process;GO:0044237//cellular metabolic process;GO:0008150//biological_process;GO:0009058//biosynthetic process;GO:0034641//cellular nitrogen compound metabolic process;GO:0009987//cellular process;GO:0032502//developmental process

id	CON_Mean	KD_Mean	AllMean	log2FoldChange	pvalue	qvalue	State	gene_id	XF_1_count	XF_1_FPKM	XF_1_TPM	XF_1_CPM	XF_2_count	XF_2_FPKM	XF_2_TPM	XF_2_CPM	XF_3_count	XF_3_FPKM	XF_3_TPM	XF_3_CPM	Symbol	Description	KEGG_A_class	KEGG_B_class	Pathway	K_ID	GO Component	GO Function	GO Process
SLC9A1	4.467376362	5.62121089	5.044293626	1.166820153	0.000492868	0.029098001	Up	ENSG00000090020	736	3.581400101	13.522336045	22.186773626	1634	8.787547616	35.291890145	54.438857484	1283	7.024211633	28.257701436	43.514991067	SLC9A1	solute carrier family 9 member A1 [Source:HGNC Symbol;Acc:HGNC:11071]	Organismal Systems;Environmental Information Processing;Human Diseases;Cellular Processes	Cell motility;Cancers;Endocrine system;Signal transduction;Circulatory system;Digestive system	ko04810//Regulation of actin cytoskeleton;ko05205//Proteoglycans in cancer;ko04024//cAMP signaling pathway;ko04261//Adrenergic signaling in cardiomyocytes;ko04919//Thyroid hormone [illegible]	K05742	GO:0044464//cell part;GO:0044424//intracellular part;GO:0044444//cytoplasmic part;GO:0043229//intracellular organelle;GO:0012505//endomembrane system;GO:0005615//extracellular space;GO:0032991//macromolecular complex;GO:0005623//cell;GO:0005654//nucleoplasm;GO:0005737//cytoplasm;GO:0005783//endoplasmic reticulum;GO:0005739//mitochondrion;GO:0005576//extracellular region;GO:0043233//organelle lumen;GO:0071944//cell periphery;GO:0031974//membrane-enclosed lumen;GO:0043226//organelle;GO:0005886//plasma membrane;GO:0070013//intracellular organelle lumen;GO:0044446//intracellular organelle part;GO:0005575//cellular_component;GO:0005634//nucleus;GO:0043227//membrane-bounded organelle;GO:0031981//nuclear lumen;GO:0044421//extracellular region part;GO:0044422//organelle part;GO:0044428//nuclear part;GO:0005622//intracellular;GO:0043231//intracellular membrane-bounded organelle;GO:0016020//membrane	GO:0005215//transporter activity;GO:0008289//lipid binding;GO:0060090//binding, bridging;GO:0005488//binding;GO:0022857//transmembrane transporter activity;GO:0019899//enzyme binding;GO:0043167//ion binding;GO:0005515//protein binding;GO:0030674//protein binding, bridging;GO:0003674//molecular_function	GO:0022607//cellular component assembly;GO:0044763//single-organism cellular process;GO:0044767//single-organism developmental process;GO:0006928//movement of cell or subcellular component;GO:0008150//biological_process;GO:0048869//cellular developmental process;GO:0044281//small molecule metabolic process;GO:0044085//cellular component biogenesis;GO:0051179//localization;GO:0055085//transmembrane transport;GO:0006950//response to stress;GO:0032502//developmental process;GO:0006810//transport;GO:0048870//cell motility;GO:0061024//membrane organization;GO:0040007//growth;GO:0044237//cellular metabolic process;GO:0040011//locomotion;GO:0006807//nitrogen compound metabolic process;GO:0006996//organelle organization;GO:0003013//circulatory system process;GO:0051716//cellular response to stimulus;GO:0009987//cellular process;GO:0009056//catabolic process;GO:0023052//signaling;GO:0003008//system process;GO:0022610//biological adhesion;GO:0034330//cell junction [illegible]

id	CON_Mean	KD_Mean	AllMean	log2FoldChange	pvalue	qvalue	State	gene_id	XF_1_count	XF_1_FPKM	XF_1_TPM	XF_1_CPM	XF_2_count	XF_2_FPKM	XF_2_TPM	XF_2_CPM	XF_3_count	XF_3_FPKM	XF_3_TPM	XF_3_CPM	Symbol	Description	KEGG_A_class	KEGG_B_class	Pathway	K_ID	GO Component	GO Function	GO Process
AKR1C2	4.811283881	2.73268378	3.771983831	-1.91549	0.000504197	0.029460983	Down	ENSG00000151632	935	4.362427353	16.471270166	28.185643126	116	0.598157012	2.402273361	3.864692453	320	1.679818963	6.757743816	10.853310321	AKR1C2	aldo-keto reductase family 1 member C2 [Source:HGNC Symbol;Acc:HGNC:385]	Metabolism; Human Diseases	Lipid metabolism; Xenobiotics biodegradation and metabolism; Cancers	ko05204//Chemical carcinogenesis;ko00980//Metabolism of xenobiotics by cytochrome P450;ko00140//Steroid hormone biosynthesis	K00089	GO:0044444//cytoplasmic part;GO:0005622//intracellular;GO:0005737//cytoplasm;GO:0005829//cytosol;GO:0005575//cellular_component;GO:0044424//intracellular part;GO:0005623//cell;GO:0044464//cell part	GO:0003674//molecular_function;GO:0003824//catalytic activity;GO:0005488//binding;GO:0016491//oxidoreductase activity;GO:0043167//ion binding	GO:0044238//primary metabolic process;GO:0007165//signal transduction;GO:0048869//cellular developmental process;GO:0008150//biological_process;GO:0032502//developmental process;GO:0044699//single-organism process;GO:0007154//cell communication;GO:0044237//cellular metabolic process;GO:0065007//biological regulation;GO:0019748//secondary metabolic process;GO:0051186//cofactor metabolic process;GO:0051716//cellular response to stimulus;GO:0050896//response to stimulus;GO:0050794//regulation of cellular process;GO:0008283//cell proliferation;GO:0023052//signaling;GO:0044767//single-organism developmental process;GO:0006629//lipid metabolic process;GO:0044281//small molecule metabolic process;GO:0009987//cellular process;GO:0008152//metabolic process;GO:0050789//regulation of biological process;GO:0044700//single organism signaling;GO:0030154//cell differentiation;GO:0048856//anatomical structure development;GO:0071704//organic substance [illegible]

id	CON_Mean	KD_Mean	AllMean	log2FoldChange	pvalue	qvalue	State	gene_id	XF_1_count	XF_1_FPKM	XF_1_TPM	XF_1_CPM	XF_2_count	XF_2_FPKM	XF_2_TPM	XF_2_CPM	XF_3_count	XF_3_FPKM	XF_3_TPM	XF_3_CPM	Symbol	Description	KEGG_A_class	KEGG_B_class	Pathway	K_ID	GO Component	GO Function	GO Process
DHX34	4.725766383	5.879371665	5.302569024	1.182446726	0.000511722	0.029596622	Up	ENSG00000134815	881	4.690535004	17.710110226	26.557809191	2107	12.397999669	49.791917088	70.197474124	1424	8.530065512	34.315601103	48.297230927	DHX34	DExH-box helicase 34 [Source:HGNC Symbol;Acc:HGNC:16719]	-	-	-	-	GO:0005575//cellular_component	GO:0016818//hydrolase activity, acting on acid anhydrides, in phosphorus-containing anhydrides;GO:0097159//organic cyclic compound binding;GO:0016817//hydrolase activity, acting on acid anhydrides;GO:0016787//hydrolase activity;GO:0005488//binding;GO:1901363//heterocyclic compound binding;GO:0016462//pyrophosphatase activity;GO:0016887//ATPase activity;GO:0017111//nucleoside-triphosphatase activity;GO:0004386//helicase activity;GO:0003723//RNA binding;GO:0003674//molecular_function;GO:0003676//nucleic acid binding;GO:0003824//catalytic activity;GO:0043167//ion binding	GO:0019439//aromatic compound catabolic process;GO:0006139//nucleobase-containing compound metabolic process;GO:1901360//organic cyclic compound metabolic process;GO:0046483//heterocycle metabolic process;GO:0006725//cellular aromatic compound metabolic process;GO:1901575//organic substance catabolic process;GO:0034641//cellular nitrogen compound metabolic process;GO:0044270//cellular nitrogen compound catabolic process;GO:0009987//cellular process;GO:0009056//catabolic process;GO:0044248//cellular catabolic process;GO:0034655//nucleobase-containing compound catabolic process;GO:0046700//heterocycle catabolic process;GO:0006807//nitrogen compound metabolic process;GO:1901361//organic cyclic compound catabolic process;GO:0008150//biological_process;GO:0008152//metabolic process;GO:0044238//primary metabolic process;GO:0044237//cellular metabolic process;GO:0071704//organic substance metabolic process
OLMALINC	5.931980463	4.493281323	5.212630893	-1.35518	0.000513904	0.029622373	Down	ENSG00000235823	2037	4.512125316	17.036486599	61.405513419	467	1.143266864	4.591502697	15.558718755	935	2.330223829	9.374257595	31.712016093	-	-	-	-	-	-	-	-	-

id	CON_Mean	KD_Mean	AllMean	log2FoldChange	pvalue	qvalue	State	gene_id	XF_1_count	XF_1_FPKM	XF_1_TPM	XF_1_CPM	XF_2_count	XF_2_FPKM	XF_2_TPM	XF_2_CPM	XF_3_count	XF_3_FPKM	XF_3_TPM	XF_3_CPM	Symbol	Description	KEGG_A_class	KEGG_B_class	Pathway	K_ID	GO Component	GO Function	GO Process
ADRA1B	-0.95352	1.393933209	0.220207059	2.59028979	0.000529378	0.030411635	Up	ENSG00000170214	14	0.134105821	0.506344986	0.42203102	83	0.878695292	3.528950175	2.765254083	67	0.72208829	2.904889029	2.272411848	ADRA1B	adrenoceptor alpha 1B [Source:HGNC Symbol;Acc:HGNC:278]	Organismal Systems;Environmental Information Processing	Signal transduction;Circulatory system;Digestive system;Signaling molecules and interaction	ko04080//Neuroactive ligand-receptor interaction;ko04020//Calcium signaling pathway;ko04022//cGMP - PKG signaling pathway;ko04261//Adrenergic signaling in cardiomyocytes;ko04270/[illegible]	K04136	GO:0044464//cell part;GO:0016020//membrane;GO:0043227//membrane-bounded organelle;GO:0005622//intracellular;GO:0005886//plasma membrane;GO:0031975//envelope;GO:0012505//endomembrane system;GO:0031967//organelle envelope;GO:0043229//intracellular organelle;GO:0005575//cellular_component;GO:0043231//intracellular membrane-bounded organelle;GO:0044446//intracellular organelle part;GO:0005634//nucleus;GO:0005737//cytoplasm;GO:0071944//cell periphery;GO:0005635//nuclear envelope;GO:0043226//organelle;GO:0005623//cell;GO:0044424//intracellular part;GO:0044422//organelle part;GO:0044428//nuclear part	GO:0003674//molecular_function	GO:0050896//response to stimulus;GO:0050794//regulation of cellular process;GO:0008150//biological_process;GO:0023052//signaling;GO:0006464//cellular protein modification process;GO:0043170//macromolecule metabolic process;GO:0044237//cellular metabolic process;GO:0050789//regulation of biological process;GO:0008152//metabolic process;GO:0003013//circulatory system process;GO:0044238//primary metabolic process;GO:0007154//cell communication;GO:0065007//biological regulation;GO:0044260//cellular macromolecule metabolic process;GO:0007267//cell-cell signaling;GO:0009987//cellular process;GO:0007165//signal transduction;GO:0044699//single-organism process;GO:0051716//cellular response to stimulus;GO:0065008//regulation of biological quality;GO:0071704//organic substance metabolic process;GO:0044763//single-organism cellular process;GO:0019538//protein metabolic process;GO:0044700//single organism signaling;GO:0036211//protein modification process;GO:0032501//multi[illegible]
FBRSL1	5.376503944	6.393375175	5.884939559	1.032064753	0.000532245	0.030473732	Up	ENSG00000112787	1385	4.444897893	16.7826551 92	41.7509259 13	2854	10.1229384 51	40.6549867 31	95.0847608 68	2146	7.748856844	31.1728767 04	72.7850123 38	FBRSL1	fibrosin like 1 [Source:HGNC Symbol;Acc:HGNC:29308]	-	-	-	-	-	GO:0003723//RNA binding;GO:0003674//molecular_function;GO:0097159//organic cyclic compound binding;GO:0003676//nucleic acid binding;GO:0005488//binding;GO:1901363//heterocyclic compound binding	-

id	CON_Mean	KD_Mean	AllMean	log2FoldChange	pvalue	qvalue	State	gene_id	XF_1_count	XF_1_FPKM	XF_1_TPM	XF_1_CPM	XF_2_count	XF_2_FPKM	XF_2_TPM	XF_2_CPM	XF_3_count	XF_3_FPKM	XF_3_TPM	XF_3_CPM	Symbol	Description	KEGG_A_class	KEGG_B_class	Pathway	K_ID	GO Component	GO Function	GO Process
FOXP4	4.43780205	5.562679953	5.000241001	1.128815323	0.000540155	0.030767522	Up	ENSG00000137166	721	3.380712013	12.764595584	21.734597533	1437	7.446810325	29.907321516	47.875543576	1345	7.095632282	28.54501957	45.617819942	FOXP4	forkhead box P4 [Source:HGNC Symbol;Acc:HGNC:20842]	-	-	-	-	GO:0043227//membrane-bounded organelle;GO:0005623//cell;GO:0043228//non-membrane-bounded organelle;GO:0043232//intracellular non-membrane-bounded organelle;GO:0005575//cellular_component;GO:0031981//nuclear lumen;GO:0044428//nuclear part;GO:0043226//organelle;GO:0005622//intracellular;GO:0044464//cell part;GO:0044424//intracellular part;GO:0005634//nucleus;GO:0044446//intracellular organelle part;GO:0043229//intracellular organelle;GO:0043231//intracellular membrane-bounded organelle;GO:0005694//chromosome;GO:0000228//nuclear chromosome;GO:0043233//organelle lumen;GO:0044422//organelle part;GO:0070013//intracellular organelle lumen;GO:0031974//membrane-enclosed lumen	GO:0003676//nucleic acid binding;GO:0003700//transcription factor activity, sequence-specific DNA binding;GO:0003677//DNA binding;GO:0097159//organic cyclic compound binding;GO:0005488//binding;GO:0003674//molecular_function;GO:0043167//ion binding;GO:0001071//nucleic acid binding transcription factor activity;GO:1901363//heterocyclic compound binding	GO:0009987//cellular process;GO:0044237//cellular metabolic process;GO:0008150//biological_process;GO:0009058//biosynthetic process;GO:0006807//nitrogen compound metabolic process;GO:0008152//metabolic process;GO:0034641//cellular nitrogen compound metabolic process
ENSG00000278635	0.672320943	2.820043273	1.746182108	2.230126012	0.000540983	0.030767522	Up	ENSG00000278635	50	3.127082247	11.806962582	1.507253643	238	16.450794173	66.068446635	7.929282791	177	12.45484911	50.104613297	6.003237271	-	-	-	-	-	-	-	-	-
MT1X	-2.20336	0.094934866	-1.05421	2.99790019	0.000565655	0.031539866	Up	ENSG00000187193	4	0.069820667	0.263622743	0.120580291	24	0.462994523	1.859443905	0.799591542	34	0.667726822	2.686198276	1.153164222	MT1X	metallothionein 1X [Source:HGNC Symbol;Acc:HGNC:7405]	Organismal Systems	Digestive system	ko04978//Mineral absorption	K14739	GO:0043226//organelle;GO:0005575//cellular_component;GO:0005634//nucleus;GO:0043227//membrane-bounded organelle;GO:0005737//cytoplasm;GO:0044424//intracellular part;GO:0044464//cell part;GO:0005623//cell;GO:0043231//intracellular membrane-bounded organelle;GO:0005622//intracellular;GO:0043229//intracellular organelle	GO:0003674//molecular_function;GO:0043167//ion binding;GO:0005488//binding	GO:0065007//biological regulation;GO:0042592//homeostatic process;GO:0050896//response to stimulus;GO:0006950//response to stress;GO:0008150//biological_process;GO:0040007//growth;GO:0065008//regulation of biological quality

id	CON_Mean	KD_Mean	AllMean	log2FoldChange	pvalue	qvalue	State	gene_id	XF_1_count	XF_1_FPKM	XF_1_TPM	XF_1_CPM	XF_2_count	XF_2_FPKM	XF_2_TPM	XF_2_CPM	XF_3_count	XF_3_FPKM	XF_3_TPM	XF_3_CPM	Symbol	Description	KEGG_A_class	KEGG_B_class	Pathway	K_ID	GO Component	GO Function	GO Process
NCAM1	3.200743787	4.658571293	3.92965754	1.468691418	0.000571539	0.031764167	Up	ENSG00000149294	304	0.719486704	2.716574724	9.16410215	780	2.040254779	8.193918333	25.986725115	705	1.877302293	7.552199515	23.9111993	NCAM1	neural cell adhesion molecule 1 [Source:HGNC Symbol;Acc:HGNC:7656]	Human Diseases;Environmental Information Processing	Neurodegenerative diseases;Signaling molecules and interaction	ko04514//Cell adhesion molecules (CAMs);ko05020//Prion diseases	K06491	GO:0043227//membrane-bounded organelle;GO:0005622//intracellular;GO:0043229//intracellular organelle;GO:0005886//plasma membrane;GO:0005623//cell;GO:0005794//Golgi apparatus;GO:0071944//cell periphery;GO:0016020//membrane;GO:0044464//cell part;GO:0005737//cytoplasm;GO:0031012//extracellular matrix;GO:0005575//cellular_component;GO:0044421//extracellular region part;GO:0043231//intracellular membrane-bounded organelle;GO:0043226//organelle;GO:0005576//extracellular region;GO:0044424//intracellular part;GO:0005829//cytosol;GO:0012505//endomembrane system;GO:0044444//cytoplasmic part	GO:0003674//molecular_function	GO:0043412//macromolecule modification;GO:0007154//cell communication;GO:0009987//cellular process;GO:0022610//biological adhesion;GO:0008150//biological_process;GO:0023052//signaling;GO:0044237//cellular metabolic process;GO:0006950//response to stress;GO:0007165//signal transduction;GO:0044267//cellular protein metabolic process;GO:0044419//interspecies interaction between organisms;GO:0016043//cellular component organization;GO:0000902//cell morphogenesis;GO:0071704//organic substance metabolic process;GO:0048869//cellular developmental process;GO:0050789//regulation of biological process;GO:0002376//immune system process;GO:0048856//anatomical structure development;GO:0065007//biological regulation;GO:0044238//primary metabolic process;GO:0006464//cellular protein modification process;GO:0050794//regulation of cellular process;GO:0040011//locomotion;GO:0044699//single-organism process;GO:0008152//metabolic [illegible]

id	CON_Mean	KD_Mean	AllMean	log2FoldChange	pvalue	qvalue	State	gene_id	XF_1_count	XF_1_FPKM	XF_1_TPM	XF_1_CPM	XF_2_count	XF_2_FPKM	XF_2_TPM	XF_2_CPM	XF_3_count	XF_3_FPKM	XF_3_TPM	XF_3_CPM	Symbol	Description	KEGG_A_class	KEGG_B_class	Pathway	K_ID	GO Component	GO Function	GO Process
KDELR3	5.247213403	4.024321759	4.635767581	-1.21853	0.00058105	0.032083752	Down	ENSG00000100196	1266	19.9496404482	75.3241009002	38.1636622443	428	7.453937532	29.9359452573	14.2593824499	531	9.414381502	37.8731159558	18.009711813	KDELR3	KDEL endoplasmic reticulum protein retention receptor 3 [Source:HGNC Symbol;Acc:HGNC:6306]	Human Diseases	Infectious diseases	ko05110//Vibrio cholerae infection	K10949	GO:0005623//cell;GO:0097708//intracellular vesicle;GO:0005794//Golgi apparatus;GO:0005575//cellular_component;GO:0044424//intracellular part;GO:0031410//cytoplasmic vesicle;GO:0043229//intracellular organelle;GO:0031982//vesicle;GO:0005622//intracellular;GO:0043227//membrane-bounded organelle;GO:0005737//cytoplasm;GO:0044464//cell part;GO:0043231//intracellular membrane-bounded organelle;GO:0012505//endomembrane system;GO:0044444//cytoplasmic part;GO:0043226//organelle;GO:0005783//endoplasmic reticulum	GO:0003674//molecular_function	GO:0045184//establishment of protein localization;GO:0006950//response to stress;GO:0008104//protein localization;GO:0050789//regulation of biological process;GO:0009987//cellular process;GO:0006810//transport;GO:0044763//single-organism cellular process;GO:0007154//cell communication;GO:0051179//localization;GO:0065007//biological regulation;GO:0016192//vesicle-mediated transport;GO:0051234//establishment of localization;GO:0051716//cellular response to stimulus;GO:0008150//biological_process;GO:0023052//signaling;GO:0033036//macromolecule localization;GO:0007165//signal transduction;GO:0044699//single-organism process;GO:0050896//response to stimulus;GO:0071702//organic substance transport;GO:0050794//regulation of cellular process;GO:0044700//single organism signaling;GO:0015031//protein transport

id	CON_Mean	KD_Mean	AllMean	log2FoldChange	pvalue	qvalue	State	gene_id	XF_1_count	XF_1_FPKM	XF_1_TPM	XF_1_CPM	XF_2_count	XF_2_FPKM	XF_2_TPM	XF_2_CPM	XF_3_count	XF_3_FPKM	XF_3_TPM	XF_3_CPM	Symbol	Description	KEGG_A_class	KEGG_B_class	Pathway	K_ID	GO Component	GO Function	GO Process
PARVA	5.787832895	6.791209654	6.289521274	1.018351486	0.000591664	0.032483986	Up	ENSG00000197702	1843	4.454924969	16.820514542	55.5573692 84	2855	7.627141142	30.631552633	95.118077182	3726	10.133367977	40.765526697	126.373232047	PARVA	parvin alpha [Source:HGNC Symbol;Acc:HGNC:14652]	Cellular Processes	Cellular community	ko04510//Focal adhesion	K06275	GO:0043227//membrane-bounded organelle;GO:0043231//intracellular membrane-bounded organelle;GO:0044424//intracellular part;GO:0043232//intracellular non-membrane-bounded organelle;GO:0005622//intracellular;GO:0016020//membrane;GO:0071944//cell periphery;GO:0005623//cell;GO:0043229//intracellular organelle;GO:0005886//plasma membrane;GO:0005829//cytosol;GO:0043228//non-membrane-bounded organelle;GO:0005575//cellular_component;GO:0044464//cell part;GO:0005634//nucleus;GO:0043226//organelle;GO:0044444//cytoplasmic part;GO:0005856//cytoskeleton;GO:0005737//cytoplasm	GO:0008092//cytoskeletal protein binding;GO:0003674//molecular_function;GO:0005488//binding;GO:0005515//protein binding	GO:0051179//localization;GO:0048646//anatomical structure formation involved in morphogenesis;GO:0040011//locomotion;GO:0022610//biological adhesion;GO:0044763//single-organism cellular process;GO:0000902//cell morphogenesis;GO:0016043//cellular component organization;GO:0030154//cell differentiation;GO:0032989//cellular component morphogenesis;GO:0007010//cytoskeleton organization;GO:0006996//organelle organization;GO:0048856//anatomical structure development;GO:0008150//biological_process;GO:0048869//cellular developmental process;GO:0051674//localization of cell;GO:0044699//single-organism process;GO:0071840//cellular component organization or biogenesis;GO:0006928//movement of cell or subcellular component;GO:0044085//cellular component biogenesis;GO:0007155//cell adhesion;GO:0022607//cellular component assembly;GO:0009653//anatomical structure morphogenesis;GO:0032502//developmental process;GO:0048870//cell motility;GO:0044767//singl [illegible]
SMPD4P2	-2.41729	0.054502553	-1.18139	3.345109285	0.000592106	0.032483986	Up	ENSG00000229147	3	0.055345911	0.208970231	0.090435219	32	0.652461479	2.620366891	1.066122056	24	0.498162958	2.004059798	0.813998274	-	-	-	-	-	-	-	-	-

id	CON_Mean	KD_Mean	AllMean	log2FoldChange	pvalue	qvalue	State	gene_id	XF_1_count	XF_1_FPKM	XF_1_TPM	XF_1_CPM	XF_2_count	XF_2_FPKM	XF_2_TPM	XF_2_CPM	XF_3_count	XF_3_FPKM	XF_3_TPM	XF_3_CPM	Symbol	Description	KEGG_A_class	KEGG_B_class	Pathway	K_ID	GO Component	GO Function	GO Process
KLHL24	5.735315004	4.338786233	5.037050618	-1.32622	0.000595817	0.032582813	Down	ENSG00000114796	1777	6.19280861	23.3822630 04	53.5677944 75	431	1.6600383 17	6.66692147 5	14.3593314 42	817	3.20345178 2	12.8871663 82	27.7098579 13	KLHL24	kelch like family member 24 [Source:HGNC Symbol;Acc:HGNC:25947]	-	-	-	-	GO:0005622//intracellular;GO:0005575//cellular_component;GO:0005737//cytoplasm;GO:0044424//intracellular part;GO:0032991//macromolecular complex;GO:0005623//cell;GO:0044464//cell part	GO:0003674//molecular_function	GO:0007010//cytoskeleton organization;GO:0044763//single-organism cellular process;GO:0051716//cellular response to stimulus;GO:0044700//single organism signaling;GO:0051234//establishment of localization;GO:0006464//cellular protein modification process;GO:0051179//localization;GO:0044699//single-organism process;GO:0023052//signaling;GO:0009987//cellular process;GO:0044260//cellular macromolecule metabolic process;GO:0008152//metabolic process;GO:0050794//regulation of cellular process;GO:0007165//signal transduction;GO:0044237//cellular metabolic process;GO:0043170//macromolecule metabolic process;GO:0036211//protein modification process;GO:0016043//cellular component organization;GO:0055085//transmembrane transport;GO:0006996//organelle organization;GO:0044238//primary metabolic process;GO:0071704//organic substance metabolic process;GO:0007154//cell communication;GO:0006810//transport;GO:0071840//cellular component organization or biogenesis;GO:0050896//response to [illegible]
PAQR5	3.300512301	1.396250781	2.348381541	-1.89749	0.000611594	0.033127037	Down	ENSG00000137819	326	1.570106048	5.928268556	9.827293753	58	0.308730824	1.239901597	1.932346226	96	0.520209793	2.092752011	3.255993096	PAQR5	progestin and adipoQ receptor family member 5 [Source:HGNC Symbol;Acc:HGNC:29645]	-	-	-	-	GO:0005623//cell;GO:0005575//cellular_component;GO:0044464//cell part;GO:0071944//cell periphery;GO:0016020//membrane;GO:0005886//plasma membrane	GO:0005488//binding;GO:0003674//molecular_function;GO:0008289//lipid binding	GO:0048856//anatomical structure development;GO:0044699//single-organism process;GO:0032502//developmental process;GO:0044767//single-organism developmental process;GO:0048869//cellular developmental process;GO:0009987//cellular process;GO:0008150//biological_process;GO:0030154//cell differentiation;GO:0044763//single-organism cellular process;GO:0000003//reproduction

id	CON_Mean	KD_Mean	AllMean	log2FoldChange	pvalue	qvalue	State	gene_id	XF_1_count	XF_1_FPKM	XF_1_TPM	XF_1_CPM	XF_2_count	XF_2_FPKM	XF_2_TPM	XF_2_CPM	XF_3_count	XF_3_FPKM	XF_3_TPM	XF_3_CPM	Symbol	Description	KEGG_A_class	KEGG_B_class	Pathway	K_ID	GO Component	GO Function	GO Process
DLX2	2.821653721	4.441650418	3.631652069	1.658717541	0.000619816	0.033255651	Up	ENSG00000115844	233	2.36412049	8.926238571	7.023801977	530	5.943334417	23.8691742 55	17.6576465 52	767	8.755983903	35.2244482 13	26.0140281 75	DLX2	distal-less homeobox 2 [Source:HGNC Symbol;Acc:HGNC:2915]	-	-	-	-	GO:0043228//non-membrane-bounded organelle;GO:0031974//membrane-enclosed lumen;GO:0044446//intracellular organelle part;GO:0044424//intracellular part;GO:0000228//nuclear chromosome;GO:0031981//nuclear lumen;GO:0005575//cellular_component;GO:0044428//nuclear part;GO:0005623//cell;GO:0043226//organelle;GO:0043227//membrane-bounded organelle;GO:0043231//intracellular membrane-bounded organelle;GO:0005634//nucleus;GO:0043229//intracellular organelle;GO:0070013//intracellular organelle lumen;GO:0005694//chromosome;GO:0005622//intracellular;GO:0044422//organelle part;GO:0044464//cell part;GO:0043232//intracellular non-membrane-bounded organelle;GO:0043233//organelle lumen	GO:0003700//transcription factor activity, sequence-specific DNA binding;GO:0005488//binding;GO:0001071//nucleic acid binding transcription factor activity;GO:0003676//nucleic acid binding;GO:0003723//RNA binding;GO:1901363//heterocyclic compound binding;GO:0003677//DNA binding;GO:0097159//organic cyclic compound binding;GO:0003674//molecular_function	GO:0044767//single-organism developmental process;GO:0008152//metabolic process;GO:0044707//single-multicellular organism process;GO:0007154//cell communication;GO:0007275//multicellular organism development;GO:0065007//biological regulation;GO:0050896//response to stimulus;GO:0009987//cellular process;GO:0007165//signal transduction;GO:0032502//developmental process;GO:0044700//single organism signaling;GO:0006807//nitrogen compound metabolic process;GO:0050789//regulation of biological process;GO:0044763//single-organism cellular process;GO:0050794//regulation of cellular process;GO:0030154//cell differentiation;GO:0023052//signaling;GO:0044699//single-organism process;GO:0009790//embryo development;GO:0051716//cellular response to stimulus;GO:0044237//cellular metabolic process;GO:0009058//biosynthetic process;GO:0034641//cellular nitrogen compound metabolic process;GO:0008150//biological_process;GO:0032501//multicellular organismal process;GO:0048869//cellu[illegible]
ENSG00000225177	6.186118378	4.508921673	5.347520025	-1.5181	0.00062889	0.033631134	Down	ENSG00000225177	2430	27.7576836 13	104.805024586	73.2525270 54	413	5.213958994	20.9399113 46	13.7596377 85	1080	13.8802282 43	55.8387711 02	36.6299223 32	-	-	-	-	-	-	-	-	-

id	CON_Mean	KD_Mean	AllMean	log2FoldChange	pvalue	qvalue	State	gene_id	XF_1_count	XF_1_FPKM	XF_1_TPM	XF_1_CPM	XF_2_count	XF_2_FPKM	XF_2_TPM	XF_2_CPM	XF_3_count	XF_3_FPKM	XF_3_TPM	XF_3_CPM	Symbol	Description	KEGG_A_class	KEGG_B_class	Pathway	K_ID	GO Component	GO Function	GO Process
PFAS	6.370121489	7.35918159	6.86465154	1.003226508	0.000630756	0.033631134	Up	ENSG00000178921	2761	12.862084094	48.563527796	83.230546171	5572	28.687761242	115.213636666	185.638502998	4198	22.003069815	88.5161509	142.38186477	PFAS	phosphoribosylformylglycinamidine synthase [Source:HGNC Symbol;Acc:HGNC:8863]	Metabolism	Nucleotide metabolism	ko00230//Purine metabolism	K01952	GO:0005576//extracellular region;GO:0044444//cytoplasmic part;GO:0044424//intracellular part;GO:0005623//cell;GO:0044464//cell part;GO:0044421//extracellular region part;GO:0043226//organelle;GO:0005575//cellular_component;GO:0005622//intracellular;GO:0005737//cytoplasm;GO:0005615//extracellular space;GO:0005829//cytosol	GO:0016874//ligase activity;GO:0005488//binding;GO:0043167//ion binding;GO:0003674//molecular_function;GO:0003824//catalytic activity	GO:0008152//metabolic process;GO:0006082//organic acid metabolic process;GO:0043436//oxoacid metabolic process;GO:0044237//cellular metabolic process;GO:0009058//biosynthetic process;GO:0044763//single-organism cellular process;GO:0009987//cellular process;GO:0006520//cellular amino acid metabolic process;GO:0008150//biological_process;GO:0048856//anatomical structure development;GO:0032502//developmental process;GO:0044238//primary metabolic process;GO:0044710//single-organism metabolic process;GO:0044281//small molecule metabolic process;GO:1901564//organonitrogen compound metabolic process;GO:0019752//carboxylic acid metabolic process;GO:0034641//cellular nitrogen compound metabolic process;GO:0044699//single-organism process;GO:0071704//organic substance metabolic process;GO:0006807//nitrogen compound metabolic process
FOXRED2	5.030541373	6.045120977	5.537831175	1.023015693	0.00065531	0.034812215	Up	ENSG00000100350	1089	5.948176182	22.458601361	32.827984346	2146	12.954667581	52.027565037	71.496810379	1760	10.815946143	43.511470444	59.693206764	FOXRED2	FAD dependent oxidoreductase domain containing 2 [Source:HGNC Symbol;Acc:HGNC:26264]	-	-	-	-	GO:0043226//organelle;GO:0005737//cytoplasm;GO:0005623//cell;GO:0005622//intracellular;GO:0043227//membrane-bounded organelle;GO:0012505//endomembrane system;GO:0044424//intracellular part;GO:0043229//intracellular organelle;GO:0044444//cytoplasmic part;GO:0005783//endoplasmic reticulum;GO:0043231//intracellular membrane-bounded organelle;GO:0005575//cellular_component;GO:0044464//cell part	GO:0005488//binding;GO:0043167//ion binding;GO:0003824//catalytic activity;GO:0003674//molecular_function;GO:0016491//oxidoreductase activity	GO:0009056//catabolic process;GO:0006950//response to stress;GO:0008150//biological_process;GO:0008152//metabolic process;GO:0050896//response to stimulus

id	CON_Mean	KD_Mean	AllMean	log2FoldChange	pvalue	qvalue	State	gene_id	XF_1_count	XF_1_FPKM	XF_1_TPM	XF_1_CPM	XF_2_count	XF_2_FPKM	XF_2_TPM	XF_2_CPM	XF_3_count	XF_3_FPKM	XF_3_TPM	XF_3_CPM	Symbol	Description	KEGG_A_class	KEGG_B_class	Pathway	K_ID	GO Component	GO Function	GO Process
WIPI1	4.887646	3.476514992	4.182080582	-1.40092	0.000663109	0.034812215	Down	ENSG00000070540	986	8.734364338	32.978445966	29.723041842	279	2.731487416	10.969995045	9.295251676	379	3.777369795	15.195981193	12.854389411	WIPI1	WD repeat domain, phosphoinositide interacting 1 [Source:HGNC Symbol;Acc:HGNC:25471]	-	-	-	-	GO:0005623//cell;GO:0005773//vacuole;GO:0043227//membrane-bounded organelle;GO:0043231//intracellular membrane-bounded organelle;GO:0005737//cytoplasm;GO:0005768//endosome;GO:0005622//intracellular;GO:0044444//cytoplasmic part;GO:0031410//cytoplasmic vesicle;GO:0012505//endomembrane system;GO:0005829//cytosol;GO:0043228//non-membrane-bounded organelle;GO:0043226//organelle;GO:0005856//cytoskeleton;GO:0044424//intracellular part;GO:0097708//intracellular vesicle;GO:0005575//cellular_component;GO:0043232//intracellular non-membrane-bounded organelle;GO:0044464//cell part;GO:0005794//Golgi apparatus;GO:0031982//vesicle;GO:0043229//intracellular organelle	GO:0008134//transcription factor binding;GO:0005515//protein binding;GO:0008289//lipid binding;GO:0003674//molecular_function;GO:0005488//binding;GO:0043167//ion binding	GO:0050789//regulation of biological process;GO:0009056//catabolic process;GO:0065007//biological regulation;GO:0051234//establishment of localization;GO:0051716//cellular response to stimulus;GO:0044260//cellular macromolecule metabolic process;GO:0044700//single organism signaling;GO:0044085//cellular component biogenesis;GO:0043170//macromolecule metabolic process;GO:0007154//cell communication;GO:0071704//organic substance metabolic process;GO:0006914//autophagy;GO:0044763//single-organism cellular process;GO:0006464//cellular protein modification process;GO:0006810//transport;GO:0050794//regulation of cellular process;GO:0044699//single-organism process;GO:0016043//cellular component organization;GO:0043412//macromolecule modification;GO:0009058//biosynthetic process;GO:0008150//biological_process;GO:0023052//signaling;GO:0044237//cellular metabolic process;GO:0044267//cellular protein metabolic process;GO:0050896//response to stimulus;GO:0007005//mito[illegible]

id	CON_Mean	KD_Mean	AllMean	log2FoldChange	pvalue	qvalue	State	gene_id	XF_1_count	XF_1_FPKM	XF_1_TPM	XF_1_CPM	XF_2_count	XF_2_FPKM	XF_2_TPM	XF_2_CPM	XF_3_count	XF_3_FPKM	XF_3_TPM	XF_3_CPM	Symbol	Description	KEGG_A_class	KEGG_B_class	Pathway	K_ID	GO Component	GO Function	GO Process
SMARCA1	7.334654222	6.293577028	6.814115625	-1.00839	0.00066686	0.034901751	Down	ENSG00000102038	5391	37.247785422	140.636917743	162.512087797	1869	14.271875162	57.317635405	62.268191332	2853	22.178327946	89.221196939	96.764044828	SMARCA1	SWI/SNF related, matrix associated, actin dependent regulator of chromatin, subfamily a, member 1 [Source:HGNC Symbol;Acc:HGNC:11097]	-	-	-	-	GO:0005694//chromosome;GO:0043229//intracellular organelle;GO:0044422//organelle part;GO:0043228//non-membrane-bounded organelle;GO:0043226//organelle;GO:0005654//nucleoplasm;GO:0070013//intracellular organelle lumen;GO:0044446//intracellular organelle part;GO:0005622//intracellular;GO:0032991//macromolecular complex;GO:0043233//organelle lumen;GO:0000228//nuclear chromosome;GO:0005623//cell;GO:0044428//nuclear part;GO:0044424//intracellular part;GO:0043231//intracellular membrane-bounded organelle;GO:0031974//membrane-enclosed lumen;GO:0005575//cellular_component;GO:0044464//cell part;GO:0031981//nuclear lumen;GO:0005634//nucleus;GO:0043232//intracellular non-membrane-bounded organelle;GO:0043227//membrane-bounded organelle	GO:0003824//catalytic activity;GO:0016818//hydrolase activity, acting on acid anhydrides, in phosphorus-containing anhydrides;GO:0003676//nucleic acid binding;GO:0097159//organic cyclic compound binding;GO:0003674//molecular_function;GO:0005488//binding;GO:0043167//ion binding;GO:1901363//heterocyclic compound binding;GO:0004386//helicase activity;GO:0016817//hydrolase activity, acting on acid anhydrides;GO:0017111//nucleoside-triphosphatase activity;GO:0016787//hydrolase activity;GO:0005515//protein binding;GO:0016887//ATPase activity;GO:0016462//pyrophosphatase activity;GO:0008134//transcription factor binding;GO:0003677//DNA binding	GO:0043170//macromolecule metabolic process;GO:0044767//single-organism developmental process;GO:0008283//cell proliferation;GO:0022607//cellular component assembly;GO:0006139//nucleobase-containing compound metabolic process;GO:0048856//anatomical structure development;GO:0051276//chromosome organization;GO:0044699//single-organism process;GO:0034641//cellular nitrogen compound metabolic process;GO:0032502//developmental process;GO:0008152//metabolic process;GO:0044085//cellular component biogenesis;GO:0006259//DNA metabolic process;GO:0006807//nitrogen compound metabolic process;GO:0006996//organelle organization;GO:0048869//cellular developmental process;GO:0016043//cellular component organization;GO:0090304//nucleic acid metabolic process;GO:0046483//heterocycle metabolic process;GO:0071704//organic substance metabolic process;GO:0044260//cellular macromolecule metabolic process;GO:0071840//cellular component organization or biogenesis;GO:0009058//bi[illegible]

id	CON_Mean	KD_Mean	AllMean	log2FoldChange	pvalue	qvalue	State	gene_id	XF_1_count	XF_1_FPKM	XF_1_TPM	XF_1_CPM	XF_2_count	XF_2_FPKM	XF_2_TPM	XF_2_CPM	XF_3_count	XF_3_FPKM	XF_3_TPM	XF_3_CPM	Symbol	Description	KEGG_A_class	KEGG_B_class	Pathway	K_ID	GO Component	GO Function	GO Process
FHL1	4.737142163	3.272997954	4.005070058	-1.45441	0.000669371	0.034926011	Down	ENSG00000022267	888	4.56650029	17.241790845	26.768824701	241	1.369708587	5.500920971	8.029231734	330	1.909327238	7.68103267	11.192476268	FHL1	four and a half LIM domains 1 [Source:HGNC Symbol;Acc:HGNC:3702]	-	-	-	-	GO:0005829//cytosol;GO:0005575//cellular_component;GO:0016020//membrane;GO:0005737//cytoplasm;GO:0005623//cell;GO:0005634//nucleus;GO:0044444//cytoplasmic part;GO:0043226//organelle;GO:0043229//intracellular organelle;GO:0043231//intracellular membrane-bounded organelle;GO:0044424//intracellular part;GO:0043227//membrane-bounded organelle;GO:0005886//plasma membrane;GO:0005622//intracellular;GO:0044464//cell part;GO:0071944//cell periphery	GO:0005488//binding;GO:0043167//ion binding;GO:0003674//molecular_function	GO:0032502//developmental process;GO:0055085//transmembrane transport;GO:0000278//mitotic cell cycle;GO:0065008//regulation of biological quality;GO:0044699//single-organism process;GO:0040007//growth;GO:0065007//biological regulation;GO:0006810//transport;GO:0044767//single-organism developmental process;GO:0009987//cellular process;GO:0048869//cellular developmental process;GO:0051234//establishment of localization;GO:0007049//cell cycle;GO:0042592//homeostatic process;GO:0051179//localization;GO:0030154//cell differentiation;GO:0044763//single-organism cellular process;GO:0008150//biological_process;GO:0048856//anatomical structure development
ASMTL-AS1	0.530085499	2.635669418	1.582877458	2.186394136	0.000678672	0.035303372	Up	ENSG00000236017	45	1.016126052	3.836599527	1.356528279	165	4.117746705	16.537385708	5.497191851	197	5.004920724	20.134295906	6.681569166	-	-	-	-	-	-	-	-	-

id	CON_Mean	KD_Mean	AllMean	log2FoldChange	pvalue	qvalue	State	gene_id	XF_1_count	XF_1_FPKM	XF_1_TPM	XF_1_CPM	XF_2_count	XF_2_FPKM	XF_2_TPM	XF_2_CPM	XF_3_count	XF_3_FPKM	XF_3_TPM	XF_3_CPM	Symbol	Description	KEGG_A_class	KEGG_B_class	Pathway	K_ID	GO Component	GO Function	GO Process
TUBB2B	3.713076298	1.725020389	2.719048343	-1.92177	0.000682242	0.035323732	Down	ENSG00000137285	435	3.152189109	11.9017588 68	13.1131066 95	65	0.52056741	2.090663818	2.165560426	137	1.116964779	4.493437683	4.646573481	TUBB2B	tubulin beta 2B class IIb [Source:HGNC Symbol;Acc:HGNC:30829]	Cellular Processes;Human Diseases	Transport and catabolism;Infectious diseases;Cellular community	ko04145//Phagosome;ko04540//Gap junction;ko05130//Pathogenic Escherichia coli infection	K07375	GO:0043231//intracellular membrane-bounded organelle;GO:0005634//nucleus;GO:0005623//cell;GO:0005622//intracellular;GO:0043227//membrane-bounded organelle;GO:0005575//cellular_component;GO:0044424//intracellular part;GO:0043229//intracellular organelle;GO:0043226//organelle;GO:0043228//non-membrane-bounded organelle;GO:0005737//cytoplasm;GO:0043232//intracellular non-membrane-bounded organelle;GO:0044464//cell part;GO:0005856//cytoskeleton	GO:0016817//hydrolase activity, acting on acid anhydrides;GO:0017111//nucleoside-triphosphatase activity;GO:0005198//structural molecule activity;GO:0016787//hydrolase activity;GO:0005488//binding;GO:0003824//catalytic activity;GO:0003924//GTPase activity;GO:0016462//pyrophosphatase activity;GO:0043167//ion binding;GO:0016818//hydrolase activity, acting on acid anhydrides, in phosphorus-containing anhydrides;GO:0003674//molecular_function	GO:0000278//mitotic cell cycle;GO:0048869//cellular developmental process;GO:0009653//anatomical structure morphogenesis;GO:0032502//developmental process;GO:0032989//cellular component morphogenesis;GO:0051179//localization;GO:0071840//cellular component organization or biogenesis;GO:0044767//single-organism developmental process;GO:0000902//cell morphogenesis;GO:0007010//cytoskeleton organization;GO:0006928//movement of cell or subcellular component;GO:0030154//cell differentiation;GO:0048856//anatomical structure development;GO:0044700//single organism signaling;GO:0051674//localization of cell;GO:0009790//embryo development;GO:0044707//single-multicellular organism process;GO:0007275//multicellular organism development;GO:0040011//locomotion;GO:0016043//cellular component organization;GO:0044699//single-organism process;GO:0008150//biological_process;GO:0044763//single-organism cellular process;GO:0032501//multicellular organismal process;GO:0006996//organelle [illegible]

id	CON_Mean	KD_Mean	AllMean	log2FoldChange	pvalue	qvalue	State	gene_id	XF_1_count	XF_1_FPKM	XF_1_TPM	XF_1_CPM	XF_2_count	XF_2_FPKM	XF_2_TPM	XF_2_CPM	XF_3_count	XF_3_FPKM	XF_3_TPM	XF_3_CPM	Symbol	Description	KEGG_A_class	KEGG_B_class	Pathway	K_ID	GO Component	GO Function	GO Process
SENP7	5.314934228	4.129371888	4.722153058	-1.18059	0.000683585	0.035323732	Down	ENSG00000138468	1327	6.25430139	23.614442042	40.002511687	460	2.396107654	9.623067981	15.325504555	572	3.033191401	12.202225883	19.400292198	SENP7	SUMO specific peptidase 7 [Source:HGNC Symbol;Acc:HGNC:30402]	-	-	-	-	GO:0005575//cellular_component;GO:0005634//nucleus;GO:0043229//intracellular organelle;GO:0005737//cytoplasm;GO:0043231//intracellular membrane-bounded organelle;GO:0005622//intracellular;GO:0044424//intracellular part;GO:0043227//membrane-bounded organelle;GO:0043226//organelle;GO:0044464//cell part;GO:0005623//cell	GO:0003824//catalytic activity;GO:0008233//peptidase activity;GO:0016787//hydrolase activity;GO:0003674//molecular_function	GO:0008150//biological_process;GO:0043412//macromolecule modification;GO:0019538//protein metabolic process;GO:0008152//metabolic process;GO:0009987//cellular process;GO:0044267//cellular protein metabolic process;GO:0036211//protein modification process;GO:0071704//organic substance metabolic process;GO:0044260//cellular macromolecule metabolic process;GO:0043170//macromolecule metabolic process;GO:0044237//cellular metabolic process;GO:0006464//cellular protein modification process;GO:0044238//primary metabolic process
ITGB2-AS1	-1.22905	1.222944025	-0.00305	2.833450522	0.000686409	0.035323732	Up	ENSG00000227039	11	0.136011403	0.513539915	0.331595801	94	1.284550262	5.158914491	3.131733539	46	0.63993575	2.574397571	1.560163359	-	-	-	-	-	-	-	-	-
TAF1C	5.28645739	6.32403604	5.805246715	1.05843705	0.000687345	0.035323732	Up	ENSG00000103168	1301	7.03222876	26.551671903	39.218739793	2014	12.03138908	48.319563117	67.099056898	2762	16.797137297	67.573204727	93.677634706	TAF1C	TATA-box binding protein associated factor, RNA polymerase I subunit C [Source:HGNC Symbol;Acc:HGNC:11534]	-	-	-	-	GO:0043232//intracellular non-membrane-bounded organelle;GO:0043233//organelle lumen;GO:0043229//intracellular organelle;GO:0005623//cell;GO:0005622//intracellular;GO:0043228//non-membrane-bounded organelle;GO:0043226//organelle;GO:0044424//intracellular part;GO:0044428//nuclear part;GO:0043231//intracellular membrane-bounded organelle;GO:0043227//membrane-bounded organelle;GO:0070013//intracellular organelle lumen;GO:0044464//cell part;GO:0044422//organelle part;GO:0031981//nuclear lumen;GO:0005575//cellular_component;GO:0031974//membrane-enclosed lumen;GO:0005654//nucleoplasm;GO:0044446//intracellular organelle part;GO:0005634//nucleus;GO:0005730//nucleolus	GO:0003676//nucleic acid binding;GO:0003677//DNA binding;GO:0005488//binding;GO:0003674//molecular_function;GO:1901363//heterocyclic compound binding;GO:0097159//organic cyclic compound binding	GO:0008152//metabolic process;GO:0043933//macromolecular complex subunit organization;GO:0034641//cellular nitrogen compound metabolic process;GO:0016043//cellular component organization;GO:0006807//nitrogen compound metabolic process;GO:0044237//cellular metabolic process;GO:0009058//biosynthetic process;GO:0071840//cellular component organization or biogenesis;GO:0065003//macromolecular complex assembly;GO:0008150//biological_process;GO:0009987//cellular process;GO:0022607//cellular component assembly;GO:0044085//cellular component biogenesis

id	CON_Mean	KD_Mean	AllMean	log2FoldChange	pvalue	qvalue	State	gene_id	XF_1_count	XF_1_FPKM	XF_1_TPM	XF_1_CPM	XF_2_count	XF_2_FPKM	XF_2_TPM	XF_2_CPM	XF_3_count	XF_3_FPKM	XF_3_TPM	XF_3_CPM	Symbol	Description	KEGG_A_class	KEGG_B_class	Pathway	K_ID	GO Component	GO Function	GO Process
TMSB4X	7.470594598	6.382704782	6.92664969	-1.04525	0.0006896	0.035333223	Down	ENSG00000205542	5924	104.861662732	395.92745901	178.579411633	3161	61.839617935	248.355638912	105.312869343	1909	38.019248022	152.947635346	64.746779382	TMSB4X	thymosin beta 4 X-linked [Source:HGNC Symbol;Acc:HGNC:11881]	Cellular Processes	Cell motility	ko04810//Regulation of actin cytoskeleton	K05764	GO:0031410//cytoplasmic vesicle;GO:0005634//nucleus;GO:0097708//intracellular vesicle;GO:0044444//cytoplasmic part;GO:0043228//non-membrane-bounded organelle;GO:0005856//cytoskeleton;GO:0005575//cellular_component;GO:0043226//organelle;GO:0043231//intracellular membrane-bounded organelle;GO:0043232//intracellular non-membrane-bounded organelle;GO:0005829//cytosol;GO:0005576//extracellular region;GO:0005737//cytoplasm;GO:0043227//membrane-bounded organelle;GO:0031982//vesicle;GO:0005623//cell;GO:0005622//intracellular;GO:0043229//intracellular organelle;GO:0044424//intracellular part;GO:0044464//cell part	GO:0005488//binding;GO:0097159//organic cyclic compound binding;GO:0008092//cytoskeletal protein binding;GO:0005515//protein binding;GO:0019899//enzyme binding;GO:0003723//RNA binding;GO:0003676//nucleic acid binding;GO:0003674//molecular_function;GO:1901363//heterocyclic compound binding	GO:0065007//biological regulation;GO:0023052//signaling;GO:0048870//cell motility;GO:0007165//signal transduction;GO:0006810//transport;GO:0007154//cell communication;GO:0044237//cellular metabolic process;GO:0051674//localization of cell;GO:0044085//cellular component biogenesis;GO:0043933//macromolecular complex subunit organization;GO:0050794//regulation of cellular process;GO:0006950//response to stress;GO:0051716//cellular response to stimulus;GO:0050896//response to stimulus;GO:0006928//movement of cell or subcellular component;GO:0033036//macromolecule localization;GO:0044710//single-organism metabolic process;GO:0008104//protein localization;GO:0044700//single organism signaling;GO:0016043//cellular component organization;GO:0006807//nitrogen compound metabolic process;GO:0044699//single-organism process;GO:0015031//protein transport;GO:0022607//cellular component assembly;GO:0051234//establishment of localization;GO:0055085//tr[illegible]

id	CON_Mean	KD_Mean	AllMean	log2FoldChange	pvalue	qvalue	State	gene_id	XF_1_count	XF_1_FPKM	XF_1_TPM	XF_1_CPM	XF_2_count	XF_2_FPKM	XF_2_TPM	XF_2_CPM	XF_3_count	XF_3_FPKM	XF_3_TPM	XF_3_CPM	Symbol	Description	KEGG_A_class	KEGG_B_class	Pathway	K_ID	GO Component	GO Function	GO Process
NTN4	4.248640444	2.584942268	3.416791356	-1.64886	0.000720215	0.036355925	Down	ENSG00000074527	632	4.364647434	16.479652553	19.051686049	142	1.083829696	4.352795597	4.730916623	213	1.655036582	6.658046775	7.224234682	NTN4	netrin 4 [Source:HGNC Symbol;Acc:HGNC:13658]	Organismal Systems	Development	ko04360//Axon guidance	K06845	GO:0005576//extracellular region;GO:0005575//cellular_component;GO:0032991//macromolecular complex;GO:0016020//membrane;GO:0005623//cell;GO:0071944//cell periphery;GO:0005886//plasma membrane;GO:0031012//extracellular matrix;GO:0044421//extracellular region part;GO:0044464//cell part	GO:0003674//molecular_function	GO:0022610//biological adhesion;GO:0044763//single-organism cellular process;GO:0048870//cell motility;GO:0048869//cellular developmental process;GO:0009987//cellular process;GO:0030154//cell differentiation;GO:0040011//locomotion;GO:0007155//cell adhesion;GO:0030198//extracellular matrix organization;GO:0016043//cellular component organization;GO:0071840//cellular component organization or biogenesis;GO:0051674//localization of cell;GO:0000902//cell morphogenesis;GO:0051179//localization;GO:0032502//developmental process;GO:0021700//developmental maturation;GO:0048856//anatomical structure development;GO:0044699//single-organism process;GO:0043062//extracellular structure organization;GO:0044767//single-organism developmental process;GO:0009653//anatomical structure morphogenesis;GO:0006928//movement of cell or subcellular component;GO:0032989//cellular component morphogenesis;GO:0044085//cellular component biogenesis;GO:0022607//cellular component assembly;GO:0008150//bio[illegible]

id	CON_Mean	KD_Mean	AllMean	log2FoldChange	pvalue	qvalue	State	gene_id	XF_1_count	XF_1_FPKM	XF_1_TPM	XF_1_CPM	XF_2_count	XF_2_FPKM	XF_2_TPM	XF_2_CPM	XF_3_count	XF_3_FPKM	XF_3_TPM	XF_3_CPM	Symbol	Description	KEGG_A_class	KEGG_B_class	Pathway	K_ID	GO Component	GO Function	GO Process
CYSTM1	5.426547362	4.331105407	4.878826385	-1.09887	0.000744207	0.037351979	Down	ENSG00000120306	1434	23.278424601	87.8926317	43.2280344 84	602	10.8004422 07	43.3759265 41	20.0564211 78	579	10.5749641 15	42.5420238 27	19.6377083 62	CYSTM1	cysteine rich transmembrane module containing 1 [Source:HGNC Symbol;Acc:HGNC:30239]	-	-	-	-	GO:0044421//extracellular region part;GO:0031410//cytoplasmic vesicle;GO:0044444//cytoplasmic part;GO:0005886//plasma membrane;GO:0043227//membrane-bounded organelle;GO:0005615//extracellular space;GO:0005575//cellular_component;GO:0005623//cell;GO:0044424//intracellular part;GO:0005576//extracellular region;GO:0005737//cytoplasm;GO:0097708//intracellular vesicle;GO:0016020//membrane;GO:0043226//organelle;GO:0044464//cell part;GO:0071944//cell periphery;GO:0031982//vesicle;GO:0005622//intracellular	GO:0003674//molecular_function	GO:0008150//biological_process;GO:0002376//immune system process;GO:0051179//localization;GO:0016192//vesicle-mediated transport;GO:0051234//establishment of localization;GO:0006810//transport

id	CON_Mean	KD_Mean	AllMean	log2FoldChange	pvalue	qvalue	State	gene_id	XF_1_count	XF_1_FPKM	XF_1_TPM	XF_1_CPM	XF_2_count	XF_2_FPKM	XF_2_TPM	XF_2_CPM	XF_3_count	XF_3_FPKM	XF_3_TPM	XF_3_CPM	Symbol	Description	KEGG_A_class	KEGG_B_class	Pathway	K_ID	GO Component	GO Function	GO Process
CDC42EP5	3.489177653	0.992521727	2.24084969	-2.28594	0.000744325	0.037351979	Down	ENSG00000167617	372	11.993547705	45.284184361	11.213967105	29	1.033340228	4.150023579	0.966173113	105	3.808815453	15.322483936	3.561242449	CDC42EP5	CDC42 effector protein 5 [Source:HGNC Symbol;Acc:HGNC:17408]	-	-	-	-	GO:0044444//cytoplasmic part;GO:0005829//cytosol;GO:0005575//cellular_component;GO:0043226//organelle;GO:0043228//non-membrane-bounded organelle;GO:0016020//membrane;GO:0005737//cytoplasm;GO:0005886//plasma membrane;GO:0043232//intracellular non-membrane-bounded organelle;GO:0005623//cell;GO:0044424//intracellular part;GO:0044464//cell part;GO:0005622//intracellular;GO:0005856//cytoskeleton;GO:0071944//cell periphery;GO:0043229//intracellular organelle	GO:0005515//protein binding;GO:0003674//molecular_function;GO:0005488//binding;GO:0019899//enzyme binding	GO:0007165//signal transduction;GO:0050896//response to stimulus;GO:0006996//organelle organization;GO:0071840//cellular component organization or biogenesis;GO:0006950//response to stress;GO:0048869//cellular developmental process;GO:0043933//macromolecular complex subunit organization;GO:0032502//developmental process;GO:0043412//macromolecule modification;GO:0044085//cellular component biogenesis;GO:0043170//macromolecule metabolic process;GO:0016043//cellular component organization;GO:0065003//macromolecular complex assembly;GO:0050789//regulation of biological process;GO:0048856//anatomical structure development;GO:0065007//biological regulation;GO:0071704//organic substance metabolic process;GO:0008152//metabolic process;GO:0044260//cellular macromolecule metabolic process;GO:0044700//single organism signaling;GO:0044238//primary metabolic process;GO:0044267//cellular protein metabolic process;GO:0044767//single-organism developmental [illegible]

id	CON_Mean	KD_Mean	AllMean	log2FoldChange	pvalue	qvalue	State	gene_id	XF_1_count	XF_1_FPKM	XF_1_TPM	XF_1_CPM	XF_2_count	XF_2_FPKM	XF_2_TPM	XF_2_CPM	XF_3_count	XF_3_FPKM	XF_3_TPM	XF_3_CPM	Symbol	Description	KEGG_A_class	KEGG_B_class	Pathway	K_ID	GO Component	GO Function	GO Process
ST8SIA4	5.102674658	3.892571955	4.497623307	-1.21504	0.000753052	0.037679121	Down	ENSG00000113532	1145	4.418344653	16.682397797	34.516108427	445	1.89781872	7.621877309	14.825759841	425	1.845180846	7.422978146	14.41455277	ST8SIA4	ST8 alpha-N-acetyl-neuraminide alpha-2,8-sialyltransferase 4 [Source:HGNC Symbol;Acc:HGNC:10871]	-	-	-	-	GO:0005575//cellular_component;GO:0043231//intracellular membrane-bounded organelle;GO:0043229//intracellular organelle;GO:0044464//cell part;GO:0043227//membrane-bounded organelle;GO:0043226//organelle;GO:0044444//cytoplasmic part;GO:0005623//cell;GO:0012505//endomembrane system;GO:0044424//intracellular part;GO:0005737//cytoplasm;GO:0005622//intracellular;GO:0005794//Golgi apparatus	GO:0003824//catalytic activity;GO:0016757//transferase activity, transferring glycosyl groups;GO:0016740//transferase activity;GO:0003674//molecular_function;GO:0005488//binding; GO:0043167//ion binding	GO:0044267//cellular protein metabolic process;GO:0043170//macromolecule metabolic process;GO:0034641//cellular nitrogen compound metabolic process;GO:0009058//biosynthetic process;GO:0019538//protein metabolic process;GO:0008152//metabolic process;GO:0008150//biological_process;GO:0006807//nitrogen compound metabolic process;GO:0044237//cellular metabolic process;GO:0044260//cellular macromolecule metabolic process;GO:0044710//single-organism metabolic process;GO:0006464//cellular protein modification process;GO:0071704//organic substance metabolic process;GO:0009987//cellular process;GO:0048856//anatomical structure development;GO:0044699//single-organism process;GO:0005975//carbohydrate metabolic process;GO:0032502//developmental process;GO:0006629//lipid metabolic process;GO:0043412//macromolecule modification;GO:0044238//primary metabolic process;GO:0036211//protein modification process

id	CON_Mean	KD_Mean	AllMean	log2FoldChange	pvalue	qvalue	State	gene_id	XF_1_count	XF_1_FPKM	XF_1_TPM	XF_1_CPM	XF_2_count	XF_2_FPKM	XF_2_TPM	XF_2_CPM	XF_3_count	XF_3_FPKM	XF_3_TPM	XF_3_CPM	Symbol	Description	KEGG_A_class	KEGG_B_class	Pathway	K_ID	GO Component	GO Function	GO Process
COG5	5.833215142	4.801730236	5.317472689	-1.02659	0.000765405	0.037950758	Down	ENSG00000164597	1902	6.828144407	25.781108121	57.33592858 3	744	2.951927808	11.85531122 6	24.78733780 2	902	3.643297424	14.65662144 3	30.59276846 6	COG5	component of oligomeric golgi complex 5 [Source:HGNC Symbol;Acc:HGNC:14857]	-	-	-	-	GO:0005623//cell;GO:0043226//organelle;GO:0032991//macromolecular complex;GO:0005654//nucleoplasm;GO:0043233//organelle lumen;GO:0005829//cytosol;GO:0044444//cytoplasmic part;GO:0044464//cell part;GO:0044422//organelle part;GO:0043231//intracellular membrane-bounded organelle;GO:0043227//membrane-bounded organelle;GO:0005575//cellular_component;GO:0031974//membrane-enclosed lumen;GO:0005737//cytoplasm;GO:0044424//intracellular part;GO:0012505//endomembrane system;GO:0005794//Golgi apparatus;GO:0044446//intracellular organelle part;GO:0070013//intracellular organelle lumen;GO:0044428//nuclear part;GO:0005622//intracellular;GO:0031981//nuclear lumen;GO:0043229//intracellular organelle;GO:0005634//nucleus	GO:0003674//molecular_function	GO:0051234//establishment of localization;GO:0015031//protein transport;GO:0006810//transport;GO:0045184//establishment of protein localization;GO:0033036//macromolecule localization;GO:0051179//localization;GO:0016192//vesicle-mediated transport;GO:0071702//organic substance transport;GO:0008104//protein localization;GO:0008150//biological_process
ARL4C	5.705842087	4.667537392	5.186689739	-1.03804	0.000765603	0.037950758	Down	ENSG00000188042	1741	12.987520874	49.037140977	52.482571852	800	6.595657362	26.488984778	26.6530514	696	5.841610974	23.50021717	23.605949948	ARL4C	ADP ribosylation factor like GTPase 4C [Source:HGNC Symbol;Acc:HGNC:698]	-	-	-	-	GO:0044444//cytoplasmic part;GO:0005575//cellular_component;GO:0005634//nucleus;GO:0005623//cell;GO:0005737//cytoplasm;GO:0043231//intracellular membrane-bounded organelle;GO:0016020//membrane;GO:0044464//cell part;GO:0005829//cytosol;GO:0044424//intracellular part;GO:0071944//cell periphery;GO:0043227//membrane-bounded organelle;GO:0005622//intracellular;GO:0005886//plasma membrane;GO:0043229//intracellular organelle;GO:0043226//organelle	GO:0005515//protein binding;GO:0017111//nucleoside-triphosphatase activity;GO:0016787//hydrolase activity;GO:0016818//hydrolase activity, acting on acid anhydrides, in phosphorus-containing anhydrides;GO:0008092//cytoskeletal protein binding;GO:0003674//molecular_function;GO:0016817//hydrolase activity, acting on acid anhydrides;GO:0043167//ion binding;GO:0003824//catalytic activity;GO:0016462//pyrophosphatase activity;GO:0005488//binding;GO:0003924//GTPase activity	GO:0016192//vesicle-mediated transport;GO:0006810//transport;GO:0015031//protein transport;GO:0045184//establishment of protein localization;GO:0051234//establishment of localization;GO:0033036//macromolecule localization;GO:0008150//biological_process;GO:0071702//organic substance transport;GO:0008104//protein localization;GO:0051179//localization
ENSG00000277437	-1.22905	1.03194098	-0.09855	2.552133424	0.000765989	0.037950758	Up	ENSG00000277437	11	5.435996746	20.524759217	0.331595801	61	33.316314249	133.802484362	2.032295169	54	30.024526502	120.785669621	1.831496117	-	-	-	-	-	-	-	-	-

id	CON_Mean	KD_Mean	AllMean	log2FoldChange	pvalue	qvalue	State	gene_id	XF_1_count	XF_1_FPKM	XF_1_TPM	XF_1_CPM	XF_2_count	XF_2_FPKM	XF_2_TPM	XF_2_CPM	XF_3_count	XF_3_FPKM	XF_3_TPM	XF_3_CPM	Symbol	Description	KEGG_A_class	KEGG_B_class	Pathway	K_ID	GO Component	GO Function	GO Process
HRAS	4.53038148	5.664539282	5.097460381	1.158119188	0.000801024	0.039386383	Up	ENSG00000174775	769	9.520148267	35.945339933	23.181561031	1265	17.308064692	69.511352245	42.145137526	1760	24.514663969	98.620043277	59.693206764	HRAS	HRas proto-oncogene, GTPase [Source:HGNC Symbol;Acc:HGNC:5173]	Organismal Systems;Human Diseases;Environmental Information Processing;Cellular Processes	Development;Signal transduction;Infectious diseases;Immune system;Cancers;Endocrine system;Cellular community;Cell motility;Nervous system;Substance dependence;Transport and cata[illegible]	ko04650//Natural killer cell mediated cytotoxicity;ko05200//Pathways in cancer;ko05166//HTLV-I infection;ko04151//PI3K-Akt signaling pathway;ko04144//Endocytosis;ko04010//MAPK signalin[illegible]	K02833	GO:0005794//Golgi apparatus;GO:0070013//intracellular organelle lumen;GO:0005886//plasma membrane;GO:0005634//nucleus;GO:0005622//intracellular;GO:0005829//cytosol;GO:0005623//cell;GO:0044428//nuclear part;GO:0005654//nucleoplasm;GO:0012505//endomembrane system;GO:0044464//cell part;GO:0044444//cytoplasmic part;GO:0043226//organelle;GO:0043233//organelle lumen;GO:0005737//cytoplasm;GO:0071944//cell periphery;GO:0031974//membrane-enclosed lumen;GO:0043229//intracellular organelle;GO:0016020//membrane;GO:0043227//membrane-bounded organelle;GO:0044424//intracellular part;GO:0044446//intracellular organelle part;GO:0044422//organelle part;GO:0005575//cellular_component;GO:0043231//intracellular membrane-bounded organelle;GO:0031981//nuclear lumen	GO:0016818//hydrolase activity, acting on acid anhydrides, in phosphorus-containing anhydrides;GO:0005488//binding;GO:0016787//hydrolase activity;GO:0016462//pyrophosphatase activity;GO:0016817//hydrolase activity, acting on acid anhydrides;GO:0017111//nucleoside-triphosphatase activity;GO:0003924//GTPase activity;GO:0003824//catalytic activity;GO:0043167//ion binding;GO:0003674//molecular_function	GO:0036211//protein modification process;GO:0051649//establishment of localization in cell;GO:0015031//protein transport;GO:0008104//protein localization;GO:0006996//organelle organization;GO:0006950//response to stress;GO:0007154//cell communication;GO:0019538//protein metabolic process;GO:0006928//movement of cell or subcellular component;GO:0008219//cell death;GO:0007049//cell cycle;GO:0044237//cellular metabolic process;GO:0051641//cellular localization;GO:0044260//cellular macromolecule metabolic process;GO:0051234//establishment of localization;GO:0023052//signaling;GO:0050789//regulation of biological process;GO:0071840//cellular component organization or biogenesis;GO:0051179//localization;GO:0034613//cellular protein localization;GO:0051716//cellular response to stimulus;GO:0044699//single-organism process;GO:0008152//metabolic process;GO:0008283//cell proliferation;GO:0022607//cellular component assembly;GO:0046907//intracellular transport;GO:0048856//ana[illegible]

id	CON_Mean	KD_Mean	AllMean	log2FoldChange	pvalue	qvalue	State	gene_id	XF_1_count	XF_1_FPKM	XF_1_TPM	XF_1_CPM	XF_2_count	XF_2_FPKM	XF_2_TPM	XF_2_CPM	XF_3_count	XF_3_FPKM	XF_3_TPM	XF_3_CPM	Symbol	Description	KEGG_A_class	KEGG_B_class	Pathway	K_ID	GO Component	GO Function	GO Process
OSBPL7	3.191322259	4.624420049	3.907871154	1.445628974	0.000819186	0.040163641	Up	ENSG00000006025	302	1.38524224	5.230276024	9.103812004	681	3.452283932	13.8648039 94	22.6884100 04	770	3.973794577	15.9861784 62	26.1157779 59	OSBPL7	oxysterol binding protein like 7 [Source:HGNC Symbol;Acc:HGNC:16387]	-	-	-	-	GO:0005829//cytosol;GO:0012505//endomembrane system;GO:0005575//cellular_component;GO:0071944//cell periphery;GO:0005623//cell;GO:0044444//cytoplasmic part;GO:0005886//plasma membrane;GO:0005783//endoplasmic reticulum;GO:0044464//cell part;GO:0043226//organelle;GO:0016020//membrane;GO:0005622//intracellular;GO:0005737//cytoplasm;GO:0044424//intracellular part;GO:0043229//intracellular organelle;GO:0043231//intracellular membrane-bounded organelle;GO:0043227//membrane-bounded organelle;GO:0005773//vacuole	GO:0008289//lipid binding;GO:0003674//molecular_function;GO:0005488//binding	GO:0008150//biological_process;GO:0044238//primary metabolic process;GO:0044710//single-organism metabolic process;GO:0051234//establishment of localization;GO:0044699//single-organism process;GO:0006810//transport;GO:0006629//lipid metabolic process;GO:0071704//organic substance metabolic process;GO:0009058//biosynthetic process;GO:0009987//cellular process;GO:0051179//localization;GO:0008152//metabolic process;GO:0044281//small molecule metabolic process;GO:0006914//autophagy;GO:0009056//catabolic process
TNFAIP8L1	5.274238206	4.068039079	4.671138642	-1.19512	0.000825115	0.040259107	Down	ENSG00000185361	1290	5.831030738	22.0162938 8	38.8871439 92	426	2.128167622	8.546987305	14.1927498 7	567	2.883597125	11.6004230 61	19.2307092 24	TNFAIP8L1	TNF alpha induced protein 8 like 1 [Source:HGNC Symbol;Acc:HGNC:28279]	-	-	-	-	GO:0005623//cell;GO:0005622//intracellular;GO:0005575//cellular_component;GO:0044424//intracellular part;GO:0044464//cell part;GO:0005737//cytoplasm	GO:0003674//molecular_function	GO:0009987//cellular process;GO:0023052//signaling;GO:0044699//single-organism process;GO:0008219//cell death;GO:0050789//regulation of biological process;GO:0007154//cell communication;GO:0065007//biological regulation;GO:0008150//biological_process;GO:0050896//response to stimulus;GO:0051716//cellular response to stimulus;GO:0050794//regulation of cellular process;GO:0007165//signal transduction;GO:0044763//single-organism cellular process;GO:0044700//single organism signaling
LINC01239	5.387890246	3.882576308	4.635233277	-1.42084	0.000827125	0.040259107	Down	ENSG00000234840	1396	11.7878212 09	44.5074203 18	42.0825217 15	303	2.827687176	11.3563453 11	10.0948432 18	615	5.842774726	23.5048988 27	20.8587057 73	-	-	-	-	-	-	-	-	-

id	CON_Mean	KD_Mean	AllMean	log2FoldChange	pvalue	qvalue	State	gene_id	XF_1_count	XF_1_FPKM	XF_1_TPM	XF_1_CPM	XF_2_count	XF_2_FPKM	XF_2_TPM	XF_2_CPM	XF_3_count	XF_3_FPKM	XF_3_TPM	XF_3_CPM	Symbol	Description	KEGG_A_class	KEGG_B_class	Pathway	K_ID	GO Component	GO Function	GO Process
TLE3	4.714300192	5.817136079	5.265718136	1.126351722	0.000830571	0.040259107	Up	ENSG00000140332	874	2.085388134	7.873825413	26.346793681	1977	5.213420395	20.937748265	65.866353271	1392	3.736892504	15.033145098	47.211899895	TLE3	TLE family member 3, transcriptional corepressor [Source:HGNC Symbol;Acc:HGNC:11839]	-	-	-	-	GO:0031974//membrane-enclosed lumen;GO:0044446//intracellular organelle part;GO:0070013//intracellular organelle lumen;GO:0043227//membrane-bounded organelle;GO:0005654//nucleoplasm;GO:0005623//cell;GO:0043229//intracellular organelle;GO:0043226//organelle;GO:0044464//cell part;GO:0043231//intracellular membrane-bounded organelle;GO:0044428//nuclear part;GO:0043233//organelle lumen;GO:0005575//cellular_component;GO:0005634//nucleus;GO:0044422//organelle part;GO:0031981//nuclear lumen;GO:0032991//macromolecular complex;GO:0044424//intracellular part;GO:0005622//intracellular	GO:0008134//transcription factor binding;GO:0003674//molecular_function;GO:0005515//protein binding;GO:0005488//binding	GO:0006807//nitrogen compound metabolic process;GO:0007165//signal transduction;GO:0071840//cellular component organization or biogenesis;GO:0043933//macromolecular complex subunit organization;GO:0008150//biological_process;GO:0007267//cell-cell signaling;GO:0044699//single-organism process;GO:0034641//cellular nitrogen compound metabolic process;GO:0048856//anatomical structure development;GO:0044763//single-organism cellular process;GO:0009058//biosynthetic process;GO:0009987//cellular process;GO:0065007//biological regulation;GO:0042592//homeostatic process;GO:0050896//response to stimulus;GO:0051716//cellular response to stimulus;GO:0016043//cellular component organization;GO:0007154//cell communication;GO:0065003//macromolecular complex assembly;GO:0023052//signaling;GO:0044085//cellular component biogenesis;GO:0032502//developmental process;GO:0044700//single organism signaling;GO:0065008//reg[illegible]

id	CON_Mean	KD_Mean	AllMean	log2FoldChange	pvalue	qvalue	State	gene_id	XF_1_count	XF_1_FPKM	XF_1_TPM	XF_1_CPM	XF_2_count	XF_2_FPKM	XF_2_TPM	XF_2_CPM	XF_3_count	XF_3_FPKM	XF_3_TPM	XF_3_CPM	Symbol	Description	KEGG_A_class	KEGG_B_class	Pathway	K_ID	GO Component	GO Function	GO Process
ABCA10	4.441780495	2.901653941	3.671717218	-1.53593	0.000842618	0.0406122884	Down	ENSG00000154263	723	2.593085982	9.790746371	21.794887679	254	1.006822584	4.043525405	8.462343819	186	0.750563548	3.019442148	6.308486624	ABCA10	ATP binding cassette subfamily A member 10 [Source:HGNC Symbol;Acc:HGNC:30]	Environmental Information Processing	Membrane transport	ko02010//ABC transporters	K05652	GO:0005575//cellular_component;GO:0044464//cell part;GO:0005622//intracellular;GO:0005623//cell;GO:0043226//organelle	GO:0003674//molecular_function;GO:0016462//pyrophosphatase activity;GO:0016817//hydrolase activity, acting on acid anhydrides;GO:0022857//transmembrane transporter activity;GO:0016787//hydrolase activity;GO:0016818//hydrolase activity, acting on acid anhydrides, in phosphorus-containing anhydrides;GO:0005215//transporter activity;GO:0043167//ion binding;GO:0005488//binding;GO:0003824//catalytic activity;GO:0016887//ATPase activity;GO:0017111//nucleoside-triphosphatase activity	GO:0006810//transport;GO:0055085//transmembrane transport;GO:0008150//biological_process;GO:0051234//establishment of localization;GO:0051179//localization
TMEM187	2.168725722	0.25548717	1.212106446	-1.96899	0.000852197	0.04095829	Down	ENSG00000177854	147	2.962116117	11.184097952	4.431325711	26	0.579026852	2.325444247	0.86622417	40	0.906860822	3.648210461	1.35666379	TMEM187	transmembrane protein 187 [Source:HGNC Symbol;Acc:HGNC:13705]	-	-	-	-	GO:0043226//organelle;GO:0005737//cytoplasm;GO:0005623//cell;GO:0005575//cellular_component;GO:0031410//cytoplasmic vesicle;GO:0097708//intracellular vesicle;GO:0044444//cytoplasmic part;GO:0043227//membrane-bounded organelle;GO:0044464//cell part;GO:0005622//intracellular;GO:0044424//intracellular part;GO:0031982//vesicle	GO:0003674//molecular_function	GO:0008150//biological_process

id	CON_Mean	KD_Mean	AllMean	log2FoldChange	pvalue	qvalue	State	gene_id	XF_1_count	XF_1_FPKM	XF_1_TPM	XF_1_CPM	XF_2_count	XF_2_FPKM	XF_2_TPM	XF_2_CPM	XF_3_count	XF_3_FPKM	XF_3_TPM	XF_3_CPM	Symbol	Description	KEGG_A_class	KEGG_B_class	Pathway	K_ID	GO Component	GO Function	GO Process
TPH1	-0.72227	1.510342974	0.394036903	2.460822455	0.000860945	0.041146924	Up	ENSG00000129167	17	0.090477796	0.341618119	0.512466239	65	0.382337646	1.535515799	2.165560426	101	0.604798035	2.433042054	3.42557607	TPH1	tryptophan hydroxylase 1 [Source:HGNC Symbol;Acc:HGNC:12008]	Metabolism; Organismal Systems	Amino acid metabolism; Nervous system	ko04726//Serotonergic synapse; ko00380//Tryptophan metabolism	K00502	GO:0005575//cellular_component;GO:0044424//intracellular part;GO:0044464//cell part;GO:0044444//cytoplasmic part;GO:0005622//intracellular;GO:0005623//cell;GO:0005829//cytosol;GO:0005737//cytoplasm	GO:0005488//binding;GO:0016491//oxidoreductase activity;GO:0003674//molecular_function;GO:0043167//ion binding;GO:0003824//catalytic activity	GO:0043436//oxoacid metabolic process;GO:0044763//single-organism cellular process;GO:0044237//cellular metabolic process;GO:0006807//nitrogen compound metabolic process;GO:0030154//cell differentiation;GO:0071704//organic substance metabolic process;GO:0044767//single-organism developmental process;GO:0044281//small molecule metabolic process;GO:0032502//developmental process;GO:0044238//primary metabolic process;GO:0006950//response to stress;GO:0009058//biosynthetic process;GO:0006082//organic acid metabolic process;GO:0048856//anatomical structure development;GO:0044710//single-organism metabolic process;GO:1901564//organonitrogen compound metabolic process;GO:0008150//biological_process;GO:0019752//carboxylic acid metabolic process;GO:0034641//cellular nitrogen compound metabolic process;GO:0048869//cellular developmental process;GO:0009987//cellular process;GO:0044699//single-organism process;GO:0008152//metabolic process;GO:0050896//resp
ENSG00000280023	-3.35941	-0.91952	-2.13946	6.775675036	0.000880436	0.041844032	Up	ENSG00000280023	0	0	0	0	16	0.640698351	2.573124699	0.533061028	10	0.407651379	1.63994076	0.339165948	-	-	-	-	-	-	-	-	-

id	CON_Mean	KD_Mean	AllMean	log2FoldChange	pvalue	qvalue	State	gene_id	XF_1_count	XF_1_FPKM	XF_1_TPM	XF_1_CPM	XF_2_count	XF_2_FPKM	XF_2_TPM	XF_2_CPM	XF_3_count	XF_3_FPKM	XF_3_TPM	XF_3_CPM	Symbol	Description	KEGG_A_class	KEGG_B_class	Pathway	K_ID	GO Component	GO Function	GO Process
EEF1A2	6.253306521	5.091500613	5.672403567	-1.11171	0.000891572	0.042059637	Down	ENSG00000101210	2546	15.903306155	60.046307029	76.749355506	1321	9.119529864	36.625172367	44.010851124	760	5.341196024	21.487097833	25.776612012	EEF1A2	eukaryotic translation elongation factor 1 alpha 2 [Source:HGNC Symbol;Acc:HGNC:3192]	Genetic Information Processing;Human Diseases	Infectious diseases;Translation	ko03013//RNA transport;ko05134//Legionellosis	K03231	GO:0044464//cell part;GO:0005737//cytoplasm;GO:0044444//cytoplasmic part;GO:0044424//intracellular part;GO:0000323//lytic vacuole;GO:0043229//intracellular organelle;GO:0043227//membrane-bounded organelle;GO:0005764//lysosome;GO:0005623//cell;GO:0005575//cellular_component;GO:0043231//intracellular membrane-bounded organelle;GO:0032991//macromolecular complex;GO:0043226//organelle;GO:0005622//intracellular;GO:0005773//vacuole	GO:0003824//catalytic activity;GO:1901363//heterocyclic compound binding;GO:0043167//ion binding;GO:0016817//hydrolase activity, acting on acid anhydrides;GO:0008135//translation factor activity, RNA binding;GO:0097159//organic cyclic compound binding;GO:0019899//enzyme binding;GO:0003674//molecular_function;GO:0005488//binding;GO:0017111//nucleoside-triphosphatase activity;GO:0016787//hydrolase activity;GO:0016462//pyrophosphatase activity;GO:0005515//protein binding;GO:0016818//hydrolase activity, acting on acid anhydrides, in phosphorus-containing anhydrides;GO:0003924//GTPase activity;GO:0003676//nucleic acid binding;GO:0003723//RNA binding	GO:0043604//amide biosynthetic process;GO:0044237//cellular metabolic process;GO:0043170//macromolecule metabolic process;GO:0006807//nitrogen compound metabolic process;GO:0008152//metabolic process;GO:1901566//organonitrogen compound biosynthetic process;GO:0034645//cellular macromolecule biosynthetic process;GO:0009056//catabolic process;GO:0009058//biosynthetic process;GO:0044260//cellular macromolecule metabolic process;GO:0010467//gene expression;GO:0043043//peptide biosynthetic process;GO:0008219//cell death;GO:0009059//macromolecule biosynthetic process;GO:0043603//cellular amide metabolic process;GO:0006412//translation;GO:0044699//single-organism process;GO:0006629//lipid metabolic process;GO:0006518//peptide metabolic process;GO:0044763//single-organism cellular process;GO:0044267//cellular protein metabolic process;GO:0044710//single-organism metabolic process;GO:0044249//cellular biosynthetic process;GO:0044238//primary metabolic [illegible]

id	CON_Mean	KD_Mean	AllMean	log2FoldChange	pvalue	qvalue	State	gene_id	XF_1_count	XF_1_FPKM	XF_1_TPM	XF_1_CPM	XF_2_count	XF_2_FPKM	XF_2_TPM	XF_2_CPM	XF_3_count	XF_3_FPKM	XF_3_TPM	XF_3_CPM	Symbol	Description	KEGG_A_class	KEGG_B_class	Pathway	K_ID	GO Component	GO Function	GO Process
ANO1	3.946591803	5.350170898	4.648381351	1.49687127	0.000892368	0.042059637	Up	ENSG00000131620	512	2.563407624	9.678689433	15.4342773 05	847	4.686749405	18.8225717 08	28.2189181 69	1698	9.564919098	38.4787136 63	57.5903778 89	ANO1	anoctamin 1 [Source:HGNC Symbol;Acc:HGNC:21625]	-	-	-	-	GO:0043227//membrane-bounded organelle;GO:0005575//cellular_component;GO:0005886//plasma membrane;GO:0043233//organelle lumen;GO:0005737//cytoplasm;GO:0043231//intracellular membrane-bounded organelle;GO:0005622//intracellular;GO:0005623//cell;GO:0044422//organelle part;GO:0043226//organelle;GO:0071944//cell periphery;GO:0031981//nuclear lumen;GO:0044464//cell part;GO:0016020//membrane;GO:0032991//macromolecular complex;GO:0005634//nucleus;GO:0031974//membrane-enclosed lumen;GO:0044428//nuclear part;GO:0044446//intracellular organelle part;GO:0005615//extracellular space;GO:0005576//extracellular region;GO:0005654//nucleoplasm;GO:0044424//intracellular part;GO:0043229//intracellular organelle;GO:0070013//intracellular organelle lumen;GO:0044421//extracellular region part	GO:0005215//transporter activity;GO:0005488//binding;GO:0003674//molecular_function;GO:0043167//ion binding;GO:0022857//transmembrane transporter activity	GO:0006950//response to stress;GO:0065008//regulation of biological quality;GO:0051234//establishment of localization;GO:0065007//biological regulation;GO:0050789//regulation of biological process;GO:0050896//response to stimulus;GO:0050794//regulation of cellular process;GO:0071702//organic substance transport;GO:0033036//macromolecule localization;GO:0023052//signaling;GO:0048856//anatomical structure development;GO:0008104//protein localization;GO:0032501//multicellular organismal process;GO:0051179//localization;GO:0007165//signal transduction;GO:0045184//establishment of protein localization;GO:0044699//single-organism process;GO:0044700//single organism signaling;GO:0051716//cellular response to stimulus;GO:0050877//neurological system process;GO:0015031//protein transport;GO:0003008//system process;GO:0007267//cell-cell signaling;GO:0007154//cell communication;GO:0044763//single-organism cellular process;GO:0032502//developmental process;GO:0042592//hom[illegible]

id	CON_Mean	KD_Mean	AllMean	log2FoldChange	pvalue	qvalue	State	gene_id	XF_1_count	XF_1_FPKM	XF_1_TPM	XF_1_CPM	XF_2_count	XF_2_FPKM	XF_2_TPM	XF_2_CPM	XF_3_count	XF_3_FPKM	XF_3_TPM	XF_3_CPM	Symbol	Description	KEGG_A_class	KEGG_B_class	Pathway	K_ID	GO Component	GO Function	GO Process
RASL10A	-1.70414	0.56309334	-0.57052	2.724038585	0.000909647	0.042638444	Up	ENSG00000100276	7	0.096751724	0.365306664	0.21101551	50	0.763785288	3.06745723	1.665815712	33	0.513180938	2.064475631	1.119247627	RASL10A	RAS like family 10 member A [Source:HGNC Symbol;Acc:HGNC:16954]	-	-	-	-	GO:0044464//cell part;GO:0043226//organelle;GO:0005623//cell;GO:0043231//intracellular membrane-bounded organelle;GO:0031981//nuclear lumen;GO:0043228//non-membrane-bounded organelle;GO:0044422//organelle part;GO:0005622//intracellular;GO:0005886//plasma membrane;GO:0043233//organelle lumen;GO:0044446//intracellular organelle part;GO:0043229//intracellular organelle;GO:0043227//membrane-bounded organelle;GO:0031974//membrane-enclosed lumen;GO:0043232//intracellular non-membrane-bounded organelle;GO:0005575//cellular_component;GO:0044428//nuclear part;GO:0044424//intracellular part;GO:0005730//nucleolus;GO:0070013//intracellular organelle lumen;GO:0071944//cell periphery;GO:0016020//membrane;GO:0005634//nucleus	GO:0005488//binding;GO:0003674//molecular_function;GO:0017111//nucleoside-triphosphatase activity;GO:0003824//catalytic activity;GO:0016787//hydrolase activity;GO:0016462//pyrophosphatase activity;GO:0003924//GTPase activity;GO:0016818//hydrolase activity, acting on acid anhydrides, in phosphorus-containing anhydrides;GO:0016817//hydrolase activity, acting on acid anhydrides;GO:0043167//ion binding	GO:0044763//single-organism cellular process;GO:0050789//regulation of biological process;GO:0008150//biological_process;GO:0009987//cellular process;GO:0051716//cellular response to stimulus;GO:0050896//response to stimulus;GO:0007165//signal transduction;GO:0007154//cell communication;GO:0065007//biological regulation;GO:0044699//single-organism process;GO:0023052//signaling;GO:0050794//regulation of cellular process;GO:0044700//single organism signaling
ENSG00000111788	3.662832987	4.943000155	4.302916571	1.297768699	0.000928634	0.043369297	Up	ENSG00000111788	420	4.009161052	15.137438285	12.660930602	1030	10.866308954	43.640455634	34.315803677	793	8.516738328	34.261987173	26.895859638	-	-	-	-	-	-	-	-	-

id	CON_Mean	KD_Mean	AllMean	log2FoldChange	pvalue	qvalue	State	gene_id	XF_1_count	XF_1_FPKM	XF_1_TPM	XF_1_CPM	XF_2_count	XF_2_FPKM	XF_2_TPM	XF_2_CPM	XF_3_count	XF_3_FPKM	XF_3_TPM	XF_3_CPM	Symbol	Description	KEGG_A_class	KEGG_B_class	Pathway	K_ID	GO Component	GO Function	GO Process
DMPK	3.842098919	5.050936761	4.44651784	1.215067304	0.000930323	0.043369297	Up	ENSG00000104936	476	1.724231517	6.510201969	14.349054682	1014	4.05944997	16.303258729	33.782742649	936	3.814699914	15.346156534	31.745932688	DMPK	DM1 protein kinase [Source:HGNC Symbol;Acc:HGNC:2933]	-	-	-	-	GO:0043226//organelle;GO:0031975//envelope;GO:0012505//endomembrane system;GO:0044422//organelle part;GO:0044446//intracellular organelle part;GO:0044424//intracellular part;GO:0005635//nuclear envelope;GO:0044464//cell part;GO:0044428//nuclear part;GO:0031967//organelle envelope;GO:0005886//plasma membrane;GO:0044444//cytoplasmic part;GO:0005737//cytoplasm;GO:0005829//cytosol;GO:0071944//cell periphery;GO:0016020//membrane;GO:0005622//intracellular;GO:0043227//membrane-bounded organelle;GO:0005783//endoplasmic reticulum;GO:0005623//cell;GO:0043229//intracellular organelle;GO:0005634//nucleus;GO:0005575//cellular_component;GO:0043231//intracellular membrane-bounded organelle;GO:0005739//mitochondrion	GO:0030234//enzyme regulator activity;GO:0003824//catalytic activity;GO:0016740//transferase activity;GO:0043167//ion binding;GO:0098772//molecular function regulator;GO:0016772//transferase activity, transferring phosphorus-containing groups;GO:0016301//kinase activity;GO:0003674//molecular_function;GO:0005488//binding	GO:0008150//biological_process;GO:0042592//homeostatic process;GO:0003013//circulatory system process;GO:0050789//regulation of biological process;GO:0061024//membrane organization;GO:0050794//regulation of cellular process;GO:0051716//cellular response to stimulus;GO:0044763//single-organism cellular process;GO:0008219//cell death;GO:0007267//cell-cell signaling;GO:0016043//cellular component organization;GO:0019538//protein metabolic process;GO:0006464//cellular protein modification process;GO:0032502//developmental process;GO:0009987//cellular process;GO:0008152//metabolic process;GO:0043170//macromolecule metabolic process;GO:0048856//anatomical structure development;GO:0003008//system process;GO:0007165//signal transduction;GO:0043412//macromolecule modification;GO:0044238//primary metabolic process;GO:0006810//transport;GO:0044260//cellular macromolecule metabolic process;GO:0051234//establishment of localization;GO:0044767//s[illegible]

id	CON_Mean	KD_Mean	AllMean	log2FoldChange	pvalue	qvalue	State	gene_id	XF_1_count	XF_1_FPKM	XF_1_TPM	XF_1_CPM	XF_2_count	XF_2_FPKM	XF_2_TPM	XF_2_CPM	XF_3_count	XF_3_FPKM	XF_3_TPM	XF_3_CPM	Symbol	Description	KEGG_A_class	KEGG_B_class	Pathway	K_ID	GO Component	GO Function	GO Process
DHX37	5.498023422	6.512918186	6.005470804	1.040344572	0.000938574	0.043634727	Up	ENSG00000150990	1507	6.576234048	24.829967107	45.428624803	3245	15.650179464	62.853077845	108.11143974	2228	10.938936466	44.006248222	75.566173108	DHX37	DEAH-box helicase 37 [Source:HGNC Symbol;Acc:HGNC:17210]	-	-	-	-	GO:0043232//intracellular non-membrane-bounded organelle;GO:0043226//organelle;GO:0005623//cell;GO:0043233//organelle lumen;GO:0044464//cell part;GO:0044422//organelle part;GO:0005622//intracellular;GO:0043229//intracellular organelle;GO:0044428//nuclear part;GO:0043231//intracellular membrane-bounded organelle;GO:0044424//intracellular part;GO:0005575//cellular_component;GO:0031981//nuclear lumen;GO:0005730//nucleolus;GO:0031974//membrane-enclosed lumen;GO:0044446//intracellular organelle part;GO:0005654//nucleoplasm;GO:0070013//intracellular organelle lumen;GO:0005634//nucleus;GO:0043227//membrane-bounded organelle;GO:0043228//non-membrane-bounded organelle	GO:0004386//helicase activity;GO:0097159//organic cyclic compound binding;GO:0003676//nucleic acid binding;GO:1901363//heterocyclic compound binding;GO:0005488//binding;GO:0017111//nucleoside-triphosphatase activity;GO:0043167//ion binding;GO:0016817//hydrolase activity, acting on acid anhydrides;GO:0016787//hydrolase activity;GO:0003674//molecular_function;GO:0003824//catalytic activity;GO:0016462//pyrophosphatase activity;GO:0016818//hydrolase activity, acting on acid anhydrides, in phosphorus-containing anhydrides;GO:0003723//RNA binding;GO:0016887//ATPase activity	GO:0006807//nitrogen compound metabolic process;GO:0034641//cellular nitrogen compound metabolic process;GO:0071840//cellular component organization or biogenesis;GO:0008152//metabolic process;GO:0022613//ribonucleoprotein complex biogenesis;GO:0009987//cellular process;GO:0008150//biological_process;GO:0044237//cellular metabolic process;GO:0042254//ribosome biogenesis;GO:0044085//cellular component biogenesis

id	CON_Mean	KD_Mean	AllMean	log2FoldChange	pvalue	qvalue	State	gene_id	XF_1_count	XF_1_FPKM	XF_1_TPM	XF_1_CPM	XF_2_count	XF_2_FPKM	XF_2_TPM	XF_2_CPM	XF_3_count	XF_3_FPKM	XF_3_TPM	XF_3_CPM	Symbol	Description	KEGG_A_class	KEGG_B_class	Pathway	K_ID	GO Component	GO Function	GO Process
SH3GL2	2.590334659	0.623303066	1.606818862	-1.9715	0.000941636	0.043658137	Down	ENSG00000107295	198	2.216384859	8.368431348	5.968724427	58	0.717544087	2.881746785	1.932346226	31	0.390424967	1.570640627	1.051414437	SH3GL2	SH3 domain containing GRB2 like 2, endophilin A1 [Source:HGNC Symbol;Acc:HGNC:10831]	Cellular Processes	Transport and catabolism	ko04144//Endocytosis	K11247	GO:0044424//intracellular part;GO:0031982//vesicle;GO:0005768//endosome;GO:0016020//membrane;GO:0005773//vacuole;GO:0071944//cell periphery;GO:0043229//intracellular organelle;GO:0043226//organelle;GO:0031410//cytoplasmic vesicle;GO:0097708//intracellular vesicle;GO:0044444//cytoplasmic part;GO:0005623//cell;GO:0005886//plasma membrane;GO:0044464//cell part;GO:0005794//Golgi apparatus;GO:0005737//cytoplasm;GO:0043231//intracellular membrane-bounded organelle;GO:0012505//endomembrane system;GO:0005622//intracellular;GO:0005575//cellular_component;GO:0005829//cytosol;GO:0043227//membrane-bounded organelle	GO:0008289//lipid binding;GO:0019899//enzyme binding;GO:0005488//binding;GO:0005515//protein binding;GO:0003674//molecular_function	GO:0044763//single-organism cellular process;GO:0030154//cell differentiation;GO:0043412//macromolecule modification;GO:0065003//macromolecular complex assembly;GO:0002376//immune system process;GO:0044767//single-organism developmental process;GO:0023052//signaling;GO:0061024//membrane organization;GO:0044267//cellular protein metabolic process;GO:0040007//growth;GO:0044237//cellular metabolic process;GO:0022607//cellular component assembly;GO:0044085//cellular component biogenesis;GO:0000902//cell morphogenesis;GO:0009653//anatomical structure morphogenesis;GO:0016192//vesicle-mediated transport;GO:0051179//localization;GO:0008150//biological_process;GO:0043933//macromolecular complex subunit organization;GO:0036211//protein modification process;GO:0071840//cellular component organization or biogenesis;GO:0032989//cellular component morphogenesis;GO:0051234//establishment of localization;GO:0044802//single-organism membrane organization;GO:0007154//cell communication;GO:007170 [illegible]

id	CON_Mean	KD_Mean	AllMean	log2FoldChange	pvalue	qvalue	State	gene_id	XF_1_count	XF_1_FPKM	XF_1_TPM	XF_1_CPM	XF_2_count	XF_2_FPKM	XF_2_TPM	XF_2_CPM	XF_3_count	XF_3_FPKM	XF_3_TPM	XF_3_CPM	Symbol	Description	KEGG_A_class	KEGG_B_class	Pathway	K_ID	GO Component	GO Function	GO Process
HBP1	5.905577077	4.535674261	5.220625669	-1.28717	0.000945792	0.043732009	Down	ENSG00000105856	2000	9.503490814	35.8824461 83	60.2901457 23	482	2.531283649	10.1659516 81	16.0584634 68	961	5.13774394	20.6686304 32	32.5938475 57	HBP1	HMG-box transcription factor 1 [Source:HGNC Symbol;Acc:HGNC:23200]	-	-	-	-	GO:0000228//nuclear chromosome;GO:0005575//cellular_component;GO:0044446//intracellular organelle part;GO:0043231//intracellular membrane-bounded organelle;GO:0044424//intracellular part;GO:0031981//nuclear lumen;GO:0043233//organelle lumen;GO:0043226//organelle;GO:0005694//chromosome;GO:0043232//intracellular non-membrane-bounded organelle;GO:0043227//membrane-bounded organelle;GO:0044464//cell part;GO:0005654//nucleoplasm;GO:0044428//nuclear part;GO:0031974//membrane-enclosed lumen;GO:0070013//intracellular organelle lumen;GO:0043228//non-membrane-bounded organelle;GO:0005622//intracellular;GO:0044422//organelle part;GO:0005634//nucleus;GO:0005623//cell;GO:0043229//intracellular organelle	GO:0097159//organic cyclic compound binding;GO:0003723//RNA binding;GO:0003700//transcription factor activity, sequence-specific DNA binding;GO:0003676//nucleic acid binding;GO:0001071//nucleic acid binding transcription factor activity;GO:0003674//molecular_function;GO:1901363//heterocyclic compound binding;GO:0003677//DNA binding;GO:0005488//binding	GO:0009058//biosynthetic process;GO:0050789//regulation of biological process;GO:0007267//cell-cell signaling;GO:0008152//metabolic process;GO:0051716//cellular response to stimulus;GO:0006807//nitrogen compound metabolic process;GO:0009987//cellular process;GO:0023052//signaling;GO:0044763//single-organism cellular process;GO:0008150//biological_process;GO:0007165//signal transduction;GO:0050896//response to stimulus;GO:0044700//single organism signaling;GO:0034641//cellular nitrogen compound metabolic process;GO:0050794//regulation of cellular process;GO:0044237//cellular metabolic process;GO:0044699//single-organism process;GO:0065007//biological regulation;GO:0007154//cell communication;GO:0007049//cell cycle

id	CON_Mean	KD_Mean	AllMean	log2FoldChange	pvalue	qvalue	State	gene_id	XF_1_count	XF_1_FPKM	XF_1_TPM	XF_1_CPM	XF_2_count	XF_2_FPKM	XF_2_TPM	XF_2_CPM	XF_3_count	XF_3_FPKM	XF_3_TPM	XF_3_CPM	Symbol	Description	KEGG_A_class	KEGG_B_class	Pathway	K_ID	GO Component	GO Function	GO Process
RAB26	4.362149927	2.499259739	3.430704833	-1.77391	0.000952362	0.043883669	Down	ENSG00000167964	684	8.2874718	31.291108359	20.619229837	111	1.486378972	5.969483828	3.698110882	241	3.285329315	13.216551515	8.173899335	RAB26	RAB26, member RAS oncogene family [Source:HGNC Symbol;Acc:HGNC:14259]	-	-	-	-	GO:0016020//membrane;GO:0044464//cell part;GO:0097708//intracellular vesicle;GO:0043229//intracellular organelle;GO:0071944//cell periphery;GO:0005623//cell;GO:0012505//endomembrane system;GO:0005886//plasma membrane;GO:0044424//intracellular part;GO:0043226//organelle;GO:0043227//membrane-bounded organelle;GO:0044444//cytoplasmic part;GO:0005622//intracellular;GO:0005575//cellular_component;GO:0005737//cytoplasm;GO:0031982//vesicle;GO:0043231//intracellular membrane-bounded organelle;GO:0005794//Golgi apparatus;GO:0031410//cytoplasmic vesicle	GO:0043167//ion binding;GO:0003824//catalytic activity;GO:0016462//pyrophosphatase activity;GO:0016787//hydrolase activity;GO:0016818//hydrolase activity, acting on acid anhydrides, in phosphorus-containing anhydrides;GO:0016817//hydrolase activity, acting on acid anhydrides;GO:0005488//binding;GO:0017111//nucleoside-triphosphatase activity;GO:0003924//GTPase activity;GO:0003674//molecular_function	GO:0006810//transport;GO:0050789//regulation of biological process;GO:0009056//catabolic process;GO:0051234//establishment of localization;GO:0015031//protein transport;GO:0050896//response to stimulus;GO:0009987//cellular process;GO:0045184//establishment of protein localization;GO:0007267//cell-cell signaling;GO:0008104//protein localization;GO:0033036//macromolecule localization;GO:0008152//metabolic process;GO:0023052//signaling;GO:0051716//cellular response to stimulus;GO:0050794//regulation of cellular process;GO:0008150//biological_process;GO:0016192//vesicle-mediated transport;GO:0048856//anatomical structure development;GO:0044700//single organism signaling;GO:0065007//biological regulation;GO:0051179//localization;GO:0007154//cell communication;GO:0044763//single-organism cellular process;GO:0044699//single-organism process;GO:0032502//developmental process;GO:0007165//signal transduction;GO:0071702//[illegible]

id	CON_Mean	KD_Mean	AllMean	log2FoldChange	pvalue	qvalue	State	gene_id	XF_1_count	XF_1_FPKM	XF_1_TPM	XF_1_CPM	XF_2_count	XF_2_FPKM	XF_2_TPM	XF_2_CPM	XF_3_count	XF_3_FPKM	XF_3_TPM	XF_3_CPM	Symbol	Description	KEGG_A_class	KEGG_B_class	Pathway	K_ID	GO Component	GO Function	GO Process
JUP	3.952180877	2.13178296	3.041981919	-1.77154	0.000954216	0.043883669	Down	ENSG00000173801	514	3.13528277	11.837925398	15.494567451	92	0.620214672	2.490859685	3.065100911	173	1.187286704	4.77633576	5.867570892	JUP	junction plakoglobin [Source:HGNC Symbol;Acc:HGNC:6207]	Human Diseases	Cardiovascular diseases;Cancers	ko05200//Pathways in cancer;ko05202//Transcriptional misregulation in cancers;ko05412//Arrhythmogenic right ventricular cardiomyopathy (ARVC);ko05221//Acute myeloid leukemia	K10056	GO:0044421//extracellular region part;GO:0031410//cytoplasmic vesicle;GO:0005622//intracellular;GO:0005615//extracellular space;GO:0044464//cell part;GO:0005856//cytoskeleton;GO:0043231//intracellular membrane-bounded organelle;GO:0043232//intracellular non-membrane-bounded organelle;GO:0005575//cellular_component;GO:0005829//cytosol;GO:0032991//macromolecular complex;GO:0044424//intracellular part;GO:0071944//cell periphery;GO:0005886//plasma membrane;GO:0016020//membrane;GO:0005737//cytoplasm;GO:0005634//nucleus;GO:0005623//cell;GO:0031982//vesicle;GO:0043227//membrane-bounded organelle;GO:0043228//non-membrane-bounded organelle;GO:0044444//cytoplasmic part;GO:0005576//extracellular region;GO:0043229//intracellular organelle;GO:0097708//intracellular vesicle;GO:0043226//organelle	GO:0005198//structural molecule activity;GO:0019899//enzyme binding;GO:0005515//protein binding;GO:0005488//binding;GO:0008134//transcription factor binding;GO:0003674//molecular_function	GO:0071840//cellular component organization or biogenesis;GO:0032502//developmental process;GO:0007165//signal transduction;GO:0008152//metabolic process;GO:0044699//single-organism process;GO:0051169//nuclear transport;GO:0007267//cell-cell signaling;GO:0071702//organic substance transport;GO:0051649//establishment of localization in cell;GO:0050794//regulation of cellular process;GO:0023052//signaling;GO:0051716//cellular response to stimulus;GO:0044237//cellular metabolic process;GO:0032501//multicellular organismal process;GO:0006810//transport;GO:0044767//single-organism developmental process;GO:0008283//cell proliferation;GO:0006807//nitrogen compound metabolic process;GO:0050896//response to stimulus;GO:0007010//cytoskeleton organization;GO:0003013//circulatory system process;GO:0008219//cell death;GO:0034641//cellular nitrogen compound metabolic process;GO:0051234//establishment of localization;GO:0048870//c
COQ5	5.697547167	4.693672297	5.195609732	-1.00614	0.000964229	0.044106369	Down	ENSG00000110871	1731	20.864102808	78.77684746	52.181121124	747	9.950934324	39.964196649	24.887286744	773	10.48281797	42.171329095	26.217527743	COQ5	coenzyme Q5, methyltransferase [Source:HGNC Symbol;Acc:HGNC:28722]	Metabolism	Metabolism of cofactors and vitamins	ko00130//Ubiquinone and other terpenoid-quinone biosynthesis	K06127	GO:0043229//intracellular organelle;GO:0043227//membrane-bounded organelle;GO:0005737//cytoplasm;GO:0043231//intracellular membrane-bounded organelle;GO:0005622//intracellular;GO:0005739//mitochondrion;GO:0044444//cytoplasmic part;GO:0044424//intracellular part;GO:0044464//cell part;GO:0032991//macromolecular complex;GO:0043226//organelle;GO:0005575//cellular_component;GO:0005623//cell	GO:0016741//transferase activity, transferring one-carbon groups;GO:0003674//molecular_function;GO:0016740//transferase activity;GO:0008168//methyltransferase activity;GO:0003824//catalytic activity	GO:0009987//cellular process;GO:0008152//metabolic process;GO:0009058//biosynthetic process;GO:0044710//single-organism metabolic process;GO:0044699//single-organism process;GO:0044237//cellular metabolic process;GO:0044281//small molecule metabolic process;GO:0051186//cofactor metabolic process;GO:0008150//biological_process

id	CON_Mean	KD_Mean	AllMean	log2FoldChange	pvalue	qvalue	State	gene_id	XF_1_count	XF_1_FPKM	XF_1_TPM	XF_1_CPM	XF_2_count	XF_2_FPKM	XF_2_TPM	XF_2_CPM	XF_3_count	XF_3_FPKM	XF_3_TPM	XF_3_CPM	Symbol	Description	KEGG_A_class	KEGG_B_class	Pathway	K_ID	GO Component	GO Function	GO Process
SERPINB1	5.96690992	4.658850834	5.312880377	-1.23536	0.000975616	0.044507933	Down	ENSG00000021355	2087	20.647445705	77.958812591	62.912767062	537	5.871631359	23.58120581	17.890860752	1024	11.398291115	45.854186068	34.730593026	SERPINB1	serpin family B member 1 [Source:HGNC Symbol;Acc:HGNC:3311]	Human Diseases	Infectious diseases	ko05146//Amoebiasis	K13963	GO:0044464//cell part;GO:0031012//extracellular matrix;GO:0005737//cytoplasm;GO:0044421//extracellular region part;GO:0043226//organelle;GO:0031410//cytoplasmic vesicle;GO:0005615//extracellular space;GO:0005623//cell;GO:0031982//vesicle;GO:0005622//intracellular;GO:0005575//cellular_component;GO:0044424//intracellular part;GO:0097708//intracellular vesicle;GO:0044444//cytoplasmic part;GO:0043227//membrane-bounded organelle;GO:0005576//extracellular region	GO:0003674//molecular_function;GO:0098772//molecular function regulator;GO:0030234//enzyme regulator activity	GO:0051234//establishment of localization;GO:0045184//establishment of protein localization;GO:0044699//single-organism process;GO:0016192//vesicle-mediated transport;GO:0015031//protein transport;GO:0008283//cell proliferation;GO:0002376//immune system process;GO:0008150//biological_process;GO:0006810//transport;GO:0008104//protein localization;GO:0071702//organic substance transport;GO:0051179//localization;GO:0033036//macromolecule localization
FJX1	4.620889924	5.72506606	5.172977992	1.129938699	0.001013262	0.045674822	Up	ENSG00000179431	819	10.261352732	38.743914672	24.688814674	1870	25.894225954	103.994449608	62.301507647	1295	18.255191274	73.438810164	43.921990204	FJX1	four-jointed box kinase 1 [Source:HGNC Symbol;Acc:HGNC:17166]	-	-	-	-	GO:0005615//extracellular space;GO:0044421//extracellular region part;GO:0005575//cellular_component;GO:0005576//extracellular region	-	GO:0008150//biological_process;GO:0009653//anatomical structure morphogenesis;GO:0007154//cell communication;GO:0023052//signaling;GO:0044763//single-organism cellular process;GO:0044699//single-organism process;GO:0032502//developmental process;GO:0048856//anatomical structure development;GO:0007267//cell-cell signaling;GO:0009987//cellular process;GO:0044700//single organism signaling;GO:0048646//anatomical structure formation involved in morphogenesis

id	CON_Mean	KD_Mean	AllMean	log2FoldChange	pvalue	qvalue	State	gene_id	XF_1_count	XF_1_FPKM	XF_1_TPM	XF_1_CPM	XF_2_count	XF_2_FPKM	XF_2_TPM	XF_2_CPM	XF_3_count	XF_3_FPKM	XF_3_TPM	XF_3_CPM	Symbol	Description	KEGG_A_class	KEGG_B_class	Pathway	K_ID	GO Component	GO Function	GO Process
RUNX1	5.915624459	6.948574663	6.432099561	1.067224581	0.00101458	0.045674822	Up	ENSG00000159216	2014	3.898303374	14.718871597	60.712176743	4535	9.701392393	38.962005046	151.089485121	2918	6.354733754	25.564458834	98.968623487	RUNX1	RUNX family transcription factor 1 [Source:HGNC Symbol;Acc:HGNC:10471]	Human Diseases	Cancers	ko05200//Pathways in cancer;ko05202//Transcriptional misregulation in cancers;ko05220//Chronic myeloid leukemia;ko05221//Acute myeloid leukemia	K08367	GO:0044428//nuclear part;GO:0000228//nuclear chromosome;GO:0005654//nucleoplasm;GO:0043228//non-membrane-bounded organelle;GO:0043226//organelle;GO:0044422//organelle part;GO:0043227//membrane-bounded organelle;GO:0070013//intracellular organelle lumen;GO:0043229//intracellular organelle;GO:0031974//membrane-enclosed lumen;GO:0044446//intracellular organelle part;GO:0043232//intracellular non-membrane-bounded organelle;GO:0031981//nuclear lumen;GO:0044464//cell part;GO:0005575//cellular_component;GO:0032991//macromolecular complex;GO:0043233//organelle lumen;GO:0005623//cell;GO:0005634//nucleus;GO:0005622//intracellular;GO:0043231//intracellular membrane-bounded organelle;GO:0005694//chromosome;GO:0044424//intracellular part	GO:0003700//transcription factor activity, sequence-specific DNA binding;GO:0097159//organic cyclic compound binding;GO:1901363//heterocyclic compound binding;GO:0001071//nucleic acid binding transcription factor activity;GO:0003677//DNA binding;GO:0003674//molecular_function;GO:0043167//ion binding;GO:0003676//nucleic acid binding;GO:0005488//binding	GO:0044700//single organism signaling;GO:0044767//single-organism developmental process;GO:0007165//signal transduction;GO:0051716//cellular response to stimulus;GO:0050794//regulation of cellular process;GO:0034641//cellular nitrogen compound metabolic process;GO:0008152//metabolic process;GO:0048856//anatomical structure development;GO:0071840//cellular component organization or biogenesis;GO:0022607//cellular component assembly;GO:0009987//cellular process;GO:0065007//biological regulation;GO:0044763//single-organism cellular process;GO:0050789//regulation of biological process;GO:0007155//cell adhesion;GO:0007267//cell-cell signaling;GO:0008150//biological_process;GO:0007154//cell communication;GO:0009653//anatomical structure morphogenesis;GO:0044699//single-organism process;GO:0032502//developmental process;GO:0009058//biosynthetic process;GO:0023052//signaling;GO:0048869//cellular developmental [illegible]

id	CON_Mean	KD_Mean	AllMean	log2FoldChange	pvalue	qvalue	State	gene_id	XF_1_count	XF_1_FPKM	XF_1_TPM	XF_1_CPM	XF_2_count	XF_2_FPKM	XF_2_TPM	XF_2_CPM	XF_3_count	XF_3_FPKM	XF_3_TPM	XF_3_CPM	Symbol	Description	KEGG_A_class	KEGG_B_class	Pathway	K_ID	GO Component	GO Function	GO Process
LYPD1	1.859359499	3.777813691	2.818586595	2.022781685	0.001025858	0.045818769	Up	ENSG00000150551	118	0.893973008	3.375384789	3.557118598	561	4.697273761	18.8648838836	18.6904522294	287	2.446359059	9.841458016	9.734062694	LYPD1	LY6/PLAUR domain containing 1 [Source:HGNC Symbol;Acc:HGNC:28431]	-	-	-	-	GO:0071944//cell periphery;GO:0005575//cellular_component;GO:0005576//extracellular region;GO:0005623//cell;GO:0016020//membrane;GO:0005886//plasma membrane;GO:0044464//cell part	GO:0003674//molecular_function	GO:0050896//response to stimulus;GO:0051716//cellular response to stimulus;GO:0009987//cellular process;GO:0044700//single organism signaling;GO:0007267//cell-cell signaling;GO:0050789//regulation of biological process;GO:0044699//single-organism process;GO:0044763//single-organism cellular process;GO:0008150//biological_process;GO:0023052//signaling;GO:0007165//signal transduction;GO:0007154//cell communication;GO:0006950//response to stress;GO:0065007//biological regulation;GO:0050794//regulation of cellular process

id	CON_Mean	KD_Mean	AllMean	log2FoldChange	pvalue	qvalue	State	gene_id	XF_1_count	XF_1_FPKM	XF_1_TPM	XF_1_CPM	XF_2_count	XF_2_FPKM	XF_2_TPM	XF_2_CPM	XF_3_count	XF_3_FPKM	XF_3_TPM	XF_3_CPM	Symbol	Description	KEGG_A_class	KEGG_B_class	Pathway	K_ID	GO Component	GO Function	GO Process
PLXNB3	5.205702633	6.283850208	5.744776421	1.117653483	0.001026311	0.045818769	Up	ENSG00000198753	1230	5.291628317	19.97966558 2	37.07843962	1833	8.71539946	35.00213412 8	61.06880401 9	2870	13.89191193 6	55.88577343 4	97.34062693 9	PLXNB3	plexin B3 [Source:HGNC Symbol;Acc:HGNC:9105]	Organismal Systems	Development	ko04360//Axon guidance	K06821	GO:0044464//cell part;GO:0005575//cellular_component;GO:0016020//membrane;GO:0032991//macromolecular complex;GO:0005886//plasma membrane;GO:0071944//cell periphery;GO:0005623//cell	GO:0003674//molecular_function	GO:0044699//single-organism process;GO:0022610//biological adhesion;GO:0032989//cellular component morphogenesis;GO:0009987//cellular process;GO:0000902//cell morphogenesis;GO:0044763//single-organism cellular process;GO:0023052//signaling;GO:0048869//cellular developmental process;GO:0040011//locomotion;GO:0016043//cellular component organization;GO:0051179//localization;GO:0065007//biological regulation;GO:0050789//regulation of biological process;GO:0044767//single-organism developmental process;GO:0050896//response to stimulus;GO:0048870//cell motility;GO:0009653//anatomical structure morphogenesis;GO:0008150//biological_process;GO:0007154//cell communication;GO:0007165//signal transduction;GO:0022607//cellular component assembly;GO:0044700//single organism signaling;GO:0032502//developmental process;GO:0008283//cell proliferation;GO:0044085//cellular component biogenesis;GO:0007155//cell adhesion;GO:0051674//localization of cell;GO:0050794//regulatio[illegible]

id	CON_Mean	KD_Mean	AllMean	log2FoldChange	pvalue	qvalue	State	gene_id	XF_1_count	XF_1_FPKM	XF_1_TPM	XF_1_CPM	XF_2_count	XF_2_FPKM	XF_2_TPM	XF_2_CPM	XF_3_count	XF_3_FPKM	XF_3_TPM	XF_3_CPM	Symbol	Description	KEGG_A_class	KEGG_B_class	Pathway	K_ID	GO Component	GO Function	GO Process
ITGB2	0.268581619	2.349620437	1.309101028	2.171850063	0.001033186	0.045906819	Up	ENSG00000160255	37	0.162377012	0.613088865	1.115367696	154	0.746937312	2.999793651	5.130712394	141	0.696206123	2.800767658	4.78223986	ITGB2	integrin subunit beta 2 [Source:HGNC Symbol;Acc:HGNC:6155]	Cellular Processes;Human Diseases;Environmental Information Processing; Organismal Systems	Immune system;Infectious diseases;Signal transduction; Cardiovascular diseases;Immune diseases;Signaling molecules and interaction;Transport and catabolism;Cell motility	ko04650//Natural killer cell mediated cytotoxicity;ko05166//HTLV-I infection; ko04145//Phagosome;ko05152//Tuberculosis;ko04514//Cell adhesion molecules (CAMs); ko04810//Regulation of [illegible]	K06464	GO:0005575//cellular_component;GO:0044421//extracellular region part;GO:0097708//intracellular vesicle;GO:0044444//cytoplasmic part;GO:0005623//cell;GO:0044424//intracellular part;GO:0032991//macromolecular complex;GO:0031410//cytoplasmic vesicle;GO:0043226//organelle; GO:0071944//cell periphery;GO:0031982//vesicle; GO:0044464//cell part;GO:0005737//cytoplasm;GO:0016020//membrane;GO:0005886//plasma membrane;GO:0005622//intracellular;GO:0005576//extracellular region;GO:0043227//membrane-bounded organelle;GO:0005615//extracellular space	GO:0005515//protein binding;GO:0019899//enzyme binding;GO:0003674//molecular_function; GO:0043167//ion binding;GO:0005488//binding	GO:0006807//nitrogen compound metabolic process;GO:0006950//response to stress;GO:0023052//signaling;GO:0044260//cellular macromolecule metabolic process;GO:0008150//biological_process;GO:0040011//locomotion;GO:0009653//anatomical structure morphogenesis;GO:0050794//regulation of cellular process;GO:0048870//cell motility;GO:0048856//anatomical structure development;GO:0065007//biological regulation;GO:0007165//signal transduction;GO:0048869//cellular developmental process;GO:0007155//cell adhesion;GO:0007568//aging;GO:0044237//cellular metabolic process;GO:0044267//cellular protein metabolic process;GO:0043412//macromolecule modification;GO:0009790//embryo development;GO:0044238//primary metabolic process;GO:0019538//protein metabolic process;GO:0051179//localization;GO:0015031//protein transport;GO:0016192//vesicle-mediated transport;GO:0032501//multicellular organismal process;GO:0002376//immune system process;GO:0006464//cellular protein modification process;GO:0022610//biolo[illegible]
CYP2D8P	-2.01709	0.1308887	-0.9431	2.708615	0.001048268	0.046322636	Up	ENSG00000226450	5	0.101157963	0.381943353	0.150725364	32	0.715518158	2.873610402	1.066122056	27	0.614596012	2.472458338	0.915748058	-	-	-	-	-	-	-	-	-

id	CON_Mean	KD_Mean	AllMean	log2FoldChange	pvalue	qvalue	State	gene_id	XF_1_count	XF_1_FPKM	XF_1_TPM	XF_1_CPM	XF_2_count	XF_2_FPKM	XF_2_TPM	XF_2_CPM	XF_3_count	XF_3_FPKM	XF_3_TPM	XF_3_CPM	Symbol	Description	KEGG_A_class	KEGG_B_class	Pathway	K_ID	GO Component	GO Function	GO Process
PTGES	4.572721651	3.164020071	3.868370861	-1.41385	0.001050689	0.046322636	Down	ENSG00000148344	792	12.625540828	47.670408504	23.874897706	243	4.2812609	17.194079164	8.095864363	281	5.039959347	20.275252786	9.530563125	PTGES	prostaglandin E synthase [Source:HGNC Symbol;Acc:HGNC:9599]	Metabolism	Lipid metabolism	ko00590//Arachidonic acid metabolism	K15729	GO:0005739//mitochondrion;GO:0044424//intracellular part;GO:0044422//organelle part;GO:0012505//endomembrane system;GO:0044464//cell part;GO:0005622//intracellular;GO:0031975//envelope;GO:0043226//organelle;GO:0043227//membrane-bounded organelle;GO:0005575//cellular_component;GO:0043229//intracellular organelle;GO:0005634//nucleus;GO:0044444//cytoplasmic part;GO:0031967//organelle envelope;GO:0044446//intracellular organelle part;GO:0005635//nuclear envelope;GO:0005737//cytoplasm;GO:0005623//cell;GO:0044428//nuclear part;GO:0043231//intracellular membrane-bounded organelle	GO:0016740//transferase activity;GO:0016853//isomerase activity;GO:0016765//transferase activity, transferring alkyl or aryl (other than methyl) groups;GO:0043167//ion binding;GO:0003824//catalytic activity;GO:0003674//molecular_function;GO:0005488//binding	GO:0050794//regulation of cellular process;GO:0044281//small molecule metabolic process;GO:0050789//regulation of biological process;GO:0006950//response to stress;GO:0008150//biological_process;GO:0051186//cofactor metabolic process;GO:0044238//primary metabolic process;GO:0008283//cell proliferation;GO:0008152//metabolic process;GO:0051716//cellular response to stimulus;GO:0034641//cellular nitrogen compound metabolic process;GO:0044710//single-organism metabolic process;GO:0006629//lipid metabolic process;GO:0065007//biological regulation;GO:0007165//signal transduction;GO:0071704//organic substance metabolic process;GO:0044699//single-organism process;GO:0006807//nitrogen compound metabolic process;GO:0044237//cellular metabolic process;GO:0050896//response to stimulus;GO:0023052//signaling;GO:0044700//single organism signaling;GO:0009987//cellular process;GO:0044763//single-organism cellular process;GO:0007154//cell [illegible]

id	CON_Mean	KD_Mean	AllMean	log2FoldChange	pvalue	qvalue	State	gene_id	XF_1_count	XF_1_FPKM	XF_1_TPM	XF_1_CPM	XF_2_count	XF_2_FPKM	XF_2_TPM	XF_2_CPM	XF_3_count	XF_3_FPKM	XF_3_TPM	XF_3_CPM	Symbol	Description	KEGG_A_class	KEGG_B_class	Pathway	K_ID	GO Component	GO Function	GO Process
MRGPRX4	0.405250512	2.488416999	1.446833755	2.191934269	0.001064615	0.046695275	Up	ENSG00000179817	41	0.855919659	3.231706296	1.235947987	134	3.091680131	12.416574034	4.464386109	197	4.627125461	18.61446332	6.681569166	MRGPRX4	MAS related GPR family member X4 [Source:HGNC Symbol;Acc:HGNC:17617]	-	-	-	-	GO:0016020//membrane;GO:0005575//cellular_component;GO:0005886//plasma membrane;GO:0071944//cell periphery;GO:0005623//cell;GO:0044464//cell part	GO:0003674//molecular_function	GO:0008150//biological_process;GO:0044700//single organism signaling;GO:0044699//single-organism process;GO:0050896//response to stimulus;GO:0009987//cellular process;GO:0050789//regulation of biological process;GO:0044763//single-organism cellular process;GO:0007165//signal transduction;GO:0050794//regulation of cellular process;GO:0051716//cellular response to stimulus;GO:0007154//cell communication;GO:0065007//biological regulation;GO:0023052//signaling
DGCR8	4.645246947	5.702698617	5.173972782	1.070846047	0.00107696	0.047115608	Up	ENSG00000128191	833	3.432788201	12.961220283	25.110845694	1739	7.920310387	31.80895698	57.93707048	1350	6.259385224	25.18088127	45.787402915	DGCR8	DGCR8 microprocessor complex subunit [Source:HGNC Symbol;Acc:HGNC:2847]	-	-	-	-	GO:0005654//nucleoplasm;GO:0043232//intracellular non-membrane-bounded organelle;GO:0005730//nucleolus;GO:0005737//cytoplasm;GO:0044428//nuclear part;GO:0043229//intracellular organelle;GO:0044422//organelle part;GO:0044446//intracellular organelle part;GO:0005634//nucleus;GO:0031981//nuclear lumen;GO:0043233//organelle lumen;GO:0043226//organelle;GO:0032991//macromolecular complex;GO:0043228//non-membrane-bounded organelle;GO:0043227//membrane-bounded organelle;GO:0044464//cell part;GO:0031974//membrane-enclosed lumen;GO:0044424//intracellular part;GO:0005575//cellular_component;GO:0043231//intracellular membrane-bounded organelle;GO:0005622//intracellular;GO:0005623//cell;GO:0070013//intracellular organelle lumen	GO:0097159//organic cyclic compound binding;GO:0005488//binding;GO:0003723//RNA binding;GO:0003676//nucleic acid binding;GO:0003824//catalytic activity;GO:1901363//heterocyclic compound binding;GO:0004518//nuclease activity;GO:0003674//molecular_function;GO:0043167//ion binding;GO:0016788//hydrolase activity, acting on ester bonds;GO:0016787//hydrolase activity	GO:0044237//cellular metabolic process;GO:0009987//cellular process;GO:0006807//nitrogen compound metabolic process;GO:0008152//metabolic process;GO:0034641//cellular nitrogen compound metabolic process;GO:0008150//biological_process

id	CON_Mean	KD_Mean	AllMean	log2FoldChange	pvalue	qvalue	State	gene_id	XF_1_count	XF_1_FPKM	XF_1_TPM	XF_1_CPM	XF_2_count	XF_2_FPKM	XF_2_TPM	XF_2_CPM	XF_3_count	XF_3_FPKM	XF_3_TPM	XF_3_CPM	Symbol	Description	KEGG_A_class	KEGG_B_class	Pathway	K_ID	GO Component	GO Function	GO Process
NME3	5.549628932	4.516838339	5.033233635	-1.03419	0.001080046	0.047129769	Down	ENSG00000103024	1562	37.911919332	143.144496245	47.08660381	644	17.275125907	69.379065965	21.455706377	701	19.14294116	77.010139242	23.775532921	NME3	NME/NM23 nucleoside diphosphate kinase 3 [Source:HGNC Symbol;Acc:HGNC:7851]	Metabolism	Nucleotide metabolism	ko00230//Purine metabolism;ko00240//Pyrimidine metabolism	K00940	GO:0005829//cytosol;GO:0044424//intracellular part;GO:0005737//cytoplasm;GO:0044464//cell part;GO:0005623//cell;GO:0044444//cytoplasmic part;GO:0005622//intracellular;GO:0005575//cellular_component	GO:0016301//kinase activity;GO:0043167//ion binding;GO:0003824//catalytic activity;GO:0003674//molecular_function;GO:0016740//transferase activity;GO:0005488//binding;GO:0016772//transferase activity, transferring phosphorus-containing groups	GO:0009987//cellular process;GO:0008150//biological_process;GO:0044699//single-organism process;GO:0044237//cellular metabolic process;GO:0006807//nitrogen compound metabolic process;GO:0044710//single-organism metabolic process;GO:0034641//cellular nitrogen compound metabolic process;GO:0008152//metabolic process;GO:0009058//biosynthetic process;GO:0008219//cell death;GO:0044763//single-organism cellular process;GO:0044281//small molecule metabolic process
PROCR	5.545937417	4.487004023	5.01647072	-1.05429	0.001087143	0.047318448	Down	ENSG00000101000	1558	27.675912504	104.496280412	46.966023519	734	14.410238456	57.873319695	24.454174659	590	11.791862642	47.437484992	20.010790904	PROCR	protein C receptor [Source:HGNC Symbol;Acc:HGNC:9452]	-	-	-	-	GO:0043228//non-membrane-bounded organelle;GO:0005815//microtubule organizing center;GO:0044464//cell part;GO:0071944//cell periphery;GO:0005622//intracellular;GO:0015630//microtubule cytoskeleton;GO:0005575//cellular_component;GO:0043229//intracellular organelle;GO:0044446//intracellular organelle part;GO:0016020//membrane;GO:0005623//cell;GO:0043232//intracellular non-membrane-bounded organelle;GO:0044430//cytoskeletal part;GO:0005737//cytoplasm;GO:0044421//extracellular region part;GO:0005886//plasma membrane;GO:0044424//intracellular part;GO:0005615//extracellular space;GO:0043226//organelle;GO:0005856//cytoskeleton;GO:0005576//extracellular region;GO:0044422//organelle part	GO:0003674//molecular_function	GO:0006950//response to stress;GO:0050896//response to stimulus;GO:0008150//biological_process

id	CON_Mean	KD_Mean	AllMean	log2FoldChange	pvalue	qvalue	State	gene_id	XF_1_count	XF_1_FPKM	XF_1_TPM	XF_1_CPM	XF_2_count	XF_2_FPKM	XF_2_TPM	XF_2_CPM	XF_3_count	XF_3_FPKM	XF_3_TPM	XF_3_CPM	Symbol	Description	KEGG_A_class	KEGG_B_class	Pathway	K_ID	GO Component	GO Function	GO Process
CXXC1	4.900712099	5.900824997	5.400768548	1.00962274	0.001093026	0.047453473	Up	ENSG00000154832	995	7.782653736	29.385060638	29.994347497	1954	16.891561506	67.838623364	65.100078043	1582	13.922172522	56.007508744	53.656052898	CXXC1	CXXC finger protein 1 [Source:HGNC Symbol;Acc:HGNC:24343]	-	-	-	-	GO:0031974//membrane-enclosed lumen;GO:0005829//cytosol;GO:0005623//cell;GO:0044424//intracellular part;GO:0043226//organelle;GO:0031981//nuclear lumen;GO:0044464//cell part;GO:0005654//nucleoplasm;GO:0043231//intracellular membrane-bounded organelle;GO:0043229//intracellular organelle;GO:0005575//cellular_component;GO:0043233//organelle lumen;GO:0044428//nuclear part;GO:0044444//cytoplasmic part;GO:0043227//membrane-bounded organelle;GO:0044446//intracellular organelle part;GO:0005737//cytoplasm;GO:0005622//intracellular;GO:0005634//nucleus;GO:0044422//organelle part;GO:0070013//intracellular organelle lumen;GO:0032991//macromolecular complex	GO:0008168//methyltransferase activity;GO:1901363//heterocyclic compound binding;GO:0005488//binding;GO:0003676//nucleic acid binding;GO:0003677//DNA binding;GO:0016740//transferase activity;GO:0043167//ion binding;GO:0005515//protein binding;GO:0097159//organic cyclic compound binding;GO:0042393//histone binding;GO:0003674//molecular_function;GO:0016741//transferase activity, transferring one-carbon groups;GO:0003824//catalytic activity	GO:0034641//cellular nitrogen compound metabolic process;GO:0043170//macromolecule metabolic process;GO:0044699//single-organism process;GO:0006950//response to stress;GO:0006464//cellular protein modification process;GO:0071704//organic substance metabolic process;GO:0050789//regulation of biological process;GO:0043412//macromolecule modification;GO:0050896//response to stimulus;GO:0008150//biological_process;GO:0008152//metabolic process;GO:0006996//organelle organization;GO:0065007//biological regulation;GO:0051276//chromosome organization;GO:0044238//primary metabolic process;GO:0036211//protein modification process;GO:0023052//signaling;GO:0007165//signal transduction;GO:0009058//biosynthetic process;GO:0050794//regulation of cellular process;GO:0044260//cellular macromolecule metabolic process;GO:0009987//cellular process;GO:0016043//cellular component organization;GO:0051716//cellular response to stimulus;GO:0007154//cell [illegible]
LINC00173	1.495960085	-0.38814	0.553908417	-1.99724	0.001104687	0.047785531	Down	ENSG00000196668	91	1.052244584	3.97297271	2.74320163	16	0.204472968	0.82118901	0.533061028	24	0.312235625	1.256092716	0.813998274	-	-	-	-	-	-	-	-	-

id	CON_Mean	KD_Mean	AllMean	log2FoldChange	pvalue	qvalue	State	gene_id	XF_1_count	XF_1_FPKM	XF_1_TPM	XF_1_CPM	XF_2_count	XF_2_FPKM	XF_2_TPM	XF_2_CPM	XF_3_count	XF_3_FPKM	XF_3_TPM	XF_3_CPM	Symbol	Description	KEGG_A_class	KEGG_B_class	Pathway	K_ID	GO Component	GO Function	GO Process
DDX51	4.588957091	5.638937965	5.113947528	1.059180098	0.001106276	0.047785531	Up	ENSG00000185163	801	3.803150632	14.35960229	24.1462033 62	1618	8.490438881	34.0986643 09	53.9057964 56	1328	7.094225521	28.5393603 09	45.0412378 31	DDX51	DEAD-box helicase 51 [Source:HGNC Symbol;Acc:HGNC:20082]	-	-	-	-	GO:0043228//non-membrane-bounded organelle;GO:0043227//membrane-bounded organelle;GO:0043226//organelle;GO:0005622//intracellular;GO:0005575//cellular_component;GO:0044424//intracellular part;GO:0031974//membrane-enclosed lumen;GO:0070013//intracellular organelle lumen;GO:0005730//nucleolus;GO:0005634//nucleus;GO:0044422//organelle part;GO:0043229//intracellular organelle;GO:0031981//nuclear lumen;GO:0043231//intracellular membrane-bounded organelle;GO:0043233//organelle lumen;GO:0044464//cell part;GO:0005623//cell;GO:0044446//intracellular organelle part;GO:0043232//intracellular non-membrane-bounded organelle;GO:0044428//nuclear part	GO:1901363//heterocyclic compound binding;GO:0003723//RNA binding;GO:0017111//nucleoside-triphosphatase activity;GO:0016887//ATPase activity;GO:0004386//helicase activity;GO:0043167//ion binding;GO:0003674//molecular_function;GO:0016817//hydrolase activity, acting on acid anhydrides;GO:0016787//hydrolase activity;GO:0003676//nucleic acid binding;GO:0003824//catalytic activity;GO:0016462//pyrophosphatase activity;GO:0016818//hydrolase activity, acting on acid anhydrides, in phosphorus-containing anhydrides;GO:0005488//binding;GO:0097159//organic cyclic compound binding	GO:0009987//cellular process;GO:0008150//biological_process;GO:0044085//cellular component biogenesis;GO:0008152//metabolic process;GO:0006807//nitrogen compound metabolic process;GO:0034641//cellular nitrogen compound metabolic process;GO:0022613//ribonucleoprotein complex biogenesis;GO:0071840//cellular component organization or biogenesis;GO:0044237//cellular metabolic process;GO:0042254//ribosome biogenesis
HOTAIRM1	1.417324871	-0.54029	0.438518615	-2.06652	0.00111017	0.047832628	Down	ENSG00000233429	86	1.732938681	6.543077713	2.592476266	13	0.289513426	1.162722123	0.433112085	23	0.521444973	2.097721015	0.780081679	-	-	-	-	-	-	-	-	-

id	CON_Mean	KD_Mean	AllMean	log2FoldChange	pvalue	qvalue	State	gene_id	XF_1_count	XF_1_FPKM	XF_1_TPM	XF_1_CPM	XF_2_count	XF_2_FPKM	XF_2_TPM	XF_2_CPM	XF_3_count	XF_3_FPKM	XF_3_TPM	XF_3_CPM	Symbol	Description	KEGG_A_class	KEGG_B_class	Pathway	K_ID	GO Component	GO Function	GO Process
SLC2A1	7.155481171	8.21253331	7.684007241	1.101277076	0.001119229	0.048101474	Up	ENSG00000117394	4761	21.69953007 2	81.9311803 68	143.520691895	11254	56.689114086	227.670571365	374.941800564	6788	34.808866825	140.032592617	230.225845178	SLC2A1	solute carrier family 2 member 1 [Source:HGNC Symbol;Acc:HGNC:11005]	Organismal Systems;Human Diseases;Environmental Information Processing	Digestive system;Infectious diseases;Signal transduction;Endocrine and metabolic diseases;Endocrine system;Cancers	ko05200//Pathways in cancer;ko05166//HTLV-I infection;ko04919//Thyroid hormone signaling pathway;ko04931//Insulin resistance;ko04922//Glucagon signaling pathway;ko04066//HIF-1 [illegible]	K07299	GO:0043228//non-membrane-bounded organelle;GO:0043227//membrane-bounded organelle;GO:0012505//endomembrane system;GO:0044421//extracellular region part;GO:0005634//nucleus;GO:0005575//cellular_component;GO:0005886//plasma membrane;GO:0031410//cytoplasmic vesicle;GO:0044424//intracellular part;GO:0005737//cytoplasm;GO:0005856//cytoskeleton;GO:0043232//intracellular non-membrane-bounded organelle;GO:0016020//membrane;GO:0005622//intracellular;GO:0005576//extracellular region;GO:0097708//intracellular vesicle;GO:0031982//vesicle;GO:0005623//cell;GO:0005794//Golgi apparatus;GO:0043229//intracellular organelle;GO:0044444//cytoplasmic part;GO:0005615//extracellular space;GO:0005829//cytosol;GO:0044464//cell part;GO:0071944//cell periphery;GO:0043231//intracellular membrane-bounded organelle;GO:0043226//organelle	GO:0022857//transmembrane transporter activity;GO:0005215//transporter activity;GO:0003674//molecular_function;GO:0005515//protein binding;GO:0005488//binding;GO:0019899//enzyme binding	GO:0044238//primary metabolic process;GO:0044699//single-organism process;GO:0071840//cellular component organization or biogenesis;GO:0065003//macromolecular complex assembly;GO:0044710//single-organism metabolic process;GO:0044281//small molecule metabolic process;GO:0051234//establishment of localization;GO:0033036//macromolecule localization;GO:0009987//cellular process;GO:0008152//metabolic process;GO:0032502//developmental process;GO:0044763//single-organism cellular process;GO:0071704//organic substance metabolic process;GO:0000003//reproduction;GO:0051179//localization;GO:0016043//cellular component organization;GO:0043933//macromolecular complex subunit organization;GO:0009058//biosynthetic process;GO:0048856//anatomical structure development;GO:0007267//cell-cell signaling;GO:0023052//signaling;GO:0006950//response to stress;GO:0044237//cellular metabolic process;GO:0045184//establishment of protein localization;GO:0050896//r

id	CON_Mean	KD_Mean	AllMean	log2FoldChange	pvalue	qvalue	State	gene_id	XF_1_count	XF_1_FPKM	XF_1_TPM	XF_1_CPM	XF_2_count	XF_2_FPKM	XF_2_TPM	XF_2_CPM	XF_3_count	XF_3_FPKM	XF_3_TPM	XF_3_CPM	Symbol	Description	KEGG_A_class	KEGG_B_class	Pathway	K_ID	GO Component	GO Function	GO Process
RELT	4.049249575	5.208189795	4.628719685	1.171583348	0.0011287	0.0482653455	Up	ENSG00000054967	550	2.67977858	10.118072667	16.5797900 74	1215	6.542641315	26.276065702	40.479321813	972	5.328419282	21.435698278	32.966930099	RELT	RELT TNF receptor [Source:HGNC Symbol;Acc:HGNC:13764]	Environmental Information Processing	Signaling molecules and interaction	ko04060//Cytokine-cytokine receptor interaction	K05156	GO:0070013//intracellular organelle lumen;GO:0044424//intracellular part;GO:0044464//cell part;GO:0005622//intracellular;GO:0005737//cytoplasm;GO:0005623//cell;GO:0031981//nuclear lumen;GO:0043226//organelle;GO:0044428//nuclear part;GO:0005634//nucleus;GO:0005575//cellular_component;GO:0044446//intracellular organelle part;GO:0031974//membrane-enclosed lumen;GO:0043233//organelle lumen;GO:0071944//cell periphery;GO:0044422//organelle part;GO:0016020//membrane;GO:0043231//intracellular membrane-bounded organelle;GO:0043229//intracellular organelle;GO:0043227//membrane-bounded organelle;GO:0005654//nucleoplasm;GO:0005886//plasma membrane	GO:0003674//molecular_function	GO:0048646//anatomical structure formation involved in morphogenesis;GO:0044699//single-organism process;GO:0009653//anatomical structure morphogenesis;GO:0008219//cell death;GO:0032502//developmental process;GO:0048856//anatomical structure development;GO:0009987//cellular process;GO:0044763//single-organism cellular process;GO:0008150//biological_process
ADIRF-AS1	4.658982816	3.175149779	3.917066297	-1.45872	0.001143167	0.0484128 05	Down	ENSG00000272734	841	4.410578684	16.653075735	25.352006277	212	1.228785425	4.93495593	7.063058621	327	1.929493125	7.762158019	11.090726484	-	-	-	-	-	-	-	-	-
ART3	5.299670426	4.203716255	4.75169334	-1.09895	0.001145525	0.0484128 05	Down	ENSG00000156219	1313	12.0708998 68	45.576243865	39.580480667	524	5.324107553	21.382281734	17.457748667	557	5.761373369	23.177429302	18.891543277	ART3	ADP-ribosyltransferase 3 [Source:HGNC Symbol;Acc:HGNC:725]	-	-	-	-	GO:0071944//cell periphery;GO:0005576//extracellular region;GO:0005886//plasma membrane;GO:0005575//cellular_component;GO:0005615//extracellular space;GO:0043226//organelle;GO:0005623//cell;GO:0044421//extracellular region part;GO:0044464//cell part;GO:0016020//membrane	GO:0003674//molecular_function;GO:0003824//catalytic activity;GO:0016757//transferase activity, transferring glycosyl groups;GO:0016740//transferase activity	GO:0006464//cellular protein modification process;GO:0044238//primary metabolic process;GO:0008152//metabolic process;GO:0071704//organic substance metabolic process;GO:0043170//macromolecule metabolic process;GO:0036211//protein modification process;GO:0019538//protein metabolic process;GO:0009987//cellular process;GO:0043412//macromolecule modification;GO:0044267//cellular protein metabolic process;GO:0044237//cellular metabolic process;GO:0044260//cellular macromolecule metabolic process;GO:0008150//biological_process

id	CON_Mean	KD_Mean	AllMean	log2FoldChange	pvalue	qvalue	State	gene_id	XF_1_count	XF_1_FPKM	XF_1_TPM	XF_1_CPM	XF_2_count	XF_2_FPKM	XF_2_TPM	XF_2_CPM	XF_3_count	XF_3_FPKM	XF_3_TPM	XF_3_CPM	Symbol	Description	KEGG_A_class	KEGG_B_class	Pathway	K_ID	GO Component	GO Function	GO Process
SLC26A6	4.741990171	5.770956679	5.256473425	1.040310817	0.001146335	0.048412805	Up	ENSG000002256977	891	4.214539451	15.91288801	26.85925992	1446	7.559295529	30.3590761 63	48.1753904 05	1785	9.499626806	38.2160492 96	60.5411216 33	SLC26A6	solute carrier family 26 member 6 [Source:HGNC Symbol;Acc:HGNC:14472]	Organismal Systems	Digestive system	ko04978//Mineral absorption	K14704	GO:0005737//cytoplasm;GO:0044424//intracellular part;GO:0005623//cell;GO:0012505//endomembrane system;GO:0005783//endoplasmic reticulum;GO:0005622//intracellular;GO:0044444//cytoplasmic part;GO:0043226//organelle;GO:0044464//cell part;GO:0043229//intracellular organelle;GO:0005575//cellular_component;GO:0043227//membrane-bounded organelle;GO:0031982//vesicle;GO:0016020//membrane;GO:0005886//plasma membrane;GO:0097708//intracellular vesicle;GO:0005929//cilium;GO:0032991//macromolecular complex;GO:0042995//cell projection;GO:0031410//cytoplasmic vesicle;GO:0071944//cell periphery;GO:0043231//intracellular membrane-bounded organelle	GO:0005215//transporter activity;GO:0022857//transmembrane transporter activity;GO:0003674//molecular_function	GO:0008150//biological_process;GO:0065007//biological regulation;GO:0009987//cellular process;GO:0006950//response to stress;GO:0030154//cell differentiation;GO:0051234//establishment of localization;GO:0048869//cellular developmental process;GO:0050896//response to stimulus;GO:0044699//single-organism process;GO:0006810//transport;GO:0032502//developmental process;GO:0021700//developmental maturation;GO:0000003//reproduction;GO:0002376//immune system process;GO:0044767//single-organism developmental process;GO:0051179//localization;GO:0048856//anatomical structure development;GO:0055085//transmembrane transport;GO:0042592//homeostatic process;GO:0065008//regulation of biological quality;GO:0044763//single-organism cellular process

id	CON_Mean	KD_Mean	AllMean	log2FoldChange	pvalue	qvalue	State	gene_id	XF_1_count	XF_1_FPKM	XF_1_TPM	XF_1_CPM	XF_2_count	XF_2_FPKM	XF_2_TPM	XF_2_CPM	XF_3_count	XF_3_FPKM	XF_3_TPM	XF_3_CPM	Symbol	Description	KEGG_A_class	KEGG_B_class	Pathway	K_ID	GO Component	GO Function	GO Process
ABCC3	3.172292588	1.389770013	2.281031301	-1.78706	0.001152014	0.048412945	Down	ENSG00000108846	298	0.93429347	3.52762325	8.983231713	60	0.207902117	0.834960901	1.998978855	92	0.32452696	1.305539527	3.120326717	ABCC3	ATP binding cassette subfamily C member 3 [Source:HGNC Symbol;Acc:HGNC:54]	Environmental Information Processing; Organismal Systems	Digestive system; Membrane transport	ko04976//Bile secretion;ko02010//ABC transporters	K05667	GO:0016020//membrane;GO:0005575//cellular_component;GO:0005623//cell;GO:0044464//cell part;GO:0071944//cell periphery;GO:0005886//plasma membrane	GO:0016462//pyrophosphatase activity;GO:0005215//transporter activity;GO:0003824//catalytic activity;GO:0016818//hydrolase activity, acting on acid anhydrides, in phosphorus-containing anhydrides;GO:0017111//nucleoside-triphosphatase activity;GO:0043167//ion binding;GO:0016787//hydrolase activity;GO:0016887//ATPase activity;GO:0016817//hydrolase activity, acting on acid anhydrides;GO:0003674//molecular_function;GO:0022857//transmembrane transporter activity;GO:0005488//binding	GO:0008150//biological_process;GO:0051234//establishment of localization;GO:0006810//transport;GO:0051179//localization;GO:0055085//transmembrane transport

id	CON_Mean	KD_Mean	AllMean	log2FoldChange	pvalue	qvalue	State	gene_id	XF_1_count	XF_1_FPKM	XF_1_TPM	XF_1_CPM	XF_2_count	XF_2_FPKM	XF_2_TPM	XF_2_CPM	XF_3_count	XF_3_FPKM	XF_3_TPM	XF_3_CPM	Symbol	Description	KEGG_A_class	KEGG_B_class	Pathway	K_ID	GO Component	GO Function	GO Process
LPAR6	4.534112963	2.627579986	3.580846474	-1.78004	0.00116365	0.048634596	Down	ENSG00000139679	771	5.342954293	20.173457695	23.2418511176	114	0.873117201	3.506547866	3.798059824	281	2.190934052	8.81390874	9.530563125	LPAR6	lysophosphatidic acid receptor 6 [Source:HGNC Symbol;Acc:HGNC:15520]	Environmental Information Processing;Human Diseases	Signaling molecules and interaction;Cancers;Signal transduction	ko05200//Pathways in cancer;ko04151//PI3K-Akt signaling pathway;ko04080//Neuroactive ligand-receptor interaction	K04273	GO:0005886//plasma membrane;GO:0016020//membrane;GO:0044464//cell part;GO:0005623//cell;GO:0071944//cell periphery;GO:0005575//cellular_component	GO:0003674//molecular_function	GO:0048856//anatomical structure development;GO:0007154//cell communication;GO:0044707//single-multicellular organism process;GO:0009790//embryo development;GO:0065007//biological regulation;GO:0008150//biological_process;GO:0044699//single-organism process;GO:0032501//multicellular organismal process;GO:0042592//homeostatic process;GO:0023052//signaling;GO:0050794//regulation of cellular process;GO:0032502//developmental process;GO:0050896//response to stimulus;GO:0050789//regulation of biological process;GO:0009987//cellular process;GO:0007275//multicellular organism development;GO:0051716//cellular response to stimulus;GO:0044767//single-organism developmental process;GO:0044700//single organism signaling;GO:0044763//single-organism cellular process;GO:0007165//signal transduction;GO:0065008//regulation of biological quality
LAMB2P1	0.965486901	-0.91898	0.023252849	-2.06156	0.001165761	0.048634596	Down	ENSG00000270441	62	1.038907458	3.922615562	1.868994517	10	0.18519352	0.743760336	0.333163142	16	0.301648425	1.213501467	0.542665516	-	-	-	-	-	-	-	-	-

id	CON_Mean	KD_Mean	AllMean	log2FoldChange	pvalue	qvalue	State	gene_id	XF_1_count	XF_1_FPKM	XF_1_TPM	XF_1_CPM	XF_2_count	XF_2_FPKM	XF_2_TPM	XF_2_CPM	XF_3_count	XF_3_FPKM	XF_3_TPM	XF_3_CPM	Symbol	Description	KEGG_A_class	KEGG_B_class	Pathway	K_ID	GO Component	GO Function	GO Process
E2F2	3.793112099	5.008800602	4.40095635	1.229414518	0.00116869	0.048634596	Up	ENSG00000007968	460	2.538764833	9.585645345	13.866733516	1058	6.453434726	25.917800889	35.248660476	846	5.253284357	21.133437984	28.69343916	E2F2	E2F transcription factor 2 [Source:HGNC Symbol;Acc:HGNC:3114]	Human Diseases;Cellular Processes	Cancers;Cell growth and death;Infectious diseases	ko05200//Pathways in cancer;ko05166//HTLV-I infection;ko05206//MicroRNAs in cancer;ko05161//Hepatitis B;ko04110//Cell cycle;ko05222//Small cell lung cancer;ko05215//Prostate cancer;ko05[illegible]	K09389	GO:0043227//membrane-bounded organelle;GO:0044424//intracellular part;GO:0044464//cell part;GO:0043233//organelle lumen;GO:0031974//membrane-enclosed lumen;GO:0044428//nuclear part;GO:0005634//nucleus;GO:0043229//intracellular organelle;GO:0031981//nuclear lumen;GO:0043232//intracellular non-membrane-bounded organelle;GO:0005622//intracellular;GO:0005575//cellular_component;GO:0043228//non-membrane-bounded organelle;GO:0005623//cell;GO:0005654//nucleoplasm;GO:0070013//intracellular organelle lumen;GO:0044446//intracellular organelle part;GO:0000228//nuclear chromosome;GO:0032991//macromolecular complex;GO:0044422//organelle part;GO:0043226//organelle;GO:0043231//intracellular membrane-bounded organelle;GO:0005694//chromosome	GO:0003700//transcription factor activity, sequence-specific DNA binding;GO:0003677//DNA binding;GO:0001071//nucleic acid binding transcription factor activity;GO:0008134//transcription factor binding;GO:0097159//organic cyclic compound binding;GO:0005488//binding;GO:0003676//nucleic acid binding;GO:0005515//protein binding;GO:1901363//heterocyclic compound binding;GO:0003674//molecular_function	GO:0009058//biosynthetic process;GO:0050896//response to stimulus;GO:0044700//single organism signaling;GO:0034641//cellular nitrogen compound metabolic process;GO:0006807//nitrogen compound metabolic process;GO:0007154//cell communication;GO:0008152//metabolic process;GO:0065007//biological regulation;GO:0044699//single-organism process;GO:0050789//regulation of biological process;GO:0009653//anatomical structure morphogenesis;GO:0009987//cellular process;GO:0008150//biological_process;GO:0032502//developmental process;GO:0007165//signal transduction;GO:0048856//anatomical structure development;GO:0044763//single-organism cellular process;GO:0044237//cellular metabolic process;GO:0007049//cell cycle;GO:0048646//anatomical structure formation involved in morphogenesis;GO:0023052//signaling;GO:0008219//cell death;GO:0050794//regulation of cellular process;GO:0051716//cellular response to stimulus

id	CON_Mean	KD_Mean	AllMean	log2FoldChange	pvalue	qvalue	State	gene_id	XF_1_count	XF_1_FPKM	XF_1_TPM	XF_1_CPM	XF_2_count	XF_2_FPKM	XF_2_TPM	XF_2_CPM	XF_3_count	XF_3_FPKM	XF_3_TPM	XF_3_CPM	Symbol	Description	KEGG_A_class	KEGG_B_class	Pathway	K_ID	GO Component	GO Function	GO Process
FZD8	4.569088828	5.60500223	5.087045529	1.039394073	0.00117553	0.048800243	Up	ENSG00000177283	790	5.880150015	22.201754134	23.81461	1480	12.174850639	48.895722 68	49.308145089	1385	11.598637958	46.660161399	46.974483732	FZD8	frizzled class receptor 8 [Source:HGNC Symbol;Acc:HGNC:4046]	Organismal Systems;Cellular Processes;Environmental Information Processing;Human Diseases	Signal transduction;Infectious diseases;Cellular community;Cancers;Endocrine system	ko05200//Pathways in cancer;ko05166//HTLV-I infection;ko05205//Proteoglycans in cancer;ko04390//Hippo signaling pathway;ko04550//Signaling pathways regulating pluripotency of stem [illegible]	K02375	GO:0044464//cell part;GO:0032991//macromolecular complex;GO:0071944//cell periphery;GO:0016020//membrane;GO:0012505//endomembrane system;GO:0044424//intracellular part;GO:0005886//plasma membrane;GO:0044444//cytoplasmic part;GO:0043227//membrane-bounded organelle;GO:0043226//organelle;GO:0005794//Golgi apparatus;GO:0005737//cytoplasm;GO:0043231//intracellular membrane-bounded organelle;GO:0043229//intracellular organelle;GO:0005623//cell;GO:0005622//intracellular;GO:0005575//cellular_component	GO:0005515//protein binding;GO:0003674//molecular_function;GO:0005488//binding;GO:0019899//enzyme binding	GO:0007154//cell communication;GO:0044238//primary metabolic process;GO:0044700//single organism signaling;GO:0002376//immune system process;GO:0051716//cellular response to stimulus;GO:0008150//biological_process;GO:0050789//regulation of biological process;GO:0008152//metabolic process;GO:0043412//macromolecule modification;GO:0007267//cell-cell signaling;GO:0044767//single-organism developmental process;GO:0044267//cellular protein metabolic process;GO:0043170//macromolecule metabolic process;GO:0007165//signal transduction;GO:0071704//organic substance metabolic process;GO:0044763//single-organism cellular process;GO:0065007//biological regulation;GO:0032502//developmental process;GO:0044260//cellular macromolecule metabolic process;GO:0006464//cellular protein modification process;GO:0044699//single-organism process;GO:0019538//protein metabolic process;GO:0048869//cellular developmental process;GO:0048646//anat [illegible]

id	CON_Mean	KD_Mean	AllMean	log2FoldChange	pvalue	qvalue	State	gene_id	XF_1_count	XF_1_FPKM	XF_1_TPM	XF_1_CPM	XF_2_count	XF_2_FPKM	XF_2_TPM	XF_2_CPM	XF_3_count	XF_3_FPKM	XF_3_TPM	XF_3_CPM	Symbol	Description	KEGG_A_class	KEGG_B_class	Pathway	K_ID	GO Component	GO Function	GO Process
RRM2	8.0032417130008	9.082786621	8.543014167	1.129399832	0.001189289	0.049251594	Up	ENSG00000171848	8571	28.228277013	106.581849827	258.373419498	20911	76.114656099	305.685977329	696.677447271	12211	45.248064953	182.028443449	414.155538519	RRM2	ribonucleotide reductase regulatory subunit M2 [Source:HGNC Symbol;Acc:HGNC:10452]	Cellular Processes;Metabolism	Metabolism of other amino acids;Cell growth and death;Nucleotide metabolism	ko00230//Purine metabolism;ko00240//Pyrimidine metabolism;ko04115//p53 signaling pathway;ko00480//Glutathione metabolism	K10808	GO:0044464//cell part;GO:0044444//cytoplasmic part;GO:0044424//intracellular part;GO:0005737//cytoplasm;GO:0005829//cytosol;GO:0032991//macromolecular complex;GO:0005623//cell;GO:0005575//cellular_component;GO:0005622//intracellular	GO:0003674//molecular_function;GO:0016491//oxidoreductase activity;GO:0043167//ion binding;GO:0003824//catalytic activity;GO:0005488//binding	GO:0044237//cellular metabolic process;GO:0009987//cellular process;GO:0007049//cell cycle;GO:0043933//macromolecular complex subunit organization;GO:0044281//small molecule metabolic process;GO:0071840//cellular component organization or biogenesis;GO:0034641//cellular nitrogen compound metabolic process;GO:0000278//mitotic cell cycle;GO:0044085//cellular component biogenesis;GO:0008150//biological_process;GO:0065003//macromolecular complex assembly;GO:0044763//single-organism cellular process;GO:0022607//cellular component assembly;GO:0044710//single-organism metabolic process;GO:0006807//nitrogen compound metabolic process;GO:0016043//cellular component organization;GO:0009058//biosynthetic process;GO:0044699//single-organism process;GO:0008152//metabolic process

Supplementary Table S3. GO and KEGG annotation clustering of regulated genes in A375R cells and A375 cellstreatment with vemurafenib.

Term	database	ID	GeneRatio	BgRatio	Pvalue	Qvalue	geneIDs	geneNames	URL
Signaling pathways regulating pluripotency of stem cells	KEGG PATHWAY	hsa04550	5/46	143/8105	0.00122114412673261	0.0824746363102392	7483/6926/6657/3976/1749	WNT9A/TBX3/SOX2/LIF/DLX5	http://www.genome.jp/kegg-bin/show_pathway?hsa04550/hsa:7483%09yellow/hsa:6926%09yellow/hsa:6657%09red/hsa:3976%09yellow/hsa:1749%09red
Rheumatoid arthritis	KEGG PATHWAY	hsa05323	4/46	93/8105	0.00183418997178269	0.0824746363102392	3569/1437/2321/3589	IL6/CSF2/FLT1/IL11	http://www.genome.jp/kegg-bin/show_pathway?hsa05323/hsa:3569%09yellow/hsa:1437%09yellow/hsa:2321%09red/hsa:3589%09yellow
JAK-STAT signaling pathway	KEGG PATHWAY	hsa04630	5/46	162/8105	0.00212106262047897	0.0824746363102392	3569/1437/11009/3589/3976	IL6/CSF2/IL24/IL11/LIF	http://www.genome.jp/kegg-bin/show_pathway?hsa04630/hsa:3569%09yellow/hsa:1437%09yellow/hsa:11009%09yellow/hsa:3589%09yellow/hsa:3976%09yellow

Term	database	ID	GeneRatio	BgRatio	Pvalue	Qvalue	geneIDs	geneNames	URL
Hematopoietic cell lineage	KEGG PATHWAY	hsa04640	4/46	99/8105	0.00230698283385284	0.0824746363102392	3569/1437/3589/948	IL6/CSF2/IL11/CD36	http://www.genome.jp/kegg-bin/show_pathway?hsa04640/hsa:3569%09yellow/hsa:1437%09yellow/hsa:3589%09yellow/hsa:948%09red
Hippo signaling pathway	KEGG PATHWAY	hsa04390	4/46	157/8105	0.0117606685384024	0.2911514798030004	7483/6657/5521/2246	WNT9A/SOX2/PPP2R2B/FGF1	http://www.genome.jp/kegg-bin/show_pathway?hsa04390/hsa:7483%09yellow/hsa:6657%09red/hsa:5521%09red/hsa:2246%09red
Complement and coagulation cascades	KEGG PATHWAY	hsa04610	3/46	85/8105	0.0122161460057204	0.2911514798030004	5055/3426/2	SERPINB2/CFI/A2M	http://www.genome.jp/kegg-bin/show_pathway?hsa04610/hsa:5055%09yellow/hsa:3426%09red/hsa:2%09red
IL-17 signaling pathway	KEGG PATHWAY	hsa04657	3/46	94/8105	0.0160034198019726	0.3022038562933349	3569/1437/3934	IL6/CSF2/LCN2	http://www.genome.jp/kegg-bin/show_pathway?hsa04657/hsa:3569%09yellow/hsa:1437%09yellow/hsa:3934%09yellow

Term	database	ID	GeneRatio	BgRatio	Pvalue	Qvalue	geneIDs	geneNames	URL
Protein digestion and absorption	KEGG PATHWAY	hsa04974	3/46	103/8105	0.02038249388782 49	0.3022038562933 49	482/1295/7373	ATP1B2/COL8A1/COL14A1	http://www.genome.jp/kegg-bin/show_pathway?hsa04974/hsa:482%09red/hsa:1295%09red/hsa:7373%09red
Transcriptional misregulation in cancer	KEGG PATHWAY	hsa05202	4/46	192/8105	0.0229566916643 08	0.3022038562933 49	3569/1437/2321/8013	IL6/CSF2/FLT1/NR4A3	http://www.genome.jp/kegg-bin/show_pathway?hsa05202/hsa:3569%09yellow/hsa:1437%09yellow/hsa:2321%09red/hsa:8013%09yellow
Kaposi sarcoma-associated herpesvirus infection	KEGG PATHWAY	hsa05167	4/46	194/8105	0.02374141124102 08	0.3022038562933 49	3569/1437/440738/4773	IL6/CSF2/MAP1LC3C/NFATC2	http://www.genome.jp/kegg-bin/show_pathway?hsa05167/hsa:3569%09yellow/hsa:1437%09yellow/hsa:440738%09red/hsa:4773%09red

Term	database	ID	GeneRatio	BgRatio	Pvalue	Qvalue	geneIDs	geneNames	URL
Cytokine-cytokine receptor interaction	KEGG PATHWAY	hsa04060	5/46	295/8105	0.0250061261750817	0.3022038562933349	3569/1437/11009/3589/3976	IL6/CSF2/IL24/IL11/LIF	http://www.genome.jp/kegg-bin/show_pathway?hsa04060/hsa:3569%09yellow/hsa:1437%09yellow/hsa:11009%09yellow/hsa:3589%09yellow/hsa:3976%09yellow
TNF signaling pathway	KEGG PATHWAY	hsa04668	3/46	112/8105	0.0253597641644768	0.3022038562933349	3569/1437/3976	IL6/CSF2/LIF	http://www.genome.jp/kegg-bin/show_pathway?hsa04668/hsa:3569%09yellow/hsa:1437%09yellow/hsa:3976%09yellow
Carbohydrate digestion and absorption	KEGG PATHWAY	hsa04973	2/46	47/8105	0.0289763722631366	0.3187400948945022	482/93432	ATP1B2/MGAM2	http://www.genome.jp/kegg-bin/show_pathway?hsa04973/hsa:482%09red/hsa:93432%09red
Malaria	KEGG PATHWAY	hsa05144	2/46	50/8105	0.0324870740660715	0.331832256532017	3569/948	IL6/CD36	http://www.genome.jp/kegg-bin/show_pathway?hsa05144/hsa:3569%09yellow/hsa:948%09red

Term	database	ID	GeneRatio	BgRatio	Pvalue	Qvalue	geneIDs	geneNames	URL
PI3K-Akt signaling pathway	KEGG PATHWAY	hsa04151	5/46	354/8105	0.04893102912061 42	0.466475810949855	3569/5521/2246/2321/4915	IL6/PPP2R2B/FGF1/FLT1/NTRK2	http://www.genome.jp/kegg-bin/show_pathway?hsa04151/hsa:3569%09yellow/hsa:5521%09red/hsa:2246%09red/hsa:2321%09red/hsa:4915%09red

GO_Biological Process

GOBPID	Term	GeneRatio	BgRatio	Pvalue	Qvalue	geneIDs	geneNames
GO:0032305	positive regulation of icosanoid secretion	5/197	16/18670	4.94109860075248e-07	0.00123850478614231	3553/22925/1906/4923/5608	IL1B/PLA2R1/EDN1/NTSR1/MAP2K6
GO:2000193	positive regulation of fatty acid transport	5/197	18/18670	9.52708708244424e-07	0.00123850478614231	3553/22925/1906/4923/5608	IL1B/PLA2R1/EDN1/NTSR1/MAP2K6
GO:0032303	regulation of icosanoid secretion	5/197	19/18670	1.28190770678228e-06	0.00123850478614231	3553/22925/1906/4923/5608	IL1B/PLA2R1/EDN1/NTSR1/MAP2K6
GO:0030198	extracellular matrix organization	16/197	368/18670	1.93866246908238e-06	0.00123850478614231	4318/23213/8728/9507/3691/284217/124056/7045/3037/3672/84171/3791/1290/1634/1520/176	MMP9/SULF1/ADAM19/ADAMTS4/ITGB4/LAMA1/NOXO1/TGFBI/HAS2/ITGA1/LOXL4/KDR/COL5A2/DCN/CTSS/ACAN

Term	database	ID	GeneRatio	BgRatio	Pvalue	Qvalue	geneIDs	geneNames	URL
GO:0043062	extracellular structure organization	16/197	369/18670	2.00795198790907e-06	0.00123850478614231	4318/23213/8728/9507/3691/284217/124056/7045/3037/3672/84171/3791/1290/1634/1520/176	MMP9/SULF1/ADAM19/ADAMTS4/ITGB4/LAMA1/NOXO1/TGFBI/HAS2/ITGA1/LOXL4/KDR/COL5A2/DCN/CTSS/ACAN		
GO:1905954	positive regulation of lipid localization	8/197	82/18670	2.43658024331369e-06	0.00125240224506323	3553/22925/718/1906/6863/5244/4923/5608	IL1B/PLA2R1/C3/EDN1/TAC1/ABCB4/NTSR1/MAP2K6		
GO:0032370	positive regulation of lipid transport	7/197	61/18670	3.52691968814037e-06	0.00141234760178788	3553/22925/1906/6863/5244/4923/5608	IL1B/PLA2R1/EDN1/TAC1/ABCB4/NTSR1/MAP2K6		
GO:0050804	modulation of chemical synaptic transmission	17/197	436/18670	3.99796410868663e-06	0.00141234760178788	3553/5327/6347/7476/793/254263/3814/9783/1906/25837/6863/784/4923/1009/81832/1759/4137	IL1B/PLAT/CCL2/WNT7A/CALB1/CNIH2/KISS1/RIMS3/EDN1/RAB26/TAC1/CACNB3/NTSR1/CDH11/NETO1/DNM1/MAPT		
GO:0099177	regulation of trans-synaptic signaling	17/197	437/18670	4.12163697019809e-06	0.00141234760178788	3553/5327/6347/7476/793/254263/3814/9783/1906/25837/6863/784/4923/1009/81832/1759/4137	IL1B/PLAT/CCL2/WNT7A/CALB1/CNIH2/KISS1/RIMS3/EDN1/RAB26/TAC1/CACNB3/NTSR1/CDH11/NETO1/DNM1/MAPT		
GO:0003014	renal system process	9/197	120/18670	5.14879258431375e-06	0.00158788763300236	23213/1364/3037/2702/1906/6863/185/4987/8644	SULF1/CLDN4/HAS2/GJA5/EDN1/TAC1/AGTR1/OPRL1/AKR1C3		

Term	database	ID	GeneRatio	BgRatio	Pvalue	Qvalue	geneIDs	geneNames	URL
GO:2000191	regulation of fatty acid transport	5/197	27/18670	8.30966205666955e-06	0.00232972707116081	3553/22925/1906/4923/5608	IL1B/PLA2R1/EDN1/NTSR1/MAP2K6		
GO:1903793	positive regulation of anion transport	6/197	50/18670	1.38103394913755e-05	0.0035492572492835	3553/22925/1906/5244/4923/5608	IL1B/PLA2R1/EDN1/ABCB4/NTSR1/MAP2K6		
GO:0031644	regulation of neurological system process	9/197	137/18670	1.51237243829024e-05	0.00358781276899008	7476/254263/55/1906/784/4923/4987/81832/27242	WNT7A/CNIH2/ACPP/EDN1/CACNB3/NTSR1/OPRL1/NETO1/TNFRSF21		
GO:0032892	positive regulation of organic acid transport	5/197	34/18670	2.69747044087609e-05	0.00594214202832989	3553/22925/1906/4923/5608	IL1B/PLA2R1/EDN1/NTSR1/MAP2K6		
GO:0050729	positive regulation of inflammatory response	9/197	153/18670	3.63912561529296e-05	0.00716806179942944	3553/9173/26471/718/6863/5138/7941/185/59341	IL1B/IL1RL1/NUPR1/C3/TAC1/PDE2A/PLA2G7/AGTR1/TRPV4		
GO:0043270	positive regulation of ion transport	12/197	275/18670	3.71883880644848e-05	0.00716806179942944	3553/6347/1272/22925/1906/2273/1520/5244/784/8787/4923/5608	IL1B/CCL2/CNTN1/PLA2R1/EDN1/FHL1/CTSS/ABCB4/CACNB3/RGS9/NTSR1/MAP2K6		
GO:1905952	regulation of lipid localization	9/197	156/18670	4.23803051595297e-05	0.00720171394615873	3553/22925/718/1906/6863/5244/4923/5608/185	IL1B/PLA2R1/C3/EDN1/TAC1/ABCB4/NTSR1/MAP2K6/AGTR1		
GO:0071621	granulocyte chemotaxis	8/197	123/18670	4.87221625103616e-05	0.00720171394615873	3553/6347/3672/27178/1906/3576/2919/59341	IL1B/CCL2/ITGA1/IL37/EDN1/CXCL8/CXCL1/TRPV4		

Term	database	ID	GeneRatio	BgRatio	Pvalue	Qvalue	geneIDs	geneNames	URL
GO:0051899	membrane depolarization	7/197	91/18670	5.01252257228366e-05	0.00720171394615873	2702/3791/1906/2273/1634/784/4923	GJA5/KDR/EDN1/FHL1/DCN/CACNB3/NTSR1		
GO:0051767	nitric-oxide synthase biosynthetic process	4/197	20/18670	5.103063518234e-05	0.00720171394615873	6347/3791/1906/5608	CCL2/KDR/EDN1/MAP2K6		
GO:0051769	regulation of nitric-oxide synthase biosynthetic process	4/197	20/18670	5.103063518234e-05	0.00720171394615873	6347/3791/1906/5608	CCL2/KDR/EDN1/MAP2K6		
GO:0046717	acid secretion	8/197	124/18670	5.16176026539838e-05	0.00720171394615873	3553/22925/1906/5244/4923/5608/7032/4987	IL1B/PLA2R1/EDN1/ABCB4/NTSR1/MAP2K6/TFF2/OPRL1		
GO:0032368	regulation of lipid transport	8/197	125/18670	5.46550018051094e-05	0.00720171394615873	3553/22925/1906/6863/5244/4923/5608/185	IL1B/PLA2R1/EDN1/TAC1/ABCB4/NTSR1/MAP2K6/AGTR1		
GO:0042391	regulation of membrane potential	15/197	434/18670	6.02284583538426e-05	0.00720171394615873	3756/7476/254263/2702/3791/3728/9783/1906/2273/6863/1634/784/4923/81832/4137	KCNH1/WNT7A/CNIH2/GJA5/KDR/JUP/RIMS3/EDN1/FHL1/TAC1/DCN/CACNB3/NTSR1/NETO1/MAPT		
GO:0051931	regulation of sensory perception	5/197	40/18670	6.05979595144092e-05	0.00720171394615873	55/1906/784/4923/4987	ACPP/EDN1/CACNB3/NTSR1/OPRL1		
GO:0048871	multicellular organismal homeostasis	16/197	486/18670	6.07148387159945e-05	0.00720171394615873	3553/3552/1364/9079/3037/2810/12/57333/8013/4923/5608/7032/59341/7351/11151/50486	IL1B/IL1A/CLDN4/LDB2/HAS2/SFN/SERPINA3/RCN3/NR4A3/NTSR1/MAP2K6/TFF2/TRPV4/UCP2/CORO1A/G0S2		

Term	database	ID	GeneRatio	BgRatio	Pvalue	Qvalue	geneIDs	geneNames	URL
GO:0140353	lipid export from cell	5/197	41/18670	6.84270770758871e-05	0.0078158928037791	3553/1906/6863/5608/185	IL1B/EDN1/TAC1/MAP2K6/AGTR1		
GO:0050806	positive regulation of synaptic transmission	9/197	168/18670	7.53673630856888e-05	0.00830117670558086	6347/7476/793/3814/9783/6863/784/4923/81832	CCL2/WNT7A/CALB1/KISS1/RIMS3/TAC1/CACNB3/NTSR1/NETO1		
GO:0009612	response to mechanical stimulus	10/197	210/18670	8.11432681830285e-05	0.00862916686470551	3553/64220/3728/1906/1634/5138/784/958/7100/59341	IL1B/STRA6/JUP/EDN1/DCN/PDE2A/CACNB3/CD40/TLR5/TRPV4		
GO:0044060	regulation of endocrine process	5/197	44/18670	9.66538844020377e-05	0.00967411444284929	3553/2702/3814/6863/185	IL1B/GJA5/KISS1/TAC1/AGTR1		
GO:0060326	cell chemotaxis	12/197	304/18670	9.72430440104825e-05	0.00967411444284929	3553/6347/3672/3791/27178/1906/7941/3576/2919/185/59341/11151	IL1B/CCL2/ITGA1/KDR/IL37/EDN1/PLA2G7/CXCL8/CXCL1/AGTR1/TRPV4/CORO1A		
GO:0032412	regulation of ion transmembrane transporter activity	11/197	260/18670	0.000103509079816765	0.00997568756734068	4318/6347/254263/2273/1520/784/8787/4923/5349/4987/81832	MMP9/CCL2/CNIH2/FHL1/CTSS/CACNB3/RGS9/NTSR1/FXYD3/OPRL1/NETO1		
GO:0032309	icosanoid secretion	5/197	45/18670	0.000107811170504304	0.0100754439344023	3553/22925/1906/4923/5608	IL1B/PLA2R1/EDN1/NTSR1/MAP2K6		
GO:0019233	sensory perception of pain	7/197	104/18670	0.000117242907200664	0.0100889154227684	6347/55/1906/6863/784/4923/4987	CCL2/ACPP/EDN1/TAC1/CACNB3/NTSR1/OPRL1		
GO:0030593	neutrophil chemotaxis	7/197	104/18670	0.000117242907200664	0.0100889154227684	3553/6347/3672/27178/1906/3576/2919	IL1B/CCL2/ITGA1/IL37/EDN1/CXCL8/CXCL1		

Term	database	ID	GeneRatio	BgRatio	Pvalue	Qvalue	geneIDs	geneNames	URL
GO:0034329	cell junction assembly	14/197	409/18670	0.000117769440732705	0.0100889154227684	2045/266977/3691/7476/2702/92737/2596/94/3791/3728/64403/1009/59341/153562	EPHA7/ADGRF1/ITGB4/WNT7A/GJA5/DNER/GAP43/ACVRL1/KDR/JUP/CDH24/CDH11/TRPV4/MARVELD2		
GO:0097530	granulocyte migration	8/197	141/18670	0.000127453710356788	0.0101965097002243	3553/6347/3672/27178/1906/3576/2919/59341	IL1B/CCL2/ITGA1/IL37/EDN1/CXCL8/CXCL1/TRPV4		
GO:2001257	regulation of cation channel activity	9/197	180/18670	0.000127715647799997	0.0101965097002243	4318/6347/254263/1520/784/8787/4923/4987/81832	MMP9/CCL2/CNIH2/CTSS/CACNB3/RGS9/NTSR1/OPRL1/NETO1		
GO:0032308	positive regulation of prostaglandin secretion	3/197	10/18670	0.000131468276424966	0.0101965097002243	3553/1906/5608	IL1B/EDN1/MAP2K6		
GO:0022898	regulation of transmembrane transporter activity	11/197	268/18670	0.000135178202680213	0.0101965097002243	4318/6347/254263/2273/1520/784/8787/4923/5349/4987/81832	MMP9/CCL2/CNIH2/FHL1/CTSS/CACNB3/RGS9/NTSR1/FXYD3/OPRL1/NETO1		
GO:0030595	leukocyte chemotaxis	10/197	224/18670	0.000138151702529861	0.0101965097002243	3553/6347/3672/27178/1906/7941/3576/2919/59341/11151	IL1B/CCL2/ITGA1/IL37/EDN1/PLA2G7/CXCL8/CXCL1/TRPV4/CORO1A		
GO:0008217	regulation of blood pressure	9/197	182/18670	0.000138862972571148	0.0101965097002243	2702/5742/94/1906/347148/6863/4923/185/4987	GJA5/PTGS1/ACVRL1/EDN1/QRFP/TAC1/NTSR1/AGTR1/OPRL1		
GO:0003254	regulation of membrane depolarization	5/197	49/18670	0.000162621699604699	0.0111450071462421	2702/3791/2273/1634/4923	GJA5/KDR/FHL1/DCN/NTSR1		

Term	database	ID	GeneRatio	BgRatio	Pvalue	Qvalue	geneIDs	geneNames	URL
GO:0071715	icosanoid transport	5/197	49/18670	0.000162621699604699	0.0111450071462421	3553/22925/1906/4923/5608	IL1B/PLA2R1/EDN1/NTSR1/MAP2K6		
GO:1901571	fatty acid derivative transport	5/197	49/18670	0.000162621699604699	0.0111450071462421	3553/22925/1906/4923/5608	IL1B/PLA2R1/EDN1/NTSR1/MAP2K6		
GO:1904018	positive regulation of vasculature development	10/197	230/18670	0.000171341596533632	0.0114873366023853	3553/3552/718/94/3791/8710/3728/958/3576/185	IL1B/IL1A/C3/ACVRL1/KDR/SERPINB7/JUP/CD40/CXCL8/AGTR1		
GO:0032306	regulation of prostaglandin secretion	3/197	11/18670	0.000179364650366192	0.0117693740793476	3553/1906/5608	IL1B/EDN1/MAP2K6		
GO:0001516	prostaglandin biosynthetic process	4/197	28/18670	0.000201872698835099	0.0127056204736213	3553/5742/1906/8644	IL1B/PTGS1/EDN1/AKR1C3		
GO:0046457	prostanoid biosynthetic process	4/197	28/18670	0.000201872698835099	0.0127056204736213	3553/5742/1906/8644	IL1B/PTGS1/EDN1/AKR1C3		
GO:0032496	response to lipopolysaccharide	12/197	330/18670	0.000208890069868239	0.012884339509473	3553/1437/6347/342979/27178/1906/6863/1634/249/3576/2919/11009	IL1B/CSF2/CCL2/PALM3/IL37/EDN1/TAC1/DCN/ALPL/CXCL8/CXCL1/IL24		
GO:0032409	regulation of transporter activity	11/197	283/18670	0.000217060699180131	0.0131257881621868	4318/6347/254263/2273/1520/784/8787/4923/5349/4987/81832	MMP9/CCL2/CNIH2/FHL1/CTSS/CACNB3/RGS9/NTSR1/FXYD3/OPRL1/NETO1		
GO:0010575	positive regulation of vascular endothelial growth factor production	4/197	29/18670	0.00023224978186696	0.0137741986014943	3553/23213/3552/718	IL1B/SULF1/IL1A/C3		

Term	database	ID	GeneRatio	BgRatio	Pvalue	Qvalue	geneIDs	geneNames	URL
GO:0070486	leukocyte aggregation	3/197	12/18670	0.000237296014992568	0.0138079417025864	3553/3037/8013	IL1B/HAS2/NR4A3		
GO:1990266	neutrophil migration	7/197	118/18670	0.000257080733959604	0.0146821663616929	3553/6347/3672/27178/1906/3576/2919	IL1B/CCL2/ITGA1/IL37/EDN1/CXCL8/CXCL1		
GO:0050886	endocrine process	6/197	84/18670	0.000262531653933443	0.014720865831468	3553/2702/3814/1906/6863/185	IL1B/GJA5/KISS1/EDN1/TAC1/AGTR1		
GO:0042119	neutrophil activation	15/197	498/18670	0.000271641812918098	0.0149597026971324	4318/10326/55/5337/10788/718/12/3728/22918/1520/3576/2919/79930/230/2212	MMP9/SIRPB1/ACPP/PLD1/IQGAP2/C3/SERPINA3/JUP/CD93/CTSS/CXCL8/CXCL1/DOK3/ALDOC/FCGR2A		
GO:0098900	regulation of action potential	5/197	55/18670	0.000281882387369502	0.0152513207482025	2702/3728/6863/784/4923	GJA5/JUP/TAC1/CACNB3/NTSR1		
GO:1904062	regulation of cation transmembrane transport	12/197	342/18670	0.000289445183789181	0.0153904990828592	4318/6347/254263/2273/1520/784/8787/4923/5349/4987/81832/11151	MMP9/CCL2/CNIH2/FHL1/CTSS/CACNB3/RGS9/NTSR1/FXYD3/OPRL1/NETO1/CORO1A		
GO:0002237	response to molecule of bacterial origin	12/197	343/18670	0.000297211333794163	0.0155355890410373	3553/1437/6347/342979/27178/1906/6863/1634/249/3576/2919/11009	IL1B/CSF2/CCL2/PALM3/IL37/EDN1/TAC1/DCN/ALPL/CXCL8/CXCL1/IL24		

Term	database	ID	GeneRatio	BgRatio	Pvalue	Qvalue	geneIDs	geneNames	URL
GO:0051482	positive regulation of cytosolic calcium ion concentration involved in phospholipase C-activating G protein-coupled signaling pathway	4/197	31/18670	0.000302652386549505	0.0155563326686446	3814/1906/185/4987	KISS1/EDN1/AGTR1/OPRL1		
GO:0045766	positive regulation of angiogenesis	9/197	204/18670	0.000325144337791286	0.0164384448811201	3553/3552/718/94/3791/3728/958/3576/185	IL1B/IL1A/C3/ACVRL1/KDR/JUP/CD40/CXCL8/AGTR1		
GO:0010951	negative regulation of endopeptidase activity	10/197	250/18670	0.000334388281331135	0.0166331203165358	4318/5104/7078/718/2810/8710/12/27074/5055/25816	MMP9/SERPINA5/TIMP3/C3/SFN/SERPINB7/SERPINA3/LAMP3/SERPINB2/TNFAIP8		
GO:0015718	monocarboxylic acid transport	8/197	163/18670	0.000343350042149367	0.0168078020633119	3553/64220/22925/1906/6540/5244/4923/5608	IL1B/STRA6/PLA2R1/EDN1/SLC6A13/ABCB4/NTSR1/MAP2K6		
GO:0032310	prostaglandin secretion	3/197	14/18670	0.000386548385464759	0.0183209416634894	3553/1906/5608	IL1B/EDN1/MAP2K6		
GO:0035810	positive regulation of urine volume	3/197	14/18670	0.000386548385464759	0.0183209416634894	3037/1906/4987	HAS2/EDN1/OPRL1		
GO:0032890	regulation of organic acid transport	5/197	59/18670	0.000392082409140824	0.0183209416634894	3553/22925/1906/4923/5608	IL1B/PLA2R1/EDN1/NTSR1/MAP2K6		

Term	database	ID	GeneRatio	BgRatio	Pvalue	Qvalue	geneIDs	geneNames	URL
GO:0097529	myeloid leukocyte migration	9/197	210/18670	0.000401955048505925	0.0185019308894369	3553/6347/3672/27178/1906/7941/3576/2919/59341	IL1B/CCL2/ITGA1/IL37/EDN1/PLA2G7/CXCL8/CXCL1/TRPV4		
GO:0010574	regulation of vascular endothelial growth factor production	4/197	34/18670	0.000435193918499187	0.0197373241860514	3553/23213/3552/718	IL1B/SULF1/IL1A/C3		
GO:0051770	positive regulation of nitric-oxide synthase biosynthetic process	3/197	15/18670	0.00047943942778909	0.0208736198190113	6347/3791/5608	CCL2/KDR/MAP2K6		
GO:0010876	lipid localization	13/197	415/18670	0.000482136723811617	0.0208736198190113	3553/5104/64220/22925/718/1906/148229/6863/134429/5244/4923/5608/185	IL1B/SERPINA5/STRA6/PLA2R1/C3/EDN1/ATP8B3/TAC1/STARD4/ABCB4/NTSR1/MAP2K6/AGTR1		
GO:0010466	negative regulation of peptidase activity	10/197	262/18670	0.000483450837667978	0.0208736198190113	4318/5104/7078/718/2810/8710/12/27074/5055/25816	MMP9/SERPINA5/TIMP3/C3/SFN/SERPINB7/SERPINA3/LAMP3/SERPINB2/TNFAIP8		
GO:0098801	regulation of renal system process	4/197	35/18670	0.000487321863478864	0.0208736198190113	2702/1906/6863/185	GJA5/EDN1/TAC1/AGTR1		
GO:0003158	endothelium development	7/197	132/18670	0.000507825552957681	0.0212589835395825	3553/7476/2702/94/3791/5138/153562	IL1B/WNT7A/GJA5/ACVRL1/KDR/PDE2A/MARVELD2		

Term	database	ID	GeneRatio	BgRatio	Pvalue	Qvalue	geneIDs	geneNames	URL
GO:0044070	regulation of anion transport	6/197	95/18670	0.000510105311909567	0.0212589835395825	3553/22925/1906/5244/4923/5608	IL1B/PLA2R1/EDN1/ABCB4/NTSR1/MAP2K6		
GO:0031646	positive regulation of neurological system process	5/197	63/18670	0.000532059843533256	0.0218783007660875	7476/784/4923/4987/81832	WNT7A/CACNB3/NTSR1/OPRL1/NETO1		
GO:0010573	vascular endothelial growth factor production	4/197	36/18670	0.000543746307814175	0.0220646528065647	3553/23213/3552/718	IL1B/SULF1/IL1A/C3		
GO:1901342	regulation of vasculature development	13/197	422/18670	0.000563847990587986	0.0225832104282253	3553/23213/3552/718/94/3791/8710/3728/1634/958/3576/185/3087	IL1B/SULF1/IL1A/C3/ACVRL1/KDR/SERPINB7/JUP/DCN/CD40/CXCL8/AGTR1/HHEX		
GO:0070293	renal absorption	3/197	16/18670	0.000585506776139676	0.0231500371489072	1364/3037/8644	CLDN4/HAS2/AKR1C3		
GO:0034765	regulation of ion transmembrane transport	14/197	483/18670	0.000634251052056213	0.0247598765131818	4318/3756/6347/254263/2702/2273/1520/784/8787/4923/5349/4987/81832/11151	MMP9/KCNH1/CCL2/CNIH2/GJA5/FHL1/CTSS/CACNB3/RGS9/NTSR1/FXYD3/OPRL1/NETO1/CORO1A		
GO:0043312	neutrophil degranulation	14/197	485/18670	0.000660332981162934	0.0254558364238311	4318/10326/55/5337/10788/718/12/3728/22918/1520/2919/79930/230/2212	MMP9/SIRPB1/ACPP/PLD1/IQGAP2/C3/SERPINA3/JUP/CD93/CTSS/CXCL1/DOK3/ALDOC/FCGR2A		

Term	database	ID	GeneRatio	BgRatio	Pvalue	Qvalue	geneIDs	geneNames	URL
GO:0002283	neutrophil activation involved in immune response	14/197	488/18670	0.000701160605734291	0.0265324014505818	4318/10326/55/5337/10788/718/12/3728/22918/1520/2919/79930/230/2212	MMP9/SIRPB1/ACPP/PLD1/IQGAP2/C3/SERPINA3/JUP/CD93/CTSS/CXCL1/DOK3/ALDOC/FCGR2A		
GO:0015732	prostaglandin transport	3/197	17/18670	0.000705465927025846	0.0265324014505818	3553/1906/5608	IL1B/EDN1/MAP2K6		
GO:0050673	epithelial cell proliferation	13/197	434/18670	0.000731210961635626	0.0268764293524693	23213/3488/6347/7476/3037/26471/2810/94/3791/26298/8013/185/2173	SULF1/IGFBP5/CCL2/WNT7A/HAS2/NUPR1/SFN/ACVRL1/KDR/EHF/NR4A3/AGTR1/FABP7		
GO:0006869	lipid transport	12/197	380/18670	0.000738891200878015	0.0268764293524693	3553/5104/64220/22925/1906/148229/6863/134429/5244/4923/5608/185	IL1B/SERPINA5/STRA6/PLA2R1/EDN1/ATP8B3/TAC1/STARD4/ABCB4/NTSR1/MAP2K6/AGTR1		
GO:0051930	regulation of sensory perception of pain	4/197	39/18670	0.000740757618339782	0.0268764293524693	55/1906/4923/4987	ACPP/EDN1/NTSR1/OPRL1		
GO:0008277	regulation of G protein-coupled receptor signaling pathway	7/197	142/18670	0.000784241521415073	0.0280464652232444	55/718/1906/8787/3576/4987/1759	ACPP/C3/EDN1/RGS9/CXCL8/OPRL1/DNM1		
GO:0045765	regulation of angiogenesis	12/197	383/18670	0.000791194057854161	0.0280464652232444	3553/23213/3552/718/94/3791/3728/1634/958/3576/185/3087	IL1B/SULF1/IL1A/C3/ACVRL1/KDR/JUP/DCN/CD40/CXCL8/AGTR1/HHEX		

Term	database	ID	GeneRatio	BgRatio	Pvalue	Qvalue	geneIDs	geneNames	URL
GO:0002446	neutrophil mediated immunity	14/197	499/18670	0.000869682861701168	0.0304784311987091	4318/10326/55/5337/10788/718/12/3728/22918/1520/2919/79930/230/2212	MMP9/SIRPB1/ACPP/PLD1/IQGAP2/C3/SERPINA3/JUP/CD93/CTSS/CXCL1/DOK3/ALDOC/FCGR2A		
GO:0061028	establishment of endothelial barrier	4/197	41/18670	0.000897185382596059	0.0310889856171488	3553/7476/5138/153562	IL1B/WNT7A/PDE2A/MARVELD2		
GO:0050795	regulation of behavior	5/197	71/18670	0.000920815542025288	0.0315532792400665	1437/64220/347148/8013/4987	CSF2/STRA6/QRFP/NR4A3/OPRL1		
GO:2001259	positive regulation of cation channel activity	5/197	72/18670	0.000981172365495356	0.0331794340713428	6347/1520/784/8787/4923	CCL2/CTSS/CACNB3/RGS9/NTSR1		
GO:0002922	positive regulation of humoral immune response	3/197	19/18670	0.000989788565033571	0.0331794340713428	3553/718/57829	IL1B/C3/ZP4		
GO:0002526	acute inflammatory response	6/197	108/18670	0.00100327305479949	0.0332698290430283	3553/3552/26471/718/12/6863	IL1B/IL1A/NUPR1/C3/SERPINA3/TAC1		
GO:0052547	regulation of peptidase activity	13/197	452/18670	0.00105961645262527	0.0343833111694349	4318/2045/5104/7078/718/2810/8710/12/57333/27074/5055/25816/4137	MMP9/EPHA7/SERPINA5/TIMP3/C3/SFN/SERPINB7/SERPINA3/RCN3/LAMP3/SERPINB2/TNFAIP8/MAPT		
GO:0006692	prostanoid metabolic process	4/197	43/18670	0.00107553099669981	0.0343833111694349	3553/5742/1906/8644	IL1B/PTGS1/EDN1/AKR1C3		
GO:0006693	prostaglandin metabolic process	4/197	43/18670	0.00107553099669981	0.0343833111694349	3553/5742/1906/8644	IL1B/PTGS1/EDN1/AKR1C3		

Term	database	ID	GeneRatio	BgRatio	Pvalue	Qvalue	geneIDs	geneNames	URL
GO:0010632	regulation of epithelial cell migration	10/197	291/18670	0.00108144655753411	0.0343833111694349	4318/7476/3037/94/3791/3728/1906/6863/1634/958	MMP9/WNT7A/HAS2/ACVRL1/KDR/JUP/EDN1/TAC1/DCN/CD40		
GO:0006024	glycosaminoglycan biosynthetic process	6/197	110/18670	0.0011036671868958	0.0347317306570067	3553/3037/1634/9957/176/4166	IL1B/HAS2/DCN/HS3ST1/ACAN/CHST6		
GO:0072001	renal system development	10/197	293/18670	0.00113860991602707	0.0354694240507827	4318/23213/2045/3691/793/64220/3037/8710/1634/185	MMP9/SULF1/EPHA7/ITGB4/CALB1/STRA6/HAS2/SERPINB7/DCN/AGTR1		
GO:0022407	regulation of cell-cell adhesion	12/197	402/18670	0.00119949410433653	0.0369923981777385	3553/2045/8728/6347/10326/3037/57829/8013/10148/59341/27242/11151	IL1B/EPHA7/ADAM19/CCL2/SIRPB1/HAS2/ZP4/NR4A3/EBI3/TRPV4/TNFRSF21/CORO1A		
GO:0045785	positive regulation of cell adhesion	12/197	403/18670	0.00122509348960696	0.0374078051678007	3553/8728/6347/10326/3037/3791/57829/3728/8013/4739/10148/11151	IL1B/ADAM19/CCL2/SIRPB1/HAS2/KDR/ZP4/JUP/NR4A3/NEDD9/EBI3/CORO1A		
GO:0051346	negative regulation of hydrolase activity	13/197	463/18670	0.00131536575328003	0.0393861926426116	4318/5104/7078/10156/10788/22925/718/2810/8710/12/27074/5055/25816	MMP9/SERPINA5/TIMP3/RASA4/IQGAP2/PLA2R1/C3/SFN/SERPINB7/SERPINA3/LAMP3/SERPINB2/TNFAIP8		
GO:0045446	endothelial cell differentiation	6/197	114/18670	0.00132742073564827	0.0393861926426116	3553/7476/94/3791/5138/153562	IL1B/WNT7A/ACVRL1/KDR/PDE2A/MARVELD2		

Term	database	ID	GeneRatio	BgRatio	Pvalue	Qvalue	geneIDs	geneNames	URL
GO:0035813	regulation of renal sodium excretion	3/197	21/18670	0.00133760718676397	0.0393861926426116	1906/6863/185	EDN1/TAC1/AGTR1		
GO:0001819	positive regulation of cytokine production	13/197	464/18670	0.00134096959386324	0.0393861926426116	3553/23213/3552/1437/9173/718/27178/8710/8013/958/10148/7100/59341	IL1B/SULF1/IL1A/CSF2/IL1RL1/C3/IL37/SERPINB7/NR4A3/CD40/EBI3/TLR5/TRPV4		
GO:0006023	aminoglycan biosynthetic process	6/197	115/18670	0.0013884136100054	0.0403949771076088	3553/3037/1634/9957/176/4166	IL1B/HAS2/DCN/HS3ST1/ACAN/CHST6		
GO:0060986	endocrine hormone secretion	4/197	47/18670	0.00150431567802149	0.0430874760214154	3553/3814/6863/185	IL1B/KISS1/TAC1/AGTR1		
GO:0045861	negative regulation of proteolysis	11/197	358/18670	0.00151944984020674	0.0430874760214154	4318/5104/5327/7078/718/2810/8710/12/27074/5055/25816	MMP9/SERPINA5/PLAT/TIMP3/C3/SFN/SERPINB7/SERPINA3/LAMP3/SERPINB2/TNFAIP8		
GO:0035809	regulation of urine volume	3/197	22/18670	0.00153684252994672	0.0430874760214154	3037/1906/4987	HAS2/EDN1/OPRL1		
GO:0044342	type B pancreatic cell proliferation	3/197	22/18670	0.00153684252994672	0.0430874760214154	3488/26471/8013	IGFBP5/NUPR1/NR4A3		
GO:0022409	positive regulation of cell-cell adhesion	9/197	255/18670	0.00158859914054437	0.0441372950399894	3553/8728/6347/10326/3037/57829/8013/10148/11151	IL1B/ADAM19/CCL2/SIRPB1/HAS2/ZP4/NR4A3/EBI3/CORO1A		
GO:0008038	neuron recognition	4/197	48/18670	0.00162768267912791	0.0445349077826926	2596/84665/57549/27242	GAP43/MYPN/IGSF9/TNFRSF21		

Term	database	ID	GeneRatio	BgRatio	Pvalue	Qvalue	geneIDs	geneNames	URL
GO:0007611	learning or memory	9/197	256/18670	0.00163179136817259	0.0445349077826926	266977/793/64220/5803/6863/4923/4987/81832/4137	ADGRF1/CALB1/STRA6/PTPRZ1/TAC1/NTSR1/OPRL1/NETO1/MAPT		
GO:0051216	cartilage development	8/197	209/18670	0.001731177917000025	0.0463367130964424	23213/7476/7045/94/1906/176/6660/59341	SULF1/WNT7A/TGFBI/ACVRL1/EDN1/ACAN/SOX5/TRPV4		
GO:0035812	renal sodium excretion	3/197	23/18670	0.00175372144057541	0.0463367130964424	1906/6863/185	EDN1/TAC1/AGTR1		
GO:0090140	regulation of mitochondrial fission	3/197	23/18670	0.00175372144057541	0.0463367130964424	3791/1634/4137	KDR/DCN/MAPT		
GO:1904707	positive regulation of vascular smooth muscle cell proliferation	4/197	49/18670	0.00175791032175219	0.0463367130964424	4318/3488/1906/8013	MMP9/IGFBP5/EDN1/NR4A3		
GO:0071277	cellular response to calcium ion	5/197	83/18670	0.0018548798077943	0.0484783841291324	3756/10156/1906/100271927/8644	KCNH1/RASA4/EDN1/RASA4B/AKR1C3		
GO:0052548	regulation of endopeptidase activity	12/197	425/18670	0.0019138876837985	0.0496002488809628	4318/2045/5104/7078/718/2810/8710/12/27074/5055/25816/4137	MMP9/EPHA7/SERPINA5/TIMP3/C3/SFN/SERPINB7/SERPINA3/LAMP3/SERPINB2/TNFAIP8/MAPT		

GO-Molecular_Function

GOMFID	Term	GeneRatio	BgRatio	Pvalue	Qvalue	geneIDs	geneNames

Term	database	ID	GeneRatio	BgRatio	Pvalue	Qvalue	geneIDs	geneNames	URL
GO:0001664	G protein-coupled receptor binding	14/195	280/17696	2.82868804509395e-06	0.00119653504307474	3691/6347/7476/718/3814/1906/347148/5064/6863/3576/2919/185/7032/1759	ITGB4/CCL2/WNT7A/C3/KISS1/EDN1/QRFP/PALM/TAC1/CXCL8/CXCL1/AGTR1/TFF2/DNM1		
GO:0005149	interleukin-1 receptor binding	4/195	16/17696	2.34577755912953e-05	0.00475282242482181	3553/3552/27178/7100	IL1B/IL1A/IL37/TLR5		
GO:0005125	cytokine activity	11/195	220/17696	3.37079604597292e-05	0.00475282242482181	3553/3552/1437/6347/7476/27178/1906/10148/3576/2919/11009	IL1B/IL1A/CSF2/CCL2/WNT7A/IL37/EDN1/EBI3/CXCL8/CXCL1/IL24		
GO:0005543	phospholipid binding	14/195	427/17696	0.000284888059005557	0.0189775629808999	5104/3756/26499/124056/10156/5337/10788/2596/55803/7941/100271927/129880/5338/4137	SERPINA5/KCNH1/PLEK2/NOXO1/RASA4/PLD1/IQGAP2/GAP43/ADAP2/PLA2G7/RASA4B/BBS5/PLD2/MAPT		
GO:0048018	receptor ligand activity	15/195	482/17696	0.000302343894505774	0.0189775629808999	3553/2045/3552/1437/6347/7476/27178/1906/347148/10148/2250/3576/2919/4137/11009	IL1B/EPHA7/IL1A/CSF2/CCL2/WNT7A/IL37/EDN1/QRFP/EBI3/FGF5/CXCL8/CXCL1/MAPT/IL24		
GO:0030546	receptor activator activity	15/195	487/17696	0.000337192572606752	0.0189775629808999	3553/2045/3552/1437/6347/7476/27178/1906/347148/10148/2250/3576/2919/4137/11009	IL1B/EPHA7/IL1A/CSF2/CCL2/WNT7A/IL37/EDN1/QRFP/EBI3/FGF5/CXCL8/CXCL1/MAPT/IL24		

Term	database	ID	GeneRatio	BgRatio	Pvalue	Qvalue	geneIDs	geneNames	URL
GO:0005126	cytokine receptor binding	11/195	286/17696	0.000342095042779788	0.0189775629808999	3553/3552/1437/6347/27178/10148/3576/7185/2919/7100/7032	IL1B/IL1A/CSF2/CCL2/IL37/EBI3/CXCL8/TRAF1/CXCL1/TLR5/TFF2		
GO:0035091	phosphatidylinositol binding	10/195	245/17696	0.000399207951709612	0.0189775629808999	3756/26499/124056/5337/10788/2596/55803/129880/5338/4137	KCNH1/PLEK2/NOXO1/PLD1/IQGAP2/GAP43/ADAP2/BBS5/PLD2/MAPT		
GO:0071855	neuropeptide receptor binding	4/195	32/17696	0.000403777935763828	0.0189775629808999	3814/1906/347148/6863	KISS1/EDN1/QRFP/TAC1		
GO:0098631	cell adhesion mediator activity	5/195	59/17696	0.000476994610792461	0.0201768720365211	3672/3728/84665/57549/342035	ITGA1/JUP/MYPN/IGSF9/GLDN		
GO:0016641	oxidoreductase activity, acting on the CH-NH2 group of donors, oxygen as acceptor	3/195	16/17696	0.0006637924179793	0.0255258357095676	84171/314/8639	LOXL4/AOC2/AOC3		
GO:0005518	collagen binding	5/195	67/17696	0.000857099446373286	0.0302127554846583	4318/7045/3672/1634/1520	MMP9/TGFBI/ITGA1/DCN/CTSS		
GO:0016638	oxidoreductase activity, acting on the CH-NH2 group of donors	3/195	20/17696	0.00130815139116256	0.042565233727828	84171/314/8639	LOXL4/AOC2/AOC3		
GO:1901981	phosphatidylinositol phosphate binding	7/195	161/17696	0.00207418164891124	0.0624996297638117	3756/26499/10788/2596/55803/129880/4137	KCNH1/PLEK2/IQGAP2/GAP43/ADAP2/BBS5/MAPT		
GO:0098632	cell-cell adhesion mediator activity	4/195	50/17696	0.00221629892779474	0.0624996297638117	3728/84665/57549/342035	JUP/MYPN/IGSF9/GLDN		

Term	database	ID	GeneRatio	BgRatio	Pvalue	Qvalue	geneIDs	geneNames	URL
GO:0017124	SH3 domain binding	6/195	130/17696	0.00319545102656603	0.0788345264921425	8728/9047/2530/84665/1759/4137	ADAM19/SH2D2A/FUT8/MYPN/DNM1/MAPT		
GO:0004866	endopeptidase inhibitor activity	7/195	175/17696	0.00330601701422855	0.0788345264921425	5104/7078/718/8710/12/5055/25816	SERPINA5/TIMP3/C3/SERPINB7/SERPINA3/SERPINB2/TNFAIP8		
GO:0050840	extracellular matrix binding	4/195	57/17696	0.00358101944254348	0.0788345264921425	7045/27076/1634/1520	TGFBI/LYPD3/DCN/CTSS		
GO:0070851	growth factor receptor binding	6/195	134/17696	0.00371019691925919	0.0788345264921425	3553/3552/1437/27178/2250/7100	IL1B/IL1A/CSF2/IL37/FGF5/TLR5		
GO:0005507	copper ion binding	4/195	59/17696	0.00405631903893057	0.0788345264921425	3552/84171/314/8639	IL1A/LOXL4/AOC2/AOC3		
GO:0030414	peptidase inhibitor activity	7/195	182/17696	0.00410014085774737	0.0788345264921425	5104/7078/718/8710/12/5055/25816	SERPINA5/TIMP3/C3/SERPINB7/SERPINA3/SERPINB2/TNFAIP8		
GO:0061135	endopeptidase regulator activity	7/195	182/17696	0.00410014085774737	0.0788345264921425	5104/7078/718/8710/12/5055/25816	SERPINA5/TIMP3/C3/SERPINB7/SERPINA3/SERPINB2/TNFAIP8		
GO:0042379	chemokine receptor binding	4/195	66/17696	0.00605224437365116	0.1096923297089088	6347/3576/2919/7032	CCL2/CXCL8/CXCL1/TFF2		
GO:0045236	CXCR chemokine receptor binding	2/195	11/17696	0.00622367828135648	0.1096923297089088	3576/7032	CXCL8/TFF2		
GO:0031994	insulin-like growth factor I binding	2/195	12/17696	0.00741453519858673	0.1206287841923922	3488/3691	IGFBP5/ITGB4		
GO:0032036	myosin heavy chain binding	2/195	12/17696	0.00741453519858673	0.1206287841923922	10398/11151	MYL9/CORO1A		

Term	database	ID	GeneRatio	BgRatio	Pvalue	Qvalue	geneIDs	geneNames	URL
GO:0086080	protein binding involved in heterotypic cell-cell adhesion	2/195	13/17696	0.00869947350990716	0.1362917516552212	3728/342035	JUP/GLDN		
GO:0015926	glucosidase activity	2/195	14/17696	0.0100762969365736	0.1522240572918089	80162/197021	PGGHG/LCTL		
GO:0030246	carbohydrate binding	8/195	271/17696	0.0105147665787019	0.1533705607858893	1272/26232/22925/22918/176/497190/283208/7123	CNTN1/FBXO2/PLA2R1/CD93/ACAN/CLEC18B/P4HA3/CLEC3B		
GO:0061134	peptidase regulator activity	7/195	219/17696	0.0109063433306491	0.1537794409621522	5104/7078/718/8710/12/5055/25816	SERPINA5/TIMP3/C3/SERPINB7/SERPINA3/SERPINB2/TNFAIP8		
GO:0050780	dopamine receptor binding	2/195	16/17696	0.0130969962219187	0.1766440904446887	5064/1759	PALM/DNM1		
GO:0051219	phosphoprotein binding	4/195	83/17696	0.0133631463220567	0.1766440904446887	5327/2810/126669/90525	PLAT/SFN/SHE/SHF		
GO:0048038	quinone binding	2/195	17/17696	0.0147366601353387	0.1888971890075223	314/8639	AOC2/AOC3		
GO:0005178	integrin binding	5/195	132/17696	0.0155041846318824	0.1928902970378311	3553/3691/7045/5803/3791	IL1B/ITGB4/TGFBI/PTPRZ1/KDR		
GO:0008009	chemokine activity	3/195	49/17696	0.0167447548982678	0.2023723234847799	6347/3576/2919	CCL2/CXCL8/CXCL1		
GO:0004867	serine-type endopeptidase inhibitor activity	4/195	94/17696	0.0202144513860374	0.2375198037859399	5104/8710/12/5055	SERPINA5/SERPINB7/SERPINA3/SERPINB2		
GO:0003779	actin binding	10/195	431/17696	0.0214479801101542	0.2410408069791555	10788/51778/84665/6710/339768/4648/59341/4137/11151/130271	IQGAP2/MYOZ2/MYPN/SPTB/ESPNL/MYO7B/TRPV4/MAPT/CORO1A/PLEKHH2		

Term	database	ID	GeneRatio	BgRatio	Pvalue	Qvalue	geneIDs	geneNames	URL
GO:0004896	cytokine receptor activity	4/195	96/17696	0.0216537840785057	0.2410408069791555	9173/58985/10148/54756	IL1RL1/IL22RA1/EBI3/IL17RD		
GO:0051015	actin filament binding	6/195	198/17696	0.0226132310468323	0.2452665828925666	10788/6710/339768/4648/59341/11151	IQGAP2/SPTB/ESPNL/MYO7B/TRPV4/CORO1A		
GO:0004857	enzyme inhibitor activity	9/195	375/17696	0.0234843763449217	0.2483472798475477	5104/7078/10788/718/2810/8710/12/5055/25816	SERPINA5/TIMP3/IQGAP2/C3/SFN/SERPINB7/SERPINA3/SERPINB2/TNFAIP8		
GO:0030021	extracellular matrix structural constituent conferring compression resistance	2/195	22/17696	0.0241470129473277	0.2491264994321855	1634/176	DCN/ACAN		
GO:1902936	phosphatidylinositol bisphosphate binding	4/195	110/17696	0.0334706889400847	0.3370976528965667	3756/26499/55803/4137	KCNH1/PLEK2/ADAP2/MAPT		
GO:0005201	extracellular matrix structural constituent	5/195	163/17696	0.0345289795728799	0.3396687990541444	284217/7045/1290/1634/176	LAMA1/TGFBI/COL5A2/DCN/ACAN		
GO:0001968	fibronectin binding	2/195	27/17696	0.0354014878276938	0.3403370307071447	3488/1520	IGFBP5/CTSS		
GO:0005520	insulin-like growth factor binding	2/195	28/17696	0.0378536221834189	0.355824048524137	3488/3691	IGFBP5/ITGB4		
GO:0004181	metallocarboxypeptidase activity	2/195	29/17696	0.0403687978520399	0.3633191806683559	2346/57094	FOLH1/CPA6		
GO:0043236	laminin binding	2/195	29/17696	0.0403687978520399	0.3633191806683559	27076/1520	LYPD3/CTSS		

Term	database	ID	GeneRatio	BgRatio	Pvalue	Qvalue	geneIDs	geneNames	URL
GO:0043325	phosphatidylinositol-3,4-bisphosphate binding	2/195	30/17696	0.0429453433269993	0.378455838069181	26499/55803	PLEK2/ADAP2		
GO:0008237	metallopeptidase activity	5/195	181/17696	0.0502435590121786	0.433188295187178	4318/8728/9507/2346/57094	MMP9/ADAM19/ADAMTS4/FOLH1/CPA6		
GO:0030695	GTPase regulator activity	7/195	304/17696	0.0517571055008071	0.433188295187178	10156/10788/55803/8787/100271927/9754/6494	RASA4/IQGAP2/ADAP2/RGS9/RASA4B/STARD8/SIPA1		
GO:0019955	cytokine binding	4/195	128/17696	0.0532524618197004	0.433188295187178	9173/58985/94/10148	IL1RL1/IL22RA1/ACVRL1/EBI3		
GO:0140375	immune receptor activity	4/195	128/17696	0.0532524618197004	0.433188295187178	9173/58985/10148/54756	IL1RL1/IL22RA1/EBI3/IL17RD		
GO:0008022	protein C-terminus binding	5/195	187/17696	0.0562792133577198	0.436040318012534	284217/4987/1759/6494/11151	LAMA1/OPRL1/DNM1/SIPA1/CORO1A		
GO:0005501	retinoid binding	2/195	35/17696	0.056692110665221	0.436040318012534	5104/64220	SERPINA5/STRA6		
GO:0019199	transmembrane receptor protein kinase activity	3/195	79/17696	0.0566955496233791	0.436040318012534	2045/94/3791	EPHA7/ACVRL1/KDR		
GO:0019840	isoprenoid binding	2/195	36/17696	0.0596033592426537	0.450218231422187	5104/64220	SERPINA5/STRA6		

GO_ Cellular_Component

GOCCID	Term	GeneRatio	BgRatio	Pvalue	Qvalue	geneIDs	geneNames
GO:0062023	collagen-containing extracellular matrix	15/202	406/19717	2.02558569270037e-05	0.00613752464888211	4318/23213/8728/5104/9507/284217/7078/7045/5803/12/1290/1634/1520/176/7123	MMP9/SULF1/ADAM19/SERPINA5/ADAMTS4/LAMA1/TIMP3/TGFBI/PTPRZ1/SERPINA3/COL5A2/DCN/CTSS/ACAN/CLEC3B

Term	database	ID	GeneRatio	BgRatio	Pvalue	Qvalue	geneIDs	geneNames	URL
GO:0030667	secretory granule membrane	12/202	298/19717	6.12952748688902e-05	0.00928623414263687	5104/10326/55/5337/10788/22918/148229/25837/27074/84519/79930/2212	SERPINA5/SIRPB1/ACPP/PLD1/IQGAP2/CD93/ATP8B3/RAB26/LAMP3/ACRBP/DOK3/FCGR2A		
GO:0098858	actin-based cell projection	9/202	208/19717	0.000303706182635874	0.0306743244462233	793/55/10788/2596/5064/8639/339768/4648/59341	CALB1/ACPP/IQGAP2/GAP43/PALM/AOC3/ESPNL/MYO7B/TRPV4		
GO:0016327	apicolateral plasma membrane	3/202	18/19717	0.000771735875027803	0.0508764301827608	1364/3728/5064	CLDN4/JUP/PALM		
GO:0098978	glutamatergic synapse	11/202	349/19717	0.000984439220733128	0.0508764301827608	2045/5327/7476/793/254263/176/8787/1009/81832/1759/11151	EPHA7/PLAT/WNT7A/CALB1/CNIH2/ACAN/RGS9/CDH11/NETO1/DNM1/CORO1A		
GO:0098984	neuron to neuron synapse	11/202	350/19717	0.00100745406302497	0.0508764301827608	2045/3756/793/254263/2596/5064/9762/8787/4923/81832/1759	EPHA7/KCNH1/CALB1/CNIH2/GAP43/PALM/LZTS3/RGS9/NTSR1/NETO1/DNM1		
GO:0030863	cortical cytoskeleton	6/202	116/19717	0.00125189253745539	0.0541890626927122	793/9783/6710/59341/11151/130271	CALB1/RIMS3/SPTB/TRPV4/CORO1A/PLEKHH2		
GO:0070820	tertiary granule	7/202	164/19717	0.00153334661215832	0.0543036418494161	4318/5337/22918/1520/2919/79930/230	MMP9/PLD1/CD93/CTSS/CXCL1/DOK3/ALDOC		
GO:0002080	acrosomal membrane	3/202	23/19717	0.00161297946087375	0.0543036418494161	5104/148229/84519	SERPINA5/ATP8B3/ACRBP		

Term	database	ID	GeneRatio	BgRatio	Pvalue	Qvalue	geneIDs	geneNames	URL
GO:0043025	neuronal cell body	13/202	497/19717	0.0019078838045189	0.056520138064405	2045/3756/10382/57795/793/92737/3672/94/3814/6863/4923/2173/4137	EPHA7/KCNH1/TUBB4A/BRINP2/CALB1/DNER/ITGA1/ACVRL1/KISS1/TAC1/NTSR1/FABP7/MAPT		
GO:0045177	apical part of cell	11/202	384/19717	0.00209953401809929	0.056520138064405	5327/1364/5337/3814/50617/4648/5244/784/222962/59341/153562	PLAT/CLDN4/PLD1/KISS1/ATP6V0A4/MYO7B/ABCB4/CACNB3/SLC29A4/TRPV4/MARVELD2		
GO:0030864	cortical actin cytoskeleton	5/202	90/19717	0.00233381137169575	0.0565201380644055	793/6710/59341/11151/130271	CALB1/SPTB/TRPV4/CORO1A/PLEKHH2		
GO:1904724	tertiary granule lumen	4/202	55/19717	0.00242495641860483	0.0565201380644055	4318/1520/2919/230	MMP9/CTSS/CXCL1/ALDOC		
GO:0016342	catenin complex	3/202	29/19717	0.00318119373893792	0.0688501216355855	3728/64403/1009	JUP/CDH24/CDH11		
GO:0030175	filopodium	5/202	104/19717	0.00435406532847045	0.076171170227037 6	55/10788/2596/5064/59341	ACPP/IQGAP2/GAP43/PALM/TRPV4		
GO:0097060	synaptic membrane	11/202	432/19717	0.00511145179102967	0.0761711702270376	2045/3756/1272/254263/9783/5064/5138/9762/8787/81832/1759	EPHA7/KCNH1/CNTN1/CNIH2/RIMS3/PALM/PDE2A/LZTS3/RGS9/NETO1/DNM1		
GO:0045121	membrane raft	9/202	315/19717	0.00532224460244067	0.0761711702270376	23213/6404/1272/3037/3672/3791/5244/4923/4137	SULF1/SELPLG/CNTN1/HAS2/ITGA1/KDR/ABCB4/NTSR1/MAPT		
GO:0098857	membrane microdomain	9/202	316/19717	0.00543100423658374	0.0761711702270376	23213/6404/1272/3037/3672/3791/5244/4923/4137	SULF1/SELPLG/CNTN1/HAS2/ITGA1/KDR/ABCB4/NTSR1/MAPT		

Term	database	ID	GeneRatio	BgRatio	Pvalue	Qvalue	geneIDs	geneNames	URL
GO:0016324	apical plasma membrane	9/202	318/19717	0.00565368595957346	0.0761711702270376	1364/5337/3814/50617/5244/784/222962/59341/153562	CLDN4/PLD1/KISS1/ATP6V0A4/ABCB4/CACNB3/SLC29A4/TRPV4/MARVELD2		
GO:0034774	secretory granule lumen	9/202	321/19717	0.00600088224678815	0.0761711702270376	7078/718/5122/12/3728/3671/2919/230/7123	TIMP3/C3/PCSK1/SERPINA3/JUP/ISLR/CXCL1/ALDOC/CLEC3B		
GO:0043197	dendritic spine	6/202	159/19717	0.00600182430291677	0.0761711702270376	793/254263/26232/4923/1759/4137	CALB1/CNIH2/FBXO2/NTSR1/DNM1/MAPT		
GO:0014069	postsynaptic density	9/202	324/19717	0.00636431766772412	0.0761711702270376	2045/3756/254263/2596/5064/9762/8787/81832/1759	EPHA7/KCNH1/CNIH2/GAP43/PALM/LZTS3/RGS9/NETO1/DNM1		
GO:0044309	neuron spine	6/202	161/19717	0.00636981953329022	0.0761711702270376	793/254263/26232/4923/1759/4137	CALB1/CNIH2/FBXO2/NTSR1/DNM1/MAPT		
GO:0060205	cytoplasmic vesicle lumen	9/202	325/19717	0.00648915572132863	0.0761711702270376	7078/718/5122/12/3728/3671/2919/230/7123	TIMP3/C3/PCSK1/SERPINA3/JUP/ISLR/CXCL1/ALDOC/CLEC3B		
GO:0031983	vesicle lumen	9/202	327/19717	0.0067444800390495	0.0761711702270376	7078/718/5122/12/3728/3671/2919/230/7123	TIMP3/C3/PCSK1/SERPINA3/JUP/ISLR/CXCL1/ALDOC/CLEC3B		
GO:0032279	asymmetric synapse	9/202	328/19717	0.00687500271834661	0.0761711702270376	2045/3756/254263/2596/5064/9762/8787/81832/1759	EPHA7/KCNH1/CNIH2/GAP43/PALM/LZTS3/RGS9/NETO1/DNM1		

Term	database	ID	GeneRatio	BgRatio	Pvalue	Qvalue	geneIDs	geneNames	URL
GO:0098589	membrane region	9/202	328/19717	0.00687500271834661	0.0761711702270376	23213/6404/1272/3037/3672/3791/5244/4923/4137	SULF1/SELPLG/CNTN1/HAS2/ITGA1/KDR/ABCB4/NTSR1/MAPT		
GO:0098839	postsynaptic density membrane	4/202	74/19717	0.00703892002098037	0.0761711702270376	2045/254263/8787/81832	EPHA7/CNIH2/RGS9/NETO1		
GO:0031089	platelet dense granule lumen	2/202	14/19717	0.00876442027716559	0.0894186844542472	7078/7123	TIMP3/CLEC3B		
GO:0031252	cell leading edge	10/202	403/19717	0.00885333509447992	0.0894186844542472	3691/9079/26499/10788/5064/4739/59341/4137/11151/130271	ITGB4/LDB2/PLEK2/IQGAP2/PALM/NEDD9/TRPV4/MAPT/CORO1A/PLEKHH2		
GO:0099572	postsynaptic specialization	9/202	348/19717	0.0099155344861447	0.0951262327274621	2045/3756/254263/2596/5064/9762/8787/81832/1759	EPHA7/KCNH1/CNIH2/GAP43/PALM/LZTS3/RGS9/NETO1/DNM1		
GO:0098685	Schaffer collateral - CA1 synapse	4/202	82/19717	0.0100463348095009	0.0951262327274621	2045/5327/7476/1009	EPHA7/PLAT/WNT7A/CDH11		
GO:0005911	cell-cell junction	10/202	416/19717	0.0108995519741078	0.100077704489535	1364/284217/2702/3728/5244/64403/2173/59341/153562/11151	CLDN4/LAMA1/GJA5/JUP/ABCB4/CDH24/FABP7/TRPV4/MARVELD2/CORO1A		
GO:0005581	collagen trimer	4/202	87/19717	0.0122925431972299	0.109548252610608	1290/1634/342035/338761	COL5A2/DCN/GLDN/C1QL4		
GO:0098793	presynapse	11/202	491/19717	0.0126593579700068	0.109593870426059	3756/7476/793/1272/9783/25837/5138/4923/81615/1759/5338	KCNH1/WNT7A/CALB1/CNTN1/RIMS3/RAB26/PDE2A/NTSR1/TMEM163/DNM1/PLD2		
GO:0043209	myelin sheath	3/202	49/19717	0.0138145776510736	0.111469077084322	10382/5354/153562	TUBB4A/PLP1/MARVELD2		

Term	database	ID	GeneRatio	BgRatio	Pvalue	Qvalue	geneIDs	geneNames	URL
GO:0031527	filopodium membrane	2/202	18/19717	0.0143455061078409	0.111469077084322	2596/5064	GAP43/PALM		
GO:0099061	integral component of postsynaptic density membrane	3/202	50/19717	0.0145876032082113	0.111469077084322	2045/254263/81832	EPHA7/CNIH2/NETO1		
GO:0005788	endoplasmic reticulum lumen	8/202	309/19717	0.0145928695259534	0.111469077084322	3488/7476/718/1906/57333/1290/10148/283208	IGFBP5/WNT7A/C3/EDN1/RCN3/COL5A2/EBI3/P4HA3		
GO:0030027	lamellipodium	6/202	193/19717	0.0147153897141019	0.111469077084322	26499/10788/4739/59341/11151/130271	PLEK2/IQGAP2/NEDD9/TRPV4/CORO1A/PLEKHH2		
GO:0045178	basal part of cell	3/202	51/19717	0.0153846904157563	0.1136966145359555	1364/3672/1906	CLDN4/ITGA1/EDN1		
GO:0005604	basement membrane	4/202	95/19717	0.0165122818955021	0.1191243193889779	284217/7078/7045/176	LAMA1/TIMP3/TGFBI/ACAN		
GO:0099146	intrinsic component of postsynaptic density membrane	3/202	53/19717	0.0170513495577983	0.120152532930532	2045/254263/81832	EPHA7/CNIH2/NETO1		
GO:0045211	postsynaptic membrane	8/202	323/19717	0.0185354876760377	0.124414592789606	2045/3756/1272/254263/9762/8787/81832/1759	EPHA7/KCNH1/CNTN1/CNIH2/LZTS3/RGS9/NETO1/DNM1		
GO:0016459	myosin complex	3/202	55/19717	0.0188150502774553	0.124414592789606	84176/4648/10398	MYH16/MYO7B/MYL9		
GO:0098862	cluster of actin-based cell projections	5/202	150/19717	0.0192683094763048	0.124414592789606	793/339768/50617/4648/197021	CALB1/ESPNL/ATP6V0A4/MYO7B/LCTL		
GO:0042827	platelet dense granule	2/202	21/19717	0.0192986332049884	0.124414592789606	7078/7123	TIMP3/CLEC3B		
GO:0099634	postsynaptic specialization membrane	4/202	101/19717	0.0202064249259081	0.127553057344795	2045/254263/8787/81832	EPHA7/CNIH2/RGS9/NETO1		

Term	database	ID	GeneRatio	BgRatio	Pvalue	Qvalue	geneIDs	geneNames	URL
GO:0005796	Golgi lumen	4/202	102/19717	0.0208675602883526	0.129038178925935	7476/1634/9957/176	WNT7A/DCN/HS3ST1/ACAN		
GO:0009898	cytoplasmic side of plasma membrane	5/202	154/19717	0.0213169855169666	0.129180932232817	3728/5064/6710/51764/4923	JUP/PALM/SPTB/GNG13/NTSR1		
GO:0001669	acrosomal vesicle	4/202	108/19717	0.025112680820327	0.147035581348829	5104/3672/148229/84519	SERPINA5/ITGA1/ATP8B3/ACRBP		
GO:0042734	presynaptic membrane	5/202	161/19717	0.0252338291423733	0.147035581348829	3756/1272/9783/5138/1759	KCNH1/CNTN1/RIMS3/PDE2A/DNM1		
GO:0005775	vacuolar lumen	5/202	172/19717	0.0322718321952436	0.183223772013923	718/12/1634/1520/176	C3/SERPINA3/DCN/CTSS/ACAN		
GO:0044304	main axon	3/202	68/19717	0.0326537415470358	0.183223772013923	10382/1759/4137	TUBB4A/DNM1/MAPT		
GO:0098982	GABA-ergic synapse	3/202	71/19717	0.0364281408849253	0.196240693039801	793/2596/176	CALB1/GAP43/ACAN		
GO:0098562	cytoplasmic side of membrane	5/202	178/19717	0.0365795312811305	0.196240693039801	3728/5064/6710/51764/4923	JUP/PALM/SPTB/GNG13/NTSR1		
GO:0098936	intrinsic component of postsynaptic membrane	4/202	122/19717	0.0369165660173882	0.196240693039801	2045/1272/254263/81832	EPHA7/CNTN1/CNIH2/NETO1		
GO:0101002	ficolin-1-rich granule	4/202	124/19717	0.0388232597337164	0.197520261919701	4318/3728/1520/230	MMP9/JUP/CTSS/ALDOC		
GO:1904813	ficolin-1-rich granule lumen	4/202	124/19717	0.0388232597337164	0.197520261919701	4318/3728/1520/230	MMP9/JUP/CTSS/ALDOC		
GO:0005938	cell cortex	7/202	308/19717	0.0398178096252848	0.197520261919701	793/9783/6710/4739/59341/11151/130271	CALB1/RIMS3/SPTB/NEDD9/TRPV4/CORO1A/PLEKHH2		
GO:0008305	integrin complex	2/202	31/19717	0.0399819701721267	0.197520261919701	3691/3672	ITGB4/ITGA1		

Term	database	ID	GeneRatio	BgRatio	Pvalue	Qvalue	geneIDs	geneNames	URL
GO:0099060	integral component of postsynaptic specialization membrane	3/202	74/19717	0.0404166872574966	0.197520261919701	2045/254263/81832	EPHA7/CNIH2/NETO1		
GO:0098948	intrinsic component of postsynaptic specialization membrane	3/202	77/19717	0.04461668458813887	0.2145850068286667	2045/254263/81832	EPHA7/CNIH2/NETO1		
GO:0030018	Z disc	4/202	132/19717	0.04700457787195515	0.2170964615590 09	51778/3728/84665/10398	MYOZ2/JUP/MYPN/MYL9		
GO:0098636	protein complex involved in cell adhesion	2/202	34/19717	0.04728833816136682	0.2170964615590 09	3691/3672	ITGB4/ITGA1		
GO:0098686	hippocampal mossy fiber to CA3 synapse	2/202	34/19717	0.04728833816136682	0.2170964615590 09	2045/793	EPHA7/CALB1		
GO:0031253	cell projection membrane	7/202	326/19717	0.05120815659166621	0.2315831559294 57	26499/2596/5064/50617/129880/59341/4137	PLEK2/GAP43/PALM/ATP6V0A4/BBS5/TRPV4/MAPT		
GO:0043198	dendritic shaft	2/202	36/19717	0.05240800572860 54	0.2335239078789 33	254263/4923	CNIH2/NTSR1		
GO:0005902	microvillus	3/202	83/19717	0.05363806191221 95	0.2355410544840 94	10788/8639/4648	IQGAP2/AOC3/MYO7B		
GO:0005925	focal adhesion	8/202	405/19717	0.05759978371824 58	0.2493247780946 93	3691/3672/3728/2273/4739/5244/9754/59341	ITGB4/ITGA1/JUP/FHL1/NEDD9/ABCB4/STARD8/TRPV4		
GO:0031674	I band	4/202	143/19717	0.05969595504579 23	0.2547587940686 63	51778/3728/84665/10398	MYOZ2/JUP/MYPN/MYL9		
GO:0030055	cell-substrate junction	8/202	412/19717	0.06236458678148 31	0.2624509693720 75	3691/3672/3728/2273/4739/5244/9754/59341	ITGB4/ITGA1/JUP/FHL1/NEDD9/ABCB4/STARD8/TRPV4		

Term	database	ID	GeneRatio	BgRatio	Pvalue	Qvalue	geneIDs	geneNames	URL
GO:0031091	platelet alpha granule	3/202	91/19717	0.06690988625575561	0.2777218566580755	5104/12/3671	SERPINA5/SERPINA3/ISLR		
GO:0005769	early endosome	7/202	350/19717	0.0693190938010403	0.2838335867799466	3756/92737/3791/27074/8639/81615/11151	KCNH1/DNER/KDR/LAMP3/AOC3/TMEM163/CORO1A		
GO:0043202	lysosomal lumen	3/202	95/19717	0.07405505292877739	0.2991824138322447	1634/1520/176	DCN/CTSS/ACAN		
GO:0005903	brush border	3/202	99/19717	0.08152421946848807	0.3208796190487699	50617/4648/197021	ATP6V0A4/MYO7B/LCTL		
GO:0042581	specific granule	4/202	160/19717	0.0825128871168766	0.3208796190487699	5337/3728/22918/2919	PLD1/JUP/CD93/CXCL1		
GO:0098802	plasma membrane receptor complex	6/202	295/19717	0.0836082665149466	0.3208796190487699	3691/254263/3672/94/958/10148	ITGB4/CNIH2/ITGA1/ACVRL1/CD40/EBI3		
GO:0098563	intrinsic component of synaptic vesicle membrane	2/202	47/19717	0.0836616828542994	0.3208796190487699	25837/81615	RAB26/TMEM163		
GO:0099240	intrinsic component of synaptic membrane	4/202	164/19717	0.0884258993949128	0.329511028843764	2045/1272/254263/81832	EPHA7/CNTN1/CNIH2/NETO1		
GO:0019897	extrinsic component of plasma membrane	4/202	165/19717	0.0899354544036498	0.329511028843764	3728/51764/64403/1009	JUP/GNG13/CDH24/CDH11		
GO:0014704	intercalated disc	2/202	50/19717	0.0929691737621541	0.329511028843764	2702/3728	GJA5/JUP		
GO:0031225	anchored component of membrane	4/202	170/19717	0.0976675727461113	0.329511028843764	1272/27076/25837/249	CNTN1/LYPD3/RAB26/ALPL		
GO:0031256	leading edge membrane	4/202	170/19717	0.0976675727461113	0.329511028843764	26499/5064/59341/4137	PLEK2/PALM/TRPV4/MAPT		
GO:0005916	fascia adherens	1/202	10/19717	0.0978745630229002	0.329511028843764	3728	JUP		

Term	database	ID	GeneRatio	BgRatio	Pvalue	Qvalue	geneIDs	geneNames	URL
GO:0033179	proton-transporting V-type ATPase, V0 domain	1/202	10/19717	0.09787456302290 02	0.32951102884376 4	50617	ATP6V0A4		
GO:0033655	host cell cytoplasm part	1/202	10/19717	0.09787456302290 02	0.32951102884376 4	2634	GBP2		
GO:0043256	laminin complex	1/202	10/19717	0.09787456302290 02	0.32951102884376 4	284217	LAMA1		
GO:0097470	ribbon synapse	1/202	10/19717	0.09787456302290 02	0.32951102884376 4	1759	DNM1		
GO:0098981	cholinergic synapse	1/202	10/19717	0.09787456302290 02	0.32951102884376 4	5337	PLD1		
GO:0032420	stereocilium	2/202	52/19717	0.09933308618817 4	0.33074642983534 9	793/339768	CALB1/ESPNL		
GO:0005583	fibrillar collagen trimer	1/202	11/19717	0.10712149752685 2	0.33120218112894 1	1290	COL5A2		
GO:0030430	host cell cytoplasm	1/202	11/19717	0.10712149752685 2	0.33120218112894 1	2634	GBP2		
GO:0031094	platelet dense tubular network	1/202	11/19717	0.10712149752685 2	0.33120218112894 1	5104	SERPINA5		
GO:0032280	symmetric synapse	1/202	11/19717	0.10712149752685 2	0.33120218112894 1	4923	NTSR1		
GO:0035631	CD40 receptor complex	1/202	11/19717	0.10712149752685 2	0.33120218112894 1	958	CD40		
GO:0043220	Schmidt-Lanterman incisure	1/202	11/19717	0.10712149752685 2	0.33120218112894 1	153562	MARVELD2		
GO:0098643	banded collagen fibril	1/202	11/19717	0.10712149752685 2	0.33120218112894 1	1290	COL5A2		
GO:0030133	transport vesicle	7/202	392/19717	0.10917125305327 4	0.33413019873880 8	254263/5122/1906/25837/81615/1759/6494	CNIH2/PCSK1/EDN1/RAB26/TMEM163/DNM1/SIPA1		
GO:0016328	lateral plasma membrane	2/202	57/19717	0.11574369214390 7	0.33553387143021 8	1364/3728	CLDN4/JUP		

Term	database	ID	GeneRatio	BgRatio	Pvalue	Qvalue	geneIDs	geneNames	URL
GO:0032421	stereocilium bundle	2/202	57/19717	0.115743692143907	0.3355338714302188	793/339768	CALB1/ESPNL		
GO:0032589	neuron projection membrane	2/202	57/19717	0.115743692143907	0.3355338714302188	5064/4137	PALM/MAPT		
GO:0030877	beta-catenin destruction complex	1/202	12/19717	0.116274113861957	0.3355338714302188	51339	DACT1		
GO:0099144	anchored component of synaptic membrane	1/202	12/19717	0.116274113861957	0.3355338714302188	1272	CNTN1		
GO:1990454	L-type voltage-gated calcium channel complex	1/202	12/19717	0.116274113861957	0.3355338714302188	784	CACNB3		
GO:0099055	integral component of postsynaptic membrane	3/202	117/19717	0.1187996735959997	0.3395877462225221	2045/254263/81832	EPHA7/CNIH2/NETO1		
GO:0046658	anchored component of plasma membrane	2/202	59/19717	0.122489322872349	0.3421262243875877	1272/27076	CNTN1/LYPD3		
GO:0001931	uropod	1/202	13/19717	0.125333369330106	0.3421262243875877	6404	SELPLG		
GO:0031254	cell trailing edge	1/202	13/19717	0.125333369330106	0.3421262243875877	6404	SELPLG		
GO:0032591	dendritic spine membrane	1/202	13/19717	0.125333369330106	0.3421262243875877	5064	PALM		
GO:0098993	anchored component of synaptic vesicle membrane	1/202	13/19717	0.125333369330106	0.3421262243875877	25837	RAB26		
GO:0101003	ficolin-1-rich granule membrane	2/202	61/19717	0.1293282829599921	0.3478031120015311	22918/79930	CD93/DOK3		
GO:0035580	specific granule lumen	2/202	62/19717	0.1327807032591288	0.3478031120015311	3728/2919	JUP/CXCL1		
GO:0030673	axolemma	1/202	14/19717	0.1343002115649488	0.3478031120015311	4137	MAPT		

Term	database	ID	GeneRatio	BgRatio	Pvalue	Qvalue	geneIDs	geneNames	URL
GO:0034362	low-density lipoprotein particle	1/202	14/19717	0.134300211564948	0.347803112001531	7941	PLA2G7		
GO:0043218	compact myelin	1/202	14/19717	0.134300211564948	0.347803112001531	153562	MARVELD2		
GO:0099523	presynaptic cytosol	1/202	14/19717	0.134300211564948	0.347803112001531	793	CALB1		
GO:0005859	muscle myosin complex	1/202	15/19717	0.143175578629043	0.36455630524874	10398	MYL9		
GO:0043020	NADPH oxidase complex	1/202	15/19717	0.143175578629043	0.36455630524874	124056	NOXO1		
GO:0031093	platelet alpha granule lumen	2/202	67/19717	0.150340684865502	0.374341470978434	12/3671	SERPINA3/ISLR		
GO:0033646	host intracellular part	1/202	16/19717	0.151960399110057	0.374341470978434	2634	GBP2		
GO:0043656	intracellular region of host	1/202	16/19717	0.151960399110057	0.374341470978434	2634	GBP2		
GO:0098831	presynaptic active zone cytoplasmic component	1/202	16/19717	0.151960399110057	0.374341470978434	9783	RIMS3		
GO:0030017	sarcomere	4/202	204/19717	0.157674685830552	0.377353832879394	51778/3728/84665/10398	MYOZ2/JUP/MYPN/MYL9		
GO:0043204	perikaryon	3/202	134/19717	0.158676491622964	0.377353832879394	3756/3672/4923	KCNH1/ITGA1/NTSR1		
GO:0016471	vacuolar proton-transporting V-type ATPase complex	1/202	17/19717	0.160655592215979	0.377353832879394	50617	ATP6V0A4		
GO:0042599	lamellar body	1/202	17/19717	0.160655592215979	0.377353832879394	27074	LAMP3		
GO:0060077	inhibitory synapse	1/202	17/19717	0.160655592215979	0.377353832879394	57549	IGSF9		
GO:0099524	postsynaptic cytosol	1/202	17/19717	0.160655592215979	0.377353832879394	793	CALB1		

Term	database	ID	GeneRatio	BgRatio	Pvalue	Qvalue	geneIDs	geneNames	URL
GO:0044291	cell-cell contact zone	2/202	71/19717	0.164701279907573	0.383880675476881	2702/3728	GJA5/JUP		
GO:0150034	distal axon	5/202	285/19717	0.169230281846922	0.385612079431788	793/2596/4923/59341/4137	CALB1/GAP43/NTSR1/TRPV4/MAPT		
GO:0032426	stereocilium tip	1/202	18/19717	0.169262067869399	0.385612079431788	339768	ESPNL		
GO:0033643	host cell part	1/202	18/19717	0.169262067869399	0.385612079431788	2634	GBP2		
GO:0070821	tertiary granule membrane	2/202	73/19717	0.171970195260231	0.388857978834701	5337/22918	PLD1/CD93		
GO:0016460	myosin II complex	1/202	19/19717	0.177780726800836	0.396085001622451	10398	MYL9		
GO:0098644	complex of collagen trimers	1/202	19/19717	0.177780726800836	0.396085001622451	1290	COL5A2		
GO:0043296	apical junction complex	3/202	143/19717	0.181244429737566	0.398846924829635	1364/3728/153562	CLDN4/JUP/MARVELD2		
GO:0005912	adherens junction	2/202	76/19717	0.182969381357489	0.398846924829635	3728/59341	JUP/TRPV4		
GO:0030670	phagocytic vesicle membrane	2/202	76/19717	0.182969381357489	0.398846924829635	50617/11151	ATP6V0A4/CORO1A		
GO:0036019	endolysosome	1/202	20/19717	0.186212460641136	0.403016968387602	1520	CTSS		
GO:0005922	connexin complex	1/202	21/19717	0.194558152012944	0.418093050070369	2702	GJA5		
GO:0030016	myofibril	4/202	224/19717	0.198033769462244	0.42256501512014	51778/3728/84665/10398	MYOZ2/JUP/MYPN/MYL9		
GO:0005811	lipid droplet	2/202	81/19717	0.201515284253556	0.423821092484425	729359/50486	PLIN4/G0S2		
GO:0031258	lamellipodium membrane	1/202	22/19717	0.202818674621259	0.423821092484425	26499	PLEK2		
GO:0042629	mast cell granule	1/202	22/19717	0.202818674621259	0.423821092484425	8013	NR4A3		
GO:0099699	integral component of synaptic membrane	3/202	152/19717	0.204612868908694	0.424641775885851	2045/254263/81832	EPHA7/CNIH2/NETO1		

Term	database	ID	GeneRatio	BgRatio	Pvalue	Qvalue	geneIDs	geneNames	URL
GO:0033177	proton-transporting two-sector ATPase complex, proton-transporting domain	1/202	23/19717	0.210994893343093	0.431467666285572	50617	ATP6V0A4		
GO:0005766	primary lysosome	3/202	155/19717	0.212553281907633	0.431467666285572	55/718/12	ACPP/C3/SERPINA3		
GO:0042582	azurophil granule	3/202	155/19717	0.212553281907633	0.431467666285572	55/718/12	ACPP/C3/SERPINA3		
GO:0009897	external side of plasma membrane	6/202	393/19717	0.216621566201757	0.431467666285572	5104/3604/9173/3672/958/10148	SERPINA5/TNFRSF9/IL1RL1/ITGA1/CD40/EBI3		
GO:0044305	calyx of Held	1/202	24/19717	0.219087664316216	0.431467666285572	793	CALB1		
GO:0097386	glial cell projection	1/202	24/19717	0.219087664316216	0.431467666285572	4137	MAPT		
GO:0099522	region of cytosol	1/202	24/19717	0.219087664316216	0.431467666285572	793	CALB1		
GO:0043292	contractile fiber	4/202	234/19717	0.219293797386066	0.431467666285572	51778/3728/84665/10398	MYOZ2/JUP/MYPN/MYL9		
GO:0030057	desmosome	1/202	25/19717	0.227097835027034	0.443939638794782	3728	JUP		
GO:0033176	proton-transporting V-type ATPase complex	1/202	26/19717	0.235026244397567	0.454244891976799	50617	ATP6V0A4		
GO:0005623	cell	2/202	90/19717	0.235367815314711	0.454244891976799	6347/4137	CCL2/MAPT		
GO:0035578	azurophil granule lumen	2/202	91/19717	0.239153386754198	0.455745133248566	718/12	C3/SERPINA3		
GO:0035579	specific granule membrane	2/202	91/19717	0.239153386754198	0.455745133248566	5337/22918	PLD1/CD93		
GO:0001891	phagocytic cup	1/202	27/19717	0.242873722871575	0.457085329379423	11151	CORO1A		

Term	database	ID	GeneRatio	BgRatio	Pvalue	Qvalue	geneIDs	geneNames	URL
GO:0032281	AMPA glutamate receptor complex	1/202	27/19717	0.242873722871575	0.457085329379423	254263	CNIH2		
GO:0030666	endocytic vesicle membrane	3/202	167/19717	0.244921329556499	0.458093597874192	7476/50617/11151	WNT7A/ATP6V0A4/CORO1A		
GO:0030426	growth cone	3/202	171/19717	0.255886178887449	0.474288945699566	2596/59341/4137	GAP43/TRPV4/MAPT		
GO:0099568	cytoplasmic region	4/202	251/19717	0.256710848497455	0.474288945699566	10382/9783/129880/4137	TUBB4A/RIMS3/BBS5/MAPT		
GO:0005834	heterotrimeric G-protein complex	1/202	30/19717	0.265938751962506	0.482511627812212	51764	GNG13		
GO:0034451	centriolar satellite	1/202	30/19717	0.265938751962506	0.482511627812212	129880	BBS5		
GO:1905360	GTPase complex	1/202	30/19717	0.265938751962506	0.482511627812212	51764	GNG13		
GO:0030427	site of polarized growth	3/202	176/19717	0.269685670170723	0.486397369415054	2596/59341/4137	GAP43/TRPV4/MAPT		
GO:0005921	gap junction	1/202	32/19717	0.28092563451781	0.500708630934685	2702	GJA5		
GO:0031941	filamentous actin	1/202	32/19717	0.28092563451781	0.500708630934685	339768	ESPNL		
GO:0030672	synaptic vesicle membrane	2/202	104/19717	0.288486139727627	0.508205234520181	25837/81615	RAB26/TMEM163		
GO:0099501	exocytic vesicle membrane	2/202	104/19717	0.288486139727627	0.508205234520181	25837/81615	RAB26/TMEM163		
GO:0009925	basal plasma membrane	1/202	34/19717	0.29560802292447	0.511824176834939	1364	CLDN4		
GO:0030285	integral component of synaptic vesicle membrane	1/202	34/19717	0.29560802292447	0.511824176834939	81615	TMEM163		
GO:0031904	endosome lumen	1/202	34/19717	0.29560802292447	0.511824176834939	1520	CTSS		
GO:0044853	plasma membrane raft	2/202	109/19717	0.307406277556569	0.529227852838866	6404/3037	SELPLG/HAS2		

Term	database	ID	GeneRatio	BgRatio	Pvalue	Qvalue	geneIDs	geneNames	URL
GO:0001772	immunological synapse	1/202	36/19717	0.3099920735493888	0.5302389322128895	11151	CORO1A		
GO:0008021	synaptic vesicle	3/202	191/19717	0.3114934981316688	0.5302389322128895	25837/81615/1759	RAB26/TMEM163/DNM1		
GO:0005884	actin filament	2/202	111/19717	0.3149492660679988	0.5307925744358889	339768/11151	ESPNL/CORO1A		
GO:0034358	plasma lipoprotein particle	1/202	37/19717	0.317074112121769	0.5307925744358889	7941	PLA2G7		
GO:1990777	lipoprotein particle	1/202	37/19717	0.317074112121769	0.5307925744358889	7941	PLA2G7		
GO:0032994	protein-lipid complex	1/202	39/19717	0.3310219289939931	0.5510969477206655	7941	PLA2G7		
GO:0032590	dendrite membrane	1/202	40/19717	0.3378891700927844	0.5594558390060855	5064	PALM		
GO:0032993	protein-DNA complex	3/202	202/19717	0.3422986969579999	0.5636766585775744	26471/3728/3087	NUPR1/JUP/HHEX		
GO:0043679	axon terminus	2/202	119/19717	0.34491657720755	0.5647833751643577	793/4923	CALB1/NTSR1		
GO:0099738	cell cortex region	1/202	42/19717	0.3514139193060544	0.5647833751643577	9783	RIMS3		
GO:0070382	exocytic vesicle	3/202	207/19717	0.3562791872452488	0.5647833751643577	25837/81615/1759	RAB26/TMEM163/DNM1		
GO:0019898	extrinsic component of membrane	4/202	295/19717	0.3577963922248666	0.5647833751643577	3728/51764/64403/1009	JUP/GNG13/CDH24/CDH11		
GO:0005891	voltage-gated calcium channel complex	1/202	43/19717	0.3580728462844461	0.5647833751643577	784	CACNB3		
GO:0030658	transport vesicle membrane	3/202	208/19717	0.3590712685236777	0.5647833751643577	254263/25837/81615	CNIH2/RAB26/TMEM163		
GO:0032838	plasma membrane bounded cell projection cytoplasm	3/202	208/19717	0.3590712685236777	0.5647833751643577	10382/129880/4137	TUBB4A/BBS5/MAPT		
GO:0005923	bicellular tight junction	2/202	123/19717	0.3597465062928088	0.5647833751643577	1364/153562	CLDN4/MARVELD2		

Term	database	ID	GeneRatio	BgRatio	Pvalue	Qvalue	geneIDs	geneNames	URL
GO:0005930	axoneme	2/202	123/19717	0.359746506292808	0.564783375164357	10382/129880	TUBB4A/BBS5		
GO:0097014	ciliary plasm	2/202	124/19717	0.363435229687947	0.56763337420334	10382/129880	TUBB4A/BBS5		
GO:0034702	ion channel complex	4/202	301/19717	0.371739243740986	0.577625594120609	3756/1364/254263/784	KCNH1/CLDN4/CNIH2/CACNB3		
GO:0030139	endocytic vesicle	4/202	303/19717	0.376383375676433	0.581557304331077	7476/5337/50617/11151	WNT7A/PLD1/ATP6V0A4/CORO1A		
GO:0070160	tight junction	2/202	128/19717	0.37810821436707	0.581557304331077	1364/153562	CLDN4/MARVELD2		
GO:0016323	basolateral plasma membrane	3/202	217/19717	0.384110487204254	0.587805442539843	1364/5064/153562	CLDN4/PALM/MARVELD2		
GO:0034703	cation channel complex	3/202	220/19717	0.392411464627704	0.592883093690992	3756/254263/784	KCNH1/CNIH2/CACNB3		
GO:0045335	phagocytic vesicle	2/202	132/19717	0.392639982449475	0.592883093690992	50617/11151	ATP6V0A4/CORO1A		
GO:0001917	photoreceptor inner segment	1/202	49/19717	0.396621717836503	0.592883093690992	1759	DNM1		
GO:0031902	late endosome membrane	2/202	134/19717	0.399848956475011	0.592883093690992	5337/27074	PLD1/LAMP3		
GO:0034707	chloride channel complex	1/202	50/19717	0.402818708531898	0.592883093690992	1364	CLDN4		
GO:0060076	excitatory synapse	1/202	50/19717	0.402818708531898	0.592883093690992	81832	NETO1		
GO:0005782	peroxisomal matrix	1/202	51/19717	0.408952364955173	0.592883093690992	23600	AMACR		
GO:0008328	ionotropic glutamate receptor complex	1/202	51/19717	0.408952364955173	0.592883093690992	254263	CNIH2		
GO:0016469	proton-transporting two-sector ATPase complex	1/202	51/19717	0.408952364955173	0.592883093690992	50617	ATP6V0A4		
GO:0031907	microbody lumen	1/202	51/19717	0.408952364955173	0.592883093690992	23600	AMACR		

Term	database	ID	GeneRatio	BgRatio	Pvalue	Qvalue	geneIDs	geneNames	URL
GO:0043195	terminal bouton	1/202	51/19717	0.408952364955173	0.592883093690992	4923	NTSR1		
GO:0044306	neuron projection terminus	2/202	138/19717	0.41414585711292	0.595981371351983	793/4923	CALB1/NTSR1		
GO:0030315	T-tubule	1/202	52/19717	0.415023331205506	0.595981371351983	784	CACNB3		
GO:0031526	brush border membrane	1/202	53/19717	0.421032244864113	0.59876194651279	50617	ATP6V0A4		
GO:0098878	neurotransmitter receptor complex	1/202	53/19717	0.421032244864113	0.59876194651279	254263	CNIH2		
GO:0005874	microtubule	5/202	416/19717	0.422968497706609	0.59876194651279	10382/57326/1759/59341/4137	TUBB4A/PBXIP1/DNM1/TRPV4/MAPT		
GO:1902495	transmembrane transporter complex	4/202	324/19717	0.424864087459571	0.59876194651279	3756/1364/254263/784	KCNH1/CLDN4/CNIH2/CACNB3		
GO:1990351	transporter complex	4/202	332/19717	0.443112578856094	0.614908819537154	3756/1364/254263/784	KCNH1/CLDN4/CNIH2/CACNB3		
GO:1904115	axon cytoplasm	1/202	57/19717	0.44445990073381	0.614908819537154	4137	MAPT		
GO:0072562	blood microparticle	2/202	147/19717	0.445677190145601	0.614908819537154	718/12	C3/SERPINA3		
GO:0005637	nuclear inner membrane	1/202	58/19717	0.450167891580797	0.614908819537154	3756	KCNH1		
GO:0008023	transcription elongation factor complex	1/202	58/19717	0.450167891580797	0.614908819537154	4300	MLLT3		
GO:0035577	azurophil granule membrane	1/202	58/19717	0.450167891580797	0.614908819537154	55	ACPP		
GO:0005795	Golgi stack	2/202	149/19717	0.452556655962988	0.614908819537154	23213/2530	SULF1/FUT8		
GO:0031901	early endosome membrane	2/202	149/19717	0.452556655962988	0.614908819537154	3756/81615	KCNH1/TMEM163		
GO:0030173	integral component of Golgi membrane	1/202	60/19717	0.46140938597549	0.62413858906506	53947	A4GALT		

Term	database	ID	GeneRatio	BgRatio	Pvalue	Qvalue	geneIDs	geneNames	URL
GO:0012507	ER to Golgi transport vesicle membrane	1/202	62/19717	0.472422159021924	0.636195174149524	254263	CNIH2		
GO:0019005	SCF ubiquitin ligase complex	1/202	64/19717	0.483210841640485	0.645851979912532	26232	FBXO2		
GO:0001533	cornified envelope	1/202	65/19717	0.488522570638024	0.645851979912532	3728	JUP		
GO:0031228	intrinsic component of Golgi membrane	1/202	65/19717	0.488522570638024	0.645851979912532	53947	A4GALT		
GO:0005770	late endosome	3/202	256/19717	0.489062366534465	0.645851979912532	5337/27074/1520	PLD1/LAMP3/CTSS		
GO:0005765	lysosomal membrane	4/202	354/19717	0.492317736767271	0.645851979912532	55/5337/27074/50617	ACPP/PLD1/LAMP3/ATP6V0A4		
GO:0034704	calcium channel complex	1/202	66/19717	0.493779971448685	0.645851979912532	784	CACNB3		
GO:0098852	lytic vacuole membrane	4/202	355/19717	0.494513727193754	0.645851979912532	55/5337/27074/50617	ACPP/PLD1/LAMP3/ATP6V0A4		
GO:0001725	stress fiber	1/202	67/19717	0.498983597002976	0.646119785862827	10398	MYL9		
GO:0097517	contractile actin filament bundle	1/202	67/19717	0.498983597002976	0.646119785862827	10398	MYL9		
GO:0098791	Golgi subcompartment	4/202	359/19717	0.503257904290063	0.648881468084634	23213/7045/2530/4166	SULF1/TGFBI/FUT8/CHST6		
GO:0005881	cytoplasmic microtubule	1/202	69/19717	0.509231706122634	0.653801724386263	59341	TRPV4		
GO:0016529	sarcoplasmic reticulum	1/202	71/19717	0.519271210459725	0.658322915352706	497190	CLEC18B		
GO:0033116	endoplasmic reticulum-Golgi intermediate compartment membrane	1/202	71/19717	0.519271210459725	0.658322915352706	254263	CNIH2		

Term	database	ID	GeneRatio	BgRatio	Pvalue	Qvalue	geneIDs	geneNames	URL
GO:0048786	presynaptic active zone	1/202	71/19717	0.519271210459725	0.658322915352706	9783	RIMS3		
GO:0060170	ciliary membrane	1/202	72/19717	0.524214059665016	0.661820250327083	129880	BBS5		
GO:0018995	host	1/202	73/19717	0.529106335559527	0.662476114357589	2634	GBP2		
GO:0043657	host cell	1/202	73/19717	0.529106335559527	0.662476114357589	2634	GBP2		
GO:0032432	actin filament bundle	1/202	75/19717	0.53874122179305	0.671763745692569	10398	MYL9		
GO:0031594	neuromuscular junction	1/202	76/19717	0.543484846332191	0.674901264092844	2045	EPHA7		
GO:0010008	endosome membrane	5/202	479/19717	0.545917826006097	0.675003970976904	3756/5337/27074/50617/81615	KCNH1/PLD1/LAMP3/ATP6V0A4/TMEM163		
GO:0031984	organelle subcompartment	4/202	380/19717	0.548023025941645	0.675003970976904	23213/7045/2530/4166	SULF1/TGFBI/FUT8/CHST6		
GO:0042641	actomyosin	1/202	79/19717	0.557426423490317	0.683806503309984	10398	MYL9		
GO:0016528	sarcoplasm	1/202	80/19717	0.561978814897535	0.686611213362715	497190	CLEC18B		
GO:0030135	coated vesicle	3/202	289/19717	0.570694049792803	0.689227557735207	254263/1906/5244	CNIH2/EDN1/ABCB4		
GO:0016234	inclusion body	1/202	82/19717	0.570944280500122	0.689227557735207	4137	MAPT		
GO:0098889	intrinsic component of presynaptic membrane	1/202	82/19717	0.570944280500122	0.689227557735207	1272	CNTN1		
GO:0030136	clathrin-coated vesicle	2/202	188/19717	0.576185512819191	0.691564253371499	1906/5244	EDN1/ABCB4		
GO:0000932	P-body	1/202	84/19717	0.579727129888979	0.691564253371499	60489	APOBEC3G		
GO:0120111	neuron projection cytoplasm	1/202	84/19717	0.579727129888979	0.691564253371499	4137	MAPT		

Term	database	ID	GeneRatio	BgRatio	Pvalue	Qvalue	geneIDs	geneNames	URL
GO:0005791	rough endoplasmic reticulum	1/202	85/19717	0.58405123317235	0.6939902888328322	1906	EDN1		
GO:0008076	voltage-gated potassium channel complex	1/202	87/19717	0.5925670752596277	0.7008457077755622	3756	KCNH1		
GO:0001750	photoreceptor outer segment	1/202	88/19717	0.5967597115712711	0.7008457077755622	5742	PTGS1		
GO:1902554	serine/threonine protein kinase complex	1/202	88/19717	0.5967597115712711	0.7008457077755622	94	ACVRL1		
GO:0031234	extrinsic component of cytoplasmic side of plasma membrane	1/202	91/19717	0.6090817715687665	0.7117659060208189	51764	GNG13		
GO:0005774	vacuolar membrane	4/202	412/19717	0.6119835731355333	0.7117659060208189	55/5337/27074/50617	ACPP/PLD1/LAMP3/ATP6V0A4		
GO:0032580	Golgi cisterna membrane	1/202	92/19717	0.61310528538427	0.7117659060208189	2530	FUT8		
GO:0030134	COPII-coated ER to Golgi transport vesicle	1/202	93/19717	0.6170875902175122	0.7136547321981155	254263	CNIH2		
GO:0032587	ruffle membrane	1/202	94/19717	0.6210291060540422	0.7154822020318433	59341	TRPV4		
GO:0030117	membrane coat	1/202	96/19717	0.6287914294264422	0.7162548989331277	1759	DNM1		
GO:0034705	potassium channel complex	1/202	96/19717	0.6287914294264422	0.7162548989331277	3756	KCNH1		
GO:0048475	coated membrane	1/202	96/19717	0.6287914294264422	0.7162548989331277	1759	DNM1		

Term	database	ID	GeneRatio	BgRatio	Pvalue	Qvalue	geneIDs	geneNames	URL
GO:0036464	cytoplasmic ribonucleoprotein granule	2/202	212/19717	0.641589909169284	0.728096413776379	60489/4137	APOBEC3G/MAPT		
GO:0005882	intermediate filament	2/202	214/19717	0.646667358158092	0.731120184783216	3728/3866	JUP/KRT15		
GO:0035770	ribonucleoprotein granule	2/202	223/19717	0.668816033847013	0.753350402437342	60489/4137	APOBEC3G/MAPT		
GO:1902911	protein kinase complex	1/202	109/19717	0.675530081558004	0.758094869303983	94	ACVRL1		
GO:0097733	photoreceptor cell cilium	1/202	113/19717	0.688696523041191	0.767183782418289	5742	PTGS1		
GO:0030660	Golgi-associated vesicle membrane	1/202	114/19717	0.691904200165537	0.767183782418289	254263	CNIH2		
GO:1990204	oxidoreductase complex	1/202	114/19717	0.691904200165537	0.767183782418289	124056	NOXO1		
GO:0097731	9+0 non-motile cilium	1/202	115/19717	0.695078987267846	0.767183782418289	5742	PTGS1		
GO:0031985	Golgi cisterna	1/202	116/19717	0.698221219926345	0.767183782418289	2530	FUT8		
GO:0005802	trans-Golgi network	2/202	236/19717	0.698820871113689	0.767183782418289	7045/4166	TGFBI/CHST6		
GO:0005793	endoplasmic reticulum-Golgi intermediate compartment	1/202	126/19717	0.727924487033842	0.796249529138101	254263	CNIH2		
GO:0045111	intermediate filament cytoskeleton	2/202	251/19717	0.7306190355093	0.796322186184597	3728/3866	JUP/KRT15		
GO:0034399	nuclear periphery	1/202	131/19717	0.741666232979834	0.804861693860288	4137	MAPT		
GO:0036064	ciliary basal body	1/202	133/19717	0.746967534200358	0.804861693860288	129880	BBS5		
GO:0005777	peroxisome	1/202	134/19717	0.749577448318594	0.804861693860288	23600	AMACR		

Term	database	ID	GeneRatio	BgRatio	Pvalue	Qvalue	geneIDs	geneNames	URL
GO:0042579	microbody	1/202	134/19717	0.7495774483185594	0.8048616938602888	23600	AMACR		
GO:0031300	intrinsic component of organelle membrane	3/202	381/19717	0.7517355094470688	0.8048616938602888	53947/25837/81615	A4GALT/RAB26/TMEM163		
GO:0042383	sarcolemma	1/202	136/19717	0.7547171856442933	0.8052088283458488	784	CACNB3		
GO:0097730	non-motile cilium	1/202	149/19717	0.78565985557536	0.8352804780327522	5742	PTGS1		
GO:0031461	cullin-RING ubiquitin ligase complex	1/202	158/19717	0.8047744868524409	0.8526107325744405	26232	FBXO2		
GO:0000922	spindle pole	1/202	164/19717	0.8165652902353	0.8620880938721111	4739	NEDD9		
GO:0001726	ruffle	1/202	172/19717	0.8311911837146772	0.8744823911998111	59341	TRPV4		
GO:0005741	mitochondrial outer membrane	1/202	178/19717	0.8413936446611109	0.87911129080109	5138	PDE2A		
GO:0005798	Golgi-associated vesicle	1/202	178/19717	0.8413936446611109	0.87911129080109	254263	CNIH2		
GO:0030662	coated vesicle membrane	1/202	182/19717	0.8478519994388667	0.8828149684878933	254263	CNIH2		
GO:0031968	organelle outer membrane	1/202	201/19717	0.8751302116457177	0.9045702476866605	5138	PDE2A		
GO:0031301	integral component of organelle membrane	2/202	350/19717	0.8764982339998044	0.9045702476866605	53947/81615	A4GALT/TMEM163		
GO:0019867	outer membrane	1/202	203/19717	0.8777018244879933	0.9045702476866605	5138	PDE2A		
GO:0016607	nuclear speck	2/202	397/19717	0.9164630922131332	0.9413163286121332	9507/4137	ADAMTS4/MAPT		
GO:0005667	transcription factor complex	2/202	415/19717	0.9282821557130557	0.9502347742603225	9079/8013	LDB2/NR4A3		

Term	database	ID	GeneRatio	BgRatio	Pvalue	Qvalue	geneIDs	geneNames	URL
GO:0061695	transferase complex, transferring phosphorus-containing groups	1/202	259/19717	0.931767055017887	0.950590631886935	94	ACVRL1		
GO:0000151	ubiquitin ligase complex	1/202	282/19717	0.946334394698568	0.962212488569349	26232	FBXO2		
GO:0031965	nuclear membrane	1/202	296/19717	0.953639354107275	0.966099123652797	3756	KCNH1		
GO:0005743	mitochondrial inner membrane	2/202	473/19717	0.956533785794848	0.966099123652797	5138/7351	PDE2A/UCP2		
GO:0005819	spindle	1/202	347/19717	0.972818813578304	0.979282725960884	4739	NEDD9		
GO:0005635	nuclear envelope	1/202	464/19717	0.992057109580228	0.992465177778946	3756	KCNH1		
GO:0005759	mitochondrial matrix	1/202	469/19717	0.992465177778946	0.992465177778946	5138	PDE2A		

Supplementary Table S4. List of proteins specifically associating with LINC01198 identified by RNA pulldown followed by HPLC-MS.

Entry	Reviewed	Entry Name	Protein names	Gene Names	Organism	Length
Q96GR2	reviewed	ACBG1_HUMAN	Long-chain-fatty-acid--CoA ligase ACSBG1 (EC 6.2.1.3) (Acyl-CoA synthetase bubblegum family member 1) (hBG1) (hsBG) (hsBGM) (Lipidosin)	ACSBG1 BGM KIAA0631 LPD	Homo sapiens (Human)	724
Q6ZWH5	reviewed	NEK10_HUMAN	Serine/threonine-protein kinase Nek10 (EC 2.7.11.1) (Never in mitosis A-related kinase 10) (NimA-related protein kinase 10)	NEK10	Homo sapiens (Human)	1172
Q14517	reviewed	FAT1_HUMAN	Protocadherin Fat 1 (Cadherin family member 7) (Cadherin-related tumor suppressor homolog) (Protein fat homolog) [Cleaved into: Protocadherin Fat 1, nuclear form]	FAT1 CDHF7 FAT	Homo sapiens (Human)	4588
Q07955	reviewed	SRSF1_HUMAN	Serine/arginine-rich splicing factor 1 (Alternative-splicing factor 1) (ASF-1) (Splicing factor, arginine/serine-rich 1) (pre-mRNA-splicing factor SF2, P33 subunit)	SRSF1 ASF SF2 SF2P33 SFRS1 OK/SW-cl.3	Homo sapiens (Human)	248
Q96KV6	reviewed	BT2A3_HUMAN	Putative butyrophilin subfamily 2 member A3	BTN2A3P BTN2A3	Homo sapiens (Human)	586
Q92804	reviewed	RBP56_HUMAN	TATA-binding protein-associated factor 2N (68 kDa TATA-binding protein-associated factor) (TAF(II)68) (TAFII68) (RNA-binding protein 56)	TAF15 RBP56 TAF2N	Homo sapiens (Human)	592
Q15937	reviewed	ZNF79_HUMAN	Zinc finger protein 79 (ZNFpT7)	ZNF79	Homo sapiens (Human)	498
P14678	reviewed	RSMB_HUMAN	Small nuclear ribonucleoprotein-associated proteins B and B' (snRNP-B) (Sm protein B/B') (Sm-B/B') (SmB/B')	SNRPB COD SNRPB1	Homo sapiens (Human)	240
Q8IUE6	reviewed	H2A2B_HUMAN	Histone H2A type 2-B (H2A-clustered histone 21)	H2AC21 HIST2H2AB	Homo sapiens (Human)	130
P16989	reviewed	YBOX3_HUMAN	Y-box-binding protein 3 (Cold shock domain-containing protein A) (DNA-binding protein A) (Single-strand DNA-binding protein NF-GMB)	YBX3 CSDA DBPA	Homo sapiens (Human)	372
O43251	reviewed	RFOX2_HUMAN	RNA binding protein fox-1 homolog 2 (Fox-1 homolog B) (Hexaribonucleotide-binding protein 2) (RNA-binding motif protein 9) (RNA-binding protein 9) (Repressor of tamoxifen transcriptional activity)	RBFOX2 FOX2 HRNBP2 RBM9 RTA	Homo sapiens (Human)	390
P27348	reviewed	1433T_HUMAN	14-3-3 protein theta (14-3-3 protein T-cell) (14-3-3 protein tau) (Protein HS1)	YWHAQ	Homo sapiens (Human)	245

Entry	Reviewed	Entry Name	Protein names	Gene Names	Organism	Length
Q99832	reviewed	TCPH_HUMAN	T-complex protein 1 subunit eta (TCP-1-eta) (CCT-eta) (HIV-1 Nef-interacting protein) [Cleaved into: T-complex protein 1 subunit eta, N-terminally processed]	CCT7 CCTH NIP7-1	Homo sapiens (Human)	543
P56270	reviewed	MAZ_HUMAN	Myc-associated zinc finger protein (MAZI) (Pur-1) (Purine-binding transcription factor) (Serum amyloid A-activating factor-1) (SAF-1) (Transcription factor Zif87) (ZF87) (Zinc finger protein 801)	MAZ ZNF801	Homo sapiens (Human)	477
P21127	reviewed	CD11B_HUMAN	Cyclin-dependent kinase 11B (EC 2.7.11.22) (Cell division cycle 2-like protein kinase 1) (CLK-1) (Cell division protein kinase 11B) (Galactosyltransferase-associated protein kinase p58/GTA) (PITSLRE serine/threonine-protein kinase CDC2L1) (p58 CLK-1)	CDK11B CDC2L1 CDK11 PITSLREA PK58	Homo sapiens (Human)	795
Q96MN5	reviewed	TEAN2_HUMAN	Transcription elongation factor A N-terminal and central domain-containing protein 2	TCEANC2 C1orf83	Homo sapiens (Human)	208
P02545	reviewed	LMNA_HUMAN	Prelamin-A/C [Cleaved into: Lamin-A/C (70 kDa lamin) (Renal carcinoma antigen NY-REN-32)]	LMNA LMN1	Homo sapiens (Human)	664
P0C091	reviewed	FREM3_HUMAN	FRAS1-related extracellular matrix protein 3	FREM3	Homo sapiens (Human)	2139
Q969S9	reviewed	RRF2M_HUMAN	Ribosome-releasing factor 2, mitochondrial (RRF2mt) (Elongation factor G 2, mitochondrial) (EF-G2mt) (mEF-G 2) (Elongation factor G2) (hEFG2)	GFM2 EFG2 MSTP027	Homo sapiens (Human)	779
P08651	reviewed	NFIC_HUMAN	Nuclear factor 1 C-type (NF1-C) (Nuclear factor 1/C) (CCAAT-box-binding transcription factor) (CTF) (Nuclear factor I/C) (NF-I/C) (NFI-C) (TGGCA-binding [illegible]	NFIC NFI	Homo sapiens (Human)	508
Q5VW22	reviewed	AGAP6_HUMAN	Arf-GAP with GTPase, ANK repeat and PH domain-containing protein 6 (AGAP-6) (Centaurin-gamma-like family member 3)	AGAP6 CTGLF3	Homo sapiens (Human)	663
Q5T8P6	reviewed	RBM26_HUMAN	RNA-binding protein 26 (CTCL tumor antigen se70-2) (RNA-binding motif protein 26)	RBM26 C13orf10 PRO1777	Homo sapiens (Human)	1007
Q5T8P6	reviewed	RBM26_HUMAN	RNA-binding protein 26 (CTCL tumor antigen se70-2) (RNA-binding motif protein 26)	RBM26 C13orf10 PRO1777	Homo sapiens (Human)	1007
Q13085	reviewed	ACACA_HUMAN	Acetyl-CoA carboxylase 1 (ACC1) (EC 6.4.1.2) (Acetyl-Coenzyme A carboxylase alpha) (ACC-alpha)	ACACA ACAC ACC1 ACCA	Homo sapiens (Human)	2346
Q5T5N4	reviewed	CF118_HUMAN	Uncharacterized protein C6orf118	C6orf118	Homo sapiens (Human)	469

Entry	Reviewed	Entry Name	Protein names	Gene Names	Organism	Length
O95049	reviewed	ZO3_HUMAN	Tight junction protein ZO-3 (Tight junction protein 3) (Zona occludens protein 3) (Zonula occludens protein 3)	TJP3 ZO3	Homo sapiens (Human)	919
P48730	reviewed	KC1D_HUMAN	Casein kinase I isoform delta (CKI-delta) (CKId) (EC 2.7.11.1) (Tau-protein kinase CSNK1D) (EC 2.7.11.26)	CSNK1D HCKID	Homo sapiens (Human)	415
Q9UDY2	reviewed	ZO2_HUMAN	Tight junction protein ZO-2 (Tight junction protein 2) (Zona occludens protein 2) (Zonula occludens protein 2)	TJP2 X104 ZO2	Homo sapiens (Human)	1190
Q9UDY2	reviewed	ZO2_HUMAN	Tight junction protein ZO-2 (Tight junction protein 2) (Zona occludens protein 2) (Zonula occludens protein 2)	TJP2 X104 ZO2	Homo sapiens (Human)	1190
Q14669	reviewed	TRIPC_HUMAN	E3 ubiquitin-protein ligase TRIP12 (EC 2.3.2.26) (E3 ubiquitin-protein ligase for Arf) (ULF) (HECT-type E3 ubiquitin transferase TRIP12) (Thyroid receptor-interacting protein 12) (TR-interacting protein 12) (TRIP-12)	TRIP12 KIAA0045 ULF	Homo sapiens (Human)	1992
Q9Y3E1	reviewed	HDGR3_HUMAN	Hepatoma-derived growth factor-related protein 3 (HRP-3) (Hepatoma-derived growth factor 2) (HDGF-2)	HDGFL3 HDGF2 HDGFRP3 CGI-142	Homo sapiens (Human)	203
O75475	reviewed	PSIP1_HUMAN	PC4 and SFRS1-interacting protein (CLL-associated antigen KW-7) (Dense fine speckles 70 kDa protein) (DFS 70) (Lens epithelium-derived growth factor) (Transcriptional coactivator p75/p52)	PSIP1 DFS70 LEDGF PSIP2	Homo sapiens (Human)	530
O43704	reviewed	ST1B1_HUMAN	Sulfotransferase 1B1 (ST1B1) (EC 2.8.2.1) (Sulfotransferase 1B2) (Sulfotransferase family cytosolic 1B member 1) (Thyroid hormone [illegible]	SULT1B1 ST1B2 SULT1B2	Homo sapiens (Human)	296
Q7Z478	reviewed	DHX29_HUMAN	ATP-dependent RNA helicase DHX29 (EC 3.6.4.13) (DEAH box protein 29) (Nucleic acid helicase DDXx)	DHX29 DDX29	Homo sapiens (Human)	1369
Q9NQ90	reviewed	ANO2_HUMAN	Anoctamin-2 (Transmembrane protein 16B)	ANO2 C12orf3 TMEM16B	Homo sapiens (Human)	1003
Q13698	reviewed	CAC1S_HUMAN	Voltage-dependent L-type calcium channel subunit alpha-1S (Calcium channel, L type, alpha-1 polypeptide, isoform 3, skeletal muscle) (Voltage-gated calcium channel subunit alpha Cav1.1)	CACNA1S CACH1 CACN1 CACNL1A3	Homo sapiens (Human)	1873
P12035	reviewed	K2C3_HUMAN	Keratin, type II cytoskeletal 3 (65 kDa cytokeratin) (Cytokeratin-3) (CK-3) (Keratin-3) (K3) (Type-II keratin Kb3)	KRT3	Homo sapiens (Human)	628
P35908	reviewed	K22E_HUMAN	Keratin, type II cytoskeletal 2 epidermal (Cytokeratin-2e) (CK-2e) (Epithelial keratin-2e) (Keratin-2 epidermis) (Keratin-2e) (K2e) (Type-II keratin Kb2)	KRT2 KRT2A KRT2E	Homo sapiens (Human)	639

Entry	Reviewed	Entry Name	Protein names	Gene Names	Organism	Length
Q9P1Z3	reviewed	HCN3_HUMAN	Potassium/sodium hyperpolarization-activated cyclic nucleotide-gated channel 3	HCN3 KIAA1535	Homo sapiens (Human)	774
Q9UHJ3	reviewed	SMBT1_HUMAN	Scm-like with four MBT domains protein 1 (hSFMBT) (Renal ubiquitous protein 1)	SFMBT1 RU1	Homo sapiens (Human)	866
Q9UN81	reviewed	LORF1_HUMAN	LINE-1 retrotransposable element ORF1 protein (L1ORF1p) (LINE retrotransposable element 1) (LINE1 retrotransposable element 1)	L1RE1 LRE1	Homo sapiens (Human)	338
Q14CW9	reviewed	AT7L3_HUMAN	Ataxin-7-like protein 3 (SAGA-associated factor 11 homolog)	ATXN7L3	Homo sapiens (Human)	347
Q7KZI7	reviewed	MARK2_HUMAN	Serine/threonine-protein kinase MARK2 (EC 2.7.11.1) (EC 2.7.11.26) (ELKL motif kinase 1) (EMK-1) (MAP/microtubule affinity-regulating kinase 2) (PAR1 homolog) (PAR1 homolog b) (Par-1b) (Par1b)	MARK2 EMK1	Homo sapiens (Human)	788
P06899	reviewed	H2B1J_HUMAN	Histone H2B type 1-J (Histone H2B.1) (Histone H2B.r) (H2B/r)	H2BC11 H2BFR	Homo sapiens (Human)	126
Q9Y3D9	reviewed	RT23_HUMAN	28S ribosomal protein S23, mitochondrial (MRP-S23) (S23mt) (Mitochondrial small ribosomal subunit protein mS23)	MRPS23 CGI-138 HSPC329	Homo sapiens (Human)	190
P48730	reviewed	KC1D_HUMAN	Casein kinase I isoform delta (CKI-delta) (CKId) (EC 2.7.11.1) (Tau-protein kinase CSNK1D) (EC 2.7.11.26)	CSNK1D HCKID	Homo sapiens (Human)	415
Q15654	reviewed	TRIP6_HUMAN	Thyroid receptor-interacting protein 6 (TR-interacting protein 6) (TRIP-6) (Opa-interacting protein 1) (OIP-1) (Zyxin-related protein 1) (ZRP-1)	TRIP6 OIP1	Homo sapiens (Human)	476
P35227	reviewed	PCGF2_HUMAN	Polycomb group RING finger protein 2 (DNA-binding protein Mel-18) (RING finger protein 110) (Zinc finger protein 144)	PCGF2 MEL18 RNF110 ZNF144	Homo sapiens (Human)	344
O43709	reviewed	BUD23_HUMAN	Probable 18S rRNA (guanine-N(7))-methyltransferase (EC 2.1.1.-) (Bud site selection protein 23 homolog) (Metastasis-related methyltransferase 1) (Williams-Beuren syndrome chromosomal region 22 protein) (rRNA methyltransferase and ribosome maturation factor)	BUD23 MERM1 WBSCR22 HUSSY-03 PP3381	Homo sapiens (Human)	281
P17096	reviewed	HMGA1_HUMAN	High mobility group protein HMG-I/HMG-Y (HMG-I(Y)) (High mobility group AT-hook protein 1) (High mobility group protein A1) (High mobility group protein R)	HMGA1 HMGIY	Homo sapiens (Human)	107

Entry	Reviewed	Entry Name	Protein names	Gene Names	Organism	Length
P16989	reviewed	YBOX3_HUMAN	Y-box-binding protein 3 (Cold shock domain-containing protein A) (DNA-binding protein A) (Single-strand DNA-binding protein NF-GMB)	YBX3 CSDA DBPA	Homo sapiens (Human)	372
Q9NW75	reviewed	GPTC2_HUMAN	G patch domain-containing protein 2	GPATCH2 GPATC2	Homo sapiens (Human)	528
Q9NW75	reviewed	GPTC2_HUMAN	G patch domain-containing protein 2	GPATCH2 GPATC2	Homo sapiens (Human)	528
P08574	reviewed	CY1_HUMAN	Cytochrome c1, heme protein, mitochondrial (EC 7.1.1.8) (Complex III subunit 4) (Complex III subunit IV) (Cytochrome b-c1 complex subunit 4) (Ubiquinol-cytochrome-c reductase complex cytochrome c1 subunit) (Cytochrome c-1)	CYC1	Homo sapiens (Human)	325
Q9UQ84	reviewed	EXO1_HUMAN	Exonuclease 1 (hExo1) (EC 3.1.-.-) (Exonuclease I) (hExoI)	EXO1 EXOI HEX1	Homo sapiens (Human)	846
Q92945	reviewed	FUBP2_HUMAN	Far upstream element-binding protein 2 (FUSE-binding protein 2) (KH type-splicing regulatory protein) (KSRP) (p75)	KHSRP FUBP2	Homo sapiens (Human)	711
O95639	reviewed	CPSF4_HUMAN	Cleavage and polyadenylation specificity factor subunit 4 (Cleavage and polyadenylation specificity factor 30 kDa subunit) (CPSF 30 kDa subunit) (NS1 effector domain-binding protein 1) (Neb-1) (No arches homolog)	CPSF4 CPSF30 NAR NEB1	Homo sapiens (Human)	269
Q9BX40	reviewed	LS14B_HUMAN	Protein LSM14 homolog B (RNA-associated protein 55B) (hRAP55B)	LSM14B C20orf40 FAM61B RAP55B	Homo sapiens (Human)	385
Q7Z3B3	reviewed	KANL1_HUMAN	KAT8 regulatory NSL complex subunit 1 (MLL1/MLL complex subunit KANSL1) (MSL1 homolog 1) (hMSL1v1) (NSL complex protein NSL1) (Non-specific lethal 1 homolog)	KANSL1 CENP-36 KIAA1267 MSL1V1 NSL1	Homo sapiens (Human)	1105
Q14938	reviewed	NFIX_HUMAN	Nuclear factor 1 X-type (NF1-X) (Nuclear factor 1/X) (CCAAT-box-binding transcription factor) (CTF) (Nuclear factor I/X) (NF-I/X) (NFI-X) (TGGCA-binding protein)	NFIX	Homo sapiens (Human)	502
Q15149	reviewed	PLEC_HUMAN	Plectin (PCN) (PLTN) (Hemidesmosomal protein 1) (HD1) (Plectin-1)	PLEC PLEC1	Homo sapiens (Human)	4684
Q3YEC7	reviewed	RABL6_HUMAN	Rab-like protein 6 (GTP-binding protein Parf) (Partner of ARF) (Rab-like protein 1)	RABL6 C9orf86 PARF	Homo sapiens (Human)	729
P52298	reviewed	NCBP2_HUMAN	Nuclear cap-binding protein subunit 2 (20 kDa nuclear cap-binding protein) (Cell proliferation-inducing gene 55 protein) (NCBP 20 kDa subunit) (CBP20) (NCBP-interacting protein 1) (NIP1)	NCBP2 CBP20 PIG55	Homo sapiens (Human)	156

Entry	Reviewed	Entry Name	Protein names	Gene Names	Organism	Length
Q9QWL7	reviewed	K1C17_MOUSE	Keratin, type I cytoskeletal 17 (Cytokeratin-17) (CK-17) (Keratin-17) (K17)	Krt17 Krt1-17	Mus musculus (Mouse)	433
Q6SJ96	reviewed	TBPL2_HUMAN	TATA box-binding protein-like 2 (TBP-like 2) (TATA box-binding protein-related factor 3) (TBP-related factor 3)	TBPL2 TBP2 TRF3	Homo sapiens (Human)	375
Q13247	reviewed	SRSF6_HUMAN	Serine/arginine-rich splicing factor 6 (Pre-mRNA-splicing factor SRP55) (Splicing factor, arginine/serine-rich 6)	SRSF6 SFRS6 SRP55	Homo sapiens (Human)	344
Q08170	reviewed	SRSF4_HUMAN	Serine/arginine-rich splicing factor 4 (Pre-mRNA-splicing factor SRP75) (SRP001LB) (Splicing factor, arginine/serine-rich 4)	SRSF4 SFRS4 SRP75	Homo sapiens (Human)	494
Q13243	reviewed	SRSF5_HUMAN	Serine/arginine-rich splicing factor 5 (Delayed-early protein HRS) (Pre-mRNA-splicing factor SRP40) (Splicing factor, arginine/serine-rich 5)	SRSF5 HRS SFRS5 SRP40	Homo sapiens (Human)	272
Q00577	reviewed	PURA_HUMAN	Transcriptional activator protein Pur-alpha (Purine-rich single-stranded DNA-binding protein alpha)	PURA PUR1	Homo sapiens (Human)	322
P08727	reviewed	K1C19_HUMAN	Keratin, type I cytoskeletal 19 (Cytokeratin-19) (CK-19) (Keratin-19) (K19)	KRT19	Homo sapiens (Human)	400
P05783	reviewed	K1C18_HUMAN	Keratin, type I cytoskeletal 18 (Cell proliferation-inducing gene 46 protein) (Cytokeratin-18) (CK-18) (Keratin-18) (K18)	KRT18 CYK18 PIG46	Homo sapiens (Human)	430
P07093	reviewed	GDN_HUMAN	Glia-derived nexin (GDN) (Peptidase inhibitor 7) (PI-7) (Protease nexin 1) (PN-1) (Protease nexin I) (Serpin E2)	SERPINE2 PI7 PN1	Homo sapiens (Human)	398
P07093	reviewed	GDN_HUMAN	Glia-derived nexin (GDN) (Peptidase inhibitor 7) (PI-7) (Protease nexin 1) (PN-1) (Protease nexin I) (Serpin E2)	SERPINE2 PI7 PN1	Homo sapiens (Human)	398
O96028	reviewed	NSD2_HUMAN	Histone-lysine N-methyltransferase NSD2 (EC 2.1.1.357) (Multiple myeloma SET domain-containing protein) (MMSET) (Nuclear SET domain-containing protein 2) (Protein trithorax-5) (Wolf-Hirschhorn syndrome candidate 1 protein)	NSD2 KIAA1090 MMSET TRX5 WHSC1	Homo sapiens (Human)	1365
Q9Y399	reviewed	RT02_HUMAN	28S ribosomal protein S2, mitochondrial (MRP-S2) (S2mt) (Mitochondrial small ribosomal subunit protein uS2m)	MRPS2 CGI-91	Homo sapiens (Human)	296
Q13627	reviewed	DYR1A_HUMAN	Dual specificity tyrosine-phosphorylation-regulated kinase 1A (EC 2.7.11.23) (EC 2.7.12.1) (Dual specificity YAK1-related kinase) (HP86) (Protein kinase minibrain homolog) (MNBH) (hMNB)	DYRK1A DYRK MNB MNBH	Homo sapiens (Human)	763
Q9Y6C2	reviewed	EMIL1_HUMAN	EMILIN-1 (Elastin microfibril interface-located protein 1) (Elastin microfibril interfacer 1)	EMILIN1 EMI	Homo sapiens (Human)	1016

Entry	Reviewed	Entry Name	Protein names	Gene Names	Organism	Length
P78332	reviewed	RBM6_HUMAN	RNA-binding protein 6 (Lung cancer antigen NY-LU-12) (Protein G16) (RNA-binding motif protein 6) (RNA-binding protein DEF-3)	RBM6 DEF3	Homo sapiens (Human)	1123
Q8WY36	reviewed	BBX_HUMAN	HMG box transcription factor BBX (Bobby sox homolog) (HMG box-containing protein 2)	BBX HBP2	Homo sapiens (Human)	941
P51946	reviewed	CCNH_HUMAN	Cyclin-H (MO15-associated protein) (p34) (p37)	CCNH	Homo sapiens (Human)	323
P82912	reviewed	RT11_HUMAN	28S ribosomal protein S11, mitochondrial (MRP-S11) (S11mt) (Cervical cancer proto-oncogene 2 protein) (HCC-2) (Mitochondrial small ribosomal subunit protein uS11m)	MRPS11 RPMS11 HCC2	Homo sapiens (Human)	194
Q5T8P6	reviewed	RBM26_HUMAN	RNA-binding protein 26 (CTCL tumor antigen se70-2) (RNA-binding motif protein 26)	RBM26 C13orf10 PRO1777	Homo sapiens (Human)	1007
Q14978	reviewed	NOLC1_HUMAN	Nucleolar and coiled-body phosphoprotein 1 (140 kDa nucleolar phosphoprotein) (Nopp140) (Hepatitis C virus NS5A-transactivated protein 13) (HCV NS5A-transactivated protein 13) (Nucleolar 130 kDa protein) (Nucleolar phosphoprotein p130)	NOLC1 KIAA0035 NS5ATP13	Homo sapiens (Human)	699
Q9H9B1	reviewed	EHMT1_HUMAN	Histone-lysine N-methyltransferase EHMT1 (EC 2.1.1.-) (EC 2.1.1.367) (Euchromatic histone-lysine N-methyltransferase 1) (Eu-HMTase1) (G9a-like protein 1) (GLP) (GLP1) (Histone H3-K9 methyltransferase 5) (H3-K9-HMTase 5) (Lysine N-methyltransferase 1D)	EHMT1 EUHMTASE1 GLP KIAA1876 KMT1D	Homo sapiens (Human)	1298
Q13469	reviewed	NFAC2_HUMAN	Nuclear factor of activated T-cells, cytoplasmic 2 (NF-ATc2) (NFATc2) (NFAT pre-existing subunit) (NF-ATp) (T-cell transcription factor NFAT1)	NFATC2 NFAT1 NFATP	Homo sapiens (Human)	925
P24928	reviewed	RPB1_HUMAN	DNA-directed RNA polymerase II subunit RPB1 (RNA polymerase II subunit B1) (EC 2.7.7.6) (DNA-directed RNA polymerase II subunit A) (DNA-directed RNA polymerase III largest subunit) (RNA-directed RNA polymerase II subunit RPB1) (EC 2.7.7.48)	POLR2A POLR2	Homo sapiens (Human)	1970
O95235	reviewed	KI20A_HUMAN	Kinesin-like protein KIF20A (GG10_2) (Mitotic kinesin-like protein 2) (MKlp2) (Rab6-interacting kinesin-like protein) (Rabkinesin-6)	KIF20A MKLP2 RAB6KIFL	Homo sapiens (Human)	890
P29692	reviewed	EF1D_HUMAN	Elongation factor 1-delta (EF-1-delta) (Antigen NY-CO-4)	EEF1D EF1D	Homo sapiens (Human)	281

Entry	Reviewed	Entry Name	Protein names	Gene Names	Organism	Length
O95347	reviewed	SMC2_HUMAN	Structural maintenance of chromosomes protein 2 (SMC protein 2) (SMC-2) (Chromosome-associated protein E) (hCAP-E) (XCAP-E homolog)	SMC2 CAPE SMC2L1 PRO0324	Homo sapiens (Human)	1197
Q9Y6M4	reviewed	KC1G3_HUMAN	Casein kinase I isoform gamma-3 (CKI-gamma 3) (EC	CSNK1G3	Homo sapiens (Human)	447
Q9Y6M4	reviewed	KC1G3_HUMAN	Casein kinase I isoform gamma-3 (CKI-gamma 3) (EC	CSNK1G3	Homo sapiens (Human)	447
Q8N474	reviewed	SFRP1_HUMAN	Secreted frizzled-related protein 1 (FRP-1) (sFRP-1) (Secreted apoptosis-related protein 2) (SARP-2)	SFRP1 FRP FRP1 SARP2	Homo sapiens (Human)	314
P49760	reviewed	CLK2_HUMAN	Dual specificity protein kinase CLK2 (EC 2.7.12.1) (CDC-like kinase 2)	CLK2	Homo sapiens (Human)	499
Q9Y5T4	reviewed	DJC15_HUMAN	DnaJ homolog subfamily C member 15 (Cell growth-inhibiting gene 22 protein) (Methylation-controlled J protein) (MCJ)	DNAJC15 DNAJD1 GIG22 HSD18	Homo sapiens (Human)	150
Q9NTJ3	reviewed	SMC4_HUMAN	Structural maintenance of chromosomes protein 4 (SMC protein 4) (SMC-4) (Chromosome-associated polypeptide C) (hCAP-C) (XCAP-C homolog)	SMC4 CAPC SMC4L1	Homo sapiens (Human)	1288
Q9UKS6	reviewed	PACN3_HUMAN	Protein kinase C and casein kinase substrate in neurons protein 3 (SH3 domain-containing protein 6511)	PACSIN3	Homo sapiens (Human)	424
Q8IX01	reviewed	SUGP2_HUMAN	SURP and G-patch domain-containing protein 2 (Arginine/serine-rich-splicing factor 14) (Splicing factor, arginine/serine-rich 14)	SUGP2 KIAA0365 SFRS14	Homo sapiens (Human)	1082
Q13889	reviewed	TF2H3_HUMAN	General transcription factor IIH subunit 3 (Basic transcription factor 2 34 kDa subunit) (BTF2 p34) (General transcription factor IIH polypeptide 3) (TFIIH basal transcription factor complex p34 subunit)	GTF2H3	Homo sapiens (Human)	308
O95819	reviewed	M4K4_HUMAN	Mitogen-activated protein kinase kinase kinase kinase 4 (EC 2.7.11.1) (HPK/GCK-like kinase HGK) (MAPK/ERK kinase kinase kinase 4) (MEK kinase kinase 4) (MEKKK 4) (Nck-interacting kinase)	MAP4K4 HGK KIAA0687 NIK	Homo sapiens (Human)	1239
P62244	reviewed	RS15A_HUMAN	40S ribosomal protein S15a (Small ribosomal subunit protein uS8)	RPS15A OK/SW-cl.82	Homo sapiens (Human)	130
Q8N567	reviewed	ZCHC9_HUMAN	Zinc finger CCHC domain-containing protein 9	ZCCHC9	Homo sapiens (Human)	271
P05023	reviewed	AT1A1_HUMAN	Sodium/potassium-transporting ATPase subunit alpha-1 (Na(+)/K(+) ATPase alpha-1 subunit) (EC 7.2.2.13) (Sodium pump subunit alpha-1)	ATP1A1	Homo sapiens (Human)	1023

Entry	Reviewed	Entry Name	Protein names	Gene Names	Organism	Length
P62861	reviewed	RS30_HUMAN	FAU ubiquitin-like and ribosomal protein S30 [Cleaved into: Ubiquitin-like protein FUBI; 40S ribosomal protein S30 (Small ribosomal subunit protein eS30)]	FAU	Homo sapiens (Human)	133
Q92917	reviewed	GPKOW_HUMAN	G-patch domain and KOW motifs-containing protein (G-patch domain-containing protein 5) (Protein MOS2 homolog) (Protein T54)	GPKOW GPATC5 GPATCH5 T54	Homo sapiens (Human)	476
Q9UHK0	reviewed	NUFP1_HUMAN	FMR1-interacting protein NUFIP1 (Nuclear FMR1-interacting protein 1) (Nuclear FMRP-interacting protein 1)	NUFIP1	Homo sapiens (Human)	495
Q9H7E2	reviewed	TDRD3_HUMAN	Tudor domain-containing protein 3	TDRD3	Homo sapiens (Human)	651
P17482	reviewed	HXB9_HUMAN	Homeobox protein Hox-B9 (Homeobox protein Hox-2.5) (Homeobox protein Hox-2E)	HOXB9 HOX2E	Homo sapiens (Human)	250
Q5T5P2	reviewed	SKT_HUMAN	Sickle tail protein homolog	KIAA1217 SKT	Homo sapiens (Human)	1943
Q7L804	reviewed	RFIP2_HUMAN	Rab11 family-interacting protein 2 (Rab11-FIP2)	RAB11FIP2 KIAA0941	Homo sapiens (Human)	512
Q7Z4H7	reviewed	HAUS6_HUMAN	HAUS augmin-like complex subunit 6	HAUS6 DGT6 FAM29A KIAA1574	Homo sapiens (Human)	955
Q9NVI7	reviewed	ATD3A_HUMAN	ATPase family AAA domain-containing protein 3A	ATAD3A	Homo sapiens (Human)	634
P67809	reviewed	YBOX1_HUMAN	Y-box-binding protein 1 (YB-1) (CCAAT-binding transcription factor I subunit A) (CBF-A) (DNA-binding protein B) (DBPB) (Enhancer factor I subunit A) (EFI-A) (Nuclease-sensitive element-binding protein 1) (Y-box transcription [illegible]	YBX1 NSEP1 YB1	Homo sapiens (Human)	324
O95785	reviewed	WIZ_HUMAN	Protein Wiz (Widely-interspaced zinc finger-containing protein) (Zinc finger [illegible]	WIZ ZNF803	Homo sapiens (Human)	1651
Q5SXM2	reviewed	SNPC4_HUMAN	snRNA-activating protein complex subunit 4 (SNAPc subunit 4) (Proximal sequence element-binding transcription factor subunit alpha) (PSE-binding factor subunit alpha) (PTF subunit alpha) (snRNA-activating protein complex 190 kDa subunit) (SNAPc 190 kDa subunit)	SNAPC4 SNAP190	Homo sapiens (Human)	1469
Q92796	reviewed	DLG3_HUMAN	Disks large homolog 3 (Neuroendocrine-DLG) (Synapse-associated protein 102) (SAP-102) (SAP102) [illegible]	DLG3 KIAA1232	Homo sapiens (Human)	817
Q02241	reviewed	KIF23_HUMAN	Kinesin-like protein KIF23 (Kinesin-like protein 5) (Mitotic kinesin-like protein 1)	KIF23 KNSL5 MKLP1	Homo sapiens (Human)	960
P42694	reviewed	HELZ_HUMAN	Probable helicase with zinc finger domain (EC 3.6.4.-) (Down-regulated in human cancers protein)	HELZ DRHC KIAA0054	Homo sapiens (Human)	1942

Entry	Reviewed	Entry Name	Protein names	Gene Names	Organism	Length
Q7L2E3	reviewed	DHX30_HUMAN	ATP-dependent RNA helicase DHX30 (EC 3.6.4.13) (DEAH box protein 30)	DHX30 DDX30 KIAA0890	Homo sapiens (Human)	1194
Q92979	reviewed	NEP1_HUMAN	Ribosomal RNA small subunit methyltransferase NEP1 (EC 2.1.1.-) (18S rRNA (pseudouridine(1248)-N1)-methyltransferase) (18S rRNA Psi1248 methyltransferase) (Nucleolar protein EMG1 homolog) (Protein C2f) (Ribosome biogenesis protein NEP1)	EMG1 C2F	Homo sapiens (Human)	244
Q8IXT5	reviewed	RB12B_HUMAN	RNA-binding protein 12B (RNA-binding motif protein 12B)	RBM12B	Homo sapiens (Human)	1001
Q9UK61	reviewed	TASOR_HUMAN	Protein TASOR (CTCL tumor antigen se89-1) (Retinoblastoma-associated protein RAP140) (Transgene activation suppressor protein)	TASOR C3orf63 FAM208A KIAA1105	Homo sapiens (Human)	1670
Q14004	reviewed	CDK13_HUMAN	Cyclin-dependent kinase 13 (EC 2.7.11.22) (EC 2.7.11.23) (CDC2-related protein kinase 5) (Cell division cycle 2-like protein kinase 5) (Cell division protein kinase 13) (hCDK13) (Cholinesterase-related cell division controller)	CDK13 CDC2L CDC2L5 CHED KIAA1791	Homo sapiens (Human)	1512
Q9NYV4	reviewed	CDK12_HUMAN	Cyclin-dependent kinase 12 (EC 2.7.11.22) (EC 2.7.11.23) (Cdc2-related kinase, arginine/serine-rich) (CrkRS) (Cell division cycle 2-related protein kinase 7) (CDC2-related protein kinase 7) (Cell division protein kinase 12) (hCDK12)	CDK12 CRK7 CRKRS KIAA0904	Homo sapiens (Human)	1490
Q15311	reviewed	RBP1_HUMAN	RalA-binding protein 1 (RalBP1) (76 kDa Ral-interacting protein) (Dinitrophenyl S-glutathione ATPase) (DNP-SG ATPase) (EC 7.6.2.2, EC 7.6.2.3) (Ral-interacting protein 1)	RALBP1 RLIP RLIP1 RLIP76	Homo sapiens (Human)	655
Q13613	reviewed	MTMR1_HUMAN	Myotubularin-related protein 1 (Phosphatidylinositol-3,5-bisphosphate 3-phosphatase) (EC 3.1.3.95) (Phosphatidylinositol-3-phosphate phosphatase) (EC 3.1.3.64)	MTMR1	Homo sapiens (Human)	665
Q12789	reviewed	TF3C1_HUMAN	General transcription factor 3C polypeptide 1 (TF3C-alpha) (TFIIIC box B-binding subunit) (Transcription factor IIIC 220 kDa subunit) (TFIIIC 220 kDa subunit) (TFIIIC220) (Transcription factor IIIC subunit alpha)	GTF3C1	Homo sapiens (Human)	2109

Entry	Reviewed	Entry Name	Protein names	Gene Names	Organism	Length
P30825	reviewed	CTR1_HUMAN	High affinity cationic amino acid transporter 1 (CAT-1) (CAT1) (Ecotropic retroviral leukemia receptor homolog) (Ecotropic retrovirus receptor homolog) (Solute carrier family 7 member 1) (System Y+ basic amino acid transporter)	SLC7A1 ATRC1 ERR REC1L	Homo sapiens (Human)	629
Q16560	reviewed	U1SBP_HUMAN	U11/U12 small nuclear ribonucleoprotein 35 kDa protein (U11/U12 snRNP 35 kDa protein) (U11/U12-35K) (Protein HM-1) (U1 snRNP-binding protein homolog)	SNRNP35 HM1 U1SNRNPBP	Homo sapiens (Human)	246
P04259	reviewed	K2C6B_HUMAN	Keratin, type II cytoskeletal 6B (Cytokeratin-6B) (CK-6B) (Keratin-6B) (K6B) (Type-II keratin Kb10)	KRT6B K6B KRTL1	Homo sapiens (Human)	564
P13647	reviewed	K2C5_HUMAN	Keratin, type II cytoskeletal 5 (58 kDa cytokeratin) (Cytokeratin-5) (CK-5) (Keratin-5) (K5) (Type-II keratin Kb5)	KRT5	Homo sapiens (Human)	590
Q14807	reviewed	KIF22_HUMAN	Kinesin-like protein KIF22 (Kinesin-like DNA-binding protein) (Kinesin-like protein 4)	KIF22 KID KNSL4	Homo sapiens (Human)	665
P82673	reviewed	RT35_HUMAN	28S ribosomal protein S35, mitochondrial (MRP-S35) (S35mt) (28S ribosomal protein S28, mitochondrial) (MRP-S28) (S28mt) (Mitochondrial small ribosomal subunit protein mS35)	MRPS35 MRPS28 HDCMD11P MDS023 PSEC0213	Homo sapiens (Human)	323
Q9Y2D8	reviewed	ADIP_HUMAN	Afadin- and alpha-actinin-binding protein (ADIP) (Afadin DIL domain-interacting protein) (SSX2-interacting protein)	SSX2IP KIAA0923	Homo sapiens (Human)	614
Q03701	reviewed	CEBPZ_HUMAN	CCAAT/enhancer-binding protein zeta (CCAAT-box-binding transcription factor) (CBF) (CCAAT-binding factor)	CEBPZ CBF2	Homo sapiens (Human)	1054
Q12797	reviewed	ASPH_HUMAN	Aspartyl/asparaginyl beta-hydroxylase (EC 1.14.11.16) (Aspartate beta-hydroxylase) (ASP beta-hydroxylase) (Peptide-aspartate beta-dioxygenase)	ASPH BAH	Homo sapiens (Human)	758
Q9Y4F3	reviewed	MARF1_HUMAN	Meiosis regulator and mRNA stability factor 1 (Limkain-b1) (Meiosis arrest female protein	MARF1 KIAA0430 LKAP	Homo sapiens (Human)	1742
P07992	reviewed	ERCC1_HUMAN	DNA excision repair protein ERCC-1	ERCC1	Homo sapiens (Human)	297
P06702	reviewed	S10A9_HUMAN	Protein S100-A9 (Calgranulin-B) (Calprotectin L1H subunit) (Leukocyte L1 complex heavy chain) (Migration inhibitory factor-related protein 14) (MRP-14) (p14) (S100 calcium-binding protein A9)	S100A9 CAGB CFAG MRP14	Homo sapiens (Human)	114
Q7RTP6	reviewed	MICA3_HUMAN	[F-actin]-monooxygenase MICAL3 (EC 1.14.13.225) (Molecule interacting with CasL protein 3) (MICAL-3)	MICAL3 KIAA0819 KIAA1364	Homo sapiens (Human)	2002

Entry	Reviewed	Entry Name	Protein names	Gene Names	Organism	Length
Q9UGU0	reviewed	TCF20_HUMAN	Transcription factor 20 (TCF-20) (Nuclear factor SPBP) (Protein AR1) (Stromelysin-1 PDGF-responsive element-binding protein) (SPRE-binding protein)	TCF20 KIAA0292 SPBP	Homo sapiens (Human)	1960
O14979	reviewed	HNRDL_HUMAN	Heterogeneous nuclear ribonucleoprotein D-like (hnRNP D-like) (hnRNP DL) (AU-rich element RNA-binding factor) (JKT41-binding protein) (Protein laAUF1)	HNRNPDL HNRPDL JKTBP	Homo sapiens (Human)	420
P11388	reviewed	TOP2A_HUMAN	DNA topoisomerase 2-alpha (EC 5.6.2.2) (DNA topoisomerase II, alpha [illegible]	TOP2A TOP2	Homo sapiens (Human)	1531
P20592	reviewed	MX2_HUMAN	Interferon-induced GTP-binding protein Mx2 (Interferon-regulated resistance GTP-binding protein MxB) (Myxovirus resistance protein 2) (p78-related protein)	MX2	Homo sapiens (Human)	715
O00203	reviewed	AP3B1_HUMAN	AP-3 complex subunit beta-1 (Adaptor protein complex AP-3 subunit beta-1) (Adaptor-related protein complex 3 subunit beta-1) (Beta-3A-adaptin) (Clathrin assembly protein complex 3 beta-1 large chain)	AP3B1 ADTB3A	Homo sapiens (Human)	1094
Q9H2K8	reviewed	TAOK3_HUMAN	Serine/threonine-protein kinase TAO3 (EC 2.7.11.1) (Cutaneous T-cell lymphoma-associated antigen HD-CL-09) (CTCL-associated antigen HD-CL-09) (Dendritic cell-derived protein kinase) (JNK/SAPK-inhibitory kinase) (Jun kinase-inhibitory kinase) (Kinase from chicken homolog A) (hKFC-A) (Thousand and one amino acid protein 3)	TAOK3 DPK JIK KDS MAP3K18	Homo sapiens (Human)	898
Q7Z460	reviewed	CLAP1_HUMAN	CLIP-associating protein 1 (Cytoplasmic linker-associated protein 1) (Multiple asters homolog 1) (Protein Orbit homolog 1) (hOrbit1)	CLASP1 KIAA0622 MAST1	Homo sapiens (Human)	1538
O75122	reviewed	CLAP2_HUMAN	CLIP-associating protein 2 (Cytoplasmic linker-associated protein 2) (Protein Orbit homolog 2) (hOrbit2)	CLASP2 KIAA0627	Homo sapiens (Human)	1294
Q16531	reviewed	DDB1_HUMAN	DNA damage-binding protein 1 (DDB p127 subunit) (DNA damage-binding protein a) (DDBa) (Damage-specific DNA-binding protein 1) (HBV X-associated protein 1) (XAP-1) (UV-damaged DNA-binding factor) (UV-damaged DNA-binding protein 1) (UV-DDB 1) (XPE-binding factor) (XPE-BF) (Xeroderma pigmentosum group E-complementing protein) (XPCe)	DDB1 XAP1	Homo sapiens (Human)	1140

Entry	Reviewed	Entry Name	Protein names	Gene Names	Organism	Length
Q8TDI0	reviewed	CHD5_HUMAN	Chromodomain-helicase-DNA-binding protein 5 (CHD-5) (EC 3.6.4.12) (ATP-dependent helicase CHD5)	CHD5 KIAA0444	Homo sapiens (Human)	1954
P82675	reviewed	RT05_HUMAN	28S ribosomal protein S5, mitochondrial (MRP-S5) (S5mt) (Mitochondrial small ribosomal subunit protein uS5m)	MRPS5	Homo sapiens (Human)	430
P0C0S8	reviewed	H2A1_HUMAN	Histone H2A type 1 (H2A.1) (Histone H2A/ptl)	H2AC11 H2AFP HIST1H2AG; H2AC13 H2AFC HIST1H2AI; H2AC15 H2AFD HIST1H2AK; H2AC16 H2AFI HIST1H2AL; H2AC17 H2AFN HIST1H2AM	Homo sapiens (Human)	130
O75449	reviewed	KTNA1_HUMAN	Katanin p60 ATPase-containing subunit A1 (Katanin p60 subunit A1) (EC 5.6.1.1) (p60 katanin)	KATNA1	Homo sapiens (Human)	491
Q16891	reviewed	MIC60_HUMAN	MICOS complex subunit MIC60 (Cell proliferation-inducing gene 4/52 protein) (Mitochondrial inner membrane protein) (Mitofilin) (p87/89)	IMMT HMP MIC60 MINOS2 PIG4 PIG52	Homo sapiens (Human)	758
Q96RL1	reviewed	UIMC1_HUMAN	BRCA1-A complex subunit RAP80 (Receptor-associated protein 80) (Retinoid X receptor-interacting protein 110) (Ubiquitin interaction motif-containing protein 1)	UIMC1 RAP80 RXRIP110	Homo sapiens (Human)	719
Q6XE24	reviewed	RBMS3_HUMAN	RNA-binding motif, single-stranded-interacting protein 3	RBMS3	Homo sapiens (Human)	437
P06748	reviewed	NPM_HUMAN	Nucleophosmin (NPM) (Nucleolar phosphoprotein B23) (Nucleolar protein NO38) (Numatrin)	NPM1 NPM	Homo sapiens (Human)	294
Q9H8G2	reviewed	CAAP1_HUMAN	Caspase activity and apoptosis inhibitor 1 (Conserved anti-apoptotic protein) (CAAP)	CAAP1 C9orf82 CAAP	Homo sapiens (Human)	361
P07900	reviewed	HS90A_HUMAN	Heat shock protein HSP 90-alpha (EC 3.6.4.10) (Heat shock 86 kDa) (HSP 86) (HSP86) (Lipopolysaccharide-associated protein 2) (LAP-2) (LPS-associated protein 2) (Renal carcinoma antigen NY-REN-38)	HSP90AA1 HSP90A HSPC1 HSPCA	Homo sapiens (Human)	732
Q93073	reviewed	SBP2L_HUMAN	Selenocysteine insertion sequence-binding protein 2-like (SECIS-binding protein 2-like)	SECISBP2L KIAA0256	Homo sapiens (Human)	1101

Entry	Reviewed	Entry Name	Protein names	Gene Names	Organism	Length
P42167	reviewed	LAP2B_HUMAN	Lamina-associated polypeptide 2, isoforms beta/gamma (Thymopoietin, isoforms beta/gamma) (TP beta/gamma) (Thymopoietin-related peptide isoforms beta/gamma) (TPRP isoforms beta/gamma) [Cleaved into: Thymopoietin (TP) (Splenin); Thymopentin (TP5)]	TMPO LAP2	Homo sapiens (Human)	454
P42166	reviewed	LAP2A_HUMAN	Lamina-associated polypeptide 2, isoform alpha (Thymopoietin isoform alpha) (TP alpha) (Thymopoietin-related peptide isoform alpha) (TPRP isoform alpha) [Cleaved into: Thymopoietin (TP) (Splenin); Thymopentin (TP5)]	TMPO LAP2	Homo sapiens (Human)	694
Q9NZI7	reviewed	UBIP1_HUMAN	Upstream-binding protein 1 (Transcription factor LBP-1)	UBP1 LBP1	Homo sapiens (Human)	540
Q5QNW6	reviewed	H2B2F_HUMAN	Histone H2B type 2-F (H2B-clustered histone 18)	H2BC18 HIST2H2BF	Homo sapiens (Human)	126
P31943	reviewed	HNRH1_HUMAN	Heterogeneous nuclear ribonucleoprotein H (hnRNP H) [Cleaved into: Heterogeneous nuclear ribonucleoprotein H, N-terminally processed]	HNRNPH1 HNRPH HNRPH1	Homo sapiens (Human)	449
P52597	reviewed	HNRPF_HUMAN	Heterogeneous nuclear ribonucleoprotein F (hnRNP F) (Nucleolin-like protein mcs94-1) [Cleaved into: Heterogeneous nuclear ribonucleoprotein F, N-[illegible]	HNRNPF HNRPF	Homo sapiens (Human)	415
P52815	reviewed	RM12_HUMAN	39S ribosomal protein L12, mitochondrial (L12mt) (MRP-L12) (5c5-2) (Mitochondrial large ribosomal subunit protein bL12m)	MRPL12 MRPL7 RPML12	Homo sapiens (Human)	198
Q14644	reviewed	RASA3_HUMAN	Ras GTPase-activating protein 3 (GAP1(IP4BP)) (Ins P4-binding protein)	RASA3	Homo sapiens (Human)	834
Q14257	reviewed	RCN2_HUMAN	Reticulocalbin-2 (Calcium-binding protein ERC-55) (E6-binding protein) (E6BP)	RCN2 ERC55	Homo sapiens (Human)	317
Q75N03	reviewed	HAKAI_HUMAN	E3 ubiquitin-protein ligase Hakai (EC 2.3.2.27) (Casitas B-lineage lymphoma-transforming sequence-like protein 1) (c-Cbl-like protein 1) (RING finger protein 188) (RING-type E3 ubiquitin transferase Hakai)	CBLL1 HAKAI RNF188	Homo sapiens (Human)	491
Q14232	reviewed	EI2BA_HUMAN	Translation initiation factor eIF-2B subunit alpha (eIF-2B GDP-GTP exchange factor subunit alpha)	EIF2B1 EIF2BA	Homo sapiens (Human)	305
Q9ULJ7	reviewed	ANR50_HUMAN	Ankyrin repeat domain-containing protein 50	ANKRD50 KIAA1223	Homo sapiens (Human)	1429
P56652	reviewed	ITIH3_BOVIN	Inter-alpha-trypsin inhibitor heavy chain H3 (ITI heavy chain H3) (ITI-HC3) (Inter-alpha-inhibitor heavy chain 3)	ITIH3	Bos taurus (Bovine)	891

Entry	Reviewed	Entry Name	Protein names	Gene Names	Organism	Length
Q76L83	reviewed	ASXL2_HUMAN	Putative Polycomb group protein ASXL2 (Additional sex combs-like protein 2)	ASXL2 ASXH2 KIAA1685	Homo sapiens (Human)	1435
O75817	reviewed	POP7_HUMAN	Ribonuclease P protein subunit p20 (RNaseP protein p20) (Ribonucleases P/MRP protein subunit POP7 homolog) (hPOP7)	POP7 RPP20	Homo sapiens (Human)	140
Q9UBB5	reviewed	MBD2_HUMAN	Methyl-CpG-binding domain protein 2 (Demethylase) (DMTase) (Methyl-CpG-binding protein MBD2)	MBD2	Homo sapiens (Human)	411
O14641	reviewed	DVL2_HUMAN	Segment polarity protein dishevelled homolog DVL-2 (Dishevelled-2) (DSH homolog 2)	DVL2	Homo sapiens (Human)	736
Q9NRG0	reviewed	CHRC1_HUMAN	Chromatin accessibility complex protein 1 (CHRAC-1) (Chromatin accessibility complex 15 kDa protein) (CHRAC-15) (HuCHRAC15) (DNA polymerase epsilon subunit p15)	CHRAC1 CHRAC15	Homo sapiens (Human)	131
Q8WUZ0	reviewed	BCL7C_HUMAN	B-cell CLL/lymphoma 7 protein family member C	BCL7C	Homo sapiens (Human)	217
Q14839	reviewed	CHD4_HUMAN	Chromodomain-helicase-DNA-binding protein 4 (CHD-4) (EC 3.6.4.12) (ATP-dependent helicase CHD4) (Mi-2 autoantigen 218 kDa protein) (Mi2-beta)	CHD4	Homo sapiens (Human)	1912
Q9Y2X0	reviewed	MED16_HUMAN	Mediator of RNA polymerase II transcription subunit 16 (Mediator complex subunit 16) (Thyroid hormone receptor-associated protein 5) (Thyroid hormone receptor-associated protein complex 95 kDa component) (Trap95) (Vitamin D3 receptor-interacting protein complex 92 kDa component) (DRIP92)	MED16 DRIP92 THRAP5	Homo sapiens (Human)	877
O15198	reviewed	SMAD9_HUMAN	Mothers against decapentaplegic homolog 9 (MAD homolog 9) (Mothers against DPP homolog 9) (Madh6) (SMAD family member 9) (SMAD 9) (Smad9)	SMAD9 MADH6 MADH9 SMAD8	Homo sapiens (Human)	467
Q14671	reviewed	PUM1_HUMAN	Pumilio homolog 1 (HsPUM) (Pumilio-1)	PUM1 KIAA0099 PUMH1	Homo sapiens (Human)	1186
O75190	reviewed	DNJB6_HUMAN	DnaJ homolog subfamily B member 6 (HHDJ1) (Heat shock protein J2) (HSJ-2) (MRJ) (MSJ-1)	DNAJB6 HSJ2 MRJ MSJ1	Homo sapiens (Human)	326
Q13888	reviewed	TF2H2_HUMAN	General transcription factor IIH subunit 2 (Basic transcription factor 2 44 kDa subunit) (BTF2 p44) (General transcription factor IIH polypeptide 2) (TFIIH basal transcription factor complex p44 subunit)	GTF2H2 BTF2P44	Homo sapiens (Human)	395

Entry	Reviewed	Entry Name	Protein names	Gene Names	Organism	Length
O15169	reviewed	AXIN1_HUMAN	Axin-1 (Axis inhibition protein 1) (hAxin)	AXIN1 AXIN	Homo sapiens (Human)	862
Q9UIF7	reviewed	MUTYH_HUMAN	Adenine DNA glycosylase (EC 3.2.2.31) (MutY homolog) (hMYH)	MUTYH MYH	Homo sapiens (Human)	546
Q8WXF1	reviewed	PSPC1_HUMAN	Paraspeckle component 1 (Paraspeckle protein 1)	PSPC1 PSP1	Homo sapiens (Human)	523
Q9BU19	reviewed	ZN692_HUMAN	Zinc finger protein 692 (AICAR responsive element binding protein)	ZNF692 AREBP ZFP692	Homo sapiens (Human)	519
Q149N8	reviewed	SHPRH_HUMAN	E3 ubiquitin-protein ligase SHPRH (EC 2.3.2.27) (EC 3.6.4.-) (RING-type E3 ubiquitin transferase SHPRH) (SNF2, histone-linker, PHD and RING finger domain-containing helicase)	SHPRH KIAA2023	Homo sapiens (Human)	1683
Q8N3X1	reviewed	FNBP4_HUMAN	Formin-binding protein 4 (Formin-binding protein 30)	FNBP4 FBP30 KIAA1014	Homo sapiens (Human)	1017
Q9NQS7	reviewed	INCE_HUMAN	Inner centromere protein	INCENP	Homo sapiens (Human)	918
O75152	reviewed	ZC11A_HUMAN	Zinc finger CCCH domain-containing protein 11A	ZC3H11A KIAA0663 ZC3HDC11A	Homo sapiens (Human)	810
O60762	reviewed	DPM1_HUMAN	Dolichol-phosphate mannosyltransferase subunit 1 (EC 2.4.1.83) (Dolichol-phosphate mannose synthase subunit 1) (DPM synthase subunit 1) (Dolichyl-phosphate beta-D-mannosyltransferase subunit 1) (Mannose-P-dolichol synthase subunit 1) (MPD synthase subunit 1)	DPM1	Homo sapiens (Human)	260
P50613	reviewed	CDK7_HUMAN	Cyclin-dependent kinase 7 (EC 2.7.11.22) (EC 2.7.11.23) (39 kDa protein kinase) (p39 Mo15) (CDK-activating kinase 1) (Cell division protein kinase 7) (Serine/threonine-protein kinase 1) (TFIIH basal transcription factor complex kinase subunit)	CDK7 CAK CAK1 CDKN7 MO15 STK1	Homo sapiens (Human)	346
Q86UT8	reviewed	CATAC_HUMAN	Centrosomal AT-AC splicing factor (Coiled-coil domain-containing protein 84)	CENATAC CCDC84 DLNB14	Homo sapiens (Human)	332
Q04721	reviewed	NOTC2_HUMAN	Neurogenic locus notch homolog protein 2 (Notch 2) (hN2) [Cleaved into: Notch 2 extracellular truncation (N2ECD); Notch 2 intracellular domain (N2ICD)]	NOTCH2	Homo sapiens (Human)	2471
Q8N1G4	reviewed	LRC47_HUMAN	Leucine-rich repeat-containing protein 47	LRRC47 KIAA1185	Homo sapiens (Human)	583
Q96QB1	reviewed	RHG07_HUMAN	Rho GTPase-activating protein 7 (Deleted in liver cancer 1 protein) (DLC-1) (HP protein) (Rho-type GTPase-activating protein 7) (START domain-containing protein 12) (StARD12) (StAR-related lipid transfer protein 12)	DLC1 ARHGAP7 KIAA1723 STARD12	Homo sapiens (Human)	1528

Entry	Reviewed	Entry Name	Protein names	Gene Names	Organism	Length
Q32P28	reviewed	P3H1_HUMAN	Prolyl 3-hydroxylase 1 (EC 1.14.11.7) (Growth suppressor 1) (Leucine- and proline-enriched proteoglycan 1) [illegible]	P3H1 GROS1 LEPRE1 PSEC0109	Homo sapiens (Human)	736
Q9Y5T5	reviewed	UBP16_HUMAN	Ubiquitin carboxyl-terminal hydrolase 16 (EC 3.4.19.12) (Deubiquitinating enzyme 16) (Ubiquitin thioesterase 16) (Ubiquitin-processing protease UBP-M) (Ubiquitin-specific-processing protease 16)	USP16 MSTP039	Homo sapiens (Human)	823
Q9NUD5	reviewed	ZCHC3_HUMAN	Zinc finger CCHC domain-containing protein 3	ZCCHC3 C20orf99	Homo sapiens (Human)	403
P06493	reviewed	CDK1_HUMAN	Cyclin-dependent kinase 1 (CDK1) (EC 2.7.11.22) (EC 2.7.11.23) (Cell division control protein 2 homolog) (Cell division protein kinase 1) (p34 protein kinase)	CDK1 CDC2 CDC28A CDKN1 P34CDC2	Homo sapiens (Human)	297
Q96A72	reviewed	MGN2_HUMAN	Protein mago nashi homolog 2	MAGOHB MAGOH2	Homo sapiens (Human)	148
Q92759	reviewed	TF2H4_HUMAN	General transcription factor IIH subunit 4 (Basic transcription factor 2 52 kDa subunit) (BTF2 p52) (General transcription factor IIH polypeptide 4) (TFIIH basal transcription factor complex p52 subunit)	GTF2H4	Homo sapiens (Human)	462
Q96NE9	reviewed	FRMD6_HUMAN	FERM domain-containing protein 6 (Willin)	FRMD6 C14orf31	Homo sapiens (Human)	622
Q86W92	reviewed	LIPB1_HUMAN	Liprin-beta-1 (Protein tyrosine phosphatase receptor type f polypeptide-interacting protein-binding protein 1) (PTPRF-interacting protein-binding protein 1) (hSGT2)	PPFIBP1 KIAA1230	Homo sapiens (Human)	1011
O75534	reviewed	CSDE1_HUMAN	Cold shock domain-containing protein E1 (N-ras upstream gene protein) (Protein UNR)	CSDE1 D1S155E KIAA0885 NRU UNR	Homo sapiens (Human)	798
P29084	reviewed	T2EB_HUMAN	Transcription initiation factor IIE subunit beta (TFIIE-beta) (General transcription factor IIE subunit 2)	GTF2E2 TF2E2	Homo sapiens (Human)	291
O00567	reviewed	NOP56_HUMAN	Nucleolar protein 56 (Nucleolar protein 5A)	NOP56 NOL5A	Homo sapiens (Human)	594
Q9UEY8	reviewed	ADDG_HUMAN	Gamma-adducin (Adducin-like protein 70)	ADD3 ADDL	Homo sapiens (Human)	706
Q8IYB7	reviewed	DI3L2_HUMAN	DIS3-like exonuclease 2 (hDIS3L2) (EC 3.1.13.-)	DIS3L2 FAM6A	Homo sapiens (Human)	885
Q8TAP9	reviewed	MPLKI_HUMAN	M-phase-specific PLK1-interacting protein (TTD non-photosensitive 1 protein)	MPLKIP C7orf11 TTDN1	Homo sapiens (Human)	179
Q6P1L5	reviewed	F117B_HUMAN	Protein FAM117B (Amyotrophic lateral sclerosis 2 chromosomal region candidate gene 13 protein)	FAM117B ALS2CR13	Homo sapiens (Human)	589
Q5D862	reviewed	FILA2_HUMAN	Filaggrin-2 (FLG-2) (Intermediate filament-associated and psoriasis-susceptibility protein) (Ifapsoriasin)	FLG2 IFPS	Homo sapiens (Human)	2391

Entry	Reviewed	Entry Name	Protein names	Gene Names	Organism	Length
P46782	reviewed	RS5_HUMAN	40S ribosomal protein S5 (Small ribosomal subunit protein uS7) [Cleaved into: 40S ribosomal protein S5, N-terminally processed]	RPS5	Homo sapiens (Human)	204
Q9Y5Q9	reviewed	TF3C3_HUMAN	General transcription factor 3C polypeptide 3 (Transcription factor IIIC 102 kDa subunit) (TFIIIC 102 kDa subunit) (TFIIIC102) (Transcription factor IIIC subunit gamma) (TF3C-gamma)	GTF3C3	Homo sapiens (Human)	886
P12980	reviewed	LYL1_HUMAN	Protein lyl-1 (Class A basic helix-loop-helix protein 18) (bHLHa18) (Lymphoblastic leukemia-derived sequence 1)	LYL1 BHLHA18	Homo sapiens (Human)	280
O95758	reviewed	PTBP3_HUMAN	Polypyrimidine tract-binding protein 3 (Regulator of differentiation 1) (Rod1)	PTBP3 ROD1	Homo sapiens (Human)	552
Q8NEY1	reviewed	NAV1_HUMAN	Neuron navigator 1 (Pore membrane and/or filament-interacting-like protein 3) (Steerin-1) (Unc-53 homolog 1) (unc53H1)	NAV1 KIAA1151 KIAA1213 POMFIL3 STEERIN1	Homo sapiens (Human)	1877
P41221	reviewed	WNT5A_HUMAN	Protein Wnt-5a	WNT5A	Homo sapiens (Human)	380
O14640	reviewed	DVL1_HUMAN	Segment polarity protein dishevelled homolog DVL-1 (Dishevelled-1) (DSH homolog 1)	DVL1	Homo sapiens (Human)	695
Q9H2D6	reviewed	TARA_HUMAN	TRIO and F-actin-binding protein (Protein Tara) (Trio-associated repeat on actin)	TRIOBP KIAA1662 TARA HRIHFB2122	Homo sapiens (Human)	2365
Q6P2S7	reviewed	TTC41_HUMAN	Putative tetratricopeptide repeat protein 41 (TPR repeat protein 41) (Grp94-neighboring nucleotidase)	TTC41P GNN	Homo sapiens (Human)	1318
O15027	reviewed	SC16A_HUMAN	Protein transport protein Sec16A (SEC16 homolog A) (p250)	SEC16A KIAA0310 SEC16 SEC16L	Homo sapiens (Human)	2357
Q9BYN8	reviewed	RT26_HUMAN	28S ribosomal protein S26, mitochondrial (MRP-S26) (S26mt) (28S ribosomal protein S13, mitochondrial) (MRP-S13) (S13mt) (Mitochondrial small ribosomal subunit protein mS26)	MRPS26 C20orf193 RPMS13	Homo sapiens (Human)	205
O94804	reviewed	STK10_HUMAN	Serine/threonine-protein kinase 10 (EC 2.7.11.1) (Lymphocyte-oriented kinase)	STK10 LOK	Homo sapiens (Human)	968
Q13347	reviewed	EIF3I_HUMAN	Eukaryotic translation initiation factor 3 subunit I (eIF3i) (Eukaryotic translation initiation factor 3 subunit 2) (TGF-beta receptor-interacting protein 1) (TRIP-1) (eIF-3-beta) (eIF3 p36)	EIF3I EIF3S2 TRIP1	Homo sapiens (Human)	325
Q9ULG6	reviewed	CCPG1_HUMAN	Cell cycle progression protein 1 (Cell cycle progression restoration protein 8)	CCPG1 CCP8 CPR8 KIAA1254	Homo sapiens (Human)	757

Entry	Reviewed	Entry Name	Protein names	Gene Names	Organism	Length
P25398	reviewed	RS12_HUMAN	40S ribosomal protein S12 (Small ribosomal subunit protein eS12)	RPS12	Homo sapiens (Human)	132
P49023	reviewed	PAXI_HUMAN	Paxillin	PXN	Homo sapiens (Human)	591
Q96AE4	reviewed	FUBP1_HUMAN	Far upstream element-binding protein 1 (FBP) (FUSE-binding protein 1) (DNA helicase V) (hDH V)	FUBP1	Homo sapiens (Human)	644
Q6P158	reviewed	DHX57_HUMAN	Putative ATP-dependent RNA helicase DHX57 (EC 3.6.4.13) (DEAH box protein 57)	DHX57	Homo sapiens (Human)	1386
Q9H2U1	reviewed	DHX36_HUMAN	ATP-dependent DNA/RNA helicase DHX36 (EC 3.6.4.12) (EC 3.6.4.13) (DEAD/H box polypeptide 36) (DEAH-box protein 36) (G4-resolvase-1) (G4R1) (MLE-like protein 1) (RNA helicase associated with AU-rich element protein)	DHX36 DDX36 KIAA1488 MLEL1 RHAU	Homo sapiens (Human)	1008
Q9NZI8	reviewed	IF2B1_HUMAN	Insulin-like growth factor 2 mRNA-binding protein 1 (IGF2 mRNA-binding protein 1) (IMP-1) (IMP1) (Coding region determinant-binding protein) (CRD-BP) (IGF-II mRNA-binding protein 1) (VICKZ family member 1) (Zipcode-binding protein 1) (ZBP-1)	IGF2BP1 CRDBP VICKZ1 ZBP1	Homo sapiens (Human)	577
O00425	reviewed	IF2B3_HUMAN	Insulin-like growth factor 2 mRNA-binding protein 3 (IGF2 mRNA-binding protein 3) (IMP-3) (IGF-II mRNA-binding protein 3) (KH domain-containing protein overexpressed in cancer) (hKOC) (VICKZ family member 3)	IGF2BP3 IMP3 KOC1 VICKZ3	Homo sapiens (Human)	579
P0C0S5	reviewed	H2AZ_HUMAN	Histone H2A.Z (H2A/z)	H2AZ1 H2AFZ H2AZ	Homo sapiens (Human)	128
P18146	reviewed	EGR1_HUMAN	Early growth response protein 1 (EGR-1) (AT225) (Nerve growth factor-induced protein A) (NGFI-A) (Transcription factor ETR103) (Transcription factor Zif268) (Zinc finger protein 225) (Zinc finger protein Krox-24)	EGR1 KROX24 ZNF225	Homo sapiens (Human)	543
Q7L7X3	reviewed	TAOK1_HUMAN	Serine/threonine-protein kinase TAO1 (EC 2.7.11.1) (Kinase from chicken homolog B) (hKFC-B) (MARK Kinase) (MARKK) (Prostate-derived sterile 20-like kinase 2) (PSK-2) (PSK2) (Prostate-derived STE20-like kinase 2) (Thousand and one amino acid protein kinase 1) (TAOK1) (hTAOK1)	TAOK1 KIAA1361 MAP3K16 MARKK	Homo sapiens (Human)	1001

Entry	Reviewed	Entry Name	Protein names	Gene Names	Organism	Length
Q9UL54	reviewed	TAOK2_HUMAN	Serine/threonine-protein kinase TAO2 (EC 2.7.11.1) (Kinase from chicken homolog C) (hKFC-C) (Prostate-derived sterile 20-like kinase 1) (PSK-1) (PSK1) (Prostate-derived STE20-like kinase 1) (Thousand and one amino acid protein kinase 2)	TAOK2 KIAA0881 MAP3K17 PSK PSK1 UNQ2971/PRO7431	Homo sapiens (Human)	1235
Q5T2D3	reviewed	OTUD3_HUMAN	OTU domain-containing protein 3 (EC 3.4.19.12)	OTUD3 KIAA0459	Homo sapiens (Human)	398
Q9P2N5	reviewed	RBM27_HUMAN	RNA-binding protein 27 (RNA-binding motif protein 27)	RBM27 KIAA1311	Homo sapiens (Human)	1060
Q6IE81	reviewed	JADE1_HUMAN	Protein Jade-1 (Jade family PHD finger protein 1) (PHD finger protein 17)	JADE1 KIAA1807 PHF17	Homo sapiens (Human)	842
Q9P013	reviewed	CWC15_HUMAN	Spliceosome-associated protein CWC15 homolog	CWC15 C11orf5 AD-002 HSPC148	Homo sapiens (Human)	229
P04264	reviewed	K2C1_HUMAN	Keratin, type II cytoskeletal 1 (67 kDa cytokeratin) (Cytokeratin-1) (CK-1) (Hair alpha protein) (Keratin-1) (K1) (Type-II keratin Kb1)	KRT1 KRTA	Homo sapiens (Human)	644
P05787	reviewed	K2C8_HUMAN	Keratin, type II cytoskeletal 8 (Cytokeratin-8) (CK-8) (Keratin-8) (K8) (Type-II keratin Kb8)	KRT8 CYK8	Homo sapiens (Human)	483
P31946	reviewed	1433B_HUMAN	14-3-3 protein beta/alpha (Protein 1054) (Protein kinase C inhibitor protein 1) (KCIP-1) [Cleaved into: 14-3-3 protein beta/alpha, N-terminally processed]	YWHAB	Homo sapiens (Human)	246
P31689	reviewed	DNJA1_HUMAN	DnaJ homolog subfamily A member 1 (DnaJ protein homolog 2) (HSDJ) (Heat shock 40 kDa protein 4) (Heat shock protein J2) (HSJ-2) (Human DnaJ protein 2) (hDj-2)	DNAJA1 DNAJ2 HDJ2 HSJ2 HSPF4	Homo sapiens (Human)	397
Q96MX3	reviewed	ZNF48_HUMAN	Zinc finger protein 48 (Zinc finger protein 553)	ZNF48 ZNF553	Homo sapiens (Human)	618
Q92888	reviewed	ARHG1_HUMAN	Rho guanine nucleotide exchange factor 1 (115 kDa guanine nucleotide exchange factor) (p115-RhoGEF) (p115RhoGEF) (Sub1.5)	ARHGEF1	Homo sapiens (Human)	912
Q86YZ3	reviewed	HORN_HUMAN	Hornerin	HRNR S100A18	Homo sapiens (Human)	2850
A6NEE1	reviewed	PLHD1_HUMAN	Pleckstrin homology domain-containing family D member 1 (PH domain-containing family D member 1)	PLEKHD1	Homo sapiens (Human)	506
Q15651	reviewed	HMGN3_HUMAN	High mobility group nucleosome-binding domain-containing protein 3 (Thyroid receptor-interacting protein 7) (TR-interacting protein 7) (TRIP-7)	HMGN3 TRIP7 PNAS-24	Homo sapiens (Human)	99
Q07020	reviewed	RL18_HUMAN	60S ribosomal protein L18 (Large ribosomal subunit protein eL18)	RPL18	Homo sapiens (Human)	188

Entry	Reviewed	Entry Name	Protein names	Gene Names	Organism	Length
Q9C0A6	reviewed	SETD5_HUMAN	Histone-lysine N-methyltransferase SETD5 (EC 2.1.1.359) (EC 2.1.1.367) (SET domain-containing protein 5)	SETD5 KIAA1757	Homo sapiens (Human)	1442
Q15345	reviewed	LRC41_HUMAN	Leucine-rich repeat-containing protein 41 (Protein Muf1)	LRRC41 MUF1 PP7759	Homo sapiens (Human)	812
Q86V48	reviewed	LUZP1_HUMAN	Leucine zipper protein 1	LUZP1	Homo sapiens (Human)	1076
O60783	reviewed	RT14_HUMAN	28S ribosomal protein S14, mitochondrial (MRP-S14) (S14mt) (Mitochondrial small ribosomal subunit protein uS14m)	MRPS14	Homo sapiens (Human)	128
Q9Y2D9	reviewed	ZN652_HUMAN	Zinc finger protein 652	ZNF652 KIAA0924	Homo sapiens (Human)	606
Q96I24	reviewed	FUBP3_HUMAN	Far upstream element-binding protein 3 (FUSE-binding protein 3)	FUBP3 FBP3	Homo sapiens (Human)	572
Q9BRX2	reviewed	PELO_HUMAN	Protein pelota homolog (EC 3.1.-.-)	PELO CGI-17	Homo sapiens (Human)	385
Q99985	reviewed	SEM3C_HUMAN	Semaphorin-3C (Semaphorin-E) (Sema E)	SEMA3C SEMAE	Homo sapiens (Human)	751
Q5JSZ5	reviewed	PRC2B_HUMAN	Protein PRRC2B (HLA-B-associated transcript 2-like 1) (Proline-rich coiled-coil protein 2B)	PRRC2B BAT2L BAT2L1 KIAA0515	Homo sapiens (Human)	2229
Q9H1J1	reviewed	REN3A_HUMAN	Regulator of nonsense transcripts 3A (Nonsense mRNA reducing factor 3A) (Up-frameshift suppressor 3 homolog A) (hUpf3)	UPF3A RENT3A UPF3	Homo sapiens (Human)	476
Q8TEU7	reviewed	RPGF6_HUMAN	Rap guanine nucleotide exchange factor 6 (PDZ domain-containing guanine nucleotide exchange factor 2) (PDZ-GEF2) (RA-GEF-2)	RAPGEF6 PDZGEF2	Homo sapiens (Human)	1601
Q96BK5	reviewed	PINX1_HUMAN	PIN2/TERF1-interacting telomerase inhibitor 1 (Liver-related putative tumor suppressor) (Pin2-interacting protein X1) (Protein 67-11-3) (TRF1-interacting protein 1)	PINX1 LPTL LPTS	Homo sapiens (Human)	328
Q5VV52	reviewed	ZN691_HUMAN	Zinc finger protein 691	ZNF691	Homo sapiens (Human)	315
Q00056	reviewed	HXA4_HUMAN	Homeobox protein Hox-A4 (Homeobox protein Hox-1.4) (Homeobox protein Hox-1D)	HOXA4 HOX1D	Homo sapiens (Human)	320
Q8WTV1	reviewed	THAP3_HUMAN	THAP domain-containing protein 3	THAP3	Homo sapiens (Human)	239
Q9UK33	reviewed	ZN580_HUMAN	Zinc finger protein 580 (LDL-induced EC protein)	ZNF580	Homo sapiens (Human)	172
Q8N4C8	reviewed	MINK1_HUMAN	Misshapen-like kinase 1 (EC 2.7.11.1) (GCK family kinase MiNK) (MAPK/ERK kinase kinase kinase 6) (MEK kinase kinase 6) (MEKKK 6) (Misshapen/NIK-related kinase) (Mitogen-activated protein kinase kinase kinase kinase 6)	MINK1 B55 MAP4K6 MINK YSK2 ZC3	Homo sapiens (Human)	1332

Entry	Reviewed	Entry Name	Protein names	Gene Names	Organism	Length
Q07352	reviewed	TISB_HUMAN	mRNA decay activator protein ZFP36L1 (Butyrate response factor 1) (EGF-response factor 1) (ERF-1) (TPA-induced sequence 11b) (Zinc finger protein 36, C3H1 type-like 1) (ZFP36-like 1)	ZFP36L1 BERG36 BRF1 ERF1 RNF162B TIS11B	Homo sapiens (Human)	338
P02533	reviewed	K1C14_HUMAN	Keratin, type I cytoskeletal 14 (Cytokeratin-14) (CK-14) (Keratin-14) (K14)	KRT14	Homo sapiens (Human)	472
P13645	reviewed	K1C10_HUMAN	Keratin, type I cytoskeletal 10 (Cytokeratin-10) (CK-10) (Keratin-10) (K10)	KRT10 KPP	Homo sapiens (Human)	584
P08779	reviewed	K1C16_HUMAN	Keratin, type I cytoskeletal 16 (Cytokeratin-16) (CK-16) (Keratin-16) (K16)	KRT16 KRT16A	Homo sapiens (Human)	473
Q96A65	reviewed	EXOC4_HUMAN	Exocyst complex component 4 (Exocyst complex component Sec8)	EXOC4 KIAA1699 SEC8 SEC8L1	Homo sapiens (Human)	974
Q6UB99	reviewed	ANR11_HUMAN	Ankyrin repeat domain-containing protein 11 (Ankyrin repeat-containing cofactor 1)	ANKRD11 ANCO1	Homo sapiens (Human)	2663
Q13936	reviewed	CAC1C_HUMAN	Voltage-dependent L-type calcium channel subunit alpha-1C (Calcium channel, L type, alpha-1 polypeptide, isoform 1, cardiac muscle) (Voltage-gated calcium channel subunit alpha Cav1.2)	CACNA1C CACH2 CACN2 CACNL1A1 CCHL1A1	Homo sapiens (Human)	2221
Q14011	reviewed	CIRBP_HUMAN	Cold-inducible RNA-binding protein (A18 hnRNP) (Glycine-rich RNA-binding protein CIRP)	CIRBP A18HNRNP CIRP	Homo sapiens (Human)	172
Q8WVV9	reviewed	HNRLL_HUMAN	Heterogeneous nuclear ribonucleoprotein L-like (hnRNPLL) (Stromal RNA-regulating factor)	HNRNPLL HNRPLL SRRF BLOCK24	Homo sapiens (Human)	542
Q01804	reviewed	OTUD4_HUMAN	OTU domain-containing protein 4 (EC 3.4.19.12) (HIV-1-induced protein HIN-1)	OTUD4 HIN-1 KIAA1046	Homo sapiens (Human)	1114
Q9Y4W6	reviewed	AFG32_HUMAN	AFG3-like protein 2 (EC 3.4.24.-) (Paraplegin-like	AFG3L2	Homo sapiens (Human)	797
P82930	reviewed	RT34_HUMAN	28S ribosomal protein S34, mitochondrial (MRP-S34) (S34mt) (Mitochondrial small ribosomal subunit protein mS34)	MRPS34	Homo sapiens (Human)	218
Q9UPQ3	reviewed	AGAP1_HUMAN	Arf-GAP with GTPase, ANK repeat and PH domain-containing protein 1 (AGAP-1) (Centaurin-gamma-2) (Cnt-g2) (GTP-binding and GTPase-activating protein 1) (GGAP1)	AGAP1 CENTG2 KIAA1099	Homo sapiens (Human)	857
Q9H0U3	reviewed	MAGT1_HUMAN	Magnesium transporter protein 1 (MagT1) (Dolichyl-diphosphooligosaccharide--protein glycosyltransferase subunit MAGT1) (Oligosaccharyl transferase subunit MAGT1) (Implantation-associated protein) (IAP)	MAGT1 IAG2 PSEC0084 UNQ628/PRO1244	Homo sapiens (Human)	335

Entry	Reviewed	Entry Name	Protein names	Gene Names	Organism	Length
Q96HA1	reviewed	P121A_HUMAN	Nuclear envelope pore membrane protein POM 121 (Nuclear envelope pore membrane protein POM 121A) (Nucleoporin Nup121) (Pore membrane protein of 121 kDa)	POM121 KIAA0618 NUP121 POM121A	Homo sapiens (Human)	1249
Q8WY91	reviewed	THAP4_HUMAN	Peroxynitrite isomerase THAP4 (EC 5.99.-.-) (Ferric Homo sapiens nitrobindin) (Hs-Nb(III)) (THAP domain-containing protein 4)	THAP4 CGI-36 PP238	Homo sapiens (Human)	577
O95757	reviewed	HS74L_HUMAN	Heat shock 70 kDa protein 4L (Heat shock 70-related protein APG-1) (Heat-shock protein family A member 4-like protein) (HSPA4-like protein) (Osmotic stress protein 94)	HSPA4L APG1 OSP94	Homo sapiens (Human)	839
O15226	reviewed	NKRF_HUMAN	NF-kappa-B-repressing factor (NFkB-repressing factor) (NRF) (Protein ITBA4)	NKRF ITBA4 NRF	Homo sapiens (Human)	690
Q8N8E2	reviewed	ZN513_HUMAN	Zinc finger protein 513	ZNF513	Homo sapiens (Human)	541
Q96DN6	reviewed	MBD6_HUMAN	Methyl-CpG-binding domain protein 6 (Methyl-CpG-binding protein MBD6)	MBD6 KIAA1887	Homo sapiens (Human)	1003
Q01968	reviewed	OCRL_HUMAN	Inositol polyphosphate 5-phosphatase OCRL (EC 3.1.3.36) (EC 3.1.3.56) (Inositol polyphosphate 5-phosphatase OCRL-1) (OCRL-1) (Lowe oculocerebrorenal syndrome protein) (Phosphatidylinositol 3,4,5-triphosphate 5-phosphatase) (EC 3.1.3.86)	OCRL OCRL1	Homo sapiens (Human)	901
Q8N1G1	reviewed	REXO1_HUMAN	RNA exonuclease 1 homolog (EC 3.1.-.-) (Elongin-A-binding protein 1) (EloA-BP1) (Transcription elongation factor B polypeptide 3-binding protein 1)	REXO1 ELOABP1 KIAA1138 TCEB3BP1	Homo sapiens (Human)	1221
Q9NXV6	reviewed	CARF_HUMAN	CDKN2A-interacting protein (Collaborator of ARF)	CDKN2AIP CARF	Homo sapiens (Human)	580
O94907	reviewed	DKK1_HUMAN	Dickkopf-related protein 1 (Dickkopf-1) (Dkk-1) (hDkk-1) (SK)	DKK1 UNQ492/PRO1008	Homo sapiens (Human)	266
Q8TBF4	reviewed	ZCRB1_HUMAN	Zinc finger CCHC-type and RNA-binding motif-containing protein 1 (U11/U12 small nuclear ribonucleoprotein 31 kDa protein) (U11/U12 snRNP 31 kDa protein) (U11/U12-31K)	ZCRB1	Homo sapiens (Human)	217
P12270	reviewed	TPR_HUMAN	Nucleoprotein TPR (Megator) (NPC-associated intranuclear protein) (Translocated promoter region protein)	TPR	Homo sapiens (Human)	2363
Q92598	reviewed	HS105_HUMAN	Heat shock protein 105 kDa (Antigen NY-CO-25) (Heat shock 110 kDa protein)	HSPH1 HSP105 HSP110	Homo sapiens (Human)	858

Entry	Reviewed	Entry Name	Protein names	Gene Names	Organism	Length
Q8TBX8	reviewed	PI42C_HUMAN	Phosphatidylinositol 5-phosphate 4-kinase type-2 gamma (EC 2.7.1.149) (Phosphatidylinositol 5-phosphate 4-kinase type II gamma) (PI(5)P 4-kinase type II gamma) (PIP4KII-gamma)	PIP4K2C PIP5K2C	Homo sapiens (Human)	421
Q9UHB6	reviewed	LIMA1_HUMAN	LIM domain and actin-binding protein 1 (Epithelial protein lost in neoplasm)	LIMA1 EPLIN SREBP3 PP624	Homo sapiens (Human)	759
Q9UQE7	reviewed	SMC3_HUMAN	Structural maintenance of chromosomes protein 3 (SMC protein 3) (SMC-3) (Basement membrane-associated chondroitin proteoglycan) (Bamacan) (Chondroitin sulfate proteoglycan 6) (Chromosome-associated polypeptide) (hCAP)	SMC3 BAM BMH CSPG6 SMC3L1	Homo sapiens (Human)	1217
Q99418	reviewed	CYH2_HUMAN	Cytohesin-2 (ARF exchange factor) (ARF nucleotide-binding site opener) (Protein ARNO) (PH, SEC7 and coiled-coil domain-containing protein 2)	CYTH2 ARNO PSCD2 PSCD2L	Homo sapiens (Human)	400
Q9H7Z6	reviewed	KAT8_HUMAN	Histone acetyltransferase KAT8 (EC 2.3.1.48) (Lysine acetyltransferase 8) (MOZ, YBF2/SAS3, SAS2 and TIP60 protein 1) (MYST-1) (hMOF)	KAT8 MOF MYST1 PP7073	Homo sapiens (Human)	458
Q9Y388	reviewed	RBMX2_HUMAN	RNA-binding motif protein, X-linked 2	RBMX2 CGI-79	Homo sapiens (Human)	322
Q6WCQ1	reviewed	MPRIP_HUMAN	Myosin phosphatase Rho-interacting protein (M-RIP) (Rho-interacting protein 3) (RIP3) (p116Rip)	MPRIP KIAA0864 MRIP RHOIP3	Homo sapiens (Human)	1025
P04049	reviewed	RAF1_HUMAN	RAF proto-oncogene serine/threonine-protein kinase (EC 2.7.11.1) (Proto-oncogene c-RAF) (cRaf) (Raf-1)	RAF1 RAF	Homo sapiens (Human)	648
P15559	reviewed	NQO1_HUMAN	NAD(P)H dehydrogenase [quinone] 1 (EC 1.6.5.2) (Azoreductase) (DT-diaphorase) (DTD) (Menadione reductase) (NAD(P)H:quinone oxidoreductase 1) (Phylloquinone reductase) (Quinone reductase 1) (QR1)	NQO1 DIA4 NMOR1	Homo sapiens (Human)	274
Q9UPT9	reviewed	UBP22_HUMAN	Ubiquitin carboxyl-terminal hydrolase 22 (EC 3.4.19.12) (Deubiquitinating enzyme 22) (Ubiquitin thioesterase 22) (Ubiquitin-specific-processing protease 22)	USP22 KIAA1063 USP3L	Homo sapiens (Human)	525
Q96EP5	reviewed	DAZP1_HUMAN	DAZ-associated protein 1 (Deleted in azoospermia-associated protein 1)	DAZAP1	Homo sapiens (Human)	407

Entry	Reviewed	Entry Name	Protein names	Gene Names	Organism	Length
Q15599	reviewed	NHRF2_HUMAN	Na(+)/H(+) exchange regulatory cofactor NHE-RF2 (NHERF-2) (NHE3 kinase A regulatory protein E3KARP) (SRY-interacting protein 1) (SIP-1) (Sodium-hydrogen exchanger regulatory factor 2) (Solute carrier family 9 isoform A3 regulatory factor 2) (Tyrosine kinase activator protein 1) (TKA-1)	SLC9A3R2 NHERF2	Homo sapiens (Human)	337
P50416	reviewed	CPT1A_HUMAN	Carnitine O-palmitoyltransferase 1, liver isoform (CPT1-L) (EC 2.3.1.21) (Carnitine O-palmitoyltransferase I, liver isoform) (CPT I) (CPTI-L) (Carnitine palmitoyltransferase 1A)	CPT1A CPT1	Homo sapiens (Human)	773
Q9P227	reviewed	RHG23_HUMAN	Rho GTPase-activating protein 23 (Rho-type GTPase-activating protein 23)	ARHGAP23 KIAA1501	Homo sapiens (Human)	1491

Supplementary Table S5. The list of antibodies and primer sequences.

Antibodies

Name	Supplier	Cat no.
anti-GAPDH	Santa	sc-32233
anti-YAP	Santa	sc-101199
anti-TAZ	Santa	sc-518026
anti-TAOK1	Santa	sc-136094
anti-TAOK2	Santa	sc-514268
anti-phosphorylated TAOK1/2	Abcam	ab124841
anti-phosphorylated LATS1/2	Bioss	bs-7913R
anti-IL-1β	Santa	sc-32294
anti-TEAD4	Affinity	DF13283
anti-phosphorylated YAP	Affinity	AF3328
anti-phosphorylated TAZ	Affinity	AF4315
anti-phosphorylated PI3K	Cell Signaling Technology	4228S
anti-phosphorylated AKT	Cell Signaling Technology	9018S
anti-AKT	Cell Signaling Technology	4691S
Human IL-1β ELISA kit	Abclonal	RK00001

Primer Sequences

Primers	Primer sequences
hGAPDH_FP	5’-GGATATTGTTGCCATCAATGACC-3’
hGAPDH_RP	5’-AGCCTTCTCCATGGTGGTGAAGA-3’
LINC01198_FP	5’-TGAACTGAGGTACCCTCCAAAGC-3’
LINC01198_RP	5’-TGGACGATAACAATGGCAGAAAG-3’
IL1B_FP	5’-GACACATGGGATAACGAGGCTTA-3’
IL1B_RP	5’-ACACGCAGGACAGGTACAGATTC-3’
IL1B_TEAD ChIP_FP	5’-GAGGCAGGAGAATGGTGTGAAC-3’
IL1B_TEAD ChIP_RP	5’-ACAGTGCCTGGAGTGTAGGAACA-3’

siRNA Sequences

siLINC01198_01	5’-GACAGGATAACTTCAGGAA-3’
siLINC01198_01	5’-GACCTGAGCAAAGCAGTGA-3’
siIL1B_01	5’-AGAUGAAGGGAAAGAAGGUTT-3’
siIL1B_02	5'-CUUGUUGAAGACAAAUCGCTT-3’
siTEAD_01	5'-CUUGUGGAUGAAGUUGAUCAUTT-3’
siTEAD_02	5'-AGCUUGUGGAUGAAGUUGAUCTT-3’
siNC	siN0000001-1-5

gRNA Oligo	PAGE purification synthesis	
	gRNA946F	CACCGtcccggagcctaagtgccta
	gRNA946R	AAACtaggcacttaggctccgggaC
	gRNA433F	CACCgcaccacccgtgagagtgct
	gRNA433R	AAACagcactctcacgggtggtgc
	gRNA1020F	CACCggggagtgggttaatctaat
	gRNA1020R	AAACattagattaacccactcccc
	gRNA672F	CACCgcattatgtgattgctcagg
	gRNA672R	AAACcctgagcaatcacataatgc

Primer Sequences for Knockout verification

	Linc01198 F1	GCACAAGTTTGATGGCACAAGTC
	Linc01198 F2	ACCTCAAACACTGCAAAGGTTC
	Linc01198 R1	TATGTGCATTTCGGCTGCTCA
	Linc01198 R2	CTTAGGACCCACTTGGAGGACTGT
	Linc01198 R3	GACACCAATCGCAGGGGT
