# Supplementary material for: LINC01198 activates Hippo signaling to stimulate IL-1β autocrine for driving vemurafenib resistance by associating with TAOK1/2 in melanoma
Source: Cell Death Discov. 2025 Oct 27;11:486. doi: 10.1038/s41420-025-02773-6 (PMC12559216; doi:10.1038/s41420-025-02773-6)
Supplement: Supplementary file 2 — Original Data [file 41420_2025_2773_MOESM2_ESM.pdf]

Figure. 4C

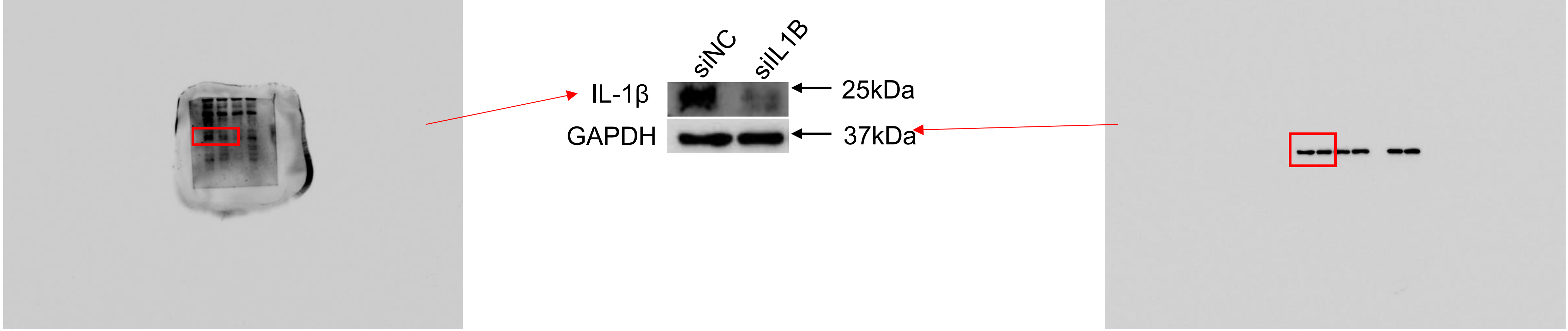

Figure. 4E

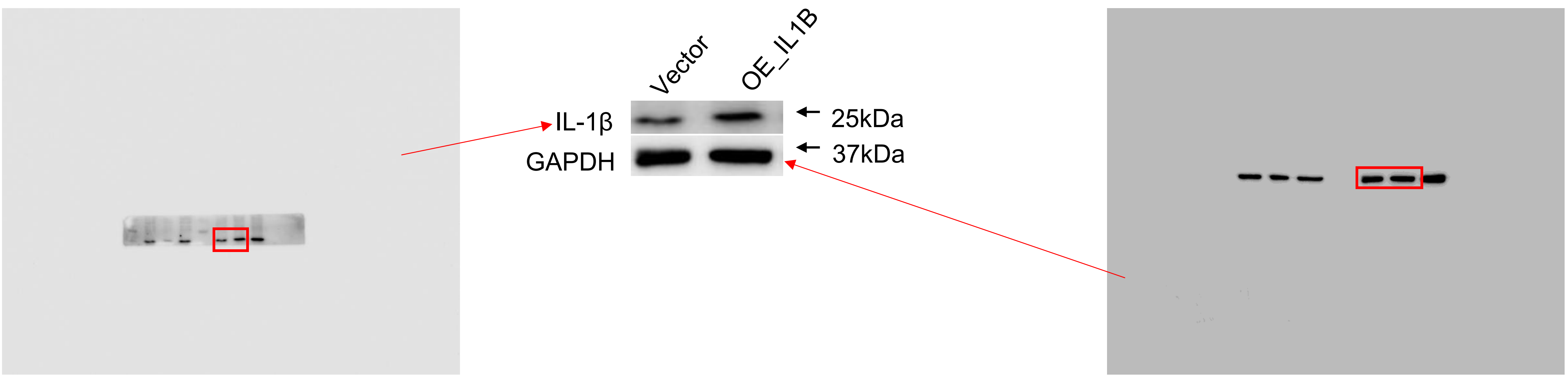

Figure. 5E

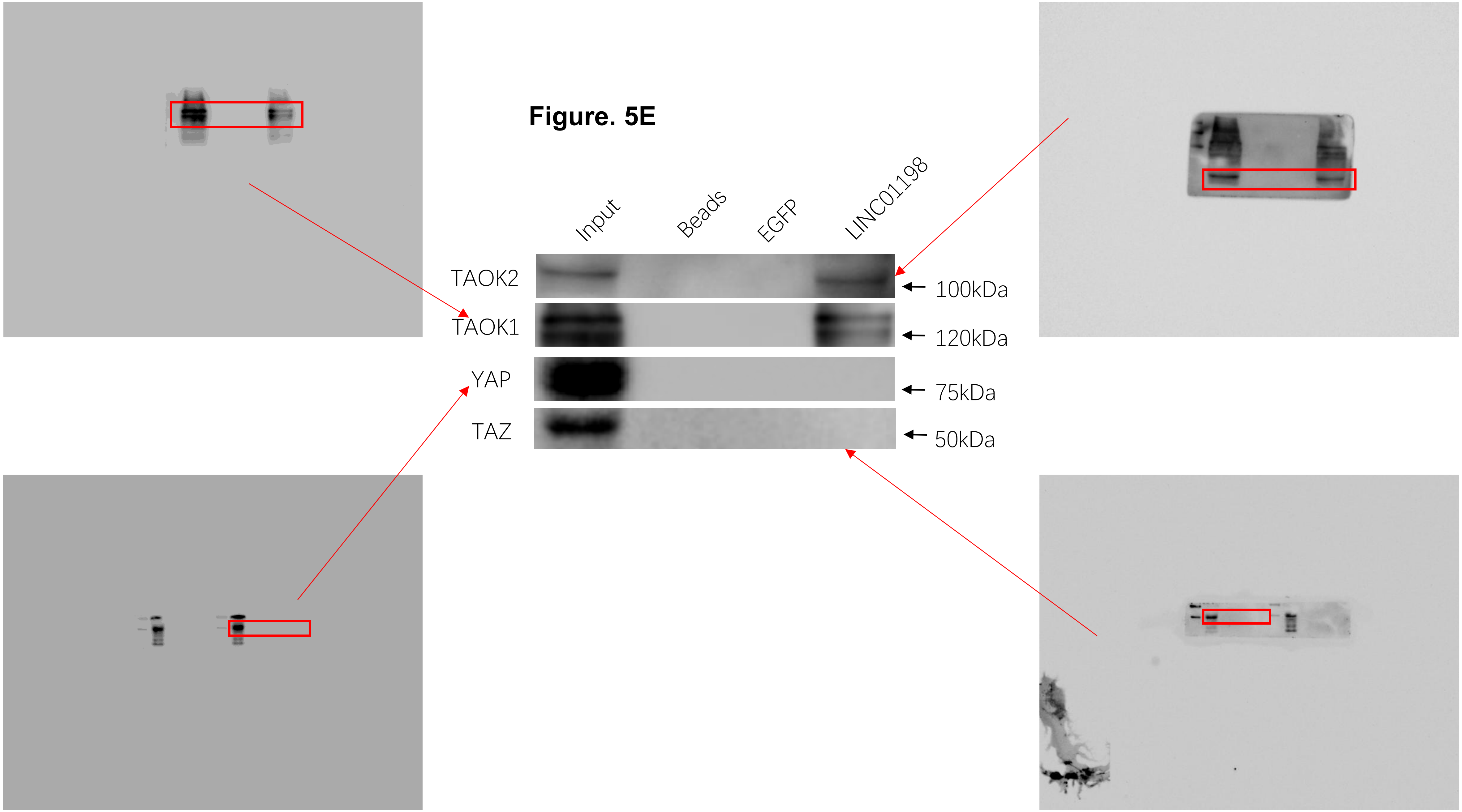
Input
Beads
EGFP
LINC01198
TAOK2
TAOK1
YAP
TAZ
100kDa
120kDa
75kDa
50kDa

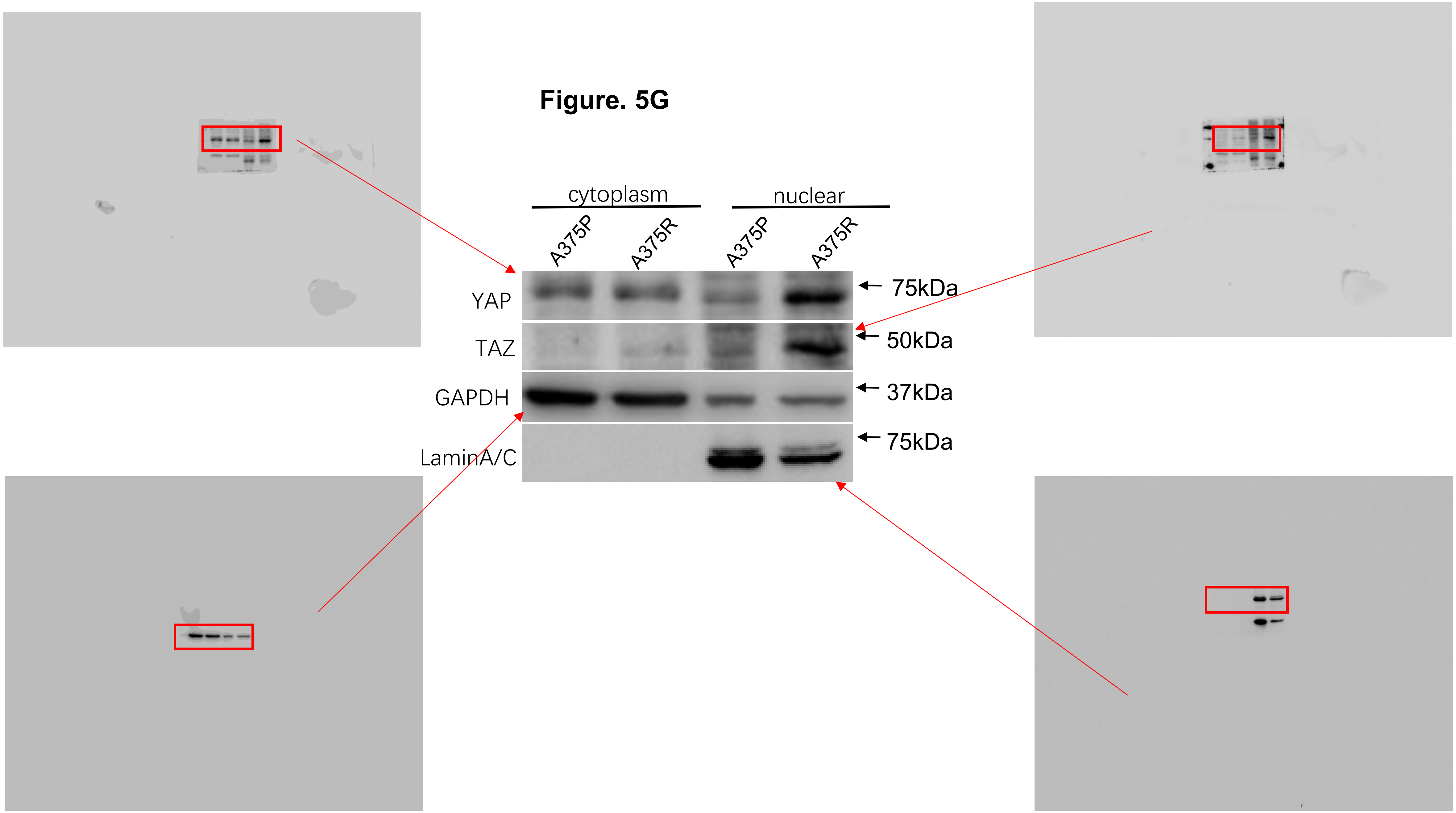
Figure. 5G
cytoplasm
nuclear
A375P
A375R
A375P
A375R
YAP
TAZ
GAPDH
LaminA/C
75kDa
50kDa
37kDa
75kDa

Figure. 5I

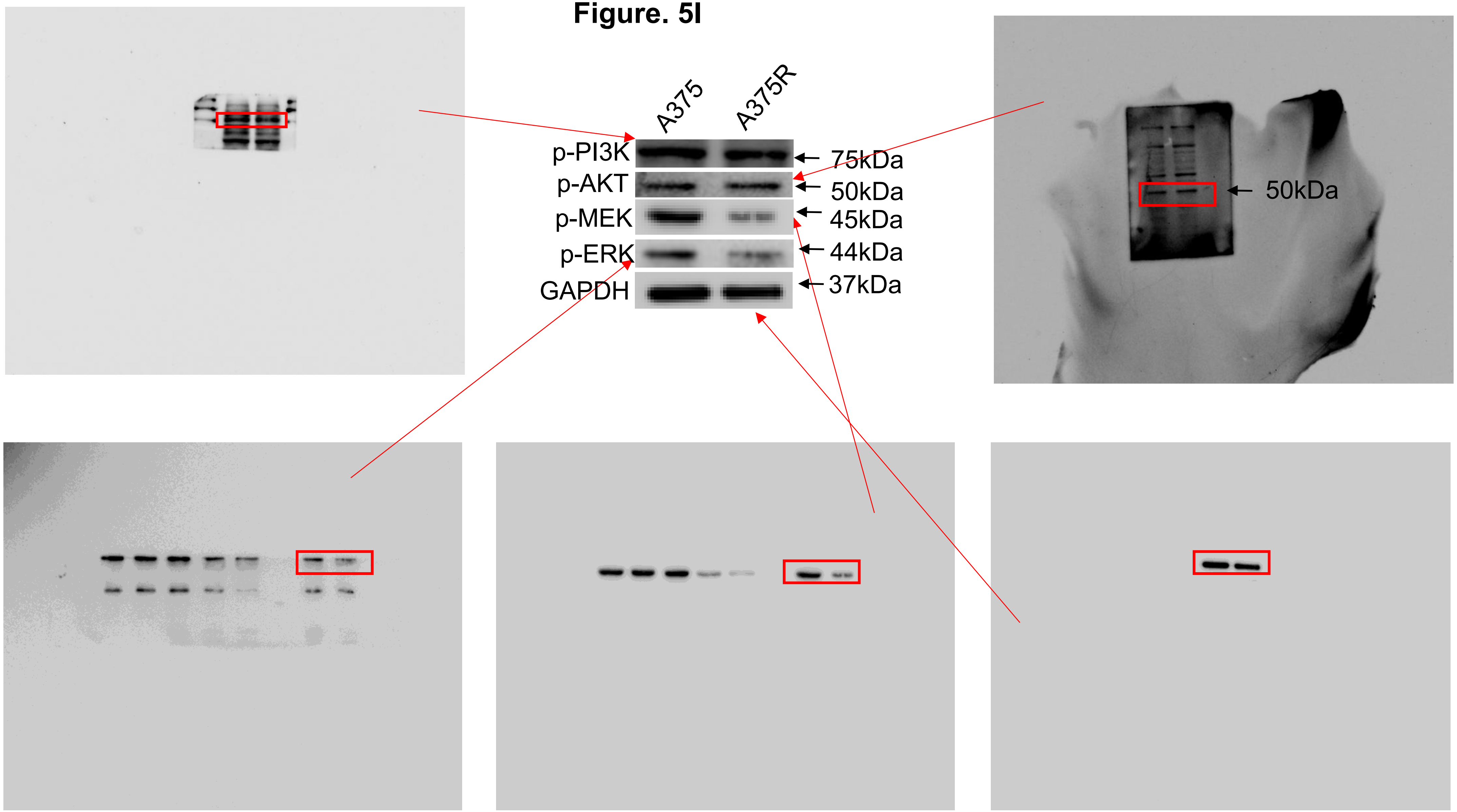
A375
A375R
p-PI3K
p-AKT
p-MEK
p-ERK
GAPDH
75kDa
50kDa
45kDa
44kDa
37kDa
50kDa

Figure. 6A

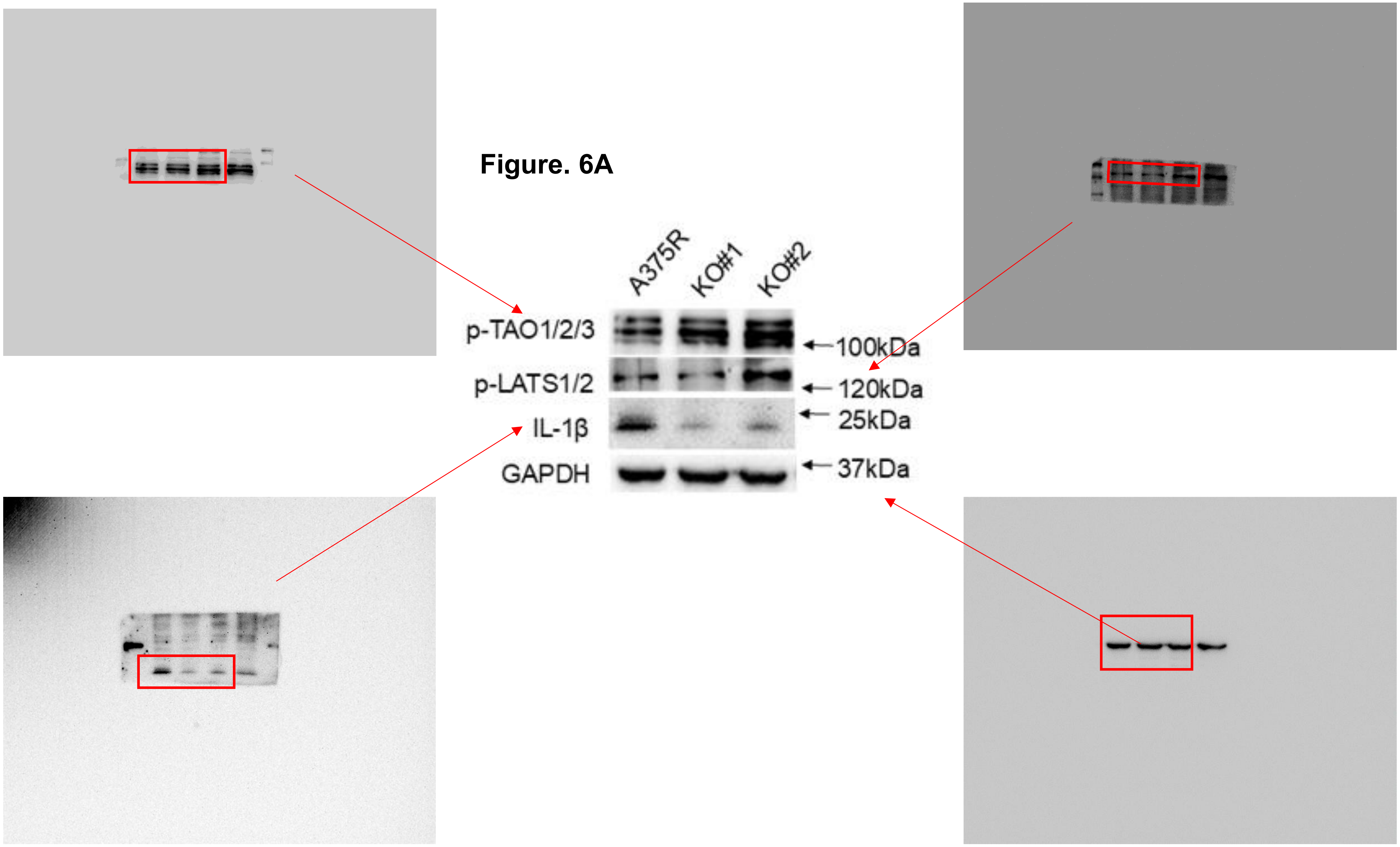
A375R
KO#1
KO#2
p-TAO1/2/3
p-LATS1/2
IL-1β
GAPDH
100kDa
120kDa
25kDa
37kDa

Figure. 6B

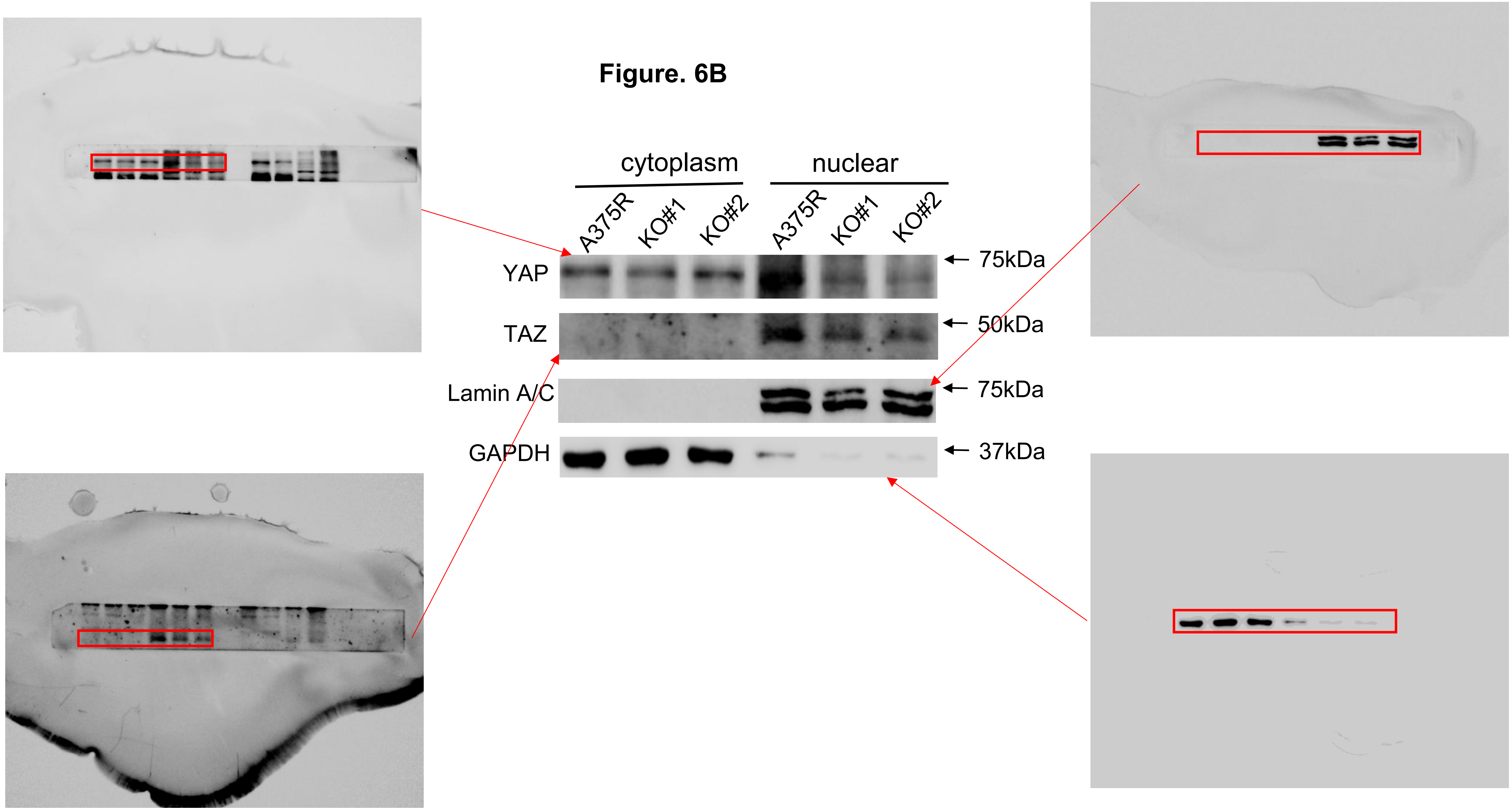
cytoplasm
nuclear
A375R
KO#1
KO#2
A375R
KO#1
KO#2
YAP
TAZ
Lamin A/C
GAPDH
75kDa
50kDa
75kDa
37kDa

Figure. 6D

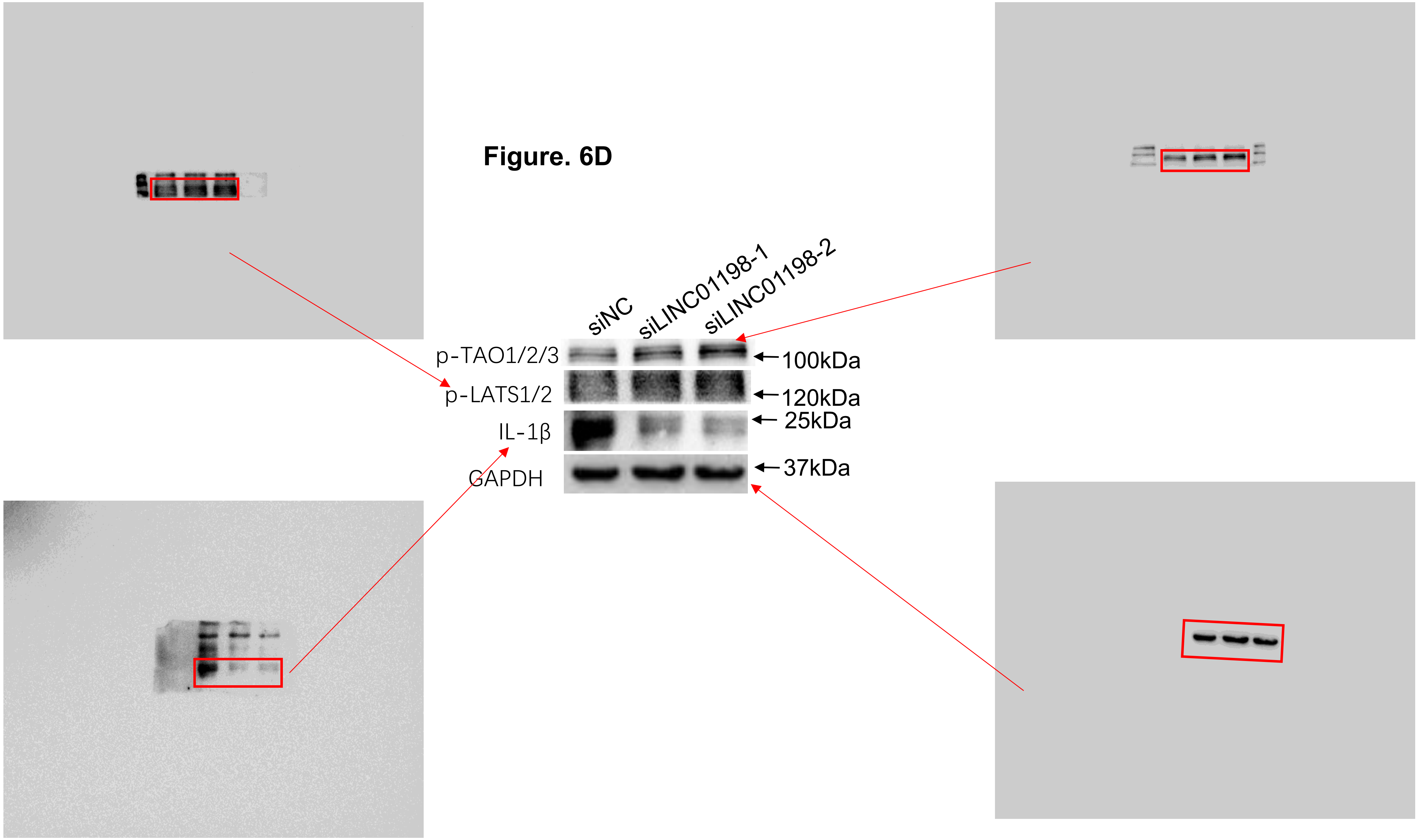
siNC
siLINC01198-1
siLINC01198-2
p-TAO1/2/3
p-LATS1/2
IL-1β
GAPDH
100kDa
120kDa
25kDa
37kDa

Figure. 6E

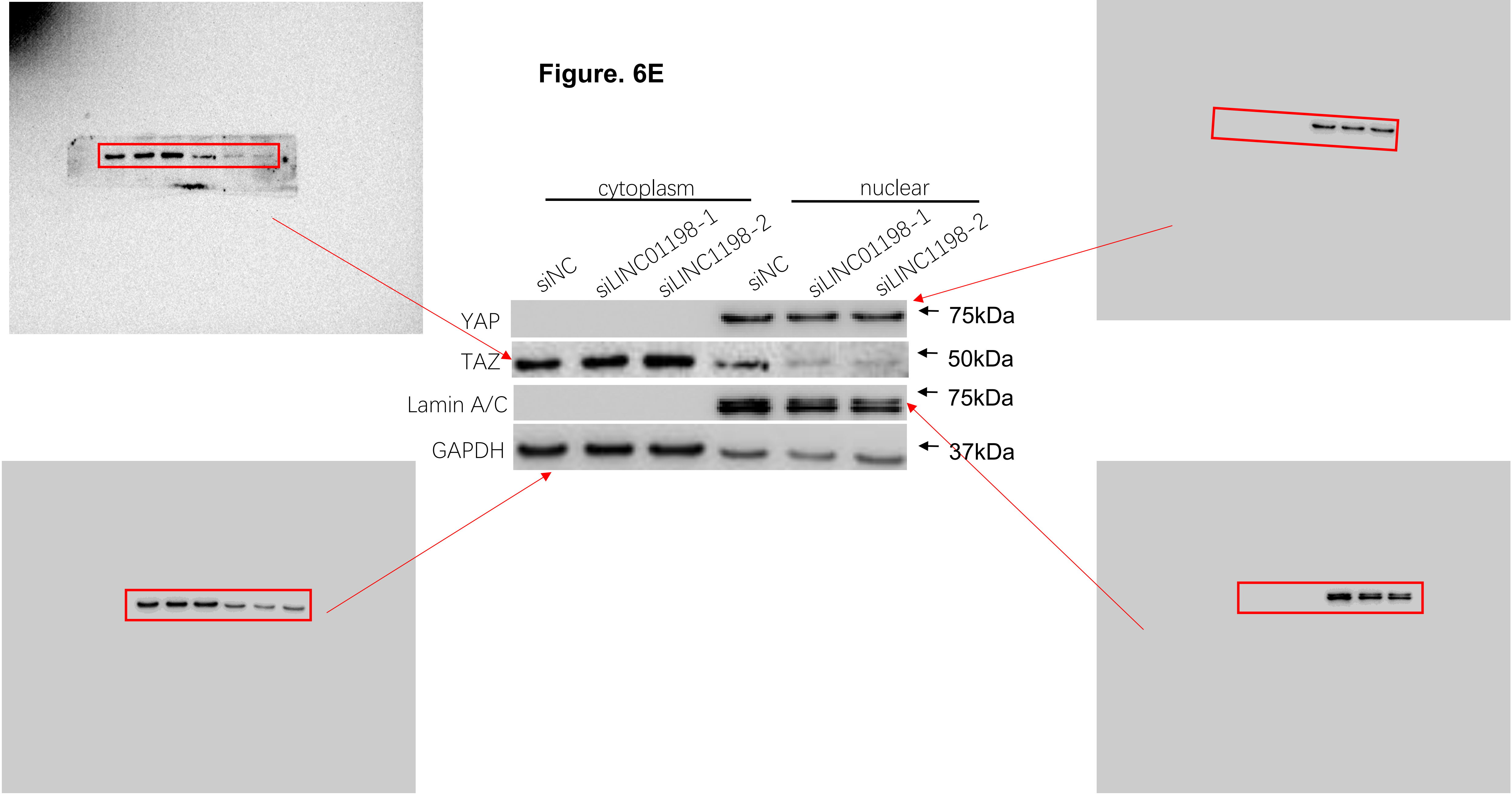

cytoplasm
nuclear
siNC
siLINC01198-1
siLINC1198-2
siNC
siLINC01198-1
siLINC1198-2
YAP
TAZ
Lamin A/C
GAPDH
75kDa
50kDa
75kDa
37kDa

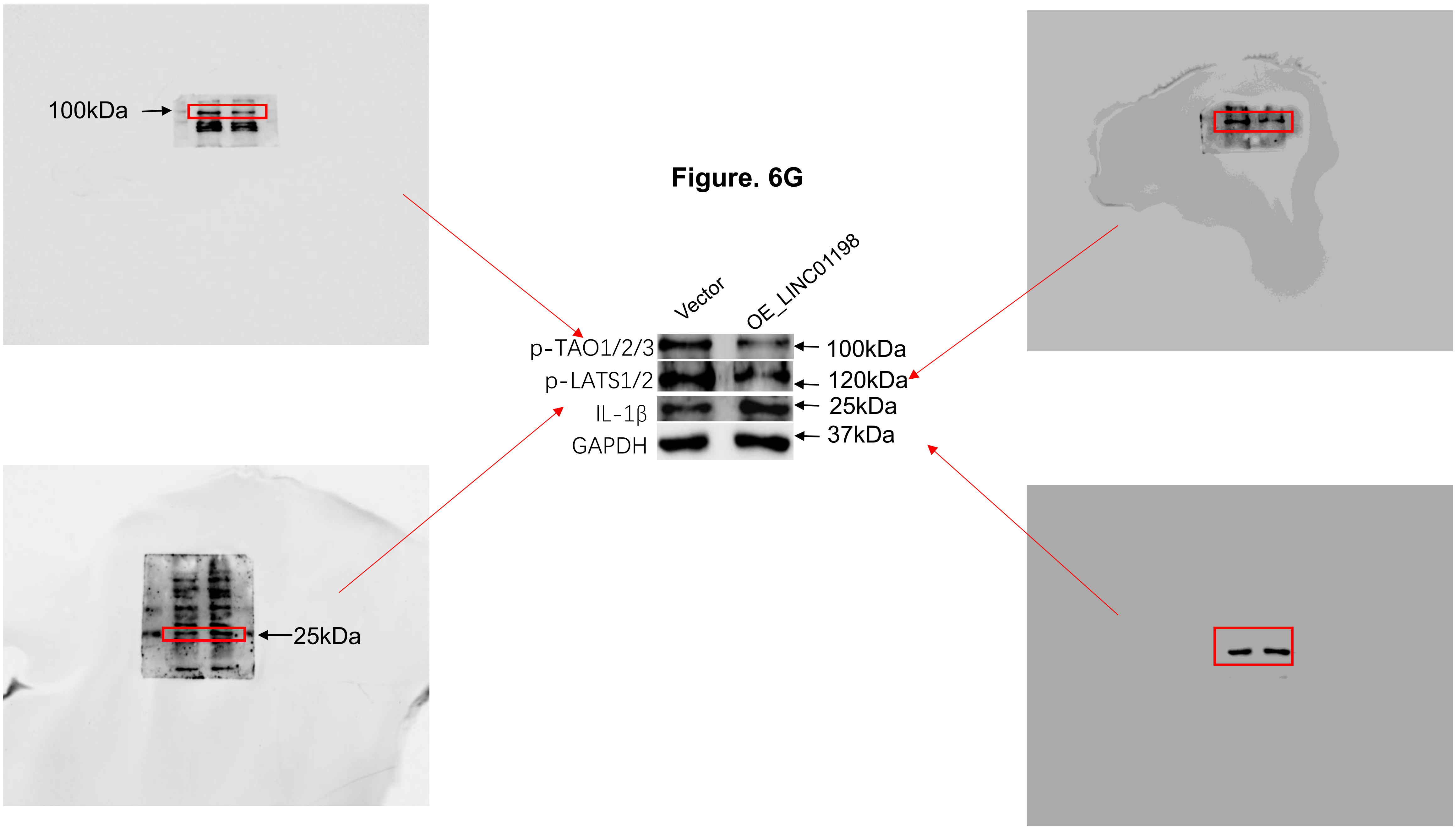
Figure. 6G
100kDa
Vector
OE_LINC01198
p-TAO1/2/3
p-LATS1/2
IL-1β
GAPDH
100kDa
120kDa
25kDa
37kDa
25kDa

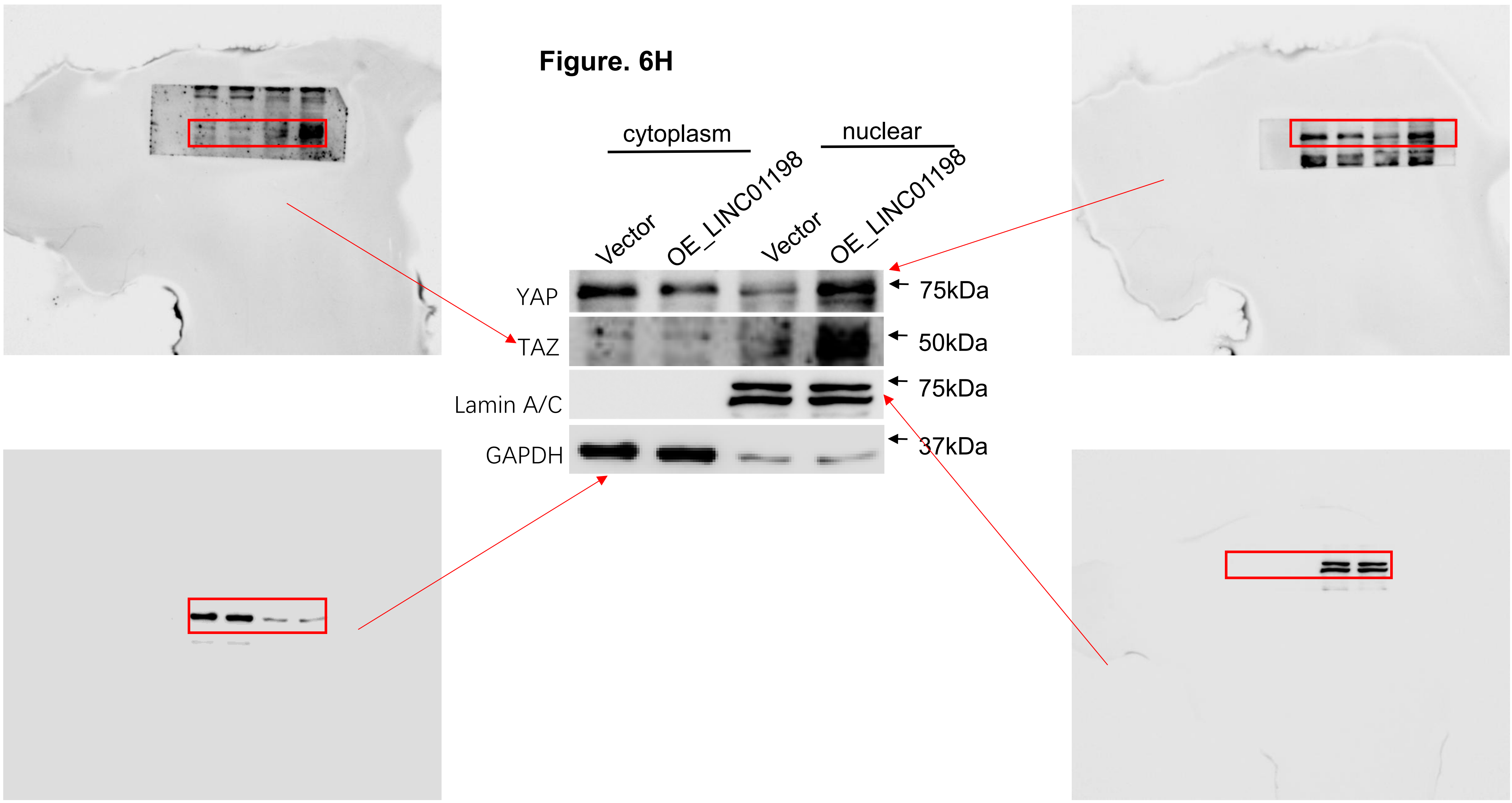
Figure. 6H
cytoplasm
nuclear
Vector
OE_LINC01198
Vector
OE_LINC01198
YAP
TAZ
Lamin A/C
GAPDH
75kDa
50kDa
75kDa
37kDa

Figure. 7B

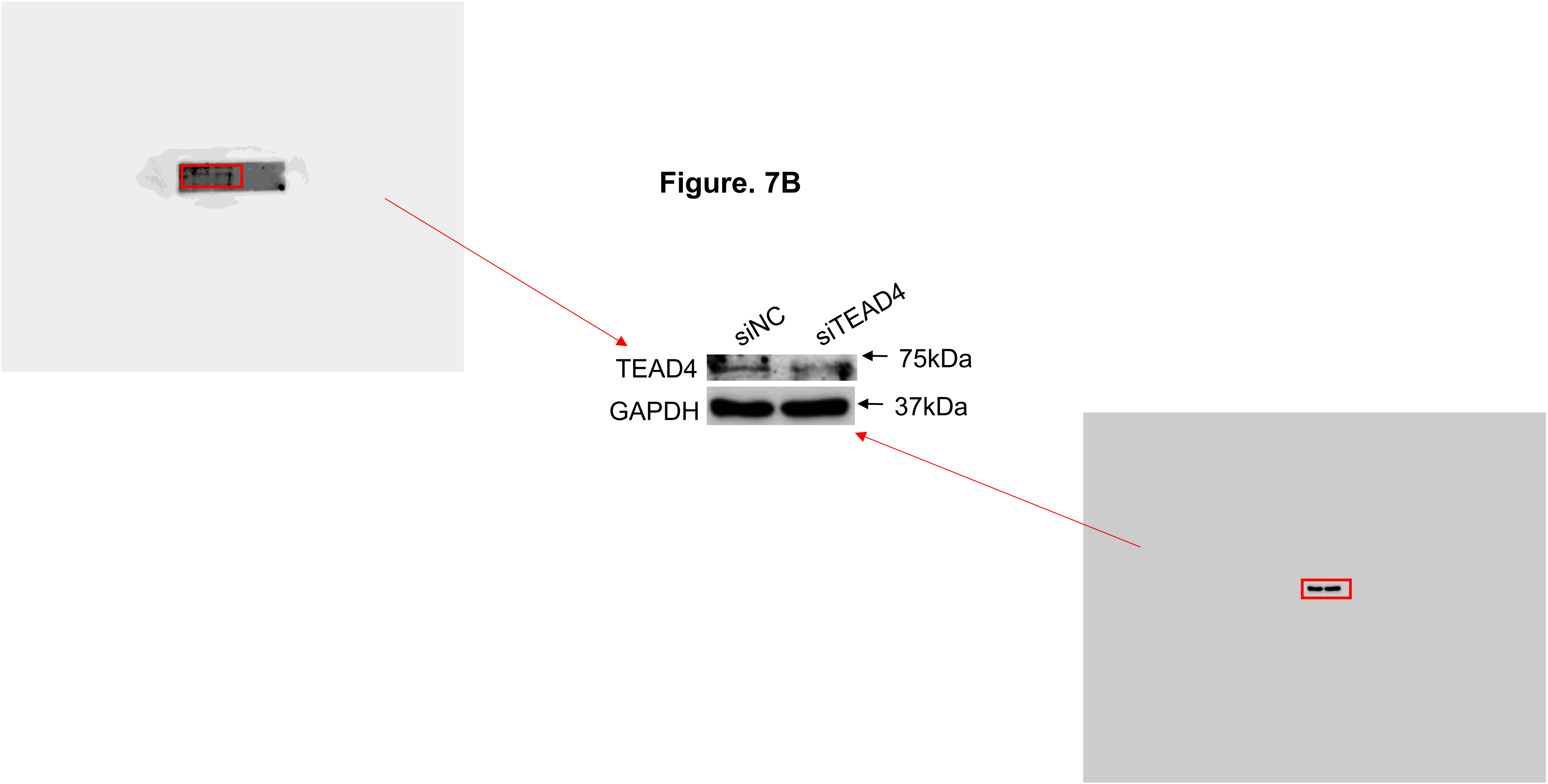

Figure. 7C

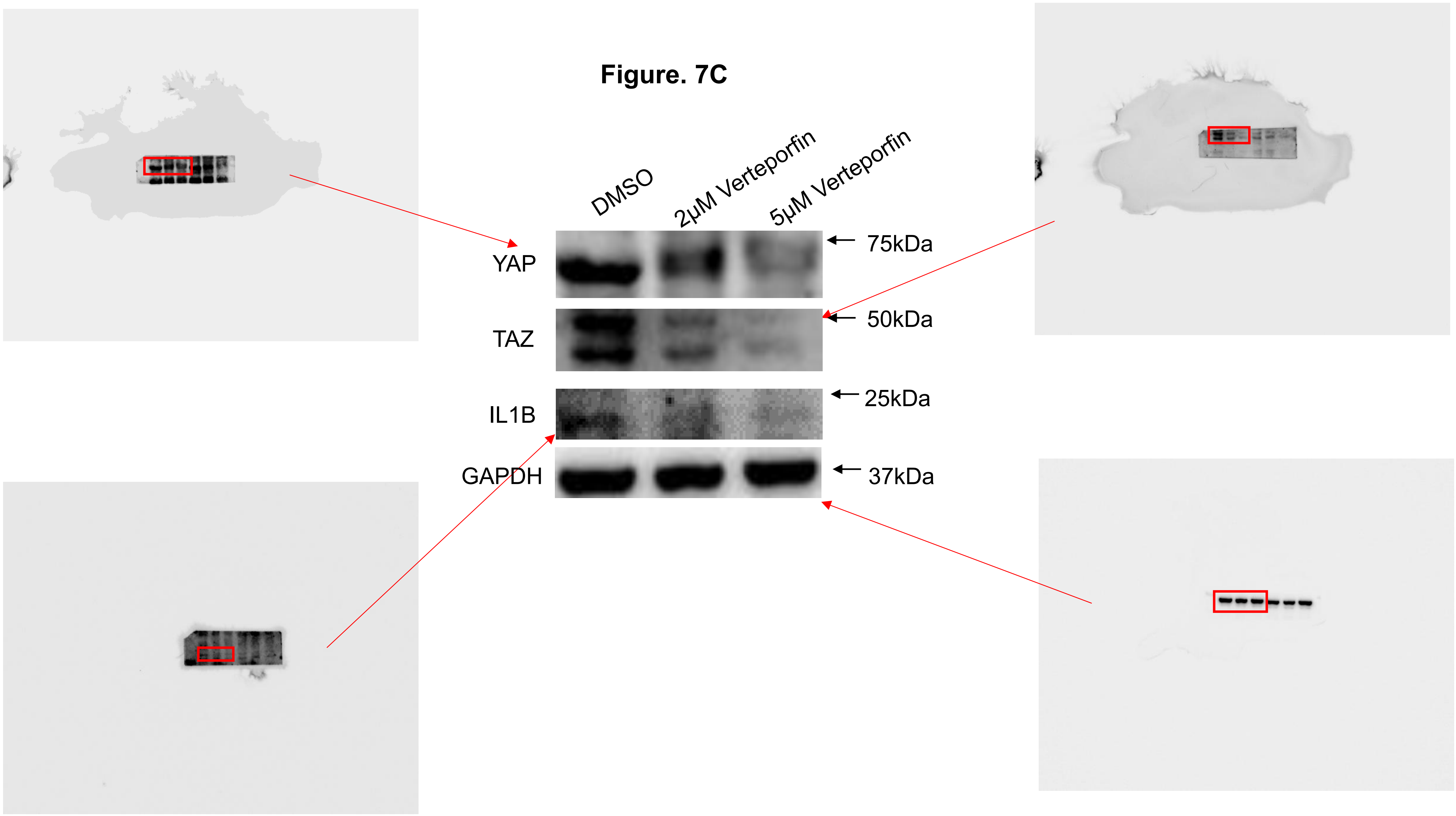
DMSO
2μM Verteporfin
5μM Verteporfin
YAP
TAZ
IL1B
GAPDH
75kDa
50kDa
25kDa
37kDa

Figure. 7E

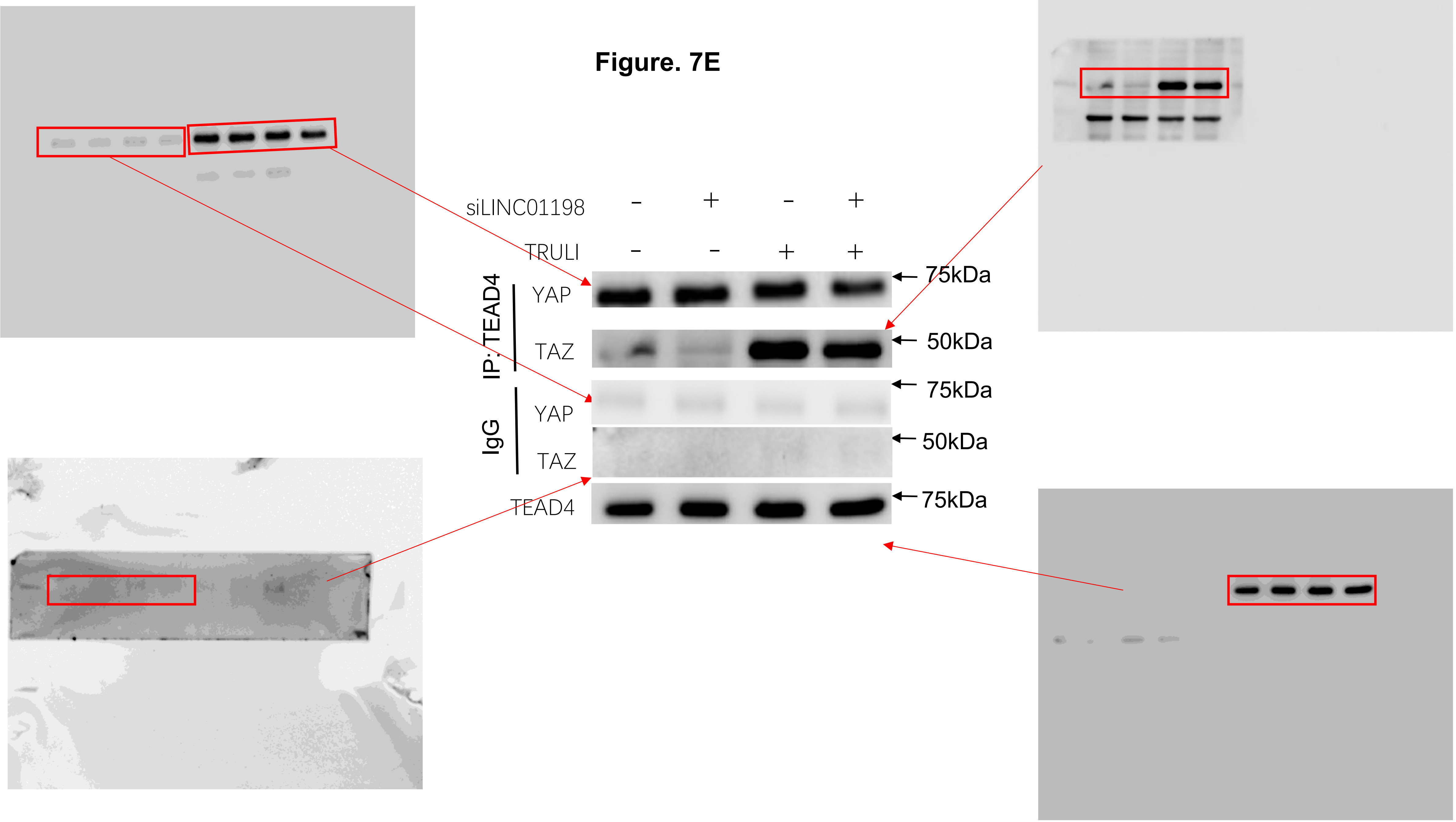
siLINC01198 - + - +
TRULI - - + +
IP: TEAD4
YAP
TAZ
IgG
YAP
TAZ
TEAD4
75kDa
50kDa
75kDa
50kDa
75kDa

Figure. 7E

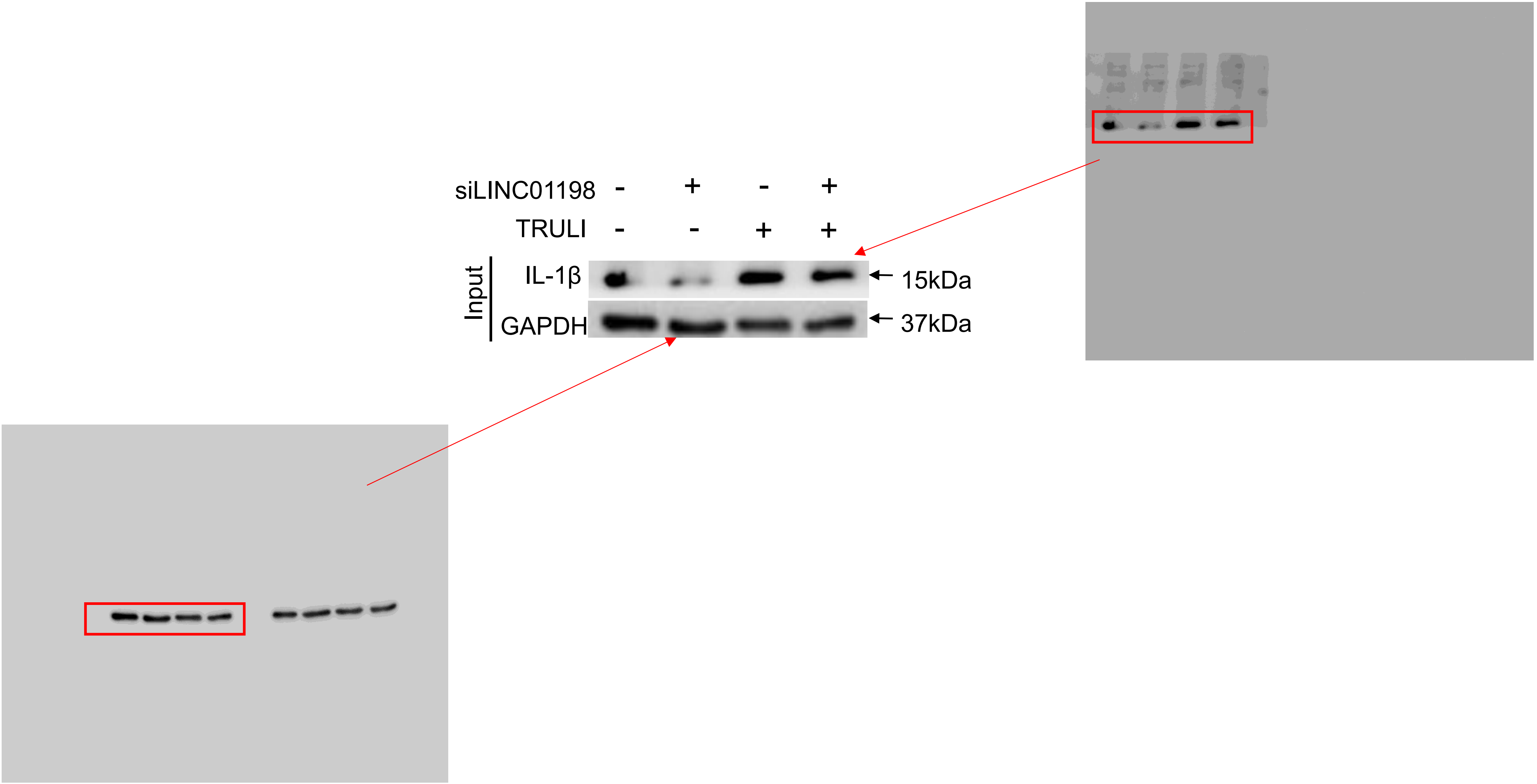

Figure. 7F

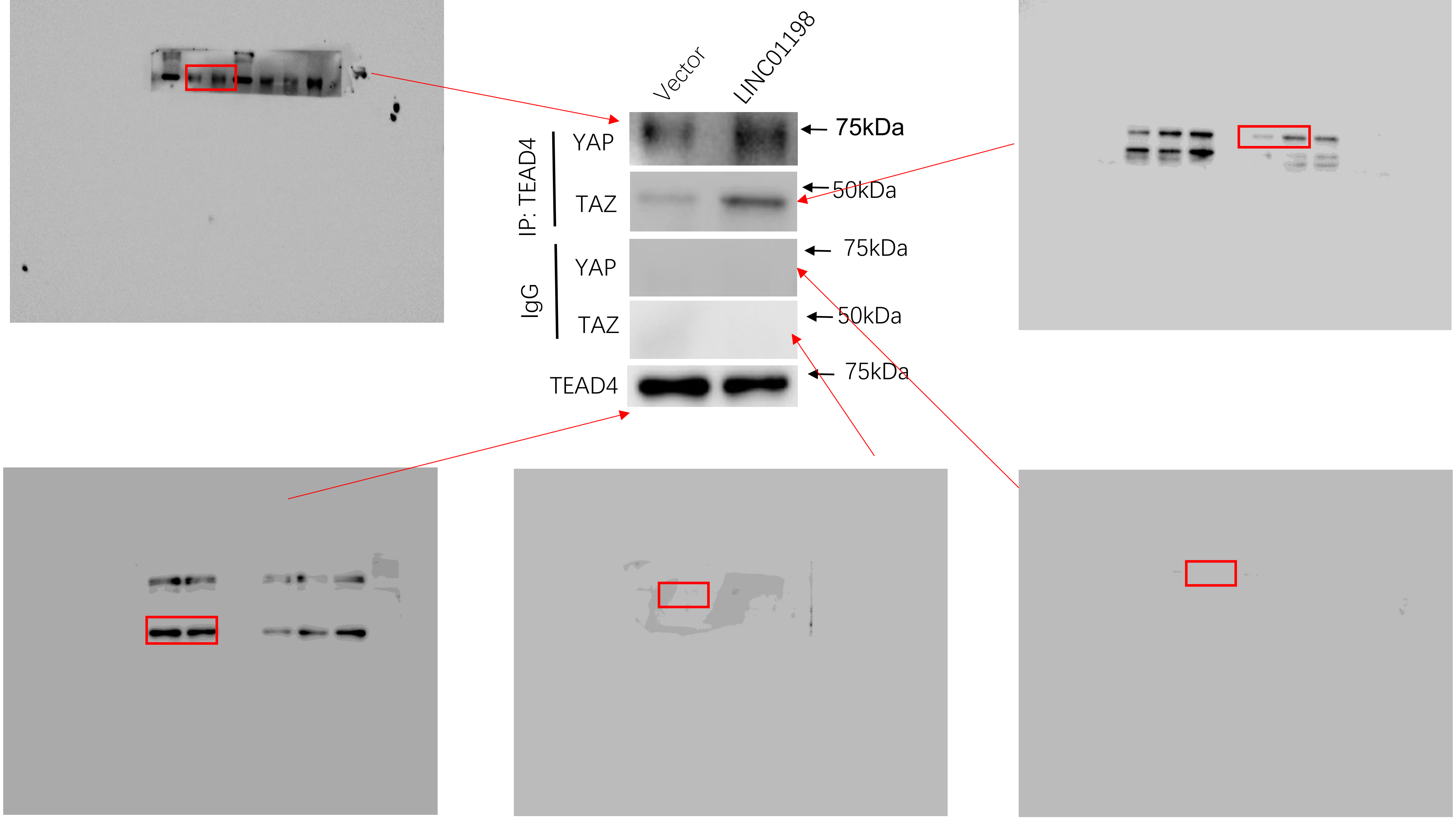

Vector
LINC01198
IP: TEAD4
YAP
TAZ
IgG
YAP
TAZ
TEAD4
75kDa
50kDa
75kDa
50kDa
75kDa

Figure. 7G

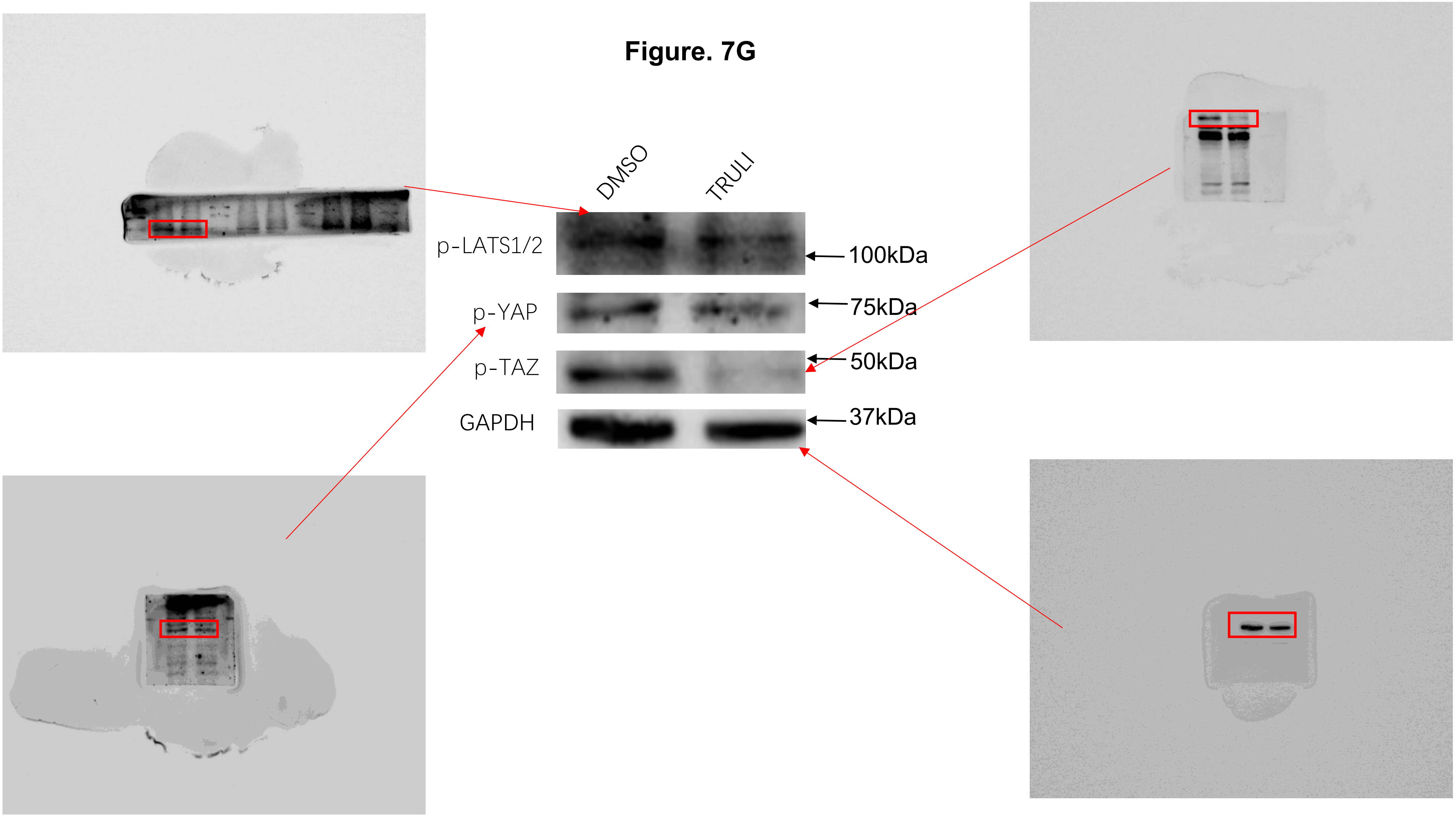
DMSO
TRULI
p-LATS1/2
p-YAP
p-TAZ
GAPDH
100kDa
75kDa
50kDa
37kDa

Figure. 7H

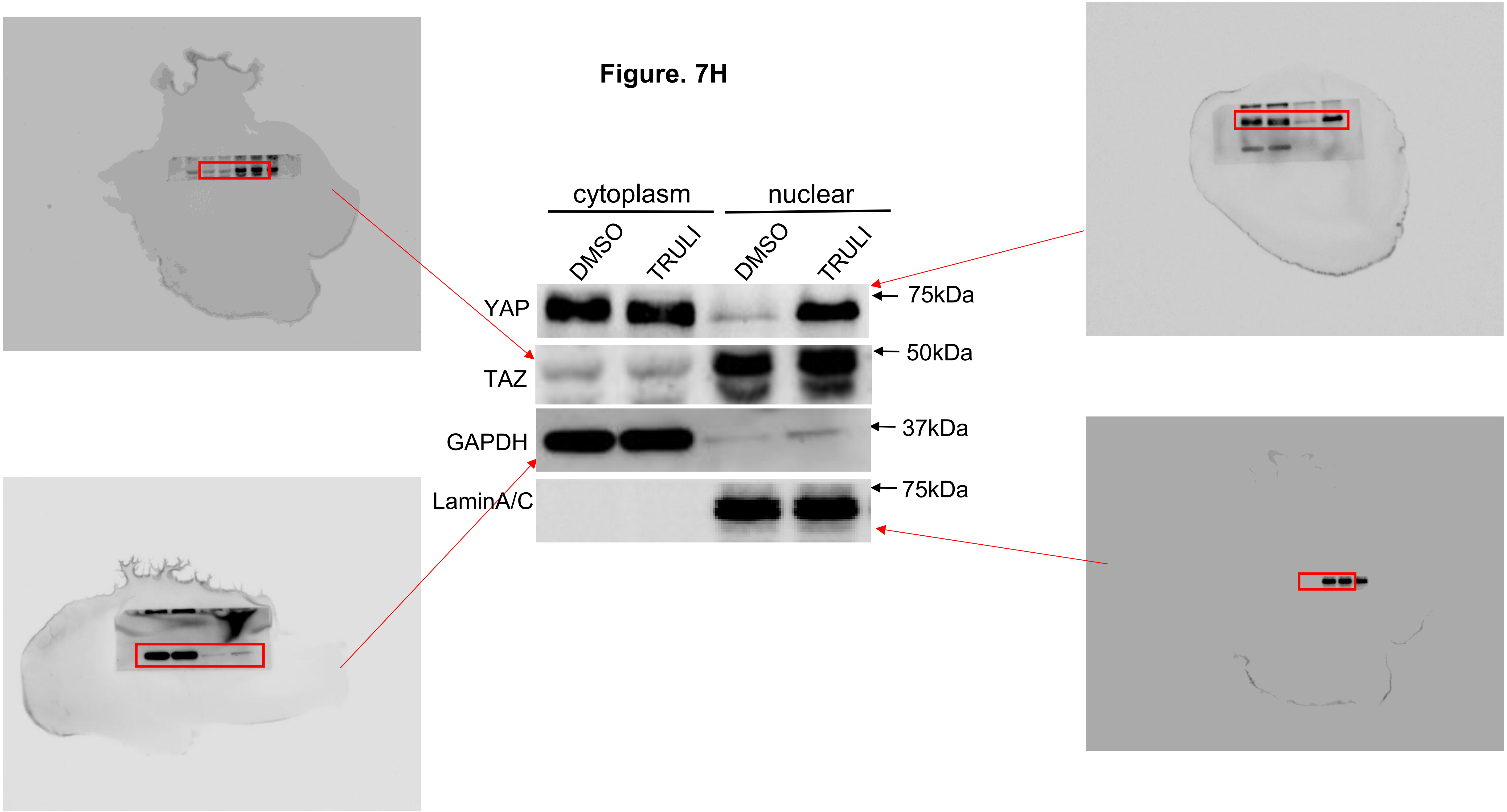

cytoplasm
nuclear
DMSO
TRULI
DMSO
TRULI
YAP
TAZ
GAPDH
LaminA/C
75kDa
50kDa
37kDa
75kDa

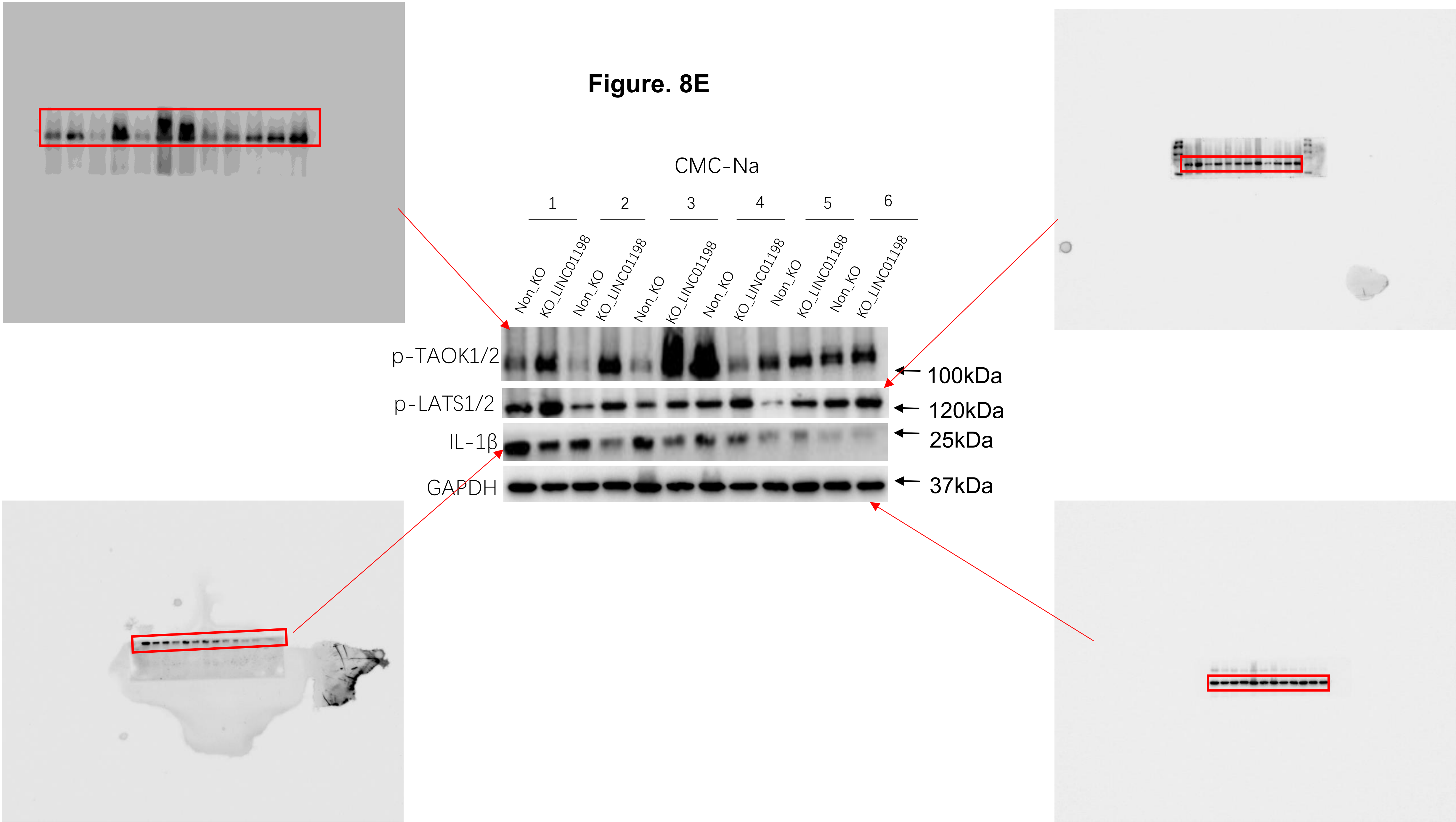
Figure. 8E
CMC-Na
1
2
3
4
5
6
Non_KO
KO_LINC01198
Non_KO
KO_LINC01198
Non_KO
KO_LINC01198
Non_KO
KO_LINC01198
Non_KO
KO_LINC01198
Non_KO
KO_LINC01198
p-TAOK1/2
p-LATS1/2
IL-1β
GAPDH
100kDa
120kDa
25kDa
37kDa

Figure. 8E

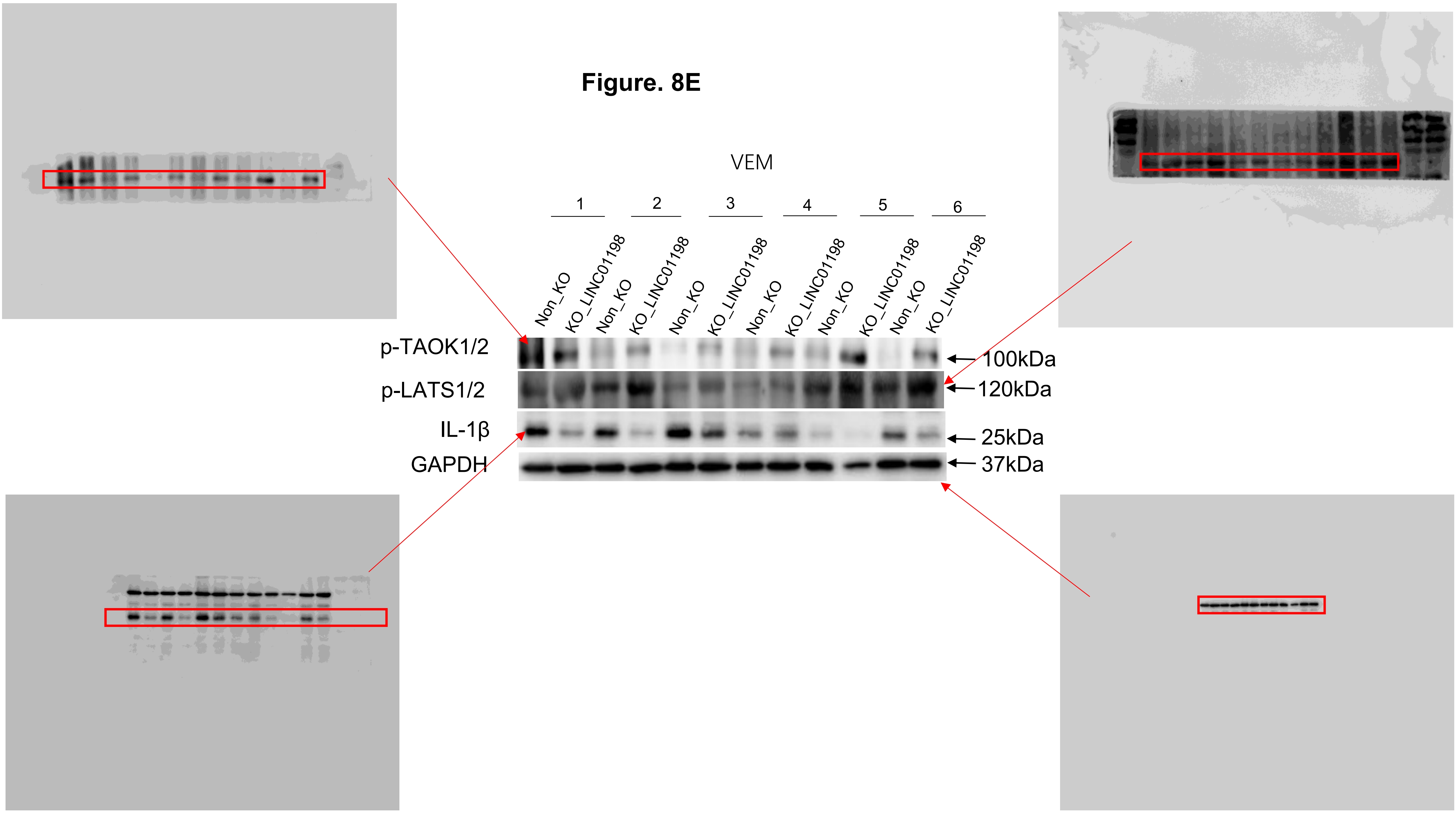
VEM
1
2
3
4
5
6
Non_KO
KO_LINC01198
Non_KO
KO_LINC01198
Non_KO
KO_LINC01198
Non_KO
KO_LINC01198
Non_KO
KO_LINC01198
Non_KO
KO_LINC01198
p-TAOK1/2
p-LATS1/2
IL-1β
GAPDH
100kDa
120kDa
25kDa
37kDa
